# Proceedings of the 27th European Paediatric Rheumatology Congress (PReS 2021): Virtual. 19-21 September 2021

**DOI:** 10.1186/s12969-021-00632-z

**Published:** 2021-11-08

## Lightning talks: Autoinflammatory diseases, Disease outcome and transition, New diseases, Patient/parent organisation initiatives

### O1 Long-term efficacy and safety of canakinumab in patients with mevalonate kinase deficiency: results from the randomized phase 3 cluster trial

#### J. Jeyaratnam^1^, A. Simon^2^, I. Calvo^3^, T. Constantin^4^, A. Shcherbina^5^, M. Hofer^6^, M. Gattorno^7^, A. Martini^8^, B. Bader-Meunier^9^, B. Vastert^10^, J. Levy^11^, E. Dekker^11^, F. de Benedetti^12^, J. Frenkel^1^

##### ^1^Department of Pediatrics, University Medical Center Utrecht, Utrecht; ^2^Department of Internal Medicine, Radboud University Medical Center, Radboudumc Expertise Center for Immunodeficiency and Autoinflammation (REIA), Nijmegen, Netherlands; ^3^Pediatric Rheumatology Unit, Hospital Universitario y Politécnico La Fe, Valencia, Spain; ^4^2nd Department of Pediatrics, Semmelweis University, Budapest, Hungary; ^5^Department of Immunology, Dmitry Rogachev National Medical Center of Pediatric Hematology, Oncology and Immunology, Moscow, Russian Federation; ^6^Unité Centre Multisite Romande d'Immuno-e Rhumatologie Pediatrique, Centre Hospitalier Universitaire Vaudois (CHUV), Lausanne, Switzerland; ^7^Center for Autoinflammatory Diseases and Immunodeficiencies, IRCCS, G. Gaslini, Genova; ^8^University of Genoa, Genoa, Italy; ^9^Department of Pediatric Immunology, Hematology and Rheumatology, Universite de Paris, Institut des Maladies Genetiques (IMAGINE Institute), Reference Centre for Rheumatic, AutoImmune, and Systemic Diseases in Children (RAISE), Necker Hospital, Assistance Publique-Hopitaux de Paris, Paris, France; ^10^Department of Pediatric Immunology, University Medical Center Utrecht, Netherlands; ^11^Novartis Pharma AG, Basel, Switzerland; ^12^Division of Rheumatology, Ospedale Pediatrico Bambino Gesù, Roma, Italy

###### **Correspondence:** J. Jeyaratnam

**Introduction:** Mevalonate Kinase Deficiency (MKD) is a a rare monogenic autoinflammatory disease characterized by fever and generalized inflammation. Evidence-based therapy has become available since canakinumab proved effective to control disease activity and prevent flares.

**Objectives:** In this study we evaluated the long-term efficacy and safety of canakinumab in patients with MKD during the open label extension period (Epoch 4, weeks 41 to 113) of the randomized controlled CLUSTER trial.

**Methods:** Patients received open label canakinumab 150 or 300mg every 4 or 8 (q4 or q8) weeks during the study period of 72 weeks. A stepwise dose increase was maintained if patients experienced a flare. Down-titration was not allowed in Epoch 4.

The disease activity was evaluated every 8 weeks using physician global assessment (PGA) and counting the number of flares. Measurement of C reactive protein (CRP) and serum amyloid A (SAA) protein concentrations were performed. The safety was studied by determination and classification of observed adverse events. The safety and efficacy were analyzed separately in three subgroups of patients receiving a cumulative dose of less than <2700 mg, >=2700-5400mg or >5400 mg

**Results:** Of the 74 MKD patients who started the CLUSTER study, 66 entered Epoch 4 and 65 completed it. Overall, 18 patients received a cumulative dose <2700 mg and 34 patients received 2700-5400 mg, while 14 patients received a cumulative dose of >5400 mg.

At the start of Epoch 4, 19 patients (29%) were receiving the lowest dose regimen (150mg q8) and in 12 (18%) this dose was sufficient to control the disease throughout epoch 4. Another 20 patients (30%) received intermediate doses (150mg q4 and 300mg q8) at the end of the study. However, the highest dose (300mg q4) was required in 32 patients (49%) at the end of Epoch 4, while this regimen was only used to treat 18 patients (27%) at the start.

During the 72-week period, 42 (64%) patients experienced no flares, while 13 (20%) had one flare, as compared with a median of 12 flares per year reported at baseline. At baseline, all patients had mild to severe disease activity according to PGA score. Low PGA scores were seen at the end of the study for all groups with >90% reporting minimal disease activity or none at all. Median CRP concentrations were consistently equal or lower than 10 mg/L. These CRP levels seemed slightly lower in patients receiving the highest cumulative dose (>5400mg). Median SAA concentrations remained only slightly above the normal range of 10mg/L.

The exposure-adjusted rate of adverse events was 2.72 per 100 day. Infection was the most frequently reported class. Twenty-seven serious adverse events were reported in fourteen patients. Some of these serious adverse events were considered to be caused by MKD flares. Eleven serious infections were reported in nine patients (pneumonia (n=3), one each: anal abscess, appendicitis, bronchitis, herpes virus infection, influenza, orchitis, pyelonephritis and tonsillitis).

**Conclusion:** Canakinumab proved effective to control disease activity and prevent flares in MKD during the 72-week study period. Individual dose adjustments may be required to maintain the therapeutic effect of canakinumab. No new or unexpected safety concerns were reported.

**Trial registration identifying number:** NCT02059291

**Patient Consent Received**

Yes

**Disclosure of Interest**

None declared

### O2 Long-term safety of canakinumab in patients with autoinflammatory periodic fever syndromes **−** interim analysis of the reliance registry

#### J. B. Kuemmerle-Deschner^1^, N. Blank^2^, J. Henes^1^, B. Kortus-Goetze^3^, P. T. Oommen^4^, J. Rech^5^, F. Weller-Heinemann^6^, G. Horneff^7^, A. Janda^8^, I. Foeldvari^9^, C. Schuetz^10^, F. Dressler^11^, M. Borte^12^, M. Hufnagel^13^, A. Braner^14^, F. Meier^14^, M. Fiene^15^, J. Weber-Arden^16^, T. Kallinich^17^

##### ^1^University Hospital, Tuebingen; ^2^University Hospital, Heidelberg; ^3^University Hospital, Marburg; ^4^University Hospital, Duesseldorf; ^5^University Hospital, Erlangen; ^6^Prof. Hess Kinderklinik, Bremen; ^7^Asklepios Clinic, Sankt Augustin; ^8^University Hospital, Ulm; ^9^Centre for Pediatric Rheumatology, Hamburg; ^10^University Hospital, Dresden; ^11^Hannover Medical School, Hannover; ^12^Hospital St. Georg gGmbH, Leipzig; ^13^University Hospital, Freiburg; ^14^University Hospital, Frankfurt; ^15^District Hospital, Demmin; ^16^Novartis, Nuernberg; ^17^Charite University Medicine, Berlin, Germany

###### **Correspondence:** J. B. Kuemmerle-Deschner

**Introduction:** Autoinflammatory periodic fever syndromes (PFS) are characterized by severe systemic and organ inflammation. In clinical trials, successful treatment was achieved with the interleukin-1β inhibitor canakinumab (CAN).

**Objectives:** The present study explores the long-term efficacy and safety of CAN in routine clinical practice conditions in pediatric (age ≥2 years) and adult patients with CAPS (cryopyrin-associated periodic syndromes), FMF (familial Mediterranean fever), TRAPS (tumor necrosis factor receptor-associated periodic syndrome) and HIDS/MKD (hyperimmunoglobulinemia D syndrome/mevalonate kinase deficiency).

**Methods:** RELIANCE is a prospective, non-interventional, observational study based in Germany. Patients with clinically confirmed diagnoses of PFS routinely receiving CAN are enrolled. Besides efficacy parameters regarding disease activity and remission, safety parameters were recorded at baseline and assessed at 6-monthly intervals.

**Results:** Here we present the interim analysis of 168 patients with PFS enrolled in the RELIANCE Registry between October 2017 and December 2020. Mean age in this cohort was 24.7 years (2-79 years) and the proportion of female patients was 51 %. At baseline, median duration of prior CAN treatment was 3 years (0-12 years).

A total of 101 patients (60%) experienced any AE and 22 patients (13%) were affected by SAE. In 9 patients (5%) SAE were classified as drug related. Of 489 AE, 53 were severe and a total of 21 SAE were classified as treatment-related (table 1). Overall, 13 AE comprised upper respiratory tract infections (ARI).

**Conclusion:** The interim data from the RELIANCE study, the longest running real-life canakinumab registry for, confirm safety of long-term canakinumab treatment across the entire study population.

**Disclosure of Interest**

J. B. Kuemmerle-Deschner Consultant for: Novartis, AbbVie, Sobi, N. Blank Consultant for: Novartis, Sobi, Lilly, Pfizer, Abbvie, BMS, MSD, Actelion, UCB, Boehringer-Ingelheim, Roche, J. Henes Consultant for: Novartis, AbbVie, Sobi, Roche, Janssen, Boehringer-Ingelheim, B. Kortus-Goetze Consultant for: Novartis, P. T. Oommen: None declared, J. Rech Consultant for: Abbvie, Biogen, BMS, Chugai, GSK, Janssen, Lilly, MSD, Mylan, Novartis, Roche, Sanofi, Sobi, UCB, Speaker Bureau of: Abbvie, Biogen, BMS, Chugai, GSK, Janssen, Lilly, MSD; Mylan, Novartis, Roche, Sanofi, Sobi, UCB, F. Weller-Heinemann: None declared, G. Horneff Speaker Bureau of: AbbVie, Bayer, Chugai, Merck Sharp & Dohme, Novartis, Pfizer, Roche, A. Janda: None declared, I. Foeldvari Consultant for: Novartis, C. Schuetz: None declared, F. Dressler Consultant for: Abbvie, Mylan, Novartis, Pfizer, M. Borte: None declared, M. Hufnagel: None declared, A. Braner Consultant for: Novartis and SOBI, F. Meier Speaker Bureau of: Novartis, M. Fiene: None declared, J. Weber-Arden Employee of: Novartis, T. Kallinich Consultant for: Sobi, Novartis, Roche

**Table 1 (abstract O2).** Overview of the CAN safety data of the RELIANCE study across all study indications (N=168 patients)

Type of event	Number of events	IR^**‡**^
**AE total**	**489**	**173.33**
**AE non-serious**	**436**	**154.33**
**AE, non-serious, not related**	**221**	**78.34**
**AE, ARI**	**13**	**4.61**
**AE, non-serious adverse drug reaction**	**215**	**76.21**
**SAE, total**	**53**	**18.79**
**SAE, not related**	**32**	**11.34**
**SADR**^**#**^**, total**	**21**	**7.44**

^#^Alport's syndrome, appendicitis, blister, cardiovascular disorder, chest pain, circulatory collapse, erythema, febrile convulsion, glomerulonephritis, Hemophilus test positive, pneumonia, premature delivery, skin discoloration, tonsillitis bacterial, tonsillitis streptococcal (each n=1 event, IR 0.4^‡^), tonsillectomy (2 events, IR 0.7^‡^), pyrexia (3 events, IR 1.1^‡^), not yet coded (hospital admission due to exsiccosis upon gastroenteritis, 1 event, IR 0.4^‡^)

^‡^IR, incidence rate per 100 patient years; AE, adverse event; SAE, severe adverse event, SADR, severe adverse drug reaction

### O3 NLRP3 splice variants inactivate caps phenotype in vitro

#### K. Theodoropoulou^1,2^, L. Spel^1^, L. Zaffalon^1^, F. Martinon^1^

##### ^1^Department of Biochemistry, University of Lausanne (UNIL); ^2^Department of Pediatrics, Univerity Hospital of Lausanne (CHUV), Lausanne, Switzerland

###### **Correspondence:** K. Theodoropoulou

**Introduction:** NLRP3 inflammasomes has been associated to the development of autoinflammatory diseases such as Cryopyrin-associated periodic syndromes (CAPS). CAPS comprise a group of rare autoinflammatory diseases, which has recently served as a pure model of IL-1β-driven diseases. However, the mechanisms of inflammasome regulation in these diseases remain unclear. Interestingly, NLRP3 contains Leucine-rich repeats (LRR) domains which are predicted to undergo extensive alternative splicing which is likely to affect the ligand recognition capability.

**Objectives:** The aim of this project is to elucidate the impact of NLRP3 LRR alternative splicing in both physiological state and in systemic autoinflammatory diseases such as CAPS.

**Methods:** N-terminally FLAG-tagged NLRP3 plasmids for every possible LRR skipped exon were generated and subcloned in a doxycyclin inducible vector using Gateway Recombination Cloning technology. All the plasmids were created in 2 different human models: NLRP3 wild type (WT) and NLRP3 CAPS with the R260W mutation. Silencing of NLRP3 in U937 cells, a laboratory monocytic cell line, was performed using CRISPR-Cas9 technique and reconstitution was perfomed with all inducible NLRP3 splice variants by lentiviral transduction. NLRP3 expression and the competence for inflammasome activation (cleavage of IL-1β) were assessed by Western-Blot, in PMA differentiated cells. ASC specks were quantified by ImageStream flow cytometric analysis.

**Results:** Our data show that Δ4, Δ5, Δ7 and Δ9 NLRP3 splice variants lose the competence for inflammasome activation in both physiological and CAPS in vitro models. However, endogenous NLRP3-driven cleavage of IL-1β, GSDMD and Caspase-1 is not affected in unfunctional variants (Δ4, Δ5, Δ7, Δ9), indicating the absence of a dominant negative role of the variants. Moreover, the absence of significant ASC speck formation in unfunctional variants, suggest a functional role of the LRR domain upstream of ASC oligomerization.

**Conclusion:** At this point, our research shows a functional implication of the LRR alternative splicing in NLRP3 inflammasome activation, with some of the LRR exon skipping variants being completely inactive, suggesting a potential regulatory role. However, the exact function of the LRR domain in the cascade of inflammasome activation and how some of its splice variants impairs this process, remain to be elucidated.

**Disclosure of Interest**

None declared

### O4 pGALSplus: a tool to facilitate the identification and assessment of children with serious musculoskeletal disease

#### V. Mercer^1,2^, N. Smith^1^, S. Jandial^3,4^, H. E. Foster^5^

##### ^1^Translational and Clinical Research Institute, Newcastle University, Newcastle upon Tyne; ^2^Children’s Physiotherapy, South Tyneside and Sunderland NHS Foundation Trust, South Shields; ^3^School of Medical Education, Newcastle University; ^4^Paediatric Rheumatology, Great North Children’s Hospital; ^5^Population Health Institute, Newcastle University, Newcastle upon Tyne, United Kingdom

###### **Correspondence:** V. Mercer

**Introduction:** Musculoskeletal (MSK) problems are common, often benign and self-limiting and present to healthcare professionals (HCPs) in the community who may not be MSK specialists; it can therefore be challenging to identify those with serious disease. pGALS (paediatric Gait, Arms, Legs and Spine) is a simple, quick MSK clinical assessment and has been shown to detect joint and functional problems in various MSK conditions including inflammatory arthritis. We aimed to develop an extended pGALS assessment, called pGALSplus, to facilitate identification of children with MSK disease who require onward referral to specialist services.

**Objectives:** To pilot the pGALSplus assessment in CYP with Juvenile Idiopathic Arthritis (JIA), Mucopolysaccharidoses (MPS), Muscular Dystrophy (MD) or Developmental Coordination Disorder (DCD) as exemplar MSK conditions and compare feasibility and acceptability with healthy controls (HC).

**Methods:** A 3-phase mixed methods approach; Phase 1 included a scoping review of the literature and qualitative interviews with expert HCPs within paediatric practice to identify key clinical assessments that inform diagnosis and progress. These results informed the initial ‘pGALSplus’ assessment which underwent iterative development in Phase 2 with an expert working group (including paediatric rheumatologists, expert MSK paediatric physiotherapists and neuromuscular specialists). Phase 3 focused on testing pGALSplus in the exemplar disease groups with feedback from HCPs, patients and carers. Patients; n=37 (JIA;n=10, DCD;n=10, MD;n=9, HC;n=8), age range 2-10 years).

**Results:** Phase 1 data identified key components of pGALSplus to include: The pGALS assessment (‘top to toe’ approach), a questionnaire to identify further indicators of DCD, components of the North Star Ambulatory Assessment (NSAA) to identify early stages of neuromuscular disease (MD), and an assessment of static balance (found to be significantly worse in children with DCD).

In Phase 2 pGALSplus was further expanded to include clinical assessment aiming to identify pain or restriction of range of movement (JIA or MPS), underlying weakness (MD) or issues with motor planning and co-ordination (DCD). The additional tests included; testing reflexes (to assess underlying neurology); leg lengths (which may indicate lower limb joint pathology); activity-based skills including standing from the floor and squatting (MD), hopping, jumping and catching a ball (DCD). Expert consensus derived a colour-coded approach to pGALSplus sequencing to facilitate identification of exemplar MSK conditions.

Phase 3 demonstrated pGALSplus to be quick to complete (mean 12.6 minutes (9 - 20), with high satisfaction from patients and carers (100% ‘about right’ time taken). The assessment was deemed ‘very easy or easy’ for HCPs (35/37, 95%) and patients (32/37, 86%). Parents and children reported high acceptability (32/37, 86% reported it to be ‘very comfortable or with minimal discomfort’).

**Conclusion:** pGALSplus is an evidence and consensus-based tool to discriminate between MSK conditions with high acceptability and feasibility. pGALSplus includes resources to aid HCPs to undertake the assessment. Our aim is that pGALSplus is implemented amongst HCPs in the community who are likely to encounter children early in the clinical pathway and are integral to diagnosis and specialist referral.

**Disclosure of Interest**

None declared

### O5 OAS1 GOF causes a novel autoinflammatory disease characterized by persistently elevated IFN signature, hypogammaglobulinemia, and alveolar proteinosis

#### F. Licciardi^1^, R. Mulatero^1^, M. Dellepiane^1^, C. Covizzi^1^, R. Mogni^1^, L. Baldini^1^, M. Proietti^2^, A. Caballero-Oteyza^2^, A.-L. Lanz^3^, T. Magg^3^, D. Montin^1^, F. Hauck ^3^

##### ^1^Department of Pediatrics and Public Health, Ospedale Infantile Regina Margherita, Turin, Italy; ^2^Institute for Immunodeficiency, University Hospital of Freiburg, Freiburg; ^3^Department of Pediatrics, University Hospital, Ludwig-Maximilians-Universität München, Munich, Germany

###### **Correspondence:** F. Licciardi

**Introduction:** Monogenic autoinflammatory diseases (AIDs) are a group of diseases characterized by dysregulation of innate immune responses. Here we describe a peculiar overlap phenotype of autoinflammation and immunodeficiency in a patient with a *gain of function* (GOF) mutation of oligoadenylate synthetase1 (OAS1). OAS1 is a type I interferon-induced, intracellular dsRNA sensor involved in antiviral defence. Recently OAS1 mutations have been linked with hereditary pulmonary alveolar proteinosis (PAP) and hypogammaglobulinemia but association with autoinflammation has never been reported.

**Objectives:** To describe the clinical phenotype of a patient affected by a novel AID due to monoallelic OAS1 GOF.

**Methods:** The patient, a 13-months old boy, was admitted, 3 weeks after MMR vaccine, for severe cutaneous vasculitis involving cheeks, lips, and nose, acute encephalopathy, and nephrotic syndrome. He had severe hypogammaglobulinemia (IgG 50mg/dl) with absent IgA and IgM. Blood exams ruled out X-linked agammaglobulinemia and severe combined immunodeficiency. He was treated with a IVIG (2g/kg, single dose) plus steroid (dexamethasone 0,6mg/kg/d than shifted to prednisone 2mg/kg); encephalopathy, vasculitis, and nephrotic syndrome slowly regressed.

Three months later, he had a severe acute respiratory insufficiency during Influenza A infection. CT scan showed an ARDS picture with interstitial disease, and areas of consolidation of both lungs. The patient was treated with high dose pulse methylprednisolone (15mg/kg daily for 3 days), followed by oral prednisone with good response. Interferon (IFN) type 1 signature was constantly elevated but NGS panel for interferonopathies failed to show a known mutation. In the following months, IVIG substitution therapy was started due to progressive IgG reduction. Clinical exome revealed a previously published *de novo* monoallelic mutation (c.326G>A, C109Y) in OAS1. The patient recently received a matched unrelated donor hematopoietic stem cell transplantation (HSCT) and during the pre-HSCT evaluation a bronchoalveolar lavage was performed and was suggestive for PAP.

**Results:** Biochemical and functional studies showed that monocytes, macrophages, and B-cells of the patients displayed dsRNA-independent OAS1 GOF that upon IFN-induced expression led to RNase L-mediated RNA-cleavage, translational arrest, and apoptosis. (Magg et al. Science Immunology, accepted).

Clinically, the patient exhibited features reminiscent of SAVI (STING-associated vasculopathy with onset in infancy) such as severe lung involvement, cutaneous vasculitis, and persistently elevated type 1 IFN signature. In contrast to SAVI, he had progressive hypogammaglobulinemia.

**Conclusion:** Monoallelic OAS1 GOF causes a novel AID. The key features of the disease are severe inflammatory flares after virus exposure, persistently elevated IFN signature, progressive PAP, and hypogammaglobulinemia. The deleterious effect of OAS1 GOF mainly affects monocytes, macrophages, and B-cells. HSCT has been already performed with success in other OAS1 GOF patients and at present is the only curative approach (Magg et al. Science Immunology, accepted). Steroids can be a valuable bridging therapy while waiting for HSCT because they might interrupt the vicious cycle induced by IFN-mediated OAS1 upregulation and ensuing IFN production.

**Patient Consent Received**

Yes

**Disclosure of Interest**

None declared

### O6 While looking for one, you may find another: Tin Soldiers and the search for undiagnosed individuals with fibrodysplasia ossificans progressiva (FOP)

#### C. Scott^1^, F. Kaplan^2^, C. Friedman^3^, P. Delai^4^, M. Al Mukaddam^2^, A. Cali^5^, V. Harries^6^, N. Ezra^6^, O. Schwegler^6,7^

##### ^1^Paediatric Rheumatology, University of Cape Town, Cape Town, South Africa; ^2^Departments of Orthopaedic Surgery & Medicine And The Center for Research in FOP & Related Disorders, The Perelman School of Medicine, The University of Pennsylvania, Philadelphia, United States; ^3^Pediatric Oral Health and Dentistry, Schulich School of Medicine and Dentistry, Ontario, Canada; ^4^Instituto de Ensino e Pesquisa, Hospital Israelita Albert Einstein, Sau Paula, Brazil; ^5^Tin Soldiers Global FOP Patient Search, New Jersey, United States; ^6^Tin Soldiers Global FOP Patient Search; ^7^Blink Pictures, Johannesburg, South Africa

###### **Correspondence:** C. Scott

**Introduction:** FOP is an ultra-rare condition where heterozygous, gain-of-function missense mutations in the ACVR1 gene result in progressive heterotopic bone formation in ligaments, tendons and muscles and result in severe disability.^1^ FOP has an estimated incidence of 0.6-1.3 per million individuals ^2,3^ suggesting that currently there are approximately 8,000 patients living with FOP worldwide, however only about 900 patients are currently diagnosed world-wide The diagnosis is made clinically by identification of typical malformations of the great toes as well as inflammatory swellings (flare-ups) that result in progressive and episodic ossification of soft connective tissues, often triggered by trauma.^4^ Muscle biopsies, though contraindicated, are often performed mistakenly during the course of diagnosis, as FOP is not well known. There is a need to identify people with FOP in order to avoid harmful biopsies and provide a pathway to care.

*Tin Soldiers* is a global FOP patient search program utilizing multimedia campaign. The mission is to identify every person with FOP who is currently undiagnosed, as well as to deliver education and support to those living with a diagnosis, but not connected to support networks. Once found, all people living with FOP are connected to pathways to care.

**Objectives:** To describe the *Tin Soldiers* global FOP patient search program approach and report early results of the program.

**Methods:** *Tin Soldiers* creates multimedia campaigns to create awareness and to educate medical professionals, healthcare workers, general public and local communities on FOP. At the heart of the communication program is story-telling of people living with FOP, from a feature-length documentary to public service announcements, animated short films and an 8-part Global Master Series - all designed to bring attention to FOP in order to find patients and provide a pathway to diagnosis and care.

**Results:** Since March 2020, Tin Soldiers has trained 535 medical professionals; established an African Clinicians Council of 10 doctors with the intention of mentoring others across the continent; increased the number of African patients with a diagnosis from 25 patients in December 2020 to 32 in April 2021. Connected previously diagnosed (but not connected) patients to a robust support network and held the first African FOP Family Gathering with clinicians from both South Africa and Nigeria.

On the journey, patients with other conditions have been discovered including Juvenile Idiopathic Arthritis (JIA), Progressive Osseus Heteroplasia (POH) and Multiple Osteochondromas (MO). These patients have been diagnosed and connected to both medical care and patient support. Another important outcome is the continued education of doctors globally with the uptake of the CME Master Series in Russia and planned rollouts in Algeria, Nigeria, Kenya, Namibia, Sweden (in partnership with the national patient organization) and Brazil (under the First Lady’s patronage).

**Conclusion:** *Tin Soldiers* offers an innovative model of patient identification, diagnosis, support and education at all levels of care, using the power of story-telling and multi-media marketing. Such a model could be considered for raising the profile of other musculoskeletal or rare conditions and connecting patients to a functioning pathway to care

**Patient Consent Received**

No

**Disclosure of Interest**

None declared

## Lightning talks: COVID-19 (Coronavirus), Immunodeficiency and infection related arthritis

### O7 Nailfold capillaroscopy: a sensitive method for evaluating microvascular involvement in children with SARS-COV-2 infection

#### F. Çakmak^1^, A. Demirbuğa^2^, D. Demirkol^3^, S. Gümüş^4^, S. Hançerli Torun^2^, G. Kavrul Kayaalp^1^, R. Eker Ömeroğlu^1^, A. Somer^2^, M. Uysalol^4^, R. Yıldız^4^, N. Aktay Ayaz^1^

##### ^1^Pediatric Rheumatology; ^2^Pediatric Infectious Diseases; ^3^Pediatric Intensive Care Unit; ^4^Pediatric Emergency Unit, Istanbul Faculty of Medicine, Istanbul, Turkey

###### **Correspondence:** F. Çakmak

**Introduction:** The coronavirus (SARS-CoV-2) pandemic, known as COVID-19 has spread all over the world in a short period of time and caused the death of more than 2 million people to date. Although in severe cases, it mainly progresses as a serious lung disease such as pneumonia or acute respiratory distress syndrome (ARDS), numerous extrapulmonary manifestations due to systemic hyperinflammation associated with COVID-19 have been described. The hyperinflammatory state and the viral invasion may result in endothelial dysfunction and capillaroscopic examination of the nailfold may be a feasible method for monitoring the microvascular circulation in SARS-CoV-2 infection.

**Objectives:** With this study, we aimed to investigate the microvascular circulation in patients diagnosed with COVID-19 and multisystem inflammatory syndrome in children (MIS-C) by nailfold videocapillaroscopy (NVC).

**Methods:** Thirty-one patients with SARS-CoV-2 infection, 26 of whom were diagnosed with COVID-19 and 6 with MIS-C, and 58 healthy peers were included in the study. All fingers except the thumbs were examined paying greater attention to the ring finger of the non-dominant hand for the presence of any abnormality bilaterally and two images from eight fingers were obtained from both the study and control groups. Sixteen images were examined for the morphology of capillaries, presence of pericapillary edema, microhemorrhage, avascular area, and neoangiogenesis. These parameters were assessed as present or absent, and the presence of signs in at least two fingers was recorded as capillary abnormality in both groups. Capillary length, capillary width, apical loop, arterial and venous width, and intercapillary distance were measured from three consecutive capillaries from the ring finger of the non-dominant hand.

**Results:** COVID-19 patients showed significantly more capillary ramification (p<0.001), capillary meandering (p=0.04), microhemorrhage (p<0.001), neoangiogenesis (p<0.001), capillary tortuosity (p=0.003). Capillary density (p=0.002) and capillary length (p=0.002) were significantly lower in the patient group while intercapillary distance (p=0.01) was significantly longer compared with healthy volunteers. Morphologically, patients with MIS-C had a higher frequency of capillary ramification and neoangiogenesis compared with COVID-19 patients (p=0.04). Patients with capillary abnormalities had significantly higher levels of C-reactive protein (CRP) and D-dimer (CRP; 16.4 *vs* 2.2, p=0.04 and D-dimer; 900 *vs* 340, p=0.04).

**Conclusion:** Children diagnosed with COVID-19 and MIS-C present with several microvascular abnormalities on NVC examination. MIS-C is an emergency phenomenon in which evidence suggests activation of ECs as the key determinant in the pathogenesis of the disease, and NVC may be a useful non-invasive, valid method for assessing the microcirculatory status of children with MIS-C. As a preliminary one, our study may take attention to the use of NVC for follow-up of patients with SARS-CoV-2 infection during clinical course and management.

**Patient Consent Received**

Yes

**Disclosure of Interest**

None declared

### O8 Outcomes of COVID-19 infection among children and young people with pre-existing rheumatic and musculoskeletal diseases

#### L. Kearsley-Fleet^1^, S. Lawson-Tovey^1^, R. E. Costello^1^, A. Belot^2^, F. Aeschlimann^3^, I. Melki^4^, I. Kone-Paut^5^, S. Eulert^6^, N. Švestková^7^, Š. Fingerhutová^7^, D. Clemente^8^, Y. Berkun^9^, Y. Uziel^10,11^, N. M. Wulffraat^12^, B. Raffeiner^13^, F. Oliveira-Ramos^14^, C. Dackhammar^15^, A. Strangfeld^6^, E. F. Mateua^16^, P. M. Machado^17^, K. L. Hyrich^1^

##### ^1^The University of Manchester, Manchester, United Kingdom; ^2^Hospices Civils de Lyon, Lyon; ^3^Hôpital Necker-Enfants Malades; ^4^Hôpital Robert-Debré; ^5^Bicêtre Hospital, Paris, France; ^6^German Rheumatism Research Center, Berlin, Germany; ^7^General University Hospital, Prague, Czech Republic; ^8^Hospital Niño Jesús, Madrid, Spain; ^9^Faculty of Medicine, Hebrew University of Jerusalem, Jerusalem; ^10^Tel Aviv University, Tel Aviv, Israel; ^11^Pediatric Rheumatology European Society; ^12^University Medical Center, Utrecht, Netherlands; ^13^Central Hospital of Bolzano, Bolzano, Italy; ^14^Centro Hospitalar Universitário Lisboa, Lisbon, Portugal; ^15^Sahlgrenska University Hospital, Sahlgrenska, Sweden; ^16^Portuguese League Against Rheumatic Diseases, Lisbon, Portugal; ^17^University College London, London, United Kingdom

###### **Correspondence:** L. Kearsley-Fleet

**Introduction:** It remains unknown whether children and young people with rheumatic and musculoskeletal diseases (RMD) who acquire COVID-19 infection have a more severe COVID-19 course, due to either underlying disease or immunosuppressive treatments.

**Objectives:** To describe outcomes among children and young people with underlying RMD who acquire COVID-19 infection.

**Methods:** All children and young people <19 years of age with COVID-19 (presumptive or confirmed) reported to the EULAR COVID-19 Database, which collects details regarding RMD diagnosis and treatment, COVID infection and outcomes, between 27 March 2020 and 9 April 2021 (cut-off date for this analysis) were included. Patient characteristics and COVID-19 outcomes are presented.

**Results:** A total of 364 children and young people (age range 2-18 years; table) have been reported to the database from 17 countries; mostly France (N=71), Germany (N=71), Czechia (N=59), Spain (N=50), Israel (N=60), and UK (N=25). Most patients had a diagnosis of juvenile idiopathic arthritis (JIA; N=244; 67%). There were 20 (5%) hospitalisations and 1 death reported due to COVID-19. The most commonly reported symptoms were fever (40%) and cough (30%). Only 42 (12%) patients reported glucocorticoid use. Any DMARD therapy was used by 251 (69%) patients; 161 (44%) were on csDMARDs, 119 (33%) on anti-TNF. 40% were in remission at time of COVID-19 infection, 28% in low, and 9% in moderate/high disease activity. Among those with hospitalisation data [N=290], patients on any DMARD therapy (cs/b/tsDMARDs) had similar odds for hospitalisation compared with those not on therapy, adjusted for age, sex, rheumatic disease, and disease severity (odds ratio 1.3; 95% CI 0.3, 4.6).

**Conclusion:** These initial data on outcomes of COVID-19 infection in paediatric RMDs are very reassuring, only one-in-twenty patients were reported to be hospitalised. Due to the database design and inherent reporting bias, this is likely an overestimate, suggesting that overall outcomes among this population appear to be generally good, with mild infection. Increasing case reports to the database will allow further exploration of drug- and disease-specific outcomes.

**Disclosure of Interest**

L. Kearsley-Fleet: None declared, S. Lawson-Tovey: None declared, R. Costello: None declared, A. Belot: None declared, F. Aeschlimann: None declared, I. Melki: None declared, I. Kone-Paut: None declared, S. Eulert: None declared, N. Švestková: None declared, Š. Fingerhutová: None declared, D. Clemente: None declared, Y. Berkun: None declared, Y. Uziel: None declared, N. Wulffraat: None declared, B. Raffeiner: None declared, F. Oliveira-Ramos: None declared, C. Dackhammar: None declared, A. Strangfeld: None declared, E. Mateua: None declared, P. Machado: None declared, K. Hyrich Consultant for: Abbvie

**Table 1 (abstract O8).** See text for description

		All (N=364)
**Gender**	Female	232 (64%)
	Male	131 (36%)
	Unknown	1 (<1%)
**Age, years**	Median (IQR)	14 (10, 17)
**Top Rheumatology Diagnoses**	Juvenile Idiopathic Arthritis (JIA)	244 (67%)
	*Polyarthritis*	*124 (34%)*
	*Oligoarthritis*	*65 (18%)*
	*Systemic*	*23 (6%)*
	*Psoriatic*	*6 (2%)*
	*Enthesitis*	*26 (7%)*
	Autoinflammatory	51 (14%)
	Connective Tissue Disorder	42 (12%)
	Other	27 (7%)
**Comorbidities** *(25 missing)*	None stated	282 (77%)
	Obesity	15 (4%)
	Ocular inflammation	28 (8%)
	Asthma	7 (2%)
**Required Hospitalisation**	Yes	20 (5%)
	No	271 (74%)
	Missing	73 (20%)
**Top Symptoms Reported**	Fever	146 (40%)
	Cough	109 (30%)
	Headache	75 (21%)
	Rhinorrhea	75 (21%)
**Deaths due to COVID-19**	Yes	1 (<1%)
**Treatment at onset of COVID-19 infection**	Glucocorticoids	42 (12%)
	csDMARDs	161 (44%)
	*Methotrexate*	*125 (34%)*
	*Antimalarials*	*22 (6%)*
	*Mycophenolate*	*17 (5%)*
	b/tsDMARDs	161 (44%)
	*Anti-TNF*	*119 (33%)*
	*IL-6*	*21 (6%)*
	*IL-1*	*12 (3%)*
	*JAK*	*2 (<1%)*
	**Any DMARD**	**251 (69%)**
**Disease Activity**	Remission	146 (40%)
	Low	103 (28%)
	Moderate / High	31 (9%)
	Unknown	84 (23%)

### O9 Benchmark analysis for congruencies and discrepancies of multisystem inflammatory syndrome in children, Kawasaki disease and macrophage activating syndrome due to systemic juvenile idiopathic arthritis

#### G. Otar Yener^1^, A. Paç Kısaarslan ^2^, K. Ulu^3^, E. Atalay^4^, F. Haşlak^5^, S. Özdel^6^, B. Bozkaya Yücel^7^, D. Gezgin Yıldırım^8^, F. Çakmak^9^, K. Öztürk^10^, M. Çakan^11^, Z. Balık^4^, C. Hasbal^12^, M. Yıldız^5^, T. Erat^13^, B. S. Çetin^14^, M. Yılmaz^15^, E. Bağlan^6^, S. Laçinel Gürlevik^16^, V. Atasayan^17^, S. G. Karadağ^18^, E. D. Batu^4^, A. Adrovic^5^, S. Çağlayan^3^, A. Tanatar^9^, F. Demirkan^9^, T. Coşkuner^3^, Ö. Akgün^9^, M. Kasap Cüceoğlu^4^, G. Kavrul Kayaalp^9^, S. Şahin^5^, Ö. Başaran^4^, F. Demir^3^, K. Barut^5^, D. Gürses^15^, A. Baykan^19^, Y. Özsürekçi^16^, H. E. Sönmez^20^, Y. Bilginer^4^, N. Aktay Ayaz^9^, Ö. Aydoğ^7^, S. Yüksel^21^, B. Sözeri^3^, Ö. Kasapçopur^5^, S. Özen^4^

##### ^1^Pediatric Rheumatology, Şanlıurfa Research and Training Hospital, Şanlıurfa; ^2^Pediatric Rheumatology, Erciyes University, Faculty of Medicine, Kayseri; ^3^Pediatric Rheumatology, University of Health Sciences, Ümraniye Research and Training Hospital, İstanbul; ^4^Pediatric Rheumatology, Hacettepe University, Faculty of Medicine, Ankara; ^5^Pediatric Rheumatology, Istanbul University-Cerrahpaşa, Cerrahpaşa Faculty of Medicine, Istanbul; ^6^Pediatric Rheumatology, University of Health Sciences, Dr. Sami Ulus Maternity and Child Health and Diseases Research and Training Hospital, Ankara; ^7^Pediatric Rheumatology, Ondokuz Mayis University, Faculty of Medicine, Samsun; ^8^Pediatric Rheumatology, Diyarbakır Training and Research Hospital, Diyarbakır; ^9^Pediatric Rheumatology, Istanbul University, Faculty of Medicine; ^10^Pediatric Rheumatology, Istanbul Medeniyet University, Göztepe Prof. Dr. Süleyman Yalçın City Hospital; ^11^Pediatric Rheumatology, University of Health Sciences, Zeynep Kamil Women and Children’s Diseases Training and Research Hospital; ^12^Pediatrics, University of Health Sciences, Ümraniye Research and Training Hospital, Istanbul; ^13^Pediatric Infectious Diseases, Şanlıurfa Research and Training Hospital, Şanlıurfa; ^14^Pediatric Infectious Diseases, Erciyes University, Faculty of Medicine, Kayseri; ^15^Pediatric Cardiology, Pamukkale University, Faculty of Medicine, Denizli; ^16^Pediatric Infectious Diseases, Hacettepe University, Faculty of Medicine, Ankara; ^17^Pediatric Cardiology, University of Health Sciences, Ümraniye Research and Training Hospital, Istanbul; ^18^Pediatric Rheumatology, Erzurum Regional Research and Training Hospital, Erzurum; ^19^Pediatric Cardiology, Erciyes University, Faculty of Medicine, Kayseri; ^20^Pediatric Rheumatology, Kocaeli University, Faculty of Medicine, Kocaeli; ^21^Pediatric Rheumatology, Pamukkale University, Faculty of Medicine, Denizli, Turkey

###### **Correspondence:** G. Otar Yener

**Introduction:** Fever and certain manifestations are comparable in patients with Multisystem inflammatory syndrome in children (MIS-C) and Kawasaki disease (KD), whereas the cytokine storm reflected in the laboratory findings of patients with MIS-C resemble macrophage activating syndrome (MAS).

**Objectives:** The aim of the study was to compare the clinical and laboratory findings of multisystem inflammatory syndrome in children (MIS-C) patients with Kawasaki disease (KD) and with macrophage activating syndrome due to systemic juvenile idiopathic arthritis (sJIA-MAS) on real-life data.

**Methods:** Patients diagnosed with MIS-C, KD, and sJIA-MAS from 12 different centers in Turkey were included in the study.

**Results:** A total of 154 MIS-C, 59 KD, and 31 sJIA-MAS patients were included in the study. The median age of patients with MIS-C were higher than those with KD while lower than those with sJIA-MAS (p < 0.001). Myalgia, cardiac, gastrointestinal, and neurological involvements were more common in patients with MIS-C compared to others. Arthritis, hepatomegaly and splenomegaly were more common in patients with sJIA-MAS compared to MIS-C. Myocarditis was a distinctive feature in patients with MIS-C (n=39) compared to patients with KD (n=0) and sJIA-MAS (n=4) (p < 0.001). MIS-C patients had lower levels of lymphocyte and thrombocyte counts and higher pro-BNP levels than those with KD. The median level of CRP was higher in patients with MIS-C but ferritin levels were higher in patients with sJIA-MAS. However, patients with MIS-C had higher levels of ferritin compared to patients with KD. Patients with MIS-C had a shorter duration of hospitalization than sJIA-MAS while they required intensive care unit admission more frequently due to myocarditis.

**Conclusion:** MIS-C patients displayed certain differences in clinical and laboratory features when compared to KD and MAS due to sJIA. Correct diagnosis with a multidisciplinary approach and appropriate management will suppress systemic inflammation and may prevent morbidity and mortality in MIS-C patients.

**Disclosure of Interest**

None declared

### O10 SARS-COV2 antibody phenotype and immune gene expression in MIS-C

#### K. Webb^1,2^, T. Moyo-Gwete^3,4^, S. C. Mendelsohn^5^, C. Butters^1^, S. Richardson^3,4^, H. Facey-Thomas^1^, D. Abrahams^1^, M. Madzivhandila^3,4^, Z. Makhado^3,4^, N. Manamela^3,4^, F. Ayres^3,4^, R. Baguma^5^, S. Kimbung Mbandi^5^, M. Erasmus^5^, L. Zühlke^6^, T. J. Scriba^5^, P. L. Moore^3,4,7^, G. Kassiotis^2^, C. Scott^1^

##### ^1^Paediatric Rheumatology, University of Cape Town, Cape Town, South Africa; ^2^Retroviral Immunology, Francis Crick Institute, London, United Kingdom, ^3^National Institute for Communicable Diseases; ^4^Antibody Immunity Research Unit, University of the Witwatersrand, Johannesburg; ^5^South African Tuberculosis Vaccine Initiative, Institute of Infectious Disease and Molecular Medicine; ^6^Paediatric Cardiology, University of Cape Town, Cape Town; ^7^Centre for the AIDS Programme of Research in South Africa (CAPRISA), Durban, South Africa

###### **Correspondence:** K. Webb

**Introduction:** Multisystem Inflammatory Syndrome in Children (MIS-C) is a severe disease that affects a small proportion of children exposed to Severe Acute Respiratory Syndrome Coronavirus 2 (SARS-CoV-2). Differences in SARS-CoV-2 antibody responses and immune gene expression between SARS-CoV-2-infected children who develop MIS-C and those who do not may provide insight into the mechanism of MIS-C.

**Objectives:** To determine the difference in SARS-CoV2 antibody responses and immune gene expression in children with MIS-C and healthy children with evidence of previous SARS-CoV2 infection.

**Methods:** Healthy children presenting for elective surgery and those with MIS-C were recruited between 22 June 2020 and 5 November 2020 from a single paediatric hospital during the first wave of SARS-CoV-2 in the region. Clinical data, whole blood RNA and serum were collected. Titres of SARS-CoV-2 spike-specific antibody (SAb) and their capacity to perform neutralization, antibody-dependent cellular phagocytosis (ADCP) and antibody dependant cellular cytotoxicity (ADCC) were measured. Whole blood RNA gene expression was measured using multiplex Fluidigm quantitative Polymerase Chain Reaction (qPCR) with a panel of 84 immune genes. Principal component analysis was performed to assess for differences in gene expression. A linear regression model was developed with a forward stepwise model selection method to assess which genes associated with C-reactive protein (CRP) in MIS-C after controlling for the neutrophil to lymphocyte ratio (NLR).

**Results:** Twenty-three children with MIS-C and 25 healthy children were recruited. Nine healthy children had detectable SARS-CoV-2 serum antibodies (healthy exposed). No children had preceding clinical disease related to SARS-CoV-2 infection. Comparing children with MIS-C and healthy exposed children showed no difference in SAb binding responses (p=0.372) or ADCC (p=0.992). Increased neutralisation titre (p=0.084) and ADCP (p=0.086) in children with MIS-C was observed although was non-significant. Antibody function or titre did not change over time or with treatment in MIS-C. There was a clear distinction in immune gene expression between healthy children and those with MIS-C. Immune gene expression in MIS-C resolved to become indistinct from healthy children with time. Whole blood immune gene expression associated with an abundance of neutrophils in MIS-C. In a model that accounted for 66% of the variance in CRP (adjusted R^2^ = 0.66) the expression of *IL27* accounted for 64% of the model effect (B=35; p<0.001) followed by NLR (15%, B=6.6, p=0.002) and the expression of *MCP2* (11%, B=-14.59, p=0.008).

**Conclusion:** Comparing children infected with SARS-COV-2 from the same time period and region with or without MIS-C provides unique mechanistic insight into the disease. A trend towards higher SAb titres and ADCP implies a distinct humoral immune response to SARS-COV-2 in children with MIS-C, although further studies are required to validate this observation. The resolution of the abnormal immune gene expression in MIS-C implies a monophasic immune perturbation. The association of *IL27* and *MCP2* with CRP suggests that these may be important targets in future studies for possible pathogenicity and as potential biomarkers in MIS-C.

**Patient Consent Received**

Yes

**Disclosure of Interest**

None declared

### O11 22Q11.2 deletion (DI George) syndrome and chronic arthritis. An international case series of 21 cases

#### C. Freychet^1^, T. Giani^2^, M. Jelusic^3^, B. Bader Meunier^1^, J. L. Stephan^4^, I. Lemelle^5^, D. Montin^6^, L. Mc Cann^7^, D. McDonald-McGinn^8^, T. B. Crowley^9^, R. Cimaz^10^, E. Liebling^9^

##### ^1^Department of Immunology, hematology and rheumatology, Necker-Enfants Malades university hospital, Paris, France; ^2^Department of Medical Biotechnology, University of Siena, Siena, Italy; ^3^Department of Paediatrics, University of Zagreb School of Medicine, Zagreb, Croatia; ^4^Department of Paediatrics, University Hospital, Saint-Etienne; ^5^Department of Pediatric Hemato-Oncology, University hospital of Nancy, Nancy, France; ^6^Department of Public Health Sciences and Pediatrics, University of Torino, Torino, Italy; ^7^Alderhey Children’s NHS Foundation Trust, Liverpool, United Kingdom; ^8^Perelman School of Medicine, University of Pennsylvania; ^9^The Children's Hospital of Philadelphia, Philadelphia, United States; ^10^Department of Clinical Sciences and Community Health, University of Milano, Milano, Italy

###### **Correspondence:** R. Cimaz

**Introduction:** 22q11.2 deletion syndrome (22q11.2 DS), the most common cause of Di George syndrome, is associated with autoimmunity in approximately 10% of cases. Arthritis can be chronic but its characteristics and treatment tolerance are not well known.

**Objectives:** We describe the first international series of such patients.

**Methods:** We gathered retrospectively 21 patients from 7 centers. Demographic, laboratory, and clinical data focused on arthritis were collected and entered into a dedicated database.

**Results:**Of the 21 patients, 13 were females. Family history of Di George syndrome was present in 3 cases. Dysmorphic features were recorded in all patients, developmental delay in 19/21, cardiac defects in 17/21, palatal abnormalities in 15/21, skeletal abnormalities in 13/21, hypoparathyroidism in 8/21, ophtalmologic abnormalities in 7/21, hypoplastic thymus in 5/21. Associated autoimmunity (except arthritis) was present in 5/21 (ITP, celiac disease, psoriasis, Evans syndrome, Hashimoto: 1 case each). T and B cell numbers were within normal limits in all but one patient.

Mean age at diagnosis of arthritis was 4.9 years, with a mean number of seven involved joints at onset. Most patients (n=18) had knee involvement, followed by ankle (n=15), MCP/IP (n=11), wrist (n=10), elbow (n=5), hip (n=2). Synovial fluid analysis, when performed, was uninformative. Uveitis was never noted during the disease course. ANA was positive in 15/21 cases, while RF was never detected. An increase of inflammatory markers was detected in 15 cases for ESR and 12 for CRP. Treatment received included systemic glucocorticoids in 10 cases, intraarticular joint injections in 13, DMARDs in 14 (mostly methotrexate), and biologics 12 (Etanercept 10, Adalimumab 2, Abatacept 3, Rituximab 1). Prior to immunosuppression, infections were noted in 12 cases, 5 requiring admission. Of note, following immunosuppression infections were recorded in only two patients (one required no admission, the second was admitted for severe Evans syndrome and MAS requiring intensive immunosuppression complicated by disseminated aspergillosis and subsequently died). No patient developed neoplasia.

At last follow-up visit (mean age 13 years), arthritis was active in 9 cases, in partial remission in 2, in remission on medication in 8, and in remission off medication in only 2. Median number of active joints was 3, ESR was increased 7/12, CRP in 2/12. Eight patients had articular damage, and erosions were noted in four.

**Conclusion:** 22q11.2 DS can be associated with chronic arthritis, which is often polyarticular and progressive. Other autoimmune disorders are common in this series. DMARDS and biologics are possible treatment options, and in our series we did not observe more infections after the beginning of immunosuppression for arthritis.

**Disclosure of Interest**

None declared

### O12 Clonally expanded peripheral t helper cells with distinct tcr vβ repertoire characterize synovial inflammation in children with antibiotic-refractory Lyme arthritis

#### J. Dirks^1^, J. Klaussner^1^, A. Almamy^1^, J. Fischer^1^, G. Haase^1^, U. Fischer^1^, A. Holl-Wieden^2^, C. Hofmann^2^, H. Girschick^3^, H. Morbach^1,2^

##### ^1^Pediatric Immunology; ^2^Pediatric Rheumatology and Osteology, University Hospital Julius-Maximilians University Würzburg, Würzburg; ^3^Children's Hospital, Vivantes Klinikum im Friedrichshain, Berlin, Germany

###### **Correspondence:** J. Dirks

**Introduction:** Antibiotic-refractory Lyme arthritis (ARLA) is defined by persistent arthritis after sufficient antibiotic treatment of acute Lyme arthritis and is seen in approximately 10 % of patients with Lyme arthritis. Although some clinical and genetic risk markers for ARLA have been elucidated, the disease pathogenesis is still inadequately understood. In detail, whether chronic inflammation is sustained by persistent borrelial antigens or triggered by autoantigens is not elucidated yet.

**Objectives:** Identifying the cellular correlate of ongoing immune responses in the inflamed joints of children with ARLA to elucidate antigen targets and disease specific pathomechanisms.

**Methods:** Flow cytometric analysis of T and B cell populations in synovial fluid (SF) samples of children with ARLA and juvenile idiopathic arthritis (JIA). High-throughput sequencing of the T cell receptor β (TCR Vβ) repertoire of SF T cells and single cell immunoglobulin expression cloning of SF B cells in children with ARLA and JIA.

**Results:** Multidimensional flow-cytometric analysis revealed a striking expansion of an IL-21 and IFN-γ co-expressing PD-1^hi^CXCR5^-^HLA-DR^+^ CD4^+^ T cell population resembling peripheral T helper (T_PH_) cells in the joints of pediatric ARLA patients compared to JIA patients. Indeed, ARLA patients display the highest frequencies of T_PH_ cells, which could separate this group of patients from JIA. Accumulating T_PH_ cells exhibited signs of clonal expansion with restricted TCR clonotypes. Those clonotypes showed an overlap between different ARLA patients but not to JIA patients. Furthermore, distinct molecular patterns within the TCR Vβ repertoires diverged in ARLA and JIA patients. Paralleling the observations made in the T cell compartment, accumulating SF B cells showed oligoclonal expansion and almost exclusively displayed the phenotype of CD21^lo/-^CD11c^+^ double-negative (DN) B cells.

**Conclusion:** The inflamed joints of children with ARLA are characterized by a striking expansion of oligoclonal T_PH_ cells and DN B cells. The distinct features of the TCR Vβ repertoire of T_PH_ from ARLA patients suggest that disease specific immune response may sustain chronic inflammation in ARLA. Having defined the cellular subsets of an ongoing immune response in the joints of children with ARLA, current experiments are ongoing to dissect whether this maladaptive immune response targets persisting Borrelial antigens or rather autoantigens.

**Disclosure of Interest**

None declared

## Lightning talks: JIA (oligo, poly, psoriatic), Imaging, Psycho-social aspects and rehabilitation

### O13 Can musculoskeletal ultrasound and serum biomarkers predict disease flare in JIA patients in clinical remission?

#### M. Mazzoni^1^, S. Merlo^2^, C. Morreale^3^, A. Pistorio^4^, S. Viola^3^, F. Magnaguagno^5^, A. Corcione^6^, P. Bocca^6^, M. Gattorno^7^, A. Consolaro^3^, A. Ravelli^3^, C. Malattia^2,3^

##### ^1^Dipartimento di Neuroscienze, Riabilitazione, Oftalmologia, Genetica e Scienze Materno-Infantili, Università degli Studi di Genova, Genova; ^2^Dipartimento di Neuroscienze, Riabilitazione, Oftalmologia, Genetica e Scienze Materno-Infantili, Università degli Studi di Genova; ^3^Clinica Pediatrica e Reumatologia; ^4^Epidemiologia e Biostatistica; ^5^Radiologia; ^6^Centro Malattie Autoinfiammatorie e Immunodeficienze; ^7^Clinica Pediatrica e Reumatologia - Centro Malattie Autoinfiammatorie e Immunodeficienze, IRCCS Istituto Giannina Gaslini, Genoa, Italy

###### **Correspondence:** M. Mazzoni

**Introduction:** Clinical remission (CR) is regarded as the ideal therapeutic target in JIA because its achievement helps to prevent physical disability. Recently the question has been raised whether current measures used to define CR truly reflect the absence of synovial inflammation. In fact, musculoskeletal ultrasound (MSUS) studies have demonstrated subclinical synovitis in a sizeable proportion of JIA patients despite “clinical inactive disease”. In addition, serum biomarkers such as S100A12 and MRP8/14 may identify patients with unstable remission and increased risk of relapse.

**Objectives:** 1) to investigate the prevalence of MSUS-detected subclinical synovitis in JIA patients in CR; 2) to evaluate the persistence of subclinical synovitis over the time; 4) to investigate whether subclinical synovitis predicts disease flare and whether it should affect the therapeutic strategy; 5) to integrate MSUS data with serum biomarkers to develop a multidimensional measure of remission status.

**Methods:** 135 consecutive JIA patients who met the Wallace criteria for CR were included in this 3-years prospective study. All patients underwent MSUS assessment of 56 joints at study entry and at 6 months follow-up visit. Joints were scanned for synovial hyperplasia, joint effusion and Power Doppler (PD) signal by two independent ultrasonographers. At inclusion serum levels of the following cytokines were determined with cytofluorometry: ILR-1, G-CSF, GM-CSF, IL-6, IL-10, IL-12, CXCL9, CXCL10, MIP-1α, TNFRI, TNFRII, RANTES, VEGF. Patients were followed clinically for 3 years. A flare of synovitis was defined as a recurrence of clinically active arthritis that required a major therapeutic intervention. The association between clinical and MSUS variables with flare, was evaluated by adjusted logistic regression models.

**Results:** 135 patients (78.5% F; median age 11.3 y; median disease duration 5.7 y; median CR duration 1.4 y) were included. Seventy-eight/135 (57.7%) patients were in CR on medication. Subclinical synovitis was detected in 82/135 (60.7%) patients. Subclinical tenosynovitis was present in 20/135 (14.8%) patients. 58.6% of patients showed persistent subclinical synovitis at 6 month follow up MSUS examination. During the 3-year follow up 45/135 (33.3%) patients experienced a disease flare (median survival time 2.2 y). PD positivity in tendons was the strongest independent risk factor of flare on multivariable regression analysis (HR: 4.8; P=0.04). Other predictors of flare were the JIA subtype (oligo-extended form: HR: 2.3; P=0.031) and the status of CR on medication (HR: 3.7; P=0.002). Serum levels of G-CSF, TNFRII and CXCL10 significant differed between patients and healthy controls (P=0.010; P=0.025; P<0.0001, respectively). However serum cytokine levels were not associated with disease relapse.

**Conclusion:** our results confirm that MSUS is more sensitive than clinical evaluation in the assessment of persistent synovial inflammation in JIA patients in CR. Subclinical tenosynovitis was the best predictor of disease flare, with important therapeutic implications. To date, the role of tenosynovitis in the diagnosis and prognosis of JIA has been poorly investigated. Our results further support the role of MSUS in monitoring JIA patients in CR and to identify patients with higher risk of disease flare.

**Disclosure of Interest**

None declared

### O14 Determinants of physician global assessment in juvenile idiopathic arthritis patients without active joints

#### A. Alongi^1^, G. Giancane^2^, N. Ruperto^2^, A. Consolaro^2^, A. Ravelli^2^

##### ^1^ARNAS Civico, Palermo; ^2^Giannina Gaslini Institute, Genoa, Italy

###### **Correspondence:** A. Alongi

**Introduction:** Physician global assessment (PGA) is an essential outcome measure in Juvenile Idiopathic Arthritis (JIA), used alone or as part of composite scores and criteria for inactive disease (ID) to summarize providers’ appraisal of disease activity. Some evidence suggests a lack of standardization of PGA, demonstrated by a tendency among physicians to assign values above zero despite no apparent signs of active disease, and little attention to the impact of factors contributing to PGA scoring.

**Objectives:** To identify the determinants of the PGA in patients with JIA without active joints, and to evaluate the relative importance of their contributions to PGA.

**Methods:** 7265 complete visit records from two multinational (the EtICA study, n = 422, and the EPOCA study, n = 9081) and one national (the Gaslini dataset, n = 669) cross-sectional cohorts were examined. Rheumatologic assessment data included active (AJC), painful (PJC), limited (LJC) and swollen joint counts. Patients with AJC = 0 were selected for the analysis. PGA was measured with a 21-numbered circle VAS (0 = best; 10 = worse) and dichotomized as equal to or above zero. Presence of pain in axial (TMJ, sacroiliac and spinal joints), large (shoulders and hips), medium (elbows, wrists, knees, foot-ankles) and small (distal interphalangeal, proximal interphalangeal, metacarpophalangeal and metatarsophalangeal) joints was coded as a dummy variable for each pattern. Multivariate logistic regression models were fitted to explain the probability of a PGA > 0 in patients without active joints, based on *erythrocyte sedimentation rate* (ESR) values, systemic features, active uveitis, morning stiffness, axial, large, medium, and small painful joints, VAS-measured overall pain, total PJC, LJC and ILAR category. We used dominance analysis to compare the relative importance of predictors.

**Results:** Among 7265 patients, 4108 (56.5%) had negative AJC; within this subgroup, PGA was marked above zero in 32.4% (median 0, IQR 0.0 - 0.5). In 14.2% PGA was the only not-met criteria for ID among the ACR 2011 set, making it the single most frequent reason for not reaching ID. ESR, systemic features, uveitis, stiffness, large joint pain, axial joint pain, overall pain, PJC, LJC and ILAR subtype were independent predictors of a PGA > 0. Large joint pain showed the highest impact on PGA (OR=9.18; CI 2.35 - 61.05, p 0.005), followed by axial joint pain (OR=6.39; CI 2.11 - 23.84, p 0.002), systemic features (OR=3.70; CI 1.75 - 8.26, p<0.001), uveitis (OR=3.77; CI 2.49 - 5.70, p<0.001), PJC (OR=2.78; CI 1.68 - 5.04, p<0.001). ERA patients showed higher odds of PGA > 0 (OR=1.42; CI 1.03 - 1.95, p 0.032), while oligoarthritis was associated with lower odds (OR=0.75; CI 0.58 - 0.97, p 0.028). The model’s explanatory power was substantial (R_M_^2^ = 0.27). As shown in the table, dominance analysis revealed pain in the large joints as the most important variable to explain PGA variability (average R_M_^2^ 0.073), followed by PJC (0.051) and axial joints pain (0.050).

**Conclusion:** A substantial proportion of patients received a PGA above zero despite the absence of active joints. Large and axial joint pain, uveitis, systemic features and PJC were the main determinants of PGA. Painful joint patterns and PJC accounted for most of the variability in PGA scoring. Further research is needed to investigate factors driving PGA and their impact on classification and response assessment in JIA.

**Disclosure of Interest**

None declared

**Table 1 (abstract O14).** See text for description

*Predictors*	*Average contribution (R*_*M*_^*2*^*)*
Large joints pain	0.073
PJC	0.051
Axial joints pain	0.050
Pain	0.042
Stiffness	0.017
LJC	0.016
ESR	0.007
Uveitis	0.005
Systemic features	0.004

### O15 Assessing the causal role of the human gut microbiome on JIA risk: a mendelian randomisation study

#### S. L. Clarke^1,2,3^, D. A. Hughes^1,3^, G. C. Sharp^1,3^, A. V. Ramanan^2,4^, C. L. Relton^1,3^, K. H. Wade^1,3^

##### ^1^MRC Integrative Epidemiology Unit, University of Bristol; ^2^Department of Paediatric Rheumatology, Bristol Royal Hospital For Children; ^3^Population Health Sciences; ^4^Translational Health Sciences, University of Bristol, Bristol, United Kingdom

###### **Correspondence:** S. L. Clarke

**Introduction:** There is growing interest in the role of the microbiome in human health and increasing evidence of associations between components of the human gut microbiome and juvenile idiopathic arthritis (JIA). However, few findings have been replicated across studies, and robust evidence of a causal association is lacking. Most microbiomic studies are of cross-sectional or case-control design and are thus subject to confounding, reverse causation, and other biases. An alternative approach to examine the association between the human gut microbiome and JIA is using Mendelian Randomisation (MR), a method of causal inference which has recently been applied in the context of microbiome research. The reliance of MR on human genetic variation, assigned at the point of conception, makes it less susceptible to reverse causation and confounding, provided key assumptions are met.

**Objectives:** To use MR to examine the evidence for a causal association between the human gut microbiome, as measured by faecal samples, and JIA risk.

**Methods:** Genetic variants strongly associated with human faecal microbial taxa have recently been reported in a genome wide association study (GWAS) meta-analysis of three European cohorts (sample size 3,890), using a presence/absence and/or an abundance model. We combined this data with summary data from the most recent JIA GWAS (sample size 12,501) to examine the causal effect of 13 microbial taxa on JIA risk. We also examined this association in reverse (i.e. whether JIA has a causal effect on microbial taxa) using genetic variants associated with JIA from an Immunochip study (sample size 15,872) and summary data from the Flemish Gut Flora Project (sample size 2,223). We undertook these analyses using the MR-Base platform. Additional sensitivity analyses were performed to assess the robustness of the MR estimates and identify potential violations of the core MR assumptions.

**Results:** Of the 13 microbial taxa examined, we found strong evidence for a causal effect of a higher abundance of bacteria within the *Firmicutes* phylum on JIA risk (OR 1.75, 95% CI 1.12-2.72 per standard deviation (SD) higher abundance). This finding is supported by weaker evidence of a causal effect of two further taxa on JIA risk; the presence of bacteria within the *Firmicutes* phylum (OR 1.15, 95% CI 0.99-1.34 per doubling in genetic liability to bacteria within the *Firmicutes* phylum) and a higher abundance of bacteria within the *Butyricicoccus* genus (OR 1.50, 95% CI 0.94-2.38 per SD higher abundance). We found no evidence of a causal association in reverse; increased genetic liability to JIA was not causally associated with alterations in these microbial taxa. There was no strong evidence that there were violations of the core MR assumptions.

**Conclusion:** Whilst our findings are inconsistent with much of the observational human literature (which suggests an inverse association between JIA and *Firmicutes* bacteria), these cross-sectional and case-control studies may reflect a post-disease or treatment-related association. Accordingly, current murine data suggests that arthritis is preceded by an increase in *Firmicutes* bacteria during the pre-clinical disease phase, as is found in our study. Further work to explore these putative causal relationships and to understand the dynamics of the microbiomic composition in disease is warranted.

**Patient Consent Received**

No

**Disclosure of Interest**

None declared

### O16 Twenty years experience with etanercept in treatment of juvenile idiopathic arthritis

#### A. Klein^1,2^, D. Windschall^3^, A. Hospach^4^, K. Minden^5^, F. Weller-Heinemann^6^, F. Dressler^7^, G. Horneff^8^, on behalf of BIKER

##### ^1^Pediatric rheumatology, Asklepios Klinik Sankt Augustin, Sankt Augustin; ^2^Universitiy Cologne, Cologne; ^3^Pediatric rheumatology, St. Josef Stift, Sendenhorst; ^4^Olga Hospital, Stuttgart; ^5^Charité Universitätsmedizin, Berlin; ^6^Prof Hess Kinderklinik, Bremen; ^7^Medizinische Hochschule Hannover, Hannover; ^8^Asklepios Klinik Sankt Augustin, Sankt Augustin, Germany

###### **Correspondence:** A. Klein

**Introduction:** Etanercept (ETA) is the most commonly prescribed biologic for treatment off juvenile idiopathic arthritis (JIA). The German biologics in JIA register - BIKER monitors long-term safety and effectiveness of ETA in the treatment of JIA in routine clinical practice.

**Objectives:** To assess long-term safety and tolerability of ETA treatment in a large cohort of JIA patients in comparison to a biologic-naïve cohort treated with methotrexate (MTX). To assess effectiveness of ETA treatment and reasons for discontinuation.

**Methods:** Patient assessment was performed at baseline, after 3 and 6 months, and every 6 months thereafter. Baseline demographics and disease activity parameters have been documented. Efficacy was determined using the JADAS10. Safety assessments were based on adverse events reports (AE) processed according to MedDRA

**Results:** Altogether, 2885 JIA patients covering 6560.3 patient years (PY) of exposure to ETA for up to eight years of continuous treatment, and 1517 biologic-naïve patients accumulating 3893.6 PY of exposure to MTX were enrolled.

A higher percentage of patients in the ETA cohort had a polyarticular course (extended oligoarthritis [20.9%], RF-negative- [33.2%] and positive [8.1%] polyarthritis) than the MTX cohort (13.5%, 27.3%, and 3.4%). Mean age at treatment start and disease duration was higher in the ETA cohort (12.1 +/- 4.4 years; 4.1 +/- 3.7 years) compared to the MTX cohort (9.8 +/-4.8 years; 2.1 +/- 2.8 years). In all, 2531 AEs were reported during ETA exposure or up to 90 days of follow-up (38.5/100 PY [95% CI 37.1-40.1]). In the MTX cohort, 1354 AEs were reported (34 /100 PY [95% CI 32.9-36.6]). More SAEs (RR=2.88, 95% CI 2.12-3.9) and serious infections (RR=4.8, 95% CI 2.2-10.6) were observed in the ETA cohort. Also, more patients experienced herpes zoster reactivation (RR=3.7, 95% CI 1.3-10.6; 0.8% versus 0.3%, p=0.027) and inflammatory bowel disease (RR=13.6, 95% CI 1.8-10.1; 0.8% versus 0.07%; p=0.0008). There was no statistical difference in the rates of malignancies in patients ever exposed to ETA or MTX. During treatment, a marked clinical response was documented with JIA-ACR 30/50/70/90 scores in 68%/61%/48%/34%. JADAS minimal disease activity/JADAS remission was achieved in 60%/38% at last follow-up on ETA. Reasons for discontinuation of ETA were remission in 40% of patients, inadequate efficacy in 35% and intolerance in 12% of all discontinuations

**Conclusion:** This registry cohort represents the largest cohort of ETA-treated JIA patients studied. A rapid improvement upon ETA treatment was observed and could be maintained up to eight years of continuous drug use. More AEs, SAE and serious infections were observed in the ETA cohort. In all, paediatric patients demonstrated a safety profile consistent with observations in adults. he benefit-risk profile of ETA remains unchanged for the approved paediatric Tindication JIA.

**Patient Consent Received**

No

**Disclosure of Interest**

None declared

**Table 1 (abstract O16).** Patient characteristics, adverse events and JADAS at baseline and last follow-up in ETAt and MTX cohort

	ETA cohort N=2885	MTX cohort N=1517
Female, n (%)	1937 (67.1)	1023 (67.4)
Disease Duration, years, mean (SD)	4.1 (3.7)	2.1 (2.8)
ANA positive, n (%)	1388 (48.1)	727 (47.9)
HLA B 27 positive, n (%)	685 (23.7)	265 (17.5)
AE, n; rate/100PY (95%CI)	2531; 38.5 (37.1-40.1)	1354; 34 (32.9-36.6)
SAE, n; rate/100PY (95%CI)	243; 3.7 (3.2-4.2)	50; 1.3 (0.9-1.6)
Serious infections, n; rate/100PY (95%CI)	57; 2.0 (1.5-2.6)	7; 0.5 (0.2-1.0)
Baseline JADAS 10, mean (SD)	15.2 (7.5)	13.8 (7.1)
Last observation JADAS 10, mean (SD)	6.3 (7.2)	5.3 (6.8)

### O17 Innovative methods for biomarker discovery in oligoarticular juvenile idiopathic arthritis

#### F. Raggi^1^, C. Rossi^1^, S. Pelassa^1^, D. Cangelosi^2^, M. Bartolucci^3^, A. Petretto^3^, F. Antonini^3^, P. Bocca^4^, F. Penco^4^, M. Rossano^5^, F. Baldo^5^, G. Filocamo^5^, C. Trincianti^6^, A. Eva^1^, A. Ravelli^4^, A. Consolaro^4^, M. C. Bosco^1^

##### ^1^Laboratory of Molecular Biology; ^2^Clinical Bioinformatic Unit; ^3^Core Facilities; ^4^Pediatric Rheumatology Clinic, IRCCS Istituto Giannina Gaslini, Genova; ^5^Fondazione IRCCS Ca' Granda, Ospedale Maggiore Policlinico, Milano; ^6^University of Genova, Genova, Italy

###### **Correspondence:** F. Raggi

**Introduction:** New biomarkers for early prediction of disease progression are demanded for the management of Oligoarticular Juvenile Idiopathic Arthritis (OJIA), the most common chronic pediatric rheumatic arthritis in Western countries. Since cells causing inflammation and tissue-destructive effects release extracellular vesicles (EV) both in plasma and synovial fluid of the inflamed joints, the characterization of EV content at disease onset may be valuable for the identification of early predictive biomarkers.

**Objectives:** This study was aimed at identifying new candidate biomarkers able to predict disease progression and response to treatment. We developed an integrated strategy that combines classical approaches for the study of inflammatory cells in liquid biopsies and system biology–driven omics methods (miRNomic, proteomic) for the analysis of EV released by these cells

**Methods:** 30 OJIA patients were enrolled in the study at disease onset and followed up for 12 months after diagnosis and initiation of therapy. EV miRNA (EV-miR) and EV-protein (EV-Prot) expression profiling were carried out in PL and SF samples using TaqMan Array RT-PCR and mass spectrometry. PL from 25 age-matched healthy children was used as control. Macrophages and T cells from 10 patients of the same cohort were isolated from SF aspirates and characterized by cytofluorimetry using Kaluza software

**Results:** Principal Component Analysis showed a separation among different biological groups on the basis of Exo-miR expression profiles. Differential expression analysis identified 16 and 34 EV-miRs significantly up- and down-regulated, respectively, in SF vs both paired and control PL. Pathway analysis of these EV-miRs identified significantly enriched processes related to inflammatory responses, cartilage/bone homeostasis, and hypoxia, including TNF, NF-kappa B, mTOR, JAK-STAT, cytokine, chemokine, TGFb, HIF-1, and VEGF signaling pathways. Macrophage and T cell derivation of these EV-miR was suggested by in vitro experiments with mononuclear cells cultured for 48h. Five candidate miRNAs with differential expression were validated by qRT-PCR and selected as disease-specific, suggesting their implication in inflammatory condition at both local and systemic level. Unsupervised K-means Clustering analysis identified a few of these EV-miRs as able to discriminate subgroups of patients within the OJIA cohort, suggesting their potential predictive value. EV-Prot analysis demonstrated mean expression of about 1000 protein in both SF and PL samples, with a specific representativeness of the tissue of origin. Proteins with potential to modulate inflammatory and immunological processes were identified. The potential correlation between EV-miR and EV-Prot expression levels and patient clinical data is under study. The analysis of SF cells revealed different ratio of M1/M2 macrophages expressing the immunostimolatory hypoxic receptor TREM1 and activated CD4/CD8 T cells among outcome groups

**Conclusion:** We provide the first database containing EV-miR, EV-Prot, and cell phenotypic data of new-onset OJIA patients. The predictive value of these results could be instrumental for a better understanding of disease molecular pathogenetic mechanisms and the definition of novel early candidate diagnostic biomarkers with potential for the development of personalized therapeutic strategy

**Patient Consent Received**

Yes

**Disclosure of Interest**

None declared

### O18

**Withdrawn**

## Lightning talks: Systemic lupus erythematosus and antiphospholipid syndrome

### O19 Disease-causing gene variants account for a minimum OF 5.5% OF juvenile-onset sle patients in the UK

#### A. Charras^1^, S. Haldenby^2^, E. M. D. Smith^3^, C. Roberts^1^, M. W. Beresford^3^, C. M. Hedrich^1^, on behalf of the UK JSLE Cohort Study

##### ^1^Department of Women’s & Children’s Health, Institute of Life Course and Medical Sciences, University of Liverpool; ^2^Centre for Genomic Research, Institute of Infection, Veterinary & Ecological Sciences, University of Liverpool; ^3^Department of Paediatric Rheumatology, Alder Hey Children's NHS Foundation Trust Hospital, Liverpool, United Kingdom

###### **Correspondence:** A. Charras

**Introduction:** Systemic Lupus Erythematosus (SLE) is a complex autoimmune/inflammatory disease. Juvenile-onset (j)SLE affects 15-20% of lupus patients and is characterized by increased organ involvement and damage, and higher need for immune suppressive treatment. Clinical heterogeneity between ethnicities, age groups and individual patients suggest variable pathophysiology.

**Objectives:** This study aimed at the definition of patient sub-cohorts with “genetic” vs. “classical” SLE to allow individualized care.

**Methods:** Applying target enrichment and new generation sequencing, jSLE patients (N=348) from the UK JSLE Cohort Study were screened for disease-causing mutations. Findings were integrated with demographic information and clinical datasets, including SLEDAI, pBILAG organ domain and SLICC damage scores.

**Results:** Approximately 5.5% of jSLE patients carried disease-causing mutations, primarily affecting nucleic acid sensing and metabolism (68%), immune complex clearance (11%), their combination (11%), immune cell signalling (5%) and NFκB signalling (5%). Patients with “genetic SLE” were younger, and exhibited less organ involvement and damage at diagnosis (neuropsychiatric, haematological, gastrointestinal), while neuropsychiatric involvement developed over time. When compared to the remaining cohort, “genetic SLE” associated with anti-dsDNA antibody positivity at diagnosis, and reduced ANA, anti-LA and anti-Sm antibody positivity at last visit which may explain reduced renal and haematological involvement.

**Conclusion:** Genetic disease accounts for ≥5.5% of jSLE cases. It associates with peri-pubertal onset, and distinct immunological and clinical pictures. As less commonly present after treatment induction, in “genetic SLE”, autoantibodies may be the result of tissue damage. Routine sequencing will allow for patient stratification, risk assessment, and target-directed treatment with reduced toxicity and increased efficacy.

**Patient Consent Received**

Yes

**Disclosure of Interest**

None declared

### O20 Induction therapy for pediatric onset lupus nephritis : mycophenolate mofetil versus cyclophosphamide

#### M. Chbihi^1^, L.-A. Eveillard^2^, Q. Riller^3^, N. Garcelon^4^, O. Boyer^2^, B. Bader-Meunier^1^

##### ^1^Immunology, Hematology and Rheumatology; ^2^Nephrology, Necker Hospital for Sick Children; ^3^INSERM UMR1163, immunogenetics of Pediatric Autoimmune Diseases; ^4^Data Science Platform, Imagine Institute, Paris, France

###### **Correspondence:** M. Chbihi

**Introduction:** Class IV lupus nephritis (LN) is one of the most severe involvements in systemic lupus erythematosus and is particularly frequent in case of pediatric onset. The gold standard induction treatment consists of intravenous (IV) pulses of Cyclophosphamide (CYC) in association with corticosteroids. It has considerably improved the renal prognosis but has potential short and long-term toxic effects. Recent studies in adults have shown similar efficacy of oral Mycophenolate Mofetil (MMF) as induction therapy with a lower toxicity. However, the pediatric literature is scarce and current treatment guidelines are extrapolated from the adult population.

**Objectives:** The aim of the study was to compare the efficacy and tolerance of CYC and MMF as induction treatment of a first episode of class IV LN in children.

**Methods:** We conducted a monocentric retrospective study including all consecutive children (<18 years) with at least 4 American college of rhumatology criteria for lupus, and biopsy-proven class IV LN according to the IRS/RPS classification, and who had not received any prior immunosuppressive treatment.

**Results:** Among the 33 patients, 17 had been treated with oral MMF (51%) and 16 with IV CYC. The basic characteristics were similar in both groups except for more neurological involvement in the CYC group (6/17 vs. 0/16). There was a non-significant trend for more severity in the CYC group with higher grade proteinuria, lower albuminuria, and more frequent acute kidney injury. At one year, 53% of the patients from the MMF group and 77% from the CYC group had achieved remission (p=0.25). 59% of the patients from the MMF group had relapsed, versus 50% of patients from the CYC group (p=0.87), respectively at 3.4 years and 4.7 years after beginning of treatment (p=0.41). The severe and mild complication rates were not significantly different between the two groups.

**Conclusion:** In conclusion, we found no difference in the kidney outcome and side effects in children receiving either MMF or CYC as induction therapy of class IV LN. However, in this retrospective study, no patient from the MMF group had neurological involvement, and there was a trend for more severity in the CYC group. Further studies are needed to confirm these results with stratification of children by disease severity.

**Disclosure of Interest**

None declared

### O21 Treatment of juvenile-onset systemic lupus erythematosus – “real world” data from the UK JSLE cohort study

#### N. Egbivwie^1,2^, A. L. Jorgensen^3^, M. W. Beresford^1,2^, C. M. Hedrich^1,2^, E. M. D. Smith^1,2^, on behalf of UK JSLE Study group

##### ^1^Department of Women’s and Children’s Health, Institute of Life Course and Medical Sciences, University of Liverpool; ^2^Department of Paediatric Rheumatology, Alder Hey Children’s NHS Foundation Trust, ^3^Department of Biostatistics, University of Liverpool, Liverpool, United Kingdom

###### **Correspondence:** N. Egbivwie

**Introduction:** In the absence of paediatric clinical trials, treatment and care plans vary significantly in Juvenile-onset systemic lupus erythematosus (JSLE). Aiming at harmonized treatment and collection of response data, collaborative efforts from leading experts delivered consensus treatment plans (Childhood Arthritis and Rheumatology Research Alliance) and recommendations (Single Hub and Access point for paediatric Rheumatology in Europe)[1, 2].

**Objectives:** The study explored how clinical manifestations impact on choice and sequence of immunosuppressants used for the treatment of JSLE.

**Methods:** ‘Real world’ treatment data from the UK JSLE Cohort Study (01/2010-05/2020) was accessed. The choice and sequence of immunomodulating drugs used in clinical management of JSLE (1^st^, 2^nd^ and 3^rd^-line) was explored from diagnosis to last visit. Paediatric British Isles Lupus Assessment Grade (pBILAG) organ domain disease activity scores were used to explore how different clinical manifestations guide treatment choice. Logistic regression was used to determine how treatment choice associated with organ domains.

**Results:** 349 patients were included with 290 females (83%). Data from 3266 visits were assessed (median: 9 visits/patient IQR=9), median age at diagnosis was 13 (IQR=4) and median follow up was 4 years (IQR=4). Immunomodulating treatments in addition to hydroxychloroquine (HCQ) and/or corticosteroids were considered. To capture the sequence of immunosuppressants used from diagnosis, this analysis focused on 197/349 patients diagnosed within the study dates. Overall, 10/197 (5%) were treated solely with HCQ, 73/197 (37%) received a single immunomodulator, 75/197 (38%) received two and 40/197 patients (20%) received three or more during follow-up. The most common 1^st^-line immunomodulating treatment was mycophenolate mofetil (MMF) (72/197, 37%) followed by azathioprine (56/197, 28%) and methotrexate (43/197, 22%). MMF was the most common 2^nd^-line treatment (40/197, 20%) followed by rituximab (RTX) (23/197, 12%). RTX was the most commonly chosen 3^rd^-line treatment (15/197, 8%).

Across most organ domains, MMF was the most common treatment (except for gastrointestinal and ophthalmic). Patients with renal disease were most likely to receive MMF (OR 1.99 95% CI: 1.65-2.41; p=0.004). Treatment with RTX was significantly more likely in patients with neuropsychiatric (OR 1.84 95% CI: 1.05-3.21), renal (OR 1.50 95% CI: 1.12-2.00) and cardiorespiratory disease (OR 2.57 95% CI: 1.40-4.74) compared to patients with other organ involvement (p<0.05). Patients with neuropsychiatric (OR 3.10 95% CI: 1.80-5.33), renal (OR 1.61 95% CI: 1.16-2.23), cardiorespiratory (OR 5.05 95% CI: 2.82-9.04), haematological (OR 2.82 95% CI: 1.92-4.16) and mucocutaneous (OR 1.95 95% CI: 1.39-2.74) involvement were more likely to receive cyclophosphamide (CPM) compared to patients with other organ involvements (p<0.01).

**Conclusion:** Most patients were treated with two immunomodulators in addition to HCQ and/or corticosteroids. Notably, MMF was the most common immunomodulator, with RTX and CPM used in more resistant disease, particular for renal, neuropsychiatric and cardiorespiratory involvement. Observations will inform subsequent treat to target study algorithms as part of the TARGET LUPUS research programme.

1 Mina R, von Scheven E, Ardoin SP et al. Consensus treatment plans for induction therapy of newly diagnosed proliferative LN in JSLE. Arthritis Care Res 2012;64(3):375-83

2 Groot N, de Graeff N, Marks SD et al. European evidence-based recommendations for the diagnosis and treatment of childhood-onset LN:the SHARE initiative. *Ann Rheum Dis* 2017;76(12):1965-73

**Disclosure of Interest**

None declared

### O22 Excessive production of interferon-γ drives the expansion of T-bet+ B cells in patients with systemic lupus erythematosus

#### E. Marasco^1^, G. M. Moneta^1^, C. Bracaglia^1^, I. Caiello^1^, C. Farroni^2^, R. Carsetti^2^, F. De Benedetti^1^

##### ^1^Division of Rheumatology; ^2^B Cell Physiopathology Unit, Ospedale Pediatrico Bambin Gesù, Roma, Italy

###### **Correspondence:** E. Marasco

**Introduction:** Paediatric systemic lupus erythematosus (pSLE) is an autoimmune disorder of childhood characterized by the production of autoantibodies against nuclear antigens. In the last decade, several studies showed an up-regulation of genes induced by type I interferons (IFNα) in peripheral blood and tissues of pSLE patients^2^. More recently, also the type II interferon (IFNγ) has been implicated in pSLE; however, its precise role has not been clarified yet^3^.

**Objectives:** To investigate the role of IFNγ in the pathogenesis of pSLE evaluating: 1) the expression levels of IFNγ-related genes in the peripheral blood of pSLE patients followed longitudinally; 2) the expression of T-bet in B cells of pSLE patients; the induction of T-bet in B cells by IFNγ.

**Methods:** Expression levels of IFNα-induced genes (IFI27, IFI44L, IFIT1, RSAD2, ISG15, SIGLEC1), IFNγ and IFNγ-induced genes (CXCL9, CXCL10, IDO1) were analysed by qPCR in whole blood of pSLE patients and healthy donors(HD). We developed a type II IFN score similarly to the type I IFN score described by Crow^4^. Expression of T-bet in B cells was evaluated by flow cytometry. Peripheral blood mononuclear cells (PBMCs) from 5 HD were stimulated *in vitro* with recombinant human IFNγ and IFNα. Serum levels of CXCL9 were evaluated by ELISA. For each patient, SLEDAI was calculated.

**Results:** Expression levels of both IFNα and IFNγ-induced genes was upregulated in patients with pSLE (n=39). The type II IFN score weakly correlated with the SLEDAI (*r*=0.33, *P*=0.03). As previously reported, the type I IFN score significantly correlated with SLEDAI (*r*=0.50, *P*<0.01). We found increased serum levels of CXCL9 in pSLE patients compared to HD (mean±SD: HD 333±117pg/mL, SLE 2125±4885pg/mL, *P*=0.0003). Eight patients were enrolled at disease onset before any treatment was administered: type I score decreased with initiation of immunosuppressive treatment; on the other hand, type II score and levels of CXCL9 were not significantly affected by treatment. Interestingly, type II score (mean±SD: No LN 3.3±3, LN 6.7±11, *P*=0.045) and CXCL9 (mean±SD: No LN 816±1225pg/mL, LN 2427±5436pg/mL, *P*=0.031) were significantly higher in patients with lupus nephritis(LN). Thus, patients with pSLE have increased activity of IFNγ, and this particularly evident in patients with LN.

B cells play a crucial role in the pathogenesis of SLE. In murine models of SLE, IFNγ was shown to activate B cells to make autoantibodies^4^. We evaluated the expression of T-bet (a transcription factor induced specifically by IFNγ) in B cells: we observed a population of B cells expressing T-bet in the naïve compartment in patients with pSLE. The frequency of T-bet^+^ naïve B cells correlated with SLEDAI. To confirm the induction of T-bet in B cells by IFNγ, we stimulated PBMCs of HD with either IFNγ or IFNα: both chemokines induced the expression of T-bet in naïve B cells. Since it is known that IFNα can induce the expression of IFNγ, we stimulated cells with IFNα and an antibody blocking IFNγ: in this setting IFNα did not upregulate the expression of T-bet in B cells.

**Conclusion:** Our data suggest a potential role of IFNγ in the pathogenesis of pSLE. IFNγ-induced genes in whole blood and CXCL9 in serum were increased in patients with pSLE, especially in patients with LN. We observed an expansion of T-bet+ naïve B cells in patients with pSLE. IFNγ specifically induced the expression of T-bet in naïve B cells. Thus, IFNγ is hyperactiaved in SLE, inducing the aberrant expression of T-bet in naïve B cells. Further research is needed to dissect the role of IFNγ-activated B cells in pSLE.

**References**

Petri M, et al. Lupus. 2009

Munroe M, et al. *Ann Rheum Dis* 2016

Rice GI, et al. *Lancet Neurol* 2013

Jackson SW, J Ex Med. 2016

**Patient Consent Received**

Yes

**Disclosure of Interest**

None declared

### O23 Low disease activity state and clinical remission are achievable targets in JSLE, leading to a significant reduction in severe flare and new damage

#### E. M. Smith^1,2^, K. Tharmaratnam^3^, C. M. Hedrich^1^, A. L. Jorgensen^3^, M. W. Beresford^1^, on behalf of on behalf of the UK JSLE Cohort Study

##### ^1^Institute of Life Course and Medical Sciences, University of Liverpool; ^2^Department of Paediatric Rheumatology, Alder Hey Children's NHS Foundation Trust; ^3^Department of Health Data Science, Institute of Population Health, University of Liverpool, Liverpool, United Kingdom

###### **Correspondence:** E. M. Smith

**Introduction:** A treat-to-target approach (T2T), where treatment is escalated until a specific target is achieved, with re-escalation if attainment of the target is lost, has been proposed as a strategy to improve adult-SLE outcomes. An international task force of experts has developed adult-onset SLE T2T guidance, to underpin development of the T2T approach. These recommendations highlight that lack of validated remission and low disease activity target state definitions (known as LDA, LDAS or LLDAS) is a fundamental knowledge gap limiting progress towards a T2T approach [1]. The **TARGET LUPUS** research programme: ‘**T**argeting disease, **A**greeing **R**ecommendations and reducing **G**lucocorticoids through **E**ffective **T**reatment, in **LUPUS**’ has been established in order to develop a Juvenile-onset Systemic Lupus Erythematosus (JSLE) T2T study.

**Objectives:** To assess the achievability and impact of attaining LDAS or remission in JSLE.

**Methods:** Achievement of three adult-SLE derived definitions of LDAS (APLC-LLDAS [2], adapted-APLC LLDAS [3, 4], Toronto-LDA [5]), and four definitions of remission (in accordance with the recommendations of the DORIS international task force remission guidance, including cSLEDAI-defined remission on/off treatment, pBILAG-defined remission on/off treatment)[6], were assessed in UK JSLE Cohort Study patients [7]. The required duration for remission to be achieved was not pre-specified. Prentice-Williams-Petersen-GAP recurrent event models assessed the impact of LDAS/remission achievement on severe disease flare (pBILAG of A or B in any organ domain) and new damage (increase in total SLICC-SDI damage score by at least one unit).

**Results:** 430 UK JSLE Cohort Study patients were included (359 female, 83%), diagnosed with JSLE at a median of 12.8 years [IQR 10.4, 14.6]. Data from 4,738 visits were analysed, median of 10 visits [5, 15] per patient, over a median of 2.0 years [0.7, 4.0]. APLC-LDAS was achieved by 286/430 (67%) of patients, adapt-APLC definition in 314/430 (73%), and Toronto-LDA in 136/430 (32%). Remission on treatment was more common (61% cSLEDAI-defined, 42% pBILAG-defined) than remission off treatment (31% cSLEDAI-defined, 21% pBILAG-defined). Achievement of each LDAS/remission target state, and disease duration (>1 year) significantly reduced the hazard of severe flare (p<0.001). Increasing SLICC-SDI damage score during follow-up increased the hazard of severe flare (p<0.001). For all targets, as cumulative time in target increased, hazard of severe flare reduced. APLC-LLDAS target achievement reduced hazard of severe flare more than adapt-APLC LDAS (p<0.001). Achievement of APLC-LDAS target had a comparable impact on severe flare, to the attainment of remission definitions (p>0.05). Achievement of all targets LDAS/remission reduced the hazards of new damage (p<0.05).

**Conclusion:** This is the first study to demonstrate that adult-derived definitions of LDAS/remission are achievable in JSLE, significantly reducing risk of severe flare and new damage. Balancing achievability and impact, the APLC-LLDAS definition performed best, demonstrating comparable effect on severe flare to the achievement of clinical remission. Paediatric-specific adaptations of these targets should be considered.

**Patient Consent Received**

Yes

**Disclosure of Interest**

None declared

### O24 BCAP as a modulator of LPS-induced interferon production in relevance to the pathogenesis of systemic lupus erythematosus

#### A. Tesser^1^, G. M. Piperno^2^, A. Pin^1^, E. Piscianz^1^, E. Valencic^1^, V. Boz^3^, F. Benvenuti^2^, A. Tommasini^1,3^

##### ^1^Department of Pediatrics, Institute for Maternal and Child Health - IRCCS “Burlo Garofolo”; ^2^International Centre for Genetic Engineering and Biotechnology; ^3^Department of Medicine, Surgery, and Health Sciences, University of Trieste, Trieste, Italy

###### **Correspondence:** A. Tesser

**Introduction:** PI3K kinases cover crucial roles in maturation, proliferation and survival of immune cells. The signaling of PI3Ks is fine-tuned by BCAP protein (encoded by *PIK3AP1* gene), which connects TLRs activation and determines the signaling of NF-kB or interferon (IFN) pathway. Moreover, the role of BCAP in promoting IFN-producing macrophages and autoimmune B cells suggests its possible involvement in the pathogenesis of SLE, along with TLR stimulation by bacterial LPS (1-5).

Previous studies on a monogenic form of SLE (DNase2 interferonopathy, D2I) demonstrated that the “priming” of type I IFN pathway make cells hyper-responsive to LPS-induced production of IFNs (6) and that BCAP could play a key role in the crosstalk LPS-IFN.

**Objectives:** By studying D2I, we aim to unravel the role of BCAP and PI3K in regulating the synergy LPS-IFN in SLE models.

**Methods:** D2I fibroblasts were pre-treated for 2h with STING inhibitor H-151 (10μM) and PI3Kδ inhibitor Leniolisib (10μM, 50μM), both in single and combined treatment, and after challenged for 1h with LPS (0.5μg/mL). Phosphorylated-TBK1 was measured by intracellular staining with specific antibodies in flow-cytometry.

D2I fibroblasts were also stimulated for a longer time (48h) with H-151 (10μM) and Leniolisib (10μM, 50μM), both in single and combined treatment, for RNA extraction and retro-transcription. *PIK3AP1* relative quantification was conducted by Real-Time PCR with specific probes and two housekeeping genes in relation to un-stimulated control fibroblasts.

**Results:** The IFN pathway activation in D2I fibroblasts after stimulation with LPS could be reduced by the combined inhibition of STING and PI3Kδ.

Moreover, in the “naturally-IFN-primed” D2I fibroblasts, *PIK3AP1* is hyper-expressed compared to control, and is down-regulated by either single or combined inhibition of STING and PI3Kδ.

**Conclusion:** Further studies to investigate the potential of STING and PI3Kδ inhibition for the reduction of LPS-induced IFN-production and for *PIK3AP1* regulation in SLE models will improve understand the clinical relevance of LPS-IFN pathways crosstalk in SLE pathogenesis, paving the way for novel therapeutic approaches.

**References**

1. Ruse M, Knaus UG. New players in TLR-mediated innate immunity: PI3K and small Rho GTPases. Immunol Res. 2006;34(1):33-48.

2. Troutman TD, Hu W, Fulenchek S, Yamazaki T, Kurosaki T, Bazan JF, et al. Role for B-cell adapter for PI3K (BCAP) as a signaling adapter linking Toll-like receptors (TLRs) to serine/threonine kinases PI3K/Akt. Proc Natl Acad Sci U S A. 2012;109(1):273-8.

3. Chu T, Ni M, Chen C, Akilesh S, Hamerman JA. Cutting Edge: BCAP Promotes Lupus-like Disease and TLR-Mediated Type I IFN Induction in Plasmacytoid Dendritic Cells. J Immunol. 2019;202(9):2529-34.

4. Ma Y, Xu X, Li M, Cai J, Wei Q, Niu H. Gut microbiota promote the inflammatory response in the pathogenesis of systemic lupus erythematosus. Mol Med. 2019;25(1):35.

5. Azzouz D, Omarbekova A, Heguy A, Schwudke D, Gisch N, Rovin BH, et al. Lupus nephritis is linked to disease-activity associated expansions and immunity to a gut commensal. Ann Rheum Dis. 2019;78(7):947-56.

6. Tesser A, Piperno GM, Pin A, Piscianz E, Boz V, Benvenuti F, et al. Priming of the cGAS-STING-TBK1 Pathway Enhances LPS-Induced Release of Type I Interferons. Cells. 2021;10(4).

**Patient Consent Received**

No

**Disclosure of Interest**

None declared

## Lightning talks: Macrophage activation syndrome and Systemic JIA

### O25 Mortality and clinical response to treatment in children with secondary hemophagocytic lymphohistiocytosis (SHLH)

#### C. Bracaglia, R. Pecoraro, D. Pires Marafon, A. De Matteis, G. Marucci, M. Pardeo, F. De Benedetti

##### Division of Rheumatology, IRCCS Ospedale Pediatrico Bambino Gesù, Roma, Italy

###### **Correspondence:** C. Bracaglia

**Introduction:** sHLH is a life-threatening condition associated with several disorders, such as infections, malignancies and rheumatologic/inflammatory diseases. In a significant number of cases an apparent underlying disease cannot be found. Data on mortality rate and on clinical response to treatments (CRT) are lacking or limited to small series.

**Objectives:** To evaluate mortality rate and CRT in a cohort of sHLH patients.

**Methods:** A retrospective chart review of sHLH patients followed at Ospedale Pediatrico Bambino Gesù from April 2006 through September 2020 was performed. Patients with sHLH in the context of sJIA and secondary to malignancy were excluded. Clinical, laboratory features and treatment data were collected at onset, at 1, 3 and 6 months in order to assess CRT. The last follow-up was used to evaluate mortality. To evaluate CRT we divided the cohort in responders and non-responders. Responders were those who achieved the criteria for CRT in the emapalumab trial for pHLH [1] after conventional therapy. Conventional therapy was defined as glucocorticoids, cyclosporine-A, intravenous Ig and/or anakinra at the dose of ≤ 5mg/kg/day. Non-responders were those who died, those who did not achieve CRT and those who required additional or prolonged (more than 1 month) treatment.

**Results:** 82 sHLH patients, 49 males, with a median age at disease onset of 5.9 years, were included. 21 patients had HLH secondary to rheumatic/inflammatory diseases (other than sJIA), 4 secondary to SLE, 2 to JDM, 1 to systemic vasculitis, 1 to Crohn’s disease, 1 to Sjögren syndrome, 1 to antiphospholipid antibody syndrome and 11 to other rheumatic/inflammatory diseases. 39 were secondary to infections, 8 to other conditions, such as metabolic disorders or immunodeficiencies and 14 had no evidence of underlining disease (unknown). The mortality rate of the entire cohort was 27% (Table 1). To analyse CRT, 9 patients, who did not receive immunosuppressive therapy, as they responded rapidly to the treatment of the HLH trigger, were excluded from this analysis. There were 32 (44%) responders: of those 7 had HLH secondary to rheumatic/inflammatory diseases, 22 to infections, 3 of unknown origin and none secondary to other conditions. 24/32 patients achieved CRT at 3 months. 41 (56%) were non-responders, included those who died (n=22), 14 of whom had HLH secondary to rheumatic/inflammatory diseases (3 to SLE), 11 to infections, 7 to other conditions and 9 of unknown. In the entire cohort only 7 patients received anakinra, 4 of them subcutaneously at the dose of 3 to 5 mg/kg/day and 3 received intravenous anakinra up to 10 mg/kg/day to treat the sHLH episode. Of the 3 patients treated with high-dose anakinra, 2 died.

**Conclusion:** The mortality rate of this cohort is high (27%) similar to those reported in smaller series. The percent of patients achieving complete response at 3 months is less than 50%, underlying the severity of the disease and the poor response to unspecific immunosuppression. Even though the use of anakinra is reported to be effective in some cases of sHLH, particularly in sHLH with an underlying rheumatic disease, in our cohort only few patients with a very severe disease were treated with anakinra.

**Reference**

[1] Locatelli F. et al N Engl J Med. 2020 May 7;382(19):1811-1822.

**Disclosure of Interest**

C. Bracaglia Consultant for: Sobi, Novartis, R. Pecoraro: None declared, D. Pires Marafon: None declared, A. De Matteis: None declared, G. Marucci: None declared, M. Pardeo: None declared, F. De Benedetti Consultant for: Abbvie, SOBI, Novimmune, Novartis, Roche, Pfizer

**Table 1 (abstract O25).** Mortality rate of sHLH patients

	N° of patients	Survivors	Died	Mortality rate cause-specific (%)	Mortality rate overall (%)
**All sHLH**	**82**	**60**	**22**	**-**	**26.8%**
**Secondary to rheumatic/inflammatory disease**	**21**	**15**	**6**	**28.6%**	**7.3%**
- SLE	4	3	1	25%	0.01%
- Other rheumatic/inflammatory diseases	17	12	5	29%	6.1%
**sHLH (excluding rheumatic/inflammatory diseases)**	**61**	**45**	**16**	**26.2%**	**19.5%**
- Infections	39	33	6	15.4%	7.3%
- Others	8	2	6	75.0%	7.3%
- Unknown	14	10	4	28.6%	4.9%

### O26 Activated CD8+ T cells discriminates patients with macrophage activation syndrome from patients with active systemic juvenile idiopathic arthritis

#### G. Prencipe^1^, A. De Matteis^2^, M. Colucci^3^, M. N. Rossi^1^, I. Caiello^1^, M. Pardeo^2^, C. Bracaglia^2^, F. De Benedetti^2^

##### ^1^Laboratory of Immuno-Rheumatology; ^2^Division of Rheumatology; ^3^Renal Diseases Research Unit, Bambino Gesù Children's Hospital, ROMA, Italy

###### **Correspondence:** G. Prencipe

**Introduction:** T cell activation profiling has been recently demonstrated to be able to distinguish patients with primary and infection-associated hemophagocytic lymphohistiocytosis (HLH) from patients with sepsis (1).

**Objectives:** In this study, we aimed to evaluate whether activated CD8^+^ T cell profile also characterizes patients with macrophage activation syndrome (MAS) in the context of systemic juvenile idiopathic arthritis (sJIA) and whether it is able to distinguish patients with MAS from those with active sJIA.

**Methods:** Flow cytometric analyses were performed on peripheral blood mononuclear cells isolated from children with inactive sJIA (n=17), active sJIA (n=27), MAS (n=14) and with HLH secondary to infection (n=7).

**Results:** To assess the activation status of CD8+ T lymphocytes, we evaluated the expression of the activation markers HLA-DR and CD38. In patients with MAS, the frequency of CD38^high^/HLA-DR^+^_,_ gated on CD8^+^ CD3^+^ cells, was significantly higher compared to those observed in patients with active and inactive sJIA (mean ± SD: 38.4 ± 21.1 % vs 6.9 ± 7.6 % and 2.6 ± 4.2 %, respectively). Receiver operating characteristic (ROC) curve analysis demonstrated that frequency of CD38^high^/HLA-DR^+^ CD8^+^ T cells was able to reliably discriminate patients with MAS from those with active sJIA [area under the curve (AUC) of 0.96 (95%CI 0.90-1.00, p<0.001)]. No statistically significant differences in the frequency of CD38^high^/HLA-DR^+^ CD8^+^ T cells between MAS and HLH secondary to infection were observed. In addition, CD38^high^ expressing cells represented the major source of IFNγ among CD8^+^ T cells. The percentage of CD38^high^/HLA-DR^+^ CD8^+^ T cells detected in MAS patients correlated with laboratory parameters of disease severity, including haemoglobin, lactate dehydrogenase and ferritin.

**Conclusion:** We found that CD8^+^ T cell activation status also characterizes patients with MAS in the context of sJIA, demonstrating that T cell activation status in HLH does not vary depending on the underlying condition/trigger. Moreover, assessment of percentage of CD38^high^/HLA-DR^+^ CD8^+^ T cells represents a valid tool for an accurate identification of patients with MAS. The correlation, in MAS patients, between the increased number of IFNγ-producing CD38^high^/HLA-DR^+^ CD8^+^ T cells and the laboratory parameters of the disease, further confirms the pathogenic role of IFNγ in HLH.

1. Chaturvedi V, Marsh RA, Zoref-Lorenz A, Owsley E, Chaturvedi V, Nguyen TC, Goldman JR, Henry MM, Greenberg JN, Ladisch S, Hermiston ML, Jeng M, Naqvi A, Allen CE, Wong HR, Jordan MB. T-cell activation profiles distinguish hemophagocytic lymphohistiocytosis and early sepsis. Blood. 2021 Apr 29;137(17):2337-2346. doi: 10.1182/blood.2020009499.

**Disclosure of Interest**

G. Prencipe: None declared, A. De Matteis: None declared, M. Colucci: None declared, M. Rossi: None declared, I. Caiello: None declared, M. Pardeo: None declared, C. Bracaglia Consultant for: SOBI, Novartis, F. De Benedetti Consultant for: Abbvie, SOBI, Novimmune, Novartis, Roche, Pfizer

### O27 Development of clinical definitions of refractory disease trajectories in systemic JIA

#### R. Erkens^1^, A. Grom^2^, R. Sinha ^3^, Y. Kimura^4^, C. Towe ^2^, A. Consolaro^5^, S. W. Canna^6^, C. Bracaglia^7^, F. De Benedetti^7^, C. Wouters^8^, K. Tenbrock^9^, A. Horne^10^, R. Yeung^11^, D. Föll^12^, N. Wulffraat^1^, G. Schulert^2^, B. Vastert^1^

##### ^1^Wilhelmina kinderziekenhuis, UMC Utrecht, Utrecht, Netherlands; ^2^Cincinnati Children’s Hospital; ^3^Systemic JIA Foundation, Cincinnati; ^4^Hackensack UMC, Hackensack, United States; ^5^IRCCS Istituto Giannina Gaslini, Genova, Italy; ^6^The Children’s Hospital of Philadelphia, Philadelphia, United States; ^7^Ospedale Pediatrico Bambino Gesù, Rome, Italy; ^8^UZ Leuven, Leuven, Belgium; ^9^RWTH Aachen University, Aachen, Germany; ^10^Karolinska Institutet, Stockholm, Sweden; ^11^The Hospital for Sick Children, University of Toronto, Toronto, Canada; ^12^University of Münster, Münster, Germany

###### **Correspondence:** R. Erkens

**Introduction:** Systemic Juvenile Idiopathic Arthritis (sJIA) is a distinct and heterogeneous disease presently classified under the umbrella of JIA, with some patients following a monophasic remitting course, while others have persistent disease with chronic organ- and life-threatening complications. Though biologic therapies have revolutionized treatment and improved outcomes, recent long-term follow-up studies report significant numbers of children with persistently active disease. Even in 2020, a significant number of sJIA patients show incomplete responses to targeted biological therapies and need to try multiple sequential treatments. Treatment-refractory sJIA patients often face severe and life-threatening complications including macrophage activation syndrome (MAS), sJIA associated lung disease (sJIA-LD), and destructive arthritis. Due to a lack of clinical trials, there is little evidence to guide treatment, making treatment of refractory sJIA patients challenging. Developing a broadly accepted consensus on the definitions of refractory sJIA will be an important first step towards collaborative international research.

**Objectives:** To develop widely accepted definitions of refractory sJIA to enable international collaborative studies of refractory sJIA patients.

**Methods:** During the 2019 NextGen Meeting of the Systemic JIA Foundation (Canna et al. Pediatr Rheumatol. 2020), a group of sJIA experts discussed preliminary definitions of refractory disease trajectories (table 1). There is overlap in these disease states, with a strong association between MAS and sJIA-LD, and active sJIA is a risk factor for developing MAS. Currently, we are performing a systematic literature review in preparation for consensus processes including an international Delphi survey followed by a consensus meeting of experts to develop broadly acceptable definitions of refractory sJIA.

**Results:**

**Conclusion:** There is an unmet need for a better understanding and definition of refractory disease in sJIA. A consensus based definition is being developed together with patients and caregivers (incl. the Systemic JIA Foundation). Accepted definitions will then enable collaborative research aimed at understanding the disease mechanisms in sJIA patients fitting these definitions. This knowledge can then be translated into targeted therapeutic strategies, which are urgently needed to improve the outcomes and daily life of these patients.

**Disclosure of Interest**

R. Erkens: None declared, A. Grom: None declared, R. Sinha : None declared, Y. Kimura Grant / Research Support from: Genentech, C. Towe Consultant for: Pediatric ILD Advisory Board for Boehringer Ingelheim, A. Consolaro Grant / Research Support from: Pfizer and AlfaSigma, Speaker Bureau of: AbbVie and Pfizer, S. Canna Grant / Research Support from: Immvention Therapeutix, AB2Bio and Novartis, Consultant for: Simcha Therapeutics, C. Bracaglia Speaker Bureau of: SOBI and Novartis, F. De Benedetti: None declared, C. Wouters Grant / Research Support from: GSK immune-inflammation, Pfizer, Novartis and Roche, Consultant for: advisory board Sobi and Novartis, K. Tenbrock Grant / Research Support from: Pfizer and Novartis, Consultant for: BMS, Pfizer and Novartis, A. Horne Speaker Bureau of: Sobi and Novartis, R. Yeung: None declared, D. Föll Speaker Bureau of: Novartis and Sobi, N. Wulffraat Grant / Research Support from: Sobi, Consultant for: UCB and Pfizer, G. Schulert Speaker Bureau of: Novartis, B. Vastert Grant / Research Support from: Sobi, Consultant for: Sobi and Novartis

**Table 1 (abstract O27).** Preliminary definitions of refractory disease courses in sJIA

Description	Proposed definitions
**Refractory sJIA arthritis**	Arthritis failing to respond to both IL-1 and IL-6 therapy, defined as continued disease activity requiring maintenance therapy with glucocorticoids (GC) (or inability to wean GC)
**sJIA-MAS**	· sJIA related MAS, requiring maintenance therapy or long term adjunctive therapy with GC OR · Recurrent (≥2 episodes) sJIA related MAS
**sJIA-LD**	· Suspected sJIA-LD: Objective findings on clinical exam (incl. but not limited to tachypnea, cough or clubbing); OR diffuse abnormalities on chest imaging* · Probable sJIA-LD: Both clinical findings and chest imaging findings as above; OR pulmonary hypertension as measured by echocardiogram · Definite sJIA-LD: tissue biopsy consistent with ILD, PAP/ELP or PAH *not due to LD that preexisted sJIA diagnosis, infection or other identifiable cause

### O28 Naive CD4+ t cell differentiation in systemic juvenile idiopathic arthritis is skewed towards a peripheral t helper cell phenotype

#### J. Kuehn, S. Schleifenbaum, A. Hellige, C. Hinze, H. Wittkowski, D. Foell, C. Kessel

##### Pediatric Rheumatology & Immunology, University Children's Hospital, Muenster, Germany

###### **Correspondence:** J. Kuehn

**Introduction:** In terms of pathogenesis, systemic juvenile idiopathic arthritis (sJIA) is a unique JIA entity in that it is thought to feature characteristics of both autoinflammatory and autoimmune diseases. A bi-phasic model of disease progression has been proposed, where initial systemic inflammation may develop to chronic destructive arthritis^1^.

**Objectives:** Our previous work indicated low interferon gamma (IFNg) expression by CD4^pos^ T helper (Th) cells in sJIA^2^, which echoed earlier findings on low IFNg immune cell exposure in disease^3^ but is in sharp contrast to sJIA-associated macrophage activation syndrome (MAS) with IFNg as a central driver of cytokine storm and anemia^4^. Thus, appart from MAS we hypothesized that due to the likely lack of cognate sJIA-associated T cell antigens in an *in vivo* environment of proinflammatory mediators with the power to drive T cell polarization in different directions, CD4^pos^ T cells in sJIA may suffer from aberrant or incomplete polarization, which may translate into insufficient IFNg expression.

**Methods:** Naïve Th cells were isolated from pediatric healthy controls (HC, n = 12) and active (excluding MAS) and inactive sJIA patients (n = 21) and were cultured under various Th1, Th17 and Tfh polarizing conditions. Following super-stimulation with PMA/ionomycin, cell surface marker, transcription factor and cytokine expression was analyzed by flow cytometry, cyto-/chemokine release was quantified by multiplexed bead array assay or ELISA. For *ex vivo* studies, PBMCs were stimulated with PMA/ionomycin and analyzed by flow cytometry.

**Results:** Among naive peripheral CD4^pos^ T cells obtained from sJIA patients, we found an impaired IFNg expression and Th1 differentiation compared to healthy controls, when exposed to respective polarizing cytokines. Low IFNg production was linked to suboptimal Eomes expression. Surprisingly, we found a substantially increased release of IL-21, which was particularly pronounced under Th1 differentiation conditions and correlated to low IFNg and Eomes expression levels. Therefore, we tested a skewing of naive sJIA T cell differentiation towards T follicular helper (Tfh) cells (PD-1^pos^ICOS^pos^CXCR5^pos^) as a major source of IL-21 in the Th cell compartment. Under Th1 and Tfh differentiating conditions, we observed a strong up-regulation of the Tfh markers PD-1 and ICOS as well as the hallmark cyto-/chemokines IL-21 and CXCL13. PD-1 and ICOS expression levels were tightly correlated to high IL-21 expression and particularly evident in inactive disease patients‘ cells. Throughout, we only observed marginal expression of CXCR5. *Ex vivo*, we observed an expanded Tfh cell compartment and IL-21 expression among inactive disease sJIA patients, whereas active disease patients CD4^pos^ cells rather revealed signs of exhaustion (PD-1^hi^ICOS^neg^).

**Conclusion:** In sJIA, naïve T helper cell differentiation appears skewed towards a peripheral T helper phenotype (PD-1^hi^ICOS^pos^CXCR5^neg^), which has been described to occur in context of chronic inflammation^5,6^. In sJIA pathogenesis this may represent an echo of autoimmunity, which could shed light on the mechanisms driving the progression towards chronic destructive arthritis.

**References**

(1) Nigrovic PA, Arthritis Rheumatol, 2014

(2) Kessel C et al., Arthritis Rheumatol, 2017

(3) Sikora K et al., Arthritis Rheum, 2012

(4) Crayne et al., Front Immunol, 2019

(5) Rao et al., Nature, 2017

(6) Bocharnikov et al., JCI Insight, 2019

**Disclosure of Interest**

None declared

### O29 Efficacy and safety of secukinumab in enthesitis-related arthritis and juvenile psoriatic arthritis in a randomised, double-blind, placebo-controlled, treatment withdrawal, phase 3 study (Junipera)

#### N. Ruperto^1^, I. Foeldvari^2^, E. Alexeeva^3^, N. A. Ayaz^4^, I. Calvo^5^, O. Kasapcopur^4^, V. Chasnyk^6^, M. Hufnagel^7^, Z. Zuber^8^, G. Schulert^9^, S. Ozen^10^, A. Popov^11^, A. Ramanan^12^, C. Scott^13^, B. Sozeri^14^, E. Zholobova^15^, X. Zhu^16^, S. Whelan^17^, L. Pricop^16^, A. Ravelli^18^, A. Martini^1^, D. J. Lovell^19^, H. Brunner^19^, on behalf of PRINTO and PRCSG

##### ^1^IRCCS Istituto G. Gaslini, Genova, Italy; ^2^Hamburger Zentrum fuer Kinder und Jugendrheumatologie, Hamburg, Germany; ^3^National Scientific and Practical Center of Children's Health, Moscow, Russian Federation; ^4^Istanbul University, Istanbul, Turkey; ^5^Hospital Universitario i Politecnic La Fe Valencia, Valencia, Spain; ^6^St. Petersburg State Pediatric Medical Academy, St. Petersburg, Russian Federation; ^7^University of Freiburg, Freiburg, Germany; ^8^Wojewodzki Specjalistyczny Szpital Dzieciecy im Sw Ludwika, Krakow, Poland; ^9^Universtiy of Cincinnati, Ohio, United States; ^10^Hacettepe University, Ankara, Turkey; ^11^Ural State Medical University, Yekaterinburg, Russian Federation; ^12^University of Bristol, Bristol, United Kingdom; ^13^University of Cape Town, Cape Town, South Africa; ^14^Health Sciences University, Istanbul, Turkey; ^15^First Moscow State Medical University, Moscow, Russian Federation; ^16^Novartis Pharmaceutical Corporation, East Hanover, United States; ^17^Novartis Ireland Ltd, Dublin, Ireland; ^18^Istituto Giannina Gaslini, Genova, Italy; ^19^University of Cincinnati, Cincinnati, United States

###### **Correspondence:** N. Ruperto

**Introduction:** Enthesitis-related arthritis (ERA) and juvenile psoriatic arthritis (JPsA) are two ILAR categories of juvenile idiopathic arthritis (JIA) with adult correlates of axial spondyloarthritis (axSpA) and adult psoriatic arthritis (PsA), respectively.^1,2^ Secukinumab (SEC) improved signs and symptoms of axSpA, PsA and non-radiographic axSpA.^3-5^

**Objectives:** Evaluate efficacy and safety of SEC in patients (pts) with active ERA and JPsA

**Methods:** In open label (OL) treatment period (TP) 1 (12 weeks), pts (2 to <18 years) were administered SEC (s.c., 75 mg in pts <50 kg and 150 mg in ≥50 kg) at baseline (BL), and at Weeks 1–4, 8 and 12. Responders (pts who achieved at least JIA ACR 30 in TP1) were randomised to double-blinded SEC or placebo (PBO) treatment q4w until experiencing a disease flare or up to Week 104 in TP2. Primary endpoint was time to flare in TP2 and key secondary endpoints were JIA ACR 30/50/70/90/100 responses, inactive disease status, Juvenile Arthritis Disease Activity Score (JADAS), enthesitis count and safety. Data are as observed.

**Results:** In TP1, 86/97 (89%; ERA, n=52; JPsA, n=34, mean age: 13.1 years, females: 33.7%) of pts received OL SEC treatment. BL mean JADAS-27 score and enthesitis count were 15.1 and 2.6, respectively. JIA ACR 30/50 responses were 90.4% and 86.7%, respectively at the end of TP1 (Table). In TP2, 10 flares in SEC and 21 flares in PBO were observed. SEC vs PBO treated pts had a significantly (*P*<0.001) longer time to flare (HR: 0.28; 95% CI: 0.13–0.63) with a 72% risk of flare reduction in TP2. SEC safety profile was similar to adults with no new safety signals.

**Conclusion:** In pts with active ERA and JPsA, efficacy of SEC was demonstrated with a significantly longer time to flare vs PBO with sustained improvement of signs and symptoms up to Week 104 and a favourable safety profile.

**References**

1. Colbert RA. Nat Rev Rheumatol. 2010;6:477–85

2. Martini A, et al. J Rheumatol. 2019;46:190–7

3. McInnes IB, et al. Lancet. 2015;386:1137–46

4. Baeten D, et al. N Engl J Med. 2015;373:2534–48

5. Deodhar A, et al. Arthritis Rheumatol. 2021;73:110–20

**Trial registration identifying number:** NCT03031782

**Patient Consent Received**

No

**Disclosure of Interest**

N. Ruperto Consultant for: Ablynx, Astrazeneca-Medimmune, Bayer, Biogen, Boehringer, Bristol Myers and Squibb, Celgene, Eli-Lilly, EMD Serono, Glaxo Smith and Kline, Hoffmann-La Roche, Janssen, Merck, Novartis, Pfizer, R-Pharma, Sinergie, Sobi and UCB, Speaker Bureau of: Ablynx, Astrazeneca-Medimmune, Bayer, Biogen, Boehringer, Bristol Myers and Squibb, Celgene, Eli-Lilly, EMD Serono, Glaxo Smith and Kline, Hoffmann-La Roche, Janssen, Merck, Novartis, Pfizer, R-Pharma, Sinergie, Sobi and UCB, I. Foeldvari Consultant for: Novartis, Speaker Bureau of: Novartis, E. Alexeeva Speaker Bureau of: Novartis, Pfizer, Sanofi, MSD, AMGEN, Eli Lilly, Roche, N. Ayaz: None declared, I. Calvo Consultant for: Sobi, Novartis, Abbvie, GlaxoSmithKline, Pfizer, Amgen, Clementia, Speaker Bureau of: Sobi, Novartis, Abbvie, GlaxoSmithKline, Pfizer, Amgen, Clementia, O. Kasapcopur: None declared, V. Chasnyk: None declared, M. Hufnagel: None declared, Z. Zuber: None declared, G. Schulert Consultant for: Sobi, Novartis, S. Ozen: None declared, A. Popov: None declared, A. Ramanan Speaker Bureau of: Roche, Sobi, Eli Lilly, UCB, Novartis, C. Scott: None declared, B. Sozeri: None declared, E. Zholobova Speaker Bureau of: Abbvie, Pfizer, Roche, X. Zhu Employee of: Novartis, S. Whelan Shareholder of: Novartis, Employee of: Novartis, L. Pricop Shareholder of: Novartis, Employee of: Novartis, A. Ravelli Consultant for: Abbvie, Bristol-Myers Squibb, Pfizer, Hoffmann-LaRoche, Novartis, Centocor, Angelini Holding, Reckitt Benckiser, Speaker Bureau of: Abbvie, Bristol-Myers Squibb, Pfizer, Hoffmann-LaRoche, Novartis, Centocor, Angelini Holding, Reckitt Benckiser, A. Martini Consultant for: Eli Lilly, EMD Serono, Janssen, Novartis, Pfizer, Abbvie, Speaker Bureau of: Eli Lilly, EMD Serono, Janssen, Novartis, Pfizer, Abbvie, D. Lovell Consultant for: AstraZeneca, Wyeth, Amgen, Abbott, Pfizer, Hoffmann-La Roche, Novartis, UBC, Takeda, Janssen, GlaxoSmithKline, Boehringer Ingelheim, Celgene, Bristol Myers Squibb, AbbVie, Forest Research, Speaker Bureau of: AstraZeneca, Wyeth, Amgen, Abbott, Pfizer, Hoffmann-La Roche, Novartis, UBC, Takeda, Janssen, GlaxoSmithKline, Boehringer Ingelheim, Celgene, Bristol Myers Squibb, AbbVie, Forest Research, H. Brunner Consultant for: Aurina, AbbVie, Astra Zeneca-Medimmune, Biogen, Boehringer, Bristol-Myers Squibb, Celgene, Eli Lilly, EMD Serono, GlaxoSmithKline, F. Hoffmann-La Roche, Merck, Novartis, R-Pharm, Sanofi, Pfizer

**Table 1 (abstract O29).** Efficacy of secukinumab in Treatment Periods 1 and 2 (Key secondary endpoints)

Efficacy Outcomes, %		TP1	TP2^¥^		
		SEC (N=83)^^^	SEC (N=37)	PBO (N=37)	*P*-value
JIA ACR	30	90.4	89.2	64.9	0.014
	50	86.7	78.4	62.2	0.152
	70	69.9	67.6	43.2	0.042
	90	39.8	51.4	40.5	0.431
	100	25.3	43.2	37.8	0.745
Inactive disease^#^		36.1	47.2	37.8	0.500
JADAS-27, mean (SD)		15.1 (7.2)	14.6 (8.1)	13.3 (5.8)	NA
Enthesitis count, mean change from BL (SD)		−1.8 (2.3)	−2.1 (2.0)	−1.9 (1.2)	NA

*P*-values: Cochran-Mantel-Haenszel test, adjusted for analysis factors, JIA category (ERA/ JPsA) and MTX use at BL

^¥^The N numbers are values at the end of TP2

^^^Efficacy outcomes (%) in TP1 calculated in patients with evaluable data at Wk 12 (N=83)

^#^Inactive disease: Definition adapted from JIA ACR criteria of Wallace et al., 2011. N=36 for SEC at the end of TP2

### O30 Single-cell genomics reveals a shared monocyte interferon program in a subset of patients with systemic juvenile idiopathic arthritis, macrophage activation syndrome and lung disease

#### E. L. Verweyen^1^, K. Thakkar^2,3^, K. Chetal^2^, S. Dhakal^1^, A. A. Grom^1,4^, N. Salomonis^2^, G. S. Schulert^1,4^

##### ^1^Division of Rheumatology; ^2^Division of Biomedical Informatics, Cincinnati Children's Hospital Medical Center; ^3^Department of Pharmacology and Systems Physiology, University of Cincinnati College of Medicine; ^4^Department of Pediatrics, Cincinnati Children's Hospital Medical Center, Cincinnati, United States

###### **Correspondence:** E. L. Verweyen

**Introduction:** Systemic juvenile idiopathic arthritis (SJIA) is a clinically heterogenous disease and can be complicated by macrophage activation syndrome (MAS) and lung disease (LD) thought to be driven by interferon signaling, though the contributing cell populations and distinctions between IFNγ and IFNα/β are undefined.

**Objectives:** To identify novel prognostic transcripts and potential patient subtypes, we aimed to characterize single-cell heterogeneity and patient-specific transcriptomics responses from the peripheral blood of children with SJIA, MAS and LD.

**Methods:** 10x Genomics single-cell RNA Sequencing (scRNA-Seq) was performed on PBMCs from 7 active SJIA and 5 inactive SJIA patients, 2 SJIA-MAS and 6 SJIA-LD patients and 5 healthy controls. Integration analyses were performed with the software Seurat 3 to identify discrete cell populations while correcting for donor and disease-level differences. To identify subsets of patients with cell-type specific signatures, we developed a new hybrid supervised/unsupervised computational pipeline in the software AltAnalyze, called cellHarmony 2.0, designed specifically for large cohort single-cell genomic studies.

**Results:** scRNA-Seq analysis was performed on a total of 234,128 individual cells (ranging from 6,662-12,647 cells/patient), with a mean number of 21,637 genes detected per sample. To assess cell-population level differences, we identified and annotated based on marker genes 34 discrete cell populations across all submitted samples. This indicated a consistent increase in Natural Killer (NK) cells and decrease in naïve and regulatory T cells in SJIA-LD, with the distribution of cells from inactive SJIA patients similar to that of controls. To exploit anticipated heterogeneity within this cohort, we applied our new cell-type aware patient subtype discovery algorithm cellHarmony 2.0. We computed an aggregate cell signature for all cell populations or pseudo-bulks (n=34) for each patient and their associated fold differences relative to matched control cell clusters, and performed unsupervised clustering of the pseudo-bulks to identify patient subtypes associated within one or more cell types. This analysis uncovered 11 pseudo-clusters of cell type gene expression differences, both shared and unique across the patients. Specifically, pseudo-cluster 4 was defined by IL-2 mediated signaling genes, composed of mostly NK cells from all SJIA subtypes except MAS. SJIA-MAS PBMCs were almost exclusively represented in three separate pseudo-clusters that contained genes mediating T-cell receptor activation, immune response and interferon signaling. Finally, pseudo-cluster 8 was composed of mainly monocytes/macrophages with specific upregulation of IFNα/β induced genes IFITM3, IFI6 and ISG15, only in active SJIA, SJIA-MAS and SJIA-LD.

**Conclusion:** Unsupervised single-cell cohort analysis provides new opportunities to uncover novel disease molecular programs and pathways in clinically heterogenous patient groups. Here, we found active SJIA, SJIA-MAS and SJIA-LD PBMCs have distinct monocytic responses characterized by upregulation of interferon-induced genes, highlighting the role for both IFNγ and IFNα/β in driving disease pathogenesis.

**Patient Consent Received**

No

**Disclosure of Interest**

E. Verweyen: None declared, K. Thakkar: None declared, K. Chetal: None declared, S. Dhakal: None declared, A. Grom Consultant for: Novartis, Sobi, AB2Bio, Cerecor, N. Salomonis: None declared, G. Schulert Speaker Bureau of: Novartis

## Lightning talks: Juvenile dermatomyositis, Scleroderma and related syndromes, Systemic lupus erythematosus and antiphospholipid syndrome, Uveitis

### O31 Investigating novel mechanisms of t cells in the pathogenesis of juvenile dermatomyositis

#### L. R. Marshall^1^, E. C. Rosser^2^, C. T. Deakin^1^, D. Eleftheriou^1^, E. J. Sumner^1^, B. Jebson^1^, K. O'Brien^1^, Q. Wu^1^, L. R. Wedderburn^1^, on behalf of JDRG

##### ^1^IR, UCL Great Ormond Street Institute of Child Health; ^2^UCL Centre for Adolescent Rheumatology Versus Arthritis, London, United Kingdom

###### **Correspondence:** L. R. Marshall

**Introduction:** Juvenile Dermatomyositis (JDM) is a rare autoimmune disease causing skin and muscle inflammation with an average onset of 7 years old. At present, JDM aetiology is poorly understood and current treatment options are not evidence based. This highlights the need for research investigating underlying disease pathogenesis. A skewed T helper (Th)17 phenotype in CD4+ T cells resulting in a Th1/17 imbalance has been observed in both child and adult-onset immune-mediated diseases including rheumatoid arthritis, and multiple sclerosis.

**Objectives:** The aim of this project is to investigate whether a Th1/17 imbalance can be observed in patients with JDM compared to age/sex-matched child healthy controls (CHC).

**Methods:** Expression of IL-17 and IFNγ in CD4+ T cells within peripheral blood mononuclear cells (PBMC) from JDM pre-treatment (JDM Pre, n=7), JDM on-treatment (JDM On, n=28) and CHC (n=22) was assessed by flow cytometry after stimulation with PMA/Ionomycin/Brefeldin A (P/I/B) for 4 hours. For secreted cytokine production, isolated CD4+ T cells were isolated by magnetic separation and stimulated with anti-CD3 or anti-CD3/anti-CD28 for 36 hours in the presence of IL-2. Supernatants were analysed for secreted IL-17 and IFNγ and measured by cytokine bead array. In parallel, extracellular Th1 (CD3+CD4+CXCR3+CCR6-), Th17 (CD3+CD4+CXCR3-CCR6+) and Treg (CD3+CD4+CD127-CD25hi) subsetting was carried out using flow cytometry and proliferative capacity of T cells was assessed following stimulation using Ki67.

**Results:** Both intracellular cytokine staining and stimulation experiments to assess secreted cytokine revealed a decreased trend of IFN-γ production in JDM compared to CHC within CD4+ T cells, regardless of treatment status. Ratio analysis of CD4+IFNγ+ to CD4+IL-17+ cells within peripheral blood after PMA/Ionomycin stimulation demonstrated that the JDM CD4+ T-cell phenotype is significantly skewed towards Th17 cells (p=<0.001) compared to CHC. A Th17 skew was also seen when analysing surface markers for Th1 and Th17 on JDM pre CD4+ T cells compared to controls (p=<0.0001). Central and Effector Memory compartments within CD4+ T cells were reduced in JDM Pre patients compared to controls (p=0.02, p=<0.001 respectively).

**Conclusion:** To summarise, our novel findings show a lack of Th1 response, via IFNγ, in JDM CD4+ T cells compared to CHC. Whilst results show promising avenues for further investigation there are no definitive explanations for this low Th1 response at present. Future work aims to investigate memory and naive compartments within JDM Pre CD4+ T cells in addition to testing of Th1 markers within the muscle. Additionally, other immune and metabolic pathways that may explain this Th17 skew could be targeted to restore IFNγ loss in JDM patients.

**Disclosure of Interest**

None declared

### O32 Oxidised mitochondrial DNA induces an interferon response in JDM: a new therapeutic target

#### M. Wilkinson^1,2,3^, E. C. Rosser^2,4^, M. Orford^5^, C. Wincup^2,4^, T. C. R. McDonnell^6^, G. Otto^3,7^, D. Kelberman^3,7^, S. Castellano^3,7^, L. R. Wedderburn^1,2,3^, S. Eaton^5^, C. T. Deakin^1,2,3^

##### ^1^Infection, Immunity and Inflammation Programme Research and Teaching Department, UCL Gosh Institute of Child Health; ^2^Centre for Adolescent Rheumatology Versus Arthritis at UCL UCLH and GOSH, University College London; ^3^NIHR Biomedical Research Centre at GOSH, GOSH; ^4^Centre for Rheumatology Research, University College London; ^5^Developmental Biology and Cancer Programme; ^6^Centre for Rheumatology Research; ^7^Genetics and Genomic Medicine Programme, UCL Gosh Institute of Child Health, London, United Kingdom

###### **Correspondence:** M. Wilkinson

**Introduction:** JDM is a rare childhood autoimmune myositis that presents with proximal muscle weakness and associated skin changes. There is an unmet need to develop new targeted treatments. A key pathological feature of JDM is a strong type 1 interferon (IFN1) signature, identifying and understanding the mechanisms up-stream is important for the development of new therapeutics.

**Objectives:** This study aimed to identify dysregulated biological processes up-stream of IFN1 by RNA-sequencing in JDM and develop functional assays to confirm these pathways.

**Methods:** Peripheral blood samples were obtained from JDM patients [pre-n=10 on-n=12 treatment] and age/sex-matched child healthy controls (CHC) [n=8]. CD4^+^, CD8^+^, CD14^+^ and CD19^+^ cells were sorted by flow-cytometry from PBMC, and RNA was extracted and RNA-sequenced. Mitochondrial superoxide was assessed in CD14+ monocytes by flow cytometry using MitoSox quantified by median fluorescence intensity (MFI), JDM pre-treatment [n=5], on-treatment [n=6] and CHC [n=3]. Oxidised mitochondrial DNA (oxmtDNA) from CD14+ monocyte isolated mitochondria was measured by western dot-blot quantified using densitometry, JDM [n=10] and CHC [n=11]. Plasma cell-free mtDNA was quantified as copy number of the mitochondrial gene *MT-CO3*per ml of plasma for CHC [n=16] and JDM pre-treatment [n=45] using standard curve qPCR. HC PBMC samples [n=6] were cultured with IFN-α or oxmtDNA (+ LL37) with or without TLR-9 antagonist or the anti-oxidant *n*-acetyl cysteine (NAC). Post-culture, IFN1 gene expression was measured by qPCR.

**Results:** RNA-seq confirmed a strong IFN1 signature pre-treatment, and demonstrated that genes involved in mitochondrial function were abnormally expressed in both pre- and on-treatment CD14+ cells compared to controls, suggesting that mitochondrial dysfunction is not corrected by current treatment strategies. Validation of RNAseq using flow cytometry and western dot-blot showed that there was an increase in mitochondrial superoxide and oxmtDNA in CD14+ monocytes, JDM pre-treatment vs. CHC (p=0.034) and JDM Vs. CHC (p=0.061). This was complimented by increased plasma cell-free mtDNA was increased in JDM pre-treatment compared to controls (p=0.0076). *In vitro*, oxmtDNA and IFN-α induced a comparative up-regulation IFN1 genes compared to unstimulated control (MX1 (p<0.0001, p<0.0001); RSAD2 (p=0.1508, p=0.001)). Both TLR-9 antagonist and NAC were able to down-regulate IFN1 genes after 24hr of oxmtDNA stimulation, suggesting that both could translate to therapeutic targets (TLR-9 (MX1, p=0.0001; RSAD2, p=0.0374); NAC (MX1, p<0.0001; RSAD2, p=0.00014)).

**Conclusion:** This study establishes that in JDM, monocytes have an increased production of mitochondrial superoxide and oxmtDNA. There is also an increased amount of plasma cell free mtDNA. We have established a mechanism of oxmtDNA induction of IFN1 signature and the potential to block this pathway with TLR-9 antagonist and NAC, identifying these pathways as novel treatment targets. Further work will investigate the mechanistic relationship between IFN1 driven inflammation and altered mitochondrial metabolism in monocytes.

**Patient Consent Received**

Yes

**Disclosure of Interest**

None declared

### O33 Long-term follow-up of juvenile localized scleroderma patients treated with methotrexate-based standardized regimens (consensus treatment plans)

#### S. Li^1^, A. Thammavongxay^2^, M. Ibarra^3^, K. Torok^4^, P. Ferguson^5^, C. E. Rabinovich^6^, R. Fuhlbrigge^7^, K. Stewart^8^, E. Pope^9^, R. Laxer^10^, S. Hong^5^, T. Mason^11^, M. Becker^6^, G. Higgins^12^, F. Dedeoglu^13^, F. the CARRA Legacy Registry Investigators^14^

##### ^1^Pediatrics, Joseph M Sanzari Children's Hospital, Hackensack Meridian School of Medicine; ^2^Bergen Academies, Hackensack; ^3^Children's Mercy Hospital, Kansas City; ^4^Children’s Hospital of Pittsburgh, Pittsburgh; ^5^University of Iowa, Iowa City; ^6^Duke University, Durham; ^7^University of Colorado, Denver; ^8^Texas Scottish Rite, Dallas, United States; ^9^Hospital for Sick Kids; ^10^Hospital for Sick Children, Toronto, Canada; ^11^Mayo Clinic, Rochester; ^12^The Ohio State University, Columbus; ^13^Boston Children's Hospital, Boston; ^14^CARRA, Milwaukee, United States

###### **Correspondence:** S. Li

**Introduction:** Juvenile localized scleroderma (jLS) is a rare chronic inflammatory and fibrosing disease associated with a high risk for morbidity in children. Methotrexate (MTX) has been identified as effective treatment, but data is limited as to the optimal duration, and need for corticosteroid (CS) treatment. The LS group of the Childhood Arthritis and Rheumatology Research Alliance (CARRA) developed standardized regimens (consensus treatment plans, CTPs) for comparative effectiveness studies. The results of a 1-year follow-up of a pilot multi-center study of the MTX-based CTPs were previously reported. Of the 50 patients enrolled, 44 (88%) completed 1 year of follow-up, with 33 (66%) of the patients rated as responders and 11 (22%) as non-responders. We now report on long-term follow-up of these patients.

**Objectives:** To determine the long-term response of jLS patients treated with MTX-based CTPs.

**Methods:** Patients enrolled in the pilot CTP study were eligible to enroll in the long-term extension, with study visits completed at 24 and 36 months. Each patient was evaluated by the same investigator for all 3 years of the study. Treatments and adverse events that occurred since the last study visit were collected. Descriptive analysis was performed, with p values calculated by Z-score or Mann Whitney U test. Inactive disease was defined as physician global assessment of activity (PGA-A) = 0, remission off medicine as PGA-A = 0 with the patient off treatment.

**Results:** Most patients were female (70%), had linear scleroderma (28/44), with a median age of onset 9.4 years. Thirty-seven (74%) of patients completed 3 years of follow-up (Table). PGA-A and skin activity scores (mLoSSI, LSCAM) declined by 12 months and were then stable. Over time, more patients achieved inactive disease, with 20% able to achieve remission off methotrexate. Disease flares were most common in years 1 and 2 when ~20% of patients flared. In addition to 11 non-responders in year 1, 5 additional patients were non-responders in years 2 and 3. Non-responders most commonly received CS and mycophenolate mofetil. The frequency of adverse events decreased 3-fold by year 3.

**Conclusion:** JLS patients treated with MTX-based standardized regimens were found to achieve further improvement in years 2 and 3 with continued treatment, with 20% able to achieve remission off medicine in year 3. Disease flares occurred in each year and overall, 16 (32%) patients were non-responders, requiring additional treatment for disease control. More work is needed to identify the at-risk patients and determine their optimal therapy.

**Disclosure of Interest**

None declared

**Table 1 (abstract O33).** See text for description

	Start of Study	Year 1	Year 2	Year 3
jLS patient number	50 (100)	44 (88)	39 (78)	37 (74)
Inactive disease	0	19 (38)	21 (42)	24 (48)
Remission off medicine	0	0	0	10 (20)
Disease Flare	NA	11 (22)	10 (20)	6 (12)
Adverse Event ≥ grade 2	NA	21 (42)	12 (24)	7 (14)
PGA-A (IQR)	5 (4-6)	1 (0-2)	0 (0-1.3)	0 (0-1)
mLoSSI (IQR)	7 (4-10)	0 (0-1)	0 (0-0)	0 (0-0)
LSCAM (IQR)	7 (4-13.3)	2 (0-4)	0 (0-2)	0 (0-2)

Numbers shown represent number (%) unless otherwise stated. Median scores are shown. IQR: interquartile range; LSCAM: Localized Scleroderma Cutaneous Activity Measure; mLoSSI: modified Localized Scleroderma Severity Index; NA: not applicable; PGA-A: Physician global assessment of activity

### O34 Genetics of age at systemic lupus erythematosus diagnosis

#### R. Carlomagno^1^, F. Liao^1^, J. Cao^2^, D. Dominguez^1^, D. D. Gladman^3^, N. Groot^4^, M. Ishimori^5^, C. Jefferies^5^, D. L. Kamen^6^, S. Kamphuis^4^, M. S. Klein-Gitelman^7^, A. M. Knight^1^, C.-C. J. Lee^5^, D. M. Levy^1^, K. B. Onel^8^, A. Paterson^2^, C. A. Peschken^9^, J. E. Pope^10^, Z. Touma^3^, M. B. Urowitz^3^, D. J. Wallace^5^, J. E. Wither^3^, D. Webber^1^, E. D. Silverman^1,11^, L. T. Hiraki^1,2^

##### ^1^Division of Rheumatology; ^2^Genetics & Genome Biology, Research Institute, The Hospital for Sick Children; ^3^Schroeder Arthritis Institute, Krembil Research Institute, Toronto Western Hospital, Toronto, Canada; ^4^Department of Pediatric Rheumatology, Sophia Children’s Hospital, Erasmus University Medical Center, Rotterdam, Netherlands; ^5^Division of Rheumatology, Department of Medicine, Cedars Sinai Medical Center, Los Angeles; ^6^Division of Rheumatology and Immunology, Medical University of South Carolina, Charleston; ^7^Division of Rheumatology, Department of Pediatrics, Ann & Robert H. Lurie Children’s Hospital of Chicago, Chicago; ^8^Pediatric Rheumatology, Hospital for Special Surgery, New York, United States; ^9^Departments of Medicine and Community Health Sciences, University of Manitoba, Winnipeg; ^10^Department of Medicine, University of Western Ontario, St. Joseph's Health Centre, London; ^11^Division of Translational Medicine Research Institute, The Hospital for Sick Children, Toronto, Canada

###### **Correspondence:** R. Carlomagno

**Introduction:** Genome wide association studies (GWAS) have identified >100 SNPs associated with systemic lupus erythematosus (SLE) risk. There may be additional loci impacting the age of diagnosis.

**Objectives:** The purpose of this study was to identify genetic variants associated with age of SLE diagnosis.

**Methods:** Our cohort included patients who met ACR and/or SLICC classification criteria for SLE, followed at tertiary care centres. We censored patients with missing data on age at diagnosis. Patients were genotyped on the Illumina Multiethnic Array (MEGA) and Illumina Global Screen Array (GSA). Ungenotyped SNPs were imputed using the TopMed reference. We restricted to SNPs with a minor allele frequency (MAF) ≥ 0.01 and imputation quality R^2^≥ 0.3. Ancestry was genetically inferred from principal components (PCs) and ADMIXTURE in reference to 1000 Genome Project. We completed genome-wide linear regression of log-transformed age at SLE diagnosis with GENESIS (genome-wide significance P<5x10^-8^). Multivariate models were adjusted for sex and 5 PCs. We also conducted a GWAS of childhood-onset SLE (cSLE) patients, defined as diagnosis <18 years of age, vs. adult-onset SLE (aSLE), using logistic regression, and adjusted for the same covariates. We conducted sensitivity analyses where we stratified GWAS by cSLE and aSLE, then meta-analyzed results using inverse variance weighting, as well as ancestry-stratified analyses (Europeans, East Asians, Africans, Amerindians and Admixed).

**Results:** Our cohort included 1489 patients, 761 (51%) with cSLE, 1306 (88%) female. The median age at diagnosis was 17.7 years (IQR 14, 31) in the total cohort, 14.1 years (IQR 11.8, 15.8) in the cSLE, and 31.2 years (IQR 24.7, 42) in the aSLE groups. In the total cohort, 576 (39%) were of European ancestry, 278 (19%) East Asian, and 253 (17%) Admixed. We included 11.7M SNPs in GWAS. In the age of SLE diagnosis GWAS, 2 loci on chr16 were genome-wide significant associated to a younger age at diagnosis (top SNP rs11641349, Beta -0.03y, SE 0.15y, P=4.33x10^-8^, MAF 0.2). Both SNPs were intronic to *CCDC113*, a component of centriolar satellites. These loci were also the most significant in the GWAS of cSLE (top SNP rs16959933, OR 1.75 [95% CI: 1.43, 2.14, P=5.45 x10^-8^]). Sensitivity analyses showed similar results, yet they did not reach genome-wide significance with top SNPs rs11641349 (P=3.84x10^-7^) and rs16959933 (P=4.50x10^-7^) in the age group model, and rs11641349 (P=3.52x10^-7^) and rs16959933 (P=4.22x10^-7^) in the ancestry model.

**Conclusion:** In our multiethnic cSLE and aSLE cohort, we identified genome-wide significant loci associated with age at diagnosis and cSLE risk, both intronic to CCDC113 (chr16). Our study requires independent validation.

**Disclosure of Interest**

None declared

### O35 Gene signature fingerprints divide sle patients in subgroups with similar biological disease profiles: a multicenter longitudinal study

#### M. J. Wahadat^1,2^, D. Schonenberg-Meinema^3^, C. van Helden-Meeuwsen^1^, S. van Tilburg^1^, N. Groot^2^, E. Schatorjé^4,5^, E. Hoppenreijs^4,5^, P. Hissink Muller^6^, D. Brinkman^6^, D. Dvorak^7^, M. Verkaaik^2^, K. Bouchalova^7^, M. van den Berg^3^, S. Kamphuis^2^, M. Versnel^1^

##### ^1^Immunology; ^2^Paediatric Rheumatology, University Medical Center Rotterdam, Rotterdam; ^3^Paediatric Immunology, Rheumatology and Infectious Diseases, Amsterdam University Medical Centre, Amsterdam; ^4^Paediatric Rheumatology, st. Maartenskliniek; ^5^Paediatric Rheumatology, Radboud University Medical Center, Nijmegen; ^6^Pediatrics, Division of Pediatric Rheumatology, Leiden University Medical Center, Leiden, Netherlands; ^7^Paediatric Rheumatology,Department of Paediatrics, Faculty of Medicine and Dentistry, Palacky University Olomouc and University Hospital, Olomouc, Czech Republic

###### **Correspondence:** M. J. Wahadat

**Introduction:** Clinical phenotyping and predicting treatment responses in Systemic Lupus Erythematosus (SLE) patients is challenging. Extensive blood transcriptional profiling has identified various gene modules that are promising for stratification of SLE patients based upon aberrantly activated immunological pathways. Yet the feasibility to implement these complicated and expensive tests for use in daily clinical practice of routine clinical laboratories is challenging if not impossible.

**Objectives:** This study aims to translate transcriptomic data into gene signatures suitable for introduction into clinical practice and to associate these signatures with disease activity.

**Methods:** RT-PCR of multiple genes from the Interferon M1.2, Interferon M5.12, neutrophil (NPh)- and plasma cell (PLC) modules, followed by a principle component analysis was used to identify indicator genes per gene signature. Gene signatures were measured in longitudinal samples from two childhood onset SLE cohorts (n=101 and n=34, respectively) and associated with clinical features. Disease activity was measured using SELENA-SLEDAI. Cluster analysis subdivided patients into three fingerprint groups termed 1) all-signatures-low, 2) only IFN high (M1.2 and/or M5.12) and 3) high NPh and/or PLC.

**Results:** All gene signatures were significantly associated with disease activity in cross-sectionally collected samples. The PLC signature showed the highest association with disease activity. Also, in longitudinally collected samples, the PLC signature was associated with disease activity and showed a decrease over time. When patients were divided into the three fingerprints, the highest disease activity was observed in fingerprint-3, the high NPh and/or PLC group. The lowest disease activity was observed in fingerprint-1, the all-signatures-low group. The same distribution could be reproduced in samples from an independent SLE cohort.

**Conclusion:** Gene signatures are associated with disease activity and can be suitable tools to sub-classify patients into groups with similar pathogenically activated immunological pathways.

**Patient Consent Received**

Yes

**Disclosure of Interest**

None declared

### O36 Frequency of uveitis in craniofacial juvenile scleroderma

#### M. K. Osminina, N. S. Podchernyaeva, M. S. Petrova, O. V. Shpitonkova, M. N. Nkcolaeva, T. V. Zubareva

##### Pediatric Department, I. M. Sechenov First Moscow State Medical University, Moscow, Russian Federation

###### **Correspondence:** M. K. Osminina

**Introduction:** Eye involvement in craniofacial juvenile scleroderma (CFJS) reported to present with sclerotic skin changes of eyelids and eyelashes, keratoconjunctivitis sicca and uveitis (anterior segment inflammation), the latter could result in reducing of the visual acuity

**Objectives:** To analyze the frequency of uveitis in CFJS, specifity of its clinical presentation,

and prognosis.

**Methods:** Retrospective analysis of clinical and instrumental observation, including brain magnetic resonance imaging (MRI), electroencephalography (EEG), ophthalmologic examination in particular with slit-lamp was done

**Results:** We observed 105 children with CFJS, aged from 3 to 17 years, the mean age was 10,2 years (M ±2,52), 62 girls and 43 (girls\ boys

boys = 1.4: 1). The majority patients (pts) – 95 have linear skin lesions only on face and head ( linear scleroderma “en coupe de sabre”type - LSCD group), 10 pts have unilateral skin damage of trunk, extremities - (UlSl) affecting face and head.

Uveitis was detected in 12 pts (11,4% ), among them 11 girls and 1 boy. Anterior uveitis was unilateral on the side of skin lesions in 10 pts, bilateral in 2.

In 2 pts uveitis was accompanied by episcleritis, in one-by chorioretinitis. In group UlSl -there were 3 uveitis, detected after 3-5 year of disease duration, while in Lubricant eye drops group -uveitis appeared during this first year of disease outburst, in most cases simultaneously with appearance of skin lesions. In 4 girls uveitis was accompanied by focal seizures, with EEG epileptic pattern and brain foci on MRI on the side of skin damage.90% of uveitis pts were ANF positive.

Pts with uveitis have no specific complains or overt symptoms of eye involvement. It was detected only by ophthalmologic examination screening with slit-lamp we use as a matter of routine in all scleroderma children .Mean follow up period for uveitis children was 5,5 (M ±0.3) years. All of 12 patients achieved remission of uveitis. Significant decrease of activity was seen in 1 month (mo) of therapy, remission in 6 mo. In 2 children uveitis relapsed, after systemic disease modifying antirheumatic therapy (BMART) was stopped by parents. In both cases it was uveitis in LSCD, in 18 and 36 mo after initiation of BMART and in 4-6 weeks (wk) of its discontinuation,the relapses of uveitis were diagnosed after exacerbation of skin lesions. BMART in all uveitis pts includes corticosteroids(CS) orally 1mg\kilo- 12 wk, followed by taping and withdrawal, methotrexate(MTX) 12-15 mg\b.sw. parenterally 30-36 mo; in some cases of severe LSCD MTX in combination with mycophenolate mofetil (600 mg\b.sq. twice a day)-Local treatment - CS and non steroid anti-inflammatory drops, and lubricant eye drops for cornea protection

**Conclusion:** Frequency of uveitis in our cohort of CFJS pts was 11,4%. In all the cases children did not have ocular complains –so called “white uveitis”, but more than 30% uveitis pts had concomitant central nervous system involvement- seizures, brain foci on MRI on the side of skin damage. The prognosis of uveitis in our pts was benign. Pts with CFJS must be regularly ( every 3 mo) checked by ophthalmologist, with obligatory slit-lamp evaluation, compulsorily at the disease debut and before discontinuation of BMART in remission.

**Patient Consent Received**

Yes

**Disclosure of Interest**

None declared

## e-Poster viewing: JIA (oligo, poly, psoriatic)

### P1 A Problem-oriented approach to assessment hand-related problems in patients with juvenile idiopathic arthritis: ICF perspective

#### N. Arman^1^, E. Tarakci^1^, O. Kasapcopur^2^

##### ^1^Department of Physiotherapy and Rehabilitation, Istanbul University-Cerrahpasa, Faculty of Health Science; ^2^Department of Pediatric Rheumatology, Cerrahpasa Medical School, Istanbul University-Cerrahpasa, Istanbul, Turkey

###### **Correspondence:** N. Arman

**Introduction:** A problem-oriented approach (POA) is suggested that the best way to assess and management in patients with chronic disease. POA provides to set a meaningful and purposeful goal for treatment. Creating a problem-oriented assessment algorithm specific to disease provides practical and efficient results in the clinic. There is no defined algorithm for POA in patients with JIA in the literature. So, our team has firstly defined an algorithm of POA in patients with JIA and use it in the clinic. Assessment and management of the hands and wrist that are the most included joints in JIA are also complex. There is little information about the prevalence of hand- and wrist-related symptoms (i.e. pain or stiffness) and impairments (i.e. problems in body function or structure, such as limitation of the range of motion (ROM)), and their resulting activity limitations (i.e. difficulties in dressing) and participation restrictions (i.e. problems with involvement in life situations, such as attending school) in JIA.

**Objectives:** The aim of this study was to use the POA for assessing hand-related problems in patients with JIA.

**Methods:** 216 patients were evaluated for eligibility, 44 patients with JIA who have bilaterally affected wrist joints were included in the study. The POA with three steps was used to evaluate the patients. In the first step in which the problems are determined, hand-related problems of the patients were questioned in 5 categories: pain, limitation of ROM, fatigue, weakness and functional incompetence. The patients were asked to rank these five problems as follows: the most important problem and the least important problem for them. In the second stage of POA, in which body structure and functions are assessed, pain and fatigue were evaluated with the Numeric Rating Scale (NRS). ROM of hand was assessed with a universal goniometer. Muscular strength was estimated at maximal isometric force for the wrist muscles by using a portable handheld dynamometer. Grip and pinch strengths were evaluated by a dynamometer. In the third stage of POA, in which activity and limitations were evaluated, activity performance was evaluated with the Jebsen Taylor Hand Function Test (JTHFT), activity limitation was performed with the Childhood Health Assessment Questionnaire (CHAQ) and Duruoz Hand Index (DHI).

**Results:** 45.5% of the patients reported that their primary problem was functional incompetence, 34.1% of weakness, 13.6% of limitation, 4.5% of fatigue and 2.3% of pain. The score of NRS-pain was five and above in 54.5 % of the patients. Also, the score of NRS-fatigue was five and above in 75 % of the patients. The ROMs of wrist flexion and extension were decreased in 72% of the patients. Also, all scores of the wrist muscle strengths and grip and pinch strengths of all patients were low according to the strength norms of healthy children. In the various grade of scores (1-3 points) according to CHAQ, 79.5% of the patients reported difficulty in dressing activities, 93.2% gripping, 70.5% arising and 51.1% eating. Significant relationships were found in the scores of JTHFT and all grip and pinch strengths, the strength of wrist extension, DHI and CHAQ-total (p<0.05). Pinch strength was also a significant predictor of fatigue severity (p<0.05).

**Conclusion:** We believe that POA is a useful method for a comprehensive evaluation in patients with JIA who hand involvement. We found that functional problems were the most critical problems reported by patients. Factors affecting functional abilities can be evaluated systematically through the POA by choosing the eligible assessment scale. Also, setting a short and long-term functional ability-oriented goal with POA will be provided with practical and systematic strategies for JIA treatment.

**Patient Consent Received**

Yes

**Disclosure of Interest**

None declared

### P2 several rare autoimmune diseases in one patient

#### L. Augunaite, M. Jakineviciute, A. Snipaitiene, L. Jankauskaite

##### Academy of Medicine, Lithuanian University of Health Sciences, Kaunas, Lithuania

###### **Correspondence:** L. Augunaite

**Introduction:** Patients diagnosed with an autoimmune disease have a substantially increased risk for another autoimmune disease, e.g, psoriasis patients can develop multiple sclerosis [1].

**Objectives:** We present a patient with several rare autoimmune diseases.

**Methods:** Case presentation.

**Results:** A 15 y/o girl referred to a pediatric rheumatologist regarding pain of the left knee, increasingly prominent limping and clumsiness. The left wrist movements were painful and limited, and the left knee was slightly swollen, painful during flexion. There were a severe skin psoriasis seen in the head, that was bothering patient already a few years. Also granuloma annulare signs were seen in the armpits and on the ankle area. Ultrasound examination (UG) of the joints showed an effusion in the left knee and signs of a ganglion-specific cyst in the left wrist joint with synovitis signs. Psoriatic juvenile idiopathic arthritis (psJIA) was suspected. Medical anamnesis revealed that the patient was diagnosed with multiple sclerosis (MS) a year ago and received treatment with methylprednisolone pulse therapy. At that time additional studies for infectious and autoimmune disease were done. There were signs of previous citomegalovirus (CMV), Epstein – Barr virus (EBV) and Herpes Simplex virus (HSV) infections (positive IgG for CMV, HSV and EBV). Immunological tests (anti-dsDNA, ANCA, ANA) were in normal range. However, because of the relapsing-remitting course of the neurological disease immunomodulatory treatment with interferon β-1α subcutaneously was initiated. Unfortunately, treatment had to be changed regarding adverse reactions. As MS progressed with new symptoms, new foci in brain MRI and poor prognosis criteria were met, the patient was prescribed second-line treatment with Fingolimoda. During treatment with Fingolimoda, the patient's neurological status remains stable for 2 years. Although there is constant slight leukopenia and lymphopenia (LY: 0,9 x 10 ^9/l, norm: 1,2 -3,34 x 10 ^9/l) seen in complete blood count (CBC). Also episodic exacerbations of psoriasis, subsequent pain of joints and signs of inflammation in the left wrist are observed every 2-3 months. Episodic treatment with NSAIDs for 2-3 weeks for psJIA improves the patient's condition. Additionally, for the left wrist severe inflammation Kenalog injection was done according to the oligoarticular course of psJIA. For now treatment with disease modifying antirheumatic drugs (DMARDs) like methotrexate or others was resisted regarding lymphopenia constantly seen in the CBC.

**Conclusion:** We present a complex patient with several rare autoimmune diseases from different organ systems (skin, joints and neurology) and remind specialists that in the presence of one autoimmune disease, other autoimmune diseases are also possible. In children psoriasis may be associated with multiple sclerosis and progress to psJIA. There are data that the development of different autoimmune diseases can be caused by certain viral infections, which in our case could be the EBV and CMV [2]. Treatment of these patients can be challenging as there is no experience with drug-drug interactions between Fingolimoda and DMARDs.

References

1. Egeberg A, Mallbris L, Gislason GH, Skov L, Hansen PR. Risk of Multiple Sclerosis in Patients with Psoriasis: A Danish Nationwide Cohort Study. J Invest Dermatol. 2016 Jan;136(1):93-8. doi: 10.1038/JID.2015.350. PMID: 26763428.

2. Langer-Gould A, Wu J, Lucas R, Smith J, Gonzales E, Amezcua L, Haraszti S, Chen LH, Quach H, James JA, Barcellos LF, Xiang AH. Epstein-Barr virus, cytomegalovirus, and multiple sclerosis susceptibility: A multiethnic study. Neurology. 2017 Sep 26;89(13):1330-1337. doi: 10.1212/WNL.0000000000004412. Epub 2017 Aug 30. PMID: 28855411; PMCID: PMC5649756.

**Patient Consent Received**

Yes

**Disclosure of Interest**

None declared

### P3 Do we all score the physician global assessment in the same way? Results from a large international survey

#### C. Trincianti^1^, M. Backström^2,3^, M. Tarkiainen^4^, N. Ruperto^5^, A. Consolaro^1,5^, P. Vähäsalo^3,6^

##### ^1^University of Genova, Genova, Italy; ^2^LAVA, Vaasa Central Hospital, Vaasa; ^3^Medical Research Center Oulu, PEDEGO Research Unit, University of Oulu, Oulu; ^4^New Children’s Hospital, Helsinki University Central Hospital and University of Helsinki, Helsinki, Finland; ^5^Pediatric Rheumatology Unit, IRCCS Istituto G.Gaslini, Genova, Italy; ^6^Department of Children and Adolescents, Oulu University Hospital, Oulu, Finland

###### **Correspondence:** M. Backström

**Introduction:** The physician global assessment (PGA) of the level of disease activity plays a central role in monitoring the disease course of juvenile idiopathic arthritis (JIA) patients and the response to therapy in clinical practice and in clinical trials as endorsed by the FDA and EMA for the so called ACR JIA coreset. However, it has not been investigated which factors influence the physicians when scoring the PGA.

**Objectives:** Aim of the study is to assess on a global scale the heterogeneity in PGA scoring and to clarify the factors having an impact on the PGA through a web-based survey.

**Methods:** A questionnaire regarding factors affecting PGA was sent electronically to all 2640 PRINTO members. The responders were asked to rate from 0 to 100 the relevance of 17 factors possibly affecting PGA scoring. The factors were chosen based on consensus of the study panel. The questionnaire also included 17 detailed patient cases selected to represent a diverse spectrum of clinical situations. The responders were asked to indicate the PGA for each patient on a 0 to 100 scale. Results were compared also after grouping for the level of experience of the assessor in the field of pediatric rheumatology (<5 years, 5-10 years, or >10 years). The heterogeneity in the PGA scoring in the 17 cases was measured through the coefficient of variation (CV). The difference between groups was analysed by Kruskal-Wallis one-way analysis of variance.

**Results:** Of the 708 health care providers who responded to the survey, 431 delivered a complete answer regarding the factors affecting PGA and 376 indicated the PGA for all the patients. There was a large individual variation in the impact of different factors on PGA (Table). The smallest variation was seen in the number of swollen joints and tender joints. PGA was scored heterogeneously in most patient cases. The median PGA of the cases did not differ between physicians divided in groups based on experience in pediatric rheumatology. The CV of PGA was >50 in 12 and >100 in 3 out of 17 patient cases. To the question, “If a patient with oligoarticular non-systemic JIA and a polyarticular patient with non-systemic JIA had the same clinical picture would your VAS be different?” 209 physicians replied “no” and 219 “yes”.

**Conclusion:** The PGA is scored heterogeneously throughout the world. Shared guidelines for scoring the PGA are needed to obtain consistent patient assessment in clinical trials and routine practice. In particular the weight of the patient clinical history (e.g. oligoarthritis versus polyarthritis), the presence of extra-articular manifestations and patient reported outcome measures should be discussed.

**Patient Consent Received**

No

**Disclosure of Interest**

None declared

**Table 1 (abstract P3).** Factors affecting physician´s global assessment (mean, standard deviation (SD) and coefficient of variation (CV)) in non-systemic JIA. N = 431

Factors	Mean (SD)	CV	Factors	Mean (SD)	CV
Number of swollen joints	86.1 (18.9)	22.0	CHAQ	54.8 (32.0)	58.3
Number of tender joints	75.4 (24.9)	33.0	Parent/patient pain VAS	50.7 (31.8)	62.7
Duration of morning stiffness	64.4(28.8)	44.7	Proportion of large vs small active joints	47.8 (31.2)	65.4
Presence of active uveitis	67.6 (31.5)	46.6	Psoriatic skin manifestations	49.4 (32.3)	65.4
Dactylitis	63.4 (29.9)	47.2	Parent/patient global VAS	48.4 (31.9)	65.9
Number of restricted joints	63.4 (30.7)	48.4	Presence of erosions	53.2 (35.8)	67.2
Laboratory findings	60.2 (29.0)	49.8	Presence of fever	49.5 (36.9)	75.5
Degree of inflammation in the active joints valued by ultrasound	60.1 (32.2)	53.6	Other factors	12.0 (28.6)	238.3
Degree of inflammation in the active joints valued by MRI	59.6 (33.6)	56.3			

### P4 The juvenile arthritis damage index: how well does this index reflect damage in patients with juvenile idiopathic arthritis?

#### R. Bax, J. van Straalen, N. M. Wulffraat, S. de Roock, J. F. Swart

##### UMC Utrecht, Utrecht, Netherlands

###### **Correspondence:** R. Bax

**Introduction:** The Juvenile Arthritis Damage Index (JADI) is used to assess articular and extraarticular damage caused by long-term inflammation in patients with Juvenile Idiopathic Arthritis (JIA)^1^. Virtually no research has been conducted about the quality of the JADI and the knowledge the index provides to us.

**Objectives:** The aim of this study is to gain insight into the characteristics of JIA patients who scored positively on the articular part of the JADI (JADI-A) and to examine whether the JADI-A is a representative index for damage.

**Methods:** This is a retrospective cohort study in which data were analyzed from patients with JIA in the Wilhelmina Children’s Hospital in Utrecht, the Netherlands. The JADI-A score is expected to increase or remain the same but not to decrease over time. To evaluate this expectation, it was examined if ever-positive JADI-A scores have flipped into a zero on their last reported cumulative JADI-A score.

To evaluate if the JADI-A identified all joints with damage, the results of the JADI-A in the included patients were compared to the limited joint count in the last joint assessment and to radiographic damage. On the other hand, the number of inactive limited joints during the last joint assessment of patients with a never-positive JADI-A was determined to examine if there are patients with inactive limited joints and a negative JADI-A score, whereas they could be expected to have a positive JADI-A score. Finally, the results of the JADI-A of a selection of patients known to have radiographic damage were evaluated to examine the assumption that the JADI-A reflects (radiographic) damage when present.

**Results:** 375 of the 914 patients (41.0%) in the database had an ever-completed JADI-A. Of these 375 patients 53 (14.1%) had an ever-positive JADI-A with a total amount of 158 ever-positive joints. 14 of these 53 patients (26.4%) had a negative JADI-A at the last time the JADI-A was assessed.

Only 29.7% of the ever-positive joints was also limited in the last joint assessment and 69.2% of all ever-positive joints did show radiographic damage on the last X-ray.

14.6% of the 322 patients with a never-positive JADI-A did have inactive limited joint(s) in their last joint assessment. Only 50.0% of the 18 selected patients known to have radiographic damage and with an ever-completed JADI-A had an ever-positive JADI-A.

**Conclusion:** A considerable discrepancy was seen between the results of the JADI-A on the one hand and the inactive limited joint count in the last joint assessment as well as radiographic damage on the other hand. Furthermore several positive JADI-A scores flipped to negative, suggesting either that rheumatologists did not complete the index correctly and/or that the JADI-A is not successful in detecting (lasting) damage in patients with JIA. Further research on this remarkable discrepancy is needed, because in the end we want to identify all children with damage as expeditiously as possible and to not unnecessarily burden children without joint damage with repeated radiographs.

**Disclosure of Interest**

None declared

### P5 Prospective study on MMR booster vaccine in children with rheumatic diseases treated with DMARDS and/or biologics

#### M. Bizjak, F. Dasoula^2^, B. Balaziova^3^, A. Adrovic^4^, D. Maritsi^2^, T. Dallos^3^, O. Kasapcopur^4^, N. Toplak^1,5^, on behalf of PReS Vaccination WP

##### ^1^Department of Allergology, Rheumatology and Clinical Immunology, UCH Ljubljana, Ljubljana, Slovenia; ^2^Second Department of Pediatrics, “P.&A. Kyriakou” Children’s Hospital, National and Kapodestrian University of Athens, Athens, Greece; ^3^Department of Paediatrics, Comenius University Medical School in Bratislava, National Institute of Children’s Diseases, Bratislava, Slovakia; ^4^Department of Pediatric Rheumatology, Istanbul University-Cerrahpasa, Istanbul, Turkey; ^5^MF Ljubljana, Ljubljana, Slovenia

###### **Correspondence:** M. Bizjak

**Introduction:** Live attenuated vaccines are not usually recommended in children with rheumatic diseases (RD) treated with immunosuppressive (IS) therapy (1). However, a retrospective study which included 234 children with RD who received live attenuated booster measles-mumps-rubella (MMR) vaccine during treatment with IS therapy showed that the booster dose was safe (2).

**Objectives:** To evaluate safety and long-term immunogenicity of the MMR booster vaccine in children with RD treated with IS therapy in a prospective study.

**Methods:** This is an ongoing multinational, multicentre prospective study. Patients with immune-mediated diseases treated with DMARDs and/or biologic therapy with stable disease were included if they were scheduled, according to their national vaccination program, to receive 2^nd^ (booster or “catch-up”) dose of MMR vaccine. Safety was monitored by tracking infection with vaccine or wild-type viruses after vaccination, possible adverse events of the MMR vaccine and by monitoring disease activity before and after vaccination. Immunogenicity was monitored by measuring protective antibodies before vaccination and then at predetermined time points after vaccination.

**Results:** By the end of May 2021, 22 patients from 4 centres were included (Greece, Slovakia, Slovenia and Turkey). One patient had localized scleroderma and all others had juvenile idiopathic arthritis (JIA) (12 oligoarticular JIA, 4 polyarticular JIA, 2 systemic JIA, 2 enthesitis-related arthritis, 1 psoriatic JIA) Median age at diagnosis was 3.8 years (range 1.4-10.5 years), median age at 1^st^ dose of MMR vaccine was 1.27 years (range 0.58-4.36 years) and median age at 2^nd^ dose of MMR vaccine was 8.05 years (range 2.8-14.3 years). At the time of 2^nd^ vaccination, 13 patients were treated with TNF-α inhibitors, 9 of them received methotrexate (MTX) concomitantly, 1 patient was on IL-1 inhibitor and corticosteroids (CS), 1 patient on IL-6 inhibitor and CS and 7 patients were on MTX. Regarding safety, there were no increase in disease activity, vaccine strain infection or serious adverse events after vaccination. Six patients reported mild local or systemic reactions (fever, fatigue, arthralgia, headache, cough) in the 6 weeks following vaccination. Protective antibodies against measles and mumps were measured in 9 patients, they were positive in 7/8 patients 2-3 months after 2^nd^ dose.

**Conclusion:** These preliminary results of prospective study corroborate the findings of recently published retrospective study (2). However, the number of included children is currently too small to draw any firm conclusions and long-term immunogenicity remains to be determined in the future. This is an ongoing project and we expect that other countries and centres will join this effort soon.

**References**

1 Heijstek MW, et al. Ann Rheum Dis. 2011; 70: 1704-12.

2 Uziel Y, et al. Vaccine. 2020; 38: 2198-201.

**Patient Consent Received**

Yes

**Disclosure of Interest**

None declared

### P6 M-ficolin: a valuable biomarker to identify leukemia from juvenile idiopathic arthritis

#### N. Brix^1,2^, M. Glerup^1^, S. Thiel^3^, C. E. Mistegaard^3^, R. G. Skals^4^, L. Berntson^5^, A. Fasth^6^, S. Nielsen^7^, E. Nordal^8^, M. Rygg^9,10^, H. Hasle^1^, B. K. Albertsen^1^, T. Herlin^1^, on behalf of the Nordic Study Group of Pediatric Rheumatology (NoSPeR) group

##### ^1^Department of Pediatrics and Adolescent Medicine, Aarhus University Hospital, Aarhus; ^2^Department of Pediatrics and Adolescent Medicine, Aalborg University Hospital, Aalborg; ^3^Department of Biomedicine, Aarhus University Hospital, Aarhus; ^4^Department of Clinical Biostatistics, Aalborg University Hospital, Aalborg, Denmark; ^5^Department of Women’s and Children’s Health, Uppsala University, Uppsala University; ^6^Department of Pediatrics, Institute of Clinical Sciences, Sahlgrenska Academy, University of Gothenburg, Gothenburg, Sweden; ^7^Department of Pediatrics, Rigshospitalet, Copenhagen University Hospital, Copenhagen, Denmark; ^8^Department of Pediatrics, University Hospital of North Norway and Department of Clinical Medicine, UiT The Arctic University of Norway, Tromsø; ^9^Department of Clinical and Molecular Medicine, NTNU - Norwegian University of Science and Technology; ^10^Department of Pediatrics, St. Olavs Hospital, Trondheim, Norway

###### **Correspondence:** N. Brix

**Introduction:** Distinction on clinical grounds between acute lymphoblastic leukemia presenting with arthropathy (ALL_arthropathy_) and juvenile idiopathic arthritis (JIA) is difficult, as the clinical and paraclinical signs of leukemia may be vague. As the only study of lectin pathway in children with ALL indicate low serum M-ficolin levels and M-ficolin has proven to be a marker of disease activity in JIA, we hypothesized that it would be possible to differentiate ALL from JIA by M-ficolin.

**Objectives:** The primary aim was to examine the use of lectin complement pathway proteins as markers to differentiate ALL_arthropathy_from JIA. The secondary aims were to compare the protein levels at baseline and follow-up in a paired number of children with ALL and to examine the correlation with hematology counts, ESR, CRP, blasts, relapse, and death.

**Methods:** In this observational study, we measured M-ficolin, CL-K1 and MASP-3 in children with ALL (n = 151) and JIA (n = 238) by time-resolved immunofluorometric assays in serum. Logistic regression was used for the predictions, considering risk of ALL as the outcome. We performed internal validation using repeated “10-fold cross-validation” with 100 repetitions computing the optimized corrected Area Under the ROC curve (AUC) as well as positive and negative predictive values in order to evaluate the predictive performance.

**Results:** The level of M-ficolin was more than 4-fold higher in JIA than in ALL_total_ and the ALL_arthropathy_ subgroup_:_ 0.65 μg/mL (IQR 0.32-1.21) versus 3.01 μg/mL (IQR 2.43-3.85)μg/mL, p <0.001. The M-ficolin level normalized after remission of ALL to 1.57 μg/mL ( IQR 0.83-2.24), p <0.001. M-ficolin could differentiate ALL from JIA with an AUC of 94% and positive predictive value (PPV) of 95%, exceeding CRP and hemoglobin. In a dichotomy predictive model with optimal cut-offs for M-ficolin, platelets and hemoglobin AUC was 99% and PPV 98% in detecting ALL from JIA.

**Conclusion:** M-ficolin is a valuable marker to differentiate the child with ALL from JIA.

**Patient Consent Received**

Yes

**Disclosure of Interest**

None declared

### P7 Nationwide Israeli study: obstacles in early diagnosis of children with juvenile idiopathic arthritis (JIA) - a retrospective study

#### Y. Butbul Aviel, Y. Frenkel^2^, I. Kraushar^3^, M. H. Saied^4^, R. Haviv^5^, Y. Uziell^5^, Y. Berkun^6^, M. Heshin-Bekenstein^7^, I. Tirosh^8^, I. Tirosh^9^, G. Amerilio^10^, L. Harel^11^, E. Ling^12^

##### ^1^Pediatric Rheumatology unit, Ruth Rappaport Children's Hospital, Rambam Medical Center, Haifa, Israel; ^2^Rambam Medical Center Haifa Israel, Haifa, Israel; ^3^Pediatric Rheumatology Service, Rambam Medical Center Haifa Israel; ^4^Pediatric Rheumatology Service, Carmel Medical center, Haifa; ^5^Pediatric Rheumatology Service, Department of Pediatrics, Meir Medical Center, Kfar Saba; ^6^Pediatric Rheumatology Service, Hadassah-Hebrew University Medical Center, Mount Scopus, Jerusalem, Jerusalem; ^7^Pediatric Rheumatology Service, Dana-Dwek Children’s Hospital Tel Aviv; ^8^Pediatric Rheumatology Service, Edmond and Lily Safra Children's Hospital, Sheba Medical Center; ^9^Israel Pediatric Rheumatology service, Edmond and Lily Safra Children's Hospital, Sheba Medical Center, Tel-Hashomer, Tel Aviv; ^10^Pediatric Rheumatology Unit, Schneider Children's Medical Center of Israel; ^11^Pediatric Rheumatology Unit, Schneider Children's Medical Center of Israel, Petach Tikva; ^12^Pediatric Rheumatology Unit, Saban Pediatric Medical Center for Israel Soroka University Medical Center, Be'er Sheva, Israel

###### **Correspondence:** Y. Butbul Aviel

**Introduction:** Juvenile idiopathic arthritis (JIA) is the leading cause of chronic arthritis in children. A delay in the diagnosis may lead to long term damage.

**Objectives:** Characterization of the stages that patients with JIA pass until diagnosis and analysis of the different outcome measures that lead to a delay in the diagnosis of JIA in Israel.

**Methods:** We conduct a retrospective cohort study in 8 pediatric rheumatology centers in Israel. All patients that were diagnosed with JIA between 2017 and 2019 included in the study. Demographic, clinical and data regarding the referral’s doctors were collected.

**Results:** 207 patients included in the study (69% female). Patients were evaluated by primary care physicians (62%), ER physicians (13%), and orthopedists (11%) prior to diagnosis. The median time until diagnosis was 56 days (1-2451 days). Patients diagnosed with ERA/SPA and psoriatic arthritis had the longest period until diagnosis (351 and 213 days respectively). A younger age was correlated with a later diagnosis (r=0.3, P<0.0001). Females were diagnosed earlier compared to males (median 48 vs 84 days respectively). The distance to the rheumatology center with regards to time until diagnosis was not significant (P=0.19). Fever at presentation, significantly shortened the time to diagnosis (P<0.0001), whereas involvement of the small joints/sacroiliac joints significantly lengthened the time (P<0.05).

**Conclusion:** This is the first nationwide multicenter study that analyzes the obstacles in the diagnosis of JIA in Israel. Raising awareness of JIA, especially of patients with SPA, is crucial in order to avoid delays in diagnosis and treatment.

**Patient Consent Received**

No

**Disclosure of Interest**

None declared

### P8 Juvenile idiopathic arthritis: assessment of the general knowledge of the different medical professionals involved

#### L. Charlène, D. Alexandra

##### Calvados, CHU de Caen, Caen, France

###### **Correspondence:** L. Charlène

**Introduction:** Juvenile idiopathic arthritis is one of the most common chronic pediatric diseases. However, it remains little known by 1st line doctors to evoke the diagnosis. This results in a diagnostic delay, sometimes several months, potentially responsible for disabilities. The associated anterior uveitis is also unknown. This is probably related to a lack of teaching, but studies on this subject are scares.

**Objectives:** The main objective is to establish an inventory of the knowledge of medical professionals of Lower Normandy, but also to sensitize them to this pathology, to make better known its network of care, and its management.

**Methods:** We carried out a cross-sectional descriptive study whose data were collected using a questionnaire addressed to the various medical professionals concerned by the AJI in the three departments of the former Basse-Normandie region: pediatricians, rheumatologists, pediatric orthopaedic surgeons, ophthalmologists, general practitioners and internal physicians of these different specialties. Three hundred and eleven questionnaires were collected out of the 178 sent out.

**Results:** The response rate was 17.4%. Juvenile idiopathic arthritis is known to 89% of professionals. Dedicated rare disease reference and skills centers are known by 31% and 32% of them; the national diagnostic and care protocol (PNDS) by 20.5%. Amalgamation with inflammatory pathologies of the digestive tract (10%) and autoimmune pathologies (9%) is common. The anti-nuclear factors are performed by 19% of professionals. Systemic corticosteroids are prescribed in 23% of cases in 1st intention. Anterior uveitis is recognized in 76% of cases as a complication (ophthalmology). Quarterly eye monitoring is carried out by 35% of professionals, 45% of whom are ophthalmologists.

**Conclusion:** Our results show that, although the majority of health professionals who responded to this questionnaire are aware of the AJI, there is a lack of education during initial training of physicians. Specific training seems necessary in order to improve the management of this chronic pathology and thus avoid sequelae. These data must be confirmed by other studies of national scope in order to determine whether the lack of knowledge about this pathology is related to a lack of communication and local or national education.

**Patient Consent Received**

Yes

**Disclosure of Interest**

None declared

### P9 Vitamin D levels and risk of juvenile idiopathic arthritis: a mendelian randomization study

#### S. L. Clarke^1,2,3^, R. E. Mitchell^1,2^, G. C. Sharp^1,2^, A. V. Ramanan^3,4^, C. L. Relton^1,2^

##### ^1^MRC Integrative Epidemiology Unit; ^2^Population Health Sciences, University of Bristol; ^3^Department of Paediatric Rheumatology, Bristol Royal Hospital for Children; ^4^Translational Health Sciences, University of Bristol, Bristol, United Kingdom

###### **Correspondence:** S. L. Clarke

**Introduction:** Evidence for the role of vitamin D in juvenile idiopathic arthritis (JIA) risk is mixed across epidemiological studies, however such studies are challenging to undertake and are susceptible to considerable bias. Low vitamin D levels are common within the general population and easily corrected, therefore there is considerable potential public health benefit if a causal association between vitamin insufficiency and JIA is established. Mendelian randomisation (MR) is a method of causal inference which relies on the use of genetic variation, assigned at conception, as a proxy of the exposure of interest and thus limits bias due to confounding and reverse causation, subject to core assumptions being met.

**Objectives:** To use MR to test whether vitamin D levels are causally associated with JIA risk.

**Methods:** Since 25-hydroxy-vitamin D (25OHD) is the major circulating form of vitamin D we used summary level data from the largest and most recent genome wide association study of 25OHD levels (sample size 443,734), alongside summary data from two JIA genome wide association studies (sample sizes 15,872 and 12,501), all from European populations. To test and account for potential bias due to violations of the core MR assumptions we employed multiple different MR methods and sensitivity analyses.

**Results:** Using a genetic instrument for 25OHD, comprising 69 25OHD-associated single nucleotide polymorphisms, we show there is no evidence of a causal association between genetically predicted 25OHD levels and JIA risk (OR 1.00, 95% CI 0.76-1.33 per standard deviation increase in standardised natural-log transformed 25OHD levels). This estimate was consistent across all methods tested. There was also no evidence of reverse causation when assessing the causal effect of genetically predicted JIA on 25OHD levels (0.004 standard deviation decrease in standardised natural-log transformed 25OHD levels per doubling odds in genetically predicted JIA, 95% CI -0.009-0.002).

**Conclusion:** In this study we found no evidence that genetically increased 25OHD levels confers protection from JIA and that population level vitamin D supplementation is unlikely to contribute to a reduction in JIA incidence.

**Patient Consent Received**

No

**Disclosure of Interest**

None declared

### P10 Achievement of controlled disease and normal physical function in patients with polyarticular course juvenile idiopathic arthritis receiving tofacitinib: a post hoc analysis of data from a phase 3, randomised, withdrawal trial

#### A. Consolaro^1^, D. J. Lovell^2^, O. Synoverska^1^, C. Abud Mendoza^1^, A. Spindler^1^, Y. Vyzhga^1^, E. Alexeeva^1^, J. Chaitow^1^, P. Chiraseveenupraprund^2^, H. Shi^3^, L. Stockert^3^, G. Sawyerr^4^, A. Diehl^3^, N. Ruperto^1^, H. I. Brunner^2^, on behalf of for PRINTO and PRCSG

##### ^1^PRINTO, IRCCS Istituto Giannina Gaslini, Genova, Italy; ^2^PRCSG, Cincinnati Children’s Hospital Medical Center, Cincinnati, OH; ^3^Pfizer Inc, Collegeville, PA; ^4^Pfizer Inc, New York, NY, United States

###### **Correspondence:** A. Consolaro

**Introduction:** Tofacitinib is an oral JAK inhibitor that is being investigated for several forms of JIA. The efficacy and safety of tofacitinib in patients (pts) with polyarticular course (pc)JIA were demonstrated in a Phase 3 trial.

**Objectives:** To evaluate the frequency of controlled disease, measured by achievement of low disease activity (LDA) status per the Juvenile Arthritis Disease Activity Score (JADAS), and normal physical function, measured by the Childhood Health Assessment Questionnaire-Disability Index (CHAQ-DI), in tofacitinib-treated pts with pcJIA.

**Methods:** Data were analysed post hoc from pts with pcJIA aged 2−<18 years in a Phase 3, randomised, double-blind, placebo (PBO)-controlled withdrawal trial, where all pts received open-label tofacitinib 5 mg BID or body weight-based lower equivalent dose until Week (W)18 (end of Part 1). Pts with ≥JIA/ACR30 response at W18 were randomised 1:1 to continue tofacitinib or switch to PBO in the double-blind phase (Part 2, W18−44). Pts with JIA flare were discontinued from the trial. pcJIA disease control was assessed using: clinical JADAS in 10 joints (cJADAS10; no CRP/ESR laboratory measure), JADAS10-CRP and CHAQ-DI. LDA for cJADAS10 was defined as a score of ≤2.5 and for JADAS10-CRP as a score of ≤3.8. Normal function was defined as a CHAQ-DI score of 0.

**Results:** 184 pts with pcJIA entered Part 1 and 142 were randomised in Part 2 to continue tofacitinib (N=72) or switch to PBO (N=70). At Part 1 baseline, pcJIA control was absent, per mean (SD) values of: cJADAS10, 18.9 (4.7); JADAS10-CRP, 20.3 (5.5); and CHAQ-DI, 1.0 (0.7). At W18, disease control was improved, per mean (SD) values of: cJADAS10, 5.4 (4.8); JADAS10-CRP, 6.2 (4.9); and CHAQ-DI, 0.5 (0.6). cJADAS10 LDA and JADAS10-CRP LDA were achieved at the end of Part 1 by 42.9% and 44.2% of pts (N=154), respectively, while 24.0% achieved cJADAS10 LDA + normal function and 24.0% achieved JADAS10-CRP LDA + normal function. In Part 2, LDA ± normal function frequency remained stable in pts who continued tofacitinib, while in pts who switched to PBO, LDA ± normal function frequency decreased over time (Table). At the end of Part 2 (W44), in pts who continued tofacitinib vs pts who switched to PBO, cJADAS10 LDA was achieved in 47.2% vs 31.4% and JADAS10-CRP LDA in 47.2% vs 32.9%, respectively.

Pts who discontinued treatment for any reason, except while in clinical remission, were counted as non-responders as of their discontinuation visit through W44

**Conclusion:** Tofacitinib reduced disease activity in a rapid and sustained fashion and improved function in pts with pcJIA. A large proportion of pts achieved LDA, a current pcJIA treatment target, at W18 and thereafter. LDA prevalence estimates were comparable using cJADAS10 vs JADAS10-CRP, suggesting that cJADAS10 may suffice to assess treatment targets in pts with pcJIA.

**Trial registration identifying number:** ClinicalTrials.gov (NCT02592434)

**Patient Consent Received**

No

**Disclosure of Interest**

A. Consolaro: None declared, D. Lovell Consultant for: AstraZeneca, Boehringer Ingelheim, GSK, Roche, Novartis, Pfizer Inc, Takeda and UBC, and DSMB chairperson for NIH, O. Synoverska Speaker Bureau of: Sanofi, C. Abud Mendoza: None declared, A. Spindler Speaker Bureau of: Eli Lilly, Y. Vyzhga: None declared, E. Alexeeva: None declared, J. Chaitow: None declared, P. Chiraseveenupraprund Consultant for: CARRA and Novartis, H. Shi Shareholder of: Pfizer Inc, Employee of: Pfizer Inc, L. Stockert Shareholder of: Pfizer Inc, Employee of: Pfizer Inc, G. Sawyerr Consultant for: Pfizer Inc, Employee of: Syneos Health Inc, A. Diehl Shareholder of: Pfizer Inc, Employee of: Pfizer Inc, N. Ruperto Consultant for: Ablynx, AstraZeneca/MedImmune, Biogen, BMS, Boehringer Ingelheim, Eli Lilly, EMD Serono, F. Hoffmann-La Roche, GSK, Janssen, Merck Sharp & Dohme, Novartis, Pfizer Inc, R-Pharm, Sanofi, Servier, Sinergie and Sobi, Speaker Bureau of: Ablynx, AstraZeneca/MedImmune, Biogen, BMS, Boehringer Ingelheim, Eli Lilly, EMD Serono, F. Hoffmann-La Roche, GSK, Janssen, Merck Sharp & Dohme, Novartis, Pfizer Inc, R-Pharm, Sanofi, Servier, Sinergie and Sobi, H. Brunner Consultant for: AbbVie, AstraZeneca/MedImmune, Bayer, Biocon, BMS, Boehringer Ingelheim, Eli Lilly, Janssen, Novartis, Pfizer Inc, Roche and R Pharm, Employee of: Cincinnati Children’s Hospital Medical Center, Speaker Bureau of: GSK, Novartis and Roche

**Table 1 (abstract P10).** Number (%) of pts with pcJIA achieving JADAS LDA ± normal function with tofacitinib vs PBO in Part 2

	Tofacitinib N=72	PBO N=70	p value	Tofacitinib N=72	PBO N=70	p value
	**cJADAS10 LDA**			**JADAS10-CRP LDA**		
W18	34 (47.2)	32 (45.7)	0.857	35 (48.6)	33 (47.1)	0.861
W24	32 (44.4)	21 (30.0)	0.072	34 (47.2)	24 (34.3)	0.114
W44	34 (47.2)	22 (31.4)	0.051	34 (47.2)	23 (32.9)	0.077
	**cJADAS10 LDA + normal function**			**JADAS10-CRP LDA + normal function**		
W18	17 (23.6)	20 (28.6)	0.501	17 (23.6)	20 (28.6)	0.501
W24	23 (32.0)	14 (20.0)	0.101	24 (33.3)	15 (21.4)	0.108
W44	26 (36.1)	13 (18.6)	0.017	25 (34.7)	14 (20.0)	0.046

### P11 Usefulness of synovial biopsy in the differential diagnosis and as predictor of clinical course in juvenile idiopathic arthritis: a monocentric study on 100 patients

#### S. Costi^1^, F. Pregnolato^1^, A. Parafioriti^2^, E. Armiraglio^2^, T. Giani^3^, R. Cimaz^4,5^

##### ^1^Pediatric Rheumatology, University of Milan, G. Pini Hospital; ^2^Department of Pathology, G. Pini Hospital, Milan; ^3^Department of Medical Biotechnology, University of Siena, Siena; ^4^Department of Clinical Sciences and Community Health, and Research Center for Adult and Pediatric Rheumatic Diseases, University of Milan; ^5^Pediatric Rheumatology, G. Pini Hospital, Milan, Italy

###### **Correspondence:** S. Costi

**Introduction:** While synovial biopsy is an invasive procedure and is not required for the diagnosis of juvenile idiopathic arthritis (JIA), it may be useful in doubtful cases. Histopathologic characterization with regard to future course of disease has not been well studied.

**Objectives:** Aims of this study were i. to investigate the usefulness of synovial biopsy for diagnostic purposes, and ii. to review histological specimens in order to evaluate possible associations between pathology features and JIA clinical outcome.

**Methods:** We reviewed data from medical records of patients under the age of 18 years who underwent a synovial biopsy requested by our Pediatric Rheumatology Unit over the last 15 years. We collected information on clinical (remission criteria, number of disease flares, number of cDMARDs/bDMARDS used) and histological characteristic (number of layers of synovial lining, inflammatory infiltrate, elementary lesions of the subsynovia). Differences in numerical variable between groups were assessed by Mann-Whitney test while associations between categorical predictors and outcomes by Chi-square or Fisher’s exact test. A logistic multivariable model with outcome as dependent variable was applied in order to measure the strength of and adjust for potential confounders.

**Results:** We identified 100 patients who underwent a synovial biopsy during the study period. Of those, 99 (65% female) had complete data. Median age at onset of 8.5 years (range 1-17), median follow-up time was 161 months (range 8-1160). We recognized two groups: patients with known/suspected JIA (44/99) and patients with unknown diagnosis (55/99). In the first group, 10 patients underwent synovial biopsy as a result as diagnostic doubt, while 34 had an orthopedic procedure. In all these 34 patients the biopsy results were consistent with JIA. Among the second group, 31/55 results were consistent with a chronic synovitis (final JIA diagnosis), while in others the histologic results led to a final diagnosis of other conditions (e.g. Giant cell tumor of the synovium and tendon sheat n=6, foreign body n=2, osteomyelitis n=1, sarcoidosis n=1). Between the two groups we identified 75 patients with JIA diagnosis. At the last follow-up visit 47 of them of patients were in clinical remission or had low disease activity, while 23 patients had a severe course of disease. In 43 cases the correlation between status at clinical outcome and histological score could be assessed. Subjects who had more than 4 flares during follow-up showed a significantly higher number of layers of synovial lining (4.5 [3.0 to 6.0] vs 3.0 [2.0 to 4.5], p = 0.035). The number of layers remained significantly predictive after adjusting for age at diagnosis and observation time (OR [95% CI]: 2.2 [1.3 - 3.9], p = 0.007). Subjects who had switched more than two bDMARDs had a higher prevalence of elementary lesions of the subsynovia (55.6% vs 10.3%, p = 0.005) and a higher prevalence of fibrin deposits at the level of the synovial lining (60.0% vs 22.6%, p = 0.04). Fibrin deposits remained significant predictors even after adjustment for observation time and age at diagnosis (OR [95% CI]: 8.1 [1.03 - 64.2, p = 0.047]). Krenn score and infiltrate pathotype were assessed but no statistically significant association was found.

**Conclusion:** Synovial biopsy may be useful in those patients whose diagnosis is unclear and in complicated cases it may allow diagnoses of rare conditions. We found statistically significant correlations between histological features of synovial membrane and the clinical phenotype.

**Patient Consent Received**

No

**Disclosure of Interest**

None declared

### P12 Influenza vaccine uptake among JIA patients in COVID-19 era: a multi-centre cross-sectional study

#### F. Dasoula^1^, D. Maritsi^1^, N. Alpert^2^, M. Bizjak^3^, M. Heshin-Bekenstein^4^, A. Ziv^5^, B. Balaziova^6^, M. Yildiz^7^, A. Gagro^8^, M. Sestan^9^, A. Khabirova^10^, B. Sozeri^11^, S. Caglayan^11^, M. Jelusic^9^, V. Opoka-Winiarska^12^, M. Kostik^10^, C. Bracaglia^13^, F. Minoia^14^, T. Dallos^6^, O. Kasapcopur^7^, N. Toplak^3^, Y. Uziel^2^, on behalf of Vaccination WP & MAS/sJIA WP

##### ^1^Infectious Diseases, Immunology and Rheumatology Unit, Second Dpt of Pediatrics, Athens Medical School, Athens, Greece; ^2^Pediatric Rheumatology Unit, Dpt of Pediatrics, Meir Medical Center, Kfar Saba, Israel; ^3^Dpt. of allergy, rheumatol, immunol UCH Ljubljana. MF Ljubljana, Ljubljana, Slovenia; ^4^Dana Children’s Hospital of Tel Aviv Medical Center; ^5^Sackler School of Medicine, Tel Aviv University, Tel Aviv, Israel; ^6^Dpt of Paediatrics, Comenius University Medical School in Bratislava, National Institute of Children’s Diseases, Bratislava, Slovakia; ^7^Dpt of Pediatric Rheumatology, Istanbul University, Cerrahpasa, Turkey; ^8^Dpt of Pediatrics, Children's Hospital Zagreb; ^9^Dpt of Paediatrics, University of Zagreb School of Medicine, Zagreb, Croatia; ^10^Saint-Petersburg State Pediatric Medical University, Saint-Petersburg, Russian Federation; ^11^University of health sciences, Umraniye Training and Research Hospital, Istanbul, Turkey; ^12^Dpt of Paediatric Pulmonology and Rheumatology, Medical University of Lublin, Lublin, Poland; ^13^Division of Rheumatology, IRCCS Ospedale Pediatrico Bambino Gesù, Roma; ^14^Fondazione IRCCS Ca’ Granda Ospedale Maggiore Policlinico, Milan, Italy

###### **Correspondence:** F. Dasoula

**Introduction:** JIA patients are targeted for vaccination as a population at risk of severe influenza disease. Recent COVID-19 pandemic is an additional challenge; nonetheless, their influenza vaccine coverage remains uncertain.

**Objectives:** To assess the flu vaccination rate in JIA patients & investigate family’s attitudes towards it; to identify how COVID-19 pandemic has affected caregivers’ decision on flu vaccine administration.

**Methods:** A multi-center cross-sectional study was conducted across 9 countries. Participants completed a questionnaire about the flu vaccination uptake history including the year of 2020-21, sociodemographics and data regarding the disease. Analysis was conducted using SPSSv.20.

**Results:** 655 JIA caregivers were surveyed across 9 countries (Table). The majority was employed (61.2%), married (78.5%) & held a tertiary education (43.1%). Patients’ median age was 11y (IQR: 7-15). Principal diagnosis was oligoJIA (34%) & most patients were treated systemically (81%). 21.7% had received influenza vaccine in the past & 18.6% in 2019-20 season; 85% were fully vaccinated.

152 children (23.2%) were vaccinated against flu the current season. The majority was informed by their ped rheumatologist (33.5%). Highest uptake was recorded in Greece (79.3%) while lowest in Turkey (1.1%) (p<0.01) (Table). Employed & self-employed and those with tertiary education were more likely to vaccinate their children compared to unemployed & those with elementary education (28.2% & 29.9% vs 13.6%, 28% vs 9.7% respectively, p<0.01). Children with psoriatic & polyJIA had the highest uptake (both 30%) while patients with undifferentiated reported the lowest (7.4%, p<0.01). Among vaccinators, 92.4% were fully vaccinated, 64.6% had been vaccinated in the past & 57.6% in 2019-20 season (p<0.05). An increase was reported in uptake between 2019-20 & 2020-21 seasons (18.6% vs 23.2%, p<0.01).

Among non-vaccinators, 52% did not have the chance to discuss their concerns. Major reason for non-vaccination was unawareness of the need (36%); 13% reported it was a doctor’s advice. Caregivers suggested that informing in advance (67%) may improve uptake.

Most of caregivers expressed their concerns regarding their children’s vulnerability to SARS-COV-2 due to JIA (51.3%) & their risk of COVID-19 (85.3%); 51.3% of them were pro COVID-19 vaccine administration to their children (Table). Those who vaccinated their children against flu in the 2019-20 & 2020-21 seasons were more likely to vaccinate them against SARS-COV-2 (79.5% & 73.4% respectively, p<0.01).

**Conclusion:** Despite variations among countries, flu vaccine uptake remains low in JIA patients. Higher education, thorough informative discussion and notifying families in advance may lead to universal vaccine coverage in children with RDs.

**Disclosure of Interest**

None declared

**Table 1 (abstract P12).** See text for description

Country	Participants N(%)	Flu vaccine uptake in 2019-20 season N(%)	Flu vaccine uptake in 2020-21 season N(%)	Intention of COVID-19 vaccine administration N(%)
Croatia	76(11.6)	20(26.3)	21(27.6)	38(50)
Greece	29(4.5)	23(79.3)	23(79.3)	21(72.4)
Israel	74(11.3)	35(47.3)	37(50)	47(63.5)
Italy	14(2.1)	2(14.3)	6(42.9)	11(78.6)
Poland	48(7.3)	14(29.2)	10(20.8)	36(75)
Russia	66(10.1)	6(9.1)	5(7.4)	11(16.7)
Slovakia	73(11.1)	3(4.1)	24(32.9)	40(54.8)
Slovenia	85(13)	8(9.4)	24(28.2)	29(34.1)
Turkey	190(29)	11(5.8)	2(1.1)	103(54.2)

### P13 Identification of pathogenic B cells in JIA by the oxysterol receptor GPR183

#### N. M. de Gruijter^1,2^, C. Brown^3^, L. R. Wedderburn^1^, E. C. Rosser^1,2^

##### ^1^Centre for Adolescent Rheumatology Versus Arthritis at UCL, UCLH and GOSH; ^2^Centre for Rheumatology Research, University College London, London, United Kingdom; ^3^Memorial Sloan Kettering Cancer Center, New York City, United States

###### **Correspondence:** N. M. de Gruijter

**Introduction:** Juvenile idiopathic arthritis (JIA) is the most common rheumatic disease of childhood and causes significant suffering. Oligoarthritis (oJIA) – the most common JIA subtype – is characterized by autoantibody production and the differentiation of autoreactive T cells. B cells produce these autoantibodies, but also contribute to pathology by secreting cytokines and presenting antigen. Case studies have shown some positive effects of B cell-depleting drugs, but B cell depletion also reduces immunoregulatory B cells. Treatment strategies specifically targeting pathogenic B cells are necessary, especially for patients that do not respond well to conventional treatments.

**Objectives:** To identify a marker for pathogenic memory B cells in JIA synovial fluid (SF) and peripheral blood (PB).

**Methods:** Single-cell RNA sequencing was used to measure gene expression in JIA B cells from SF and PB. Flow cytometry was used for in-depth phenotyping of B cells in the SF and/or PB of 31 patients with oJIA, and the PB of 5 healthy children. Mann-Whitney U test was used to compare significance of difference between groups. All numbers are portrayed as median (interquartile range), unless otherwise specified.

**Results:** Through single cell RNA sequencing we found that memory B cell subsets can be defined by the expression of oxysterol receptor GPR183. When plotting the expression of GPR183 against that of activation marker CD27, we saw a clear population co-expressing both markers. GPR183^+^CD27^+^ memory B cells are found in oJIA synovial fluid (SF), oJIA peripheral blood (PB), and healthy child and adult PB. GPR183^+^CD27^+^ memory B cells are expanded in oJIA SF compared to PB: 23.8% (19.5%-29.5%) of oJIA SF B cells are GPR183^+^CD27^+^, compared 7.2% (4.8%-9.5%) in oJIA PB (*p*<0.0001, *U*=8), and 8.8% (6.5%-11.4%) in healthy child PB (*p*=0.0004*, U*=3.5). GPR183^+^CD27^+^ memory B cells in oJIA SF have an atypical, activated phenotype, expressing high levels of CD86, HLA-DR, CD11c and FcRL4 (Table 1) compared to oJIA PB (expression did not differ between healthy child and oJIA PB; data not shown). Depletion of GPR183^+^ B cells from total oJIA synovial fluid mononuclear cells reduced T cell proliferation *in vitro*. Blocking the production of GPR183’s endogenous ligand 7α,25-dihydroxycholesterol with the anti-fungal drug clotrimazole reduced inflammation in a mouse model of experimental arthritis. These functional experiments suggest that GPR183^+^ memory B cells have a pro-inflammatory role in joint inflammation.

**Conclusion:** Our data identify GPR183 as an important, therapeutically relevant molecule for the definition of memory B cells in juvenile idiopathic arthritis.

Rerences

1. Yeo L, Lom H, Juarez M, Snow M, Buckley CD, Filer A, et al. Expression of FcRL4 defines a pro-inflammatory, RANKL-producing B cell subset in rheumatoid arthritis. Ann Rheum Dis. 2015;74(5):928–35.

**Patient Consent Received**

Yes

**Disclosure of Interest**

None declared

**Table 1 (abstract P13).** Expression of phenotypic markers on oJIA SF and PB B cells

Phenotypic marker	Brief description	Expression on oJIA synovial fluid B cells (n=18) (median, interquartile range)	Expression on oJIA peripheral blood B cells (n=17) (median, interquartile range)	Result of Mann-Whitney U test
CD86	Activation marker involved in antigen presentation	33.4% (21.7%-46.3%) positive expression	10.0% (6.9%-14.2%) positive expression	*p<*0.0001 *U*=3
HLA-DR	Activation marker involved in antigen presentation	4096 (3347-4927) median fluorescent intensity	1874 (1738-2074) median fluorescent intensity	*p<*0.0001 *U*=16
CD11c	Activation marker	59.3% (46.0%-68.9%) positive expression	20.0% (15.1%-25.2%) positive expression	*p<*0.0001 *U*=6
FcRL4	Atypical marker expressed on pro-arthritogenic B cells in rheumatoid arthritis (1)	20.0% (13.7%-46.3%) positive expression	13.4% (10.3%-18.0%) positive expression	*p=*0.0240 *U*=85

### P14 Lubricin concentration of synovial fluid and blood serum in two diseases: juvenile idiopathic arthritis and camptodactyly, arthropathy, coxa-vara and pericarditis syndrome

#### G. Dobson^1,2,3^, M. Wilkinson^1,2,4^, L. Marshall^1,2,4^, L. Wedderburn^1,2,3,4^

##### ^1^Infection, Immunity and Inflammation Programme Research and Teaching Department, UCL Great Ormond Street Institute of Child Health, University College London; ^2^Centre for Adolescent Rheumatology Versus Arthritis at UCL UCLH and GOSH, at UCL UCLH and GOSH, University College London; ^3^Rheumatology, Great Ormond Street Hospital; ^4^NIHR Biomedical Research Centre at GOSH, London, United Kingdom

###### **Correspondence:** G. Dobson

**Introduction:** Lubricin (coded for by the gene *PRG4)* is a mucinous glycoprotein, which provides mechanical lubrication and may also function as an inflammation modulator [1]. Lubricin protein is highly expressed in synovial fluid, blood and many other organs [1,2]. In a rare, autosomal recessive condition, lubricin is absent or non-functioning causing disabling manifestations associated with the disease: camptodactyly, arthropathy, coxa-vara and pericarditis (CACP) [3]. There are limited treatment options for CACP patients as understanding the role of lubricin in normal and diseased states is still being defined [3]. To date, synovial fluid of adult Rheumatoid Arthritis and Osteoarthritis patients have shown reduced lubricin concentrations compared to healthy adult controls [4]. Concentrations in paediatric cohorts have never been described.

**Objectives:** To define the concentration of lubricin protein in blood and synovial fluid of juvenile idiopathic arthritis (JIA) patients, CACP patients, and blood serum of healthy child controls.

**Methods:** The mean age was 7 years (range 1-13 years). Lubricin concentrations in venous blood serum (VBS) and synovial fluid (SFS) were measured by sandwich ELISA (Mybiosource). Samples were from the following patient groups: rheumatoid factor negative polyarticular and oligoarticular JIA patients’ blood (n=15) and synovial fluid (n=13), CACP blood (n=1) and synovial fluid (n=2) and child healthy control blood (n=10). Lubricin concentrations in JIA paired VBS and SFS samples (n=11) were analysed on SPSS by Spearman’s Rank Order Coefficient. Lubricin concentrations in VBS from JIA and CACP patients were compared to data from child healthy controls by Kruskal Wallis ANOVA analysis.

**Results:** The median concentrations of lubricin were 3280ng/ml (IQR: 2640-3491ng/ml) in JIA synovial fluid, 3240ng/ml (IQR: 3085-3394ng/ml) in CACP synovial fluid, 341ng/ml (IQR: 182-645ng/ml) in JIA blood serum, 105ng/ml in CACP blood serum, and 115ng/ml (IQR: 106-195ng/ml) in healthy child control blood serum. The blood serum concentrations variation was significantly different between JIA, CACP and healthy child controls, p=0.008. The relationship of lubricin concentration between JIA paired blood and synovial fluid serum had a correlation coefficient 0.545 (p=0.083).

**Conclusion:** This study is the first to show measure of lubricin in both blood and synovial fluid of paediatric patients. The data suggest that lubricin concentration in JIA blood was significantly higher compared to CACP and healthy child controls, but there was no correlation between levels in VBS compared to SFS. Lubricin measured from the synovial space of CACP patients could be non-functioning lubricin, given the positive phenotype and confirmed PRG4 mutations in these patients. Further work will investigate the functionality of lubricin in patients.

**Disclosure of Interest**

None declared

### P15 Proof-of-concept study of accelerometry to quantify knee joint movement to assist with juvenile idiopathic arthritis diagnosis

#### A. Garner^1^, R. Saatchi^2^, D. Hawley^3^, O. Ward^3^

##### ^1^University of Sheffield Medical School; ^2^Industry and Innovation Research Institute, Sheffield Hallam University; ^3^Department of Paediatric Rheumatology, Sheffield Children's Hospital, Sheffield, United Kingdom

###### **Correspondence:** A. Garner

**Introduction:** Body-worn accelerometers can accurately quantify joint movements, and could potentially assist with the diagnosis of juvenile idiopathic arthritis (JIA) as joint movement restriction is a feature of the condition.

**Objectives:** This proof-of-concept study aimed to evaluate the use of accelerometry to objectively quantify knee joint movements in children with clinically active JIA. Restriction in joint movement can be observed in JIA, so an accurate, cost effective, and portable tool that objectively measures restriction could aid diagnosis.

**Methods:** Seven participants (age: 11.7 (2.7) years) with suspected active arthritis of a single knee joint were recruited. Participants had established diagnoses of oligoarticular (n = 2) and polyarticular JIA (n = 5) and were recruited just prior to planned appointments for steroid joint injections. paediatric Gait, Arms, Legs and Spine (pGALS) examination^1^ was performed by an experienced practitioner on the day of data collection, to confirm the suspicion of active arthritis. The contralateral knee joint acted as reference. An accelerometer is a miniature sensor for measuring movements in three perpendicular dimensions. Four tri-axial accelerometers were integrated individually in soft elastic bands. The data from the accelerometers were collected using a microprocessor and stored on a computer. Accelerometers were placed above and below each knee and participants were asked to perform ten consecutive flexion and extension movements of each knee joint while lying, followed by walking ten meters. Accelerometry data were processed using a data analysis package called Matlab^©^ to quantify knee movement according to range of movement, maximum velocity, maximum acceleration, angular displacement, and period of movement. A participant questionnaire was used to establish procedural acceptability.

**Results:** The accelerometry results were concordant with pGALS examinations in 86% (n = 6) of cases. In all variables measured, the extent of movement was reduced in the knee joint with active JIA, this was most pronounced during flexion and extension movements compared to walking. Joint range of movement had a greater standard deviation (16.6 degrees) and interquartile range (29.1 degrees) in the knees with active JIA compared to the healthy knees during flexion and extension movements, demonstrating inconsistency of movement in the joints with active JIA. There were statistically significant differences between the range of movement (p = 0.032) and angular displacement (p = 0.030) of the knees with active JIA and the healthy contralateral knee joints during flexion and extension. No statistically significant differences were identified between knee joints with active JIA and the healthy knee joints during walking. The questionnaire indicated one participant suggested future improvement in the accelerometer's band design due to discomfort, and the remaining participants found the procedure acceptable.

**Conclusion:** The study demonstrated proof-of-concept for the use of accelerometry to quantify knee joint movement in JIA. It examined accelerometry variables that suitably represented joint movement. It was found that accelerometry has potential for differentiating between joints with active arthritis and unaffected joints, particularly through assessment of flexion and extension movements. Further research is required to confirm these findings and refine the use of this novel technology in children with JIA.

**Reference**

1. Foster HE et al. Musculoskeletal screening examination (pGALS) for school-age children based on the adult GALS screen. Arthritis Rheum. 2006 Oct 15;55(5):709-16.

**Disclosure of Interest**

None declared

### P16 The influence of BMI on JIA course in children

#### M. F. Gicchino, A. Di Sessa, R. Melone, S. Zarrilli, P. Marzuillo, E. Miraglia del Giudice, A. N. Olivieri

##### Department of Woman, Child and General and Specialistic Surgery, University of the Study of Campania Luigi Vanvitelli, Naples, Italy

###### **Correspondence:** M. F. Gicchino

**Introduction:** Juvenile Idiopathic Arthritis (JIA) represents the most common chronic rheumatic disease in childhood affecting joints and other structures. According to International League of Association for Rheumatology (ILAR), seven subtypes of arthritis can be defined in relation with the number of joints and the extra-articular involvement occurring in the first six months of disease. In the last years, greater improvements in long-term outcomes have been provided by new therapeutic options. Growing evidence has supported the role of obesity as a risk factor for severity disease both in adults and children, but recent studies have also showed a potential effect of underweight in this context. To date, the influence of Body mass index (BMI) on JIA course is poorly studied, in particular in children.

**Objectives:** To evaluate the influence of BMI on disease course in children with JIA.

**Methods:** We retrospectively examined 113 children with JIA classified according to ILAR criteria attending our Rheumatology Clinic. At the time of the first visit, anthropometric and laboratory data were assessed. Age at disease onset, disease duration, joint involvements, presence of comorbidities, and medications were also collected. Disease activity was assessed by JADAS-10. According to BMI Z-score, our cohort was divided into five groups: underweight, normal weight, overweight (OW), obesity (OB), and severe obesity. Differences for continuous variables were analysed with the independent-sample t test for normally distributed variables and with the Mann-Whitney test in case of non-normality. Qualitative variables were compared using the chi-squared test. Linear regression was used to investigate the association of JADAS-10 with BMI categories.

**Results:** The mean age was 7.43±4.03 years. Forty-one percent were persistent oligoarticular, 9.1% extended oligoarticular, 23.6% RF- polyarticular, 8.2% RF+ polyarticular, 7.3% systemic, 8.2% enthesitis-related arthritis, and 2.7% psoriatic arthritis. The prevalence of underweight, normal weight, overweight, obesity, and severe obesity was 7.2%, 54.1%,10.8%,17.1%, and 10.8%, respectively. Ferritin levels, erythrocyte sedimentation rate values, and disease duration were significantly higher in patients with severe obesity and in those underweight compared to subjects belonging to normal weight, OW, and OB groups (p=0.02, p=0.03, and p=0.04, respectively). Similarly, underweight patients and those with severe obesity showed the greater JADAS-10 than other groups (p=0.013). Obese patients presented with the greater number of affected joints (p=0.04). More, a major involvement of lower limbs was observed in patients with obesity and severe obesity, including sacroiliac and midfoot joints (p=0.004 and p= 0.005, respectively). These patients had also an increased number of relapses compared to other groups (p=0.025).

**Conclusion:** BMI seems to influence JIA course in children through different mechanisms. In addition to the mechanical role of obesity and its potential pro-inflammatory effect, underweight seems also to affect the course of the disease. This could be explained by hypothesising an impairment on weight gain by active disease, but further studies are needed to a better understanding. In addition to the arthritis control, these findings underscore the need to maintain an adequate BMI through dietary and lifestyle intervention in order to avoid a more unfavourable JIA course.

**Patient Consent Received**

Yes

**Disclosure of Interest**

None declared

### P17 The metabolic perspective in children with JIA

#### A. Di Sessa, M. F. Gicchino, S. Zarrilli, R. Melone, S. Guarino, E. Miraglia del Giudice, A. N. Olivieri

##### Department of Woman, Child and General and Specialistic Surgery, University of the Study of Campania Luigi Vanvitelli, Naples, Italy

###### **Correspondence:** M. F. Gicchino

**Introduction:** Juvenile Idiopathic Arthritis (JIA) is the most common inflammatory chronic disease in childhood. According to International League of Association for Rheumatology (ILAR) seven subtypes of arthritis can be defined in relation with the number of joints and the extra-articular involvement occurring in the first six months of disease. Although it has been largely recognized that these patients are at risk for disease-specific complications and for metabolic syndrome (MetS) in adulthood, very limited data are available on metabolic risk at this age. Robust evidence demonstrates that high acid uric (UA) levels represent a risk factor for cardiometabolic diseases such as MetS, cardiovascular disease, and type 2 diabetes in adults. In children, serum UA levels increased in youth with obesity and metabolic abnormalities.

**Objectives:** To investigate the metabolic risk in children with JIA.

**Methods:** We retrospectively evaluated 113 children affected by JIA classified according to ILAR criteria attending our Rheumatology Clinic. Both clinical and biochemical assessments were performed. Participants were stratified in four groups according to sex-specific quartiles of UA. Disease activity was calculated by Juvenile Arthritis Disease Activity Score 10 (JADAS-10). Differences for continuous variables were analysed with the independent-sample t test for normally distributed variables and with the Mann-Whitney test in case of non-normality. Qualitative variables were compared using the chi-squared test.

**Results:** The mean age of our cohort was 7.43±4.03 years. Systolic blood pressure levels and BMI-Z score significantly increased across quartiles (p= 0.002 and p=0.003, respectively). Patients belonging to the highest UA quartile also showed higher triglycerides and total cholesterol (p=0.01 and p=0.025, respectively) and lower HDL cholesterol levels (p<0.0001) than subjects belonging to the lowest quartiles. JADAS-10 score, ferritin and erythrocyte sedimentation rate levels, and age at disease onset did not significantly differ across UA quartiles (all p >0.05), but a trend for JADAS-10 score was observed (p=0.06). With regard to treatment, the prevalence of the use of biological drugs significantly increased across UA quartiles (p=0.04).

**Conclusion:** A worse cardiometabolic profile across UA quartiles has been observed in children with JIA. Our preliminary data suggest that in clinical practice UA might represent a useful marker of cardiometabolic risk in children with JIA. Taking into account the increased MetS development risk later in life in these patients, a careful global management is highly recommended, by paying attention not only to disease-specific comorbidities (e.g. uveitis, etc) but also to both metabolic and cardiovascular derangements at an earlier stage of JIA. Further studies are needed to better clarify the early cardiometabolic risk in JIA patients and its potential influence on treatment response.

**Patient Consent Received**

Yes

**Disclosure of Interest**

None declared

### P18 Relapse rate and associated factors after discontinuation of biologic dmards in non-systemic JIA patients

#### J. Gieling^1^, B. van den Bemt^2^, E. Hoppenreijs^3^, E. Schatorjé^3^

##### ^1^Department of Pediatric Rheumatology, Pediatrics, Radboud University Medical Center; ^2^Departments of Pharmacy; ^3^Department of Pediatric Rheumatology, Pediatrics, Sint Maartenskliniek / Radboud University Medical Center, Nijmegen, Netherlands

###### **Correspondence:** J. Gieling

**Introduction:** Biologic disease-modifying antirheumatic drugs (bDMARDs) have changed the treatment of juvenile idiopathic arthritis (JIA) patients notably, as nowadays substantially more patients achieve remission. Research has proven their safety and resulted in guidelines upon how and when to start bDMARDs. When sustained remission is achieved, tapering or even discontinuation of the bDMARD is advocated to reduce side effects and costs. However, when and how to discontinue bDMARD therapy once remission is attained and what happens afterwards, is less known.

**Objectives:** With this scoping review we aim to collect available data in the current literature on relapse rate (RR), time to relapse (TTR) and possible flare associated variables (such as time spent in remission and method of discontinuation) after discontinuing bDMARDs in non-systemic JIA patients.

**Methods:** We performed a literature search using the Pubmed database and found 607 articles related to JIA and bDMARDs available for non-systemic JIA treatment. Based on title/abstract and full text screening we obtained 27 articles reporting RR, TTR or factors associated with flare after tapering and/or stopping of bDMARDs.

**Results:** The 27 selected articles included in total 605 non-systemic JIA patients who tapered and/or stopped bDMARD therapy. RR after discontinuation of bDMARDs, either abruptly or following tapering, were 25-48%, 36.8-46.2% and 60-78% at 6, 8 and 12 months respectively. Other studies did not report RR per month but noted a total RR ranging from 26.3-83.3% with a either a mean TTR of 2-8.4 months or a median TTR of 3-8.4 months. All studies stated a good response after restart of therapy after flare. Not all studies reported RR and/or TTR. Results of the largest studies reporting RR are shown in table 1.

JIA subtype, type of bDMARD, concomitant methotrexate use, treatment duration, tapering method, age, sex and time in remission could not conclusively be related to RR or TTR. However, some studies reported a positive association of RR or TTR with ANA-positivity, younger age at disease onset, male sex, disease duration and delayed remission, which were not confirmed in other studies.

**Conclusion:** Flares seem to be common after bDMARD discontinuation, but little is known about which factors influence these flares in JIA patients. Follow up after discontinuation with careful registration of patient variables, information about tapering methods and flare rates are required to better guide tapering and/or stopping of bDMARDs in JIA patients in the future.

**Disclosure of Interest**

None declared

**Table 1 (abstract P18).** Relapse rate in non-systemic JIA patients after withdrawal of bDMARD

Main author (n)	Chang (25*, 7#)	Aquilani (110)	Anink (26)	Lovell (106)	Leong (39)	Iglesias (18)	Windschall (24)	Hissink Muller (19)	Foeldvari (22)
Medication type	Etanercept Adalimumab Infliximab	Etanercept	Etanercept	Etanercept Adalimumab Infliximab	Etanercept Adalimumab Infliximab	Etanercept Adalimumab Infliximab	Etanercept	Etanercept	Etanercept
Time in inactive disease, months, mean (range)	unknown	14.6	at least 6	at least 6	al least 6	12.2	at least 18	5.8 (3-6)	unknown
Follow up duration, months	unknown	12	6	8	8	unknown	unknown	unknown	unknown
% relapse after withdrawal	78%, 76%	60%	46.2%	36.8%	46.2%	77.8%	50%	26.3%	59.1%
Time to relapse, months, mean (range) and/or *[median (range)]*	unknown	*[4.3]*	unknown	7, *[8.3]*	unknown	3	8.4 (4-72)	*[3 (3-6.8)]*	*[7]*
% relapse after 6 months	47%, 48%	40%	46.2%						
% relapse after 8 months				36.8%	46.2%				
% relapse after 12 months	78%, 76%	60%							

*bDMARD with concomitant methotrexate, #bDMARD monotherapy

### P19 Clinical features of juvenile idiopathic arthritis in Batna -Algeria-

#### D. Hadef^1^, S. Slimani^2^, M. C. Khamari^3^, W. Mekaoussi^3^, S. Brahmi^1^, A. Belot^4^, P. Quartier^5^

##### ^1^Department of Pediatrics, University Hospital Center of Batna, Faculty of Medicine, Batna 2 University; ^2^Atlas Clinic of Rheumatology; ^3^Private Clinic of Rheumatology, Batna, Algeria; ^4^Pediatric Nephrology, Rheumatology, Dermatology Unit, Hôpital Femme Mère Enfant, Hospices Civils de Lyon, National Referee Centre for Rheumatic and AutoImmune and Systemic Diseases in Children (RAISE), Lyon; ^5^Pediatric Immunology-Hematology and Rheumatology Unit Necker Hospital, Assistance Publique Hôpitaux de Paris, National Referee Centre for Rheumatic and AutoImmune and Systemic Diseases in Children (RAISE), Paris, France

###### **Correspondence:** D. Hadef

**Introduction:** Juvenile idiopathic arthritis (JIA) is the most common chronic rheumatic disease in childhood. There is a disparity in the prevalence of Juvenile idiopathic arthritis (JIA) subsets between different geographical areas or ethnic groups. In Arabic and African populations, data describing JIA are scarce. However, the epidemiological studies remain the best tool to understand the disease and to improve its management.

**Objectives:** The aim of this study was to determine subtype frequencies, demographic and clinical features of JIA in Batna -Algeria- and to compare the findings with other JIA populations worldwide.

**Methods:** A multicenter retrospective descriptive study was conducted in Batna health centers (public and private sectors), over a seven-year period from January 2013 to December 2019, based on data collected on JIA patients. As public sector source, we referred to the department of pediatrics of the university hospital center (CHU Benflis Touhami Batna), and as private sector source, we referred to private adult rheumatologists based in Batna. The studied variables were: gender, age at the initial symptoms, age at diagnosis, JIA subtype based on International League of Associations for Rheumatology (ILAR) criteria, symptoms at onset, disease duration at the latest follow up, uveitis presence, auto antibodies (antinuclear antibodies, Rheumatoid Factor and anti-CCP) pattern, joint imaging results, JIA medications, JIA status at the time of enrollment and the latest follow-up. The study was approved by local ethics committee of University hospital center of Batna.

**Results:** The study included a total of 69 cases of JIA that were being followed in Batna health centers over the study period. The female to male ratio was 1.83. The median age at diagnosis was 9 years (range 1-16). Forty-six patients (72%) were diagnosed within the first year after disease onset. At the latest follow-up, the median disease duration onset was 1year (range 1-8 years). There were 34 oligoarthritis (49.3%), 9 polyarthritis rheumatoid factor (RF) negative (13%), 8 polyarthritis (RF) positive (11.6%), 6 systemic arthrits (8.7%), 6 enthesitis-related arthritis (8.7%), 3 psoriatic arthritis (4.3%), and 3 undifferentiated arthritis (4.3%). Nine patients (18.7%) were anti-nuclear antibody (ANA) positive, and 21 patients (30.4%) had indeterminate ANA status. Sixty-three patients (91.3%) had benefited from a slit lamp examination, uveitis was found in 7.9% of cases. The used medications included non steroidal anti-inflammatory drugs (NSAIDs) in 54 patients (79.4%), steroids in 37 (54.4 %), intra articular steroid injections in 17 (24.6 %), conventional disease-modifying anti-rheumatic drug (c DMARDs) in 51 (72.5 %), and biologic agents in 11 patients (15.9%).

**Conclusion:** Oligoarthritis was the most common JIA subtype in our study with cases of uveitis at diagnosis. The RF positive polyarthritis frequency was higher than in literature. The use of c DMARDs was common whilst few patients received biologics. Prospective multicenter studies are necessary to better identify the JIA particularities in our country.

**Disclosure of Interest**

None declared

### P20 The consistency of the physician global assessment: a single centre study

#### L. Hussen^1^, M. J. H. Doeleman^1,2^, N. M. Wulffraat^1,2^, M. H. A. Jansen^1,2^, A. van Royen^1,2^, B. J. Prakken^1,2^, B. J. Vastert^1,2^, S. de Roock^1,2^, J. F. Swart^1,2^

##### ^1^Faculty of Medicine, Utrecht University; ^2^Pediatric Rheumatology and Immunology, Wilhelmina Children’s Hospital, Utrecht, Netherlands

###### **Correspondence:** L. Hussen

**Introduction:** With the Juvenile Arthritis Disease Activity Score (JADAS), the disease activity in Juvenile Idiopathic Arthritis (JIA) can be monitored. The JADAS consists of 4 aspects: Physician Global Assessment (PGA), active joint count (AJC), parent of patient Visual Analogue Scale (VAS) of overall wellbeing and the erythrocyte sedimentation rate (ESR). The ESR can be left out in clinical settings, resulting in the clinical Juvenile Disease Activity Score (cJADAS). The overall impact on daily life does not only depend on the number or location of joints, but also on the amount of pain and limitation every separate involved joint causes. These differences are supposed to be reflected by the PGA ^1,2^

**Objectives:** To determine the differences of PGA scores for similar AJC across paediatric rheumatologists in the Wilhelmina Children’s Hospital (WCH) as a part of the (c)JADAS.

**Methods:** This is a monocentric retrospective cohort study with pseudonymized data from the WCH, gathered since 2011. 4 criteria were required for inclusion: (1) have visited a currently active physician, (2) have any subtype of JIA, (3) have a documented PGA, and (4) have an available joint assessment of the visit.[MD1] 972 patients met the inclusion criteria, of which 9704 visits have been observed by 1 of 6 paediatric rheumatologists.

Correlations of PGA scoring across physicians with AJC and weighted active joint count (wAJC) were established with Spearman’s Rank correlation (with 95% CI) for three subcategories of the cohort. The subcategories are based on the number of active joints; (1) an AJC equal to zero, (2) an AJC of 1 to 4 or (3) an AJC of 5 or more. With the Kruskall-Wallis test, and Bonferroni corrections for multiple comparisons, differences were determined in PGA scoring (median, IQR) between physicians considering a single active joint. The joints of the knee, ankle, hip, wrist, jaw and DIP/PIP of the fingers were investigated. A difference of 30 points in PGA scoring was considered clinically relevant as it could make the difference between low, moderate or high disease activity.^3,4^

**Results:** Spearman’s Rank of the PGA and AJC or wAJC for the total cohort showed a high correlation of 0.858 (95% CI 0.850 - 0.865) (p<0.001) and 0,861 (0.854 to 0.869) (p<0.001), respectively. In table 1, Spearman’s Rank correlation coefficients (with 95% CI) for the categories is shown below.

Due to the constant value of the AJC (0), the correlation rank could not be computed for category 1. Spearman’s Rank is noticeably without consideration of category 1, suggesting high consistency of physicians when scoring the PGA without any active joints.

Single joint analysis shows a statistically significant difference in PGA scoring among physicians for the joints of the knee (max. 15, P<0.001), hip (max. 20, P<0.001), ankle (max. 15, P<0.001), wrist (max. 23 P<0.001) and DIP/PIP (max. 5, P=0.005), but not for the jaw (max. 8, P=0.735). The difference in PGA scoring did not exceed 30 points, thus had no clinical relevance.

**Conclusion:** Despite statistically significant differences in PGA scoring for the same median AJC across paediatric rheumatologists in the WCH, the clinical relevance of the difference is negligible. Linear mixed models could give more insight in which patient and disease characteristics contribute to the difference in PGA scoring across paediatric rheumatologists.

**Trial registration identifying number:**

**References**

1. Backström M, Tynjälä P, Ylijoki H, et al. Finding specific 10-joint juvenile arthritis disease activity score (JADAS10) and clinical JADAS10 cut-off values for disease activity levels in non-systemic juvenile idiopathic arthritis: A finnish multicentre study. *Rheumatology (Oxford, England)*. 2016;55(4):615-623.

2. Consolaro A, Ravelli A. Defining criteria for disease activity states in juvenile idiopathic arthritis: what are the optimal JADAS cut-offs? *Rheumatology (Oxford, England)*. 2016;55(4):595-596.

3. Consolaro A, Bracciolini G, Ruperto N, et al. Remission, minimal disease activity, and acceptable symptom state in juvenile idiopathic arthritis: Defining criteria based on the juvenile arthritis disease activity score. *Arthritis Rheum*. 2012;64(7):2366-2374.

4. Consolaro A, Ruperto N, Bracciolini G, et al. Defining criteria for high disease activity in juvenile idiopathic arthritis based on the juvenile arthritis disease activity score. *Ann Rheum Dis*. 2014;73(7):1380-1383.

**Patient Consent Received**

No

**Disclosure of Interest**

None declared

**Table 1 (abstract P20).** Spearman's Rank for the categories

		AJC		wAJC	
	Observations, n (%)	Correlation coefficient	P value	Correlation coefficient	P value
AJC = 0	5739 (59.2%)	-	-	-	-
AJC 1 – 4	3235 (33.3%)	0.386 (0.356 to 0.414)	<0.001	0.448 (0.420 to 0.477)	<0.001
AJC ≥ 5	730 (7.5%)	0.407 (0.338 to 0.472)	<0.001	0.605 (0.551 to 0.652)	<0.001

### P21 Neutrophil count, platelet indices, CRP and their association with the disease activity of juvenile idiopathic arthritis (JIA) patients: a study from Bangladesh

#### M. I. Islam, S. Parvin Sonia, S. A. Rahman

##### Department of Paediatrics, Bangabandhu Sheikh Mujib Medical University, Dhaka, Bangladesh

###### **Correspondence:** M. I. Islam

**Introduction:** Chronic inflammation of the joints in JIA patients are associated with raised levels of serum inflammatory biomarkers that vary according to disease activity. Neutrophil Count, Platelet count, Mean Platelet Volume (MPV), Platelet Distribution Width (PDW), ESR and CRP are the essential inflammatory markers to evaluate JIA patients' disease activity status.

**Objectives:** To assess Neutrophil Count, Platelet count, Mean Platelet Volume (MPV), Platelet Distribution Width (PDW), CRP and Juvenile Arthritis Disease Activity Score (JADAS) in JIA patients and determine the association between them at the initial visit and at six months of follow up after treatment.

**Methods:** This prospective observational study was conducted from March 2019 to December 2020 in the department of Paediatrics, Bangabandhu Sheikh Mujib Medical University,Dhaka, Bangladesh. Fifty newly diagnosed JIA cases who fulfilled the ILAR classification criteria were included in the study. Disease activity was assessed by JADAS 27. A predesigned questionnaire was completed for each patient which included socio-demographic, clinical and laboratory parameters. JADAS 27 and inflammatory biomarkers were evaluated at the initial visit and at follow-up after six months of treatment. Statistical analysis of data was done by using SPSS version 22.

**Results:** Mean neutrophil count, platelet count, and CRP significantly decreased at follow-up after six months of treatment. MPV and PDW were increased at follow-up after six months compared to the initial visit, but PDW change was significant. Mean JADAS 27 also decreased significantly at follow-up compared to initial visit. Neutrophil count, Platelet indices and CRP were significantly associated with JADAS 27.

**Conclusion:** In this study, neutrophil count, platelet indices, and CRP at follow-up after six months of treatment were improved. Significant association of JADAS 27 was found with platelet indices, neutrophil count and CRP

**Trial registration identifying number:** No BSMMU/2019/1431 Date : 12-02-2019

**Disclosure of Interest**

None declared

**Table 1 (abstract P21).** Changes of Leukocyte count, Platelet Indices at initial visit and at follow up

Parameters	Visits	Oligo arthritis (n= 5)	Poly arthritis (RF Positive) (n=2)	Poly arthritis (RF negative) (n=12)	SJIA (n=15 )	ERA (HLA positive) (n= 16 )	TOTAL Patients (N= 50 )
Platelet count (per cubic mm)	N_1_	274000 ±45055	400000 ±28284	462500 ±125923	593333 ±200487	454187 ±146535	477740 ±174957
	N_2_	296200 ±56010	400000 ±70710	423333 ±87835	439333 ±212752	391000 ±88211	404140 ±138275
	**P-value**	**0.547**^**NS**^	**1.00** ^**NS**^	**0.258** ^**NS**^	**0.035**^**S**^	**0.013**^**S**^	**0.003**^**S**^
Platelet distribution width (PDW) (fL)	N_1_	12.96 ±1.99	10.70 ±0.42	10.22 ±1.32	9.27 ±0.77	12.46 ±7.16	10.95 ±4.33
	N_2_	15.20 ±8.01	11.05 ±1.62	18.29 ±12.90	11.24 ±2.18	16.49 ±11.23	15 ±9.54
	**P-value**	**0.572**^**NS**^	**0.751** ^**NS**^	**0.049** ^**S**^	**0.004**^**S**^	**0.278** ^**NS**^	**0.009**^**S**^
Total count of Leukocyte (per cubic mm)	N_1_	8900 ±1024	10500 ±2121	11791 ±2719	16760 ±7099	10850 ±2049	12640 ±5055
	N_2_	8910 ±1582	12650 ±1626	10566 ±3466	12280 ±4334	9118 ±2431	10535 ±3489
	**P-value**	**0.99**^**NS**^	**0.103**^**NS**^	**0.166** ^**NS**^	**0.017** ^**S**^	**0.003** ^**S**^	**0.001**^**S**^

Data were presented as mean ±SD

P value reached from unpaired t-test and p values < 0.05 accepted as statistically significant

SD = Standard Deviation

n = Number of patients in each JIA types

N = Total number of patients

N1= Initial visit

N2= 06 months

S = Statistically Significant

NS = Not Significant

### P22 Anti-TNF agents impair seroprotection in paediatric patients with juvenile idiopathic arthritis and inflammatory bowel disease vaccinated with the meningococcal ACWY vaccin

#### M. Jansen^1^, A. J. J. Sellies^1^, M. Ohm^2^, M. Zijlstra^3^, J. F. Swart^1^, S. J. Vastert^1^, J. Montfrans van^1^, G. C. Joode de^1^, M. Bartels^4^, A. Royen-Kerkhof van^1^, J. G. Wildenbeest^5^, C. A. Lindemans^6^, V. Wolters^3^, R. A. Wennink^7^, F. M. Verduyn-Lunel^8^, J. H. de Boer^7^, M. W. Heijstek^9^, G. A. Berbers^2^, N. M. Wulffraat^1^, on behalf of PRES Vaccination Working Group

##### ^1^Pediatric Immunology and Rheumatology, University Medical Center Utrecht, Utrecht; ^2^Centre for Infectious Disease Control, National Institute for Public Health and the Environment, RIVM, Bilthoven; ^3^Pediatric gastroenterology; ^4^Pediatric Hematology; ^5^Pediatric Infectiology-immunology, University Medical Center Utrecht; ^6^Stem Cell Transplantation, Princess Maxima Centre; ^7^Ophthalmology; ^8^Microbiology and Virology; ^9^Rheumatology and Immunology, University Medical Center Utrecht, Utrecht, Netherlands

###### **Correspondence:** M. Jansen

**Introduction:** In 2018, the Meningococcal C vaccination was replaced by the Meningococcal ACWY (MenACWY) vaccination in the Dutch Immunisation Programme, due to rising numbers of meningococcal W. We investigated the immunogenicity and safety of this vaccine in paediatric patients with (auto)immune disease, here focussing on Juvenile Idiopathic Arthritis (JIA) and Inflammatory Bowel Disease (IBD).

**Objectives:** The primary objective was to assess the immunogenicity of the MenACWY vaccine in paediatric patients with JIA or IBD, compared to the healthy control population. Within the patient cohort we studied the effect of biological DMARDs on seroprotection. The secondary objective was to assess safety of the MenACWY vaccine in patients.

**Methods:** A prospective study was set up in the Wilhelmina Childrens Hospital Utrecht in patients with immune disorders. Immunogenicity measures for meningococcal serotypes A, C, W-135 and Y were performed by the Centre for Infectious Disease Control, National Institute for Public Health and the Environment, using the Fluorescent bead-based Multiplex ImmunoAssay (FMIA). Immunogenicity is reached at titre levels above the cut-off of 2 μg/ml for each meningococcal serotype. Patient were sampled at baseline and 3, 12 and 24 months post-vaccination. Safety was measured by asking for adverse events during the first hospital visit after vaccine administration. Alterations in disease activity were measured by the Clinical Juvenile Arthritis Disease Activity Score-27 (cJADAS-27) in JIA patients, the Paediatric Crohn’s Disease Activity Index (PCDAI) in Crohn’s Disease patients and the Paediatric Ulcerative Colitis Activity Index (PUCAI) in Ulcerative Colitis patients.

**Results:** 222 patients were included (41% male). Median age was 16 years (range 15-19). 80% of the patients was using immunosuppressive drugs of which 47% biologicals, which were all anti TNF-inhibitors. Protected proportions for MenACWY 12 months post-vaccination were respectively 45% for A, 91% for C, 36% for W and 48% for Y compared to 94% for MenACWY in healthy controls. There was a significant difference in seroprotection rates in patients using anti TNF versus no TNF agents at 12 months post-vaccination: for MenC 100 versus 90%, MenW 61% versus 21%, and MenY 77% versus 31%. The MenACWY vaccine did not aggravate JIA or IBD disease and no severe adverse events were observed.

**Conclusion:** The MenACWY vaccine is well tolerated in JIA and IBD patients but less immunogenic compared to healthy controls. Seroprotection rates at 12 months-post vaccination are significantly lower in patients treated with anti-TNF agents. We therefore advice to measure antibodies in patients on anti-TNF 12 months post-vaccination and to consider a booster vaccination accordingly.

**Patient Consent Received**

Yes

**Disclosure of Interest**

None declared

### P23 JIA-uveitis: a B-cell-driven disease?

#### B. Jebson^1,2^, N. de Gruijter^2,3^, A. L. Solebo^1,4^, L. Wedderburn^1,2,4^, E. Rosser^2,3^

##### ^1^UCL GOS Institute of Child Health; ^2^Centre for Adolescent Rheumatology Versus Arthritis; ^3^Centre for Rheumatology, Division of Medicine, UCL; ^4^Great Ormond Street Hospital for Children, NHS, London, United Kingdom

###### **Correspondence:** B. Jebson

**Introduction:** Approximately one third of patients with poly- and oligo-articular juvenile idiopathic arthritis (JIA) are susceptible to developing the comorbidity uveitis (JIA-Uveitis). JIA-Uveitis is commonly asymptomatic and if not detected early, can cause disabling vision loss. At present, all JIA patients are screened for uveitis- often for many years following diagnosis. Given two thirds will never develop eye disease, this wastes both NHS and patient time. There is an unmet need for a clinical test which can identify JIA patients that are at risk of developing JIA-Uveitis. Although a lack of evidence surrounds the connection between JIA & JIA-Uveitis, risk factors such as anti-nuclear antibody (ANA) presence and B-cell infiltrates in JIA-Uveitis eye tissue suggest B-cells may be involved in development of eye disease.

**Objectives:** To explore whether B-cell signatures in the peripheral blood can be used to distinguish JIA-Uveitis patients versus JIA patients with no eye disease.

**Methods:** We performed multi-parameter flow cytometry analysis on peripheral blood mononuclear cells (PBMC) of JIA (no uveitis) n=31 and n=15 JIA-Uveitis patients to explore phenotypic differences in the B-cell compartment (based on expression of CD19, CD24 & CD38). JIA-Uveitis patients included those with current or historical uveitis diagnosed by a paediatric ophthalmologist. Flow cytometry was also used to assess the class switched antibody profiles of B-cells in JIA patients with (n=9) and without eye disease (n=8). For patients to be included as JIA (arthritis only), all previous ophthalmology screenings must have been negative.

**Results:** JIA-U patients showed a significant increase in ‘atypical’ memory B-cells (CD19posCD24loCD38lo, p=0.0029) and a decrease in immature B-cells (CD19posCD24hiCD38hi, p=0.0078) compared to those with no uveitis. JIA-U patients also displayed a higher proportion of class switched B-cells expressing IgG (p=0.0061) and higher levels of potentially anergic IgD+, IgM- B-cells (p=0.0633) when compared with JIA patients.

**Conclusion:** The differences in B-cell proportions in JIA-Uveitis compared with JIA suggest a skew towards a more inflammatory B-cell phenotype. An increase in atypical memory B-cells has shown to be inflammatory in autoimmune conditions such as JIA, while a reduction of immature B-cells suggests a lack of regulatory cell presence. In addition, the increase in IgG producing B-cells and anergic B-cell levels may be contributing to the autoimmune and hyperreactive pathology of JIA-Uveitis. By developing a clinical assay to test the B-cell profile of all JIA patients, we may be able to detect JIA-Uveitis before any eye damage occurs.

**Disclosure of Interest**

B. Jebson: None declared, N. de Gruijter: None declared, A. Solebo: None declared, L. Wedderburn Paid Instructor for: The CLUSTER consortium PI, Prof. Lucy Wedderburn, has partnerships with industrial partners currently: GSK, Abbvie, UCB, sobi & Pfizer. None of these were directly involved in the data analysis or interpretation of this work, E. Rosser: None declared

### P24 CACP syndrome and juvenile polyarthritis – is it a single disease or separate conditions. a familial case

#### Z. Kolkhidova^1^, S. Salugina^1^, A. Shapovalenko^1^, M. Kostik^2^, I. Nikishina^1^, V. Matkava^1^

##### ^1^Pediatric Department, V. A. Nasonova Research Institute of Rheumatology, Moscow; ^2^St-Petersburg Pediatric Medical University, Saint-Petersburg, Russian Federation

###### **Correspondence:** V. Matkava

**Introduction:**

Camptodactyly-arthropathy-coxa vara-pericarditis syndrome (CACP) is a rare autosomal recessive genetic disorder caused by a mutation in the PRG4 gene. It is characterized by congenital or early-onset camptodactyly and symmetric polyarticular non-inflammatory arthropathy of large joints with synovial hyperplasia, coxa vara, non-inflammatory pericarditis / pleurisy, and the absence of inflammatory markers. Symptoms appear from birth and do not differ by sex or race. This condition more common among children of blood related couples. Currently, there is no pathogenetic therapy. NSAIDs are used for symptomatic treatment and hip arthroplasty is used as surgical treatment of coxa vara. Therapy used for juvenile idiopathic arthritis (JIA) is ineffective.

**Objectives:**

To report a case of CACP in 2 siblings (sister and brother) in rheumatological practice.

**Methods:**

The patients were examined in the rheumatology department. Both patients were diagnosed with the CACP using genetic analysis - Sanger sequencing. Two PRG4 mutations were identified on chromosome 1q25-q31 (pathogenic p.K918fs * 10 and probably pathogenic p.T1161Hfs * 2).

**Results:**

Case report: Girl and boy born in 2014 and 2017, respectively. Parents are not blood related. Table 1 shows the clinical and demographic characteristics of patients. From the moment of birth, both had contractures of the metacarpophalangeal joints of the hands with further rapid formation of symmetric exudative total polyarthritis without signs of significant inflammatory, including laboratory activity. Type 6 mucopolysaccharidosis and Blau's syndrome were excluded in the girl. Both were diagnosed with JIA and treated with methotrexate subcutaneously, GC per os, intra-articular steroid injections with insufficient effect. During the one year, the girl received the TNF-inhibitor etanercept with a partial response. Patients receive the IL-6 inhibitor tocilizumab with an 30% of ACR response. Nevertheless, both pts currently have polyarthritis with joints effusion and moderate functional impairments, flexion contractures in the small joints of the hands.

**Conclusion:**

Due to the polyarthropathy pts with CACP syndrome can meet in the practice of rheumatologist under the mask of JIA. Both our pts had polyarthritis, no laboratory activity, they received antirheumatic therapy, including biologics with insufficient effect, but partial respond. The question of whether arthritis in our patients belongs to the CACP manifestation or is a separate disease has not been answered yet. Further study requires

**Patient Consent Received**

Yes

**Disclosure of Interest**

None declared

**Table 1 (abstract P24).** Clinical and demographic characteristics of patients and in compare to literature data

	Girl A, 6 years	Boy B, 3 years 9 months	Literature data
Sex (M / F)	F	M	1,3 / 1
Age of onset	From birth	From birth	1-24 months
Age of diagnosis (years)	4	1	-
Camptodactyly	+	+	68%-100%
Coxa vara	+	+	70%-100%
Polyarthritis Pain, morning stiffness	+	+	45,7-100%
Pericarditis/ Pleurisy	+	+	7,6-50%
Laboratory activity	-	-	Rarely
	No	No	No
Therapy/Response to therapy:			Traditional antirheumatic therapy is not effective.
Methotrexate	+/-	+/-	
GC per os	+/ partial	+/ partial	
Etanercept	+/ partial	-	
Tocilizumab	+/ partial	+/ partial	

### P25 Joints involvement in two diseases: mucopolysaccharidosis and juvenile idiopathic arthritis or how do not confuse them up with each other

#### N. Buchinskaya^1^, N. Vashakmadze^2,3^, L. Sorokina^4^, M. Kostik^4^

##### ^1^Saint-Petersburg State Medical Diagnostic Center (Genetic medical center), Saint-Petersburg; ^2^Pirogov Medical University; ^3^Central Clinical Hospital RAS, Moscow; ^4^Saint-Petersburg State Pediatric Medical University, Saint-Petersburg, Russian Federation

###### **Correspondence:** M. Kostik

**Introduction:** Mucopolysaccharidosis (MPS) is an inherited metabolic disease which can involve joints and be close resemble to juvenile idiopathic arthritis (JIA).

**Objectives:** To evaluate discriminative features between MPS and JIA.

**Methods:** In the retrospective study we included 255 children with RF-negative polyarthicular JIA and 155 patients with MPS with joints involvement. MPS patients were I type (n=42), II type (n=67), III type (n=27), IV type (n=9), VI type (n=10). We calculated involved joints, hematology, biochemistry markers, patient’s demographics.

**Results:**

**Conclusion:** MPS and JIA have similar inflammatory parameters. Early onset age, growth delay and predominantly involvement of upper arm joints can help to discriminate two diseases.

This work supported by the Russian Foundation for Basic Research (grant № 18-515-57001)

**Patient Consent Received**

No

**Disclosure of Interest**

None declared

**Table 1 (abstract P25).** See text for description

Parameter	MPS (n=155)	JIA (n=255)	p	Parameter	MPS (n=155)	JIA (n=255)	p
Females, n (%)	42 (27)	186 (73)	0.000001	Shoulder, n (%)	26 (61)	27 (11)	0.0000001
Onset age, y	1.0 (0.4; 2.0)	4.7 (2.1; 8.8)	0.0000001	Elbow, n (%)	39 (91)	60 (24)	0.000001
Hemoglobin, g/l	129 (122; 135)	123 (116; 130)	0.0000001	Wrist, n (%)	39 (91)	114 (45)	0.0000001
PLT*10^9^/l	249 (193; 322)	328 (275; 400)	0.0000001	MCP, n (%)	39 (91)	114 (45)	0.0000001
Height, cm	109 (96; 120)	130 (104; 151)	0.0000001	PIP, n (%)	40 (90)	125 (49)	0.0000001
Weight, kg	22 (17; 28)	27 (16; 43)	0.001	DIP, n (%)	39 (91)	41 (16)	0.000001
Active joints	58 (36; 61)	11 (7; 18)	0.0000001	Hip, n (%)	25 (58)	49 (19)	0.0000001
Cervical spine, n (%)	18 (42)	48 (18)	0.0008	MTP, n (%)	24 (56)	53 (21)	0.000001
TMJ, n (%)	0 (0)	24 (9)	0.036	Foot IF, n (%)	25 (58)	51 (20)	0.0000001

### P26 evaluation of the safety and effectiveness of vaccination of children against measles, rubella, mumps and diphtheria with juvenile idiopathic arthritis: data from a cross-sectional study

#### N. Lyubimova^1^, I. Fridman^2^, O. Goleva^2^, S. Kharit^2,3^, M. Kostik^1,3^

##### ^1^Almazov National Medical Research Centre, Russian Federation; ^2^Pediatric Research and Clinical Center for Infection Diseases; ^3^Saint Petersburg State Pediatric Medical University, Saint Petersburg, Russian Federation

###### **Correspondence:** M. Kostik

**Introduction:** Patients with juvenile idiopathic arthritis (JIA) often stop vaccination after the onset of the disease for fear of relapse or worsening of the course, although international experience shows the effectiveness and safety of vaccination in patients with immunocompromised diseases.

**Objectives:** To evaluate the efficacy and safety of vaccination against measles, rubella, mumps, and diphtheria in patients with JIA who continued to be vaccinated after the onset of JIA.

**Methods:** In a cross-sectional study, from a database containing information on 170 patients with JIA aged 2 to 17 years, who were identified with antibodies against measles, rubella, mumps and diphtheria, patients were selected who continued to be vaccinated against measles, rubella and mumps (n=19) and diphtheria (n=25) or refused to be vaccinated against MMR (n=39) and diphtheria (n=51), due to the development of JIA. The decision on vaccination was entirely voluntary, made by the legal representatives of the patient together with the district pediatrician or the rheumatologist together with the immunologist of the child health center. All legal representatives signed an informed consent for revaccination. The study selected patients whose age corresponded to the terms of revaccination, according to the National Schedule of Vaccinations of the Russian Federation. The diagnosis of JIA was established based on the ILAR criteria. In all patients, the levels of antibodies (IgG) against vaccines were determined using ELISA. The data is presented with a median and 25%>75%.

**Results:** Children with the oligoarticular variant of JIA were more often subjected to revaccination against measles, rubella, and mumps. There were no significant differences in antibody levels and the proportion of patients who did not have protective antibodies against measles, rubella and mumps between the compared groups, as well as differences in the characteristics of the course of JIA and the therapy. Patients with less severe JIA, less frequently treated with methotrexate, and less likely to need both primary administration of biologics and switching between biologics were more likely to be revaccinated against diphtheria. Vaccination against diphtheria was effective, as evidenced by an almost twofold prevalence of patients with a protective antibody titer compared to those who refused revaccination. Methotrexate (OR = 9.5 [95%CI: 1,004; 90.3]) and biologics (OR=4.4 [95%CI: 1.6; 12.1]) were predictors of failure to revaccinate against diphtheria. Serious adverse events, as well as JIA flares in the 3-month period after vaccination were not recorded.

**Conclusion:** Vaccination of children with juvenile idiopathic arthritis against measles, rubella, mumps and diphtheria is effective and safe. Further research is needed to increase physicians' confidence in vaccinating children with rheumatic diseases.

This work supported by the Russian Foundation for Basic Research (grant № 18-515-57001)

**Disclosure of Interest**

None declared

**Table 1 (abstract P26).** See text for description

Parameter	Vaccination against MMR		р	Vaccination against Diphtheria		p
	continued (n=19)	declined (n=39)		continued (n=25)	declined (n=51)	
Duration of the JIA, g.	6.9 (6.3;11.6)	5.5 (3.2; 8.8)	**0.031**	6.5 (4.9; 8.2)	6.2 (3.6; 9.5)	0.699
Antibodies, IgG, IU/mL (*measles only)	0.2* (0,01; 0.6)	0,16* (0.0; 0,46)	0.775	0.14 (0.1; 0.34)	0.06 (0.02; 0.22)	0.695
Patients with a protective titer, n (%)	9 (47.4)	19 (48.7)	0.923	15 (60.0)	18 (35.3)	**0.041**
Time since the last vaccination, y.	4.8 (4.3;6.8)	5.3 (3.6; 7.3)	0.772	5.6 (3.6; 10.3)	6.7 (4.0; 10.7)	**0.025**

### P27 Investigation of the immunoregulatory role of the PD-1 pathway in juvenile idiopathic arthritis - preliminary results

#### A. Koutsonikoli, A. Taparkou, P. Pratsidou-Gertsi, V. Sgouropoulou, M. Trachana

##### Pediatric Immunology and Rheumatology Referral Centre, 1st Pediatric Dept, Aristotle University of Thessaloniki, Hippokrateio General Hospital, Thessaloniki, Greece

###### **Correspondence:** A. Koutsonikoli

**Introduction:** The Programmed cell Death protein-1 (PD-1) pathway promotes self-tolerance, by inhibiting immune responses. The soluble form of PD-1 (sPD-1) may antagonize the binding of the membrane-bound PD-1 with its ligands, leading to the blocking of the pathway’s functions. Data regarding the role of the PD-1 pathway in Juvenile Idiopathic Arthritis (JIA) are still limited.

**Objectives:** To investigate the immunoregulatory role of the PD-1 pathway in JIA patients.

**Methods:** A. Determination of sPD-1 levels in serum and synovial fluid (SF) samples using ELISA. B. Analysis of the PD-1 expression on T-helper and T-cytotoxic cells in the peripheral blood (PB) and SF, by applying flow cytometry. C. Search for an association between the above biomarkers, as well as their relation with JIA activity. Inactive disease was defined according to Wallace criteria.

**Results:** Fifty-six Caucasian patients (39 female) participated in this study, with a median (range) age of 13 (2-19) years, with oligoarthritic (33%), polyarthritic (29%), psoriatic (9%), enthesitis-related (14%), systemic (11%) and undifferentiated (4%) JIA. There was no correlation between the sPD-1 levels and the PD-1 cellular surface expression (n=16 PB/serum, n=11 SF). The median sPD-1 was statistically significantly higher in the SF [1104.4pg/ml (560.4-1419)] than in the serum [773.4pg/ml (215.4-980.4)] (n=7) (p=0.028). The median serum sPD-1 was statistically significantly higher in patients with active JIA [218.3pg/ml (149.8-980.4)] (n=22) than in those with an inactive disease [186.7pg/ml (46.8-340.4)] (n=10) (p=0.035). The median percentage of positive for PD-1 T-helper cells in PB was statistically significantly higher in patients with active JIA [1.79% (0.14-19.1)] (n=26) than in those with inactive disease [0.16% (0.1-2.43)] (n=6) (p=0.006). The median percentage of positive for PD-1 T-cytotoxic cells in PB was statistically significantly higher in patients with active JIA [2.65% (0.73-12.93)] (n=26) than in those with inactive disease [0.55% (0.26-4.2)] (n=6) (p=0.014).

**Conclusion:** These preliminary results indicate that the sPD-1 levels rise in active JIA, more prominently in the inflamed joint than in the PB. Also, in active JIA a higher number of T-helper and T-cytotoxic cells expressing PD-1 were detected. Further investigation in a larger sample of JIA patients may verify these observations and contribute to unraveling the precise role of the PD-1 pathway in the pathogenesis and maintenance of the joint inflammation.

**Disclosure of Interest**

None declared

### P28 Multifactorial aspects of IGA nephropathy onset in a 13 year-old boy affected by oligoarticular juvenile idiopathic arthritis (JIA) with ongoing adalimumab treatment

#### B. Lattanzi^1^, A. Omenetti^1^, A. Ranghino^2^, L. Caponi^3^, S. Cazzato^1^

##### ^1^Pediatric Unit, Salesi Children's Hospital; ^2^Nephrology, Dialysis and Transplantation Unit, AOU Ospedali Riuniti; ^3^Department of Pediatria, Polytechnic University of Marche, Ancona, Italy

###### **Correspondence:** B. Lattanzi

**Introduction:** According to anecdotal reports, renal involvement due to uncontrolled inflammation or long exposure to anti-rheumatic drugs, may rarely occur during juvenile idiopathic arthritis (JIA). Most of these cases refer to systemic JIA, associated with inflammation and, eventually, renal amyloidosis. Little is known about oligoarticular JIA. Biological agents, such as adalimumab, can induce IgA nephropathy but this usually resolves following cessation of therapy.

**Objectives:** To highlight potential aetiology of renal involvement in oligoarticular JIA.

**Methods:** We herein report a case of a 13 years old boy affected by JIA and uveitis, who developed proteinuria and haematuria during adalimumab treatment.

**Results:** The patient was diagnosed by ANA positive oligoarticular JIA at the age of 3. He was initially treated with intra-articular steroid injections and methotrexate, with subsequent switch to etanercept due to frequent articular relapses. Despite complete remission, he later presented relapsing uveitis for which adalimumab was started in Oct 2017. Complete remission was successfully obtained and maintained with ongoing treatment. However, on Nov 2020, unforeseen proteinuria and haematuria occurred regardless persistent JIA remission. Drug-induced renal damage was considered and adalimumab promptly discontinued. Infectious triggers were ruled out. Autoimmune profiling was unremarkable (i.e. negative ENA, dsDNA, p-ANCA, c-ANCA, p anti-phospholipase A2 receptor and glomerular basal membrane antibodies antibodies, anti-thyroid antibodies) except for known ANA positivity (1:160). Complement fractions were in normal range whereas IgA levels were slightly elevated (330 mg/dl, normal values 61-301). Intriguingly, laboratory tests unveiled the presence of HLA-DQ2/DQ8 positivity and IgA-class tissue transglutaminase antibodies (tTGA), which were persistently negative at previous yearly screening. Coeliac disease may actually elicit IgA nephropathy with consequent beneficial effect of gluten-free diet. However, tTGA levels remained in borderline ranges at following assessments, not allowing a definitive serological diagnosis. Meanwhile, given worsening course of proteinuria and haematuria, renal biopsy was performed, and unveiled findings consistent with mesangial IgA nephropathy. Unfortunately, discontinuation of biological therapy resulted in both ocular and articular disease relapse. In order to rapidly target either kidney and JIA disease manifestations, systemic corticosteroids regimen according to “Pozzi protocol” was started with prompt improvement of both proteinuria and haematuria, and resolution of articular and ocular relapse.

**Conclusion:** The case herein presented addresses the multifactorial putative causes underlying onset of an IgA nephropathy in oligoarticular JIA. In particular, this report rises several considerations: 1) the patient developed IgA nephropathy during adalimumab therapy, with worsening proteinuria and haematuria despite drug discontinuation, weaking the drug-induced hypothesis; 3) several evidence indicate a role for gut-renal connection in IgA nephropathy onset and celiac disease is part of the autoimmune clinical spectrum including JIA. In conclusion, due to rarity of reports demonstrating safety of other anti-TNFα agents, once renal remission will be achieved by the ongoing steroid regimen, JIA maintaining therapy with different biologic drug (i.e. abatacept) will be considered. In addition, the ongoing suspicious celiac disease onset will need to be confirmed or excluded by small bowel biopsy as soon as the high dose steroid regimen will be concluded in other to avoid potential relapse of IgA nephropathy.

**Patient Consent Received**

Yes

**Disclosure of Interest**

None declared

### P29 Evaluation of the immunological profile and vacinal status of patients with juvenile idiophatic arthritis

#### M. Lopes^1^, L. A. C. Cronemberger^2^, C. F. C. Valença^2^, F. S. Tavares^2^, A. G. Islabão^1^

##### ^1^Rheumatology; ^2^Immunology, Hospital da Criança de Brasília José Alencar, Brasília, Brazil

###### **Correspondence:** M. Lopes

**Introduction:** Children and adolescents with Juvenile Idiopathic Arthritis (JIA) suffer a great impact on quality of life, due to changes caused by the disease and association with the treatment, which increase the susceptibility to infections. Thus, a safe and effective vaccination is important. B cells have an important role in immune responses with antibodies production against pathogens and response to vaccines.

**Objectives:** Due to the scarce literature on this topic, the main objective of this study was to trace the immunological profile including lymphocytes subsets and vaccine response of patients with JIA being followed up at the pediatric rheumatology outpatient reference clinic.

**Methods:** An observational, analytical, cross-sectional study was carried out, obtaining data through the collection of laboratory tests and charts in the period from April to December 2020. Consecutive patients diagnosed with JIA according to the International League of Associations for Rheumatology (ILAR) were included.

The evaluated subtypes of JIA were oligoarticular, polyarticular, systemic, enthesitis-related arthritis and undifferentiated. Each patient underwent a single laboratory collection. The tests used were blood count, immunoglobulins (IgM, IgG, IgA and IgE), lymphocyte profile by flow cytometry (CD3, CD4, CD8, CD19 and CD56), serology IgG for measles and rubella and anti-HBs for hepatitis B.

**Results:** Sixty patients, out of the eighty-nine diagnosed with JIA, were included. Median age was 11 years and 3 months and the most prevalent gender was female 44/60 (73.3%).

Among the patients studied, 37/60 (62.7%) had positive Anti-Nuclear Antibodies Hep-2 (*ANA*) and 10/60 (16.6%) positive Rheumatoid Factor (RF). The most prevalent JIA subtype was the oligoarticular 28/60 (46.6%) and polyarticular 24/60 (40%). The main hematological alteration found was lymphopenia, in 14/60 (23.3%) patients. The number of patients with reduced IgA levels corresponded to 12/60 (20%), with no emphasis on the reduction of IgM, IgG or IgE. Among the evaluated patients, 37/60 (63.7%) presented serology that was not reactive to hepatitis B, 10/60 (17.8%) to measles and 6/10 (10.3%) to rubella. Regarding to lymphocytes subsets, one patient did not collected the exam. Observed that 11/59 (18.6%) had CD3 below the 10th percentile (p10), 8/59 (13.5%) CD4 below the p10, 12/59 (20.3%) CD8 below p10, 40/59 (67.8%) CD19 below p10, 27 (45%) CD56 below p10.

**Conclusion:** In conclusion, our study demonstrated an important reduction in lymphocytes subsets, mainly CD19 and CD56, and protection against hepatitis B. We also evidenced JIA association with IgA deficiency.

**Disclosure of Interest**

None declared

### P30 Effects of Humira in children with juvenile idiopathic arthritis (JIA) and polymorphic manifestations of uveitis- in example of one rheumatology center in Georgia

#### M. Lekishvili^1^, K. Mamamtavrishvili^2^, M. Ioseliani^1^

##### ^1^Rheumatology; ^2^New Hospitals, Tbilisi, Georgia

###### **Correspondence:** K. Mamamtavrishvili

**Introduction:** JIA is the most prevalent rheumatologic disease among children and is commonly associated with chronic uveitis. Without adequate control of ocular inflammation, patients may develop complications leading to permanent visual loss.

**Objectives:** 4 female patients starting from 4 to 20 years of age, length of JIA varies between the months and 15 years.

**Methods:** Patient presented with various ophthalmic complications such as:

Case #1: Cataract, peripheral corneal dystrophy, Iritis, cystoid macular edema of right eye, glaukoma and artiphakia of left eye (20 y/o female)

Treatment Humira 40 mg per 2 weeks.

Case #2: Artiphakia and vitritis of both eyes, (8 y/o female)

Treatment Humira 20 mg per 2 weeks.

Case# 3. Uveitis and vitritis of both eyes (5 y/o female)

Treatment Humira 20 mg per 2 weeks, Methothrexate, 5 mg orally once a week

Case # 4 Unilateral uveal cataract (5 y/o female)

Treatment Humira 20 mg per 2 weeks, Methothrexate, 7,5 mg orally once a week, Methilprednizolone 2 mg orally daily

In all this cases methothrexate treatment alone was not enough for ocular complication.

**Results:** : visual acuity was improved in all 4 cases due to this treatment.

Case #1 Cystoid macular edema reduced

Case # 2 Vitreous body is partially transparent

Case # 3 Condition of both eyes is markedly improved

Case #4 Cataract surgery was performed without postoperative complication

**Conclusion:** Humira was successful in all 4 cases.

Humira was proven to be effective and should be considered in treatment in JAI complicated with the various ophthalmic problems.

**Disclosure of Interest**

None declared

### P31 TNF-α inhibition pre and post-rituximab in JIA: what shall we expect? A pilot study

#### A. Marino^1^, F. Pregnolato^2^, F. Orsini^3^, I. Pontikaki^1^, M. V. Gattinara^1^, R. Cimaz^2^

##### ^1^ASST G.PINI-CTO; ^2^Department of Clinical Sciences and Community Health, and Research Center for Adult and Pediatric Rheumatic Diseases; ^3^University of Milan, Milan, Italy

###### **Correspondence:** A. Marino

**Introduction:** Biologic agents have revolutionized the treatment of Juvenile idiopathic arthritis (JIA). However, difficult to treat patients need several biological swaps. In this context, it is important to improve the effectiveness of available drugs and to spare our weapons with a lifespan perspective. The resetting of peripheral B cells promoted by rituximab (RTX), a chimeric monoclonal antibody against B-cell antigen CD20, represents a quite interesting option in autoimmune diseases such as JIA not only for the immediate advantage but also for the possible future use of subsequent therapies that might be necessary to achieve disease control.

**Objectives:** To evaluate whether TNF-α inhibition, which had already been used could be repurposed after RTX therapy.

**Methods:** This is a retrospective pilot study involving JIA patients who took a TNF-α inhibitior before and after having been treated with RTX. Clinical and laboratory data were collected and statistically analyzed. Clinical status, number of flares, and retention on treatment were then evaluated at different time points. Confidence intervals (CI) lower than 95% were used to estimate a probabilistic range of pre and post-RTX variations and trend was considered relevant when at least one of CI excluded the reference value of “no change”. As the sample size was limited, a bootstrapping procedure (i.e. a statistical procedure that resamples a single dataset to create many simulated samples) was used to estimate disease activity parameters and related CI.

**Results:** Eight patients (6 girls) were identified: 4 patients with oligoarticular JIA, 2 with psoriatic JIA, 1 with systemic JIA and 1 with RF-negative polyarticular JIA. 6/8 patients were ANA positive. The median (range) age at disease onset, at the selected biologic agent pre-RTX (SB pre-RTX), at RTX onset, at the SB post-RTX were 2.1 years (1.2-15.8), 17.8 years (9.6-49.9), 21.6 years (15.3-51.6), and 23.2 years (17.4-52.5), respectively.

Clinical remission was achieved in 7 patients on SB pre-RTX and in 8 patients on SB post-RTX; furthermore, the median time to achieve clinical remission was shorter (2 months vs 3 months) before and after RTX. Both the remission duration and the retention time of the TNF-α inhibitor were longer (3.9 years vs 2.2 years) on the SB post-RTX.

After adjustment for the exposition time, the median number of flares was 1.03 (0.29 to 3.02) during the treatment with TNF-α inhibitor pre-RTX and decreases post-RTX to 0.64 (0.0 to 2.09).

The bootstrapped differences of remission time, TNF-α inhibitor retention rate and flare rate ratio of post- versus pre-RTX are shown in Table 1. As the reference value of no change (0 or 1 according to the effect measure used) does not fall within the 95% and 90% CI, our findings highlight a trend towards a substantial variation.

**Conclusion:** This pilot study documented a possible amelioration of disease response to TNF-α inhibition after treatment with RTX. A larger cohort is advisable in order to verify this opportunity in the context of a chronic disease starting in early childhood.

**Disclosure of Interest**

None declared

**Table 1 (abstract P31).** Bootstrapped estimates of measures of effect and related CI post- vs pre-RTX

Estimate	Flare rate ratio - ref. value = 1	Diff. time on remission (months) – ref value = 0	Diff. retention time (years) – ref value =0
Mean [95% CI]	0.54 [0.27 to 0.82]	18.3 [-0.7 to 38.6]	2.1 [0.2 to 4.1]
Mean [90% CI]	0.54 [0.31 to 0.77]	18.3 [2.5 to 35.4]	2.1 [0.5 to 3.8]
Mean [85% CI]	0.54 [0.34 to 0.74]	18.3 [4.5 to 33.3]	2.1 [0.7 to 3.6]
Mean [80% CI]	0.54 [0.36 to 0.72]	18.3 [6.1 to 31.8]	2.1 [0.9 to 3.4]
Mean [75% CI]	0.54 [0.38 to 0.7]	18.3 [7.4 to 30.5]	2.1 [1.0 to 3.3]

### P32 Toll-like receptor 4 expression on peripheral blood mononuclear cells in juvenile idiopathic arthritis

#### O. Mukvich, A. Matskevych

##### Department of Pediatric Rheumatology and Autoinflammatory Diseases, State Institution “Institute of Pediatrics, Obstetrics and Gynecology of NAMS of Ukraine”, Kyiv, Ukraine

###### **Correspondence:** O. Mukvich

**Introduction:** Juvenile idiopathic arthritis (JIA) is currently considered a heterogeneous group of inflammatory arthritis of unknown etiology (ACR, 2019). The formation of its pathological mechanisms is due to dysregulation of both adaptive and innate immunity, the key ligands of which are toll-like receptors (TLR). TLR4 play an important role in the recognition of inflammation caused by bacterial lipopolysaccharides (LPS), activation of the nuclear factor-κB (NF-κB) and other intracellular signaling pathways with subsequent expression of pro-inflammatory cytokine genes. An increased expression of endogenous TLR4 ligands (heat shock proteins, fibronectin, fibrinogen, HSP, EDA, etc.) has been found in the tissue synoviocytes and peripheral blood monocytes, which are recruited to the site of inflammation and participate in the pathogenesis of synovial inflammation, which is believed to be an important mechanism in the pathogenesis of rheumatoid arthritis. To clarify the pathogenetic role of TLR4, the levels of its stimulation in different subtypes of JIA are of interest.

**Objectives:** To determine the levels of TLR4 expression on CD14+ monocytes in the heparinized whole blood in children with different subtypes of JIA.

**Methods:** 62 children from 10 to 17 years old were examined, including 42 children diagnosed with JIA and 20 healthy children. Patients with JIA were stratified by subtypes: systemic - 6, polyarthritis -17, oligoarthritis - 19 children. The intensity of TLR4 expression on CD14+ monocytes was determined in the heparinized whole blood during incubation with a cocktail of CD14-FITC/TLR4-PE monoclonal antibodies (Biolegend, USA) using flow cytometry. The Student's t-test was used to assess the differences between the groups in cases of normal distribution. The difference with p<0.05 was considered significant.

**Results:** Patients diagnosed with JIA had an increased expression of TLR4 - (51.51±6.18)%, which showed a statistical difference (t=3.01, α=0.05, p=0.0033), compared to the control group of healthy children (23.47±6.68)%. The level of its expression was (54.43±12.10)% (t=2.24, α=0.05, p=0.033) in children with oligoarthritis and (52.86±7.14)% (t=3.01, α=0.05, p=0.005) in children with polyarthritis. The highest level of stimulation was determined in children with oligoarthritis, which was 2.3 times higher, compared to the control group. In systemic JIA, TLR4 expression was lower (38.48±10.32)% and showed no statistical differences compared to healthy children (t=2.12, p>0.05).

**Conclusion:** High expression of TLR4 on CD14+ blood monocytes, which are able to migrate to synovial membranes and increase the synthesis of pro-inflammatory cytokines and chemokines, determines the role of innate immunity, and the TLR signaling pathway in particular, in the formation and persistence of chronic inflammation induced by microbial ligands. The obtained data indicates the possibility of differences in the formation of inflammatory mechanisms in articular and systemic JIA. The implementation of the TLR4 signaling pathway orientation in the clinical practice has a therapeutic potential in JIA

**Patient Consent Received**

Yes

**Disclosure of Interest**

None declared

### P33 Predictive factors of uveitis and its complications in a cohort of 302 JIA patients

#### A. T. Melo^1,2^, J. M. Martinho^1,2^, P. Martins^1,2^, R. Ferreira^3^, P. José^3^, S. Mano^3^, I. Leal^3^, P. C. Reis^2,4,5^, F. O. Ramos^1,2,4^, J. E. Fonseca^1,2^, R. C. Marques^1,2,4^

##### ^1^Serviço de Reumatologia e Doenças Ósseas Metabólicas, Hospital de Santa Maria, Chuln; ^2^Unidade de Investigação em Reumatologia, Instituto de Medicina Molecular, Faculdade de Medicina, Universidade de Lisboa; ^3^Serviço de Oftalmologia; ^4^Unidade de Reumatologia Pediátrica; ^5^Serviço de Pediatria, Hospital de Santa Maria, Chuln, Lisbon, Portugal

###### **Correspondence:** A. T. Melo

**Introduction:** Screening and prompt diagnosis are essential to avoid complications, such as cataracts and glaucoma in uveitis.

**Objectives:** To identify clinical or laboratorial predictive factors of uveitis complications in patients with juvenile idiopathic arthritis (JIA).

**Methods:** A retrospective observational study of patients with JIA, registered at the Portuguese Rheumatic Diseases Register (Reuma.pt) was performed. Demographic variables, data on presence of uveitis and its complications (synechiae, band keratopathy, glaucoma, cataracts, macular edema), clinical features and treatment were collected and complemented with hospital clinical data. Statistical analysis was done using SPSS 26.0, with a significance of p<0.05. Univariate analysis was performed using Fisher’s exact test, Mann-Whitney U test or Chi-Square. Multivariate analysis was also performed.

**Results:** We included 302 JIA patients, 59.6% females, with a mean age at JIA onset of 8.4±4.8 years and a mean disease duration of 12.8±11.1 years. Uveitis was identified in 52 of the 302 patients (17.2%), with a mean age at uveitis onset of 11.3± 9.4 years. The mean time since JIA diagnosis until uveitis onset was 5.4± 8.3 years. Only in 2 cases uveitis started before JIA. Uveitis developed only in adulthood in 11 of those patients.

Younger age at JIA diagnosis was associated with uveitis (6.2 [4.5] vs 8.9 [4.7], p=0.01). Oligoarticular persistent (opJIA) (23.7% vs 14.1%, p=0.040) and extended form (oeJIA) (39.3% vs 15.0%, p=0.003) were also positively correlated with the presence of uveitis. On the other hand, polyarticular JIA had a lower frequency of uveitis (7.9% vs 20.4%, p=0.013). First uveitis episode at adulthood was more common in JIA ERA patients (15.4% vs 62.5%, p=0.011), whereas uveitis onset <18years old was associated with an opJIA form (36.0% vs 9.0%, p=0.03).

Uveitis was more frequent in patients with positive antinuclear antibodies (ANA) (25.8% vs 13.6%, p=0.012), however, there were no significant differences between the ANA title (≤1/160 vs > 1/160) or pattern. We found no association between the development of uveitis and positivity to rheumatoid factor, anticyclic citrullinated peptides, human leukocyte antigen B27 or with inflammatory markers. Multivariate analyses showed that opJIA and oeJIA were independent predictors of uveitis (OR 2.9 95%CI: 1.3-6.6; OR 6.1 95% CI: 2.3-16.2; respectively)

Ocular complications occurred in 15 patients out of the 52 patients (28.3%): synechiae occurred in 28.3%, band keratopathy in 22.7%, cataracts in 17%, glaucoma in 15.1%, macular edema in 7.6%. We found an association between oeJIA form and the development of glaucoma (80% vs 22%, p=0.033) and with the need for ocular surgical procedures (75.0% vs 14.8%, p=0.028). Multivariate analyses showed that oeJIA form was an independent predictor of ocular surgical procedures (OR 14.9 95% CI: 1.2-193.4).

**Conclusion:** Uveitis was more frequent in opJIA and oeJIA patients and was related to ANA positivity, which is consistent with the literature. The need of ocular surgeries and prevalence of glaucoma seems to correlate with oeJIA.

**Disclosure of Interest**

None declared

### P34 Self-reported physical activity in children and adolescents with juvenile idiopathic arthritis (JIA): correlates and comparison with the general population

#### F. Milatz^1^, S. Hansmann^2^, M. Niewerth^1^, J. Klotsche^1^, J. Hörstermann^1^, J.-P. Haas^3^, D. Windschall^4^, J. Peitz^5^, T. Kallinich^6^, R. Trauzeddel^7^, H. Girschick^8^, K. Minden^1,9^

##### ^1^Epidemiology and Health Care Research, German Rheumatism Research Centre, Berlin; ^2^Center for Pediatric Rheumatology, autoinflammation reference centre Tuebingen (arcT), University Children's Hospital Tuebingen, Tuebingen; ^3^German Center for Paediatric and Adolescent Rheumatology, Garmisch-Partenkirchen; ^4^Clinic for Paediatric and Adolescent Rheumatology, Northwest German Center for Rheumatology, St. Josef-Stift Hospital, Sendenhorst; ^5^Paediatric Rheumatology Centre, Asklepios Clinic, Sankt Augustin; ^6^Department of Pediatric Respiratory Medicine, Immunology and Critical Care Medicine, University Medicine Charité Berlin; ^7^Department of Paediatrics, Helios Klinik Berlin-Buch; ^8^Children's Hospital, Vivantes Hospital im Friedrichshain; ^9^Department of Rheumatology and Clinical Immunology, University Medicine Charité Berlin, Berlin, Germany

###### **Correspondence:** F. Milatz

**Introduction:** Physical activity (PA) is essential throughout growth and maturation to ensure optimal physical function and fitness, especially for those suffering from chronic conditions such as JIA [1].

**Objectives:** This study aimed i) to estimate the proportion of JIA patients meeting the recommended minimum level of PA compared with general population controls and ii) to identify clinical parameters associated with physical (in)activity.

**Methods:** Patients’ (≥12 years) or parents’ (≤11 years) self-reported data on PA were considered from the German Paediatric Rheumatologic Database. In accordance with the methodology used in the general population survey [2], achievement of WHO recommendations on PA of at least 60 minutes per day was determined among 3-17-year-olds. For comparability reasons with the general population, 2017 served as the year for which sex- and age-matched pairs were formed. Multinomial logistic regression was used to analyze the association between physical (in)activity and clinical as well as self-reported outcomes.

**Results:** Data from 6.297 matched-pairs (mean age 11.2 ± 4.2 years, female 67%, patients’ disease duration 4.5 ± 3.7 years, persistent oligoarthritis 43%) were eligible for analysis. Almost 36% of patients aged 3 to 17 years met the recommended PA amount (72% aged 3 to 6; 48% aged 7 to 10; 28% aged 11 to 13; 16% aged 14 to 17). In matched controls, 21% fulfilled the WHO recommendations on PA (42% aged 3 to 6; 25% aged 7 to 10; 17% aged 11 to 13; 10% aged 14 to 17). While no relevant sex differences were found in JIA, considerable variation between categories could be identified (e.g. 42% persistent oligoarthritis vs. 22% enthesitis-related arthritis). Older age (OR = 0.79, 95% CI = 0.78-0.80), longer disease duration (OR = 0.88, 95% CI = 0.86-0.89), higher BMI (OR = 0.96, 95% CI = 0.93-1.00) and more frequent use of biologics (OR = 0.74, 95% CI = 0.65-0.85) were associated with a lower likelihood of achieving the PA recommendations. According to patient-reported data (adolescents ≥12 years), the proportion of physically inactive (PA <2 days/week) was highest (15%, n=493). Among them, female sex (OR = 0.74, 95% CI = 0.60-0.92), age (OR = 1.08, 95% CI = 1.02-1.15), cJadas-10 (OR = 1.08, 95% CI = 1.06-1.10), CHAQ (OR = 2.16, 95% CI = 1.77-2.63), and treatment with glucocorticoids (OR = 3.19, 95% CI = 1.35-7.5) were associated with physical inactivity.

**Conclusion:** Although children and adolescents with JIA meet the WHO recommendation on PA more often than general population controls, a remarkable decline towards physical inactivity is observed with increasing age. Because this cannot be explained by disease- or symptom-related factors alone, overprotective attitudes should be avoided and social, emotional, cultural, and environmental barriers to PA considered.

**References**

[1] Gualano B et al. Physical activity for paediatric rheumatic diseases: standing up against old paradigms. Nat Rev Rheumatol 2017;13:368-379.

[2] Finger JD et al. Körperliche Aktivität von Kindern und Jugendlichen in Deutschland - Querschnittergebnisse aus KiGGS Welle 2 und Trends. Journal of Health Monitoring 2018;3:24-31.

The National Paediatric Rheumatological Database has been funded by AbbVie, Chugai, Novartis and GSK.

**Trial registration identifying number:**

**Disclosure of Interest**

None declared

### P35 PD-1+ CD8+ T lymphocytes as a potential molecular marker of JIA activity – preliminary results

#### V. Opoka-Winiarska^1^, I. Morawska^2^, S. Mertowski^2^, J. Ludian^2^, I. Korona-Głowniak^3^, E. Grywalska^2^, J. Roliński^2^

##### ^1^Department of Paediatric Pulmonology and Rheumatology; ^2^Chair and Department of Clinical Immunology; ^3^Chair and Department of Pharmaceutical Microbiology, Medical University of Lublin, Lublin, Poland

###### **Correspondence:** I. Morawska

**Introduction:** Juvenile idiopathic arthritis (JIA) is a chronic inflammatory disease in which immune-related mechanisms involved in the pathogenesis still remains unclear. Similarly, immunological markers of disease activity require explanation.

**Objectives:** The aim of the study was to assess the expression of programmed-death cell receptor 1 (PD-1) on lymphocytes in patients diagnosed with JIA and determine whether those results correlates with the type of the disease, chosen laboratory parameters, as well as disease activity.

**Methods:** The study included 34 children, 18 patients with newly diagnosed JIA prior to any therapy and 16 healthy volunteers (HV) with a similar age distribution. 9 patients were diagnosed with the enthesitis-related (ERA) JIA type, 7 the oligo- and 2 poly-arthritis. Disease activity was assessed by the Juvenile Arthritis Disease Activity Score 71 (JADAS 71) calculated on the following parameters: parent global assessment of well-being, physician’s global assessment of disease activity, number of active joints and erythrocyte sedimentation rate (ESR).

After obtaining the appropriate consents, blood samples were taken from the patients and HV’s. Samples after preparation according to the protocol were stained using anti-human antibodies (BD Biosciences, USA) and analyzed using FACSCaliburTM flow cytometer (BD Biosciences) and CellQuest Pro Software. Results were statistically analyzed using Statistica 12. Kruskal-Wallis test and Spearman rank correlation were used to determine statistical significance.

**Results:** Data analysis showed no significant differences in the percentages of PD-1^+^CD4^+^, PD-1^+^CD8^+^ and PD-1^+^CD19^+^ cells between the JIA patients and the control group. A higher level of PD-1^+^ lymphocytes was observed in patients with polyarthritis form, but this relationship was not statistically significant. Preliminary results indicated that there was no correlation between the percentage of PD-1 receptor and other laboratory and clinical parameters. However, there was a strong statistically significant correlation (p=0.0057) between the percentage of CD8^+^PD-1^+^ lymphocytes and the disease activity measured by the JADAS 71 (Tab.1.)

**Conclusion:** Our research showed a correlation between the percentage of PD-1+CD8+ T lymphocytes and the disease activity. The search for a molecular markers of the disease activity is extremely important in the process of choosing proper treatment strategies and preventing the disability of children diagnosed with JIA. Flow cytometry is a cheap, quick and clinically available method. The role of the PD-1+CD8+ cells in chronic inflammation is also interesting. More research is needed to assess whether the PD-1+CD8+ lymphocytes may be used as a marker of disease activity or the potential target of therapy.

Bioethics Committee approval number KE-0254/93/2021.

**Disclosure of Interest**

None declared

**Table 1 (abstract P35).** Correlation of % lymphocyte (PD-1^+^CD4^+^, PD-1^+^CD8^+^ and PD-1^+^CD19^+^) and JADAS 71

	Spearman R	t(N-2)	p-value
CD4^+^/PD-1+ and JADAS 71	0.13	0.54	0.60
CD8^+^/PD-1+ and JADAS 71	0.64	3.22	0.0057
CD19^+^/PD-1+ and JADAS 71	0.21	0.83	0.422

### P36 Psychological symptoms during COVID-19 pandemic in a cohort of patients with juvenile idiopathic arthritis

#### C. Traverso, R. Naddei, T. Lastella, F. Aversano, M. Alessio

##### Pediatric Rheumatology Unit, Mother and Child Department, University of Naples Federico II, Naples, Italy

###### **Correspondence:** R. Naddei

**Introduction:** Juvenile idiopathic arthritis (JIA) is the most common pediatric chronic rheumatic disease. Since SARS-CoV-2 outbreak, patients with JIA had to cope with some challenges which may have impacted not only their routine disease management but also their mental health.

**Objectives:** Aim of our study was to evaluate psychological status of children and adolescents affected by JIA during COVID-19 pandemic.

**Methods:** A web-based survey, consisting in two questionnaires, was conducted through the Google-Forms platform. Between November 11 and December 4, 2020, the links to access the online survey pages were sent out to the parents of patients with JIA, aged 7-18 years, followed at the Pediatric Rheumatology Unit of the University of Naples Federico II. The survey included the Children's Depression Inventory (CDI) and the Anxiety Questionnaire for Developmental Age. A CDI score of 19 was used as threshold to discriminate children at risk of depression from nondepressed children. Anxiety questionnaire scores equal or higher than the 80° centile were considered suggestive of anxiety symptoms. Univariate statistical analysis was performed.

**Results:** 83 patients (mean age 12,9 years, SD 3) answered the survey. 54 out of 83 patients were on-medication (65.15%); the most frequent JIA subtype was the oligoarticular one (63.8%), followed by the RF-negative polyarticular (25.3%) and the systemic ones (8.4%). 6 patients scored more or equal to 19 at CDI, resulting in a prevalence of depressive symptoms of 7.2% in our cohort. CDI scores resulted significantly higher in children on medication compared to patients off-therapy (median CDI 7 [IQR 4-9.5] vs 4 [2-7.50], p=0.046) and in females compared to male patients (6 [3-11] vs 4.5 [2-6.75], p=0.046). CDI levels were not associated with JIA subtype, age or presence of uveitis, meanwhile a weak correlation was found between CDI score and disease duration (Spearman's ρ 0.244, p=0.026). Anxiety symptoms were present in 12 out of 83 patients (14.5%). Females presented higher score at the anxiety test compared to males (33 [IQR 26-42) vs 26 [IQR 20.2-33], p=0.019). No association was found between the scores of Anxiety Questionnaire for Developmental Age and patient age, JIA subtype, treatment for JIA, presence of uveitis or disease duration. A moderate correlation was detected between depressive and anxiety symptoms (Spearman's ρ 0.486, p<0.0005).

**Conclusion:** About 7% and 14.5% of our patients with JIA resulted at risk of depression and anxiety, respectively. SARS-CoV-2 pandemic may have impacted their psychological status; therefore, a screening evaluation should be performed in patients with JIA, especially in females on-medication, in order to provide psychological support to children experiencing depressive or anxiety symptoms, which may have been triggered and worsened by COVID-19 pandemic.

**Disclosure of Interest**

None declared

### P37 Lipoma arborescens in childhood: a rare condition mimicking oligoarticular juvenile idiopathic arthritis (JIA) at onset

#### A. Omenetti^1^, B. Lattanzi^1^, V. Galeazzi^2^, M. Marinelli^3^, S. Cazzato^1^

##### ^1^Pediatric Unit, Salesi Children's Hospital; ^2^Clinical Radiology, Departments of Radiologic Science; ^3^Clinical of Adult and Paediatric Orthopedic, AOU Ospedali Riuniti, Ancona, Italy

###### **Correspondence:** A. Omenetti

**Introduction:** Mild painful knee swelling with functional limitation is one of the most common clinical picture at onset of monoarticular juvenile idiopathic arthritis (JIA).

**Objectives:** To present an unusual condition featured by overlapping clinical signs typical of monoarticular JIA, which may need to be reminded during differential diagnosis.

**Methods:** Diagnostic work up including routine and immunological blood test combined to imaging assessments (i.e. knee ultrasound and magnetic resonance imaging, MRI) and synovial biopsy was carried out. Literature revision of similar case reports was performed to confirm significance of the findings.

**Results:** A 12 year-old female presented with left knee swelling, mild pain and functional limitation, persisting for 8 weeks without history of recent trauma nor infections. Morning stiffness was not referred. Physical examination was unremarkable except for local mild painful swelling in the absence of calor, rubor nor significant joint effusion, and associated to functional limitation at squatting. General condition were good except for obesity, in the absence of constitutional symptoms. By investigating her personal and medical history, a similar episode, occurring on the same joint 18 months earlier and resolved following evacuative arthrocentesis, was described. The procedure had been performed in emergency room with no further measures due to referred serosal synovial fluid and unremarkable routine blood tests with negative knee X-ray. Persistent well-being was referred until the ongoing relapse. Given the disease history and the presence of autoimmunity in the family (i.e. psoriasis and autoimmune thyroiditis in father and sibling, respectively), onset of JIA was suspected. Routine tests were normal with negative ERS, CRP and anti-streptococcal titer antibodies. Autoimmune profiling was unremarkable (i.e. negative ENA, dsDNA, rheumatoid factor, anticitrullinated antibodies, and eye examination ruled out signs of uveitis. Knee ultrasound reported a prominent diffuse synovial thickening with mamillated aspects, associated with mild corpuscolated joint effusion, without certain signs of hypervascularization. Pigmented villonodular hyperplasia was thus considered, and bilateral knee MRI performed. Surprisingly, MRI findings were consistent with lipoma arborescens, a benign intra-articular tumor featured by villous synovial hypertrophy and lipomatous infiltration of the subsynovial tissue. Namely, the MRI reported bilateral villous proliferation of the synovia with lipomatous features. Synovial biopsy eventually confirmed the diagnosis of lipoma arborescens and the patient was referred to orthopedics for therapeutical synovectomy. Patient lost at follow up.

**Conclusion:** Lipoma arborescens usually affects the knee (mostly but not exclusively in monoarticular pattern) although every joint can be involved. Although rare in children, we revised the available literature in order to assess the significance of this finding. Actually, to date only 15 cases have been described in pediatric age, affecting one knee (N=9), bilateral knees (N=4), one ankle (N=1) and one knee and one elbow (N=1). Due to the rarity of this condition, delayed diagnosis (in terms of months-years) usually occurred. Interestingly, in at least 3 cases patients had been previously diagnosed with JIA and treated accordingly, mostly for years. In one case the patient also obtained diagnosis and treatment for rheumatic fever, before receiving JIA misdiagnosis. In conclusion, although rare, lipoma arborescens should be considered in differential diagnosis of oligoarticular JIA at onset, in order to avoid misdiagnosis and overtreatment.

**Patient Consent Received**

No

**Disclosure of Interest**

None declared

### P38 Juvenile idiopathic arthritis damage index articular and extraarticular: single-center report

#### S. Asadova^1^, A. Paç Kisaarslan^2^, S. Özdemir Çiçek^3^, N. Şahin^4^, S. N. Taşkın^2^, S. Doğantan^2^, M. H. Poyrazoğlu^2^

##### ^1^Pediatrics; ^2^Pediatric Rheumatology, Erciyes University School of Medicine; ^3^Pediatric Rheumatology, Kayseri City Hospital, Kayseri; ^4^Pediatric Rheumatology, Bursa City Hospital, Bursa, Turkey

###### **Correspondence:** A. Paç Kisaarslan

**Introduction:** After biological treatment options, quality of life and articular functions in patients with Juvenile Idiopathic Arthritis (JIA) have been maintained close to normal. The damages have decreased considerably compared to the past.

**Objectives:** We aimed to evaluate the damage status of the patients with JIA following in our center.

**Methods:** 202 JIA patients who had been followed up for two years or more were included. The data of the patients were collected retrospectively. Demographic data, comorbid diseases, laboratory data (at baseline and during follow-up), disease activity during the follow-up period, and treatments were evaluated. Disease activities, quality of life and Juvenile Arthritis Damage Index articular (JADI-A) and extraarticular (E) were evaluated at the final examination. Factors affecting JADI-A and E were assessed by univariate and multivariate logistic regression analysis.

**Results:** Two hundered two patients with disease duration of more than two years and still being followed up were included in the study. 127 (62.6%) of the patients were female and 75 (36.9%) were male. Their median age was 13 (IQR: 11-16), and age at diagnosis was 7 (IQR: 4-10) years. The median follow-up time was 5 (IQR: 4-8) years. 17(8%) in systemic, 82(40,5%) in oligo, 41(20%) in poly, 54(26%) in enthesitis-related arthritis, 5(2,4%) in psöriatic, 3 (1,5%) in undifferentiated arthritis were involved. Ninety-two (45.3%) patients had comorbid diseases. The median age at diagnosis was 8(IQR: 4-11) years and the follow-up period was 5(IQR: 2-9) years in patients with at least a single comorbid disease. A fifty-four(26.6%) patients had a family history of rheumatologic disease. There was no statistical difference between the disease subgroups in terms of demographic data (p> 0.05).

JADI-A scores were median:0(min-max: 0-24), JADI-E scores were median:0(min-max:0-4) in whole study population. In multivariate analysis, the mean annual attacks number [OR: 1,759 (CI: 1,300-2,379], p: 0,000), mean annual eritrocyte sedimantation rate (ESR) [OR: 1,072 (CI: 1,021-1,125), p: 0.005], duration of metotrexate usage [OR: 1.029 (CI: 1.013-1.046, p: 0.001] and biological drug usage [OR: 5.810 (CI: 1.296-26.054), p: 0.022) were effective on JADI-A scores. The CRP value at the first admission [OR: 1.007 (CI: 1,000-1,014), p: 0.037], the mean annual ESR value [OR: 1,051 (CI: 1,008-1,095), p: 0.019] were found to be effective on the JADI-E scores. The ideal cut-off point of the annual attacks number was detected 1.38 [AUC: 0.734 (0.641-0.828) / p: 0.001], the ideal cut-off point for mean annual ESR was detected 14.32 [AUC: 0.617 (0.514-0.7121) / p: 0.027] affecting JADI-A scores. The ideal cut-off point of the CRP value at the first admission was detected 13,25 [AUC: 0,662 (0,541-0,782) / p: 0,009], the ideal cut-off point for the mean annual ESR value was detected 15,10 [AUC: 0.674(0.567-0.780) / p: 0.002] affecting JADI-E scores.

**Conclusion:** In this study, JIA damages were evaluated after a period of biological treatment usage. Both JADI-A and E scores were very low in our cohort. This study showed that the importance of timely and effective suppression of inflammation. The parameters used in routine clinical practice can help to predict damage.

**Disclosure of Interest**

None declared

### P39 NRF2 regulates redox metabolism of CD4+T cells in chronic inflammatory conditions

#### A. Rajendiran, P. Klemm, K. Tenbrock, K. Ohl

##### Clinic for Pediatrics and Adolescent Medicine, University Hospital RWTH Aachen, Aachen, Germany

###### **Correspondence:** A. Rajendiran

**Introduction:** By entering inflamed tissues, T cells adapt to low levels of oxygen, lack of key nutrients and oxidative stress conditions. To study how this environment affects T cells, we analyzed T cell metabolism and function in synovial fluid cells from Juvenile Idiopathic Arthritis (JIA) patients.

**Objectives:** We aimed to investigate how oxidative stress regulates T cell responses within inflamed joints of JIA patients and analyzed Nrf2-the key regulator of the antioxidative stress response- and it’s signaling pathways.

**Methods:** Flow cytometry analyses were performed to determine oxidative status and metabolic characteristics in the mononuclear cells from arthritic joint and peripheral blood of JIA patients. Seahorse assay were performed to analyze their metabolic activity. qRT-PCR were performed to analyze expression of genes involved in glucose and fatty acid metabolism.

**Results:** We identified high ROS levels in CD4+ T cells from synovial fluid (SF). Nrf2 and its target gene Nqo1 were less expressed in SF compared to blood CD4+ T cells. SF CD4+ T cells expressed high levels of mitochondrial mass, high glucose uptake and ECAR levels and high fatty acid uptake. Vice versa, Nrf2 activation of SF T cells yielded in downregulation of ROS, ECAR and fatty acid uptake and also reduced secretion of IFN-g.

**Conclusion:** These findings suggest that Nrf2 signaling regulates the metabolism of SF T cells and its dysregulation in T cells during chronic inflammation could contribute to disease progression.

**Patient Consent Received**

Yes

**Disclosure of Interest**

None declared

### P40 Impact of concomitant methotrexate use and prior bdmard exposure on tofacitinib efficacy and safety in patients with polyarticular course juvenile idiopathic arthritis: post hoc analysis of a phase 3 withdrawal study

#### N. Ruperto^1^, D. J. Lovell^2^, O. Synoverska^1^, C. Abud Mendoza^1^, A. Spindler^1^, Y. Vyzhga^1^, I. Tirosh^1^, L. Imundo^2^, E. Alexeeva^1^, P. Chiraseveenuprapund^2^, H. Shi^3^, G. Sawyerr^4^, A. Blum^5^, P. Klaus^5^, A. Shapiro^6^, A. Diehl^3^, A. Ebrahim^3^, A. Martini^1^, H. I. Brunner^2^, on behalf of for PRINTO and PRCSG

##### ^1^PRINTO, IRCCS Istituto Giannina Gaslini, Genova, Italy; ^2^PRCSG, Cincinnati Children’s Hospital Medical Center, Cincinnati, OH; ^3^Pfizer Inc, Collegeville, PA; ^4^Pfizer Inc, New York, NY, United States; ^5^Pfizer Pharma GmbH, Berlin, Germany; ^6^Pfizer Inc, Peapack, NJ, United States

###### **Correspondence:** N. Ruperto

**Introduction:** Tofacitinib is an oral JAK inhibitor that is being investigated for several forms of JIA. The efficacy and safety of tofacitinib in patients (pts) with polyarticular course (pc)JIA were demonstrated in a Phase 3 trial.

**Objectives:** To assess tofacitinib efficacy/safety in pts with pcJIA, stratified by concomitant methotrexate (MTX) use and prior exposure to biologic (b)DMARDs.

**Methods:** This post hoc analysis included data from pts with pcJIA aged 2–<18 years in a Phase 3, randomised, double-blind, placebo (PBO)-controlled withdrawal study. Pts received tofacitinib 5 mg BID or body weight-based lower equivalent dose. Pts achieving ≥JIA/ACR30 response at Week (W)18 were randomised 1:1 to continue tofacitinib or switch to PBO to W44. Here, pts were analysed in subgroups: concomitant MTX use on study Day 1 (yes/no) and prior bDMARD exposure (yes/no). Efficacy for tofacitinib vs PBO was assessed to W44, by subgroup: JIA flare rate; JIA/ACR50/70/90 response and JIA/ACR inactive disease (ID) rates; and least squares mean (LSM) change from W18 (∆) in JADAS27-CRP. Safety with tofacitinib was assessed throughout, by subgroup.

**Results:** 184 pts with pcJIA received tofacitinib to W18, when pts were randomised to receive tofacitinib (n=72) or PBO (n=70). Of these pts, 106 received concomitant MTX and 43 had prior bDMARD exposure. Across subgroups, most pts were female and white, and mean age ranged from 10.9–12.9 years. JIA flare rate was lower and JIA/ACR50/70/90 response and JIA/ACR-ID rates were higher with tofacitinib vs PBO at W44 across subgroups (Table). The greatest differences between treatments were in pts who did not use concomitant MTX and those with prior bDMARD exposure. Across subgroups, JADAS27-CRP improved or was stable at W44 vs W18 in pts receiving tofacitinib; scores worsened with PBO, most evidently in pts with prior bDMARD exposure (Table). AEs and serious AEs, respectively, occurred in: 72.9% and 3.0% (+MTX); 88.2% and 3.9% (-MTX); 80.3% and 4.5% (prior bDMARDs); and 75.4% and 2.5% (no prior bDMARDs) of pts. The most common AEs, by system organ class, were infections in all subgroups (43.6–58.8% of pts). AEs of special interest occurred at a low frequency across subgroups; overall, 1.6% had hepatic events, 1.1% had herpes zoster (all events non-serious) and 1.6% had serious infections. No pts died, nor had opportunistic infections (including TB), malignancies, macrophage activation syndrome, MACE, GI perforations, interstitial lung disease or thrombotic events.

**Conclusion:** In pts with pcJIA, tofacitinib was efficacious irrespective of concomitant MTX use or prior bDMARD exposure. Tofacitinib safety in all subgroups was generally consistent with the overall Phase 3 study population. The analysis is limited by the small sample size in the subgroups.

**Trial registration identifying number:** ClinicalTrials.gov (NCT02592434)

**Patient Consent Received**

No

**Disclosure of Interest**

N. Ruperto Consultant for: Ablynx, AstraZeneca/MedImmune, Biogen, BMS, Boehringer Ingelheim, Eli Lilly, EMD Serono, F. Hoffmann-La Roche, GSK, Janssen, Merck Sharp & Dohme, Novartis, Pfizer Inc, R-Pharm, Sanofi, Servier, Sinergie and Sobi, Speaker Bureau of: Ablynx, AstraZeneca/MedImmune, Biogen, BMS, Boehringer Ingelheim, Eli Lilly, EMD Serono, F. Hoffmann-La Roche, GSK, Janssen, Merck Sharp & Dohme, Novartis, Pfizer Inc, R-Pharm, Sanofi, Servier, Sinergie and Sobi, D. Lovell Consultant for: AstraZeneca, Boehringer Ingelheim, GSK, Novartis, Pfizer Inc, Roche, Takeda and UBC, and DSMB chairperson for Forest Research and NIH, O. Synoverska Speaker Bureau of: Alpen Pharma AG, Nestlé, Sanofi and SPERCO, C. Abud Mendoza: None declared, A. Spindler Speaker Bureau of: Eli Lilly, Y. Vyzhga: None declared, I. Tirosh: None declared, L. Imundo: None declared, E. Alexeeva: None declared, P. Chiraseveenuprapund Consultant for: CARRA and Novartis, H. Shi Shareholder of: Pfizer Inc, Employee of: Pfizer Inc, G. Sawyerr Consultant for: Pfizer Inc, Employee of: Syneos Health Inc, A. Blum Shareholder of: Pfizer Pharma GmbH, Employee of: Pfizer Pharma GmbH, P. Klaus Shareholder of: Pfizer Pharma GmbH, Employee of: Pfizer Pharma GmbH, A. Shapiro Shareholder of: Pfizer Inc, Employee of: Pfizer Inc, A. Diehl Shareholder of: Pfizer Inc, Employee of: Pfizer Inc, A. Ebrahim Shareholder of: Pfizer Inc, Employee of: Pfizer Inc, A. Martini Consultant for: Aurinia, BMS, Eli Lilly, EMD Serono, Janssen and Pfizer Inc, H. Brunner Consultant for: AbbVie, AstraZeneca/MedImmune, Bayer, Biocon, BMS, Boehringer Ingelheim, Eli Lilly, Janssen, Novartis, Pfizer Inc, Roche and R-Pharm, Employee of: Cincinnati Children’s Hospital Medical Center, Speaker Bureau of: GSK, Novartis and Roche

**Table 1 (abstract P40).** Efficacy in pts with pcJIA at W44

	Tofacitinib		PBO		Tofacitinib		PBO	
Rate, %	+MTX N=52	-MTX N=20	+MTX N=54	-MTX N=16	Prior bDMARDs N=23	No prior bDMARDs N=49	Prior bDMARDs N=20	No prior bDMARDs N=50
JIA flare	28.9	30.0	48.2	68.8	26.1	30.6	70.0	46.0
JIA/ACR50^a^	65.4	70.0	51.9	31.3	73.9	63.3	30.0	54.0
JIA/ACR70^a^	53.9	55.0	40.7	25.0	47.8	57.1	25.0	42.0
JIA/ACR90^a^	36.5	30.0	24.1	12.5	26.1	38.8	10.0	26.0
JIA/ACR-ID	25.0	30.0	18.5	12.5	17.4	30.6	5.0	22.0
∆JADAS27-CRP, LSM (N)	0.9 (36)	-2.4 (13)	3.6 (27)	2.3 (5)	-1.1 (16)	0.8 (33)	12.1 (6)	4.4 (26)

^a^Relative to study Day 1

### P41 Review of the worldwide epidemiological data of juvenile idiopathic arthritis

#### V. Sevostyanov^1^, I. Razumov^1^, E. Zholobova^2^

##### ^1^I.M. Sechenov First Moscow State Medical University (Sechenov University); ^2^I.M. Sechenov First Moscow State Medical University, Moscow, Russian Federation

###### **Correspondence:** V. Sevostyanov

**Introduction:** Juvenile idiopathic arthritis (JIA) is a chronic, systemic autoimmune disorder that is characterized by joint inflammation of unclear etiology. The study of the epidemiology of JIA is one of the important areas of pediatrics, knowledge of the prevalence allows planning the necessary resources for the diagnosis and treatment of patient.

**Objectives:** A Review of new epidemiological data of the worldwide incidence and prevalence of rheumatic diseases in children.

**Methods:** Studies searched in the electronic database PubMed.

**Results:** 125 studies were screened, 11 epidemiological studies of JIA since 2010 were undertaken, 11 countries included in the review. Prevalence. Europe: Germany - prevalence was 73.4 to 101.5; The Russian Federation - 65.7; Spain - prevalence was 39.7; France - prevalence was 15.7. Asia: Bangladesh - prevalence was 60.5, India - prevalence was 48, Singapore - prevalence 19.2. Africa: Egypt - prevalence was 3.43. South America: Brazil - prevalence was 196. North America: USA - prevalence was 44.7 per 100,000 children. Incident. Europe: Germany - incidence was 16.6, Sweden - incidence was 12.8, Spain - incidence was 6.9. North America: USA - incidence was 11.9 per 100,000 children.

**Conclusion:** An analysis was made of articles on morbidity. Data is heterogeneous across countries, requiring further in-depth study. There was a lack of updated data on the incidence of JIA worldwide.

**Patient Consent Received**

No

**Disclosure of Interest**

None declared

### P42 factors that cause ineffective supplementation of vitamin D in patients with JIA

#### N. S. Shevchenko^1,2^, I. Khadzhynova^1,2^, L. Bohmat^1,2^

##### ^1^Department of pediatrics № 2, V. N. Karazin Kharkiv National University; ^2^Department of rheumatology and comorbid states, SI Institute for Children and Adolescents Health Care of NAMS of Ukraine, Kharkiv, Ukraine

###### **Correspondence:** N. S. Shevchenko

**Introduction:** Modern publications discuss the role that vitamin D plays in the onset, development and course of rheumatic diseases. The question of additional supplementation with calciferol and dosage in persons with chronic inflammatory diseases remains open.

**Objectives: T**o determinate the tendency of changes of VD status in children with juvenal idiopathic arthritis JIA after its 3-months supplementation based on characteristics of JIA and methotrexate (MTX) therapy.

**Methods:** 40 children with JIA (23 females, 10 males) were included to the study. The average age of patients was 10.8±4.6 years. The results were analyzed depending on physical development of children, taking into account body mass index (BMI), variant of the disease (oligoarthritis (n=17), polyarthritis (n=13), undifferentiated (n=10) arthritis), disease activity on a scale JADAS-27 and mode of application of basic therapy (the presence of methotrexate or its absence, duration, doses). The study was conducted twice: the first one- in the absence of additional intake of vitamin D, the second one - after its 3 months supplementation in a dose of 2000 IU. Serum 25-hydroxyvitamin D [25(OH)D] levels were measured using chemiluminescent method (Cobas 6000, Roche Diagnostics, Switzerland).

**Results:** The average primary level of vitamin D was 21,40±1,97 ng/ml. The re-examination found 28,89±2,11 ng/ml of vitamin D (p<0,05). Positive dynamic was observed, but the optimal level of VD was not reached by patients. Analysis of the changes of vitamin D level in the blood showed the following regularities (Table). Its increase was not determined in overweight children. The oligoarticular variant of JIA was more favorable with respect to both the initial and the repeated level of vitamin D. Children with achieved remission of the disease and a low degree of JIA activity did not show a significant increase in the level of vitamin D. There was no positive dynamics of vitamin D status in children who received MTX monotherapy for more than six months at a MTX dose of less than 10 mg / m^2^.

**Conclusion:** Children with JIA have an impaired vitamin D status, its decrease. The effectiveness of additional supplementation with cholecalciferol is higher in children with low and normal body weight, with oligoarticular JIA. In order to achieve an optimal response to additional intake of vitamin D, its early prescription is necessary, especially against the background of high JIA activity. Rational prescription of basic therapy and its intensification after 6 months of use is one of the links in the prevention of vitamin D deficiency in children with JIA.

**Disclosure of Interest**

None declared

**Table 1 (abstract P42).** Dynamics of vitamin D levels in children with JIA after its 3-months supplementation М±m, ng/ml

Sings	vitamin D level		Significance of differences
	before additional intake of vitamin D	after taking of vitamin D	
general group	21,40±1,97	28,89±2,11	**p<0,05**
normal BMI	22,57±3,94	27,08±5,47	**p<0,05**
decreased BMI	17,69±3,15	30,01±6,26	**p<0,05**
increased BMI	25,02±5,73	25,72±4,74	p>0,05
oligoarthritis	24,69±1,89	31,42±2,30	**р<0,05**
polyarthritis	18,03±2,25	24,21±2,74	р>0,05
undifferentiated arthritis	22,24±2,46	25,32±3,01	р>0,05
inactive disease	22,56±4,95	24,57±5,87	p>0,05
moderate activity	25,38±3,78	25,56±3,79	p>0,05
average activity	25,45±3,89	30,35±4,78	**p<0,05**
high activity	22,49±3,45	27,61±4,12	**p<0,05**
MTX +	21,53±7,16	29,03±9,99	**p<0,05**
MTX -	22,67±2,86	23,03±3,42	p>0,05
Duration of MTX therapy:			
before 6 months	22,98±2,71	31,86±6,28	**p<0,05**
7-12 months	21,27±3,24	28,82±8,34	p>0,05
1-2 years	20,50±3,03	25,16±10,40	p>0,05
3-5 years	20,87±2,71	21,53±3,74	p>0,05
Doses of MTX:			
less than 10 mg/m2	22,37±2,67	22,99±2,45	p>0,05
10,1-12,4 mg/m2	21,09±2,78	32,22±3,74	**p<0,05**
12,5-15 mg/m2	22,31±2,36	30,71±3,46	**p<0,05**

### P43 Trajectories of response to etanercept identified in four UK juvenile idiopathic arthritis cohorts

#### S. J. W. Shoop-Worrall^1,2^, K. L. Hyrich^1,3^, L. R. Wedderburn^4,5,6^, W. Thomson^3,7^, N. Geifman^2^, on behalf of BSPAR-ETN Study, BCRD Study, CAPS, CHARMS, CLUSTER

##### ^1^Centre for Epidemiology Versus Arthritis; ^2^Centre for Health Informatics, UNIVERSITY OF MANCHESTER; ^3^NIHR Manchester BRC, Manchester University NHS Foundation Trust, Manchester Academic Health Science Centre, Manchester; ^4^Centre for Adolescent Rheumatology Versus Arthritis, GOS Institute of Child Health, University College London; ^5^Paediatric Rheumatology, Great Ormond Street Hospital NHS Foundation Trust; ^6^NIHR Great Ormond Street Hospital Biomedical Research Centre, London; ^7^Centre for Genetics and Genomics Versus Arthritis, UNIVERSITY OF MANCHESTER, Manchester, United Kingdom

###### **Correspondence:** S. J. W. Shoop-Worrall

**Introduction:** In children and young people (CYP) with JIA, we have previously identified clusters with different patterns of disease impact following methotrexate (MTX) initiation. It is unclear whether clusters of treatment response following etanercept (ETN) therapy exist and whether, in a group of CYP who have responded inadequately to or had adverse events on methotrexate, similar treatment response patterns exist. Novel response patterns would aid stratified treatment approaches through better understanding and potential forecasting of more specific response patterns across multiple domains of disease.

**Objectives:** To identify and characterise trajectories of juvenile arthritis disease activity score (JADAS) components following ETN initiation for JIA.

**Methods:** ETN-naïve CYP with non-systemic JIA were selected if enrolled prior to January 2019 in at least one of four CLUSTER consortium studies: the UK BSPAR Etanercept Register, the Biologics for Children with Rheumatic Diseases Study, the Childhood Arthritis Prospective Study and the Childhood Arthritis Response to Medication Study at point of starting ETN as their first biological therapy. JADAS components (active joint count, physician’s global assessment (0-100mm), parental global evaluation (0-100mm) and ESR (mm/hr) were collected at ETN initiation and during the following year.

Multivariate group-based trajectory models, that identify clusters of CYP with similar patterns of change over time, were used to explore ETN response clusters across the different JADAS components. Censored-normal (global scores, ESR) and zero-inflated Poisson (active joint count) models were used, adjusting for year of ETN initiation. Optimal models were selected based on a combination of model fit (BIC), parsimony, and clinical plausibility.

**Results:** Of the 1003 CYP included, the majority were female (70%) and of white ethnicity (90%), with rheumatoid factor-negative JIA the most common disease category (39%).

The optimal model identified five trajectory clusters of disease activity following initiation of ETN. Clusters following ETN were similar and covered similar proportions of CYP to those previously identified following MTX: Fast (Group 1: 13%) and Slow (Group 2: 10%) response, active joint count improves but either physician (Group 3: 6%) or parent global scores (Group 4: 34%) remain persistently raised and a group with persistent raised scores across all JADAS components (Group 5: 36%). Compared to the persistent disease cluster, those with greater improvement had lower age and higher functional ability at ETN initiation and those with persistent raised parent global scores had lower ESR levels and were less likely to be RF-positive at ETN initiation.

**Conclusion:** This study has identified that within CYP initiating ETN, similar response clusters are evident to those previously identified following MTX. This commonality suggests a new framework for understanding treatment response, beyond a simple responder/non-responder analysis at a set point, which applies across multiple drugs despite different mechanisms of action and previous unfavourable treatment outcomes. Understanding both clinical factors associated with, and biological mechanisms underpinning, these clusters would aid stratified medicine in JIA.

**Patient Consent Received**

Yes

**Disclosure of Interest**

None declared

### P44 Treatment to target reduced pain significantly in children with juvenile idiopathic arthritis participating in the best for kids study

#### K. Spekking^1^, P. de Boer^1^, S. A. Bergstra^2^, J. M. van den Berg^3^, D. Schonenberg-Meinema^3^, L. W. van Suijlekom-Smit^4^, M. A. van Rossum^5,6^, Y. Koopman-Keemink^7^, R. ten Cate^1^, C. F. Allaart^2^, D. M. Brinkman^1^, P. C. E. Hissink Muller^1^

##### ^1^Department of Pediatrics, division of Pediatric Rheumatology, Willem Alexander Children’s Hospital, Leiden University Medical Center; ^2^Department of Rheumatology, Leiden University Medical Center, Leiden; ^3^Department of Pediatric Immunology, Rheumatology and Infectious Diseases, Emma Children’s Hospital, Amsterdam University Medical Center, Amsterdam; ^4^Department of Pediatrics/Pediatric Rheumatology, Sophia Children's Hospital Erasmus Medical Center, Rotterdam; ^5^Department of Pediatrics, Emma Children's Hospital, Amsterdam University Medical Centers; ^6^Department of Pediatric Rheumatology, Amsterdam Rheumatology and Immunology Center | Reade, Amsterdam; ^7^Department of Pediatrics, Hagaziekenhuis Juliana Children’s Hospital, The Hague, Netherlands

###### **Correspondence:** K. Spekking

**Introduction:** In Juvenile Idiopathic Arthritis (JIA), a treat-to-target (T2T) strategy is recommended to improve clinical outcomes and was proven effective in suppression of disease activity in the BeSt for Kids study. Does this approach also help to reduce pain in children with JIA?..

**Objectives:** To compare pain in three T2T strategies in JIA patients participating in the BeSt for Kids study and to identify baseline characteristics predicting high pain levels during follow up.

**Methods:** DMARD naïve children who participated in the BeSt for kids study with oligoarticular JIA, RF-negative polyarticular JIA and juvenile psoriatic arthritis were treated with a T2T strategy aiming at (drug free) inactive disease in 1 of 3 initial treatment groups;

1. Initial sequential DMARD monotherapy (Methotrexate (MTX) or Sulphasalazine)
2. Initial MTX with 6 weeks of prednisolone bridging
3. Initial MTX with etanercept.

Pain intensity was measured using a 100 mm Visual Analogue Scale during 24 months of follow-up with 3-monthly intervals. Potential differences in VAS pain scores over time between treatment arms were compared using linear mixed models. A similar multivariable mixed model was used to assess the ability of several baseline characteristics to predict high pain levels during follow-up and to determine the effect of inactive disease on pain.

**Results:**

92 patients were randomized.

Overall, pain scores over time reduced from mean 55.3 (SD 21.7) mm at baseline to 19.5 (SD 25.3) mm after 24 months. When comparing pain over time per arm, pain scores decreased significantly β -1.37 (95% CI -1.73; -1.02). No significant difference was found in pain over time between initial treatment groups. Correction for sex and symptom duration as possible confounders yielded similar results. Inactive disease contributed to pain reduction by -11.36 mm (95% CI -13.80; -8.93). However, 7 children still experienced pain during inactive disease.

Several baseline characteristics demonstrated a significant predictive value for pain over time when tested in a multivariable model. A higher baseline VAS pain and number of active joints at baseline were predictive of higher pain over time. VAS of the patient/parent, symptom duration and NSAID use were not predictive for pain over time.

**Conclusion:** In children with JIA participating in the BeSt for Kids study treatment to target (drug free) remission is effective in reducing pain irrespective of initial treatment. However, some children still experience pain despite reaching inactive disease. This emphasizes the necessity of patient related outcomes for targeted treatment. High VAS pain and many active joints at baseline can help to identify non-systemic JIA patients with a high risk of pain over time despite applying a treat-to-target strategy.

**Trial registration identifying number:** Trial NL1504 (NTR1574)

**Disclosure of Interest**

None declared

**Table 1 (abstract P44).** Baseline Characteristics

	β	p-value	95% Confidence Interval
**VAS Pain**	0.45	0.000	0.25 to 0.65
**VAS Physician**	-0.31	0.014	-0.55 to -0.06
**VAS Patient/parent**	-0.02	0.873	-0.21 to 0.18
**No. of active joints**	0.77	0.009	0.19 to 1.34
PhS*	-0.42	0.008	-0.72 to -0.11
PsS**	-0.42	0.022	-0.77 to -0.06
**Symptom duration (mo.)**	8.09	0.118	-2.08 to 18.26
**NSAID use**	-1.30	0.791	-10.91 to 8.31

VAS Pain, VAS physician and VAS patient/parent was measures on a 100mm scale

*PhS= Physical Summary Score of the Child Health Questionnaire Parent form (CHQ-PF50) (scale 0-100)

**PsS= Psychosocial Summary Score of the CHQ-PF50 (scale 0-100)

### P45 Evaluation of factors for predicting risk of uveitis in juvenile idiopathic arthritis

#### N. Tekgoz, E. Celikel, F. Aydın, Z. Tekin, T. Kurt, M. Sezer, B. Acar

##### Department of Pediatric Rheumatology, University of Health Science, Ankara City Hospital, Ankara, Turkey

###### **Correspondence:** N. Tekgoz

**Introduction:** Juvenile idiopathic arthritis (JIA) is the most common chronic inflammatory arthritis in childhood and causes much disability. Uveitis developed during the disease course due to uveal inflammation. The most common form of JIA-U is chronic anterior uveitis which has frequently seen in the oligoarticular subtype. It often has a silent and insidious onset, which results in severe ocular complications. Therefore screening becomes more critical in JIA patients. It is very difficult to distinguish the treatment of JIA from JIA-U.

**Objectives:** The aim of this study is to evaluate the risk factors that play a role in the development and recurrence of uveitis and determine the relationship between arthritis and uveitis activity.

**Methods:** Patients who were diagnosed juvenile idiopathic arthritis (JIA) with and without uveitis between April 2005 and May 2020, were retrospectively reviewed. The Juvenile Arthritis Disease Activity Score (JADAS-27) was used to evaluate the disease activity. JADAS-27 was calculated separately at the onset of arthritis and uveitis.

**Results:** Uveitis developed in 26 (13.3%) of 195 JIA patients. Of 26 JIA-U patients, 19 (73%) had oligoarticular subtype. The age at the onset of JIA was lower in patients with uveitis than those without uveitis (*p*=0.015). MTX and biological DMARDs treatments were significantly higher in patients with JIA-U than JIA without uveitis (*p*<0.001, *p*=0.038). Oligoarticular JIA was found to be associated with recurrence of uveitis (*p*=0.021). Patients with the recurrent course had a significantly earlier onset of arthritis and uveitis than the none-recurrent group (*p*=0.041, *p*=0.002, respectively). The median JADAS27 score at the onset of uveitis was lower in the recurrent group (*p*=0.038). MTX treatment's median duration and the median time interval between MTX and biologic DMARDs were significantly longer in the recurrent group (*p*=0.013, *p*=0.045).

**Conclusion:** In conclusion, “early age” is a significant risk factor for developing and recurrence of uveitis. It is important to keep in mind patients with low disease activity may also develop uveitis. Therefore, treatment and follow-up should be planned with a multi-disciplinary approach.

**Patient Consent Received**

No

**Disclosure of Interest**

None declared

### P46 Children with juvenile idiopathic arthritis have alterations in B and T follicular cell subsets in peripheral blood

#### C. Tomé^1^, F. Oliveira-Ramos^1,2^, R. Campanilho-Marques^1,2^, A. F. Mourão^3^, S. Sousa^4^, A. P. Martins^5^, P. C. Reis^1,6^, A. T. Melo^1,2^, R. L. Teixeira^1,2^, M. Gonçalves^5^, M. J. Santos^1,4^, L. Graca^1,7^, J. E. Fonseca^1,2^, R. A. Moura^1^

##### ^1^Instituto Medicina Molecular João Lobo Antunes, Faculdade de Medicina, Universidade de Lisboa; ^2^Rheumatology Department, Hospital de Santa Maria; ^3^Rheumatology Department, Centro Hospitalar Lisboa Ocidental, Lisbon; ^4^Rheumatology Department, Hospital Garcia de Orta, Almada; ^5^Pediatric Surgery Department; ^6^Pediatric Department, Hospital de Santa Maria, Lisbon; ^7^Instituto Gulbenkian de Ciência, Oeiras, Portugal

###### **Correspondence:** C. Tomé

**Introduction:** Juvenile idiopathic arthritis (JIA) is the most common rheumatic disease in children. Our group has recently demonstrated that extended oligoarticular (eoJIA) and polyarticular JIA (pJIA) mostly evolve to a rheumatoid arthritis (RA) like phenotype in adulthood. Disturbances in B cells, T follicular helper (Tfh) and T follicular regulatory (Tfr) cell immune responses are associated with the pathogenesis of RA, but their exact role in JIA development is not entirely known.

**Objectives:** The main goal of this study was to characterize the frequency and phenotype of B, Tfh and Tfr cells in peripheral blood of children with eoJIA and pJIA when compared to healthy controls and children with persistent oligoarticular JIA (poJIA).

**Methods:** Blood samples were collected from children with eoJIA (n=5), pJIA (n=11) and poJIA (n=19) treated with disease modifying anti-rheumatic drugs. A group of age-matched healthy children (n=8) was used as control. Peripheral blood mononuclear cells were isolated and the frequency and phenotype of B, Tfh and Tfr cells were evaluated by flow cytometry.

**Results:** The frequency of B, Tfh and Tfr cells was similar between JIA patients and controls. Children with eoJIA and pJIA, but not poJIA, had higher levels of naïve B cells and lower frequencies of post-switch memory B cells and plasmablasts when compared to controls. Th17-like Tfh cells were significantly increased in all JIA patients when compared to controls. B cell phenotype was similar between JIA patients and controls, but a reduced activated phenotype of Tfh cells was observed in JIA patients in comparison to controls.

**Conclusion:** Changes in B and Tfh cell subpopulations, but not in Tfr cells, were found in peripheral blood of children with JIA when compared to controls. The increased frequencies of Th17-like Tfh cells detected in JIA when compared to controls suggests a potential role of these cells in JIA pathogenesis. A treatment effect on the activation state of B, Tfh and Tfr cells cannot be excluded.

**Patient Consent Received**

No

**Disclosure of Interest**

None declared

### P47 Duration of remission in non-systemic juvenile idiopathic arthritis after termination of tumor necrosis factor inhibitors

#### I. Tsulukiya^1^, E. Alexeeva^1,2^, T. Dvoryakovskaya^1,2^, R. Denisova^1^, K. Isaeva^1^, O. Lomakina^1^, A. Mamutova^1^, A. Chomakhidze^1^, A. Fetisova^1^, M. Gautier^1^, I. Kriulin^1,2^, E. Krekhova^1,2^

##### ^1^Rheumatology, National Medical Research Center of Children's Health; ^2^Pediatric, Sechenov First Moscow State Medical University, Moscow, Russian Federation

###### **Correspondence:** E. Alexeeva

**Introduction:** Currently, little is known about when or how to stop tumor necrosis factor inhibitors (TNFi) in non-systemic juvenile idiopathic arthritis (JIA) when a good clinical response is achieved.

**Objectives:** To evaluate the rate of flare during the first 24 months following TNFi withdrawal in patients with non-systemic JIA who have achieved remission while taking the medication.

**Methods:** 85 patients (27—male, 58—female) with JIA and a mean age at diagnosis of 4 years (range 1–18 years) were analyzed retrospectively.

All of them had been receiving TNFi for more than 24 months and discontinued TNFi due to a long-term remission on treatment. Inactive disease was defined according to the criteria of Wallace et al. [1].

**Results:** The clinical subtypes of JIA were persistent oligoarthritis – 39 (46%), RF-negative polyarthritis– 34 (40%), extended oligoarthritis—9 (10,5%), enthesitis-related arthritis—3 (3,5%). 22 (26%) patients have been diagnosed with JIA-associated uveitis.

TNFi were discontinued after 46 (range 10–114) months after initiation of therapy. Duration of remission prior to discontinuing TNFi was 41 (range 6 – 121) months.

In 60 (71%) patients TNFi had been withdrawn after long-term remission was achieved, in 17 (20%) patients as a result of side effects, in 4 (4,5%) patients had been discontinued because of organization problem, and in 4 (4,5%) patients had been stopped biologic therapy by parents. All of them had remission prior to discontinuing TNFi more than 24 months.

The mean duration of remission after TNFi discontinuation was 29 (range 1–92) months.

14 (16%) of patients had flares after less than 6 months after discontinuing of TNFi, 33 (39%) had flares after 6 – 24 months, 38 (45%) had not flares and had remission due to 24 months after discontinuation TNFi.

In 47/85 (55%) patients flares had been appeared, 37/47 (79%) of patients flared with active arthritis, while 10/47 (21%) flared with uveitis.

Disease was successfully controlled in 7/47 (15%) patients with non-biological DMARDs, 40/47 (89%) patients restarted TNFi after flares, due to lack of improvement after non-biological DMARDs.

**Conclusion:** The mean duration of TNFi therapy was 46 months, duration of remission prior to discontinuing TNFi was 41 months. The mean time of remission after discontinuation of TNFi was 29 months, although it should be noted that in as many as 38 children (45 %), flares occurred in more than 24 months.

Data from our experience with TNFi in the treatment of JIA suggest that 45% of patients can be successfully withdrawn from TNFi for at least 24 months.

**Disclosure of Interest**

None declared

### P48 No significantly different effect of etanercept and adalimumab on well-being in non-systemic JIA

#### J. W. van Straalen^1^, S. de Roock^1^, G. Giancane^2,3^, M. Rygg^4^, E. B. Nordal^5,6^, N. Rubio-Perez^7^, M. Jelusic^8^, J. de Inocencio^9^, J. Vojinovic^10^, N. M. Wulffraat^1^, P. C. J. L. Bruijning-Verhagen^11^, J. F. Swart^1^, N. Ruperto^2^, on behalf of Paediatric Rheumatology International Trials Organisation (PRINTO)

##### ^1^Department of Pediatric Immunology and Rheumatology, Wilhelmina Children's Hospital, Utrecht, Netherlands; ^2^Clinica Pediatrica e Reumatologia, IRCCS Istituto Giannina Gaslini; ^3^Dipartimento di Neuroscienze, Riabilitazione, Oftalmologia, Genetica e Scienze Materno-Infantili (DiNOGMI), Università degli Studi di Genova, Genoa, Italy; ^4^Department of Clinical and Molecular Medicine, Faculty of Medicine and Health Sciences, NTNU - Norwegian University of Science and Technology; ^5^Department of Pediatrics, University Hospital of North Norway; ^6^Department of Clinical Medicine, UiT the Arctic University of Norway, Tromsø, Norway; ^7^Departamento de Pediatria, Hospital Universitario "Dr. J. E. González", Facultad de Medicina, Universidad Autónoma de Nuevo León, Monterrey, Mexico; ^8^Division of Rheumatology and Immunology, University School of Medicine, University Hospital Centre Zagreb, Zagreb, Croatia; ^9^Department of Pediatric Rheumatology, 12 de Octubre University Hospital, Madrid, Spain; ^10^Department of Pediatric Immunology and Rheumatology, Faculty of Medicine, University of Nis, Nis, Serbia; ^11^Julius Centre for Health Sciences and Primary Care, University Medical Centre Utrecht, Utrecht, Netherlands

###### **Correspondence:** J. W. van Straalen

**Introduction:** Current treatment guidelines consider adalimumab (ADA) and etanercept (ETN) equally effective in treating arthritis in JIA (1) and in practice the choice between the two drugs is based on patient and physician preferences (2).

**Objectives:** To compare the effects of ADA and ETN on well-being in non-systemic JIA.

**Methods:** Biologic therapy naïve non-systemic JIA patients without a history of uveitis were selected from the international observational Pharmachild registry. Patients who started ETN were matched 1:1 to patients who started ADA based on propensity score, i.e. the probability of receiving one of the two drugs. Outcomes were collected around therapy initiation and 3-12 months afterwards. Primary outcome at follow-up was the change in VAS well-being score from the Juvenile Arthritis Multidimensional Assessment Report (JAMAR). Secondary outcomes were the change in active joint count, number of adverse events and uveitis events. Outcomes were analyzed using linear and logistic mixed model analyses.

**Results:** 46 ADA and ETN starters were matched (Table 1). The estimated change in VAS well-being was lower for ADA compared to ETN, but not clinically and statistically significant (-0.81, 95% CI: -1.72 – 0.08). Similarly, no significant differences were observed for the change in active joint count and number of adverse events. One event of uveitis occurred in the ETN group.

**Conclusion:** No clinically and statistically significant different effect of ADA and ETN on well-being was observed in non-systemic JIA patients.

**References**

1. Beukelman T, Patkar NM, Saag KG, Tolleson-Rinehart S, Cron RQ, DeWitt EM, et al. 2011 American College of Rheumatology recommendations for the treatment of juvenile idiopathic arthritis: initiation and safety monitoring of therapeutic agents for the treatment of arthritis and systemic features. Arthritis Care Res (Hoboken). 2011 Apr;63(4):465–82.

2. Anink J, Otten MH, Gorter SL, Prince FHM, van Rossum MAJ, van den Berg JM, et al. Treatment choices of paediatric rheumatologists for juvenile idiopathic arthritis: etanercept or adalimumab? Rheumatology. 2013 Sep 1;52(9):1674–9.

**Patient Consent Received**

No

**Disclosure of Interest**

None declared

**Table 1 (abstract P48).** Outcomes at follow-up

	ADA starters (n = 46)	ETN starters (n = 46)	Effect estimate for ADA vs. ETN (95% CI)
Decrease in VAS well-being compared to baseline, median (IQR)	2.0 (0.0 – 4.0)	2.0 (0.1 – 5.0)	-0.81 (-1.72 – 0.08)^a^
Decrease in active joint count compared to baseline, median (IQR)	2.5 (1.0 – 4.0)	2.0 (1.0 – 6.0) n = 45	0.44 (-0.22 – 1.10)^a^
Adverse events, n (%)	15 (34.1%) n = 44	13 (28.3%)	1.82 (0.59 – 5.60)^b^
Uveitis events, n (%)	0 (0.0%)	1 (2.2%)	-

^a^average difference, ^b^odds ratio

### P49 Clinico-epidemiological profile of juvenile idiopathic arthritis (JIA) from a single centre in North-West India

#### S. Verma, A. Rafi, A. Sharma

##### Pediatrics, Dr Rajendra Prasad Govt .Medical College,Tanda, Kangra, HP,India-176001, Kangra, India

###### **Correspondence:** S. Verma

**Introduction:** Juvenile idiopathic arthritis is the most common chronic arthritis affecting the children leading to long-term disability. Its incidence and prevalence varies according to ethnicity and environmental influences and each sub type of JIA has distinct age of presentation and gender predilection.

**Objectives:** To evaluate the clinical, laboratory and treatment profile of children with JIA.

**Methods:** This retrospective study was done at a tertiary-care centre in North-India in a government run, tertiary-care medical institute. Demographic data, clinical manifestations, laboratory findings and treatment of patients registered in Pediatric Rheumatology Clinic (PRC) of the institute over last three and half years was analysed. JIA was classified according to the ILAR criteria.

**Results:** We analysed the records of 191 children who were enrolled in PRC, 96(50%) had joint involvement. JIA was the most common rheumatological disorder observed in 61(63.5%) out of 96 children who had arthritis as their predominant presenting manifestation. Thirty two (52.5%) were boys with M:F ratio of 1.1:1. In twenty five (42%) children oligoarthritis was identified, making it the most common subtype of JIA. Enthesitis related arthritis (ERA) was found in 16(26%) patients, predominantly in boys with M:F ratio of 5:1. Eight (13%) and 11(18%) children had systemic JIA (sJIA) and polyarthritis JIA with M:F ratios being 1:1.6 and 1:4.5 respectively. In polyarthritis more children were RF positive 6(55%). Only one case, 15 years old male adolescent had psoriatic arthritis with history of psoriasis in mother. Mean age at disease onset was 7.78 years, 4.75 years, 14.8 years and 14.4 years in oJIA, sJIA, polyarthritis and ERA, respectively. Minimum age of presentation was 13 months, a male child diagnosed with sJIA. Knee joint was the most common joint involved with bilateral involvement in polyarthritis and unilateral in rest of JIA. Hip joint, 6(37.5%) and axial skeleton, 1(6.25%) involvement was found exclusively in ERA while small joints of hands, were involved in all the patients of RF positive polyarthritis 6(100%). None of the patients had temporomandibular joint and cervical spine involvement. Five (62.5%) out of 8 patients of sJIA developed macrophage activation syndrome (MAS) with 50% mortality, may be due to late presentation to our institute. Antinuclear antibodies (ANA) was found positive in 65% cases of polyarthritis and 56% cases of oJIA while 94% (15 out of 16) cases of ERA had HLA B-27 positive. Only 2(3.3%) patients of oJIA had uveitis, both were ANA positive. After enrolment, 75% children were given nonsteroidal anti-inflammatory drugs (NSAIDs). Forty percent of sJIA patients required methylprednisolone pulse therapy and intravenous immunoglobulin (IVIg) for the management of MAS. Intra-articular corticosteroid injections (IACI) were used in 5(20%) patients of oligoarthritis and 4(25%) patients of ERA to alleviate inflammatory symptoms. Oral corticosteroids was given in 4(50%), 5(20%) and 4(25%) patients of sJIA, oJIA and ERA to relieve extra-articular manifestations in sJIA and to bridge the effect of disease modifying anti rheumatic drugs (DMARDs) in oJIA and ERA. Methotrexate (MTX) was the most commonly used DMARD, mainly in polyarthritis RF positive (83%) children. Sulfasalazine was used in 9(56%) children with ERA. Biological agents were not used in any of our patients.

**Conclusion:** The clinical and epidemiological profile of children in our study with JIA was different from the Western countries with almost equal frequency of JIA in boys and girls and high prevalence of ERA. Uveitis was uncommon. Majority of Patients are doing well on follow-up and thus use of biological agents has not been considered in some whereas cost is the limiting factor in others.

**Disclosure of Interest**

None declared

### P50 MS Developed on a JIA background drug induced side effect or autoimmune prone patients?

#### S. Karamichalou^1^, A. Stamati^1^, M. Chondrogianni^2^, G. Vartzelis^1^, V. Konstantinos^2^, O. Vougiouka^1^

##### ^1^2nd Department of Pediatrics, ‘P&A Kyriakou’ Children’s Hospital, National and Kapodistrian University of Athens; ^2^2nd Department of Neurology, Attikon Hospital, National and Kapodistrian University of Athens, Athens, Greece

###### **Correspondence:** O. Vougiouka

**Introduction:** Tumor necrosis factor antagonists (anti-TNFa) have been paramount in the management of several autoimmune diseases. Despite their clinical effectiveness, there are reports documenting their potential role in the induction or aggravation of demyelination of the central nervous system (CNS) [1, 2]. Or it may be only a coincidence due to the underlying genetic susceptibility of these patients to disturbed autoimmune reactivity [3].

**Objectives:** We report two new cases of demyelinating events of the CNS, following treatment with anti-TNFa for two and nine years respectively, in two girls with polyarticular type of Juvenile idiopathic arthritis background, firstly diagnosed at the age of seven and twelve years old respectively.

**Methods:** We investigated these cases of demyelination with laboratory examinations (screening for auto-antibodies, viral infection), lumbar puncture and magnetic resonance imaging (MRI) of the brain and spinal cord.

**Results:**

**Conclusion:** Inflammatory demyelination of the CNS may be associated with the use of anti-TNFa. Thus, all patients should, before and throughout the treatment, be monitored for any neurological complication, indicative of demyelination and evaluate appropriate treatment interventions [1]. However, we should not forget the co-occurrence of MS with other autoimmune disorders because of immunologic predisposition [4].

REFERENCES

1. Atzeni, F., et al., *Concerns about the safety of anti-TNF agents when treating rheumatic diseases.* Expert Opinion on Drug Safety, 2020. 19(6): p. 695-705.

2. Seror, R., et al., *Pattern of demyelination occurring during anti-TNF-α therapy: a French national survey.* Rheumatology, 2013. 52(5): p. 868-874.

3. Andreadou, E., et al., *Demyelinating Disease following Anti-TNFa Treatment: A Causal or Coincidental Association? Report of Four Cases and Review of the Literature.* Case Reports in Neurological Medicine, 2013. 2013: p. 671935.

4. Kaouther, B.A., et al., *Concurrence of Juvenile Idiopathic Arthritis and Multiple Sclerosis.* Case Reports in Rheumatology, 2011. 2011: p. 162857.

**Patient Consent Received**

No

**Disclosure of Interest**

None declared

**Table 1 (abstract P50).** See text for description

	Patient 1	Patient 2
RF/anti – CCP / ANA	(-) / (-) / (-)	(+) /(+) /(+)
Rheumatoid nodules / morning stiffness	(-) / (+)	(+)/ (+)
**Anti- TNF a**	Etanercept (4 months) Adalinumab (2 years)	Adalinumab (9 years)
**Neurological symptoms**	right facial palsy Romberg right	Left optic neuritis
Anti- MOG / Anti- AQP4	(-) / (-)	(-) / (-)
Oligoclonal bands	type 3	type 2
MRI lesions of the brain – focal high intensity white matter lesions	in cerebral hemispheres & brain stem	in optic nerves & next to the lateral ventricles
MRI lesions of the spinal cord without enhancement	at the cervical level C2 and thoracic level T6, T11- T12	at the cervical level C5
**Autoimmune medical history**	ENA (-)	ENA (-) Anti- TPO (+) Anti-TG (+)
**Family medical history of RA**	paternal aunt	grand mother

*ANA* antinuclear antibodies, *RF* rheumatoid factor, *RA* rheumatoid arthritis

*Anti-CCP* anti-cyclic citrullinated peptide, *ENA* extranuclear antibodies

*Anti-TPO* anti thyroid peroxidase antibodies, *Anti-TG* anti thyroglobulin antibodies

*Anti-MOG* anti-myelin oligodendrocyte glycoprotein, *Anti-AQP4* anti-aquaporin 4

### P51 Down syndrome associated arthritis or JIA: clinical case

#### Y. Vyzhga, N. Tokarchuk

##### National Pirogov Memorial Medical University, Vinnytsya, Vinnitsya, Ukraine

###### **Correspondence:** Y. Vyzhga

**Introduction:** In abstract presented clinical case of arthritis in 9 yr. patient with Down syndrome. In the literature it’s still doubtful, if patients with Downs syndrome may present clear JIA or its some other type of the arthritis associated with genetic pathology.

**Objectives:** Our goal is to check and evaluate possibility of the JIA development in patient with Down syndrome.

**Methods:** To evaluate clinical importance, we examined patient with Down syndrome who was presenting signs of arthralgia for a time frame more than 6 weeks and who was admitted to pediatric rheumatologist with suspicion of arthritis onset.

**Results:** Patient D., 9 yr. came to the center 6 months ago. Patient became sick approximately 8 months ago, but due to difficulties associated with mental and behavior development, mother didn’t concern about joint pain. Acute episode of the disease started with limping gait, child wasn’t able to move at the morning, that was associated with pain syndrome in both knees. Before this episode of the disease child was healthy. The baby was delivered from the 1st pregnancy, in term, didn’t have any complicated family or epidemiological anamnesis. Mom went to the family doctor; symptomatic treatment was started together with systemic antibacterial drugs. Within 14-day condition wasn’t significantly improved, child still presented pain, less intense than at the onset of the disease, and as well limping gait. General practitioner diagnosed reactive arthritis and continued NSAIDs. The child was followed with complete blood count that showed mild inflammation, X-ray of the joints didn’t show any pathological changes. General duration of the disease was longer than 6 weeks, but general practitioner ensured mom, that they don’t require consultation of any other specialist. When the child was sick for 2 months mother by herself admitted him to the pediatric rheumatologist. During first investigation child showed signs of the arthritis of both knee joints, with typical swelling, mild to moderate pain, movements limitation, morning stiffness up to 1 hour. Other joints were not involved to the process, laboratory results showed mild increased ESR (21 mm/h), negative CRP, signs of the synovitis of both knee joints. Aspiration of the synovial fluid was presented with following intraarticular injection of the steroid. During detailed investigation child performed ANA 1:320, aCCP – 34 IU/ml (N – up to 8 IU/ml). After the intraarticular injection situation much more improved, resolved pain syndrome, movement limitation. In 2-month, child returned back with the same complains on pain and movement limitation in both knee joints, but with minimal synovitis. Laboratory activity was mild, but taking into account previous disease anamnesis, results of additional investigations, diagnose of JIA was estimated and started treatment with methotrexate. By this time child was taking methotrexate for 3,5 months, JADAS-27 – 5 was achieved (compare with initial JADAS-27 - 9).

**Conclusion:** By this time a lot of discussions are opened in arthritis associated with Down syndrome. Classical presentation has small joints involvement without autoimmune markers. Our patient showed other clinical signs so diagnose of the JIA is more adequate in this particular case.

**Disclosure of Interest**

None declared

### P52 Knowledge of juvenile idiopathic arthritis among Israeli pediatricians might improve early referral and disease outcomes

#### A. Ziv^1^, M. H. Bekenstein^2^, D. Schujovitzky^3^, S. Eylon^4^, P. J. Hashkes^5^, Y. Uziel^6^, R. Haviv^6^, Z. Grossman^7^

##### ^1^1Pediatric Rheumatology Unit, Department of Pediatrics, Meir Medical Center, Kfar Saba, Israel., Meir Medical Center, kfar saba; ^2^pediatrics, 3Pediatric Rheumatology Clinic, Department of Pediatrics, Dana-Dwek Children’s Hospital, Tel Aviv, Tel Aviv; ^3^Pediatrics, Meir Hospital, Kfar Saba, Israel, kfar saba; ^4^Pediatric Orthopedic Service, Alyn Rehabilitation for Children & Adolescents, Jerusalem, Israel, Chair of the Israel Pediatric Orthopedic Society, Alyn Rehabilitation for Children & Adolescents; ^5^Paediatric Rheumatology Unit, Shaare Zedek Medical Centre, Jerusalem, Israel, Shaare Zedek Medical Center, Jerusalem; ^6^1Pediatric Rheumatology Unit, Department of Pediatrics, Meir Medical Center, Kfar Saba, Israel., Meir Medical Center, kfar saba; ^7^5Adelson School of Medicine, Ariel University, Ariel, Israel and Maccabi Healthcare Services, Tel Aviv, Israel, Israel and Maccabi Healthcare Services, Tel Aviv, Israel

###### **Correspondence:** A. Ziv

**Introduction:** Approximately 1 child in 1,000 is affected by chronic juvenile idiopathic arthritis (JIA). Persistent, undiagnosed JIA with high disease activity interferes with daily life and carries a risk of irreversible physical and psychosocial damage. Due to its relative rarity, primary physicians often do not recognize it; thus, diagnosis and referral to pediatric rheumatologists are delayed.

**Objectives:** To evaluate the knowledge of Israeli pediatricians and pediatric-orthopedic surgeons regarding epidemiology, clinical manifestations, laboratory parameters and treatment of JIA.

**Methods:** An 11-item online questionnaire regarding JIA sent to the lists of Israeli Society of Pediatrics and Pediatric Orthopedics, was completed by 274 pediatricians and 27 pediatric-orthopedic surgeons.

**Results:** Average score was 67.8% overall participants. Several groups were associated with better overall scores: hospital physicians compared to community physicians; pediatric residents (especially after board exams) compared to seniors; exposure to rheumatology during residency; and more JIA patients during the last 5 years. No significant difference was found between pediatricians and pediatric-orthopedic surgeons. 40% of participants underestimated the true incidence of JIA; 30-45% were not familiar with its clinical presentation (age of onset, pain characteristics, chronic uveitis symptoms), and up to 65% were not familiar with up-to-date treatments.

**Conclusion:** Israeli pediatricians and pediatric-orthopedic surgeons have misconceptions regarding JIA. This could result in delayed referral and treatment, which might affect outcomes. The results of this study highlight the need for better education and exposure to a rheumatologist, leading to the goals of better health and quality of life for JIA patients by improving knowledge.

**Patient Consent Received**

No

**Disclosure of Interest**

None declared

## e-Poster viewing: Systemic JIA

### P53 Systemic juvenile idiopathic arthritis associated lung disease in Europe

#### C. Bracaglia^1^, on behalf of MAS/sJIA Working Party, F. Minoia^2^, C. Kessel^3^, S. Vastert^4^, M. Pardeo^1^, A. Arduini^1^, O. Basaran^5^, N. Kiper^6^, M. Kostik^7^, M. Glerup^8^, S. Fingerhutova^9^, R. Caorsi^10^, A. Horne^11^, G. Filocamo^2^, H. Wittkowski^3^, M. Jelusic^12^, P. Dolezalova^9^, A. Ravelli^13^, S. Ozen^5^, F. De Benedetti^1^, on behalf of MAS/sJIA Working Party

##### ^1^Division of Rheumatology, IRCCS Ospedale Pediatrico Bambino Gesù, Roma; ^2^Fondazione IRCCS Ca’ Grande Ospedale Maggiore Policlinico, Milano, Italy; ^3^Department of Pediatric Rheumatology & Immunology, WWU Medical Center (UKM), Muenster, Germany; ^4^Pediatric Rheumatology & Immunology, University Medical Center Utrecht, Utrecht, Netherlands; ^5^Department of Pediatrics, Division of Pediatric Rheumatology; ^6^Department of Pediatrics, Division of Pediatric Pulmonology, Hacettepe University, Ankara, Turkey; ^7^Saint-Petersburg State Pediatric Medical University, Saint-Petersburg, Russian Federation; ^8^Department of Pediatrics, Aarhus University Hospital, Aarhus, Denmark; ^9^Paediatric Rheumatology and Autoinflammatory Diseases Unit, General University Hospital, Prague, Czech Republic; ^10^Department of Pediatrics and Rheumatology, IRRCS Istituto Giannina Gaslini, Genova, Italy; ^11^Department of pediatric rheumathology Karolinska University Hospital and Department of pediatrics, Karolinska Institute, Stockholm, Sweden; ^12^Department of Paediatrics, University of Zagreb School of Medicine, Zagreb, Croatia; ^13^IRCCS Istituto Giannina Gaslini and Università degli Studi di Genova, Genova, Italy

###### **Correspondence:** C. Bracaglia

**Introduction:** Chronic parenchymal lung disease (LD) is a new emerging severe life-threatening complication of sJIA. The number of sJIA patients with LD is apparently increasing and interestingly it is reported more frequently in North America. Data regarding frequency and features of sJIA-LD in Europe are not available.

**Objectives:** To evaluate the burden of sJIA-LD in Europe.

**Methods:** Patients with diagnosis of sJIA with LD, including pulmonary alveolar proteinosis (PAP), interstitial lung disease (ILD) and pulmonary arterial hypertension (PAH), followed in European paediatric rheumatology centres were identified through a survey sent to the members of the MAS/SJIA Working Party.

**Results:** Data from 26 sJIA-LD patients, diagnosed in 10 European paediatric rheumatology centres between 2006 and 2020, were collected. 25 patients were Caucasian and 1 African-American, 16 were female, the median age at sJIA onset was 6.4 years and LD onset occurred after a median time of 2.7 years. 15 patients had a chronic persistent sJIA disease course, 10 had a polycyclic course and only 1 patient had a monocyclic course; 24 (92%) had active sJIA at time of LD diagnosis. During the disease course, 21 (81%) patients developed MAS, 9 (34%) of whom had MAS at sJIA onset and 15 (57%) had full-blown MAS at time of LD diagnosis; 21 (80%) patients had >1 MAS episode. 19 (73%) patients were treated with at least one IL-1 or IL-6 inhibitor before LD diagnosis: 12 with canakinumab, 17 with anakinra and 10 with tocilizumab. 10 (38%) patients experienced drug adverse reaction to a cytokine inhibitor: 8 to tocilizumab and 2 to anakinra. 20 (77%) patients developed ILD, 4 (15%) PAP and 3 (11%) PAH. 13 (50%) patients presented acute digital clubbing; 10 (38%) patients developed hypoxia and 6 (23%) developed pulmonary hypertension. A chest CT scan was performed in all patients with evidence of septal thickening and peri-bronchovascular thickening in the majority of patients (22 and 14 respectively). In 12 patients a bronchoalveolar lavage was performed and 9 underwent a lung biopsy. The histopathological pattern was alveolar proteinosis in 4 patients, endogenous lipoid pneumonia in 3, vasculitis in 1 and fibrosis in 1. Twelve (46%) patients required ICU admission and 3 (11%) died. All patients were treated with glucocorticoids (GCs) at time of diagnosis, and 22 received IL-1 or IL-6 inhibitor after the diagnosis (12 canakinumab, 15 anakinra, 13 tocilizumab).

**Conclusion:** Lung involvement is an emerging life-threatening complication of sJIA in Europe, especially in patients with a history of MAS, and a prompt recognition is crucial. New strategies are needed to reduce the risk and improve outcome of this complication.

**Disclosure of Interest**

C. Bracaglia Consultant for: Sobi, Novartis, F. Minoia Consultant for: SOBI, C. Kessel Consultant for: SOBI, Novartis, S. Vastert Consultant for: SOBI, Novartis, M. Pardeo: None declared, A. Arduini: None declared, O. Basaran: None declared, N. Kiper: None declared, M. Kostik: None declared, M. Glerup: None declared, S. Fingerhutova: None declared, R. Caorsi Consultant for: Novartis, Lilly, Speaker Bureau of: SOBI, A. Horne: None declared, G. Filocamo Consultant for: SOBI, H. Wittkowski: None declared, M. Jelusic: None declared, P. Dolezalova: None declared, A. Ravelli Consultant for: AbbVie, Novartis, Pfizer, Angelini, Reckitt Benkiser, S. Ozen Consultant for: Novartis, Pfizer, and Sobi, F. De Benedetti Consultant for: Abbvie, SOBI, Novimmune, Novartis, Roche, Pfizer

### P54 Comparison of potential biomarkers differentiating systemic juvenile idiopathic arthritis from other causes of fever of unknown origin in children

#### P. R. Chickermane^1^, S. Krishnan^2^, A. Tiwari^1^, H. Hari^3^, S. Balan^1^

##### ^1^Department of Clinical Immunology and Rheumatology; ^2^Department of Biochemistry; ^3^Department of Biostatistics, Amrita Institute of Medical Sciences, Kochi, India, Kochi, India

###### **Correspondence:** P. R. Chickermane

**Introduction:** Systemic juvenile idiopathic arthritis (sJIA), a prototype of systemic rheumatic diseases in children is one of the common conditions presenting as fever of unknown origin (FUO) in the pediatric age group. Children with sJIA often prsent initially with FUO with systemic features preceding the onset of arthritis by weeks, months or even years. Diagnosing sJIA in these cases is challenging and highlights the need for a diagnostic biomarker to facilitate an early diagnosis and treatment. Dysregulation of innate immune response with overproduction of macrophage derived cytokines IL-1, IL-6, IL-18, S100 calcium-binding proteins S100A8 and S100A9 (calprotectin) is implicated in the pathogenesis of sJIA. A few studies have assessed potential biomarkers for sJIA in the recent years, however no such study has been conducted in the Indian population.

**Objectives:** To evaluate serum IL-1, IL-6, IL-18, S100A8 and S100A9 as potential diagnostic markers to distinguish sJIA from other conditions presenting as FUO in children.

**Methods:** A prospective cross-sectional study was conducted at a 1500-bedded tertiary care centre in Kerala, southern India between May 2019 and October 2020. Children under 16 years of age who presented with FUO defined as "fever > 38.0°C (100.4°F) lasting for at least 8 days without a clear source" were enrolled on the study. Patients who had already received glucocorticoids and/or immunosuppressive therapy were excluded. Serum concentrations of IL-1, IL-6, IL-18, S100A9 and S100A8 were determined using enzyme-linked immunosorbent assay (ELISA) kits. Receiver operating curve (ROC) analysis was used to determine the cut-off values for IL-1, IL-6, IL-18, S100A8 and S100A9 for differentiating sJIA from other causes of fever.

**Results:** Forty-seven children (females- 27) who presented with FUO were enrolled. Nineteen of them were eventually diagnosed with sJIA according to the International League of Associations for Rheumatology (ILAR) classification. In the other 28 children, fever was attributed to conditions other than sJIA (non-sJIA). The non-sJIA group comprised of children with acute lymphoblastic leukemia (n=6), hemophagocytic histiocytosis (n=5), systemic lupus erythematosus (n=4), systemic infections (n=4), Kawasaki disease(n=2), Kikuchi disease (n=2) and inflammatory bowel disease (n=2). This group also included one child each diagnosed with Sweet syndrome with polyarthritis, drug rash with eosinophilia and systemic symptoms (DRESS) syndrome and post-infection multisystem inflammatory disease. Serum levels of IL-18, S100A8 and S100A9 were significantly higher in patients with sJIA compared to the non- sJIA group (p<0.05) (Table-1). ROC analysis showed that the area under the curve (AUC) was significant for IL-18 (77.9%), S100A8 (74.9%) and S100A9 (71.2%). A serum IL-18 cut-off level of > 2030.45 pg/ml was useful for differentiating between sJIA and other diseases with a sensitivity of 66.67% and specificity 75.86% for the diagnosis of sJIA.

**Conclusion:** Serum IL-18, S100A8 and S100A9 can be useful in differentiating sJIA from other causes of FUO in children.

**Patient Consent Received**

Yes

**Disclosure of Interest**

None declared

**Table 1 (abstract P54).** Comparison of candidate biomarkers in patients with sJIA and other causes of FUO in children (non- sJIA)

Parameter	sJIA (n=19)	Non- sJIA (n=28)	p value
IL-1 (pg/ml)	42.81 (3.77-94.57)	85.06 (12.34-248.45)	0.104
1L-6 (pg/ml)	25 (4.65-120.41)	9.73 (2.98-27.21)	0.106
1L-18 (pg/ml)	2,042.8 (1,998.3-2,063)	1,942.15 (1,227.62-2026)	**0.001**
S100A8 (ng/ml)	478.2 (291.5-576.4)	233.15 (145.6-446.95)	**0.005**
S100A9 (ng/ml)	465.1 (417.1-681)	239.05 (96.25-491.9)	**0.018**

Values expressed as: median (inter-quartile range)

### P55 Fatty acid uptake is increased in monocytes of patients with systemic juvenile idiopathic arthritis and blockade regulates IL8 and TNFΑ production

#### R. Erkens^1^, K. Ohl^2^, R. Scholman^1^, J. Van Loosdregt^1^, K. Tenbrock^2^, B. Vastert^1^

##### ^1^Wilhelmina kinderziekenhuis, UMC Utrecht, Utrecht, Netherlands; ^2^RWTH Aachen University, Aachen, Germany

###### **Correspondence:** R. Erkens

**Introduction:** Fatty acids (FA) are key players in cellular homeostasis. Interestingly changes in FA metabolism can influence inflammation by controlling transcriptional and posttranscriptional events that are central to immune activation. FA metabolism might also play an important role in JIA inflammation. Indeed, levels of polyunsaturated FA are associated with JIA disease course and active disease shows decreased arachidonic acid levels(1). Monocytes are essential cellular components of the innate immune system and in systemic idiopathic arthritis (sJIA) monocytes play a central role(2). In a glucose deprived milieu monocytes shift to increased FA metabolism so cytokine secretion, migration, and phagocytosis remain intact(3). This is also the case in a low oxygen environment such at the synovium(4). Therefore FA uptake and metabolism might play a role in monocyte inflammation in sJIA. We hypothesize that investigating the effects of FA uptake inhibition on monocyte metabolism and inflammation can contribute to a better understanding of sJIA pathogenesis, the identification of cellular pathways contributing to disease and possibly to new therapeutic targets.

**Objectives:** To investigate the effects of fatty acid uptake inhibition on monocyte metabolism and phenotype, assessed by changes in cytokine production.

**Methods:** We used the monomac 6 cell line, as well as primary monocytes from healthy control and SJIA patients in active disease. We measured the uptake of FA analog C_1_-BODIPY-C_12_ using flow cytometry and studied the effects of inhibition of FA uptake by pre-incubation with FA uptake inhibitors, lipofermata and grassofermata. In addition, we studied the effects of FA uptake inhibition on monocyte cytokine and prostaglandin production and metabolism using FACS and qPCR.

**Results:** Monomac 6, a cell line for human monocytes, show increased uptake of FA after LPS stimulation compared to unstimulated cells. The uptake of the FA can be inhibited with lipofermata and grassofermata in a dose depend way. FA uptake inhibition decreases IL 8 and TNFα production in these cells upon LPS stimulation. Preliminary results from experiments using primary cells show an increased uptake of FA in monocytes from active SJIA synovium compared to monocytes derived from healthy donor blood and a decreased inhibition of FA uptake with lipofermata.

**Conclusion:** The analysis of FA uptake and metabolism offers new insights in immune regulation. Our preliminary results in a monocyte cell line and sJIA patient monocytes suggest that this might play a role as well in monocytes in the inflammatory cascade in active sJIA. Targeting FA immunometabolism could potentially be exploited for the treatment of autoimmune diseases in the future.

**References**

1. Gorczyca D, Postępski J, Czajkowska A, Paściak M, Prescha A, Olesińska E, et al. The profile of polyunsaturated fatty acids in juvenile idiopathic arthritis and association with disease activity. Clin Rheumatol. 2017;

2. Leong JY, Guan YJ, Albani S, Arkachaisri T. Recent advances in our understanding of the pathogenesis of juvenile idiopathic arthritis and their potential clinical implications. Expert Review of Clinical Immunology. 2018.

3. Raulien N, Friedrich K, Strobel S, Rubner S, Baumann S, von Bergen M, et al. Fatty acid oxidation compensates for lipopolysaccharide-induced Warburg effect in glucose-deprived monocytes. Front Immunol. 2017;

4. Rodgers LC, Cole J, Rattigan KM, Barrett MP, Kurian N, McInnes IB, et al. The rheumatoid synovial environment alters fatty acid metabolism in human monocytes and enhances CCL20 secretion. Rheumatol (United Kingdom). 2020;

**Disclosure of Interest**

R. Erkens: None declared, K. Ohl: None declared, R. Scholman: None declared, J. Van Loosdregt: None declared, K. Tenbrock Grant / Research Support from: Pfizer and Novartis, Consultant for: BMS, Pfizer and Novartis, B. Vastert Grant / Research Support from: Sobi, Consultant for: Sobi and Novartis

### P56 Systemic onset juvenile idiopathic arthritis, experience of one center

#### S. S. Hashad, H. M. Etayari, M. N. Etfil, E. A. Almsallati, A. A. Altawati, Z. O. Awhaidah, S. Alhadi Mohamed, M. S. MOUJRANI

##### Pediatric Rheumatology, Tripoli Children Hospital, Tripoli, Libya

###### **Correspondence:** H. M. Etayari

**Introduction:** Systemic onset Juvenile idiopathic arthritis (sJIA), differs from other JIA subtypes in its clinical manifestations and pathogenesis. It is characterized by a severe course and has the highest rate of mortality and morbidities.

**Objectives:** To study initial manifestations, laboratory findings, course, outcome and complications of patients of sJIA in pediatric rheumatology clinic in Tripoli Children Hospital, Which is one of two referral clinic covering the western and southern part of Libya

**Methods:** The files of the patients diagnosed with sJIA from 5/2000 t0 5/2021 were reviewed and data was retrospectively collected regarding the presenting symptoms, initial investigations, disease course, complications and treatment used. Patients who followed up for less than 6 months were excluded.

**Results:**

There were 38 patients with sJIA diagnosed according to the International League Against Rheumatism (ILAR) criteria were included. 47.4% of them were males and 52.6% were females. They were followed up for a period of 6.3±4.4y.

The mean period from appearance of symptoms to first follow up in rheumatology clinic 5±6 months.

The following table summarizes the most common presenting symptoms among the patients:

**Table:** 

Symptom	percentage
Fever	100%
Rash	78.9%
Lymphademopathy	55.3%
Hepato and/or splenomegally	15.8%
Pericarditis	18.4%
Myocarditis	2.6%
Pleuritis	2.6%
Arthralgia without arthritis initially	7.9%
Arthritis	92.1%

There is one patient presented with MAS and required ICU management.

The most frequently affected joints are the knees (76.3%) followed by the ankles (73.7%) then the wrists (55.3%) and the elbows (39.5%).

The disease course was monocyclic in 39.5%, polycyclic in 28.9% and persistent in 31.6% of the patients. and in 65.8% the disease has polyarticular pattern while in the rest of the patients it followed an oligoarticular pattern.

The number of patients who achieved remission is 30 (78.9%), 16 (53.3%) of them were in remission on treatment while 14 (46.7%) of them were in remission off treatment

Oral steroids, NSAIDs and MTX were used as a first line treatment to control the disease in the patients. Also intraarticular injections were used in 21.1% of the patients. In 55.3% of the patients switching to biologics was necessary to control the disease, Etanercept was sufficient in controlling the disease in only 2 patients (5.2%) while Anakinira was sufficient in controlling the disease in 5 patients (13.2%) and Tocilizumab was sufficient in controlling the disease in 6 patients (15.8%). More than one biologic used in 8 patients.

Joint deformities were present in 10 (26.3%) of the patients, and evidence of extra-articular damage is present in 12 (31.6%). MAS occured in 4 (10%) of the patients and one patient experienced 3 mas episodes

**Conclusion:** There is a high rate of disease remission on and off treatment and high rate of disease related articular and extra-articular damage because alot of patients started biologics late after disease onset.

**Patient Consent Received**

No

**Disclosure of Interest**

None declared

### P57 Thalidomide in 30 patients with refractory systemic juvenile idiopathic arthritis

#### A. Khan^1^, D. Ramadoss^1^, P. Pimpale Chavan^2,3^, K. Tiwari^4^, R. Khubchandani^1^

##### ^1^Pediatric Rheumatology; ^2^Former Pediatric Rheumatologist, NH SRCC Childrens Hospital, Mumbai, India; ^3^Current: Inflammatory Disease Section, National Human Genome Research Institutes,National Institutes of Health, Bethesda, United States; ^4^Former Pediatric Subspeciality Fellow, Jaslok Hospital and Research Centre, Mumbai, India

###### **Correspondence:** A. Khan

**Introduction:** Thalidomide made a comeback at the turn of century for use in Erythema nodosum leprosum (anti TNF) and multiple myeloma (anti angiogenic). It has also been used in various pediatric autoinflammatory conditions notably sJIA. We report the largest experience with this drug in sJIA, adding to our earlier reported series.

**Objectives:** Share our experience with thalidomide in 30 patients of sJIA refractory to cDMARDs with /without biologicals.

**Methods:** Thalidomide was commenced in 30 patients beyond 4 years of age (children who could vocalize pain and paraesthesia) who failed to achieve disease control with NSAIDs,steroids,cDMARDs like methotrexate (n=3) or combination of methotrexate and leflunomide (n=24). 23/30 refused step up treatment to biologics due to cost constraints. 7 had also received biologicals in combination with methotrexate (etanercept- 4, tocilizumab-3) but were shifted to thalidomide due to poor response (6) and cost constraints (1). Before initiating thalidomide,families were educated in their preferred language about thalidomide,its adverse effects and limited evidence in medical literature for use in children and sJIA. After consent, thalidomide available as capsules of 50 /100 mg was commenced as a single night dose (2-3mg/kg/day) along with high fibre diet. Thalidomide was started along with at least one cDMARD. On 8-12 weekly follow up,disease activity was assessed based on systemic features, active joint counts, acute phase reactants and reduction of steroid dose. Complete response was defined as no active joints or systemic features, ESR < 25mmhr, dose of prednisolone < 0.3mg/kg/day and no response as no reduction in joint counts with persistent systemic features, ESR > 40mm/hr and prednisolone dose > 0.5mg/kg/day. Others were deemed partial responders. At every visit specific enquiry about features of peripheral neuropathy (tingling,numbness and paraesthesia), neurological examination (nerve conduction study if needed ), complete hemogram and liver function were studied.

**Results:** The age range of our patients was 4-17 years. Thalidomide was discontinued in 2/30 patients due to rash and thrombocytopenia respectively within a month of starting treatment and 1 patient was lost to follow up. 27/30 patients were followed up over 970 patient – months with a range of 2-96 months (Median= 26.5). 16/27 (59.2%) showed complete response. In 1/16 of responders’ thalidomide was discontinued after 22 months due to peripheral neuropathy and 1 patient with constipation was managed with diet modification and stool softeners. 6/27 (22.2 %) patients had no response. 4/6 were shifted over to biologics of whom 3 have responded to tocilizumab and 1 to etanercept biosimilar.2/6 were lost to follow up. They had no adverse effects related to thalidomide while being followed on drug. 5/27 (18.5 %) patients showed partial response with no side effects. 7/30 patients were on biologicals before being shifted to thalidomide. 4/7 had shown no response to etanercept biosimilar tried in a dose of 0.8 mg/kg/week for 8 - 48 weeks. 1 of these patients failed to show response to thalidomide but eventually responded to tocilizumab. 3/7 patients received tocilizumab in a dose of 8-12 mg/kg fortnightly intravenously for duration ranging 16 weeks to 28 weeks. All 3/7 patients who showed no response to tocilizumab responded well to thalidomide.

**Conclusion:** In resource challenged situations cost of biologics is high. Thalidomide offers an alternative in patients failing cDMARDs and/or biologics (after due parent information through a regulated drug availability program)**.** 10% of our patients had major adverse effects necessitating stoppage emphasizing the need for close supervision.

**Patient Consent Received**

No

**Disclosure of Interest**

None declared

### P58 Experience of tocilizumab use for systemic juvenile idiopathic arthritis in children under 2 years

#### E. Krekhova^1,2^, E. Alexeeva^1,2^, T. Dvoryakovskaya^1,2^, R. Denisova^2^, K. Isaeva^2^, O. Lomakina^2^, A. Mamutova^2^, A. Chomakhidze^2^, A. Fetisova^2^, M. Gautier^2^, I. Kriulin^1,2^, I. Tsulukiya^2^

##### ^1^Pediatric, Sechenov First Moscow State Medical University; ^2^Rheumatology, National Medical Research Center of Children's Health, Moscow, Russian Federation

###### **Correspondence:** E. Krekhova

**Introduction:** Tocilizumab (TOC) was approved in the treatment of patients with systemic juvenile idiopathic arthritis (sJIA) aged 2 to 17 years. Currently, one open-label phase 1 clinical trial has been carried out on the use of TOC under the age of 2 years.

**Objectives:** To evaluate the drug efficacy and safety of TOC in patients younger than 2 years with sJIA treated at the National Medical Research Center of Children`s health, Moscow, Russia.

**Methods:** We analyzed retrospectively the medical records of 23 patients younger than 2 years with sJIA which were prescribed to TOC. Informed consent of the patient's parents and approval of the medical commission for the prescription of off-label drugs was obtained. Inactive disease was defined according to the criteria of Wallace et al.

**Results:** 23 patients (11 — male, 12 — female) were initiated on TOC therapy (8-12 mg/kg IV every 2 weeks). The average age of onset of sJIA was 1.15 year (IQR 0.79-1.5), the average age at initiation - 1.4 year (IQR 1.2-1.7). At initiation, systemic features were noted in all patients, arthritis was present in 5/23 patients. All patients underwent exclusion of autoinflammatory syndromes due to the early age of onset of sJIA. At TOC initiation 16/23 patients received glucocorticosteroids (GCS), 6/23 patients received methotrexate. AE was noted in 7 patients at TOC initiation (6 - neutropenia; 1 - severe allergic reaction).

After 1 month of therapy, 30/50/70/90% improvement according to the ACR criteria was noted in 86/86/82/70% of 23 patients, and 57% of patients achieved remission according to C. Wallace. The lack of disease activity according to the JADAS-71 index was noted in 65% (13/23) of patients.

By the 6th month of therapy, 30/50/70/90% improvement according to the ACR criteria was noted in 78/78/78/74% of 23 patients, 70% (16/23) of patients achieved remission according to C. Wallace. 74% (17/23) of patients achieved JADAS-71 remission.

After 12 months of therapy, 30/50/70/90% improvement according to ACR criteria was noted in 81/81/76/76% of 21 patients (2 patients were follow-up for less than 1 year), remission according to C. Wallace and JADAS-71 index was reached by 71% (15/21) of patients.

TOC was discontinued in 11/23 patients. 3 patients had primary inefficiency, so we changed the therapy. 1 patient had a severe allergic reaction to the first infusion of TOC, but the stage of remission was achieved after infusion, today he continues to receive methotrexate. 1 patient developed secondary inefficiency. 6/23 (26%) patients achieved drug-off remission.

AE was noted in 15/23 patients in our observation. The most common AE was neutropenia (in 11/23 patients), especially at the beginning of TOC therapy. 4 patients were infected with tuberculosis without signs of an active process. 4 episodes of pneumonia were recorded and 2 cases of chickenpox. 2 (8.7%) patients had severe allergic reactions on TOC therapy.

Only 2/20 patients had a flare of sJIA during TOC therapy after achieving remission. 1 patient was switched to other biological drug. 1 patient received GCS pulse therapy due to a temporary delay of TOC administration.

**Conclusion:** The mean duration of TOC therapy was 27 months (IQR 9-40). The mean time of remission at TOC therapy was 29 months (IQR 9.5-56). Data from our experience suggest that TOC provided efficacy in sJIA patients younger than 2 years comparable to those in patients aged 2 to 17 years and also has an acceptable safety profile.

**Disclosure of Interest**

None declared

### P59 Lung functioning and inflammation in a mouse model of systemic juvenile idiopathic arthritis

#### B. Malengier Devlies^1^, T. Decaesteker^2^, K. Dekoster^3^, A. Vanstapel^2^, K. Ahmadzadeh^1^, F. Poosti^4^, T. Mitera^1^, L. Seldeslachts^3^, E. Verbeken^5^, C. Wouters^1,6,7^, G. Vande Velde^3^, J. Vanoirbeek^2^, P. Matthys^1^

##### ^1^Laboratory of Immunobiology, Department of Microbiology and Immunology, Rega Institute KU Leuven; ^2^Laboratory of Respiratory Diseases and Thoracic Surgery (BREATHE), Department of Chronic Diseases and Metabolism; ^3^Biomedical MRI, Department of Imaging & Pathology, KU Leuven; ^4^Laboratory of Molecular Immunology, Department of Microbiology and Immunology, Rega Institute KU Leuven; ^5^Morphology and Molecular Pathology Section; ^6^Division of Pediatric Rheumatology; ^7^European Reference Network for Rare Immunodeficiency, Autoinflammatory and Autoimmune Diseases (RITA), University Hospitals Leuven, Leuven, Belgium

###### **Correspondence:** B. Malengier Devlies

**Introduction:** Systemic juvenile idiopathic arthritis (sJIA) is an immune disorder characterized by fever, skin rash, arthritis and splenomegaly. Recently, increasing number of sJIA patients were reported having lung disease.

**Objectives:** We explored lung abnormalities in a mouse model for sJIA relying on injection of IFN-γ deficient (IFN-γ KO) mice with complete Freund's adjuvant (CFA).

**Methods:** Lung abnormalities were assessed using microcomputer tomography, lung functionality tests, histology, flow cytometry and quantitative PCR.

**Results:** Monitoring of lung changes during development of sJIA using microcomputer tomography revealed a moderate enlargement of lungs, a decrease in aerated and increase in non-aerated lung density. When lung function and airway reactivity to methacholine was assessed, gender differences were seen. While male mice showed an increased tissue hysteresivity, female animals were characterized by an increased airway hyperactivity, mirroring ongoing inflammation. Histologically, lungs of sJIA-like mice showed subpleural and parenchymal cellular infiltrates and formation of small granulomas. Flow cytometric analysis identified immature and mature neutrophils, and activated macrophages as major cell infiltrates. Lung inflammation in sJIA-like mice was accompanied by augmented expression of IL-1β and IL-6, two target cytokines in the treatment of sJIA. The increased expression of granulocyte colony stimulating factor, a potent inducer of granulopoiesis, in lungs of mice was striking considering the observed neutrophilia in patients.

**Conclusion:** We conclude that development of sJIA in a mouse model is associated with lung inflammation which is distinct to the lung manifestations seen in sJIA patients. Our observations however underscore the importance of monitoring lung disease during systemic inflammation and the model provides a tool to explore the underlying mechanism of lung pathology in an autoinflammatory disease context.

**Patient Consent Received**

No

**Disclosure of Interest**

None declared

### P60 Lenalidomide- an effective drug for refractory systemic onset juvenile idiopathic arthritis

#### A. P. Rao^1^, A. Girdhar^1^, J. Raghuram^2^

##### ^1^Pediatric Rheumatology, Manipal Hospital; ^2^Pediatric Rheumatology, Indira Gandhi Institute of Child Health, Bangalore, India

###### **Correspondence:** A. P. Rao

**Introduction:** A small subset of Systemic onset Juvenile Idiopathic arthritis (SJIA) patients, remain refractory to first line treatment including NSIADs/steroids/methotrexate. Biologic response modifiers like IL6 or IL1 antagonists are either expensive or are unavailable to many patients in Indian subcontinent. There is a felt need for drugs which are affordable and available to treat these patients.

**Objectives:** The objective of the study was to determine the efficacy, tolerability and safety of lenalidomide in patients with refractory SJIA and in whom biologics were not a plausible option either due to lack of affordability or non-availability.

**Methods:** 9 SJIA patients who were refractory to steroids and methotrexate were started on lenalidomide after informed consent and followed up for a period of 6 months at least. Patients were monitored for clinical improvement in the form of subsidence of fever, joint symptoms and laboratory markers in the form of hemoglobin (Hb), WBC count, ESR and platelet count. All the subjects were also closely monitored for any adverse effects of the drug. The statistical analysis was performed by STATA 11.2 (College Station TX USA).

**Results:** At the end of 6 months of therapy all patients were free from systemic features and most of the patients experienced clinical improvement in articular symptoms. There was a statistically significant improvement in the Hb(p=0.045) and ESR(p=0.008) in the patients. Steroid dosage could be reduced in all the patients and marked improvement was noted in all the laboratory markers. The drug was tolerated well by all the patients and there were no major adverse events observed. The most common side effect observed was generalized hyperpigmentation of skin.

**Conclusion:** Lenalidomide holds the potential of being an important drug in refractory cases of SJIA in a resource poor setting wherein biologic therapies are either unaffordable or unavailable. The limitation of the study is the small sample size and the need is felt for larger studies which can further the knowledge about lenalidomide use in SJIA.

**Patient Consent Received**

Yes

**Disclosure of Interest**

None declared

### P61 Natural history of disease on a systemic juvenile idiopathic arthritis case without diagnosis and treatment. A case report in a developing country

#### S. Rodríguez Aguayo, E. Faugier Fuentes, M. I. De La Cera Rodríguez, H. W. Bermudez Canales, A. Guzman Revilla

##### Paediatric Rheumatology, Hospital Infantil de México Federico Gómez, Ciudad de México, Mexico

###### **Correspondence:** S. Rodríguez Aguayo

**Introduction:** Juvenile idiopathic arthritis (JIA) is the most prevalent chronic rheumatic disease in children and young people, is a major cause of musculoskeletal disability. Low-income countries face a unique set of challenges, which limit the ability to deliver high-quality care to patients with JIA. These challenges include a lack of trained pediatric rheumatologists or pediatricians, inadequate healthcare funding, delay in diagnosis and restricted access to medications.

**Objectives:** The objective of this study is to present the case of a pediatric patient with severe sequelae of systemic JIA, who failed in the diagnosis, treatment and timely referral, to raise awareness of the importance of early diagnosis and timely referral to the pediatric rheumatologist for effective treatment and prevention of disease progression and sequelae.

**Methods:** We presented the case of a pediatric patient with Systemic JIA severe sequelae, that failed in diagnosis, treatment and timely referral to pediatric rheumatologists.

**Results:** A 9-year-old patient who lives on a low socioeconomic and cultural level, came to the emergency room for fever of unknown origin. He has a history of hospitalization for fever in a second level care center 2 years ago. He received diagnosis of community-acquired pneumonia, and was treated intravenous antibiotics, with no response. Due to the persistence of fever, an approach is initiated, infectious causes are ruled out (negative blood and urine cultures, negative serologies for Brucellosis, Ricketssiosis and Lyme disease), and oncological causes too (negative bone marrow aspirate). A computed tomography of the thorax and abdomen reported lymphadenopathy and splenomegaly. Splenectomy is performed in that center, in the abstract of reference to our hospital, they do not explain the surgical indication, however, after the procedure apparently there was fever resolution as equal to the other symptoms, so they perform the hospital egress, but the family did not follow up with the pediatrician, so the patient lost tracing for more than 1 year. Two months before his admission to our center, presented high fever (100-104 °F), arthralgia, arthritis, weight loss 8 pounds, and constitutional symptoms. Clinically patient presented cachexia, malnutrition, paleness, retrognathia, generalized adenopathy, hepatomegaly 4-4-5 cm, arthritis in more than 15 joints, including the temporomandibular joint, shoulders, elbows, wrists, knees and ankles, as well as an axial skeleton that include cervical and lumbar vertebrae with a 2cm Schober test. Presented severe functional limitation with decreased arches of mobility because of the ankylosis. Patient has microcytic anemia (8.4mg/dl), leukocytosis (14,000 cel/ml), thrombocytosis (900,000 cel/ml) and elevated acute phase reactants ESR 23 mm/seg and PCR 11mg/dl. On the radiography images can observe periarticular bone loss, juxta-articular bone erosions and ankylosis. In two years of evolution, any paediatric rheumatologist had evaluated the patient, at the time of our evaluation, the patient had not received DMARDs or other JIA treatment.

**Conclusion:** The early recognition of signs and symptoms suggestive of rheumatic disease is imperative to make a timely diagnosis and provide effective treatment, in order to avoid progression and sequelae. In low-income countries, we must seek strategies to solve the challenges they present, such as a lack of trained pediatricians or pediatric rheumatologists, inadequate funding, and restricted access to medications with the objective of implement a hard and early hit strategy to improve in the evolution and prognosis of the disease.

**Disclosure of Interest**

None declared

### P62 What is hidden under the diagnosis of systemic juvenile idiopathic arthritis: lessons of real clinical practice

#### S. Salugina, M. Kaleda, E. Fedorov, I. Nikishina

##### Pediatrics, V.A. Nasonova Research Institute of Rheumatology, Moscow, Russian Federation

###### **Correspondence:** S. Salugina

**Introduction:** Systemic juvenile idiopathic arthritis (sJIA) is a rare and serious autoinflammatory disease characterized by systemic inflammation (fever, typical rash, serositis, hepatosplenomegaly, lymphadenopathy, acute phase reaction) and is variously accompanied by chronic arthritis, but at the onset of the nonspecific symptoms may be similar to other diseases with a pronounced acute phase inflammatory reaction.

**Objectives:** According to the data of a retrospective study, to analyze in which part of patients (pts) with sJIA the diagnosis is revised, in what time frame, what distinguishes this group at onset.

**Methods:** A total of 104 pts (57 females) with an initial diagnosis of sJIA who underwent inpatient treatment at our center from 2012 to 2020yy were included. The diagnosis of sJIA was verified in accordance with the ILAR criteria (2001), after excluding infectious, neoplastic or autoimmune diseases in regional hospitals. The median age at the onset was 4.5 years [interquartile range (IQR) 2.0; 8.0]. Molecular genetic testing for mutations in the NLRP3, MVK and TNFRSF1A genes was carried out in 101 pts.

**Results:** The diagnosis was changed in 27 pts (25.9%). The median duration of the disease at the time of the revision of the diagnosis was 3.5 [1.5; 6.25] years. Monogenic autoinflammatory diseases (mAIDs) were diagnosed in 22 pts (CAPS – 13, TRAPS – 7, HIDS – 1, FMF – 1). Diagnoses of mAIDs were confirmed by the detection of pathogenic mutations of causal genes. Also polymorphisms NLRP3 was revealed in 74.7% pts in the group who retained the diagnosis of sJIA, but they were clinical insignificant. In 2 pts verified inflammatory bowel disease, 1 – Behcet’s disease, 1 – PFAPA, 1 – neuroblastoma. The median age at the onset and gender were not statistically different between the groups (sJIA and non sJIA). Erythematous rash, typical for sJIA, and arthritis were significantly less common in pts in whom the diagnosis was changed (p=0.048 and p=0.0043, respectively) without difference between groups by other criteria sJIA at onset (p>0.05).

**Conclusion:** According to our data, about a quarter of pts with an inflammatory phenotype similar to the manifestations of sJIA suffer from mAIDS. Typical erythematous rash and arthritis are more likely to indicate the onset of sJIA, but the diagnosis of sJIA should be established with great caution, these pts need a thorough and critical assessment of all symptoms, anamnesis data and mandatory molecular genetic testing for the most common mAIDs. Identification of polymorphisms NLRP3 in pts with sJIA requires a dynamic assessment for a more correct interpretation of its clinical significance.

**Disclosure of Interest**

None declared

### P63 Retrospective analysis evaluating clinical outcomes in Turkish SJIA patients with biological therapeutic agents - tursis study

#### B. Sozeri^1^, K. Barut^2^, E. Atalay^3^, A. Pac Kisaarslan^4^, S. Ozdel^5^, O. Altug Gucenmez^6^, F. Demir^1^, B. Makay^7^, N. Aktay Ayaz^8^, E. Acar^9^, F. Haslak^2^, E. Sag^3^, M. Yıldız^2^, U. Kaya Akca^3^, A. Adrovic^2^, Y. Bilginer^3^, H. Poyrazoglu^4^, E. Unsal^7^, O. Kasapcopur^2^, S. Ozen^3^

##### ^1^Pediatric Rheumatology, University of Health Sciences, Istanbul, Umraniye Training and Research Hospital; ^2^Pediatric Rheumatology, Istanbul University-Cerrahpasa, Istanbul; ^3^Pediatric Rheumatology, Hacettepe University Faculty of Medicine, Ankara; ^4^Pediatric Rheumatology, Erciyes University Faculty of Medicine, Kayseri; ^5^Pediatric Rheumatology, Health Sciences University, Sami Ulus Training and Research Hospital, Ankara; ^6^Pediatric Rheumatology, Health Sciences University, Dr Behcet Uz Children's Hospital; ^7^Pediatric Rheumatology, Dokuz Eylul University Faculty of Medicine, Izmir; ^8^Pediatric Rheumatology, Istanbul University, Faculty of Medicine; ^9^Senior Medical Manager, Novartis, Istanbul, Turkey

###### **Correspondencec:** B. Sozeri

**Introduction:** Systemic Juvenile Idiopathic Arthritis (SJIA) is the most acute and severe form of JIA. Despite the substantial evidence supporting the effectiveness of biologic drugs in the treatment of SIA, the clinical response to these treatment options has not been largely investigated in Turkey.

**Objectives:** TURSIS, the 24-month, multicenter, retrospective, non-interventional study aimed to assess the clinical response to biological treatments for SJIA in Turkey and provide real life data that might help improve the disease outcomes.

**Methods:** This was a retrospective and multicenter study in patients with SJIA for whom a biological treatment had been initiated during the index period. The data collection from medical records of patients evaluated at the 8 Pediatric Rheumatology Clinics in Turkey from July 2019 till December 2020. Patients’ characteristics, clinical inactivity, and safety related variables and ACR70 response were assessed.

**Results:** The study population of 147 patients consisted of 76 females (51.7%). The mean age (SD) at diagnosis and baseline were 4.25 (2.91) and 8.00 (4.99) years, respectively. The percentage of patients without corticosteroids were 27.9% at baseline and gradually increased up to Month 24 (p<0.001 for all visits). The proportion of patients with no articular involvement gradually increased from baseline to Month 6. The median (min-max; IQR) VAS was 0 (0-90;20) at Month 3. The overall functional ability of patients was good. The median (min-max) global assessment score was 7.00 (0.00- 10.00). The scores differed significantly across biologics (p=0.026). The only significant difference between the drug pairs was observed between anakinra and etanercept (mean [SD]: 6.70 [2.69] vs 5.04 [2.87]; p=0.007). During the 2-year study period, 51.0% of patients (n=75) remained on the same biologic. Sixty-two patients (42.2%) had experienced 1 switch in biologic treatment, and 10 patients (6.8%) switched twice. In patients who required switching, canakinumab was the first alternative for patients treated with anakinra (44.9%) followed by tocilizumab (14.5%). A total of 22 adverse events were observed in 12 patients (8.2%) and included one death. Thirty-two patients (22.4%) had experienced a MAS attack between the diagnosis of SJIA and baseline. Nine patients experienced MAS after baseline. At Month 3, 73.5% of patients were clinically inactive. The proportion of clinically inactive patients was highest at Month 18 (84.69%), and 45.5% of patients were reported to have ACR70 response at Month 3. ACR30, 50 and 70 responses could be achieved in 95.5%, 50% and 45.5% of patients, respectively.

**Conclusion:** This study described the patient characteristics and the impact of biologics on disease activity in a real-life study of patients with SJIA in Turkey. Overall biological therapies resulted in improvement in clinical activity early after initiation. There was a decrease in the frequency of MAS after a biological drug initiation compared to the period to the start of biological therapy. Biologics were well tolerated. Further studies with larger study size can reveal the differences between the biologics on disease outcomes and guide treatment decisions, thereby improving patient management.

**Patient Consent Received**

Yes

**Disclosure of Interest**

None declared

## e-Poster viewing: Spondyloarthritis (SpA) and enthesitis related arthritis (ERA)

### P64 Comparing patient reported outcome measures in patients with enthesitis related arthritis and ankylosing spondylitis: a single-centre study

#### R. Amarnani, M. Leandro, D. Sen, C. Fisher

##### Rheumatology, University College London Hospitals NHS Foundation Trust, London, United Kingdom

###### **Correspondence:** R. Amarnani

**Introduction:** Enthesitis related arthritis (ERA) and ankylosing spondylitis (AS) can present with similar clinical symptoms but have a distinct age of onset. Peripheral arthritis is more common in ERA, whereas axial arthritis is more common in AS. Although validated in ERA, it is not fully understood how patient reported outcome measures (PROMs) for AS such as the Bath Ankylosing Spondylitis Disease Activity Index (BASDAI) and Bath Ankylosing Spondylitis Functional Index (BASFI) perform in patients with ERA.

**Objectives:** To compare the differences in commonly used PROMs including BASDAI and BASFI in patients with a diagnosis of ERA and AS.

**Methods:** Adult patients with a diagnosis of AS or ERA under the care of the rheumatology department at University College London Hospital were included in this study. Data was collected over a 3 month period. Patients were asked to complete questionnaires of different PROMs as part of their routine clinical care. These included BASDAI, BASFI and also Global and Spinal Visual Analogue Scale (VAS), Jenkins Sleep Score (JSS), AS quality of life score (ASQOL) and the Mood & Feelings Questionnaire (MFQ). Baseline patient demographics were extracted manually from our electronic healthcare database. Data analysis was completed using IBM SPSS v26 and tested for significance using a Mann Whitney U test.

**Results:** A total of 44 patients with ERA (n=29) and AS (n=15) were included. 23% of patients were female (n=10, AS=5/15 (33.3%), ERA=5/29 (17.2%)). Those with a diagnosis of ERA were significantly younger (p<0.0001) with a median age of 21 (range 18-30) compared to 40 (range 23-65) in the AS group. A similar proportion of patients in each group were on biologic treatment (ERA 15/29 (51.7%), AS 7/15 (46.7%)), although a higher proportion of patients with ERA were treated with conventional DMARDs (ERA 23/29 (79.3%), AS 2/15 (13.3%)). There was no significant difference between total mean BASDAI in the AS group compared to the ERA group (4.4 vs 3.18, p= 0.13). Interestingly, a significant difference was found for question 2 of the BASDAI which focuses on neck, back and hip pain and patients with AS recorded higher median scores (5 vs 3, p=0.01). The mean BASFI was significantly higher in patients with AS compared to patients with ERA (3.94 vs 1.78, p=0.005). In all other PROMs, patients with AS scored significantly higher including mean scores for global VAS (5.35 vs 2.93, p=0.009), spinal VAS (5.0 vs 2.58, p=0.013), JSS (10.6 vs 4.72, p=0.003), ASQOL (9.26 vs 4.34, p=0.003) and the MFQ (6.92 vs 2.79, p=0.015).

**Conclusion:** Our results show that patients with a diagnosis of AS reported significantly worse PROMs in a wide range of domains compared to patients with ERA. Total BASDAI was the only PROM where no significant difference was found, although the higher score for question 2 in patients with AS may reflect the prominence of axial disease compared to ERA. Questions in the BASFI are also weighted towards spinal disease and may underestimate enthesitis and peripheral arthritis, resulting in lower scores in patients with ERA. However, poor levels of sleep, quality of life and low mood in patients with AS compared to those with ERA are not explained by this and need further investigation. Possible explanations include differences in disease duration and age of onset, patient age, HLA-B27 status, disease activity, early treatment with biologic medication and pattern of joint involvement. Although this study is limited to a single centre and a small cohort of patients, it identifies important differences between ERA and AS and may indicate PROMs for AS are not as sensitive in patients with ERA.

**Disclosure of Interest**

None declared

### P65 Looking beyond joints: gastrointestinal involvement in children and adolescents with juvenile idiopathic arthritis

#### F. Civitelli^1^, F. Ardenti Morini^1^, F. Soscia^1^, G. Bruno^2^, F. Ferrari^1^, V. D'Ovidio^2^, M. E. Bazuro^2^, G. Santeusanio^3^, E. Cortis^1^

##### ^1^Department of gender diseases, children and adolescents, Pediatric Unit, Sant'Eugenio Hospital; ^2^Sant'Eugenio Hospital, Endoscopy Unit; ^3^Department od Diagnostic Services, UOC of Pathology, Sant'Eugenio Hospital, Rome, Italy

###### **Correspondence:** F. Civitelli

**Introduction:** Gastrointestinal (GI) symptoms are frequent in patients (pts) with chronic disease such as juvenile idiopathic arthritis (JIA). Disease-related drugs, psychological issues due to the chronic condition should be considered as possible causes. However, gut inflammation has been described in some forms of JIA and inflammatory bowel disease (IBD) occur in 7-10 % of pts with sponidloarthropaty (SpA). Accurate assessment of GI symptoms in JIA pts with is necessary to identify those requiring a further invasive diagnostic work-up with endoscopy.

**Objectives:** To describe the presence and type of GI involvement in a population of children and adolescents with different subtypes of JIA. The secondary aim was to assess the accuracy of non-invasive tests, such as fecal calprotectin (FC) and bowel ultrasonography (BWUS), in identifying pts requiring a further diagnostic work-up with endoscopy.

**Methods:** Consecutive JIA pts complaining of GI symptoms for at least 12 weeks were prospectively enrolled during a twelve-month period. All underwent a complete clinical assessment, including evaluation by a pediatric gastroenterologist, blood inflammatory markers, FC (Calprest®, Eurospital), and BWUS with Colour-Doppler examination (Esaote equipment, 3.5 MHz convex and 12 MHz linear transducers). US assessed parameters were: Bowel Wall Thickness (BWT) > 3 mm, presence of BW vascularity, presence of enlarged mesenteric nodes and mesenteric fat hypertrophy (MFH). In pts with high clinical suspicion of IBD upper and lower endoscopy with multiple biopsies was performed. Pts who received NSAID in the 14 days before enrolment were excluded to avoid any possible effect on symptoms and FC levels. Sensitivity (SE), specificity (SP), positive and negative predictive value (PPV and NPV) of laboratory and US parameters were analysed according to the final diagnosis of gut inflammation.

**Results:** 32 pts (16 female) aged 5-16 years (median 11) were enrolled: 11 with oligoarthritis, 6 polyarthritis, 2 systemic, 10 SpA, 3 undefined. Eight pts were receiving methotrexate, 7 sulfasalazine, 10 anti-TNFα therapy, 1 canakinumab, 1 tocilizumab. All pts presented abdominal pain, 12 complained of diarrhoea, 7 also with blood in the stools, 2 failure-to-thrive and 2 recurrent oral aphthosis. 14 pts (43%) showed a first-degree relative with Rheumatoid Arthritis (8) or IBD (6). The mean time between the diagnosis of JIA and clinical evaluation (months) was 20.5 ± 19.4. BWUS revealed increased BWT of the terminal ileum in 17 pts, increased BW vascularity in 13, enlarged mesenteric nodes and MFH in 8. Sixteen pts underwent endoscopy: 14 showed gut inflammation, 5 had a final diagnosis of IBD, 9 showed chronic aspecific inflammation at histology requiring a further GI follow-up. One pt had celiac disease, 15 were finally diagnosed as irritable bowel syndrome based on Rome IV criteria. Pts with gut inflammation at histology had higher FC levels (200 ± 190 ug/g vs 48 ± 25, p<0.001) and higher BWT at US (4 ± 0.5 vs 3.2 ± 0.7, p< 0.05 ) and a more frequent family history (10 vs 4, p=0.4). SE, SP, PPV, NPV of BWT and FC > 50 ug/g vs the final diagnosis of gut inflammation were (%): 92.8, 85.7, 81.2, 94.3, and 86.7, 81.2, 76.5, 90, respectively

**Conclusion:** Pts with JIA and GI symptoms should be managed together by the pediatric rheumatologist and the pediatric gastroenterologist. FC and BWUS may be useful non-invasive tests to identify pts requiring a additional diagnostic workup with endoscopy. Further studies with larger number of pts are needed to assess the usefulness of non-invasive test in detecting sub-clinical gut inflammation also in asymptomatic pts with JIA.

**Patient Consent Received**

No

**Disclosure of Interest**

None declared

### P66 The challenge of classification in juvenile spondyloarthritis

#### M. Katsicas, M. Bertinotti, G. Villarreal

##### Immnulogy& Rheumatology, Hospital De Pediatría Garrahan, Buenos Aires, Argentina

###### **Correspondence:** M. Katsicas

**Introduction:** Juvenile spondyloarthropathies (JSpA) are a group of rheumatic diseases that involves sacroiliitis and spondylitis, peripheral large joints, and enthesitis. JSpA are represented by enthesitis related arthritis (ERA), juvenile psoriatic arthritis and undifferentiated arthritis (UA) in the ILAR criteria. The proposed PRINTO classification criteria for JIA introduced a new approach of axial imaging changed ERA by enthesitis/spondylitis-related JIA (ESRA).

**Objectives:** To assess the sensibility and specificity of the provisional new PRINTO JIA classification criteria ESRA for identification of patients with JSpA To compare the performance of ESRA and ASAS classification criteria.

**Methods:** Consecutive patients with JSpA (defined as ERA, or UA according to ILAR) followed in our center with complete records were included. Randomly selected patients with oligoarthritis, systemic arthritis and polyarthritis RF negative served as controls. Variables recorded were: arthritis, enthesitis, tarsitis, inflammatory back pain, sacroiliac joint tenderness, presence of HLA-B27 antigen, acute anterior uveitis, history of SpA in a first-degree relative. Imaging on sacroiliac joints. Summary statistics included overall sensitivity, specificity, positive predictive value (PPV), negative predictive value (NPV), positive Likelihood ratio (+LR).

**Results:** 103 patients with JSpA (84 ERA, 19 UA) were included (M:89), median age at onset: 10(1-15) years, disease duration at time of first visit 10 (1-48) months, follow-up time 6 (1-15) years. Controls: 69 patients with JIA (25 oligoarthritis, 24 polyarthritis RF negative, 20 systemic) (M:27)***.*** At first visit cases showed: 100 (97%) arthritis, 64(62%) elevated CRP, 52 (50%) limitation of lumbar spine motion, 52 (50 %) HLA-B27, 46 (45%) enthesitis, 40(39%) tarsitis, 39(38%) low back pain, 31 (30%) sacroiliac joint tenderness, 30 (29%) good response to NSAIDS, 26 (25%) positive family history, 13 (13%) dactylitis, 7(7%) uveitis,7(7%) diarrhea, 5 (5 %) infectious previous disease, 2(2%) urethritis, 2(2%) inflammatory bowel disease, 2 (2%) buttock pain. Imaging criterion for sacroiliitis: 28 (27%) on X-Ray (21 grade 2 bilateral and 7 grade 3), 26 (25%) on magnetic resonance imaging (MRI). At first visit (103 patients): 90 (87%) fulfilled ESRA criteria. During disease course (97patients): 92 (95%). Axial radiological arm allowed to classify 6 UA patients at baseline and others 7 UA patients could be reclassified during the follow-up. Table shows accuracy PRINTO and ASAS classification criteria

**Table:** 

First Visit	Sensitivity	Specificity	PPV	NPV	+LR
PRINTO ESRA	87%	86%	90%	81%	6.31
Peripheral ASAS	74%	86%	89%	68%	5.39
Axial ASAS	24%	86%	73%	42%	1.75
Global ASAS	77%	86%	89%	70%	5.53
Disease Course					
PRINTO ESRA	95%	86%	91%	92%	6.85
Peripheral ASAS	92%	86%	91%	87%	6.66
Axial ASAS	40%	86%	82%	48%	2.94
Global ASAS	92%	86%	91%	87%	6.66

**Conclusion:** In our cohort ESRA provides a wider clinical and imaging spectrum by adding some UA. ESRA showed better accuracy than ASAS. The validation of these provisional criteria are expected to be used in clinical practice

**Disclosure of Interest**

None declared

### P67 The performances of the ILAR and ASAS classification criteria in JIA patients

#### U. Kaya Akca, E. D. Batu, S. Sener, Z. Balik, M. Kasap Cuceoglu, E. Atalay, O. Basaran, Y. Bilginer, S. Ozen

##### Department of Pediatrics, Division of Rheumatology, Hacettepe University Faculty of Medicine, Ankara, Turkey

###### **Correspondence:** U. Kaya Akca

**Introduction:** Enthesitis-related arthritis (ERA) is considered the equivalent of ankylosing spondylitis in adults albeit with certain significant differences. In our routine practice, the International League of Associations for Rheumatology (ILAR) classification criteria are used in children with ERA and the Assessment of Spondyloarthritis International Society (ASAS) classification criteria for axial and peripheral spondyloarthritis (SpA) in adults.

**Objectives:** We aim to evaluate the sensitivity and specificity of ILAR and ASAS classification criteria in ERA patients.

**Methods:** The medical records of ERA patients who were followed up in the Department of Pediatric Rheumatology at Hacettepe University between January 2005 and September 2020 have been analyzed. The control group consisted of patients with oligoarticular JIA (n=146), polyarticular JIA (n=55), and psoriatic arthritis (n=20). Patients followed up for at least 6 months were included. The diagnosis of ERA was based on expert opinion.

**Results:** This retrospective study included 108 ERA (26.9% female) and 221 control patients (74.2% female). The median age at diagnosis for ERA and control patients were 12.5 and 4.0 years, respectively (p<0.001). Arthritis was observed more frequently in the control group at diagnosis and follow-up (p<0.001 for both), while enthesitis, sacroiliac joint tenderness, and inflammatory back pain were more common in the ERA group both at diagnosis and follow-up (p<0.001 for all). The presence of human leukocyte antigen (HLA)-B27 and elevated C-reactive protein levels were also more frequent in the ERA group. In sacroiliac imaging, 70.1% of ERA patients had positive findings suggestive of sacroiliitis at diagnosis and 78% at follow-up. There was a low response rate to non-steroidal anti-inflammatory drugs in the ERA group compared to the control group. The sensitivities of ILAR and ASAS criteria for axial SpA, and peripheral SpA at diagnosis were 73.1%, 21.3%, and 83.3%, respectively which increased to 83.3%, 35.1%, and 92.5%, respectively at follow-up. The specificity of all three classification criteria was >90% at diagnosis and follow-up (Table 1).

**Conclusion:** The ASAS criteria for peripheral SpA was the most sensitive while ASAS classification criteria for axial SpA was the most specific criteria in ERA patients. The sensitivities of all three classification criteria has increased during follow-up, which is probably due to the additive nature of the disease.

**Disclosure of Interest**

None declared

**Table 1 (abstract P67).** Comparison of the sensitivity and specificity of the ILAR criteria, ASAS classification criteria for axial and peripheral SpA at diagnosis and follow-up

Criteria set	Sensitivity at diagnosis (%)	Specificity at diagnosis (%)	Sensitivity at follow-up (%)	Specificity at follow-up (%)
ILAR classification criteria	73.1	98.6	83.3	98.6
ASAS classification criteria for axial SpA	21.3	99.1	35.1	99.1
ASAS classification criteria for peripheral SpA	85.1	90.9	92.5	90.9

### P68 Quality of life of patients with ankylosing spondylitis with anemic syndrome among ukrainian population

#### O. Zviahina, S. Shevchuk

##### Internal Medicine #2, National Pirogov Memorial Medical University, Vinnytsya, Ukraine, Vinnytsya, Ukraine

###### **Correspondence:** O. Zviahina

**Introduction:** Anemic syndrome is one of the manifestations of ankylosing spondylitis (AS). Anemia in this cohort of patients may be, on the one hand, a completely independent disease, as well as a complication of both the underlying disease and the consequence of the pharmacotherapy of AS. Anemia often complicates the course and prognosis of the disease, which in turn impairs the quality of life (QOL) of this group of patients. This topic is being researched for the first time among the Ukrainian population of AS patients.

**Objectives:** The purpose of this work was to evaluate QOL in patients with AS and its relationship with the presence and type of anemic syndrome.

**Methods:** The study included 118 patients diagnosed with ankylosing spondylitis according to the modified New York criteria. All patients were divided into three groups: 84 patients with AS without anemia, 34 patients with AS and anemia, and the control group consisting of 26 almost healthy persons. All hematologic, biochemical and enzyme-linked immunosorbent assays were performed to determine the indicators of hematopoiesis and ferrokinetics. AS activity assessment was performed using ASDAS (Ankylosing Spondylitis Disease Activity Score) and BASDAI (Bath ankylosing spondylitis disease activity index) questionnaires. QOL was evaluated using the SF-36 questionnaire. Statistical processing of the results was performed using a set of statistical programs "Microsoft Office Excel 2007".

**Results:** The QOL score according to the SF-36 questionnaire in the control group was equal to 96.41 ± 0.25 points, and in patients with AS – 42.2 ± 1.01 points. The mean rate of QOL in patients with AS without anemia was significantly different from patients with AS with anemia (44.65 ± 1.18 versus 36.13 ± 1.51, respectively (p <0.05). Analysis of QOL in patients with different pathogenetic types of anemia syndrome showed that the highest rate of QOL according to SF-36 was among patients with IDA and was 42,36 ± 3,39, which was significantly higher compared to the groups of patients with ACD and ACD with iron deficiency (p <0.05). SF-36 in the group of patients with ACD was 34.62 ± 1.48 points, and in persons with ACD with iron deficiency 31.98 ± 2.91 points. It should be noted that the physical component of QOL suffered more than the psychic component. In the group of patients with IDA, the physical component of health according to SF-36 was 27.71 ± 4.89 points, in the group with ACD - 22.81 ± 1.64 points, in the group with ACD and iron deficiency - 23,95 ± 3,27 points. Mental component in the group of patients with IDA was 57,04 ± 4,31 points, and was significantly higher than in the group with ACD - 46,48 ± 2,26 and ACD with iron deficiency - 40.05 ± 2.98 (p <0.05). Analysis of the subscales of the SF-36 questionnaire revealed no significant differences between the groups, except for the “Role limitations due to emotional problems” indicator, which was significantly better in the group of patients with IDA compared with patients with ACD and ACD with iron deficiency. In our opinion, this difference between the groups is explained by the fact that the significantly lower value of BASDAI and ASDAS was in particular among patients with IDA (5.40 ± 0.46 points and 3.95 ± 0.29 points, respectively), whereas ACD (7.42 ± 0.25 points and 4.44 ± 0.11 points) and ACD with iron deficiency (6.76 ± 0.38 points and 4.04 ± 0.15 points) were characterized by higher rates of disease activity (p < 0.05).

**Conclusion:** QOL in patients with AS is lower, compared with a relatively healthy population. Anemic syndrome in patients with AS is associated with worse QOL according to SF-36. ACD and ACD with iron deficiency are characterized by lower rates of QOL than patients with IDA.

**Trial registration identifying number:** ClinicalTrials.gov: NCT03926195

**Patient Consent Received**

Yes

**Disclosure of Interest**

None declared

## e-Poster viewing: Autoinflammatory diseases

### P69 The rare autoinflammatory disease manifesto: a key educational tool to help raise awareness and improve care

#### S. Angevare^1^, B. Bori^2^, A. Runov^3^, M. Nudel^4^, C. Normand^5^, L. Bergamini^6^, M. Jesenak^7^, L. Fagerhed^8^

##### ^1^KAISZ, Amersfoort, Netherlands; ^2^Novartis, Origgio, Italy; ^3^Sunflower Foundation, Moscow, Russian Federation; ^4^Mifrakim Tz’eirim, Haifa, Israel; ^5^ENCA, Geneva, Switzerland; ^6^Spedali Civili, Brescia, Italy; ^7^Centre for Periodic Fever Syndromes, Department of Paediatrics and Department of Pulmonology and Phthisiology, Jessenius Faculty of Medicine in Martin, Comenius University in Bratislava, University Hospital in Martin, Bratislava, Slovakia; ^8^Novartis, Espoo, Finland

###### **Correspondence:** S. Angevare

**Introduction:** Rare autoinflammatory diseases are an emerging and rapidly evolving group of rare conditions characterised by spontaneous and disabling attacks of systemic inflammation, resulting in a broad spectrum of symptoms and consequences, including recurring fevers and severe fatigue.^1-6^ The Rare Autoinflammatory Disease Manifesto was developed in collaboration with the Rare Autoinflammatory Disease Council (a group of patients, patient advocacy groups [PAGs] and healthcare professionals [HCPs]) to define how rare autoinflammatory diseases are discussed and align the community voice.

**Objectives:** The aim of the manifesto is to explore the unmet needs of people living with rare autoinflammatory diseases and identify a call to action for key aspects of the patient journey, in order to improve patient care.

**Methods:** Extensive research was conducted to gain a deeper understanding of rare autoinflammatory diseases and the patient journey, including disease burdens and unmet needs. Primary research included two virtual advisory board meetings in May and July 2020 with members of the Rare Autoinflammatory Disease Council and a physician specialising in periodic fevers to discuss their experiences. A literature review was performed to supplement the primary research.

**Results:** The manifesto clarifies key scientific aspects of rare autoinflammatory diseases including disease definition, categorisation and genetic background. It reports significant physical, emotional, social and financial burdens on people living with these conditions. Notably, the rarity and low awareness of disease results in substantial delays to appropriate patient care, and a lack of understanding and support from their social environment. We found that paediatric patients are especially impacted as symptoms disrupt physical, educational and social development. The transition to adult care is considered one of the most challenging times. The lifelong nature of these conditions places a substantial economic burden on healthcare systems. Early diagnosis, increasing therapy options, optimising disease management and facilitating patient engagement and empowerment were identified as key strategies to overcome the barriers to effective care. Based on these goals, the manifesto outlines actions targeted towards HCPs, policymakers, PAGs and other stakeholders to help improve care and health outcomes.

**Conclusion:** The Rare Autoinflammatory Disease Manifesto is an important educational resource that was published on the Periodic Fevers website (www.periodicfevers.com/about-periodic-fevers/manifesto) in March 2021 and has since been translated in six languages. A multi-channel campaign is underway to promote the manifesto internationally. Our efforts are focused on continued collaboration with PAGs to disseminate and evolve the manifesto as we uncover further insights from the rare autoinflammatory community.

**References**

1.Erbis G, Schmidt K, Hansmann S, Sergiichuk T, Michler C, Kuemmerle-Deschner JB, et al. Living with autoinflammatory diseases: identifying unmet needs of children, adolescents and adults. Pediatr Rheumatol Online J. 2018;16(1):81. Available from: https://www.ncbi.nlm.nih.gov/pmc/articles/PMC6302479

2.Savic S, Wood P. Does this patient have periodic fever syndrome? Clin Med. 2011;11(4):396–401. Available from: https://www.ncbi.nlm.nih.gov/pmc/articles/PMC5873756

3.Gurcay E, Akinci A. Autoinflammatory Diseases and Physical Therapy. Mediterr J Rheumatol. 2017;28(4):183–91. Available from: https://www.ncbi.nlm.nih.gov/pmc/articles/PMC7046004

4.Touitou I, Galeotti C, Rossi-Semerano L, Hentgen V, Piram M, Kone-Paut I, et al. The expanding spectrum of rare monogenic autoinflammatory diseases. Orphanet J Rare Dis. 2013;8:162. Available from: https://ojrd.biomedcentral.com/articles/10.1186/1750-1172-8-162

5.Krainer J, Siebenhandl S, Weinhausel A. Systemic autoinflammatory diseases. J Autoimmun. 2020;109:102421. Available from: https://pubmed.ncbi.nlm.nih.gov/32019685

6.McDermott MF, Aksentijevich I, Galon J, McDermott EM, Ogunkolade BW, Centola M, et al. Germline mutations in the extracellular domains of the 55 kDa TNF receptor, TNFR1, define a family of dominantly inherited autoinflammatory syndromes. Cell. 1999;97(1):133–44. Available from: https://pubmed.ncbi.nlm.nih.gov/10199409

**Patient Consent Received**

No

**Disclosure of Interest**

S. Angevare: None declared, B. Bori Employee of: Novartis, A. Runov: None declared, M. Nudel: None declared, C. Normand: None declared, L. Bergamini Consultant for: Novartis, M. Jesenak Speaker Bureau of: Novartis, SOBI, Sanofi Genzyme, CSL Behring, Takeda, GlaxoSmithKline, L. Fagerhed Employee of: Novartis

### P70 Assessment of physical fitness with fitnessgram® and physical activity in children and adolescents with familial mediterranean fever: a pilot study for health risk analysis

#### A. Albayrak^1^, N. Arman^2^, E. Tarakci^2^, O. Kasapcopur^3^

##### ^1^Master Program of Physiotherapy-Rehabilitation, Graduate Education Institute, Istanbul University-Cerrahpasa; ^2^Department of Physiotherapy and Rehabilitation, Istanbul University-Cerrahpasa, Faculty of Health Science; ^3^Department of Pediatric Rheumatology, Cerrahpasa Medical School, Istanbul University-Cerrahpasa, Istanbul, Turkey

###### **Correspondence:** N. Arman

**Introduction:** Familial Mediterranean Fever (FMF) is the most common inherited autoinflammatory disease, which is characterized by recurrent fever attacks, inflammation of serosal membranes and arthritis mostly occurring in childhood. Although the clinical findings during attacks may vary, the most common attack type is a combination of fever, abdominal pain, and joint findings. Children and adolescents with FMF may also have symptoms not related to attacks, such as prolonged myalgia, exertional leg pain, post-exercise erythema, and sacroiliitis.

**Objectives:** The aim of the study was to assess physical fitness and physical activity in children and adolescents with FMF.

**Methods:** Eleven patients with FMF were included in the study. Health-related components of physical fitness were evaluated using the FitnessGram® Test Battery. These tests included curl-ups, push-ups, a 20-m shuttle run test (20mSRT), trunk lift test, the modified back-saver-sit-and-reach test (mBSRT) and body composition measurements. The curl-up and push-up tests were used to evaluate muscular strength and endurance. The 20mSRT was used to evaluate cardiorespiratory fitness. The trunk lift test was used to evaluate trunk extensor strength. mBSRT was used to evaluate flexibility. The percent of body fat with Bioelectrical Impedance Analysis and Body Mass Index (BMI) were used to evaluate body composition. All tests were conducted according to the FitnessGram® measurement procedures. Participants were then classified according to the age and sex-specific cut-off points of FitnessGram as Needs Improvement (NI)-health risk, NI and healthy fitness zone (HFZ). The Physical Activity Questionnaire (PAQ) was used to evaluate the physical activity levels of patients.

**Results:** Four of the participants were male and 7 of them were female. The mean age was 13.54±3.69 and the mean disease duration was 6±3.90 years. When the FitnessGram standards were applied, %63.6 of them were categorized in the NI and %36.4 of them in the HFZ for curl-up test, %72.7 of them in the NI and %27.3 in the HFZ for the push-up test, %90.9 of them in the NI-health risk, %9.1 of them in the NI for the 20mSRT, %72.7 of them in the NI and %27.3 of them in the HFZ for the trunk lift test, %81.8 of them in the NI and %18.2 of them in the HFZ for the mBSRT. Besides, according to FitnessGram body composition classification criteria, %27.3 of them were categorized in the NI, %27.3 of them in the very lean and %45.5 of them in the HFZ for percent body fat, %9.1 of them in the NI-health risk, %18.2 of them in the NI, %9.1 of them in the very lean and %63.6 of them in the HFZ for BMI. The physical activity level of the patients was categorized “low-active” in %81.8 of them and “sufficiently active” in %18.2 of them, according to the PAQ.

**Conclusion:** The results of this pilot study reveal that the physical fitness and physical activity levels in children and adolescents with FMF were considerably poor at a risk level for health. However, further research on physical fitness and physical activity in childhood FMF with larger sample sizes is required. We consider that physical activity and exercise programs should focus on improving physical fitness and increasing physical activity levels to minimize health risks in childhood FMF.

**Patient Consent Received**

Yes

**Disclosure of Interest**

None declared

### P71 Pediatric chronic non-bacterial osteomyelitis: a single-center case series

#### P. O. Avar-Aydin^1^, B. Ozcakar^1^, N. Cakar^1^, D. Karakas^1^, S. Fitoz^2^, F. Yalcinkaya^1^

##### ^1^Department of Pediatric Rheumatology; ^2^Department of Pediatric Radiology, Ankara University Faculty of Medicine, Ankara, Turkey

###### **Correspondence:** P. O. Avar-Aydin

**Introduction:** Chronic nonbacterial osteomyelitis (CNO) is an autoinflammatory bone disorder that most commonly affects children and adolescents and covers a wide clinical spectrum from unifocal involvement and nonrecurrent course to recurrent or persistent multifocal disease. CNO remains a diagnosis of exclusion.

**Objectives:** To present a single-center experience of pediatric CNO patients.

**Methods:** Children with a diagnosis of CNO who were followed up at the Department of Pediatric Rheumatology of Ankara University School of Medicine between 2013 and 2021 were included in this study. Patient data was documented from electronic medical records retrospectively. The clinical score by Jansson et al. was used to classify patients. Patients without remission despite treatment for 6 months were defined to have a persistent course.

**Results:** A total number of 24 patients with a median follow-up duration of 32.5 months (0-84) were included. Clinical and laboratory features are summarized in Table 1. Co-existing diseases were as follows: familial Mediterranean fever (n:4), psoriasis and severe acne (n: 2), Crohn’s disease (n:1), and juvenile idiopathic arthritis (n: 1). A family history of autoinflammatory diseases was present in 4 patients (16.7%).

All patients had multifocal bone lesions at diagnosis. The most affected bones were the pelvis (58.3%), femur (50%), and vertebra (50%). Long bone lesions were metaphyseal in 92.9% of the cases. Increased CRP levels were present in 70.8% and antinuclear antibody positivity in 16.7% of the patients. Patients with sacroiliitis were screened for the presence of HLA-B27 and one patient was found positive.

More than 50% of the patients with a follow-up of more than 6 months had a persistent course who experienced remission after anti-TNF agents. All patients with two mutations in the MEFV gene (n: 4) benefitted from concurrent use of colchicine and anti-TNFs.

**Conclusion:** Chronic nonbacterial osteomyelitis presents with a variety of clinical findings and has a variable disease course. NSAIDs are effective in treatment; however, persistent or recurrent disease activity may need biologics and other therapies.

**Patient Consent Received**

Yes

**Disclosure of Interest**

None declared

**Table 1 (abstract P71).** Demographic, Clinic, and Laboratory Features of Patients with CNO

Characteristics	Patients (N: 24 (% of N) or median (min-max))
Male gender	16 (66.7)
Age at disease onset, years	9.75 (1.50-16.00)
Age at diagnosis, years	11.04 (3.50-16.17)
Total clinical score	40.00 (26.00-54.00)
Normal blood cell count	17 (70.8)
Presence of MEFV mutation (n: 22)	7 (31.8)
Bone biopsy and/or bone marrow aspiration	16 (66.7)
Whole-body imaging findings	
Total number of lesions	6.50 (2.00-17.00)
Multifocal lesions	24 (100)
Symmetric lesions	18 (75)
Lower extremity lesions	22 (91.7)
Vertebral lesions	12 (50)
Sacroiliitis	9 (37.5)
Treatment	
NSAID	24 (100)
Corticosteroids	6 (25)
Colchicine	6 (25)
Biphosphonate	4 (16.7)
Methotrexate	2 (8.3)
Anti-TNF agents	8 (33.3)

### P72 Probiotic use in the prophylaxis of PFAPA (Periodic fever, adenitis, pharyngitis, aphthous stomatitis) syndrome

#### E. D. Batu, U. Kaya Akca, M. Kasap Cuceoglu, E. Atalay, Z. Balık, S. Sener, O. Basaran, Y. Bilginer, S. Ozen

##### Department of Pediatrics, Division of Rheumatology, Hacettepe University Faculty of Medicine, Ankara, Turkey

###### **Correspondence:** E. D. Batu

**Introduction:** Periodic fever, aphthosis, pharyngitis, and adenitis (PFAPA) syndrome is an autoinflammatory recurrent fever syndrome which mainly affects children. Cimetidine, colchicine, and anti-IL-1 agents could be used to prevent attacks. Probiotics were previously reported to be beneficial in a few PFAPA patients for prevention of attacks.

**Objectives:** We aimed to evaluate the response to probiotics in PFAPA patients.

**Methods:** Patients with PFAPA syndrome who received probiotics were included in this retrospective study. Demographic and clinical features, and response to probiotics were assessed.

**Results:** Twenty patients with PFAPA syndrome (F/M:1) were included. All had pharyngitis during attacks while oral aphthosis (65%), lymphadenitis (50%), and fatigue (80%) were also common attack-associated features. 9 (45%) patients also had abdominal pain during attacks. Periodicity was present in 15 (75%) patients. Throat culture was performed during an attack in 16 (80%) patients and revealed normal flora in all. *MEFV* variant analysis (n=17) revealed heterozygosity for M680I in 3, M694V in 2, and E148Q in 3 patients. The median (min-max) ages at symptoms onset and diagnosis were 24 (3-72) and 51.5 (11-120) months, respectively. All patients received probiotics during the disease course. The probiotic they used included a combination of two lactobacilli as *Lactobacillus plantarum* HEAL9 (Lp HEAL9) and *Lactobacillus paracasei* 8700:2 (Lpa 8700:2). The median age at probiotic onset was 60 (33-192) months while duration of probiotic use was 4.5 (3-19) months. All patients except one experienced decrease in attack frequency with probiotic use. The attack frequency decreased significantly with probiotics (median number of attacks per 3 months: 3 vs. 1, respectively; p<0.001). 8 (40%) patients had no attacks during the 3-months period after probiotic initiation. And, 5 (42%) of 12 patients who had ≥1 attacks on probiotics mentioned that the attack severity (duration and the degree of fever) decreased significantly during probiotic use. Colchicine was also administered to 4 (20%) patients for a duration of 30 (6-55) months before the onset of probiotics. Two of them did not respond to colchicine and colchicine was discontinued. Other two patients experienced decrease in attack frequency with colchicine treatment; however, they were still having >1 attack/month. Probiotic was beneficial in all four of these patients.

**Conclusion:** Intake of the probiotic strains Lp HEAL9 and Lpa 8700:2 were previously shown to decrease the incidence and severity of common cold in randomized controlled trials. A significant reduction in pharyngeal symptoms was also noted in these studies. We may speculate that these lactobacilli might be beneficial in PFAPA syndrome by down-regulating the inflammatory response in tonsils through changes in the microbiota.

**References**

Bergggren et al. Randomised, double-blind, and placebo-controlled study using new probiotic lactobacilli for strengthening the body immune defence against viral infections. Eur J Nutr 2011;50:203-10.

Bush et al. Randomised, double-blind, and placebo-controlled study using a combination of two probiotic lactobacilli to alleviate symptoms and frequency of common cold. Food and Nutrition Sciences 2013;4:13-20

Francesco et al. The use of *Streptococcus salivarious K12* in attenuating PFAPA syndrome; a pilot study. Altern Integr Med 2016;5:4.

**Patient Consent Received**

Yes

**Disclosure of Interest**

None declared

### P73 Long term safety profile of anti IL-1 therapy in patient with autoinflammatory childhood onset disease

#### M. Camacho-Lovillo, L. Fernandez-Silveira, M.-I. Garcia Ruiz-Santa Quiteria, A. Capilla Miranda, O. Neth

##### Hospital Virgen Del Rocio, Sevilla, Spain

###### **Correspondence:** M. Camacho-Lovillo

**Introduction:** There are currently two IL-1 inhibitors approved for clinical use in Europe. However, long term follow-up data of five or more years regarding security and efficacy of anti IL-1 treatment in autoinflammatory diseases (AID) in children are scarce.

**Objectives:** A retrospective study of patients with AID diagnosed in a tertiary referral pediatric rheumatology department receiving anti IL-1 therapy for 5 years or longer was conducted. Demographic data, symptoms, treatments and adverse events (AE) were collected from clinical chart, not only from hospital but also from primary attention in order to improve information quality.

**Methods:** A retrospective study of patients with AID diagnosed in a tertiary referral pediatric rheumatology department receiving anti IL-1 therapy for 5 years or longer was conducted. Demographic data, symptoms, treatments and adverse events (AE) were collected.

**Results:**

**Table:** 

Patient	1 male	2 male	3 male	4 male	5 female	6 male	7 male	8 male	9 female
Current Age (y.o)	19	23	21	13	18	17	12	22	11
Diagnosis	JIAs	JIAs	Recurrent pericarditis	JIAs	FMF	JIAs	Recurrent pericarditis	JIA s	CINCA
Genetic test	No mutation found	No mutation found	No mutation found	No mutation found	V267A heterozygosis in MEVF gene	No mutation found	No mutation found	pI591T heterozygosis in MEVF gene	p. E387G in NLRP3 gene
Diagnosis age (years)	6	7	12	5	4	6	7	5	4
Years with anti IL-1 treatment	13	15	9	7	11	10	5	10	7
Starting anti IL-1 therapy date	2008 May	2006 April	2009 Nov	2013 March	2007 Sept	2009 Sept	2016 May	2010 Jan	2014 Dec
Stop anti IL-1 therapy date (cause)	-	-	2018 Lost Follow-up	2020 october (remission)	2018 february (remission)	2019 september (remission)	-	2020 january (inefficacy)	-
Current Treatment	Anakinra100 mg/ 48 h	Anakinra 100 mg/ 24 h	Anakira 100 mg / 24 h	-	-	-	Anakinra 100 mg /48 h	Tocilizumab	Canakinumab150 mg/ 4 w
Adverse events during anti IL-1 therapy	Nephrolithiasis, Gastroenteritis, Flu, sinusitis	vertebral fracture	Intracraneal hypertension cataract		Varicella, pneumonia				Mild covid disease, lysteriosis

We identified a total of 9 patients, 8 who received anakinra and one canakinumab. 5 diagnosed with systemic juvenile idiopathic arthritis, 2 recurrent pericarditis, one familial Mediterranean fever with renal and skin vasculitis and one with cryopyrin-associated periodic syndromes. 7/9 were males. Patients are currently 11 to 23 years old; mean disease duration being 6.2 years (range 4-12 y) and a mean therapy duration of 9.7 years (range 5-15 y). No laboratory abnormalities were detected. No malignancy or macrophage activation syndrome was observed, no fatalities occurred. Although infections were uncommon, 4 infectious episodes required admission, importantly no opportunistic infections were diagnosed. Other side effects were a vertebral fracture, cataract and an intracranial hypertension, probably related to steroid therapy. 4 patients are currently receiving antiIL-1 therapy as mono therapy only, all 4 being in full clinical remission not needing of steroid therapy. To date only 3 patients stopped treatment due to inactive disease without occurrence of flares.

**Conclusion:** In our experience long-term use anti IL-1 therapy appears to be safe and effective in pediatric AID. Early treatment initiation can avoid prolonged corticoids treatment thereby reducing secondary adverse events.

**Disclosure of Interest**

None declared

### P74 Through the development of a composite score to assess disease activity in recurrent fevers

#### R. Caorsi^1^, V. Hentgen^2^, I. Gueli^3^, C. Castellano^3^, C. Matucci Cerinic^3^, M. P. Teodoro^3^, A. Consolaro^1,4^, A. Ravelli^5^, I. Kone-Paut^6^, M. Gattorno^1^, N. Ruperto^7^

##### ^1^Clinica pediatrica e reumatologia, IRCCS Istituto Giannina Gaslini, Genova, Italy; ^2^National Referral Centre of Auto-Inflammatory Diseases and inflammatory amyloidosis, Versailles Hospital, Le Chesnay (Paris), France; ^3^Università degli studi di Genova, Genova, Italy; ^4^DiNOGMI, Università degli studi di Genova; ^5^IRCCS Istituto Giannina Gaslini, Genova, Italy; ^6^National Referral Centre of Auto-Inflammatory Diseases and inflammatory, University of Paris Sud, le Kremlin Biĉetre, France; ^7^Clinica pediatrica e reumatologia, PRINTO, IRCCS Istituto Giannina Gaslini, Genova, Italy

###### **Correspondence:** R. Caorsi

**Introduction:** Recurrent Fevers syndromes are autoinflammatory diseases characterized by febrile episodes associated with systemic symptoms and elevation of acute phase reactants; many of these diseases can present long-term complications, which can be avoided by an appropriate therapeutic approach. In light of this, monitoring of disease activity over time is mandatory.

Currently, the available tools to assess disease activity in recurrent fever syndrome take into account the clinical manifestations of the diseases and not the laboratory parameters, that nowadays are clearly important in the definition of disease activity.

**Objectives:** The purpose of the study is the creation of a composite score for the disease activity of autoinflammatory syndrome characterized by recurrent fever: FMF, TRAPS, MKD, PFAPA and systemic undefined recurrent fever (SURF).

**Methods:** the project is divided into two main phases: the first dedicated to the selection of variables of the score, the second intended for the validation of the latter.

In the first phase, a literature review was carried out to search for the parameters defining the disease activity. At the same time, through the Delphi method, a questionnaire was proposed to the main experts of Recurrent Fever, in which they were asked to list the variables used in the daily clinical practice to assess the disease activity of their patients. Finally, the same question was asked to the families of patients suffering from these conditions. In the second round of the Delphi Survey expert of the diseases and family representatives were ask to select and rank the 10 variables the think to be most effective for the purpose.

**Results:** by the review of the literature, 3005 articles were taken into consideration, of those 90 presented parameters defining the disease activity. 54 patients/families responded to the questionnaire. 114 experts were contacted, of those 95 reply to the first Delphi survey. From the three parallel searches, a list of different variables (clinical, laboratory, instrumental and other) was obtained; repetitions and redundancies were deleted and a final list of 147 parameters was obtained. The second round of the Delphi survey is actually ongoing.

**Conclusion:** the work lays the foundations for the creation of a composite score for the assessment of disease activity in patients with recurrent fever. We hope that this tool will be of help not only in the daily clinical practice, but also in future trials and experimental studies.

**Disclosure of Interest**

None declared

### P75 Serum protein signatures differentiate paediatric autoimmune/inflammatory disorders

#### E. Carlsson^1^, A. Midgley^1^, S. Perkins^2,3^, E. Caamano-Gutierrez^2,3^, J. F. Gritzfeld^1^, M. W. Beresford^1,4,5^, C. M. Hedrich^1,4,5^

##### ^1^Department of Women's and Children's Health, Institute of Life Course and Medical Sciences; ^2^Computation Biology Facility, Technology Directorate, Faculty of Health and Life Sciences; ^3^Department of Biochemistry and Systems Biology, Institute of Systems, Molecular and Integrative Biology, Faculty of Health and Life Sciences, University of Liverpool; ^4^Department of Rheumatology; ^5^National Institute for Health Research Alder Hey Clinical Research Facility, Alder Hey Children's NHS Foundation Trust Hospital, Liverpool, United Kingdom

###### **Correspondence:** E. Carlsson

**Introduction:** Because of their rarity, limited awareness among non-specialists, and significant overlaps in their clinical presentation, autoimmune/inflammatory conditions represent a diagnostic and therapeutic challenge in paediatrics. Juvenile idiopathic arthritis (JIA), with its 7 sub-forms, is the most common paediatric “rheumatic” disease. Juvenile-onset systemic lupus erythematosus (jSLE) is a severe autoimmune/inflammatory disease that can affect any organ system and shares clinical features with JIA.

**Objectives:** To overcome challenges around diagnostic approaches in the context of clinical overlap, we aimed to define disease sub-types through specific cytokine and chemokine profiles.

**Methods:** Serum samples from patients with JIA (n=77) and jSLE (n=48), as well as healthy controls (n=30), were collected. Samples were analysed using the Meso Scale Discovery (MSD) U-PLEX Biomarker Group 1 (hu) panel. The antibody-set contains biotinylated capture antibodies and corresponding detection antibodies for 71 cytokines and chemokines involved in multiple biological processes.

**Results:** Differential serum protein levels were investigated across disease groups and healthy controls. Significant differences were seen in several proteins, many of which have previously been implicated in the pathogenesis of JIA and/or jSLE. Partial least squares discriminant analysis (PLSDA) models of two components were trained to discriminate between samples from healthy controls, JIA or jSLE patients. This bioinformatic model allowed discrimination between the three groups with ~90% accuracy. Variable importance in projection (VIP) scores of the model detailing the contribution of each feature (i.e. protein) towards each of the components in the PLSDA model were used to predict potential biomarkers. The top VIP proteins included IL-23, MIP-1β, MCP-1 and M-CSF as most promising candidates.

To estimate the minimum number of proteins necessary to quantify in order to accurately discriminate between groups, PLSDA models with various different feature counts and selection features were produced. Reduction of proteins to approximately 27 could be done without significant impact on the accuracy of the model. Furthermore, serum IL-18, MIF, MIP-5 and YKL-40 vary between systemic JIA and other JIA subtypes, while serum IL-33 levels were elevated in jSLE patients with “very high” (SLEDAI ≥ 10) when compared to patients with ”high” (SLEDAI 5-9) or “moderate” (SLEDAI ≤ 4) disease activity.

**Conclusion:** Distinct cytokine/chemokine signatures, including a minimum of 27 serum proteins, associate with paediatric autoimmune/inflammatory diseases, and discern between JIA and jSLE. Some of these proteins may correlate with disease activity in jSLE (IL-33) or associate with sub-forms of JIA (IL-18, MIF, MIP-5, YKL-40). Individual proteins or their combination may therefore be used for future diagnostic approaches, assessment of disease activity or to inform treatment decisions.

**Disclosure of Interest**

None declared

### P76 Aphtosis prevalence in autoinflammatory and multi-factorial rheumathological diseases in a population of children and adolescents

#### T. M. Caruso^1^, M. Guida^1^, S. Imparato^1^, C. Alizzi^2^, M. C. Maggio^2^

##### ^1^Università Degli Studi di Palermo; ^2^Azienda di Rilievo Nazionale ed Alta Specializzazione Ospedali "Civico Di Cristina Benfratelli", Palermo, Italy

###### **Correspondence:** T. M. Caruso

**Introduction:** Many patients have a recurrent aphtosis 2 to 4 times a year (single aphtosis); others may have recurrent lesions after the recovery of the previous (complex aphtosis). We also distinguish minor and major aphtosis: the latter are often in patients affected by diseases such as systemic lupus erythematosus (SLE), and Behçet disease. Recurrent aphtosis is in several diseases such as celiac disease, chronic inflammatory intestinal diseases, autoimmune disorders (Behçet disease), SLE, in recurrent fever as PFAPA and in monogenic autoinflammatory syndromes. A20 haploinsufficiency, due to the mutation of the TNFAIP3 gene, shows a clinical picture similar to Behçet disease’s.

The PFAPA and Behçet disease are both associated with the mutation of IL12A gene.

Therefore, recurrent aphtosis, PFAPA and Behçet disease are three clinical aspects of a single defect.

**Objectives:** The main objectives of our retrospective study are:

- to correlate the recurrent aphtosis with the most common rheumathological conditions and with defects in both innate and adaptive immunity in a paediatric population related to our Paediatric O.U.
- to detect genetic mutations related to defects of innate immunity associated with recurrent aphtosis.

**Methods:** We enlist 44 patients with recurrent aphtosis associated with fever or not, followed in our Pediatric Reumatology ward of Children Hospital “G. Di Cristina”, ARNAS Palermo, in the past two years.

The presence or absence of fever, complement alterations, serum amyloid increase, association with HLA, autoimmunity, ocular involvement, dermatological lesions and genetics for monogenic autoinflammatory diseases have been evaluated.

**Results:** Out of 44 patients enlisted, we have seen recurrent aphtosis in 28 females and 16 males, with an average age of 12 years, M:F ratio 1:1.75.

The prevalent symptom associated with aphtosis was fever (in 39/44 patients).

Skin lesions were in 15/44 patients: 1 erythema nodosum, 2 vasculitis, 3 evanescent rash, 5 urticaria/maculopapular exantema and 1 chronic urticaria.

Ocular disorders (in 6/44 patients): 2 anterior uveitis (with or without synechiae) and 4 conjunctivitis.

Laboratory abnormalities:

serum amyloid increased in 16/44 patients (very high in 5 patients and moderately high in 11). These patients had positive genetic diagnosis for FMF, cryopyrinopathy, TRAPS and A20 haploinsufficiency.

The diagnosis were: 18 FMF, 5 TRAPS, 6 CAPS, 5 SLE, 1 Behçet disease, 4 PFAPA, 1 A20 haploinsufficiency, 1 RAP, aphtosis and cyclic vomiting, 1 Behçet with isolated uveitis, 1 autoimmune thyroiditis and 1 acrocyanosis.

C3 changes were observed in three patients with SLE.

We found HLA B51 in 1 patient, HLAB57 in 1 patient and HLAB27 in 1 patient.

There were no serum amyloid increase far away from fever attacks or genetic for periodic fever syndromes in patients with PFAPA.

The genetic alterations in our patients with aphtosis were:

-among patients with FMF, 5 heterozigosis mutation P369S and R408Q, and in 1 also the A744S and 1 R202Q mutations; 2 patients had heterozigosis E148Q. Another patient had heterozigosis mutations for R202Q, E148Q.
-all patients with TRAPS had the R92Q mutation.
-among patients with CAPS, 2 had a NLRP3 (VAL200MET) mutation and 1 a NLRP12mutation .
-One patient had heterozigosis mutations for the TNFAIP3 gene with A20 haploinsufficiency.
-among patients with PFAPA 1 had TLR9 mutation.

**Conclusion:** Behçet disease in Paediatric population appears incomplete. From literature evidence bringing PFAPA to Behçet disease, it could be assumed that PFAPA could be an incomplete start of Behçet; so the genetic analysis should be extended to the IL12A gene sequencing.

It is clear from our study that the recurring aphtosis is mostly associated with monogenic diseases.

**Disclosure of Interest**

None declared

### P77 Monitoring of BK viral reactivation on JAK1/2 inhibition with baricitinib

#### K. Cetin Gedik^1^, G. Materne^1^, G. A. Montealegre Sanchez^1^, A. M. Ortega-Villa^2^, S. Alehashemi^1^, A. A. de Jesus^1^, R. Goldbach-Mansky^1^

##### ^1^Translational Autoinflammatory Diseases Section; ^2^Biostatistics Research Branch, Division of Clinical Research, National Institute of Allergy and Infectious Diseases, National Institutes of Health, Bethesda, United States

###### **Correspondence:** K. Cetin Gedik

**Introduction:** Baricitinib has been used to treat pediatric patients with rare type I interferonopathies (1). Safety profile including BK viral (BKV) reactivation in urine and pharmacokinetic model have been reported (1,2).

**Objectives:** Given the association of BK nephropathy and BK viremia (3), we determined the prevalence of BKV reactivation [BKV load in urine/blood] in baricitinib-treated patients with rare type I interferonopathies and assessed the impact of BK viremia on renal function longitudinally.

**Methods:** Between October 2011 and August 2018, 25 patients [10 CANDLE (Chronic atypical neutrophilic dermatosis with lipodystrophy and elevated temperatures), 7 SAVI (Stimulator of IFN genes–associated [STING-associated] vasculopathy with onset in infancy), 8 other interferonopathies] were enrolled in an institutional review board approved protocol and their parents gave informed consent. Safety was longitudinally assessed during study period (cut off October 2020). After detection in an index patient in June 2015, BKV load and renal function were monitored longitudinally with baseline assessments in 7 additionally enrolled patients. Summary statistics and linear mixed model were used to characterize the association of BKV load and renal function.

**Results:** The mean age at enrollment was 10.9 years [range 1.2-24.2]. On baricitinib, BKV loads (log_10_ copy/mL) in blood and urine were monitored for a mean of 3.6±2.1 years [83.9 patient years] and 3.2±1.8 years [74.9 patient years] respectively. Of 23 patients monitored, 20 had BK viruria (3/20 pre-baricitinib) and 14 BK viremia (2/14 pre-baricitinib). All except one patient with BKV load in urine >7 log_10_ copy/ml had BK viremia. Three patients with no BK viruria before treatment became positive at a mean age of 4.3 ± 1.5 years after 10.1 ± 13.2 months of baricitinib treatment. The time from achieving therapeutic doses of baricitinib to detection of BK viremia [n=12, 18.8±13.9 months] was twice as long as the time to detection of BK viruria. Of 14 patients with BK viremia, 4 had transient elevation >4 log_10_ copy/ml. Index patient developed significant BK viremia (6 log_10_ copy/ml) with azotemia and baricitinib was discontinued. One was on combination of baricitinib and a biologic agent at the time of transient elevation. Two were on baricitinib and BKV load in blood was associated with baricitinib dose and inversely associated with renal function. The baricitinib dose was adjusted to keep BK viremia <4 log_10_ copy/ml and all except index patient had stable renal function over 83.9 patient years. No difference in BKV load between gender and disease groups was observed. Dermatomal herpes zoster reactivation was seen in 2 patients and resolved on antiviral treatment without holding baricitinib.

**Conclusion:** Overall, baricitinib was well tolerated. Prevalence of BK viruria in patients on therapeutic doses of baricitinib was 87% (20/23), and 60% (14/23) for BK viremia. Our data suggest that BKV load should be monitored in blood and urine. The presence of pre-baricitinib BK viremia/viruria suggests evaluation of BKV load in patients on other chronic immunosuppressive regimens. Dose adjustments to keep BK viremia <4 log_10_ copy/ml preserved normal renal function over 83.9 patient years.

**Acknowledgements**

This baricitinib expanded access program was supported by Eli Lilly. We thank the physicians of the JAGA study network for ongoing collaboration. This research was supported by the Intramural Research Program of the NIH, Division of Intramural Research and NIAID.

**References**

1. Montealegre Sanchez GA, et al. J Clin Invest. 2018;128(7):3041-3052.

2. Kim H, et al. Clin Pharmacol Ther. 2018;104(2):364-373.

3. Hirsch H, et al. Transplantation. 2005;79(10):1277-1286

**Trial registration identifying number:** NCT01724580

**Disclosure of Interest**

K. Cetin Gedik: None declared, G. Materne: None declared, G. Montealegre Sanchez: None declared, A. Ortega-Villa: None declared, S. Alehashemi: None declared, A. de Jesus: None declared, R. Goldbach-Mansky Consultant for: Received study support under government CRADA from Lilly, IFM, Regeneron, SOBI and Novartis

### P78 Much more than the fcas phenotype; NLRP12 related periodic syndrome, data from the uper-aid registry

#### F. Demir, T. Coşkuner, K. Ulu, S. Çağlayan, B. Sözeri

##### Department of Pediatric Rheumatology, Umraniye Training and Research Hospital, Istanbul, Turkey

###### **Correspondence:** T. Coşkuner

**Introduction:** NLRP12 associated periodic syndrome (NAPS12) is a rarely seen autoinflammatory disease also known as familial cold autoinflammatory syndrome 2 (FCAS2), caused by autosomal dominant inherited mutations in the NLRP12 gene. Common clinical features of recurrent NAPS12 attacks have been described as fever, fatigue and musculoskeletal symptoms that are typically activated by cold exposure. Skin manifestations can be seen during attacks in about half of the patients.

**Objectives:** We aimed to present our single-center NAPS12 patient experience and results.

**Methods:** The data of 320 patients from next generation sequencing genetic database of autoinflammatory diseases, Umraniye Training and Resarch Hospital, Department of Pediatric Rheumatology (UPER-AID registry), were included in the study. Patients with VUS-likely pathogenic or pathogenic mutations in the NLRP12 gene were collected from these patients. In at least three months of follow-up, patients with autoinflammatory disease compatible recurrent episodes (with high acute phase response) were defined as NAPS12, in which other etiological reasons (infectious, autoimmune, malignant diseases) were excluded. Demographic, clinical and laboratory data, treatments and responses of patients with a diagnosis of NAPS12 were presented.

**Results:** Eight patients were diagnosed with NAPS12. The F/M ratio was found as 5/3. The mean age of onset of the symptoms was 56.6 months (min-max: 7-120), and the mean age at diagnosis was 109 months (min-max: 10-122). The mean follow-up time was found 17.5 months (min-max: 3-42). The duration of the attack varied between 1-14 days. The attack findings frequency was found as follow respectively; fever (n: 7), urticarial rash (n: 6), myalgia (n: 4), arthralgia (n: 3), abdominal pain (n: 2), conjunctivitis (n: 2), diarrhea (n: 1) ranked as headache (n: 1) and pericarditis (n: 1). CRP and serum amyloid A levels were found to be elevated in all patients during the attack period. In the NGS gene panel, three patients had pathogenic, one had likely pathogenic, three had VUS, and one had novel variants. Partial response was obtained with colchicine in 3 patients and complete response in 2 patients. The other three patients that unresponsive to colchicine, were achieved to inactive disease with anti-IL 1 treatments.

**Conclusion:** While the clinical findings were compatible with FCAS in four patients, attacks accompained with varied clinical findings and lasting longer than 3 days were observed in the other four. It was observed that the attack duration of patients who were not compatible with the FCAS, could extend up to 14 days. NAPS12 should be considered in the differential diagnosis in patients with a clinical manifestations of autoinflammatory diseases who do not show typical findings for any monogenic SAID.

**Trial registration identifying number:** (Approval No/Date: B.10.1.TKH.4.34.H.GP.0.01/233/18.12.2019).

**Patient Consent Received**

Yes

**Disclosure of Interest**

None declared

### P79 Cluster analysis of pediatric behçet’s disease: data from the Pediatric Rheumatology Academy (PERA)-research group

#### F. Demir^1^, H. E. Sönmez^2^, E. Bağlan^3^, Ö. Akgün^4^, T. Coşkuner^5^, G. Otar Yener^6^, K. Öztürk^7^, M. Çakan^8^, S. G. Karadağ^9^, S. Özdel^3^, N. Aktay Ayaz^4^, B. Sözeri^1^ on behalf of The Pediatric Rheumatology Academy (PeRA)-Research Group

##### ^1^Department of Pediatric Rheumatology, University of Health Sciences, Ümraniye Research and Training Hospital, Istanbul; ^2^Pediatric Rheumatology, Kocaeli University, Faculty of Medicine, Kocaeli; ^3^Department of Pediatric Rheumatology, University of Health Sciences, Sami Ulus Maternity and Children's Diseases Training and Research Hospital, Ankara; ^4^Department of Pediatric Rheumatology, Istanbul University, Faculty of Medicine; ^5^Pediatric Rheumatology, Umraniye Training and research Hospital, Istanbul; ^6^Department of Pediatric Rheumatology, Şanlıurfa Training and Research Hospital, Sanliurfa; ^7^Department of Pediatric Rheumatology, Istanbul Medeniyet University, Göztepe Prof. Dr. Süleyman Yalçın City Hospital; ^8^Department of Pediatric Rheumatology, University of Health Sciences, Zeynep Kamil Maternity and Children's Diseases Training and Research Hospital, Istanbul; ^9^Department of Pediatric Rheumatology, Erzurum Regional Research and Training Hospital, Erzurum, Turkey

###### **Correspondence:** F. Demir

**Introduction:** Behçet‘s Disease (BD) is a systemic vasculitis affecting many organ systems with the involvement of all sized arteries and veins. Although recurrent oral aphthous and cutaneous lesions are the predominant features during the course of the disease, five different forms of disease have been defined previously: 1) mucocutaneous-only cluster, 2) papulopustular lesion–arthritis–enthesopathy cluster, 3) ocular cluster, 4) gastrointestinal cluster, 5) vascular cluster.

**Objectives:** To determine the main characteristics of pediatric BD patients and also analyze the clustering phenotype of pediatric BD in a large multicentric cohort.

**Methods:** Eight pediatric rheumatology centers from Turkey have participated in this study. Demographic data, clinical manifestations, laboratory features, radiological findings, treatment schedules, and disease outcomes were achieved from patient charts retrospectively. A cluster analysis was performed according to five independent clinical forms.

**Results:** A total of 241 patients with BD were enrolled in the study. Among them, 120 (49.7%) were male and 121 (50.3%) were female. Eighty-three (34.4%) patients had a family history of BD. The median age of diagnosis was 144 (36-216) months. The median time between onset of symptoms and diagnosis was 1 (0-196) months. Oral aphthous (83.8%) was both the most common initial symptom and the most frequent symptom (97.9%) during the disease course followed by genital ulcers (66.8%) and pseudofollicutis (33.2%). Uveitis was observed in 34 (14.1%) patients. Thirty-three (13.7%) patients had neurological involvement. Pathergy test was positive in 64 (26.6%) patients and HLA-B51 was positive in 108 (44.8%) patients. According to cluster analysis; 133 (55.1%) patients belonged to the mucocutaneous-only cluster while 37 (15.4%) patients fitted to papulopustular lesion–arthritis–enthesopathy cluster, 33 (13.7%) were in ocular cluster, 19 (7.9%) were in gastrointestinal cluster and 19 (7.9%) belonged to the vascular cluster. Ocular and vascular clusters were more common in boys (p<0.001) while girls usually presented with mucocutaneous-only cluster. The age of diagnosis were similar among the clusters. The activity scores of disease at the diagnosis and at the last control were higher in ocular and gastrointestinal clusters (p=0.001).

**Conclusion:** To our best of knowledge, this is the first cluster analysis in pediatric BD patients. Our analysis showed that mucocutaneous-only cluster was the common form while phenotype of the disease differed according to gender. Furthermore, ocular and gastrointestinal involvements affected disease activity not only at the diagnosis but also during the course of the disease.

**Trial registration identifying number:** .

**Patient Consent Received**

Yes

**Disclosure of Interest**

None declared

### P80 Evaluation of childhood behçet's disease cases: a single center experience

#### S. Doğantan, S. N. Taşkın, A. P. Kısaarslan, H. M. Poyrazoğlu

##### Division of Pediatric Rheumatology, Kayseri, Erciyes University Faculty of Medicine, Kayseri, Turkey

###### **Correspondence:** S. N. Taşkın

**Introduction:** Behçet's disease is a multisystem vasculitis that can affect all sizes of arteries and veins. Diagnosis can be made mostly in adult ages. Evaluation of childhood findings is important for early diagnosis. Serious complications can be seen years after diagnosis in the disease, which has periods of exacerbation and recovery. Although the mortality of Behçet's disease is low, intestinal perforation and central nervous system involvement are risky in terms of morbidity and mortality. Long-term effects are related to organ involvement. Young age, male gender, early onset disease are poor prognostic factors (1,2).

**Objectives:** We aimed to evaluate the demographic, clinical and laboratory data of patients who were followed up with a diagnosis of Behçet's disease in our clinic.

**Methods:** In our study, data were collected by retrospectively examining the files of patients who were followed up with the diagnosis of Behçet's disease between 01.01.2010 and 01.01.2021 in Erciyes University Children's Hospital Pediatric Rheumatology Department. While making the diagnosis, the diagnostic criteria of the International Behçet's Disease Working Group and the Consensus Classification of pediatric Behçet's Disease-2016 were used. Patients with 2 other criteria (genital ulcers, skin lesions, uveitis, pathergy) in addition to recurrent oral ulcers according to the diagnostic criteria of the International Behçet's Disease Study Group were included in the study (3). According to the Consensus Classification of Pediatric Behçet's Disease-2016 diagnostic criteria, three of six items are required (recurrent oral aphthae, genital ulcer, skin involvement, eye involvement, neurological involvement, vascular involvement) (4)

**Results:** We had a total of 42 cases in the study group. 22 (52.4%) of the patients were female and 20 (47.6%) were male. The average age at the time of first application was 12.56 ± 3.34. There was first degree consanguinity among the parents of 9.5% (n = 4) of the patients. 35.7% of the patients (n = 15) had a family history of BD. Recurrent oral aphthae were present in 85.7% (n = 36) of the patients.

Genital aphthae in 28.6% (n = 12) of patients, uveitis in 26.1% (n = 11), skin lesions in 21.4% (n = 9), 14.2% (n = 6) vascular involvement, 4.8% (n = 2) central nervous system vascular involvement, 7.14% (n = 3) had GIS findings, and one patient (2.4%) had epididymoorchitis. 45.4% (n = 5) of uveitis cases had anterior uveitis, 36.3% (n = 4) posterior uveitis, 9% (n = 4) middle uveitis and 9% (n = 1) ) panuveitti. Of the cases with skin lesions, 11.1% (n = 1) erythema nodosum, 22.2% (n = 2) pseudofolliculitis, 33.3% (n = 3) acneiform lesions and 33.3% ( n = 3) livedo reticularis was present. Pathergy test was positive in 11.9% of the patients (n = 5). One patient had GIS bleeding (rectal bleeding), one patient had chronic active inflammation in the colon, and one patient had an ulcer in the terminal ileum. HLA B51 was found to be positive in 57.14% (n = 24) of the patients. Systemic steroid in 28.6% (n = 12) of patients, colchicine in 95.2% (n = 40), azathioprine in 19% (n = 8), and methotrexate in 33.3% (n = 14), 11.9% (n = 5) sulfasalacin, 4.8% (n = 2) infliximab, 4.8% (n = 2) adalimumab were given. 26.1% (n = 11) of the patients had additional disease. Two patients had familial Mediterranean fever, 1 patient had phenylketonuria, 2 patients had atrial septal defect, 1 patient had epilepsy, 1 patient had essential tremor, 1 patient had ulcerative colitis, 1 patient had Sydenham's Chorea.

**Conclusion:** The clinical features of childhood Behçet's patients vary widely. The clinical findings becoming evident over the years indicates the importance of follow-up.

**Patient Consent Received**

Yes

**Disclosure of Interest**

None declared

### P81 Early onset of mevalonate kinase deficiency syndrome: 2 cases in the one center

#### M. Dubko, A. Zinchenko, E. Goltsman, I. Solodkova

##### Saint Petersburg Pediatric Medical University, Saint-Petersburg, Russian Federation

###### **Correspondence:** M. Dubko

**Introduction:** Mevalonate kinase deficiency (MKD) is an extremely rare autoinflammatory disorder with an autosomal recessive inheritance mechanism.

According to the EUROFEVER register, the age of onset of MKD is on average 6 months, and the delay in establishing the diagnosis is 6.8 years. Patients are often treated as cases with infectious diseases, primary immunodeficiency conditions, or other autoinflammatory disorders

In the clinical picture, the most important is the periodicity of fevers and associated rashes, cervical lymphadenopathy, hepatosplenomegaly and increased markers of inflammation (CRP, ESR). Early manifestation is usually associated with a severe course and poor prognosis

**Objectives:** Show the efficiency of therapy with the IL-1 blocker canakinumab at the example of two cases of early onset of mevalonate kinase deficiency syndrome.

**Methods:** Observation and description of clinical picture.

Therapy with the IL-1 blocker canakinumab.

**Results:** In the center, we observed 2 patients with genetically confirmed mevalonate kinase deficiency syndrome.

Both babies (girl and boy) were born prematurely: 36 and 35 weeks. The girl matched the gestational age in terms of weight and height parameters, and the boy had signs of intrauterine growth retardation (P 10). The condition of the children at birth was assessed as severe. The leading symptoms were: thrombocytopenia (31*10)^9 and (98*10)^9, anemia (hemoglobin 68 g / l and 83 g / l), significant hepatosplenomegaly, stable over the entire observation period. Both babies were treated as patients with intrauterine infection and received repeated courses of combined antibiotic therapy, IVIG, transfusion of thrombus suspension and erythrocyte mass. Both babies had no fevers until age of 4 months. Equivalent to fevers there were periodic increases in CRP and ESR. A typical clinical picture appeared only by the age of 4 months. Genetic analysis revealed in the girl the pathogenic variant c.118C> T (p.R40W) and c.206_207del (p.S69fs) in the MVK gene in a compound heterozygous state. The boy has c.939_940del (p.L315Gfs * 49) and c.1006G> A (p.G336S). Both children are successfully receiving canakinumab therapy.

**Table:** 

Attribute	Patient 1	Patient 2
Weight at birth	2915	1910
Body length at birth	48	43
Platelets	(31*10)^9	(98*10)^9
Hb	68 g / l	83 g / l
Organomegaly	Hepato-splenomegaly	Hepato-splenomegaly since 2 month
Fever start	3 month	4 month
Course of disease	Recurrent at short intervals	Continuously relapsing
Canakinumab dose	6 mg/kg 1 per month	4 mg/kg 1 per month
Result	No exacerbations	No exacerbations

**Conclusion:** We described 2 cases of mevalonate kinase deficiency syndrome with onset in the neonatal period and successful therapy with the IL-1 blocker canakinumab.

**Patient Consent Received**

Yes

**Disclosure of Interest**

None declared

### P82 An interesting relationship between inattention/hyperactivity and familial mediterranean fever in children and adolescents

#### G. Durcan^1^, K. Barut^2^, F. Haslak^2^, M. Yildiz^2^, H. Doktur^1^, M. T. Kadak^3^, Z. Koyuncu^3^, A. Adrovic^2^, S. Sahin^2^, B. Dogangun^3^, O. Kasapcopur^2^

##### ^1^Department of Child and Adolescent Psychiatry, University of Health Sciences, Bakirkoy Research and Training Hospital for Psychiatry, Neurology and Neurosurgery; ^2^Department of Pediatric Rheumatology; ^3^Department of Child and Adolescent Psychiatry, Medical Faculty of Cerrahpasa, Istanbul University-Cerrahpasa, Istanbul, Turkey

###### **Correspondence:** M. Yildiz

**Introduction:** Although familial Mediterranean fever (FMF) progresses with attacks, its subclinical inflammation may continue in attack-free periods. To date, increased inflammatory cytokines have been reported in many psychiatric diseases.

**Objectives:** In this study, we aimed to evaluate the psychological symptoms, especially inattention/hyperactivity, in children and adolescents with FMF.

**Methods:** The study included 272 children and adolescents with FMF and 250 healthy peers as a control group. The Strengths and Difficulties Questionnaire-Parent Form was used to assess emotion, behavior and peer related problems, as well as inattention/hyperactivity and prosocial behavior in participants.

**Results:** The mean age of the patients was 12.35, ± 2.65 years, and the mean age of the control group was 12.08, ± 2.67. In total, 51% (n = 139) of patients with FMF and 56% (n = 140) of the control group were females. The age and gender of the children were similar across groups (p=0.265 for age; p=0.262 for gender). The emotional and behavioral problem subscale scores of patients with FMF were significantly higher than those of healthy controls. The inattention/hyperactivity scores of patients with FMF were also significantly higher than those of the control group (3.99 ± 2.34 vs 2.93± 2.26, p <0.001). The psychological scale comparisons of the patient and control groups are given in Table 1 in detail. When patients with FMF were compared according to the presence of attacks in the last year, presence of exertional leg pain as well as their mutation types, no differences were found in terms of inattention/hyperactivity scores. However, patients whose FMF symptoms were onset before the age of 6 had significantly higher inattention/hyperactivity subscale scores (4.21 ± 2.42) when compared to patients whose symptoms started after 6 years of age (3.42 ± 2.02; p =0.016).

**Conclusion:** This research demonstrated that FMF patients had increased inattention/hyperactivity, similar across all ages and genders, which was unaffected by FMF-related variables, except for age of onset. The FMF-inattention/hyperactivity relationship may be due to a common etiology in which proinflammatory cytokines play a role.

**Patient Consent Received**

Yes

**Disclosure of Interest**

None declared

**Table 1 (abstract P82).** Comparison of psychological scale scores in patient and control groups

	Patients (n=272)	Controls (n=250)	p
	Mean ± SD		
SDQ- total score	11.82 ± 5.71	8.97 ± 5.54	<0.001
SDQ- emotional problems	3.22 ± 2.42	1.97 ± 2.09	<0.001
SDQ- conduct problems	1.93 ± 1.69	1.34 ± 1.57	<0.001
SDQ- hyperactivity/inattention	3.99 ± 2.34	2.93 ± 2.26	<0.001
SDQ- peer problems	2.67 ± 1.57	2.73 ± 1.87	0.914
SDQ- prosocial behavior	8.10 ± 1.93	7.98 ± 2.21	0.858

*SDQ* Strenghts and Difficulties Questionarie

### P83 Refractory panuveitis with splenomegaly in a 14-year-old adolescent

#### P. Dusser^1^, C. BOROCCO^1^, C. Titah^2^, G. Sarrabay^3^, I. Koné-Paut^1^

##### ^1^Rheumatology-Pediatrics Department and CeRéMAIA (Reference Centre for Autoinflammatory Diseases and Inflammatory Amyloidosis), APHP - Bicêtre Hospital, Le Kremlin Bicêtre; ^2^Ophthalmology Department, Rothschild Ophthalmological Foundation, Paris; ^3^Laboratory of rare genetic and autoinflammatory diseases and CeRéMAIA (Reference Centre for Autoinflammatory Diseases and Inflammatory Amyloidoses), University of Montpellier, Montpellier, France

###### **Correspondence:** P. Dusser

**Introduction:** ROSAH syndrome is characterised by the association of retinal dystrophy, optic nerve oedema, splenomegaly, anhidrosis and migraines. It has recently been shown that this syndrome is monogenic, caused by dominant mutations in the *ALPK1* gene. Up to date, 18 patients of ROSAH syndrome have been reported.

**Objectives:** Clinical and biological description of an atypical panuveitis.

**Methods:** Herein we present the case of a 14-year-old male who presented to us with an atypical bilateral posterior uveitis associated with splenomegaly and migraines.

**Results:** N. is a Caucasian teenager, monophthalmic, with bilateral posterior uveitis complicated by right papilledema and left anterior retinal detachment, cortico-dependent, evolving since the age of 5 years old. At the age of 8 years old, he presented with a febrile episode with splenomegaly and pancytopenia, in relation to a PVB19 infection, which resolved spontaneously. On the paternal side, there was evidence of splenectomy in the father and grandfather during an EBV infection. The grandfather also had uveitis and died at 59 years old of kidney cancer in the context of familial polycystic hepatorenal disease. Biologically, no systemic inflammation was found except for a significant synthesis of IL1β in our adolescent. Several treatment lines have been tried (anti-IL1, anti-TNF and anti-IL6) for N. without efficacy. In this context, a trio exome was performed showing a mutation in the ɑkinase gene, *ALPK1* (NM_025144.4 :c.710C>T, [p.Thr237Met]) in favour of ROSAH syndrome for N. and his father.

**Conclusion:** ROSAH syndrome is therefore a differential diagnosis to be considered in cases of posterior uveitis with collapsed visual acuity resistant to treatment and should be investigated for a family history of splenomegaly and/or uveitis.

**Patient Consent Received**

Yes

**Disclosure of Interest**

None declared

### P84 Clinical features and colchicine response in patients with undifferentiated systemic autoinflammatory disease carrying E148Q VS. other MEFV mutations

#### B. Egeli^1^, H. Wobma^2^, M. Marques^3^, J. Hausmann^4^, F. Dedeoglu^1^

##### ^1^Immunology; ^2^IImmunology, Boston Children's Hospital, Boston; ^3^UPMC Children's Hospital, Pittsburgh; ^4^Boston Children's Hospital, Boston, United States

###### **Correspondence:** B. Egeli

**Introduction:** Undifferentiated systemic autoinflammatory diseases (uSAID) are diverse syndromes characterized by acute flares of fever and inflammation, which do not meet clinical criteria for known disorders like Familial Mediterranean Fever (FMF). As part of the uSAID workup, many patients undergo genetic testing, sometimes revealing variants of uncertain significance in genes associated with autoinflammation. E148Q is a common polymorphism in exon 2 of the *MEFV* gene, which is not thought to be a disease-causing variant for FMF. The contribution of E148Q mutations in patients with uSAID is poorly understood, and it is unknown how it may respond to empiric treatment with colchicine, which is first line for FMF.

**Objectives:** To compare the clinical characteristics and colchicine response of children with uSAID identified to have E148Q vs non-E148Q mutations in the *MEFV* gene.

**Methods:** Children with uSAID ≤18 years old at initial evaluation seen at a single-center during 2000-2019 were included if they received ≥3 months of colchicine therapy and carried at least one *MEFV* mutation but did not meet clinical criteria for FMF (n = 25). Data on demographics, clinical features, laboratory/genetic studies, and treatment responses were collected.

**Results:** Results: In our cohort of 25 children with uSAID and *MEFV* mutations, 8 (32%) were heterozygous for E148Q mutations. Half of these patients also carried another non- exon 10 *MEFV* mutation (2x 369S, 1x L110P, 1x I591T). Distribution of the remaining variants on *MEFV* gene is shown in Figure 1. Clinical features of children with E148Q vs. other *MEFV* mutations are shown in Table 1. Asian ancestry was seen in 3/8 (37%) children with E148Q mutations and in no child with other *MEFV* mutations. Children with E148Q mutations had longer length of febrile episodes (8.36.5 vs. 3.4 days; p=0.009) and were less likely to have a full response to colchicine (25% vs 70%; p = 0.03).

**Conclusion:** In our cohort of children with uSAID and *MEFV* mutations, E148Q was associated with longer duration of fever flares and a reduced colchicine response. Larger studies will be helpful in elucidating the unique role of these mutations in autoinflammation.

**Patient Consent Received:** No

**Disclosure of Interest**

B. Egeli: None declared, H. Wobma: None declared, M. Marques: None declared, J. Hausmann Consultant for: Novartis, Pfizer, Biogen, Rheumatology Research Foundation, CARRA, F. Dedeoglu Consultant for: Novartis

**Table 1 (abstract P84).** Characteristics of uSAID patients with heterozygous *MEFV* mutations

	E148Q group (n=8)	Non-E148Q group (n=17)	p value
Complete response, (%)	2 (25)	12 (70.6)	0.03
Mean episode duration (days), (SD)	8.3 (6.5)	3.7 (1.4)	0.009
Asian ancestry, (%)	3 (37.5)	0 (0)	0.04
GI symptom, (%)	5 (62.5)	8 (47.1)	0.47
MSK symptom, (%)	3 (37.5)	9 (52.9)	0.47
Pharyngitis, (%)	3 (37.5)	5 (29.4)	0.69
Rash, (%)	3 (37.5)	11 (64.7)	0.20

Figure 1 - Location of non-E148Q *MEFV* mutations in uSAID patients (n=17)

### P85 Efficacy, safety and tolerance of anakinra in paediatric rheumatology and periodic fever clinics: real life experience

#### S. Fingerhutova^1^, E. Jancova^2^, P. Dolezalova^1^

##### ^1^Centre for Paediatric Rheumatology and Autoinflammatory Diseases, Department of Paediatrics and Inherited Metabolic Disorders; ^2^Department of Nephrology, First Faculty of Medicine, Charles University and General University Hospital in Prague, Prague, Czech Republic

###### **Correspondence:** S. Fingerhutova

**Introduction:** Interleukin 1 (IL-1) induced proinflammatory signals were discovered as a causative aetiology in a spectrum of diseases. Efficacy and safety of the recombinant IL-1 receptor antagonist anakinra across autoinflammatory and autoimmune diseases has been demonstrated in many studies. Despite the recommended dosage in patients above 8 months and weighing more than 10 kg, use of higher doses or earlier onset of therapy have been occasionally reported.

**Objectives:** To an institutional review of data on efficacy, safety and tolerance of anakinra in patients with autoinflammatory diseases (AID).

**Methods:** A single-centre retrospective review of electronic records of patients treated with anakinra between August 2007 and May 2021.

**Results:** A total of 47 patients (30 children, 17 adults) were identified. The median follow-up was 35 months (range 1-165 months). Patients have been treated for diagnosis of systemic juvenile idiopathic arthritis (sJIA) (n = 18; 38%), cryopyrinopathy (CAPS) (n = 10; 21%), mevalonate-kinase deficiency (MKD) (n = 7; 15%), undifferentiated AID (uAID) (n = 6; 13%), PIMS-TS (n = 3; 6%), NLRC4-GOF (n = 1, 2%), PAPA syndrome (n = 1; 2%) and polyarticular JIA (n = 1, 2%). The most frequent indication for starting anakinra was macrophage activation syndrome (MAS) (n = 20; 42,5%) which occurred in patients with sJIA (n=14, 70%), uAID (n=3, 15%), PIMS (n=1, 5%), NLRC4-GOF (n=1, 5%) or polyarticular JIA (n=1, 5%). Fourteen patients with sJIA (78%) received anakinra due to macrophage activation syndrome. MAS was the first manifestation of sJIA in 6 patients (33%).

Recommended dosing of anakinra (1-4 mg/kg/day) was exceeded in 44,6% of patients (n=21) with the following dose range: 4-6 mg/kg (n = 8; 38%), 6-9,9mg/kg (n = 4; 19%), ≥10 mg/kg (n = 9; 43%). Paediatric cohort received anakinra in very wide dosing range of 1,4 -26,1 mg/kg (average 5,59 mg/kg, median 4,15 mg/kg). The highest dose (10-26mg/kg) was required by patients with uAID (n=1, 8 days of age), sJIA/MAS (n=2, 3 and 5 years of age), CINCA (n=1, 4 years of age) and NLRC4-GOF (n=1, 4 weeks of age). The median dose of anakinra in adult patients was 1,6 mg/kg (range 0,9-7,7mg/kg). In severely sick patients the daily dose was divided into 2-4 intravenous applications, one patient received continuous anakinra i.v. infusion. Rapid therapeutic effect (within 24-48 hours from starting anakinra) was observed in all patients.

The most frequent recorded adverse effects were already well-known injection-site reaction in 25,5% (n = 12) of patients which disappeared within one month in all of them. Persistent eosinophilia (highest values 3,6 and 2,3x10*9 cells) was documented in 2 sJIA patients. Mild asymptomatic neutropenia (ANC min 0,8 x10*9/L) and transient liver transaminase elevation (up to 3-times ULN) both occurred in 4,2% (n = 2) of patients each.

**Conclusion:** Use of anakinra in a wide dosing range was reported. Our observation illustrates the need as well as safety of higher anakinra dosing in younger age groups including 2 newborns. No serious adverse effects that would require discontinuation or termination of anakinra were observed at all dosing regimens.

ANC = absolute neutrophil count

This work was supported by the Ministry of Health of the Czech Republic (NU21-05-00522)

**Disclosure of Interest**

None declared

### P86 Myositis as a deciding but late symptom of DADA2 syndrome – report of two cases

#### K. Palm-Beden^1^, F. Gohar^1^, D. Windschall^1,2^

##### ^1^Clinic for Paediatric and Adolescent Rheumatology, St. Josef-Stift Hospital, Northwest German Center for Rheumatology, Sendenhorst; ^2^University Halle-Wittenberg, Halle, Germany

###### **Correspondence:** F. Gohar

**Introduction:** The recognition of adenosine deaminase-2-(ADA2)-deficiency (DADA2), a monogenic vasculitis syndrome, is important due to the significant morbidity associated with the increased risk of stroke and vasculopathy.

**Objectives:** DADA2 has a highly variable clinical presentation including signs of vasculopathy (e.g. livedo reticularis, vasculitis, stroke), systemic inflammation and musculoskeletal findings e.g. arthritis and myositis, which is not widely recognised to be a typical finding associated with the diagnosis.

**Methods:** We report the diagnostic work-up and management of two patients with DADA2, where myositis was a key symptom to reaching the diagnosis.

**Results:** Patient 1 and 2 (both female, Turkish-German ethnicity), presented aged 5 and 10 years respectively, with muscle and joint pain, a history of fever attacks and livedo reticularis beginning in infancy. Inflammatory markers including serum amyloid A, were persistently elevated in both patients. Diagnostic tests ruled out HIDS, Blau Syndrom (Patient 1), as well as Bechet’s Disease (negative HLA-B51 and Pathergie test), CAPS, TRAPS and FMF. However, Patient 2 was positive for the non-pathogenic FMF heterozygous mutation A744S. Initial therapy for both patients was colchicine with varying compliance and effectiveness. In patient 1, therapy was extended to include azathioprine and due to minimal effect, oral cortisone. Unfortunately, both patients attended sporadically and were non-compliant with therapy. In this time, Patient 1 was found to be a distant relative of Patient 2. Ten years after initial presentation, Patient 1 complained of muscle pains in the thighs. CK was normal, PM-Scl antibodies were positive and whole-body MRI showed muscle oedema and inflammation particularly affecting the vastus medialis and gracilis muscles. In combination with a history of trochlear nerve paralysis, the diagnosis of DADA2 was suspected. Once confirmed with genetic testing, treatment was initiated with the TNF-Inhibitor adalimumab. Fever and inflammation episodes did not reoccur, and the muscle pain resolved. Patient 2 was invited for re-evaluation after being lost to follow-up. However, before attending, she suffered a lacunar thalamus infarct. Genetic testing and etanercept were initiated after acute neurological management. The patient had first developed muscle pains in the upper and lower legs 2-3 weeks after the infarct, without elevated CK. Whole-body MRI confirmed myositis of the symptomatic muscles.

**Conclusion:** In patients with recurrent autoinflammation and myositis and an otherwise unclear diagnosis, DADA2 should be considered as a possible cause. Whole-body MRI can help confirm myositis. A thorough family history and genetic testing are vital to reach the diagnosis.

**Patient Consent Received**

Yes

**Disclosure of Interest**

None declared

### P87 Influence of canakinumab dosing on efficacy and safety of long-term treatment in patients with familial mediterranean fever **−** interim analysis of the reliance registry

#### T. Kallinich^1^, J. Henes^2^, N. Blank^3^, F. Dressler^4^, I. Foeldvari^5^, M. Hufnagel^6^, G. Horneff^7^, B. Kortus-Goetze^8^, F. Weller-Heinemann^9^, F. Meier^10^, J. Weber-Arden^11^, J. B. Kuemmerle-Deschner^2^

##### ^1^Charite University Medicine, Berlin; ^2^University Hospital, Tuebingen; ^3^University Hospital, Heidelberg; ^4^Hannover Medical School, Hannover; ^5^Centre for Pediatric and Adolescence Rheumatology, Hamburg; ^6^University Hospital, Freiburg; ^7^Asklepios Clinic, Sankt Augustin; ^8^University Hospital, Marburg; ^9^Prof. Hess Kinderklinik, Bremen; ^10^University Hospital, Frankfurt; ^11^Novartis, Nuernberg, Germany

###### **Correspondence:** T. Kallinich

**Introduction:** Familial Mediterranean Fever (FMF) is characterized by severe systemic and organ inflammation. Successful treatment with rapid remission of symptoms and normalization of laboratory parameters was achieved in most patients with the interleukin-1β inhibitor canakinumab (CAN) in clinical trials.

**Objectives:** The aim of the present analysis was the evaluation of long-term efficacy and safety of CAN in pediatric (age ≥2 years) and adult patients with FMF with respect to weight-dependent CAN dosing in routine clinical practice.

**Methods:** RELIANCE is a prospective, non-interventional, observational study based in Germany. Patients with clinically confirmed diagnoses of autoinflammatory periodic fever syndromes routinely receiving CAN are enrolled. Efficacy- and safety-parameters, CAN dosing as well as weight were recorded at baseline and assessed at 6-monthly intervals within the 3-year observation period of the study.

**Results:** The interim analysis of the RELIANCE Registry comprises data of 54 FMF patients enrolled by December 2020. Of these, the % of patients reported to receive standard dose CAN (SD CAN; 150 mg or 2mg/kg respectively per 4 weeks) halved from 77% at baseline to 36% at month 18 in favor of less than SD CAN (<87.5% of SD) and higher than SD CAN (>112.5% of SD).

Patients´ and physicians´ rating of disease activity was higher in patients receiving SD CAN and higher (table 1), even though CRP was equally well controlled in all three dosing groups. A total of 11 serious adverse events was reported, of which 1 case of tonsillectomy was classified as drug-related.

The interim analysis of the RELIANCE Registry comprises data of 54 FMF patients enrolled by December 2020. Of these, the number of patients reported to receive less than standard CAN dose* (SD CAN; <87.5% of SD) / SD CAN / higher than SD CAN (>112.5% of SD) were 2/36/9 at baseline, 10/13/9 at 6 months, 11/7/8 at 12 months and 5/5/4 at 18 months.

Even though CRP was equally well controlled in all three dosing groups, disease activity was rated higher by patients and physicians in patients receiving SD CAN and higher (table 1). A total of 11 serious adverse events was reported, of which 1 case of tonsillectomy was classified as drug-related.

**Conclusion:** The present interim data from the RELIANCE study confirm efficacy and safety of long-term CAN treatment in clinical routine. CRP levels were well controlled in all dosing groups. Remaining disease activity was mainly observed in patients under SD CAN and higher than SD CAN.

**Disclosure of Interest**

T. Kallinich: None declared, J. Henes Consultant for: Novartis, AbbVie, Sobi, Roche, Janssen, Boehringer-Ingelheim;, N. Blank Consultant for: Novartis, Sobi, Lilly, Pfizer, Abbvie, BMS, MSD, Actelion, UCB, Boehringer-Ingelheim, Roche, F. Dressler Consultant for: Abbvie, Mylan, Novartis, Pfizer, I. Foeldvari Consultant for: Novartis, M. Hufnagel: None declared, G. Horneff Speaker Bureau of: AbbVie, Bayer, Chugai, Merck Sharp & Dohme, Novartis, Pfizer, Roche, B. Kortus-Goetze Consultant for: Novartis, F. Weller-Heinemann: None declared, F. Meier: None declared, J. Weber-Arden Employee of: Novartis, J. B. Kuemmerle-Deschner Consultant for: Novartis, AbbVie, Sobi

**Table 1 (abstract P87).** Stratification of efficacy parameters by CAN dose category and weight (N=54)^#^

		Baseline	6 months	12 months	18 months
% of patients on low/standard/high dose CAN, *number*^*#*^ *of patients analysed (N)*		4/77/19, *N=47*	31/41/28, *N=32*	42/27/31, *N=26*	36/36/28, *N=14*
		**PGA absent**/**mild-moderate**/**severe**			
Physician Global Assessment (PGA), %^#^ of patients	lower than SD* CAN	**54/23/15**	**80/10/0**	**78/22/0**	**80/20/0**
	SD* CAN	**42/50/0**	**88/12/0**	**50/33/0**	**50/25/0**
	higher than SD* CAN	**20/60/20**	**33/67/0**	**0/67/0**	**0/100/0**
		**VAS 0–10**, (min; max)			
Patients' assessment of current disease activity (median)	lower than SD* CAN	**1** (0; 9)	**1** (0; 7)	**1** (0; 4)	**2** (0; 3)
	SD* CAN	**2** (0; 10)	**3** (0; 7)	**3** (0; 10)	**4** (0; 10)
	higher than SD* CAN	**4** (1; 5)	**3** (0; 6)	**3** (1; 5)	**4** (1; 6)

*Body weight >40kg: SD is 150 mg per 4 weeks; Body weight ≤40 kg: SD is 2mg/kg per 4 weeks ^#^Numbers/percentage do not sum up to N=54/100%, due to unknown weight in a number of patients

### P88 Two siblings with Majeed syndrome and neutropenia

#### M. Kasap Cuceoglu^1^, E. D. Batu^1^, A. E. Yildiz^2^, U. Kaya Akca^1^, E. Atalay^1^, S. Sener^1^, Z. Balik^1^, O. Basaran^1^, Y. Bilginer^1^, S. Ozen^1^

##### ^1^Pediatric Rheumatology; ^2^Department of Radiology, Hacettepe University, Ankara, Turkey

###### **Correspondence:** M. Kasap Cuceoglu

**Introduction:** Majeed syndrome (MS) is a rare monogenic autoinflammatory disease characterized with early-onset chronic nonbacterial osteitis and hematological features; especially dyserythropoietic microcytic anemia.

**Objectives:** Here, we report the first sibling cases of Majeed syndrome from Turkey.

**Methods:** Case Report

**Results: Patient 1**

A seven-year-old boy presented to the local pediatrician with recurrent joint and bone pain. Bone pain started at the age of 18 months. There was no joint swelling, recurrent abdominal, chest pain or fever in his past medical history, and there were no signs of uveitis. His parents were first degree cousins. Physical examination was normal. Acute phase reactants (APRs) were high at admission (erythrocyte sedimentation rate (ESR) 50 mm, normal range 0-20, and C-reactive protein (CRP) 3 mg/dl, normal range 0-0.5). Neutropenia (1100/mm^3^) was detected with normal white blood cell (WBC) count. Antinuclear antibody (ANA) and extractable nuclear antigen antibodies (ENA) were negative. The local pediatrician initially started him on colchicine treatment, suspecting familial Mediterranean fever. However, *MEFV* gene variant analysis did not reveal any mutations. After two years of colchicine treatment, his family discontinued the drug since there was no response. The patient was then referred to our center. A periodic fever gene panel analysis (including *LPIN2, MEFV, MVK, NLRP3, PSTPIP1* and *TNFRSF1A* genes) was performed with next generation sequencing and homozygous mutation was detected in exon 4 of the *LPIN2* gene; c.589C>T (p. Arg197*). Both his parents were carriers for this variant. The whole body musculoskeletal system magnetic resonance imaging (MRI) was normal. After the diagnosis of Majeed syndrome, recombinant IL-1RA (anakinra) treatment was initiated at a dose of 2 mg/kg/day via the subcutaneous route. The patient has remained free of symptoms on anakinra treatment. However, neutropenia did not improve as of yet.

**Patient 2**

The sister of patient 1, presented to our department with recurrent joint and bone pain, fatigue, and anemia in the last six months. Physical examination was unremarkable. APRs were elevated at admission (ESR 120 mm and CRP 14 mg/dl). Severe neutropenia (600/mm^3^) with normal WBC count, microcytic anemia (Hb 8.6 mg/dl), and thrombocytosis (700x10^3^/mm^3^) were detected. She was consulted to the pediatric hematology department. Her bone marrow assessment revealed a normocellular bone marrow with megaloblastic changes. The whole body musculoskeletal system MRI demonstrated osteitis at the distal femur, proximal, and distal tibia, bilaterally. The periodic fever gene panel analysis revealed homozygote mutations in exon 4 of the *LPIN2* gene; c.589C>T (p. Arg197*), like her brother. After the diagnosis of Majeed syndrome, anakinra was initiated at a dose of 2 mg/kg/day at the same time with her elder brother. Patient 2 has also remained free of symptoms with normal APRs on anakinra treatment. However, she still has neutropenia (900/mm^3^) four months after the diagnosis.

**Conclusion:** Majeed syndrome should be considered in children with joint and bone pain, anemia, and neutropenia especially in the presence of parental consanguinity or positive family history. Anti-IL-1 drugs seems to be effective in treating of Majeed syndrome-related inflammation while neutropenia seems to be unresponsive to this treatment.

**References**

1. El-shanti H, Ferguson P. Majeed Syndrome – Retired Chapter, For Genetic counseling Clinical Diagnosis. Published online 2021:1-11.

2. Ferguson PJ, Hedrich CM. *40 - Autoinflammatory Bone Diseases*. Eighth Edi. Elsevier Inc.; 2021. doi:10.1016/B978-0-323-63652-0.00040-9

**Patient Consent Received**

Yes

**Disclosure of Interest**

None declared

### P89 Amyloid arthropathy in a young girl with familial mediterranean fever

#### B. Kasap-Demir^1,2^, F. C. Sarıoğlu^3^, A. Kaya^4^

##### ^1^Department of Pediatric Nephrology and Rheumatology; ^2^Izmir Katip Çelebi University; ^3^Radiology; ^4^Orthopedisc and Traumatology, Health Sciences University Tepecik Training and Research Hospital, Izmir, Turkey

###### **Correspondence:** B. Kasap-Demir

**Introduction:** Aymloid arthropathy is characterized by infiltrative deposition of amyloid into articular and periarticular spaces. Although it has been more commonly reported in cases with rheumatoid arthritis, multiple myeloma and in those on chronic hemodialysis programme, it has rarely been reported in cases with familial mediterranean fever (FMF).

**Objectives:** Here we report a case with FMF complicated with aymloid arthropathy.

**Methods:** An 18-year-old girl was admitted with bilateral knee swelling. Her past medical history was remarkable for recurrent fever, abdominal pain and joint pain beginning at the age of 3 and was diagnosed with FMF at the age of 6. Genetic analysis revealed M694V homozygous mutation. Renal biopsy performed at the age of 10 upon proteinuria revealed amyloidosis. She had attacks every two weeks which were resistant to colchicine, thus canacinumab was initiated. Despite canacinumab, chronic kidney disease developed and she was put on peritoneal dialysis programme at the age of 13 and two years later she received a living unrelated kidney transplant.

At admission, she had bilateral swollen knees without warmth or hyperemia. All other physical examination findings were normal.

**Results:** Laboratory parameters were as follows: WBC 10,500/mm^3^, Hb 9.7 g/dL, MCV 66.3 fL, RBC 4.68 x 10^6^, plt 412,000/mm^3^, urea 25 mg/dL, serum creatinine 1.06 mg/dL, CRP 0.3 mg/L, ESH 14 mm/h, serum amyloid A: 0.75 mg/L (N<6). Knee MRI revealed widespread irregularity in the bony cortex at the level of the lateral malleolus of the femur and millimetric degenerative bone cysts in the tendency to merge were observed. There were heterogeneous contrast enhancements in degenerative bony cysts. Effusion was observed in the suprapateller bursa and intraarticular area. The findings were compatible with amyloid arthropathy. Non-steroid anti-inflammatory drugs were prescribed and colchicine dose was tried to increase.

**Conclusion:** The typical radiological findings should suggest amyloid arthropathy in cases who have predisposition to amyloidosis. This is the first case with amyloid arthropathy associated with FMF in the literature.

**Patient Consent Received**

No

**Disclosure of Interest**

None declared

### P90 Features of children with fmf and a defined variant in MEFV gene: a single center experience

#### S. Güneş Yılmaz^1^, B. Kasap-Demir^2,2,2,2,3,3,4,4^, E. Soyaltın^1^, G. Erfidan^5^, Ö. Özdemir Şimşek^5^, S. Arslansoyu Çamlar^5^, D. Alaygut^5^, F. Mutlubas^6^

##### ^1^Pediatrics, Health Sciences University Tepecik Training and Research Hospital; ^2^Pediatric Nephrology and Rheumatology, Izmir Katip Çelebi University; ^3^Pediatric Nephrology and Rheumatology; ^4^Pediatric Rheumatology; ^5^Pediatric Nephrology, Health Sciences University Tepecik Training and Research Hospital; ^6^Pediatric Nephrology, Izmir Katip Çelebi University, Izmir, Turkey

###### **Correspondence:** B. Kasap-Demir

**Introduction:** Familial Mediterranean Fever (FMF) is the most common monogenic autoinflammatory disease and autosomal recessive mutations in the MEFV gene are responsible for the clinical manifestations. The phenotype-genotype correlations have not been clarified definitively yet.

**Objectives:** This study aimed to present the demographic, clinical, and laboratory features of children clinically diagnosed with FMF and had a defined variant in at least one allele. The secondary aim was to predict more severe mutations by evaluating clinical findings.

**Methods:** We enrolled cases diagnosed with FMF according to Tel-Hashomer criteria and a defined variation in at least one allele, being followed up for at least 6 months. The medical charts of the patients were reviewed retrospectively. The patients were grouped as homozygous, compound heterozygous, and simple heterozygous cases with and without M694V mutation and the data were compared between the groups.

**Results:** A total of 263 (M/F:109/154) cases were included. The mean age at the onset of symptoms, follow-up duration, and time to diagnosis was 81.10±47.00 (3-204), 51.78±39.31 (6-166), and 9.23±14.44 (1-132) months, respectively. The rates of parental consanguinity, positive family history, and FMF in a first-degree relative were 15%, 42%, and 31.4%, respectively. The most common symptom was abdominal pain (85%). There was no difference between the growth parameters of the cases during the initial and final control periods. The most frequent alleles were M694V, E148Q, V726A. The most common accompanying disease was IgA vasculitis (20%). Almost 90% of the cases fulfilled all the defined criteria. Hb values ​​were lower and the ESR and CRP values ​​were higher during the attack period; ESR and CRP values were higher in the attack-free period; Pras scores were higher and the use of high dose colchicine was more frequent in homozygous and compound heterozygous cases carrying M694V. The presence of FMF in a first-degree relative increases the probability of this genetic predisposition 2.63 times; and each 1 unit increase in Pras score increases this probability 1.63 times. The threshold Pras score for this possibility is 5.5 (sensitivity: 65%, specificity: 55%).

**Conclusion:** M694V is the most common and severe mutation in our cohort. First degree relative with the disease and Pras scores ≥5.5 may predict an M694V homozygous or a compound heterozygous mutation.

**Patient Consent Received**

No

**Disclosure of Interest**

None declared

### P91 performance of Eurofever/PRINTO classification criteria to differentiate fmf from PFAPA

#### B. Kasap-Demir^1,2^, S. Güneş Yılmaz^3^, A. Kanık^4^

##### ^1^Pediatric Rheumatology, Health Sciences University Tepecik Training and Research Hospital; ^2^Pediatric Nephrology and Rheumatology, Izmir Katip Çelebi University; ^3^Pediatrics, Health Sciences University Tepecik Training and Research Hospital; ^4^Pediatrics, Izmir Katip Çelebi University, Izmir, Turkey

###### **Correspondence:** B. Kasap-Demir

**Introduction:** Eurofever/PRINTO classification criteria has been established to differentiate autoimmune diseases from each other.

**Objectives:** We aimed to differentiate cases with FMF and PFAPA with this new set of crtieria.

**Methods:** Patients diagnosed with PFAPA syndrome and FMF and followed up at Pediatric Rheumatology and Pediatrics outpatient clinics between March 2016 and March 2021 were included in the study. Patients were diagnosed with PFAPA syndrome regarding modified Marshall’s criteria, while patients with FMF were diagnosed using Yalçınkaya-Özen criteria. Regardless of their primary diagnosis, all the patients and their primary caregivers were exposed to a questionnaire including demographic and clinical data. MEFV gene mutations of the cases with FMF were noted. Homozygous or compound heterozygous pathogenic MEFV gene variants were defined as “confirmatory” and heterozygous pathogenic MEFV mutations, or compound heterozygous for one pathogenic MEFV variant and one variant of unknown significance (VUS), or biallelic VUS were defined as “non-confirmatory” genotype. Patients with heterozygous VUS or benign variants were defined as “non-pathogenic” and were not taken into consideration for Eurofever/PRINTO “genetic and clinical (CG)” criteria. The consistency between Eurofever/PRINTO “clinical only (CO)” and (CG) criteria was established. The effectivity of the CO criteria to differentiate cases with FMF from those with PFAPA, sensitivity, specificity, negative and positive predictive values (NP and PP, respectively) and accuracy of the CO criteria were calculated.

**Results:** 407 patients (M/F: 200/207) were included. Of those patients, 230 were diagnosed with FMF and 177 were diagnosed with PFAPA. In patients with FMF, 80 had confirmatory, 97 had non-confirmatory, and 53 had non-pathogenic mutations. When patients with FMF and PFAPA were compared, age at disease onset and diagnosis were significantly younger, male gender, pharyngotonsillitis, aphthous stomatitis, lymphadenitis, rinorrhea, cough, and febrile convulsion were more prevalent in cases with PFAPA, while abdominal and chest pain, maculopapular rash was more prevalent in cases with FMF (p<0.01). Disagreement between CO and CG criteria was established in only 5 of 177 (3%) patients with pathogenic mutations. The sensitivity, specificity, PP, NP and accuracy levels of CO criteria to differentiate FMF cases from PFAPA were 98.26%, 63.84%, 77.93%, 96.58%, and 83.29%, respectively. When evaluated considering MEFV gene groups, the highest sensitivity, specificity, PP, NP and accuracy were found in cases with “non-confirmatory” genotype. Since not all PFAPA cases have MEFV gene analysis, these values for CG criteria could not be defined for the whole group.

**Conclusion:** The sensitivity is higher, whereas the specificity and accuracy was lower for CO to differentiate FMF from PFAPA in our series when compared to the values established by Eurofever/PRINTO to differentiate FMF from other autoinflammatory diseases. Also, CO shows the best performance for cases with non-confirmatory mutations, which may cause diagnostic challenge for clinicians.

**Patient Consent Received**

No

**Disclosure of Interest**

None declared

### P92 Comparison of the performance of four different criteria to diagnose PFAPA

#### A. Kanık^1^, K. Sözmen^2^, B. Kasap-Demir^3,4^

##### ^1^Pediatrics; ^2^Public Health; ^3^Pediatric Nephrology and Rheumatology, Izmir Katip Çelebi University; ^4^Pediatric Rheumatology, Health Sciences University Tepecik Training and Research Hospital, Izmir, Turkey

###### **Correspondence:** B. Kasap-Demir

**Introduction: P**eriodic **f**ever, **a**phthous stomatitis, **p**haryngitis, and **a**denitis (PFAPA) syndrome is a periodic fever syndrome. No universally accepted criteria for PFAPA hasbeen developed yet. An international consensus has been established recently to define a new set of classification criteria for PFAPA syndrome.

**Objectives:** We aimed to evaluate the diagnostic validity of four different criteria for the diagnosis of PFAPA.

**Methods:** The patients diagnosed with PFAPA syndrome and Familial Mediterranean fever (FMF) and followed up at Pediatric Rheumatology and Pediatrics outpatient clinics of Tepecik Teaching Hospital, İzmir, Turkey between April 2016 and April 2021 were included in the study. Patients who fulfilled the modified Marshall’s criteria irrespective of age and responded to steroids during the follow up were recruited for PFAPA syndrome. Patients with FMF were diagnosed using Yalçınkaya-Özen criteria and were included as the control group. All the patients and their primary caregivers were exposed to a questionnaire including demographic and clinical data mentioned in the criteria sets. The agreement between the four criteria was established. In addition, the sensitivity, specificity, positive predictivity (PP), negative predictivity (NP) and accuracy levels were calculated for each criteria set.

**Results:** There were 418 patients (M/F: 203/215) included in the study. Of those patients, 238 were diagnosed with FMF and 180 were diagnosed with PFAPA. When evaluated according to four different sets of criteria, modified Marshall’s criteria misdiagnosed none, Vanoni criteria misdiagnosed 4, Takeuchi criteria misdiagnosed 34, and Eurofever/PRINTO classification criteria for PFAPA misdiagnosed 1 cases with FMF as PFAPA, while modified Marshall’s criteria missed 8, Vanoni criteria missed 4, Takeuchi criteria missed none, and Eurofever/PRINTO classification criteria for PFAPA missed 22 of the cases with PFAPA. The sensitivity, specificity, PP, NP and accuracy levels as % for the abovementioned criteria were as follows: 95.48, 100, 100, 96.75, 98.0 for modified Marshall’s; 97.74, 98.32, 97.74, 98.32, 98.07 for Vanoni; 100, 85.71, 83.89, 100, 91.81 for Takeuchi; and 87.57, 99.58, 99.36 91.51, 94.46 for Eurofever/PRINTO, respectively. The agreement between the four criteria set was “very good” (kappa: 0.834, CI: 0.832-0.835). The four sets of criteria agreed in 353 patients (all diagnosed 147 and ruled out PFAPA in 206 cases), while there was disagreement between the citeria in 65 cases.

**Conclusion:** Although all criteria sets defined for PFAPA have a high level of agreement between each other, the most recent Eurofever/PRINTO classification criteria has the lowest sensitivity in our series. Further studies in higher number of patients would be needed to verify our data.

**Patient Consent Received**

No

**Disclosure of Interest**

None declared

### P93 Blood pressure, arterial stiffness and left ventricular hypertrophy in FMF patients with confirmatory mutations

#### B. Kasap-Demir^1,2^, G. Erfidan^3^, T. Demircan^4^, Ö. Özdemir Şimşek^3^, C. Başaran^3^, S. Arslansoyu Çamlar^3^, D. Alaygut^3^, F. Mutlubas^5^

##### ^1^Pediatric Nephrology and Rheumatology, Izmir Katip Çelebi University; ^2^Pediatric Nephrology and Rheumatology; ^3^Pediatric Nephrology; ^4^Pediatric Cardiology, Health Sciences University Tepecik Training and Research Hospital; ^5^Pediatric Nephrology, Izmir Katip Çelebi University, Izmir, Turkey

###### **Correspondence:** B. Kasap-Demir

**Introduction:** Chronic inflammatory era may result in cardiovascular changes.

**Objectives:** We aimed to evaluate whether children with familial Mediterranean fever (FMF) have increased blood pressure (BP) values, higher arterial stiffness or left ventricular mass index (LVMI).

**Methods:** Patients diagnosed with FMF who have homozygous or compound heterozygous mutations in the exon 10 of the MEFV gene and being followed up between April 2020 and April 2021 were included in the study. Demographic data, office blood pressure (OBP) measurements were recorded at the first visit. Ambulatory blood pressure monitoring (ABPM), central BP (cBP), pulse wave velocity (PWV), and augmentation index (AIx@75) values recorded within the same device and LVMI calculated with echocardiographic measurements were recorded in the following visit. The same tests were performed for a sex and age-matched control group.

**Results:** Finally, data would be collected for 26 cases with FMF and 26 cases as the healthy controls. Body weight, height, body mass index SDS values were similar between the groups (p>0.05). Both systolic and diastolic OBP SDS values were similar as well (p>0.05). 24-hour, daytime and nighttime systolic and diastolic BPs and 24-hour mean arterial pressure (MAP) SDS and nighttime mean MAP SDS levels were similar between the groups (p>0.05). However, daytime MAP SDS was significantly higher in FMF patients (p:0.033). Both daytime systolic and diastolic loads were significantly higher in FMF cases (p=0.005 and p=0.034, respectively), whereas nighttime systolic and diastolic loads and systolic and diastolic dips were similar between the groups. 24-hour, daytime and nighttime AIx@75 and PWV levels, and systolic and diastolic central BP levels were similar between FMF patients and the control group (p>0.05).

**Conclusion:** The results of this preliminary study suggested that FMF cases with confirmatory mutations were prone to have higher daytime systolic BP and MAP, however negative effects of these disorders on the left ventricle and arterial stiffness could not be established. Evaluation of these parameters in larger patient groups and re-assessment of the same patients in the follow-up would be rather valuable.

**Patient Consent Received**

No

**Disclosure of Interest**

None declared

### P94 And quickly there were ten: DADA2, experience from a center in Mumbai, India

#### R. Khubchandani^1^, D. Ramadoss^1^, A. Khan^1^, P. Lee^2^, P. Pimpale Chavan^3,4^

##### ^1^Section of Pediatric Rheumatology, NH SRCC Children's Hospital, Mumbai, India; ^2^Division of Allergy, Immunology and Rheumatology, Boston Children's Hospital, Boston, United States; ^3^Former Pediatric Rheumatologist, NH SRCC Children's Hospital, Mumbai, India; ^4^Current : Inflammatory Disease Section, National Human Genome Research Institute, National Institutes of Health, Bethesda, United States

###### **Correspondence:** R. Khubchandani

**Introduction:** The number / phenotype of DADA2 continues to expand rapidly though series from Asia are scant.

**Objectives:** Share experience with 10 DADA2 patients (9 unrelated families) identified over 2 years.

**Methods:** We diagnosed the first case in April 2019 following which we recalled and diagnosed 4 more patients on renewed suspicion. In 2, their phenotypes did not match the initial provisional diagnosis of primary CNS vasculitis and inflammatory bowel disease (IBD) respectively while 2 had been treated as classic PAN. 4 patients were diagnosed prospectively on clinical suspicion and 1in whom we suspected syndromic bone dysplasia with inflammatory features was a diagnostic surprise.

**Results:** 7/10 are males. Age of onset ranged from 4 months – 17 years 9 months. Referrals were by varied specialists including primary pediatrician, pediatric hematologist, ophthalmologist, adult neurologist and urosurgeon. Medium-vessel dominated disease was seen in 6 patients and in 3 we suspected a systemic autoinflammatory disease (SAID) {1-febrile relative of a previously diagnosed DADA2 patient, 1-IBD-like with cutaneous vasculitis,1- early onset prolonged fever with granulomatous mediastinal adenitis suspected Blau syndrome} and 1 patient with progressive deforming symmetric inflammatory arthropathy and acquired micrognathia.

Cutaneous features were the commonest; seen in 7 patients and stroke was seen in 3. Other systems involved were musculoskeletal (5-including the bone dysplasia mimic described above),renal (4- notable were renal artery stenosis and perinephric hematoma), gastrointestinal (2- notable was bowel perforation), while ocular involvement was seen in 2 (notable being central retinal artery occlusion and episcleritis). Hematological features were seen in 5 and included pure red cell aplasia, persistent leucopenia and thrombocytopenia in 1 patient each and anemia in 2 (notable-unexplained anemia of infancy). None of the patients had exclusive hematological disease or immunodeficiency.

5 were homozygous for p.G47R variant and 2 are compound heterozygous with p.G47R and splice mutation c.753+2T>A and p.G47R and p.H219P respectively. Of those with p.G47R variant 4 belong to Agarwal community in whom endogamy is known. 2 patients born of a first cousin marriage (and even related three generations higher) have a homozygous pathogenic variant p.G358R. The patient with symmetrical skeletal affliction has a homozygous pathogenic variant in p.R169Q. All 4 patients in whom ADA2 enzyme assay was performed were deficient.

4 patients are on etanercept originator molecule and 6 on etanercept biosimilar with treatment duration varying between 2 weeks to 116 months and no drug side effects. 9 patients are in clinical remission off steroids and growing well with no restriction of activities of daily living. 2 have residual hypertension. 1 unvaccinated patient contracted COVID 19 and recovered uneventfully.

**Conclusion:** Since our first case in 2019, DADA2 is now the commonest SAID in our cohort (10/44). Due to its initial presentation to varied specialists we need to spread awareness to increase diagnosis. We report an unusual phenotype mimicking a bone dysplasia and alert colleagues that the classic phenotype originally described is being overshadowed by a wide spectrum. The p.G47R mutation is the commonest in our series and seen in the endogamous Agarwal community. The disease is very responsive to etanercept and treatment is progressively affordable with etanercept biosimilar. Residual hypertension may be seen with renal involvement and 1 patient with COVID19 on etanercept recovered uneventfully.

**Disclosure of Interest**

None declared

### P95 Long-term efficacy and safety of canakinumab in patients with hids (Hyper-igd syndrome) **−** interim analysis of the reliance registry

#### J. B. Kuemmerle-Deschner^1^, T. Kallinich^2^, J. Rech^3^, N. Blank^4^, J. Weber-Arden^5^, P. T. Oommen^6^

##### ^1^University Hospital, Tuebingen; ^2^Charite University Medicine, Berlin; ^3^University Hospital, Erlangen; ^4^University Hospital, Heidelberg; ^5^Novartis, Nuernberg; ^6^University Hospital, Duesseldorf, Germany

###### **Correspondence:** J. B. Kuemmerle-Deschner

**Introduction:** Autoinflammatory periodic fever syndromes such as the Hyper-IgD syndrome/mevalonate kinase deficiency (HIDS/MKD) are rare autoinflammatory conditions characterized by severe systemic and organ inflammation. Successful treatment with rapid remission of symptoms and normalization of laboratory parameters was achieved in most patients with the interleukin-1β inhibitor canakinumab (CAN) in clinical trials^1^ and real life. CAN has been approved and applied for the treatment of HIDS/MKD patients since 2017^2^.

^1^ De Benedetti F, et al. Canakinumab for the treatment of autoinflammatory recurrent fever syndromes. N Engl J Med 2018;378:1908–19

^2^ Ilaris, INN-canakinumab (europa.eu)

**Objectives:** To explore the long-term efficacy and safety of CAN under routine clinical practice conditions in pediatric (age ≥2 years) and adult HIDS/MKD patients.

**Methods:** RELIANCE is a prospective, non-interventional, multi-center, observational study based in Germany with a 3-year follow-up period. Patients with clinically confirmed diagnoses of TRAPS, CAPS, FMF or HIDS/MKD who routinely receive CAN are enrolled in order to evaluate efficacy and safety of CAN under standard clinical practice conditions at baseline and at 6-monthly intervals.

**Results:** The present interim analysis comprises baseline data of 7 HIDS/MKD patients enrolled by December 2020 as well as preliminary 12-month data. Of these patients, 4 (57%) were females and median age at baseline was 7 years (2-39 years). The median duration of prior CAN treatment at baseline was 2.0 years (0-5 years). Standard, low, and high dose CAN treatment was evenly distributed at every interval.

Preliminary results indicate stable remission and disease control by physicians´ and patients´ assessment as well as laboratory parameters (table 1). In total, 3 patients were affected by adverse drug reactions, however, none of these events was classified as serious.

**Conclusion:** Baseline characteristics and preliminary data of HIDS/MKD patients from the RELIANCE study indicate good clinical and laboratory disease control and no unexpected safety concerns at the 12 months interim analysis.

**Disclosure of Interest**

J. B. Kuemmerle-Deschner Consultant for: Novartis, AbbVie, Sobi, T. Kallinich Consultant for: Sobi, Novartis, Roche, J. Rech Consultant for: Abbvie, Biogen, BMS, Chugai, GSK, Janssen, Lilly, MSD, Mylan, Novartis, Roche, Sanofi, Sobi, UCB, Speaker Bureau of: Abbvie, Biogen, BMS, Chugai, GSK, Janssen, Lilly, MSD; Mylan, Novartis, Roche, Sanofi, Sobi, UCB, N. Blank Consultant for: Novartis, Sobi, Lilly, Pfizer, Abbvie, BMS, MSD, Actelion, UCB, Boehringer-Ingelheim, Roche, J. Weber-Arden Employee of: Novartis, P. T. Oommen: None declared

**Table 1 (abstract P95).** Baseline characteristics and interim analysis data of patients with HIDS

	Baseline (N=7)	6 months (N=6)	12 months (N=4)
Number (%*) of patients in disease remission (physician assessment)	4 (57.1)	5 (83,3)	3 (75.0)
Physician Global Assessment, percentage of absent/mild-moderate/severe rating	43 / 43 / 14	83 / 17 / 0	50 / 50 / 0
Patients´ assessment of current disease activity; 0–10, median (min; max)	0 (0; 7)	1.0 (0; 3)	0.5 (0; 8)
Patients´ assessment of current fatigue; 0–10, median (min; max)	2.0 (0; 7)	1.5 (0; 7)	2.5 (0; 4)
Number (%*) of patients without impairment of social life by the disease	2 (40)	4 (80)	3 (100)
Number (%*) of patients with days absent from work/school during last 6 months	2 (29)	3 (50)	0 (0)
CRP, median (mg/dl)	0.2	0.5	0.3
SAA, median (mg/dl)	0.6	0.9	0.8

*CRP* c-reactive protein, *SAA* serum amyloid A

*not reported for all patients

### P96 Long-term efficacy and safety of canakinumab in patients with traps (Tumor necrosis factor receptor-associated periodic syndrome) − interim analysis of the reliance registry

#### J. B. Kuemmerle-Deschner^1^, N. Blank^2^, C. Schuetz^3^, M. Borte^4^, P. T. Oommen^5^, J. Henes^1^, J. Weber-Arden^6^, T. Kallinich^7^

##### ^1^University Hospital, Tuebingen; ^2^University Hospital, Heidelberg; ^3^University Hospital, Dresden; ^4^Hospital St. Georg gGmbH, Leipzig; ^5^University Hospital, Duesseldorf; ^6^Novartis, Nuernberg; ^7^Charite University Medicine, Berlin, Germany

###### **Correspondecne:** J. B. Kuemmerle-Deschner

**Introduction:** Tumor necrosis factor receptor-associated periodic syndrome (TRAPS) is a rare autoinflammatory condition characterized by severe systemic and organ inflammation. In a phase 3 pivotal trial^1^, TRAPS patients have been successfully treated with the interleukin-1β inhibitor canakinumab (CAN). 45% of patients reached clinical remission after 16 weeks (primary endpoint)^1^. CAN has been approved and applied for the treatment of TRAPS patients since 2017^2^.

^1^ De Benedetti F, et al. Canakinumab for the treatment of autoinflammatory recurrent fever syndromes. N Engl J Med 2018;378:1908–19

^2^ Ilaris, INN-canakinumab (europa.eu)

**Objectives:** The present study explores the long-term efficacy and safety of CAN under routine clinical practice conditions in pediatric (age ≥2 years) and adult TRAPS patients.

**Methods:** RELIANCE is a prospective, non-interventional, multi-center, observational study based in Germany with a 3-year follow-up period. Patients with clinically confirmed diagnosis of TRAPS who routinely receive CAN are enrolled in order to evaluate efficacy and safety of CAN at baseline and at 6-monthly intervals.

**Results:** The interim analysis of TRAPS patients enrolled by December 2020 includes baseline (N=16, 1 patient with atypical TRAPS) and preliminary 18-month data. Of these patients, 11 (69%) were females and median age at baseline was 23 years (3-43 years). 10 (62.5%) patients had been pre-treated with anakinra and 1 (6.3%) with tocilizumab.

Preliminary results indicate stable remission by physicians´ assessment and laboratory parameters. Disease control by patients´ assessment showed no major changes (table 1). In total, 7 adverse drug reactions where observed, of which none was classified as severe.

**Conclusion:** Baseline characteristics and interim data of TRAPS patients are available from the RELIANCE study. Further interim and end-of-study data will be analyzed to assess efficacy and safety of long-term treatment as well as dosing effects in TRAPS patients.

**Disclosure of Interest**

J. B. Kuemmerle-Deschner Consultant for: Novartis, AbbVie, Sobi, N. Blank Consultant for: Novartis, Sobi, Lilly, Pfizer, Abbvie, BMS, MSD, Actelion, UCB, Boehringer-Ingelheim, Roche, C. Schuetz: None declared, M. Borte: None declared, P. T. Oommen: None declared, J. Henes Consultant for: Novartis, AbbVie, Sobi, Roche, Janssen, Boehringer-Ingelheim, J. Weber-Arden Employee of: Novartis, T. Kallinich Consultant for: Sobi, Novartis, Roche

**Table 1 (abstract P96).** Baseline characteristics and interim analysis data of patients with TRAPS

	Baseline (N=16)	6 months (N=13)	12 months (N=10)	18 months (N=6)
Median duration of prior CAN therapy at baseline, years (min; max)	1.0 (0; 4)	1.0 (0; 4)	1.0 (0; 4)	1.5 (0; 2)
Number (%*) of patients in disease remission (physician assessment)	9 (60.0)	9 (81.8)	7 (77.8)	4 (80.0)
Physician Global Assessment, percentage of absent/mild-moderate/severe rating	40 / 53 / 0	82 / 9 / 0	44 / 44 / 11	80 / 20 / 0
Patients´ assessment of current disease activity; 0–10, median (min; max)	1.5 (0; 5)	1.0 (0; 4)	1.0 (0; 6)	0.0 (0; 3)
Patients´ assessment of current fatigue; 0–10, median (min; max)	2.0 (0; 8)	1.0 (0; 7)	2,5 (0; 8)	4.0 (0; 7)
Number (%*) of patients without impairment of social life by the disease	4 (50)	5 (63)	2 (33)	3 (60)
Number (%*) of patients with days absent from work/school during last 6 months	8 (50)	5 (39)	5 (56)	3 (50)
CRP, median (mg/dl)	0.1	0.1	0.1	0.0
SAA, median (mg/dl)	0.5	0.4	0.4	0.3

*CRP* c-reactive protein, *SAA* serum amyloid A

^*^Numbers/percentage do not sum up to N=16/13/10/6/100%, due to missing data in some patients

### P97 Long-term safety and effectiveness of canakinumab in Cryopyrin-Associated Periodic Syndromes (CAPS) – 30-month data from the reliance registry

#### J. B. Kuemmerle-Deschner^1^, N. Blank^2^, B. Kortus-Goetze^3^, P. T. Oommen^4^, A. Janda^5^, J. Rech^6^, F. Weller-Heinemann^7^, G. Horneff^8^, I. Foeldvari^9^, C. Schuetz^10^, M. Borte^11^, A. Braner^12^, J. Weber-Arden^13^, T. Kallinich^14^

##### ^1^Pediatrics, University Hospital, Tuebingen; ^2^University Hospital, Heidelberg; ^3^University Hospital, Marburg; ^4^University Hospital, Duesseldorf; ^5^University Hospital, Ulm; ^6^University Hospital, Erlangen; ^7^Prof. Hess Kinderklinik, Bremen; ^8^Asklepios Clinic, Sankt Augustin; ^9^Centre for Pediatric and Adolescence Rheumatology, Hamburg; ^10^University Hospital, Dresden; ^11^Hospital St. Georg gGmbH, Leipzig; ^12^University Hospital, Frankfurt; ^13^Novartis, Nuernberg; ^14^Charite University Medicine, Berlin, Germany

###### **Correspondence:** J. B. Kuemmerle-Deschner

**Introduction:** In clinical trials as well as in real-life, the IL-1β inhibitor canakinumab leads to rapid remission of symptoms in the treatment of CAPS, a monogenic autoinflammatory disease with severe systemic and organ inflammation.

**Objectives:** The RELIANCE registry is designed to explore long-term safety and effectiveness of canakinumab under routine clinical practice conditions in pediatric (≥2 years) and adult patients with CAPS, including Muckle-Wells syndrome (MWS), familial cold autoinflammatory syndrome (FCAS), and neonatal onset multisystem inflammatory disease (NOMID)/chronic infantile neurological cutaneous and articular syndrome (CINCA).

**Methods:** This prospective, non-interventional, observational study with a 3-year follow-up enrolls patients with clinically confirmed diagnoses of CAPS routinely receiving canakinumab. In 6-monthly visits, clinical data, physician assessments and patient-reported outcomes are evaluated starting at baseline with last update at 30 months of follow-up in the total cohort including the cohort with severe subtypes (NOMID/CINCA).

**Results:** 91 CAPS patients (50% female; 14 [15%] NOMID/CINCA subtypes) were enrolled by December 2020 (table 1). At baseline, median age was 20.5 years and median duration of prior canakinumab treatment was 6 years. 20 drug related severe adverse events (11 per 100 patient years) were reported. 68% of patients reached disease remission by physicians´ assessment along with rates of 40-61% absent disease activity in PGA. CAPS was impairing social life in 50% of patients. Lab parameters were within normal limits.

**Conclusion:** The 30-month interim analysis of the RELIANCE study demonstrates that long-term canakinumab treatment is safe and effective in patients with any subtype of CAPS. However, impairment of social life still exists.

**Disclosure of Interest**

J. B. Kuemmerle-Deschner Consultant for: Novartis, AbbVie, Sobi, Speaker Bureau of: Novartis, AbbVie, Sobi, N. Blank Consultant for: Novartis, Sobi, Lilly, Pfizer, Abbvie, BMS, MSD, Actelion, UCB, Boehringer-Ingelheim, Roche, B. Kortus-Goetze Consultant for: Novartis, P. T. Oommen: None declared, A. Janda: None declared, J. Rech Consultant for: Abbvie, Biogen, BMS, Chugai, GSK, Janssen, Lilly, MSD, Mylan, Novartis, Roche, Sanofi, Sobi, UCB, Speaker Bureau of: Abbvie, Biogen, BMS, Chugai, GSK, Janssen, Lilly, MSD; Mylan, Novartis, Roche, Sanofi, Sobi, UCB, F. Weller-Heinemann: None declared, G. Horneff Speaker Bureau of: AbbVie, Bayer, Chugai, Merck Sharp & Dohme, Novartis, Pfizer, Roche, I. Foeldvari Consultant for: Novartis, C. Schuetz: None declared, M. Borte: None declared, A. Braner Consultant for: Novartis and SOBI, J. Weber-Arden Employee of: Novartis, T. Kallinich Consultant for: Sobi, Novartis, Roche

**Table 1 (abstract P97).** Patient and physician assessment of clinical CAPS disease activity and laboratory markers over time

	Baseline		12 months		30 months	
	Total cohort	NOMID/ CINCA	Total cohort	NOMID/ CINCA	Total cohort	NOMID/ CINCA
Number of patients, N	91	14	67	8	28	4
Number (%) of patients in disease remission (physician assessment)	61 (68.5)	11 (78.6)	42 (66.7)	4 (66.7)	19 (67.9)	4 (100.0)
Physician Global Assessment, percentage of absent/mild-moderate/severe rating, %	40 / 53 / 2	57 / 36 / 0	33 / 60 / 2	33 / 50 / 0	61 / 39 / 0	75 / 25 / 0
Patient assessment of current disease activity; 0–10, median (min; max)	2.0 (0; 7)	1.0 (0; 6)	1.0 (0; 7)	1.0 (0; 5)	0.0 (0; 7)	0.0 (0; 4)
Patient assessment of current fatigue; 0–10, median (min; max)	3.0 (0; 9)	2.0 (0; 6)	3.0 (0; 8)	2.0 (0; 8)	1.0 (0; 8)	4.0 (0; 5)
Number (%) of patients without impairment of social life by the disease	32 (52.5)	4 (50.0)	31 (62.0)	3 (42.9)	11 (47.8)	1 (33.3)
CRP/SAA, median (mg/dl)	0.1/0.3	0.2/0.4	0.1/0.5	0.5/0.9	0.0/0.3	0.2/0.1

*CRP* c-reactive protein, *SAA* serum amyloid A

### P98 Cryopyrin-associated periodic syndromes: gosh and national amyloidosis centre experience

#### O. Kul Cinar^1^, C. Papadopoulou^1,2^, A. Putland^1^, K. Wynne^1^, H. J. Lachmann^3^, D. Eleftheriou^1,2^, P. Brogan^1,2^

##### ^1^Paediatric Rheumatology, GREAT ORMOND STREET HOSPITAL FOR CHILDREN NHS TRUST; ^2^Infection, Immunity & Inflammation, UCL GOS Institute of Child Health; ^3^National Amyloidosis Centre, UCL Medical School, Royal Free Campus, London, United Kingdom

###### **Correspondence:** O. Kul Cinar

**Introduction:** Cryopyrin-associated periodic syndrome (CAPS) is a rare, heterogenous inflammasomopathy associated with gain-of-function mutations in *NLRP3*. Mutations in *NLRP3* result in excessive IL-1ß production that may underlie wide range of symptoms including skin rashes, conjunctivitis, arthritis, and sensorineural deafness. IL-1 blockade demonstrated complete responses to treatment which has been life-changing in this monogenic inflammasomopathy.

**Objectives:** We aimed to demonstrate the disease activity scores, inflammatory markers, genetic mutations, and MRI brain of the CAPS patients followed by GOSH and the National Amyloidosis Centre, encompassing a very large cohort of paediatric patients, including adolescents now transitioned to adult care. Data collection was carried out before and after treatment with IL-1 inhibitors.

**Methods:** Patients followed-up in GOSH and NAC specialist CAPS clinics from 2005 to 2021 were identified. Data on following parameters collected: disease subtype, presenting symptoms, CAPS disease activity scores, serum amyloid A (SAA) and C-reactive protein (CRP) levels, MRI brain; and treatment. Statistical analysis was performed by GraphPad Prism version 9.1.1.

**Results:** A total of 48 patients [female (n=20, 42%), male (n=28, 58%)] with CAPS diagnosis in childhood/adolescence were identified. Median age at disease presentation was 3.50 years (range:0.20–16.23). Disease subtypes were: CINCA (n=4), MWS (n=37), FCAS (n=6), CAPS-like disease (n=1). Two of 6 patients with FCAS harboured *NLRP3* variants of unknown significance, and were discharged since they ultimately proved asymptomatic after review. Clinical symptoms were recorded and CAPS disease activity score was calculated in each clinic visit. There was a significant drop in the mean CAPS activity scores between first and last visits (8.66/20 (±2.59), 1.12/20 (±1.29), respectively). Median treatment duration was 5.83 years (range:0.17–15.83). Mutations could not be detected by sanger sequencing in 6/48 (12,5%), although clinical characteristics of CAPS were identified. Two/6 had next-generation sequencing without evidence of mosaicism. The commonest mutation was p.A439V in *NLRP3* gene (19/48), followed by p.V198M (3/48), p.T348M (3/48) and p.R488K (3/48) mutations. CRP and SAA levels were checked prior to treatment and at each clinic visit. 45 of 48 patients (94%) were on anti-IL1 treatment: 42/45 (93%) canakinumab, 27 of whom were switched from anakinra; and 3/45 (7%) on anakinra. Two FCAS patients were discharged from clinic without any treatment, 1 patient with compound heterozygous *IRAK4* mutations in addition to *NLRP3* p.E457D was switched from canakinumab to tocilizumab, with complete clinical and serological response. Median CRP before and after treatment were 5.0 mg/L (range:1.0-83.0) and 5.0 mg/L (range:1.0–51.0). Median SAA before and after treatment were 8.5 mg/L (range: 2.4 – 680.0) and 3.50 mg/L (range: 2.0 – 222.0). Thirteen of 48 (27%) patients underwent MRI brain due to neurological involvement, mainly due to recurrent headaches, with no abnormalities identified in 9/13 (70 %); whereas 4 patients (3 with CINCA) had changes on their MRI brain. None of the patients experienced deterioration neither in their clinical symptoms nor in MRI brain scans after starting anti-IL1.

**Conclusion:** Anti-IL1 treatment has had a major impact in paediatric patients for the prevention and treatment of CAPS symptoms. Treatment efficacy was observed by improved CAPS clinical disease activity scores; and normalised inflammatory markers. In our cohort, neurological symptoms including sensorineural hearing problems improved and MRI brain scans have remained stable with anti-IL1 therapies.

**Disclosure of Interest**

None declared

### P99 Serum calprotectin as a marker of disease activity in the assessment of the paediatric patient with autoinflammatory disease: a cross-sectional study

#### M. Marti Masanet^1^, M. I. González Fernández^1^, B. López Montesinos^2^, L. Lacruz Perez^2^, A. Alba Redondo^3^, B. Laiz Marro^3^, I. Calvo Penadés^2^

##### ^1^Pediatric Rheumatology Unit, Medical Research Institute Hospital La Fe; ^2^Pediatric Rheumatology Unit; ^3^HOSPITAL Universitari i Politecnic La Fe, Valencia, Spain

###### **Correspondence:** M. Marti Masanet

**Introduction:** Serum calprotectin (S100A8/9 or MRP8/14) is considered a good plasmatic marker for the assessment of systemic-onset JIA (sJIA) and other autoinflammatory diseases (AID).

**Objectives:** To evaluate the use of serum calprotectin in a paediatric population with autoinflammatory disease for assessment of disease activity and to correlate it with other blood inflammatory parameters.

**Methods:** Patients who fulfilled International League of Association for Rheumatology Criteria for sJIA and patients with defined genetic or clinically diagnosed AID were included. Serum calprotectin and other inflammatory markers (CRP, ESR, serum amyloid A, ferritin) were tested in a blood sample from the patients, MRP8/14 was tested by ELISA Kit sandwich of Bülmann (automated in an immunoassay analyzer DSX). Physician global assessment of disease activity and parent/patient global assessment of well-being were measured on a 0-10 Visual Analogue Scale (VAS). Sample was divided into active and inactive cases at the time of calprotectin testing: sJIA patients were considered active if they had active joint count and/or systemic symptoms (such as fever and rash) and patients with AID diagnosis were considered active if they had typical symptoms of disease activity.

Graphpad Prism Version 9.0 was used for data analyses. Between-group comparisons were done by Mann-Whitney U test. Correlations were studied with Spearman’s test. Receiver operating characteristic curve (ROC) analysis was done to assess the use of an inflammatory parameter to differentiate patients active than inactive.

**Results:** 84 patients were included in this study, 40% females and 60% males. Diagnosis, number of active/inactive patients and serum calprotectin levels (μg/mL) are described in table 1.

Association between MRP8/14 and disease activity status was analysed. Globally (including sJIA and AID together), active patients had higher levels of MRP8/14 than inactive patients (p=0.0031). For FMF patients, serum calprotectin levels were higher in the active group but not statistically significant (p= 0.06). For sJIA patients, levels were also higher in the active group and statistically significant (p= 0.044), but in this case the sample was very small. It should be noted that PAPA patient had by far the highest value of serum calprotectin, despite being inactive.

Low positive correlation between MRP8/14 and CRP was found (r=0.34), that was statistically significant (p=0.01), as well as between MRP8/14 and ferritin (r=0.39; p=0.0064). Low positive correlations were found between each serum calprotectin/CRP/amyloid A and physician VAS, that were statistically significant. The best correlation was with calprotectin (r=0.388; p=0.0006).

Globally, we analyzed the use of serum calprotectin to differentiate between active and inactive patients. Compared to CRP and serum amyloid A, MRP8/14 showed better ability to differentiate activity (AUC=0.69). We obtained a cut-off value of 2.26 μg/mL for serum calprotectin, with 55,56 % sensitivity and 87,18% specificity.

**Table:** 

				Serum calprotectin (μg/mL) Median (IQR)	
	N	active	inactive	active	inactive
FMF	35	13	22	3.19 (1.3-4.6)	1.31 (0.49-1.86)
TRAPS	10	6	4	2.37 (1.44-4.85)	1.49 (1.14-1.96)
HIDS-MVD	8	7	1	1.76 (0.6-2.3)	-
PFAPA	11	3	8	1.52 (0.97-5.26)	1.32 (1-2.02)
CAPS	5	3	2	4.54 (4.4-8.4)	1.75 (1.2-2.3)
PLAID	1	1	0	2.57	-
PAPA	1	0	1	-	27
Systemic-onset JIA	13	2	11	10.14 (7.7-12.57)	1.65 (0.75-2.12)

**Conclusion:** Although our sample is small and we need to increase the number of patients to obtain stronger evidence, our study showed the potencial role of serum calprotectin in the assessment of paediatric patients with AID and sJIA.

**Patient Consent Received**

Yes

**Disclosure of Interest**

None declared

### P100 A case report of haploinsufficiency of A20 in a russian patient with Behҫet-like disease in pediatric rheumatogist practice

#### V. Matkava, S. Salugina, I. Nikishina, A. Shapovalenko, E. Fedorov, Z. Kolkhidova

##### Pediatric Department, V. A. Nasonova Research Institute of Rheumatology, Moscow, Russian Federation

###### **Correspondence:** V. Matkava

**Introduction:** A20 Haploinsufficiency (HA20) is a recently described autoinflammatory disease (AID) caused by a loss-of-function mutation in the TNFAIP3 gene. HA20 can be described like rare autosomal-dominant syndrome with early onset of systemic inflammation. Clinical manifestations of HA20 are similar to Behҫet’s disease (BD) and represented by periodic fever, recurrent oral aphthosis and genital ulcers, arthralgia/arthritis, uveitis. In world practice there are a few described cases of gastrointestinal involvement (abdominal pain, diarrhea, vomiting, rectorrhagia). Elevated acute-phase markers and immune reactants (ANA, anti-dsDNA, anti-Sm, RNP) and positive HLAB51 have been observed in some patients.

**Objectives:** To present clinical manifestations of Behҫet-like syndrome (HA20), initially diagnosed as an rheumatic disease.

**Methods:** Case report of patient with HA20 genetically confirmed by high-throughput DNA sequencing and detection TNFAIP3 gene mutation.

**Results:** An adolescent girl (16 у.о.) was examined in our Federal Rheumatologic Centre. From the age of 14, disease manifested with repeated episodes of oral and genital ulcerations, arthritis of proximal and distal interphalangeal joints (PIP, DIP), CRP was 124 g/l (Normal=0-5 mg/l). Initially diagnosis was verified as juvenile idiopathic arthritis (JIA). The treatment included subcutaneous methotrexate (MTX) 15 mg/week, NSAID courses without positive respond. Since 15 y.o., hips and ankles arthritis were developed, deformation of PIP, DIP joints were getting worse. BD, JIA were included in differential conditions. Because persistence of symmetric polyarticular damage, increased ESR, CRP - abatacept was prescribed, without any response. Gastrointestinal involvement (erosive enteritis, endocolitis), loss of 9 kg weight for 6 months and diffuse alopecia were detected after 1.5 years from the onset of the disease. A lot of laboratory disturbance including ESR-46 mm/h (N= 2,0-30,0); ANA-1/2560 (N<1/160); anti-dsDNA-23.9 IU/ml (N= 0,0-20,0); lymphopenia (0,72x109/l, N= 1,20-3,00), anemia (HGB 111 g/l, N=120-140) was preserved. Overlap-syndrome (JIA and Systemic Lupus Erythematosus) was discussed. Rituximab (RTX) 500 mg №1 and glucocorticoids (GC) 15 mg/day of methyl prednisolone have been started with improvement. Despite of reversing polyarthritis, there were relapses of ulcerations and joining a disorder of the psychoemotional state. High-throughput DNA sequencing was performed due to atypical disease course. The mutation c.591_593delTGT in the TNFAIP3 gene in a heterozygous state was detected. The treatment has been continued (MTX, GC, RTX) with quite good clinical response. At first time have been found high levels of Anti-CCP - 84,7 u/ml (N= 0,0-5,0). The girl is under long-term follow-up nowadays.

**Conclusion:** Rare genetic AID with early-onset systemic inflammation including HA20 can be determined in rheumatologist’s practice like common rheumatic disease.

**Patient Consent Received**

Yes

**Disclosure of Interest**

None declared

### P101 Clinical characteristics of children with chronic rheumatological presentations associated with pathogenic and variants of unknown significance in the NOD2 gene: the gosh experience

#### K. Mclellan, K. Nott, A. Shivpuri, C. Papadopoulou, S. Compeyrot-Lacassagne

##### Paediatric Rheumatology, Great Ormond Street Hospital, London, London, United Kingdom

###### **Correspondence:** K. Mclellan

**Introduction:** Blau syndrome describes an autoinflammatory granulomatous condition comprising a triad of dermatitis, uveitis and synovitis caused by gain-of-function mutations in NOD2. Genetic testing can also identify variants of unknown significance (VUS) the role of which in pathogenesis is unknown. Recent reports suggest that some VUS in patients with Early Onset Sarcoidosis (EOS) might be pathogenic.

**Objectives:** To describe the phenotype of children with chronic rheumatological disorders and NOD2 mutations.

**Methods:** Retrospective case note review.

**Results:**

1. Three groups identified; 3 patients (Group A) with an EOS phenotype with pathogenic mutations in NOD2 gene; A334G, H603A & G498A (the latter’s affected mother also had the same mutation). 3 patients (Group B) with an EOS phenotype with VUS in NOD2 (2 sisters with mutations in P268S & 1 with A725G) and 11 patients (Group C) with a heterogeneous presentation and VUS in NOD2; 2 patients with severe uveitis, 2 with hepatic sarcoidosis (1 of whom had uveitis), 2 with severe polyarticular JIA, (1 with fever, without rash or uveitis), 2 with chronic rashes and 2 with systemic JIA.
2. Demographics: In Group A, median age of symptom onset 2 years (IQR,1.75-5.63), Group B median age of symptom onset 1.33 (1.08-5.25) and Group C: median age of symptom onset 4.83 (2.88-11.25). 67% in Group A Caucasian, 33% South Asian. 100% in Group B Black. Group C; 45% Caucasian, 27% Black, 18% Mixed Race and 9% Middle Eastern. 1/17 patients had a history of consanguinity; in Group A with H603A variant. Median follow-up duration 4.33 years (2.67-6.17).
3. Clinical features: summarised in Table 1.
  Rash: Group A: 2/3 patients (1 erythema nodosum, 1 scaly erythematous plaques); Group B 3/3 patients (100% nodular rash)
  Renal involvement: 100% patients in Group B (67% proteinuria, 67% raised tubulo-interstitial markers, 67% raised urine: creatinine ratio)
  Pulmonary involvement: 2 patients in the entire cohort; both in Group B with interstitial lung disease (1 with variant P268S & 1 with A725G).
  No patients had significant cardiac or vascular involvement or hearing loss.
  Systemic inflammation: 100% patients in Group A had a raised erythrocyte sedimentation rate (ESR) at presentation (median 32mm/hr (IQR 22.5-46), Group B: ESR 22mm/hr (20-44) and Group C: ESR 28mm/hr (11-105). 1/3 in Group A had a raised angiotensin converting enzyme (ACE) at presentation median value 49U/L (IQR 37.5-55.5), 3/3 in Group B median 270U/L (IQR 167.5-345).
4. Tissue biopsy: 2/3 in Group A (skin: non-necrotising granulomatous inflammation; synovium: non- specific chronic synovitis), 3/3 in Group B (synovium, skin and lymph node showing non-necrotising granulomas).
5. Management: 88% received steroids, methotrexate was given to 100% in Group A received methotrexate, 67% in Group B and 55% in Group C. 100% in Group A & B received biologics (anti-TNF), with biologics used in 55% of Group C.

**Table:** 

Group (no. patients(%))	Arthritis (clinically/radiologically)	Tenosynovitis	Rash	Fever (>38^**0**^C)	Lymphadenopathy (clinically/ultrasonography)	Uveitis	Hepatic involvement (hepatomegaly/deranged liver function)	Pancreatic involvement (biochemical markers)	Splenic involvement (splenomegaly)
**A(n=3)**	3(100)	3(100)	2(67)	1(33)	1(33)	3(100)	2(67)	1(33)	1(33)
**B(n=3)**	3(100)	0(0)	3(100)	2(67)	3(100)	3(100)	3(100)	2(67)	2(67)
**C(n=11)**	4(36)	1(9)	5(45)	3(27)	5(45)	3(27)	4(36)	3(27)	1(9)

**Conclusion:** With greater availability of genetic testing, variants of unclear significance are identified. We plan to test the activity of these NOD2 variants to assess their significance. Increased genetic testing of patients with relevant phenotypes and advances in genomics may improve understanding of the role of these variants in these complex diseases.

**Patient Consent Received**

No

**Disclosure of Interest**

None declared

### P102 Recurrent fevers and septal panniculitis in aicardi- goutières syndrome caused by TREX1 gene mutation with response to baricitinib

#### K. Mclellan, P. Brogan, D. Eleftheriou, E. Moraitis

##### Paediatric Rheumatology, Great Ormond Street Hospital, London, London, United Kingdom

###### **Correspondence:** K. Mclellan

**Introduction:** Aicardi-Goutieres syndrome (AGS) is a heterogenous disorder in terms of phenotypical expression and severity. Mutations in *TREX1* are associated with early-onset encephalopathy with basal ganglia calcification, familial chilblain lupus, retinal vasculopathy with cerebral leukodystrophy.

**Objectives:** To describe a rare presentation of AGS *TREX1* mutation with a predominantly systemic autoinflammatory phenotype and response to baricitinib.

**Methods:** Retrospective case notes review.

**Results:** A 21 month old girl presented with a 9 month history of recurrent fevers lasting 3-5 days, approximately every 14 days, associated with arthralgia, urticarial rashes, blistering panniculitic lesions on hands, legs and face and irritability. She had mild delay in gross motor skills and speech.

She had been born to Caucasian non-consanguineous parents at 35+4 weeks gestation. She developed a persistent cough from five weeks of age and and was treated for bilateral lower respiratory tract infections secondary to pseudomonas aeruginosa. Subsequently unsafe swallow was noted and she was fed via a nasogastric tube. Bronchoscopy, video fluoroscopy, bulbar EMG, and cystic fibrosis screening were performed, all with negative results. There was a family history of chilblains in father and paternal grandmother.

Laboratory parameters revealed normal full blood count and elevated inflammatory markers during the episodes with CRP 24 mg/L (reference range [RR] < 20 mg/L), erythrocyte sedimentation rate (ESR) 45 mm/hr (RR < 10), serum amyloid A 100mg/L (RR < 10), with normalisation between episodes. A skin biopsy of the rash showed features of urticaria and septal panniculitis, but no vasculitis. An extensive work up for infections, autoimmune diseases and common immunodeficiencies was negative. Ophthalmic examination was normal. Echocardiogram and magnetic resonance imaging of brain were normal, and an ultrasound of the abdomen showed a mildly coarse liver. CT scan brain showed left frontal lobe calcification.

A systemic neuro-autoinflammatory disease was suspected, leading to genetic screening with targeted next-generation sequencing. Empirical treatment with colchicine 0.5mg/kg/day was commenced and subsequently azathioprine 2 mg/kg/day pending genetic results. No benefit was observed from the treatment with colchicine or azathioprine, and oral prednisolone (1mg/kg for 3 days) was introduced with improvement in shortening duration of fever episodes. Motor skills and language remained delayed, and there was a decline in neurological function with mild spasticity in the lower limbs.

Genetic studies revealed a heterozygous c.G217A mutation (p.D73N) in *TREX1* gene confirming a diagnosis of AGS. Expression of interferon-stimulated genes was found to be upregulated. Treatment with baricitinib (2 mg three times a day (0.34mg/kg/day)), a selective JAK1/2 inhibitor, was initiated at age 4 years. At 5 months follow up post initiation of baricitinib there was significant improvement in the clinical features, with only one episode of fever and mild rash which lasted 2 days over a 5 month period. The neurological function remained stable.

**Conclusion:** We describe a rare autoinflammatory phenotype as the predominant manifestation of AGS caused by a heterozygous mutation in *TREX1* and highlight the response to baricitinib. Recognition of this phenotype and early diagnosis is of outmost importance as prompt initiation of treatment may be of greatest benefit in the early stages of the disease. The patient has shown clinical response to JAK inhibition, although we cannot comment on the long term benefits at preventing progression of neurological manifestations.

**Patient Consent Received**

Yes

**Disclosure of Interest**

None declared

### P103 Chronic recurrent multifocal osteomyelitis refractory to various immunosuppressive therapy:case description

#### Z. Nesterenko, A. Kozlova, V. Burlakov, A. Moiseeva, S. Dibirova, J. Rodina, A. Horeva, A. Roppelt, A. Shcherbina

##### Dmitry Rogachev National Research Center of Pediatric Hematology, Oncology and Immunology, Moscow, Russian Federation

###### **Correspondence:** Z. Nesterenko

**Introduction:** Chronic recurrent multifocal osteomyelitis (CRMO) is a rare mostly multifactorial autoinflammatory disease. It is characterized by non-infectious recurrent bone lesions, usually manifesting in childhood. There is no standard and universal treatment approach that is able to control disease progression in all patients with CRMO.

**Objectives:** We aim to report our positive experience with treatment of a refractory CRMO case with tocilizumab.

**Methods:** We describe clinical, laboratory, imaging characteristics and treatment response of a CRMO patient. Known genetic defects, leading to CRMO, were excluded via whole exome sequencing. The diagnosis was confirmed histologically, bone tumors and infections were excluded.

**Results:** A 7-year-old girl was admitted to our hospital because of pain in the right knee and left ankle, accompanied by fever and increased laboratory inflammatory markers (C-reactive protein (CRP), erythrocyte sedimentation rate (ESR). Treatment with NAID, antimicrobial therapy was not effective. She was started on bisphosphonate therapy, with minimal reduction of the clinical symptoms. TNF-α inhibitors (adalimumab, then infliximab) were ineffective. Steroids (2 mg/kg/day) + methotrexate relived her pain, as well as led to normalization of CRP levels, yet all the symptoms relapsed with steroids tapering.

Therefore, she was started on anakinra with partial effect. Two days after cessation of anti-IL-1 therapy she relapsed with exudative and proliferative lesions in the knee, ankle joints, joints of the wrists, and palmar-plantar pustulosis (erythema) on the feet

Canakinumab 150 mg, later 300 mg every 4 weeks, was initiated and the patient's general condition improved, but increased acute phase reactants and pain persisted. Denosumab, nuclear factor kappa-B ligand (RANKL) inhibitor, had been added to the сanakinumab and MTX therapy, without. MRI and bone scintigraphy showed persistent inflammatory changes in the bones. She was then started on tocilizumab and MTX. Now the patient is 8 months on treatment, is clinically asymptomatic, has no fever and pain. Laboratory tests showed decrease inflammatory activity.

**Conclusion:** Our experience in treating a patient with CRMO demonstrates the insufficient effectiveness of previous therapy with bisphosphonate, TNF inhibitors, IL1 inhibitors, denosumab. Improvement of the condition was achieved on therapy with an IL6 inhibitor, which requires additional monitoring and study.

**Patient Consent Received**

Yes

**Disclosure of Interest**

None declared

### P104 Long term visual outcome of blau-associated uveitis in a single centre study

#### A. V. Ramanan^1^, S. K. Ng^1^, P. Sinnappurajar^1^, C. M. Guly^2^

##### ^1^Paediatric Rheumatology, Bristol Royal Hospital for Children; ^2^Bristol Eye Hospital, University Hospitals Bristol NHS foundation Trust, Bristol, United Kingdom

###### **Correspondence:** S. K. Ng

**Introduction:** Blau Syndrome is a rare dominantly inherited autoinflammatory disease resulting from a mutation of a pattern recognition receptor NOD2 gene and typically manifest as a triad features of arthritis, dermatitis and uveitis with a paediatric onset. Several case series have reported that ocular manifestation is a frequent presentation (70-80%) and is often a bilateral disease with progression towards panuveitis in a long run. Management of uveitis in Blau Syndrome has been particularly challenging due to the chronicity and persistent uveitis despite systemic corticosteroids and institution of immune modulatory and biologic treatments. Combination of persistent active ocular disease, with post-inflammatory ocular complications and long term topical steroids, inevitably result in progressive decrease in visual acuity. The visual burden of patients suffering from Blau-associated uveitis is essentially unknown. There is lack of a published data assessing long term visual outcomes of patients with Blau-associated uveitis and the data published so far are based on case series without a standardized follow up.

**Objectives:** Sight threatening complications remains as one of the major factor of long term morbidity in patients with Blau syndrome. In this review, we aim to identify and quantify the visual prognosis of Blau syndrome uveitis in a retrospective longitudinal review of a single centre study.

**Methods:** Clinical data were collected retrospectively from patients with a diagnosis of Blau Syndrome associated uveitis with a proven NOD2 mutations attending the regional South West of England Paediatric Rheumatology and Ocular Inflammatory Service, Bristol Eye Hospital. Patients who had at least more than 5 year review were included in this study. Data was collected at standard time intervals ; at baseline, 1,3,5,10,15,20,25 and 30 years post diagnosis. General demographics, laterality of the uveitis, age of onset, anatomical classification and course of uveitis were recorded for each patient. Ocular disease activity, ocular complications and surgical interventions required were recorded for each patient.

**Results:** A total of 16 eyes of 8 patients ( 4 female, 4 male) with genetic proven NOD-2 mutation Blau Syndrome associated uveitis patients who had at least more than 5 year review were included in this study. The median age of systemic symptoms (arthritis / skin manifestation) was 1.25 years and the median age of uveitis diagnosis in 7 out of 8 patients known was 4.67 years. Two (25%) out of eight patients present with unilateral eye involvement. All of the eight patients on follow-up eventually develop bilateral eye involvements with one eye presented with phthisis bulbi at first review. Mean visual acuity at the last clinic visit was 0.26 logMAR ; range -0.1-NPL]). 4 out of 16 eyes (25%) had logMAR ≥0.3 and 4 out of 16 eyes (25%) had logMAR ≥1.0 or worse.

12 eyes (75%) developed cataract, 1 patient had both eyes developed ocular hypotony, 2 eyes had glaucoma with an eye needing glaucoma tube surgery. 3 eyes in 2 patients developed ocular hypertension. 6 out of 8 patients continues to have persistent anterior chamber inflammation till last review.

**Conclusion:** Blau-associated uveitis confers a major long term morbidity with a quarter patient developing severe visual impairment.

**Patient Consent Received**

No

**Disclosure of Interest**

None declared

### P105 Severe recurrent oral ulcers - is there a genetic cause?

#### M. Niemuth^1^, A. Gabrielyan^1^, J. Fischer^2^, M. Laass^1^, R. Berner^1^, J. Roesler^1^, N. DiDonato^2^, M. A. Lee-Kirsch^1,2^, C. Schuetz^1^

##### ^1^Department of Paediatrics; ^2^Department of Genetics, Medical Faculty Carl Gustav Carus, Technische Universität, Dresden, Germany

###### **Correspondence:** M. Niemuth

**Introduction:** In 2016 an early-onset autoinflammatory disorder with a clinical phenotype resembling Behcet's disease was described by Zhou et al. (1) caused by heterozygous loss-of-function mutations in the *TNFAIP3* gene encoding A20. Haploinsufficiency of A20 (HA20) decreases the NF-kB regulatory protein A20 and thereby amplifies action of the transcription factor NF-kB, a central mediator within inflammatory and innate immune signaling pathways.

**Objectives:** This review/case report is to provide additional information on clinical presentation and genetic findings in a rare, only recently, described autoinflammatory disorder.

**Methods:** A 10y/o girl repeatedly presented with severe oral ulcers, gingivitis, lymphadenopathy and occasional fevers. Serum levels of C-reactive protein (CrP) and erythrocyte sedimentation rate (ESR) were mildly increased, Immunglobulin E levels considerably elevated. No ulcers were found by endoscopy in stomach and colon, but few intramucous lymphoid follicles were seen. Interestingly, her mother also suffers from recurrent oral ulcers in addition to lung emphysema, autoimmune hepatitis of unknown origin, and scleroderma.

**Results:** Whole exome sequencing in the index patient revealed a novel heterozygous mutation in the TNFAIP3 gene (NM_006290.4:c.176_177delAG, p.Gln59fs). This variant leads to a frameshift and a premature STOP codon at the beginning of exon 2 and thereby most probably to nonsense mediated decay. Mutations in this gene can vary in location and usually lead to STOP codons or frame shift mutations.

Due to the decreased A20 levels and hence reduced inhibition of NF-kB, patients with HA20 suffer from episodes of fever, recurrent oral and genital ulcers, skin rashes, and polyarthritis, as well as from gastrointestinal and neurological symptoms. Ocular manifestations are far less frequent than in Behcet’s disease, and age at manifestation is usually earlier.

**Conclusion:** In patients with severe “familial” aphthosis and unexplained fever, genetic testing may guide clinical treatment decisions as exemplified in this family with unexpected monogenic disease. Our patient is presently stable under colchicine (1 mg/d) and on-demand steroid treatment. TNF-alpha-inhibitors and IL-1 antagonists may control ulcers, arthritis and sterile abscesses in patients with HA20. In the presence of systemic autoimmunity and an elevated interferon signature, JAK-inhibitors are a therapeutic option as stated by Schwartz et al. (2).

Consent for publication was signed by the patient's parents.

**References**

(1) Zhou Q, Wang H, Schwartz DM, Stoffels M, Park YH, Zhang Y, et al. Loss-of-function mutations in TNFAIP3 leading to A20 haploinsufficiency cause an early-onset autoinflammatory disease. Nat Genet. 2016;48(1):67–73.

(2) Schwartz DM, Blackstone SA, Sampaio-Moura N, Rosenzweig S, Burma AM, Stone D, et al. Type I interferon signature predicts response to JAK inhibition in haploinsufficiency of A20. Ann Rheum Dis. 2020;79(3):429–31.

**Patient Consent Received**

Yes

**Disclosure of Interest**

None declared

### P106 Expanding the autoinflammatory phenotype of sideroblastic anemia with immunodeficiency, fevers and development delay (SIFD) syndrome

#### F. Orlando^1^, M. Tardi^1^, D. De Brasi^1^, R. Naddei^2^, R. Borrelli^1^, M. Alessio^2^, L. Martemucci^1^

##### ^1^Department of Pediatrics, AORN Santobono Pausilipon; ^2^Department of Translational Medical Sciences, Section of Pediatrics, University of Naples Federico II, Naples, Italy

###### **Correspondence:** F. Orlando

**Introduction:** *TRNT1* is a nuclear gene encoding a ubiquitous enzyme (CCA-adding tRNA nucleotidyltransferase enzyme) necessary for aminoacylation of both mitochondrial and cytosolic tRNA. Mutations of that gene were firstly associated to SIFD but over the years phenotypic heterogeneity was described.

**Objectives:** To expand autoinflammatory phenotype of SIFD and to report a novel mutation of *TRNT1* gene.

**Methods:** Case report of a 10-years-old female admitted to our Rheumatology Pediatric Unit of Santobono Children's Hospital of Naples due to febrile illness associated with vomit and diarrhoea, evolved in shock, treated with broad spectrum antibiotics and cardiovascular support in Intensive Care Unit. No infective causes were found.

**Results:** At the admission, the anamnesis revealed recurrent episodes of fever since the second month of life treated with antibiotics even without evidence of infection, with poor clinical response. She underwent to haematological investigations due to microcytic anaemia requiring blood transfusions. Bilateral cataract was diagnosed at the age of one years, ascribed to perinatal infection of Cytomegalovirus. Physical examination evidenced facial dysmorphisms, brittle hair, intellectual disability, failure to thrive. Laboratory assessment showed microcytic anemia (Haemoglobin 9.4 g/dl, MCV 60.9 fL, RDW 43 fL), lymphopenia (Lymphocytes 619/uL), elevated inflammatory markers (C-Reactive Protein 324 mg/L, Procalcitonin 610 ng/ml, Ferritin 2071 ng/ml). Immunological assessment was performed, showing hypogammaglobulinemia with IgA <0.22 g/l (0.5-3) and IgG 6,3 g/dl (7-15), low levels of CD3+ T cells 49% (55-78%). The clinical history plus the phenotype and the laboratory data, led us to assume mutations of TRNT1 gene. Genetic analysis was done, confirming our suspicion. Sanger sequencing was performed for genetic confirmation, resulting for *TRNT1* mutations in SFID in compound heterozygous status in the proband: c.1205_1206dupAA (p.Glu403Lysfs*27) and c.1246A>G (p.Lys416Glu), the first mutation never described in literature, the second one already reported as pathogenic. She required low-dose prednisone to control a new febrile episode and then she started on anti-TNFα therapy (Etanercept), with resolution of recurrent fever and improvement of laboratory inflammation.

**Conclusion:** Since the first publication on SIFD, several studies have described patients with heterogeneous phenotypes and systemic involvement of variable severity and progression. Our patient presented a clinical picture characterized by several features which until the age of ten had not been considered as a part of a single disease. Currently, about 50 cases are outlined in literature. We also reported a novel mutation in the *TRNT1* gene (c.1205_1206dupAA) and even if functional analysis of the protein expression was not performed, the in-silico prediction led to consider the mutation as pathogenic. Taking into account the clinical and immunological phenotype altogether with the found mutations and response to treatment, we conclude that the patient is affected by SIFD syndrome. Due to the rarity of that syndrome, diagnostic delay is observed, as in our patient. Early diagnosis of this condition would enable patients to promptly access to therapies thus allowing the opportunity of a better outcome.

Written informed consent for the publication was obtained.

**Patient Consent Received**

Yes

**Disclosure of Interest**

None declared

### P107 Real-life data from the largest pediatric familial mediterranean fever cohort

#### K. Öztürk^1^, T. Coşkuner^2^, E. Bağlan^3^, H. E. Sönmez^4^, G. Otar Yener^5^, F. Çakmak^6^, F. G. Demirkan^6^, A. Tanatar^6^, S. G. Karadağ^7^, S. Özdel^3^, F. Demir^2^, M. Çakan^8^, N. Aktay Ayaz^6^, B. Sözeri^2^ on behalf of PeRA-Research Group

##### ^1^Pediatric Rheumatology, Istanbul Medeniyet University Prof. Dr. Süleyman Yalçın City Hospital; ^2^Pediatric Rheumatology, University of Health Sciences, Ümraniye Research and Training Hospital, Istanbul; ^3^Pediatric Rheumatology, University of Health Sciences, Dr. Sami Ulus Maternity and Child Health and Diseases Research and Training Hospital, Ankara; ^4^Pediatric Rheumatology, Kocaeli University, Faculty of Medicine, Kocaeli; ^5^Pediatric Rheumatology, Şanlıurfa Research and Training Hospital, Şanlıurfa; ^6^Pediatric Rheumatology, Istanbul University, Faculty of Medicine, Istanbul; ^7^Pediatric Rheumatology, Erzurum Regional Research and Training Hospital, Erzurum; ^8^Pediatric Rheumatology, University of Health Sciences, Zeynep Kamil Maternity and Children's Diseases Training and Research Hospital, Istanbul, Turkey

###### **Correspondence:** K. Öztürk

**Introduction:** Familial Mediterranean fever (FMF) is the most common monogenic autoinfammatory disease manifesting with phenotypic heterogeneity. It is a clinically diagnosed disease supported by MEditerranean FeVer (MEFV) gene mutation analysis. However, the phenotype–genotype correlation is not yet established clearly.

**Objectives:** We aimed to determine the clinical findings, phenotype-genotype correlation and treatment outcomes within a large pediatric FMF cohort.

**Methods:** The medical charts of children with FMF who were diagnosed and followed up at the eight pediatric rheumatology units were reviewed retrospectively. All patients in the cohort were analyzed for sequence variants in exon 2,3,5 and 10 of the MEFV gene. Patients without any mutations or with polymorphisms including R202Q were excluded.

**Results:** A total of 3454 children (1755 girls, 1699 boys) were involved in the study. The mean±standard deviation of current age, age at symptom onset, and age at diagnosis were 12.1±5.2, 5.1±3.8, and 7.3±4.0 years, respectively. Of 3454 patients, 88.2% had abdominal pain, 86.7% had fever, 27.7% had arthritis, 20.2% had chest pain, 23% had myalgia and 13.1% had erysipelas-like erythema. The most common MEFV mutation patterns were homozygous (32.5%) and heterozygous (29.9%) mutations of exon 10. Homozygous M694V was present in 969 patients (28.1%). Allele frequencies of common mutations were M694V (n=3373, 55.3%), M680I (n=782, 11.3%), V726A (n=529, 7.6%) and E148Q (n=503, 7.2%). Children carrying homozygous or compound heterozygous mutations had an earlier age of disease onset (4.6 vs 5.6 years, p=0.000) and a higher number of attacks per year (11.1 vs 9.6, p=0.001). Although 8% of the patients had a family history of amyloidosis, 0.3% (n=11) had presence of amyloidosis. M694V homozygosity was detected in nine patients who developed amyloidosis. Colchicine resistance was present in 4.2% (n = 145) of our patients.

**Conclusion:** In this largest pediatric cohort studied and presented since now, it has been demonstrated that exon 10 mutations, particularly the M694V homozygous mutation, are important in disease severity and outcome. Although E148Q is considered as a polymorphism in some populations, it was identified as a disease-causing mutation in our cohort. Secondary amyloidosis is still happening in adults however, it is extremely rare among children, presumably due to increased awareness, tight control and the availability of anti-IL1 agents in colchicine-resistant cases.

**Patient Consent Received**

Yes

**Disclosure of Interest**

None declared

### P108 NLRC4-associated periodic autoinflammatory syndrome, phenotype and genotype of two clinical cases with novel variants

#### N. Palmou- Fontana^1^, C. Alvarez^2^, G. Ocejo-Viñals ^3^, J. M. Garcia-aznar^4^, B. Jimenez^2^, A. Tejerina-Puente^2^, D. Prieto^1^, M. J. Cabero^2^, M. A. Gonzalez-Gay^1^

##### ^1^Rheumatology; ^2^paediatrics; ^3^Iinmunology, Hospital Universitario Marques De Valdecilla, Santander; ^4^Inmunology, Healthincode, A Coruña, Spain

###### **Correspondence:** N. Palmou- Fontana

**Introduction:** To date, fewer than 20 variants with demonstrated pathogenicity in the NLRC4 gene associated with the development of autoinflammatory syndrome due to gain-of-function (GoF) of the NLRC4 inflammasome have been described. This autosomal dominant disorder encompasses a spectrum,from cold-induced relapsing fevers (FCAS) to neonatal-onset multisystem autoinflammatory disease (NOMID), autoinflammatory syndrome with infantile enterocolitis (AIFEC) or macrophage activation syndrome (MAS). In most cases, the onset of the disease occurs during early infancy with recurrent fever, and rash Treatment varies according to the degree of presentation, being key the identification of severe forms, associated with macrophage activation and secretion of pro-inflammatory cytokines (IL-1β and IL-18) for disease control with targeted drugs.

**Objectives:** In this study, we report two clinical cases in which the genetic study revealed two novel variants in NLRC4

**Methods:**

23-year-old woman with a history of self-recurrent episodes of oral aphthous ulcers and odynophagia treated with antibiotics since the age of 4 years. At age 21, she presented mononucleosis followed by a generalized skin rash, pharyngotonsillitis, fever of 39°C, being diagnosed with scarlet fever. Currently, she refers to have recurrent episodes, usually 2 or 3 a month, of oral aphthous ulcers, abdominal pain and pharyngotonsillitis, along with increase of acute phase reactants, treated with glucocorticoids. They are mainly associated with stress.

Girl aged 2 years and 5 months, with recurrent fever every 15 days (fever up to 39-40°C for 4-5 days) with pharyngotonsillitis in the last 11 months. The febrile episodes are associated with a marked elevation of acute phase reactants. She had experienced an immediate response to methylprednisolone in two of the three episodes in which they were administered. Her father had similar symptoms during childhood that disappeared with tonsillectomy

**Results:** The genetic study revealed two heterozygous variants not previously described in NLRC4. Case 1 carried the p. Cys258Arg variant (Allelic Freq<0.01%), absent in her asymptomatic mother, located within the NACHT-NBD domain, in which the presence of GoF variants has not been statistically associated with mild or severe forms of the disease. Case 2 carried the p. Glu311Lys variant (Allelic Freq. unknown), inherited from the affected father during childhood and absent in her asymptomatic mother, which is located within a region of the NACHT domain (between NACHT-NBD and NACHT-WHD), in which the presence of GoF variants has been found in a more significant number of patients with severe forms of the disease (AIFEC/MAS).

**Conclusion:** Nowadays, these two variants remain uncertain clinical significance, although potentially associated with the development of NLRC4 inflammasomopathy. Nevertheless, the genetic study has been of prognostic utility to identify genotype-phenotype correlations in other cases in the scientific literature with variants in NLRC4, because of that, if its pathogenicity is confirmed in these two cases, its result allows us to anticipate severe forms of the disease and to evaluate targeted treatment options.

**Patient Consent Received**

Yes

**Disclosure of Interest**

None declared

### P109 Focal myositis – a case series of a rare cause of hip immobility and calf Pseudotumor in children

#### F. Patel^1^, A. Sridhar^1^, A. F. Sharaf^2^

##### ^1^Paediatric rheumatology; ^2^Department of radiology, University Hospitals of Leicester NHS trust, Leicester, United Kingdom

###### **Correspondence:** F. Patel

**Introduction:** Case 1 -

A systemically well 7 year-old girl presented with 5-weeks of right calf tenderness and swelling following a short episode of pharyngitis and generalised maculopapular rash. There was no gait abnormality, focal neurology or restriction in activity aside from fatigability on walking distances. There were no skin rashes, joint involvement, eye changes or involvement of other muscles. She had a raised creatine kinase, plasma viscosity and lactate dehydrogenase. Her other blood results were normal including an extended autoimmune screen, immunoglobulins, complement levels, ASOT and titres of mycoplasma, EBV and CMV. MRI showed evidence of extensive inflammation of the gastrocnemius and soleus. A muscle biopsy showed heavy interstitial inflammatory cell infiltrate of predominantly lymphocytes, features of fibre necrosis including phagocytosis and hyalinisation with concurrent fibre regeneration (figures 1+2). She was initially managed with physiotherapy and anti-inflammatory medications but then developed intermittent right calf pain, restriction in activity and tiptoe walking due to gastrocnemius contractures. She was commenced on an 8-week tapering course of oral steroids and is improving with weekly methotrexate.

Case 2-

A systemically well 14-year-old presented with 6-months of left-sided hip pain, weight loss and inability to weight-bear without crutches. On examination there was painful fixed limitation of the left hip to 45^o^ on abduction and external rotation with bilateral mild swelling of the proximal interphalangeal (PIP) joints on both upper limbs. Otherwise, there was a full range of movement in all joints, with no rashes or other joint swelling or inflammation. Her blood tests were ANA positive 1:6000 and MRI of her hips demonstrated high T2 signal intensity in the left gluteus minimis and medius, obturator internus, obturator externus in keeping with myositis (figure 3). She received a pulse of corticosteroids followed by a course of methotrexate. There was immediate improvement in her PIP joint swellings and within a few weeks she was able to walk without crutches for the first time in 6 months. Unfortunately, 18 months after her diagnosis, she had developed anterior uveitis of her left eye with posterior synechiae; this responded well to steroid and cyclopentolate eye drops.

**Objectives:** .

**Methods:** .

**Results:** .

**Conclusion:** Summary-

Focal myositis is a rare immune-mediated pseudotumour of a single skeletal muscle group ^(1)^. Only around 200 cases have been described in the literature so little is known on incidence, prevalence, patient management and outcomes ^(2)^. This differs and should not be confused with post-viral myalgia which bears neither the histological changes nor chronicity of focal myositis. Treatment options are centred on immunomodulation and in severe cases surgical management of contractures ^(3)^.

We emphasise that clinicians should bear this rare differential diagnosis for in mind for consideration of early conservative management, assessment for uveitis, immunomodulation and possibly surgical correction to improve patient outcome.

References

1. Auerbach A, Julie CFS, Wang G, Rushing EJ. Focal myositis a clinicopathologic study of 115 cases of an intramuscular mass-like reactive process. Am J Surg Pathol. 2009;33: 1016–1024.

2. Devic P, Gallay L, Streichenberger N, Petiot P. Focal myositis: a review. Neuromuscul Disord. 2016 Nov;26(11):725-733.

3. Milani GP, Mazzoni MBM, Gatti H, et al. Recurrent focal myositis in childhood: a case report and systematic review of the literature. Pediatr Neurol 2017;71:77.e1–81.e1

**Trial registration identifying number:** Figure 1 – Coronal magnetic resonance imaging of the calves showing hypertrophy and inflammation of the medial head of the right gastrocnemius muscle compared to the left (White asterisk).

Figure 2 – Muscle biopsy stained with H+E shown at x40 (left) and x400 magnification (right). Heavy interstitial inflammatory cell infiltrate of predominantly lymphocytes, features of fibre necrosis including phagocytosis and hyalinisation with concurrent fibre regeneration is shown.

Figure 3 - T2-weighted coronal magnetic resonance imaging of the hips showing high T2 signal intensity in the left gluteus minimis, gluteus medius, obturator internus and obturator externus in keeping with myositis (yellow arrows). Minimal T2 high signal noted in the left acetabulum, iliac bone and left femoral head (white arrow) likely secondary to patient positioning artefact rather than true marrow oedema.

**Patient Consent Received**

Yes

**Disclosure of Interest**

None declared

### P110 Interferon inflammation in a case with PAMI syndrome and possible relationship with gene expression

#### A. Pin^1^, A. Tesser^1^, M. Girardelli^1^, S. Federici^2^, C. Celani^2^, P. Tomietto^3^, D. Stolfo^4^, A. Taddio^1,5^, A. Insalaco^2^, A. Tommasini^1,5^

##### ^1^Institute for Maternal and Child Health IRCCS "Burlo Garofolo", Trieste; ^2^IRCCS Ospedale Pediatrico Bambino Gesù, Roma; ^3^ASUGI, Rheumatology Unit; ^4^ASUGI, Department of Cardiolody; ^5^University of Trieste, Trieste, Italy

###### **Correspondence:** A. Pin

**Introduction:** Pathogenic heterozygous variants in *PSTPIP1* lead to excessive IL1 mediated inflammation, which is the cause of rare autoinflammatory syndromes: PAPA (pyogenic arthritis, pyoderma gangrenosum, acne) and the more recently described PAMI (PSTPIP1-associated myeloid-related proteinemia inflammatory) (1).

Gene expression characterization may lead to better understand the molecular pathogenesis of these diseases.

**Objectives:** We investigated the gene expression pattern in two patients with familial PAPA syndrome (father and son, pt1 and pt2), two siblings with PAMI syndrome (pt3 and pt4) and a sporadic case of PAMI syndrome in a girl who presented an atypical clinical picture (pt5).

**Methods:** Gene expression studies of whole blood cells of patients with PAPA and PAMI syndrome compared to a group of healthy subjects and pathway over-representation analysis to investigate the main biological processes involved.

**Results:**

**Table:** 

Family	Patient (pt)	***PSTPIP1*** mutations	Clinical symptoms	Treatment	Reference
Family-1 (PAPA)	Pt1-father	A230T	Arthritis, acne	Anakinra	(2)
	Pt2-son	A230T	Arthritis	Canakinumab	
Family-2 (PAMI)	Pt3-brother	E250K	Leukopenia, neutropenia, acne	Anakinra	(3)
	Pt4-sister	E250K	Leukopenia, neutropenia	Anakinra	
Family-3 (PAMI)	Pt5	E250K	Leukopenia, Atypical: SLE-like features (autoantibodies, nephritis, pulmonary arterial hypertension)	Hydroxychloroquine (HCQ)	

Table above describes the analyzed families including the results of target sequencing panels and pharmacological treatments.

Gene expression analysis highlighted the heme metabolism pathway which is over-expressed among all the patients compared to controls already known to be involved in physiological and pathological processes (4). Only patients with PAMI syndrome showed among the over-represented signaling pathways the neutrophils degranulation pathway probably due to neutrophiles hyper-activation. Interestingly, only pt5 presented a high interferon signature score that recovered after therapy with HCQ.

**Conclusion:** The presence of lupus-like manifestations in PAMI has never reported so far. Even if only one of our patients with PAMI displayed interferon-related-inflammation, we can speculate a possible cross-talk between IL1 and interferon pathways. A possible hypothesis, which could be worth investigating, is that the increased neutrophil activation pathway found in PAMI could be associated to the release of neutrophil extracellular traps and to the activation of interferon signaling.

**References**

1. Holzinger D, Fassl SK, de Jager W, Lohse P, Röhrig UF, Gattorno M, et al. Single amino acid charge switch defines clinically distinct proline-serine-threonine phosphatase-interacting protein 1 (PSTPIP1)-associated inflammatory diseases. J Allergy Clin Immunol. 2015;136(5):1337-45.

2. Cortis E, De Benedetti F, Insalaco A, Cioschi S, Muratori F, D'Urbano LE, et al. Abnormal production of tumor necrosis factor (TNF) -- alpha and clinical efficacy of the TNF inhibitor etanercept in a patient with PAPA syndrome [corrected]. J Pediatr. 2004;145(6):851-5.

3. Belelli E, Passarelli C, Pardeo M, Holzinger D, De Benedetti F, Insalaco A. Haematological involvement associated with a mild autoinflammatory phenotype, in two patients carrying the E250K mutation of PSTPIP1. Clin Exp Rheumatol. 2017;35 Suppl 108(6):113-5.

4. Wu B, Wu Y, Tang W. Heme Catabolic Pathway in Inflammation and Immune Disorders. Front Pharmacol. 2019;10:825.

**Patient Consent Received**

Yes

**Disclosure of Interest**

None declared

### P111 A peculiar phenotype of ADA2 deficiency

#### M. Rossano, F. Baldo, S. Torreggiani, L. Baselli, S. Giliani, S. Lanni, A. Petaccia, G. Filocamo, F. Minoia, C. Agostoni

##### Fondazione IRCCS Ca’ Granda, Ospedale Maggiore Policlinico, ^2^ASST Spedali Civili di Brescia, Milan, Italy

###### **Correspondence:** M. Rossano

**Introduction:** ADA2 deficiency (DADA2) is an autosomal recessive disease that causes a monogenic vasculitis syndrome characterized by protean manifestations, including haematological, cutaneous, articular and vascular involvement. Diagnosis is often difficult, due to the large phenotypic variability and the indolent course of the disease.

**Objectives:** To report an unusual case of ADA2 deficiency presenting with myositis, due to a new compound heterozygosity for a missense mutation and a novel deletion in *ADA2.*

**Methods:** A 7-year old boy was investigated for intermittent bilateral leg pain. Clinical evaluation was unremarkable, with normal strength. Blood tests revealed elevated inflammatory markers and normal lactic dehydrogenase (LDH) and creatinkinase (CK). Moderate lymphopenia was observed with low count of both CD19+ and NK cells, elevated CD3+HLA-DR+ cells, and low levels of IgM; double negative αβ T lymphocytes were normal (Table 1). MRI presented bilateral signs of myositis of the lower limbs. He subsequently developed nocturnal fever, multiple inguinal, axillary lymphadenopathy and splenomegaly. Infectious workup, the autoimmunity panel including myositis specific and associated autoantibodies, and serum markers of malignancies were all negative. PET-scan showed multiple tracer uptake in axillary, inguinal and abdominal lymph nodes (SUV max 2.2). A myogenic pattern of active denervation was confirmed by electromyography. Muscle biopsy did not reveal clear sign of vasculitis, only showed non-specific signs of inflammation with scarce macrophagic infiltration; no alteration of muscle fibres or perifascicular atrophy. Bone marrow and lymph node biopsy with flow cytometry were negative for malignancy.

Symptomatic treatment with indomethacin was started because of extremely painful musculoskeletal involvement associated to the development of recurrent infiltrative burning nodular skin lesions of both hands and feet. The result was a dramatic and prompt relief, but persistence of elevated acute phase reactants.

Genetic sequencing of ***ADA2*** revealed compound heterozygosity for a novel frameshift deletion in the catalytic domain, c.1100_1113del:p.I367Tfs*41, inherited from the father, and a maternally inherited missense variant, **c.1148G>A:p.G383D**, previously described in compound heterozygosity in two patients with DADA2. ADA2 functional analysis confirmed pathological low activity of the enzyme. Brain angio-MRI was unremarkable. Etanercept treatment was deployed with rapid response of muscoloskeletal and skin involvement, normalization of inflammatory markers and regression of lymphoproliferation.

**Results:**

**Table:** 

Weeks from disease onset	4	6	14	16	21	43	46	56	61
Therapy			Indomethacin start			Etanercept start			
CRP (mg/dl)	2,64	3,19	5,13	2,08	0,9	2,53	0,05	0,05	0,05
ESR (mm/h)	52	63	52	40	35	44	13	4	2
Hb (g/dl)	11,1	9,9	9,9	9,4	10,2	9,5	10,3	11,3	12
WBC (cell/mm3)	5400	6100	7050	4990	5060	5540	4650	3260	4020
N (cell/mm3)	3760	4270	4760	2840	2910	3320	2210	1050	1890
L (cell/mm3)	850	750	1390	1380	1310	1470	1790	1570	1440

**Conclusion:** Here, we report a case of an unusual presentation of DADA2 with myositis and an optimal response to the anti-TNF α therapy. We described a new compound mutation with a novel ADA2 deletion leading to premature protein truncation in heterozygosity with the recently described c.1148G>A mutation. The pathogenic role of the latter, so far reported in only two siblings in compound heterozygosity, is supported by our data. DADA2 phenotype spectrum is constantly increasing and description of peculiar cases and new mutations could improve genotype/phenotype correlation leading to significant progresses in diagnosis, management and prognosis of these patients.

**Patient Consent Received**

Yes

**Disclosure of Interest**

None declared

### P112 Family case of SAVI-syndrome in the practice of a rheumatologist and pulmonologist

#### S. Salugina^1^, E. Fedorov^1^, N. Lev ^2^, S. Zhikrivetskaya ^3^, A. Shapovalenko^1^, A. Torgashina^4^, O. Golovina^4^

##### ^1^Children, V. A. Nasonova Research Institute of Rheumatology; ^2^Veltishev Research and Clinical Institute for Pediatrics of the Pirogov Russian National Research Medical University of the Russian Ministry of Health; ^3^Veltishev Research and Clinical Institute for Pediatrics of the Pirogov Russian National Research Medical University of the Russian Ministry of Health; ^4^V. A. Nasonova Research Institute of Rheumatology, Moscow, Russian Federation

###### **Correspondence:** S. Salugina

**Introduction:** SAVI-syndrome – STING-associated vasculopathy with onset in infancy, a rare monogenic autosomal-dominant autoinflammatory disease associated with a mutation in the TMEM173 gene; belongs to type 1 interferonopathies. It is characterized by an early onset, fever, skin rashes (vasculopathy), arthritis, interstitial lung disease (ILD), increased levels of acute phase markers, and presence of autoantibodies (ANA, RF and other antibodies). The main treatment: glucocorticoids (GC), JAK inhibitors

**Objectives:** To present a family case of SAVI-syndrome in the practice of a rheumatologist and pulmonologist

**Methods:** Family case: three patients from the same family were sequentially hospitalized at the federal rheumatology center and diagnosed with SAVI-syndrome: 2 children-twins (2018 y.o.b.), the 1^st^ pregnancy (IVF), the 1^st^ cesarean operation (the girl was examined at the age of 1 year and 7 months, the boy – at the age of 2 years and 3 months), the children’s father (1980 y.o.b.) was diagnosed at the age of 41. All the three of them were revealed to have a genetic mutation in the TMEM173 gene c.463G> A, p.V155M in a heterozygous state. No mutation was found in the mother. Prior to the diagnosis, all these patients were monitored by a pulmonologist

**Results:** The main characteristics of the pts are presented in Table 1. Earlier than all, the manifestation of the disease was noted in the boy: shortness of breath from the birth, ILD, up from 1.5 months - skin rash, up from 2 years - ankle arthritis, changes in fingers like drumsticks and nail plates like watch glasses. The girl from the age of 1.5 years has suffered from skin rash, arthritis of the knees, ankles, small joints of the hands, fever, lymphadenopathy, enlarged liver, spleen, ILD in the absence of shortness of breath and manifestations of a respiratory failure. The father had a manifestation of a pulmonary pathology in the form of shortness of breath, decreased exercise tolerance from 9 years old, cutaneous vasculopathy up from 20 years old, arthritis of the knee, ankle, hand joints up from 24 years old, interstitial pulmonary involvement, fibrosis established at the age of 29. Treatment: prednisolone (Pr), cyclophosphamide, rituximab without any effect. The children were not treated. In all the patients during the course of the disease there was an increase in ESR, CRP; autoantibodies were detected. GC at a dose of 1 mg/kg in terms of Pr, tofacitinib - 5 mg per day were prescribed to the children; Pr - 5 mg per day, tofacitinib -10 mg / day were prescribed to the father. The children’s treatment duration lasted for 8 months, the father’s treatment - 1 month. During the treatment, the children showed positive dynamics in terms of the systemic inflammatory response. It is not yet possible to assess the changes in the lungs.

**Conclusion:** Pts suffering from the SAVI-syndrome can be encountered in the practice of a rheumatologist and a pulmonologist. Pts with an early onset, with systemic manifestations (fever, skin rashes, lymphadenopathy, hepatolienal syndrome), arthritis in combination with ILD, increased ESR, CRP, and the presence of autoantibodies require special attention. A family history is crucial to identify similar cases in relatives and to perform a genetic testing

**Patient Consent Received**

Yes

**Disclosure of Interest**

None declared

**Table 1 (abstract P112).** Clinical and demographic characteristics of patients from the same family with SAVI- syndrome

	Sister 2,5 y	Brother 2,5 y	Father 41 y
Gender	Female	Male	Male
Age at onset (years old)	1,5	From birth	9
Fever	++	+	+
Rash	+	+	++
Arthritis	+	+	+
Respiratory insufficiency	-	+	+
ILD	++	++	++
ESR, CRP	+	+	+
Autoantibodies	ANA+	АNA+	RF+,ANA+,Аnti-CCP+

### P113 Biological therapy for monogenic autoinflammatory diseases - experience of using it in rheumatological practice

#### S. Salugina, E. Fedorov, M. Kaleda

##### Pediatrics, V. A. Nasonova Research Institute of Rheumatology, Moscow, Russian Federation

###### **Correspondence:** S. Salugina

**Introduction:** Monogenic autoinflammatory diseases (MAIDS) are a heterogeneous group of rare genetically determined conditions, the main manifestations of which are episodes of fever in combination with other signs of a systemic inflammatory process: skin rash, musculoskeletal, neurological and other clinical symptoms often imitating a rheumatic pathology, acute phase markers, lack of autoantibodies. The most common and well-studied are 4 MAID: FMF, TRAPS, HIDS/MKD, CAPS. The use of a biological therapy in pts with MAIDS, especially IL-1 inhibitors, has led to a significant progress in the supervision of such patients.

**Objectives:** To present the experience of the Federal Rheumatology Center of using biological drugs (BD) in the treatment of pts with MAID.

**Methods:** For the period from 2008 to 2020 the study included 153 pts with MAID, of whom 48 were prescribed BD, among them 10 pts with FMF, 24–with CAPS (MWS-20, CINCA/NOMID - 4); TRAPS-11; HIDS/MKD - 3. The patient’s characteristics are presented in table 1. These were 20 male and 28 female pts aged from 1.5 to 38 years old, 40 children (under 18) and 8 adults. Interleukin-1 inhibitors were administered subcutaneously: canakinumab at a dose of 2-4 mg / kg or 150 mg per injection every 4-8 weeks, anakinra 1-3 mg / kg or 100 mg per day on a daily basis. TNF inhibitors were injected subcutaneously: etanercept at a dose of 0.4-0.8 mg / kg 1-2 times a week, adalimumab 20-40 mg once every 2 weeks. Tocilizumab was administered intravenously at a dose of 8-12 mg / kg once every 2-4 weeks. The disease duration at the time of the treatment beginning ranged from 1 to 44 years. The duration of treatment with BD for pts with MAID ranged from 3 months to 12 years.

**Results:** For 48 pts with MAID (31.4%) BD were used in the treatment, more often in pts with CAPS (47.1%), TRAPS (45.8%) and HIDS/MKD (50%), less often for pts with FMF (13.9%). Among BD IL-1 inhibitors (89.6%) were predominantly prescribed: canakinumab (33) and anakinra (10), which were used in half of pts with CAPS (50.9%), in a third of pts with TRAPS and HIDS (33.3 %), in 9.7% of patients with colchicine-resistant FMF. Against the background of treatment with IL-1 inhibitors, all the pts with MAID within the first few days showed a significant clinical improvement: normalization of well-being, significant improvement in mood, emotional uplift, relief of fever, disappearance of rash, decrease in the severity of lymphadenopathy and hepatosplenomegaly, relief of eye symptoms or significant positive dynamics, subjective improvement in hearing and audiogram with a further dynamic control in pts with CAPS, a decrease in the level of acute phase markers in all. In 7 patients with CAPS, receiving anakinra, after a significant positive response was achieved, switched to canakinumab, while maintaining the full efficiency. Less often tocilizumab (14.6%) and TNF-inhibitors (20.8%) were administered: adalimumab in 3, etanercept in 4, mainly in FMF and TRAPS pts with a positive response. The tolerability of therapy with BD was satisfactory in all the pts.

**Conclusion:** In pts with MAID BD are successfully used, more often for pts with CAPS, TRAPS and HIDS/MKD, less often for pts with FMF. Among them, IL-1 inhibitors predominate, which have shown high efficiency and good tolerance, especially in pts with CAPS.

**Patient Consent Received**

Yes

**Disclosure of Interest**

None declared

**Table 1 (abstract P113).** The characteristics of patients with MAIDS

	FMF(10)	CAPS(24)	TRAPS(11)	HIDS/MKD(3)	Total (48)
M/F	5/5	10/14	5/6	0/3	20/28
Age (years old)	4,5-38	1,5-9	4,5-21	4-17	1,5-38
The duration of disease (y)	1month-15,5	1,5-44	3 months -21	3,5-15,5	1 month -44
Etanercept	3	-	1	-	4
Аdalimumab	2	-	1	-	3
Tocilizumab	1	2	3	1	7
Сanakinumab	6	18	8	1	33
Аnakinra	1	8	-	1	10

### P114 The structural and functional changes of phospholipids in the biomembrains of lymphocytes and erythrocites for familial mediterranean fever and Henoch-Schonlein Purpura with children

#### H. Sargsyan, P. Ghazaryan

##### Republican Hematological Center, Yerevan, Armenia

###### **Correspondence:** H. Sargsyan

**Introduction:** According to some clinical feature of similarities (fever, abdominal pain, arthritis, skin manifestation, gastro-intestinal disorders and kidney diseases) Familial Mediterranean Fever (FMF) and Henoch-Schonlein Purpura (HSP) we attempt to identify membranes aspects of pathogeneses these diseases.

**Objectives:** The aim of our study is to identify some changes of individual phospholipids (PL), the activity of phospholipase A_2_, glycerol kinase and glycerophosphate dehydrogenase in lymphocytes and erythrocyte membranes and comparative analysis of FMF and HSP patients.

**Methods:** The examinations have carried out in 85 non complicated 7-14 ages of FMF patients and 32 healthy volunteers. Clinical studies have carried out in the National FMF Children Center, Center “Arabkir”. Biochemical changes have studied in Hematological Center. 85 patients in erythrocyte and lymphocytes membranes by the separate individual PL are studied: phosphatidylcholines (PCH), phosphatidylethanolamines (PE), phosphatidylinosites (PI), sphingomyelins (SPM), phosphatidic acids (PA), phosphatidylserine (PS), cytotoxic - LysoPCH (LPCH), and above mentioned enzymes changes. Previously it has been investigated the changes of indicators during the HSP diseases. The fractions of separate PL in erythrocyte and lymphocytes membranes are carried out by thin-layer chromatography methods and decay their enzymes of phospholipase A_2_, glycerol kinase and glycerophosphate dehydrogenase activity was determined by the microspectrophotometry method.

**Results:** Comparative studies are identified on the basis of membrane of erythrocyte and lymphocytes structural metabolism disorders, for the prevention and regulation some changes of FMF and HSP. Based on the results obtained by our research is an attempt to separate the membrane lipids metabolism disorder specific data which are responsible for affection of biomembranes.

**Conclusion:** According to our investigation we can conclude, that level of phospholipase A_2_ and citotoxic-LPCH are increase, at the same time, decreases the level of processes of phosphatidogeneses in mentioned diseases.

**Disclosure of Interest**

None declared

### P115 Assessment of clinical and genetic profile of greek patients with familial mediterranean fever using quantitative contemporary tools

#### V. Sgouropoulou, E. Farmaki, P. Pratsidou-Gertsi, M. Trachana

##### Pediatric Immunology and Rheumatology Referral Centre, 1st Dept of Pediatrics, Aristotle University, Hippocration Hospital, Thessaloniki, Greece, Aristotle University, Thessaloniki, Greece, Thessaloniki, Greece

###### **Correspondence:** V. Sgouropoulou

**Introduction:** Familial Mediterranean Fever (FMF) is the most common monogenic autoinflammatory disease, common in people of Mediterranean descent.

**Objectives:** To depict the clinical, laboratory and genetic profile in Greek patients with FMF, as well as to assess the severity, course, outcome of the disease using contemporary tools.

**Methods:** This is a single center, retrospective study including 94 young patients monitored at a paediatric rheumatology reference center in Northern Greece for a long time up to 30 years. Genotyping was performed with the conventional techniques PCR and NIRCA, disease severity was assessed at baseline with the ISSF, response to treatment with FMF50 at 6, 12 months post treatment initiation and at the end of follow-up and quality of life with GHQ-28 and SMILY-Illness questionnaires.

**Results:** The patients’ mean age at the disease onset was 3.49±3.04 years and the attack duration for 87.2% of the patients was 1-3 days. The clinical phenotype at the disease onset included fever (96.8%), abdominal pain (85.1%), arthralgia (54.3%), thoracic pain (45.7%), arthritis (21.3%), myalgia (20.2%), diarrhoea (19.1%), vomiting (19.1%), erysipelas-like rash (10.6%), lymphadenopathy (8.5%), splenomegaly (6.4%) and orchitis (2.1%). Regarding severity 36.2% had mild disease, 62.7% moderate and 1 patient had severe disease. The severity of the disease was significantly correlated with the M694V mutation (p=0.00), M694V/M694V homozygosity (p=0.001), rheumatic manifestations [arthritis (p=0.00)/arthralgia (p=0.002)], chest pain (p=0.00), erysipelas-like rash (p=0.001) and myalgia (p=0.00). No renal amyloidosis was observed in patients. All patients were treated with colchicine. Response to treatment was correlated with compliance to treatment (p=0.00). At 6 months post treatment onset an FMF50 score was observed in 67.02% of patients. At 12 months this score was achieved by 95.7%, by improving the patients’ compliance with experiential education, adjusting the colchicine dose, where needed and adding Canakinumab to the treatment of 3 patients. At the end of the patients΄ follow-up FMF50 score was achieved by 98.9%.The genetic profile of patients consisted of the following mutations: M694V (55.3%), M680I (29.8%), V726A (8.5%), E148Q (7.4%), M694I (3.2%), R761H, K695R and P369S (2.1% each). Molecular testing with NIRCA identified additionally R202Q (9.6%), E230K (2.1%), A761H and M694Q (1.1% each), that PCR does not include in its own panel. It was observed that homozygotes constituted 22.3% of the patients, heterozygotes 34%, compound heterozygotes 30.9%, while patients with complex genotype were 3.2%. Absence of mutation was observed in 9.6% of patients. The majority of patients showed a good quality of life. According to the GHQ-28 questionnaire, only 3 of the 78 patients were found to have some degree of psychological disorder, while the mean score of the SMILY-Illness questionnaire, which was 81.17 ± 11.93, proves the minor effect of the disease on patients' quality of life.

**Conclusion:** This study presented for the first time the long-term outcome of a large cohort of Greek pediatric patients and contributed in capture the national profile of the disease. FMF in Greece has a mild to moderate course. Compliance to treatment remains the cornerstone in taming disease activity, leading to a favorable outcome.

**Patient Consent Received**

No

**Disclosure of Interest**

None declared

### P116 Familial cold auto-inflammatory syndrome type 1 – first report from the Indian subcontinent!

#### R. Shanbhag Mohite, S. Bhattad

##### Pediatric Immunology and Rheumatology, Aster CMI hospital, Bangalore, India

###### **Correspondence:** R. Shanbhag Mohite

**Introduction:** Familial cold autoinflammatory syndrome (FCAS), is a rare inherited inflammatory disorder, caused by mutation in *NLRP3* gene which encodes a protein NLRP3.

**Objectives:** To describe a family with FCAS involving multiple members across 4-generations.

**Methods:** Retrospective review of clinical records was performed. A detailed clinical history including the age of presentation, symptoms, family history including details of affected family members, findings on physical examination, laboratory findings and details of treatment taken were recorded. Whole exome sequencing was performed on Next generation sequencing (NGS)platform.

**Results:** A 4-year-old boy, first born of non-consanguineous marriage, presented with skin rashes during the winter season from age of 2 years. The rashes were macular, red in color not associated with itching lasting for 12-24 hours. He complained of similar episodes on travelling to areas with low temperatures that resolved spontaneously. He had no history of any joint complaints or any other systemic involvement.

He had a strong family history involving at least 10 members across four generations of family members on the paternal side. All these family members reported rash during the winter season or on travel to areas with low temperature. The father had had macular rashes on the lower limbs and arms on exposure to low temperature during winter season followed by pain in ankles, knees, elbows, and small joints of fingers. Similar history was reported by the grandfather with rashes and joint pains during winters.

On examination, the boy was healthy looking, having multiple blanching macular rashes on his lower limbs on both flexor and extensor surfaces and on the soles of his feet. Systemic examination was normal. Inflammatory markers were slightly elevated, however immunological evaluation showed normal immunoglobulin levels.

In view of the strong family history, an autosomal dominant pattern of inheritance was suspected, and genetic evaluation was carried out. A pathogenic heterozygous mutation c.13222C>T (p.Ala441Val) was identified in exon 3 of the *NLRP3* gene by whole exome sequencing. Father was also noted to have the same mutation. A diagnosis of FCAS was established and they were started on colchicine for the persisting complaints and are under close follow-up.

FCAS is characterized by early onset fever, urticarial rashes, and joint pains after 1-3 hours of generalized cold exposure. Cold sensitivity is unique to FCAS among the autoinflammatory disorders and has been shown to increase the activity of the transcription factor AP-1, which increases synthesis of secretory proteins from inflammatory cells.

Mean age of presentation in a large series, was 47 days, with a range of 2 hours to 10 years. The most consistent finding in all affected subjects included recurrent fever and chills (93%), arthralgia (96%) and recurrent conjunctivitis (84%). Amyloidosis was reported only in 2% among those affected. In the index family, none of the family members developed amyloidosis, suggesting a milder phenotype.

Hoffman et al studied the benefit of the IL-1 receptor antagonists in patients with FCAS. Unfortunately, IL-1 blockers are not readily accessible in our subcontinent. There are case reports of thalidomide being used, however, prolonged exposure to thalidomide is known to cause peripheral neuropathy. We chose colchicine as it seems to be least toxic among the choices available. As majority of patients with FCAS seem to have a milder phenotype especially in tropical countries, it is often a clinical dilemma deciphering the choice and duration of therapy.

**Conclusion:** Cold induced urticarial rashes, arthritis and fever must make one think of FCAS especially in the setting of a positive family history. To the best of our knowledge, this is the first Indian family to be reported with FCAS type 1.

**Patient Consent Received**

Yes

**Disclosure of Interest**

None declared

### P117 Hypogammaglobulinemia and periodic fevers – think of TRNT1 deficiency!

#### R. Shanbhag Mohite, S. Bhattad

##### Pediatric Immunology and Rheumatology, Aster CMI hospital, Bangalore, India

###### **Correspondence:** R. Shanbhag Mohite

**Introduction:** Sideroblastic anemia associated with immunodeficiency, fevers and developmental delay (SIFD) is a newly described inborn error of immunity caused by TRNT1 deficiency. Not many cases have been described in literature.

**Objectives:** To describe a case of TRNT1 deficiency.

**Methods:** Retrospective review of clinical records was performed. A detailed clinical history including the age of presentation, symptoms, family history, findings on physical examination, laboratory findings and treatment details were recorded. Whole exome sequencing was performed on Next generation sequencing (NGS) platform.

**Results:** A 3-year-old boy born to a non-consanguineously married Indian couple, developmentally normal, presented with recurrent fevers from 6 months of age. Fever was intermittent, high grade; each episode lasting for 4-7 days and recurred 2-3 times a month occasionaly associated with loose stools and vomiting. He was also treated for pneumonia with intravenous antimicrobials twice in the past.

Family history was significant as the boy had lost two elder brothers within the first 2 years of life. Both had recurrent fevers from 6 months of age. While the first boy did not receive any blood transfusion, the second boy was transfused blood only while he was critically ill. No immunological or genetic studies had been performed in either of the children. Both parents and three daughters were healthy.

Systemic examination was normal. Immunological work up showed panhypogammaglobulinemia and low B cells. This in the setting of a family history suggestive of X-linked inheritance, a provisional diagnosis of X-linked agammaglobulinemia was made and he was started on monthly intravenous immunoglobulin (IVIG) transfusions. Despite which, he continued to have episodic periodic fevers and intermittent episodes of diarrhea, with no response to antibiotics. Inflammatory parameters were found to be elevated during the febrile episodes and they were normal while he was afebrile.

Genetic evaluation showed compound heterozygous mutation in *TRNT1* gene: exon 2 – c.143_144insTT and exon 7- c.1043A>T on whole exome sequencing. A diagnosis of SIFD was established and the family has been counselled for bone marrow transplant.

TRNT1 is an enzyme necessary for mitochondrial and cytosolic tRNA function. A deficiency of TRNT1 disrupts the protein synthesis machinery, leading to multi-organ involvement. The death of two boys in the index family, misled us to think on the lines of a possible X-linked disease. However, occurrence of periodic aseptic febrile episodes with no respite despite regular IVIG infusions, pointed towards an alternate diagnosis such as TRNT1 deficiency.

In a recent study on TRNT1 deficiency, hypogammaglobulinemia was detected in 7/9 with persistent B-cell lymphopenia in a few patients. Index case was noted to have panhypogammaglobulinemia and was treated with regular IVIG infusions. He, however, did not have severe anaemia warranting transfusions, retinitis pigmentosa (RP) or developmental delay, highlighting the heterogeneity of mitochondrial diseases.

Of late, TNF blockers have been effectively used to treat periodic fevers in SIFD. However, bone marrow transplant offers a more definitive therapy in these patients. Long term neurological outcome in transplanted patients remains undetermined as of now.

Sensorineural hearing loss, nephrocalcinosis, panniculitis, myofascitis, hemophagocytic lymphohistiocytosis, thromboembolism have been reported in a few cases of TRNT1 deficiency. Fortunately, index case has none of the above mentioned manifestations and is under close follow-up.

**Conclusion:** TRNT1 deficiency must be considered as a differential diagnosis in children presenting with periodic fevers and hypogammaglobulinemia. We describe a 3-year-old boy with periodic aseptic febrile episodes, hypogammaglobulinemia and low B-cells due to TRNT1 deficiency.

**Patient Consent Received**

Yes

**Disclosure of Interest**

None declared

### P118 Sjögren disease and Sjögren syndrome in children

#### O. Shpitonkova, N. Podchernyaeva, M. Osminina, V. Seraya, M. Nicolaeva, E. Afonina, J. Kostina

##### I.M.Sechenov First Moscow State Medical University, Moscow, Russian Federation

###### **Correspondence:** O. Shpitonkova

**Introduction:** Sjögren disease/syndrome (SD/SS) is uncommon in children. The standard clinical criteria used in diagnosis of adult Sjögren syndrome do not allow to make a diagnosis in children at an early stage. Xerostomia and xerophthalmia are the basic criteria of SD/SS more often revealed in late stage of the disease in children.

**Objectives:** To analyze and clarify the clinical and laboratory signs that indicates early stage of disease.

**Methods:** Analysis of 28 childhood cases SD/SS was done. All patients (pts) had satisfacted for disease criteria (**Sjogren’s International Collaborative Clinical Alliance = SICCA, 2012г).** All children carried out physical examination, salivary glands ultrasound investigation, sialometry, sialography, tests for xerophthalmia. Lab test included the measuring of CBC, total protein (TP), α-, β- and γ-globulins, IgA, IgM, IgG, rheumatoid factor (RF), ANA, ENA-profile with anti-SSA/Ro and anti-SSB/La detection.

**Results:** we observed 28 pts, aged from 3.5 to 17 years, the mean age 10.5 years, 24 girls and 4 boys (girls/boys = 7:1). SD was diagnosed in 13 girls. SS was diagnosed in 15 pts. Systemic lupus erythematosus (SLE) and SS – 11 pts ( 9 girls, 2 boys), systemic scleroderma (SSD) and SS – 2 girls, juvenile rheumatoid arthritis (JRA) and SS – 1 boy, juvenile dermatomyositis (JDM) and SS – 1 girl. Pts with SD (13) more often had mild course of the disease. Arthralgia, fatigue and low subfebrile temperature after acute respiratory infection was common complaints in all 13 pts (100%). Two girls had hemorrhagic nonspecific rush. Lab investigations revealed increased ESR in all pts (100%), leukopenia in 6 (46%) pts. Due to prolonged arthralgia, fatigue and subfebrile fever subsequent studies revealed hyper γ-globulinaemia (100%), ANA and RF in unexplained high titers (100%), and SSA/SSB antibodies (100%). Only two girls complained of dry mouth. And also two girls had episodes of recurrent parotitis. So the diagnosis was made on average 6-12 months after the onset by excluding other systemic diseases and by instrumental investigations. Salivary glands ultrasound investigation revealed initial stage of parotitis in all pts (100%) that was suggested by sialometry and sialography. As to xerophthalmia no one of patients complained of dry eyes. Low lacrimation was detected in 5 girls (38%). Low dose of prednisone and micophenolate mofetil (MF) have been used for treatment in all patients. In compare with SD course SS depend on associated rheumatic diseases and seems more aggressive. Strong fever up to 38C had all pts with SLE, one of with SSD and one pts with JRA. This two pts demonstrated high level of TP, significant hyper γ-globulinaemia with polyclonal secretion that required excluding lymphoma. Body weight loss had 6 children (40%). Generalized lymphoadenophaty had 8 pts (40%). Purpura and nonspecific rash registered in 9 (60%) children. Seven pts (46%) demonstrated Raynaud phenomenon. Six pts complained of dry mouth and 5 pts complained of dry eyes in disease onset. Twenty six pts (90%) with SS/SD demonstrated good response for immunosupressive treatment with reducing of clinical symptoms (fever, arthralgia, rush, function of salivary glands, lacrimation) after six month of treatment but not for lab parameters. Increased level of RF, ANA, anti-SSA/Ro and anti-SSB/La persisted in all patients for six month of follow up. In children with SS persistence of RF, ANA, anti-SSA/Ro and anti-SSB/La was strong despite more intensive immunosuppression. Two patients with SSD and JRA were not responsible for therapy.

**Conclusion:** SD/SS in children is characterized with late complain of xerophthalmia and xerostomia. Persistence of RF, ANA, anti-SSA/Ro and anti-SSB/La required the investigations for revealing of SD or SS.

**Patient Consent Received**

Yes

**Disclosure of Interest**

None declared

### P119 Recurrent attacks of fever and serositis associated with multiple polymorphisms: a case report

#### E. Baskın, M. Siddiqui, K. S. Gülleroğlu, A. Ç. Yılmaz, O. Söylemezoğlu

##### Department of Pediatrics, Başkent University, Ankara, Turkey

###### **Correspondence:** E. Baskın

**Introduction:** Hereditary periodic fever syndromes (HPF) are a group of autoinflammatory diseases characterized by recurrent attacks of fever and localized or systemic inflammatory manifestations, mainly in serosal surfaces, joints, and skin. HPFs are monogenic autoinflammatory disorder with autosomal dominant or recessive transmission. The well-known HPFs are Familial Mediterranean fever (gene MEFV), mevalonate kinase deficiency (gene MVK), TNF-receptor associated periodic fever syndrome (gene TNFRSF1A) and Cryopyrin-Associated Periodic Syndrome (gene NLRP3). Since the discovery of known HPFs genes, many variants, with clear or unclear pathogenic significance have also been reported. The possible manifestations of these variations are still largely unknown and maybe associated with atypical presentation in patients.

**Objectives:** Here we describe a 13-year-old patient with recurrent attacks of fever, pleural effusion, and chronic periodontitis, whose genetic workup for autoinflammatory diseases revealed polymorphism in genes coding for the inflammasome proteins.

**Methods:** Genetic workup for autoinflammatory diseases revealed NLRP3 (c.2113 C>A, p.Q705K) heterozygous polymorphism located in axon 5, SH3BP2 (c.1429 C>T, p.R477W) heterozygous polymorphism in exon 11 and MEFV (c.605 G>A), p.R202Q) heterozygous polymorphism in exon 2.

**Results:** A 13-year-old male patient, with a history of chronic periodontitis, was hospitalized with fever, chest pain, shortness of breath and fatigue. A computed tomography (CT) scan of the thorax revealed atelectasis at lower lobes of the left lung and left-sided pleural effusion. Two years later, recurrent symptoms with left sided pleural effusion led to the second and third hospitalisation of the patient. On the second attack, the MEFV genetic analysis revealed c. 605 G> A (p. R202Q) (p. Arg202Gln) heterozygous polymorphism, which has not been reported so far with such severe symptoms. However, colchicine 1.5mg/day was initiated. The patient was hospitalised again with recurrent symptoms on the third month of colchicine treatment. Genetic testing for the autoinflammatory diseases revealed polymorphism in genes coding for the inflammasome proteins. Clinical symptoms such as recurrent fever, myalgia, pleural effusion, and chronic periodontitis in our patient were atypical compared to the CAPS forms described at present. Therefore, canakinumab treatment was initiated. He remains symptom-free at one year on canakinumab and colchicine treatment.

**Conclusion:** The relationship between clinical heterogeneity and weak genotype phenotype in CAPS has been previously reported, and it has been shown that disease expression may be affected by other factors in addition to specific gene polymorphism. It is proposed that NLRP3 variants with unknown clinical significance may be associated with atypic inflammatory presentation. In our case, we presumed that the associations of NLRP3 Q705K polymorphism with R202Q and SH3BP2 polymorphisms have aggravated the clinical presentation and caused the resistant findings.

**Patient Consent Received**

Yes

**Disclosure of Interest**

None declared

### P120 Differentiation of phenotypes in patients carrying the Q705K mutation in the cryopyrin-associated periodic syndrome-data from the uper-aid registry

#### B. Sozeri^1^, K. Ulu^1^, T. Coskuner^1^, S. Caglayan^1^, S. Canbek^2^, F. Demir^1^

##### ^1^Pediatric Rheumatology; ^2^Medical Genetic, University of Health Sciences, Istanbul, Umraniye Training and Research Hospital, Istanbul, Turkey

###### **Correspondence:** B. Sozeri

**Introduction:** Cryopyrin-associated periodic syndrome (CAPS) is a hereditary autoinflammatory syndrome caused by mutations in NLRP3 (encoding cryopyrin), which presents with fever, fatigue and arthralgia and increased interleukin-1 (IL-1) secretion. There are three classical phenotypes observed: familiar cold autoinfammatory syndrome (FCAS), MuckleWells syndrome (MWS), and chronic infantile neurological, cutaneous articular syndrome (CINCA).

**Objectives:** We aim to describe NLRP3 gene mutations clinical presentation and relationship of phenotype and genotype.

**Methods:** The data of 320 patients from next generation sequencing genetic database of autoinflammatory diseases, Umraniye Training and Research Hospital, Department of Pediatric Rheumatology, were included in the study. Patients with VUS-likely pathogenic or pathogenic mutations in the NLRP3 gene were collected from these patients. In at least three months of follow-up, patients with autoinflammatory disease (AID) compatible recurrent episodes (with high acute phase response) were included, in which other etiological reasons (infectious, autoimmune, malignant diseases) were excluded. Demographic, clinical and laboratory data, treatments and responses of patients were presented

**Results:** 23 patients (16 male, 69.6%) were included in the study. The median age (IQR) at disease onset was 29 months ( 9 months – 5 years) and the median disease duration (IQR) was 33 months (14-40 months) . The mean number of febrile episodes was 11.2±7.3 per year and the mean duration of fever attacks was 4.45±2.50 days. The history of AID in family members was in 6 (26%) patients and consanguinity was in 3 patients. All patients were characterized by symptoms consistent with recurrent inflammatory syndrome. 52.2 % of patients (n= 12) presented with urticarial rash, 39% (n=9) with tonsillitis, 82.6% (n= 19) with arthralgia, 30.7 % (n=7) with conjunctivitis, 17.4 % (n=4) with headache. In 11/23 patients had worsening symptoms with cold contact. The phenotypes of PFAPA (n=5,21.7%), CAPS (FCAS and MWS) (n=11,47.8%) and undifferentiated AID (uAID) (n=7, 30.4%) were determined . There was no statistical significance between phenotypes in terms of median age at onset of attacks (>0.05). The most of patients (n=19, 82.6%) had Q705K variant in NRLP3 gene. Patients carrying the q705k variant had 26.3% PFAPA, 42% FCAS and 31.6% uAID phenotype. In 13 (59%) patients responded to colchicine treatment. Remained 9 (40.9%) patients had partial response or unresponsive to colchicine, were achieved to inactive disease with anti-IL 1 treatments.

**Conclusion:** We suggest that the Q705K variant causes autoinflammatory syndromes in a variety of phenotypes.

**Patient Consent Received**

Yes

**Disclosure of Interest**

None declared

### P121 Recurrent fever: an epidemic in a pandemic?

#### S. Torreggiani^1^, L. A. Baselli^1^, M. Gallizzi^2^, R. M. Dellepiane^1^, F. Minoia^1^

##### ^1^Fondazione IRCCS Ca' Granda Ospedale Maggiore Policlinico; ^2^University of Milan, Milano, Italy

###### **Correspondence:** S. Torreggiani

**Introduction:** Periodic Fever, Aphthous Stomatitis, Pharyngitis, Adenitis (PFAPA) Syndrome is the most common periodic fever syndrome in children. It is a benign self-limiting multifactorial disorder with an estimated incidence of 2.3/10000 children up to 5 years of age. Year 2020 was dramatically affected by the Covid-19 pandemic, with a yet undefined impact on several aspects of child health.

**Objectives:** To describe the demographic and clinical features of pediatric patients with recurrent febrile episodes referred to a single centre, with a focus on cases with onset in 2020.

**Methods:** Patients referred to the Pediatric Immunology and Pediatric Rheumatology departments between 01/01/2015 and 01/05/2021 for recurrent fever without an infectious cause were included in the study. Demographic, clinical and laboratory features were retrospectively reviewed and compared between patients with fever onset before and after January 1st, 2020. For the classification of PFAPA, both the modified Marshall criteria and the Eurofever/PRINTO criteria were used. Resolution of PFAPA was defined in presence of a 3-month fever-free interval. For statistical analysis, Mann-Whitney U test and Fisher’s exact test were used as appropriate.

**Results:** 84 patients were included; 33 were female, 82 were white. Median age at onset was 3.7 years (IQR 2-4.6, range 0.3-12.9). Median length of follow-up was 1.3 years (IQR 1.1-2.3, range 0.5-6.4 years). 57/84 (68%) patients met the modified Marshall criteria, 72/82 (88%) patients met the Eurofever/PRINTO criteria for PFAPA. Thirty-nine patients had disease onset between 01/2015 and 12/19, while 45 patients in 2020. One patient with onset in 2015 and resolution in 2018, restarted presenting recurrent fever in January 2020. In 29 patients recurrent fever resolved during the follow-up, 39 in the 2015-2019 cohort and 10 in the 2020 cohort. Median follow-up since onset was 2.4 years in the 2015-2019 group and 1.1 years in the 2020 group. While median age at onset was not significantly different in the two groups (3.2 vs 3.8 years), in patients with onset in 2020 median time to referral was shorter (14.3 vs 7.5 months, p<0.0001) and recurrent fever episodes tended to resolve faster (median 2.3 vs 0.9 years, p<0.0001). Aphtous stomatitis and lymphadenitis appeared more common in patients with onset before 2020 (64% vs 32%, p=0.0062 and 84% vs 58, p=0.0322 respectively). Duration of fever episodes and intercritic interval, periodicity, prevalence of pharyngitis, abdominal pain and arthralgia were not significantly different between the two cohorts.

**Table:** 

Onset	2015-2019 (n=39)	2020 (n=45)	***p***
Female	14/39 (35.9%)	19/45 (42%)	0.6556
Median age at onset (years)	3.2	3.8	0.5694
Median time to referral (months)	14.3	7.5	< 0.0001
Median time to resolution (years)	2.3 (n=19)	0.9 (n=10)	< 0.0001
Resolution within 1 year since onset*	1/39 (2.6%)	10/36 (27.8%)	0.0025
Aphtous stomatitis	23/36 (63.9%)	13/41 (31.7%)	0.0062
Lymphadenitis	26/31 (83.9%)	21/36 (58.3%)	0.0322
Modified Marshall Criteria	28/39 (71.8%)	29/45 (64.4%)	0.4933
Eurofever/PRINTO Criteria	33/37 (89.2%)	39/45 (86.7%)	1

* In patients with at least 1 year of follow-up

**Conclusion:** We observed an increase in referrals for recurrent fever with onset during 2020. Demographic and clinical features did not significantly differ between patients with onset in 2015-2019 and patients with onset in 2020, with the exception reduced occurrence of aphtous stomatitis and lymphadenitis, and faster referral and resolution for the latter. Whether an increase in parental attention and concern for fever episodes, or environmental factors, including SARS-CoV-2, contributed to the increase of referrals for recurrent fever still needs to be clarified.

**Disclosure of Interest**

None declared

### P122 Role of Interferon Signature (IS) in children with chronic non-bacterial osteomyelitis

#### S. Della Paolera^1^, C. Udina^2^, M. Klanjscek^2^, M. Giangreco^1^, B. M. Feldman^3^, R. M. Laxer^3^, N. Naraidoo^3^, T. Duong^3^, A. Tesser^1^, A. Taddio^1,2^

##### ^1^Institute for Maternal and Child Health IRCCS Burlo Garofolo; ^2^University of Trieste, Trieste, Italy; ^3^Hospital for Sick Children, Toronto, Canada

###### **Correspondence:** C. Udina

**Introduction:** Chronic Nonbacterial Osteomyelitis (CNO) is a non-infectious inflammatory disease characterized by uni- or multifocal bone lytic lesions that may cause local pain and swelling. CNO pathogenesis remains unknown, but many findings suggest that CNO might be an autoinflammatory disorder. It has been demonstrated that some rare variants in genes involved in chronic sterile bone inflammation are often reported in CNO patients; moreover, an increased type I interferon (IFN)-induced gene expression has been described in a subset of patients with autoinflammatory diseases.

**Objectives:** To assess the IFN signature in a cohort of children affected by CNO, and to evaluate its possible correlations with disease activity, disease severity, clinical manifestations, presence of comorbidities and different response to treatment.

**Methods:** We performed a multicenter cohort study involving two tertiary pediatric rheumatology units (The Hospital for Sick Children Hospital, Toronto, Canada and Institute of Maternal and Child Health “Burlo Garofolo”, Trieste, Italy). From May 1^st^ 2018 to May 31^st^ 2019 all patients aged <18 years with an established diagnosis of CNO were considered eligible for the study. Clinical, laboratory and radiological findings were collected into an anonymized electronic database (REDCap). An IFN score (measure of IFN signature intensity) >2 was considered positive. Informed consent was obtained from caregivers. Categorical variables are presented as absolute numbers and percentages; continuous variables are reported as median and interquartile range (IQR); *p*-values <0.05 were considered statistically significant.

**Results:** 37 patients aged <18 years with an established diagnosis of CNO were enrolled. Sixteen of 37 patients (43%) had a positive IFN score with a median value of 7.37 (IQR 3.2-11.3). Patients with a positive score did not differ in age at onset of disease, clinical or radiological features of disease, laboratory findings or response to treatment when compared to those with a negative score. The presence of complications was detected in 10 of 37 patients; 7 showed a positive IFN score (*p*=0.07). Analysis of continuous variables showed a higher IFN score in subjects with active disease, but this was not statistically significant (*p*=0.18) (Table 1).

**Table:** 

	Disease status	N	Min	25°pc	Median	75°pc	Max	Wilcoxon Mann Whitney P-value
**IFN score**	Active	23	0.45	0.89	2.07	8.43	23.65	0.18
	Inactive	14	0.53	0.80	1.07	3.52	12.89	

**Conclusion:** Nearly half of children with CNO enrolled in our study had a positive IS thus suggesting an IFN autoinflammatory pathway may be involved in a subset of patients with CNO. However, in our study, the IS did not distinguish between different disease phenotypes or response to treatment, or clinical course. Further studies with larger samples and with repeated measures of IFN score are warranted.

**Patient Consent Received**

Yes

**Disclosure of Interest**

None declared

### P123 Chronic nonbacterial osteomyelitis: clinical characteristics, therapy, and outcome in Turkish pediatric patients

#### K. Ulu^1^, S. G. Karadağ^2^, E. Bağlan^3^, G. Kavrul Kayaalp^4^, G. Otar Yener^5^, M. Çakan^6^, K. Öztürk^7^, H. E. Sönmez^8^, S. Özdel^3^, F. Demir^1^, N. Aktay Ayaz^4^, B. Sözeri^1^ on behalf of PeRA-Research Group

##### ^1^Pediatric Rheumatology, University of Health Sciences, Ümraniye Research and Training Hospital, Istanbul; ^2^Pediatric Rheumatology, Erzurum Regional Research and Training Hospital, Erzurum; ^3^Pediatric Rheumatology, University of Health Sciences, Sami Ulus Maternity and Children's Diseases Training and Research Hospital, Ankara; ^4^Pediatric Rheumatology, Istanbul University, Faculty of Medicine, Istanbul; ^5^Pediatric Rheumatology, Şanlıurfa Research and Training Hospital, Şanlıurfa; ^6^Pediatric Rheumatology, University of Health Sciences, Zeynep Kamil Maternity and Children's Diseases Training and Research Hospital; ^7^Pediatric Rheumatology, Istanbul Medeniyet University, Göztepe Prof. Dr. Süleyman Yalçın City Hospital; ^8^Pediatric Rheumatology, Kocaeli University, Faculty of Medicine, Istanbul, Turkey

###### **Correspondence:** K. Ulu

**Introduction:** Chronic non-bacterial osteomyelitis (CNO) and its severe form chronic recurrent multifocal osteomyelitis (CRMO) are considered as rare autoinflammatory diseases. It is a heterogeneous condition and is characterized by recurrent attacks of localized bone pain, swelling or loss of function in any area, mainly affecting the metaphysis of the long bones, clavicles, vertebrae, and pelvis. The pathophysiology of the disease is not fully understood, but it has been shown that there is unbalanced cytokine expression and increased inflammatory activation in patients' monocytes, a pro-inflammatory response contributing to osteitis.

**Objectives:** The aim of this study was to describe the characteristics and outcome of patients with CNO.

**Methods:** We retrospectively reviewed clinical, pathological, and radiological data of children with CNO at 8 pediatric rheumatology centers from Turkey.

**Results:** Sixty-seven patients were assessed (31 females and 36 males) with a median follow-up time of 20 months [min.-max. (3-70 mo.)]. The median age at diagnosis and median time of diagnostic delay were 12 years (3-17,4 yrs.) and 12 months (1-96 mo.), respectively. Arthralgia in various joints, especially in the ankle, and bone pain were the most common presenting symptoms (58.2%). Peripheral arthritis was detected in 30 patients (44.7%), isolated sacroiliitis in 12 patients (17.9%) and both type of involvement in 5 patients (7.4%).

The distribution of the lesions was examined by MRI (local or whole body) at the time of diagnosis and throughout the course of the disease: metaphysis of the long bones, especially lower extremities were affected most commonly (76.1%). Vertebrae and clavicles were affected in 19.4% and 16.4% of the patients, respectively. Biopsy was performed in 26 patients (38.8%). The disease course was unifocal non-recurrent in 8 patients (11.9%), unifocal recurrent in 4 patients (6%), multifocal non-recurrent in 37 patients (55.2%), and multifocal recurrent (CRMO) in 18 patients (26.9%). Patients with vertebral involvement had multifocal disease generally. All patients enrolled in the study received non-steroidal anti-inflammatory drugs (NSAIDs) as initial therapy, of which thirty (44.8%) were partially responsive, twenty-five (37.3%) were unresponsive, and eleven (16.4%) were in remission. Disease-modifying antirheumatic drugs (methotrexate or salazopyrin) were used in 56 patients (83.5%). Biological therapy was required in 22 patients (32.8%) with a median duration of 18 months. Among these patients, 5 (7.4%) had a flare under biologic therapy. Also four patients (5.9%) received bisphosphonate therapy. At the last visit evaluation, active disease findings were present in 8 patients (11.9%).

**Conclusion:** This large multicentric cohort gives a detailed evaluation of clinical presentation, extent of involvement, therapeutic approaches and outcomes of children with the diagnosis of CNO. Most of the patients had multifocal involvement and a third of them had a relapsing course. NSAIDs were not able to control disease activity alone but disease activity was controlled in most of the patients by addition of methotrexate/salazopyrin or a biologic agent.

**Patient Consent Received**

Yes

**Disclosure of Interest**

None declared

### P124 Severe pulmonary fibrosis as the first manifestation of Sting Associated Vasculitis of Infancy (SAVI)

#### O. Vougiouka, S. Karamichalou, S. Oikonomou, G. Kolitsida, M. Tsolia

##### 2nd Department of Pediatrics, ‘P&A Kyriakou’ Children’s Hospital, National and Kapodistrian University of Athens, Athens, Greece

###### **Correspondence:** O. Vougiouka

**Introduction:** SAVI is a rare autosomal or de novo heterozygous genetic disorder, classified under the newly discovered type I interferonopathies [1]. We present a case of a child with SAVI, without typical cutaneous vasculopathy and genotype.

**Objectives:** The presentation of a 14 month old girl, born to a consanguineous couple of Syrian descent, with multiple episodes of aggressive cough, tachypnea and failure to thrive, since the age of 6 months.

**Methods:** Whole exon sequencing was ordered and a novel homozygous mutation in the STING gene [(c.10822 C>T (p.Arg281Trp)] was confirmed, resulting in constitutive activation of STING protein. In addition, a strong interferon type I signature had been found in peripheral blood.

**Results:** The child attended the emergency department with difficulty in breathing (respiratory rate > 60/min, Paco2= 55mmHg), and low fever. Radiograph of lung demonstrated diffuse lung opacities and initial impression was of bacterial infection, so antibiotics were administered, without improvement. During her hospitalization, she entered a state of complete oxygen dependency, with increasing needs of high flow oxygen supply and occasionally fever spikes accompanied by elevation of acute inflammatory indexes: WBC (max 29.000/μl), CRP (130mg/L), ESR (51mmHg), Ferritin (360ng/ml) and SAA (30,7mg/L). While none of pathogen was identified by laboratory investigation, administration of corticosteroids was attempted with good treatment response (1-2 mg /kg/d). CT lung scan revealed diffuse lung disease and whole exon sequencing confirmed the cause. Until now, the identified mutation has been described in 8 children, all of whom of Arabic ethnicity [2]. Treatment with baricitinib, a JAK-1, 2 inhibitor, was commenced in increasing dose with impressive response (max dose: 10mg per day). As a result the deterioration of the disease stopped and at the same time the status of our patient improved: needs of oxygen supply decreased to 2 liters normal flow, oral steroids tampered (0,2mg/kg/d) and the respiratory status improved.

**Conclusion:** Interferonopathies are the newly added subject of current inflammatory syndromes uncovering decisive steps of etiological pathways and treatments [1]. One of them is SAVI, recognized in 2014. SAVI is formally presented as peripheral necrotizing vasculitis on which lung fibrosis and end stage respiratory failure develops [3]. There are a few cases in literature with initial lung involvement and vasculitis development under environmental circumstances [4]. Our patient never developed any kind of skin involvement, even if she had past exposure to cold. Diagnosis was challenging. Early diagnosis and treatment are of vital importance in order to avoid progression to pulmonary failure, which is the first cause of death in SAVI [3]. Clinical suspicion of SAVI among infants, presented with severe, intractable respiratory distress, is of prime importance in order to diagnose the disease, using whole exon sequencing test examination. ‘*Chance favors the prepared mind’*, *Louis Pasteur*.

REFERENCES

1. J. Munoz et al. Interferonopathies de type I, Elsevier Masson 2015, 142 (653-663)

2. M. A. Alghmadi et al, A Novel biallelic STING 1 Gene Variant Causing SAVI in two siblings, Frontier in immunology, Jan 2021, vol 11, article 599564.

3. F. Staels et al, Adult –onset ANCA – associated vasculitis in SAVI: Extension of the phenotypic spectrum, case report and review of literature, Frontier in Immunology, Sept 2020, vol 11, article 575219.

4. S. Cazzato et al. Lung involvement in monogenic interferonopathies, Eur Respir Rev 2020; 29: 200001.

**Patient Consent Received**

No

**Disclosure of Interest**

None declared

### P125 Phenotypic overlap of (autoinflammation and) PLCG2-associated antibody deficiency and immune dysregulation: case series and literature search

#### T. Welzel^1,2^, L. Oefelein^1^, U. Holzer^3^, J. B. Kuemmerle-Deschner^1^

##### ^1^Pediatric Rheumatology and Autoinflammation Reference Center Tuebingen (arcT), University Children`s Hospital Tuebingen, Tuebingen, Germany; ^2^Pediatric Pharmacology and Pharmacometrics, University Children`s Hospital Basel (UKBB), University of Basel, Basel, Switzerland; ^3^Pediatric Hematology and Oncology, University Children`s Hospital Tuebingen, Tuebingen, Germany

###### **Correspondence:** T. Welzel

**Introduction:** Mutations in the *phospholipase C gamma 2* (*PLCG2*) gene are typically associated with a spectrum of diseases ranging from allergy and immunodeficiency to autoimmunity and autoinflammation [1]. *PLCG2* gene variants can cause the PLCG2-associated antibody deficiency and immune dysregulation (PLAID) and the autoinflammation and PLCG2-associated antibody deficiency and immune dysregulation (APLAID) syndrome. In recent years, awareness for monogenic syndromes with concomitant symptoms of autoinflammation and immunodeficiency has expanded. However, making a diagnosis is still challenging.

**Objectives:** To elaborate phenotype and genotype characteristics of five patients with suggestive history for autoinflammation and immunodeficiency.

**Methods:** This is a single center case series of five patients (P). P1 is a 44-year-old mother of 14 (P2) and 11 year (P3) old boys and twins (male/female) aged 4 years (P4, P5). They presented with recurrent fevers, episodes of conjunctivitis, lymphadenopathy, headaches, myalgia, abdominal pain, cold induced urticaria and upper airway infections. In P1 symptoms appeared in early adulthood. P2-P5 are affected since infancy. Family history was unremarkable. Parents were not consanguineous.

Work-up included physical and laboratory examinations (genetic panel test, flow cytometry for lymphocyte subsets, expression of Interleukin (IL-)2, IL-4, IL-17, IFNγ, and CFSE proliferation of T cells. High-frequency pure tone audiometry (HF-PTA), ophthalmology examination and skin biopsy were performed.

**Results:** Examination confirmed conjunctivitis (P1–5) and mild hearing loss (P1, P2). Skin biopsy in P1 indicated urticaria. Serum amyloid A and S100A8/A9 were slightly elevated during flares. Unswitched B-cells were decreased. Naive IgD^+^CD27^-^ B-cells and unswitched IgD^+^CD27^+^ B-cells were decreased, switched IgD^-^CD27^+^ B-cells were slightly increased (P1-5). T-cell function was normal. Genetic testing revealed a new heterozygous missense variant (c.77C>T, p.Thr26Met) in the *PLCG2*.

A literature search in Pubmed and Infevers yielded reports on PLAID patients with cold-induced urticaria, recurrent infections, signs of autoimmunity, allergic diseases and granulomatous dermatitis along with immunologic findings (reduced immunoglobulins (Igs), low circulating class switched memory B cells, reduced NK cells). In APLAID patients, rash, granuloma, cutis laxa, eye inflammation, recurrent infections, abdominal pain/inflammatory bowel disease, musculoskeletal complaints and immunologic findings (reduced/normal Igs, reduced circulating class-switched CD27^+^ memory B-cells, decreased/normal NK-cells) were reported. P1-5 showed similarities to both, PLAID (cold-induced urticaria) and APLAID (eye inflammation, musculoskeletal complaints, no circulating antibodies). In addition, they displayed PLAID and APLAID symptoms (recurrent infections, abdominal pain/diarrhea, normal T-cell function). Sensorineural hearing loss (P1, P2) was reported in one APLAID patient [2].

**Conclusion:** This case series describes a new heterozygous missense *PLCG2* variant (c.77C>T, p.Thr26Met) which might cause a phenotypical overlap of PLAID and APLAID disease patterns.

**References**

[1] Giannelou A, et al. Curr Opin Allergy Clin Immunol. 2014;14:491-500.

[2] Neves JF, et al. Front Immunol. 2018;9:2863.

**Patient Consent Received**

Yes

**Disclosure of Interest**

T. Welzel: None declared, L. Oefelein: None declared, U. Holzer: None declared, J. Kuemmerle-Deschner Consultant for: SOBI, Novartis

### P126 Colchicine effectiveness in periodic fever, aphthous stomatitis, pharyngitis and adenitis (PFAPA)

#### T. Welzel^1,2^, M. Ellinghaus^1^, A. L. Wildermuth^1^, N. Deschner^3^, S. M. Benseler^4^, J. B. Kuemmerle-Deschner^1^

##### ^1^Pediatric Rheumatology and Autoinflammatory Reference Center, University Children`s Hospital Tuebingen, University of Tuebingen, Tuebingen, Germany; ^2^Pediatric Pharmacology and Pharmacometrics/ Pediatric Rheumatology, University Children`s Hospital Basel (UKBB), University of Basel, Basel, Switzerland; ^3^Department of Anaesthesiology and Intensive Care Medicine, University Hospital Tuebingen, University of Tuebingen, Tuebingen, Germany; ^4^Rheumatology, Department of Paediatrics, Alberta Children`s Hospital, Cumming School of Medicine, Alberta Children’s Hospital Research Institute, University of Calgary, Alberta, Canada

###### **Correspondence:** T. Welzel

**Introduction:** Periodic fever, aphthous stomatitis, pharyngitis, and cervical adenitis (PFAPA) syndrome is the most common recurrent fever syndrome in children. PFAPA has often a good prognosis, but disease activity dramatically impacts health-related quality of life, family and psychological wellbeing. Thus, disease control is imminent. Colchicine may be effective in reduction of disease activity in PFAPA.

**Objectives:** To evaluate the effectiveness of colchicine for PFAPA in children.

**Methods:** A cohort study of consecutive children diagnosed with PFAPA treated with colchicine was performed between 03/2012 and 12/2020. Patients were excluded if (i) genetic AID testing revealed any gene variant, (ii) they had elevated liver enzymes or decreased kidney function. Demographics, clinical features, inflammatory parameters and PGA/PPGA were collected. Disease activity was defined as patient/parent (PPGA) and physician (PGA) global assessment recorded on a 10 cm visual analogue scale, with 0 representing no and 10 maximal disease activity. PPGA/PGA was captured at baseline (colchicine start) and at follow-up. Primary outcome was colchicine effectiveness defined as improvement of disease activity (PPGA or PGA ≥2).

**Results:** A total of 30 PFAPA patients were included, 50% were female, median age was 5.2 years (1- 10.75). At baseline, the median PPGA was 4 (0 – 9) and median PGA was 3.5 (0 – 6). All patients had fever (mean 40.08 °C, ± 0.58°C). Median flare frequency was 4 weeks (1 – 6); flares lasted 3 to 6 days. Lymphadenopathy (100%), pharyngitis (97%) and aphthous stomatitis (47%) were commonly reported. During flares, serum amyloid A (mean 423 mg/l, SD ± 327) and C-reactive protein (mean 5.05 mg/dl, SD ± 4.8) were elevated. In the past, 11 patients (37%) were treated with corticosteroids (CS). CS reduced flare intensity in all, but increased frequency in 9 patients (82%). Median follow-up time was 3.9 months (2 – 10.6). Primary Outcome: colchicine was effective in 19 patients (63%). Of these, 13 patients (43%) achieved a PPGA reduction ≥2 and 14 patients (47%) a PGA improvement ≥2. No PPGA or PGA changes were seen in 4 and 3 patients, respectively. Secondary Outcome: Children with CS mediated increased flare frequency experienced on colchicine PGA (100%) and PPGA (55%) improvement. At follow-up, a total of 17 patients (57%) experienced any PPGA improvement and 25 (83%) had any PGA improvement. Median PPGA and PGA decreased to 2 (0 – 8 and 0 – 4, respectively).

**Conclusion:** Colchicine was effective in controlling disease activity in PFAPA patients. More than 60% of PFAPA patients had a PPGA or PGA improvement ≥2. In patients with CS mediated increased flare frequency colchicine effectively reduced disease activity. Taken together, colchicine is an effective treatment in PFAPA and a potent alternative to CS, particularly in PFAPA patients with CS mediated increased flare frequency.

Susanne M. Benseler and Jasmin B Kuemmerle-Deschner have contributed equally to this work and should be therefore be considered as co-senior authors.

**Disclosure of Interest**

T. Welzel: None declared, M. Ellinghaus: None declared, A. Wildermuth: None declared, N. Deschner: None declared, S. Benseler: None declared, J. Kuemmerle-Deschner Consultant for: SOBI, Novartis

### P127 The development and preliminary validation of a scoring tool for monitoring disease activity in patients with iga vasculitis (HSP)

#### C. E. C. Williams^1^, J. Murphy^2^, T. Dowsett^3^, L. Oni^1^

##### ^1^Department of Women’s and Children’s Health, Institute of Life Course and Medical Sciences; ^2^Royal Liverpool and Broadgreen University Hospitals; ^3^Department of Paediatric Nephrology, Alder Hey Children’s NHS Foundation Trust Hospital, Liverpool, United Kingdom

###### **Correspondence:** C. E. C. Williams

**Introduction:** IgA vasculitis (IgAV, Henoch-Schoenlein purpura, HSP) is the most common form of childhood vasculitis. The Paediatric Vasculitis Activity Score (PVAS) is an objective tool used for scoring all types of vasculitis in clinical trials and it includes 64 manifestations of various active vasculitides. However, the inclusion of some subsystems such as chest, cardiovascular and ENT suggest it may not be specific enough for evaluating IgAV disease activity.

**Objectives:** The aim of this study was to develop and perform preliminarily validation of a vasculitis activity scoring tool designed specifically for IgAV (the IgA-VAS) and compare performance to the PVAS.

**Methods:** A cohort of children with IgAV were retrospectively scored in February 2021 using both the IgA-VAS and the PVAS. Test validity, concurrent validity and inter-rater agreement were assessed. A randomly selected subgroup were also scored using a physician visual analogue scale as a marker of global disease activity. Any domains which scored 0 for all patients were excluded from the analysis.

**Results:** The IgA-VAS consists of 40 manifestations, each with a score from 0-10, divided into 5 domains: cutaneous, gastrointestinal, musculoskeletal, renal and other. For preliminary validation, retrospective scoring was performed in a single tertiary centre over a 5-year period. 196 children were identified; 153 met inclusion criteria. 54% were male with a median age of 5.7 years (range 0.6-16.7). Median total scores for the IgA-VAS were 7/125 (range 2-31) and 5/125 (range 2-29) for rater 1 and rater 2 respectively. Median PVAS scores were 6/63 (range 2-25) and 5/63 (range 2-20). Correlation between all overlapping domains of the two tools was strong (all *r*>0.5,*p*<0.001). Inter-rater reliability overall was low for both tools (0.131 and 0.225, *p*<0.001). For the IgA-VAS, inter-rater reliability was low for the cutaneous, renal and other domains (0.332, 0.237, 0.288 *p*<0.001) and high for the gastrointestinal and musculoskeletal domains (0.543 and 0.667, *p*<0.001). The general, cutaneous and renal subsystems in the PVAS had a low inter-rater reliability (0.347, 0.213, 0.304, *p*<0.001) and was better for the abdominal domain (0.579, *p*<0.001). The IgA-VAS moderately correlated with the visual analogue scale for both raters (*r*=0.482, *r*=0.362, *p*<0.05), however the PVAS strongly correlated with rater 1 (*r*=0.504, *p*=0.004) and moderately correlated with rater 2 (*r*=0.372, *p*=0.043).

**Conclusion:** The IgA-VAS has improved since its initial circulation however further work is needed to optimise the tool before prospective validation.

**Disclosure of Interest**

None declared

### P128 Temporary colchicine treatment in children with heterozygous familial mediterranean fever - an analysis of aid-registry and JIRcohorte

#### C. Vinit^1^, V. Hentgen^1^, A. L. Hitzegrad^2^, J. Klotsche^3^, E. Lainka^4^, T. Niehues^5^, U. Neudorf^4^, I. Kone-Paut^6^, C. Hinze^7^, E. M. Michalski^7^, S. Fuehner^7^, M. Hofer^8^, D. Foell^7^, T. Kallinich^2,9^, H. Wittkowski^7^

##### ^1^Department of General Pediatrics, French reference center for autoinflammatory diseases (CEREMAIA), Versailles Hospital, Versailles, France; ^2^Pediatric Pneumology and Immunology, Charité University Medicine; ^3^Department of Epidemiology, German Rheumatism Research Centre Berlin, Berlin; ^4^Pediatric Rheumatology, University Children`s Hospital, Essen; ^5^Center of Pediatrics and Youth Medicine, Helios Klinikum Krefeld, Krefeld, Germany; ^6^Pediatric Rheumatology and CEREMAIA, Bicêtre Hospital APHP, University of Paris Sud Saclay, Le Kremlin-Bicêtre, Paris, France; ^7^Pediatric Rheumatology and Immunology, UNIVERSITY HOSPITAL MUENSTER, Muenster, Germany; ^8^Pediatric Rheumatology Unit of Western Switzerland, Lausanne University Hospital (CHUV), Lausanne, Switzerland; ^9^German Rheumatism Research Centre Berlin, Leibniz Institute, Berlin, Germany

###### **Correspondence:** H. Wittkowski

**Introduction:** Familial Mediterranean Fever (FMF) is a prototypic autoinflammatory disorder associated with MEFV pyrin-encoding gene mutations, characterized by unprovoked episodes of inflammation. In a substantial number of patients with familial Mediterranean fever (FMF) only one mutation within the MEFV gene (MEditerranean FeVer gene) can be found.

**Objectives:** To analyse whether colchicine can be terminated in a subgroup of these patients without reoccurrence of symptoms and/or inflammation.

**Methods:** All FMF-patients registered in the German AID-registry (Autoinflammatory Disease registry) and the international Juvenile Inflammatory Rheumatism cohort (JIRcohort) with a heterozygous MEFV-genotype and confirmed clinical FMF diagnosis, that stopped colchicine treatment during follow-up, where enrolled. Duration of colchicine withdrawal, reasons of re-introduction, and epidemiologic characteristics of the patients successfully terminating colchicine in comparison to patients re-introducing the medication where analysed.

**Results:** We identified 169 heterozygous pediatric FMF patients, in 44 of them colchicine therapy was discontinued. Among those, 27 stayed in colchicine-free remission during a follow-up of 2,04 ± 1,55 years after discontinuation. Compared to heterozygous FMF patients continuously taking colchicine (n=125) and patients who had to resume colchicine after temporary discontinuation (n=17), these patients were treated with lower initial colchicine dosages, 0,7 ± 0,32 mg/day vs 0,77 ± 0,38 mg/day (not significant). They were older at disease onset (4,84 ± 3,15 years vs 4,04 ±3,86 years) and in tendency they had lower initial CRP-levels during subclinical disease (15,68 ± 19, 56 mg/L vs. 19,07 ± 27,23 mg/L).

**Conclusion:** This study underlines previous observations of a non-chronic phenotype of heterozygous FMF, which follows a rather mild clinical course, may respond to lower colchicine dosages to control the disease and may require therapy only temporarily. Further prospective and long-term observations are warranted before formal recommendations for colchicine termination can be drawn.

**Patient Consent Received**

No

**Disclosure of Interest**

None declared

### P129 Evaluation of the medical conditions of first-degree relatives of patients with familial mediterranean fever

#### S. Yildirim, F. Haslak, M. Yıldız, A. Aliyeva, O. Koker, A. Adrovic, S. Sahin, K. Barut, O. Kasapcopur

##### Department of Pediatric Rheumatology, Istanbul University-Cerrahpasa, Istanbul, Turkey

###### **Correspondence:** M. Yıldız

**Introduction:** Familial Mediterranean fever (FMF) is the most common monogenic autoinflammatory disease. It was recently shown that the most common additional two diseases were juvenile idiopathic arthritis (JIA) and immunoglobulin (Ig) A vasculitis in children with FMF. Furthermore, it was demonstrated in the studies involving all age groups that the frequencies of spondyloarthropathies, Behçet disease, Sjögren disease, polyarteritis nodosa (PAN), inflammatory bowel diseases, multiple sclerosis (MS), and psoriasis were increased in patients with FMF.

**Objectives:** Given the strong genetic background of FMF, the diseases that have been previously demonstrated as co-exists in children with FMF should also be investigated in the other family members. Therefore, we aimed to examine the diseases of first-degree relatives (FDRs) of our pediatric patients with FMF in the present study.

**Methods:** In total, 449 patients with FMF and 147 patients with JIA who are being followed up at Istanbul University-Cerrahpasa Department of Pediatric Rheumatology and 93 healthy controls were interviewed between March 2019- November 2019 during routine outpatient visits. The medical conditions of their FDRs were asked. Among the FDRs of index cases, those with FMF were excluded from the study.

**Results:** The mean age of healthy children (n=93), patients with FMF (n=449), and patients with JIA (n=147) were 7.7 ± 4.6 years, 12.6 ± 4.8 years, and 11.6 ± 5.2 years, respectively. A total of 3071 FDRs (FMF:1975, JIA: 690, Healthy Children: 406) were included in the study. While the most common medical conditions reported among the FDRs of the patients with FMF were asthma (n=90, 4.5%), tonsillectomy history (n=66, 3.3%) and type 2 diabetes (n=59, 2.98%), the most common medical conditions detected among the FDRs of the patients with JIA were type 2 diabetes (n=17, 2.4%), asthma (n=15, 2.1%) and tonsillectomy history (n=13, 1.8%). Among the FDRs of the healthy children, asthma (n=15, 3.69%), tonsillectomy history (n=12, 2.95%), and type 2 diabetes (n=6, 1.47%) were the most commonly detected ones. The frequencies of acute rheumatic fever (ARF), asthma, allergic rhinitis, and appendectomy history were significantly higher among the FDRs of the patients with FMF compared to other FDRs (all <0.05).

**Conclusion:** This is the first study evaluating the FDRs of patients with FMF. ARF, asthma, allergic rhinitis, and appendectomy history were found to be significantly more frequent in FDRs of the patients with FMF compared to the FDRs of healthy children and the patients with JIA.

**Patient Consent Received**

Yes

**Disclosure of Interest**

None declared

### P130 A Very precocious inflammatory colitis: a case of A20 haploinsufficiency (HA20)

#### F. Biscaro, L. Zanatta, V. Piazza, S. Martelossi

##### Pediatric, Ca' Foncello Hospital, Treviso, Italy

###### **Correspondence:** L. Zanatta

**Introduction:** We describe a case of non-specific colitis as the first manifestation of an autoinflammatory disease.

**Objectives:** A., 12 months old, comes to our attention for severe normocytic anemia, without signs of hemodynamic failure. From 3 months of age, A. had a history of bloody diarrhea during exclusive maternal breast-feeding. This condition was diagnosed as allergic proctitis and maternal diet free of milk and derivatives was started. The weaning was conducted in a diet free from milk too. Despite a reduction in bloody diarrhea complete resolution of the symptom never occurred. At admission to our Center, the patient performed a colonoscopy, which revealed the presence of small, eroded translucent nodules and bleeding mucosa suggestive of nodular lymphoid hyperplasia throughout the colon. Histological examination showed active colitis without signs of colitis IBD or allergic colitis. After discharge, we began oral steroid therapy and gastroenterological follow-up. Two weeks later, during steroid withdrawal, A. came to Emergency Room for acute panniculitis of the limbs for which we prescribed an anti-inflammatory therapy. After 48 h the skin condition worsened and she was febrile and suffering. Therefore, A. was hospitalized for further investigations. Blood tests showed a rise in inflammation indexes. Given the inflammatory status, intravenous steroid therapy was started with rapid response. At each attempt to stop steroid treatment, the same symptoms recurred. We also noted the presence of adverse effects related to therapy (Cushing-like aspect and irritability).

**Methods:** In differential diagnosis, the following hypotheses were evaluated and relative tests were performed. 1) Haematological disease: bone marrow aspirate negative for neoplastic conditions. 2) Infectious disease: swabs for viruses and bacteria, as well as serological tests all negative. No history of COVID19 or contact with COVID19 patients. 3)Immunological/inflammatory disease: family history of autoimmune conditions (father with recurrent oral aphthosis, vitiligo and thyroiditis) and unkown diseases with early-onset (paternal grandfather with liver disease started when he was young, brother of paternal grandfather died at 16 years old due to unexplained causes). Therefore, first level autoimmune tests were done, resulted all normal. Then the possibility of early-onset chronic inflammatory bowel disease (VEO-IBD) were evaluated but genetics was negative. Finally, since autoinflammatory genesis was suspected, interferon signature and molecular investigations of the main known genes responsible for auto-inflammatory diseases were performed. These tests revealed mutation in TNFAIP3, leading to the diagnosis of haploinsufficiency A20 (HA20). In view of this diagnosis, therapy with a biological drug, IL1 receptor antagonist (Anakinra), was started.

**Results:** HA20 is an autoinflammatory disease due to a loss of function of A20 protein, whose role is to down-regulate the pro-inflammatory pathway of NF-kb. It is autosomal dominant, with early-onset and with very heterogeneous manifestations even within the same family. This condition is often called Behcet-like, with which it shares some features (oral ulcers, gastrointestinal and skin involvement). Regarded A., the disease appeared at 3 months of age with ulcerative colitis and later skin manifestations started. Probably father and paternal grandfather have the same condition but with milder manifestations.

**Conclusion:** This case taught us that HA20 should be considered in patients with very early-onset inflammatory disease, characterized by colitis as the first manifestation and cutaneous involvement, often with positive family history.

**Patient Consent Received**

Yes

**Disclosure of Interest**

None declared

## e-Poster viewing: Uveitis

### P131 Challenging a case of intermediate uveitis in a young female

#### A. M. A. Abushhaiwia^1^, Y. AElfawires^1^, A. Ateeq^2^, A. A. Abdullah^3^, A. Nuwayji^2^, A. M. Kouklah^2^

##### ^1^Paediatric Rheumatology, Tripoli Children's Hospital, Faculty of Medicine, Tripoli university; ^2^Paediatric Rheumatology; ^3^Paediatric Rheumatology, Tripoli Children's Hospital, Tripoli, Libya

###### **Correspondence:** A. M. A. Abushhaiwia

**Introduction:** Intermediate uveitis (IU) is described as inflammation in the anterior vitreous, ciliary body and the peripheral retina. The diagnostic term pars planitis should be used only for that subset of IU where there is snow bank or snowball formation occurring in the absence of an associated infection or systemic disease (that is, “idiopathic”). It is usually bilateral and is usually asymmetric in severity. Pars planitis is a chronic condition that may reoccur for many years. Hereby, we are presenting one rare case report of a 13-year Libyan girl who was diagnosed with intermediate uveitis

**Objectives:** illustrating importance of not considering all types of uveitis as pars planitis, and the need to rule out sarcoidosis, multiple sclerosis and Lyme disease as possible causes.

**Methods:** case report study

**Results:** A 13-year-old Libyan, female presenting to our Rheumatology clinic, Dec/2020 with blurred vision and floating objects for one month involving her Lt > than Rt eye, not associated pain nor irritation, with history of previous episodes of bilateral uveitis in 2017 and 2018. Managed with topical steroids and mydriatics under ophthalmology care. There was no history of trauma, headaches, tick bites, fever, rash, joint pain or swelling, cough, respiratory distress, oral ulcer, no weakness, numbness, or clinical features suggestive of enthesitis or other organ system involvement. There was significant family history of autoimmune diseases (vitligio, psoriasis, her father has vaculitis churge Strauss syndrome). Neurological examination was free with pain free eye movement, no diplopia . On detailed ophthalmological evaluation, she was found to have the presence of vitreous reaction more on left eye suggesting inflammatory exudates, predominantly near pars plana region (vitritis), thereby diagnosis of bilatral intermediate uveitis (pars planitis) was established, with no retinal (no macular edema), corneal, or choroidal involvement. She was evaluated to exclude other systemic associations such as JIA, sarcoidosis, TINU and MS. Blood tests, CBC with differential, ESR, CRP, Toxocara, CMV and Toxoplasma titres, ACE level, serum creatinine, ANA, (HLA) B27, testing for syphilis, TB, hepatitis, HIV, c-ANCA, p-ANCA and RF all were within normal limits, chest X-ray and MRI brain were within normal limits, urinary β2-microglobulin also normal . Diagnosis was consistent with idiopathic pars planitis with no macular oedema and minimal visual acuity involvement. She was started on local therapy, followed by oral steroid (prednisolone) 1 mg/kg/day for 2 weeks, tappered over the next 4 weeks. Her vision improved dramatically, with V/A 10\10 bilaterally. A relapse after few months bilateral pars planitis affecting Lt eye more (moderate vitritis and minimal decreased in visual acuity of left eye 6\10). She was started a short course of repeat oral prednisolone for 4 weeks and on methotrexate injection at 15 mg/m2/week in April 2021, with consideration of Humira (40 mg/every other week) according to her response.

**Conclusion:** To highlight the importance of the detailed description of type of uveitis by ophthalmologists considering that they are playing a major role and can guide the rest of work up and management in such cases.

**Patient Consent Received**

Yes

**Disclosure of Interest**

None declared

### P132 Visual function and quality of life: a cross-sectional study on a cohort of juvenile idiopathic arthritis-associated and idiopathic uveitis

#### G. B. Beretta^1^, F. Minoia^1^, L. Marelli^2^, C. Mapelli^1^, G. Leone^1^, M. Rossano^1^, T. Giani^3^, P. Nucci^2,4^, E. Miserocchi^5^, R. Cimaz^4,6^ on behalf of Pediatric Rheumatology Associated Group of the Milan Area

##### ^1^Fondazione IRCCS Ca’ Granda Ospedale Maggiore Policlinico; ^2^Ospedale San Giuseppe, IRCCS Multimedica, Milan; ^3^Università di Siena, Siena; ^4^Università degli Studi di Milano; ^5^San Raffaele Scientific Institute; ^6^ASST Gaetano Pini-CTO, Milan, Italy

###### **Correspondence:** G. B. Beretta

**Introduction:** Juvenile idiopathic arthritis (JIA) is the main cause of chronic uveitis in childhood and JIA associated uveitis (JIA-U) is the most common extraarticular complication of JIA. Despite continuous improvement in its management, pediatric uveitis still represents a serious condition with potential sight-threatening complications and a significant impact on quality of life (QoL).

**Objectives:** To evaluate visual function (VF) and QoL in children with JIA-U and idiopathic uveitis.

**Methods:** A cross-sectional study was conducted in two tertiary Pediatric Rheumatology Centres, enrolling all patients seen with JIA-U, JIA without uveitis and idiopathic uveitis. VF was assessed by a translated form of the available EYE-Q, adapted for cross-cultural feasibility into a 10-question tool, while QoL was evaluated by the Italian version of the Pediatric Rheumatology Quality of Life scale part of the Juvenile Arthritis Multidimensional Report (JAMAR), shortened for feasibility to an 8-question tool. JAMAR section on treatment compliance and school attendance was also included. Parents, and patients when appropriate, were asked to complete each patient/parent-reporting outcome measure, answering on a 4-point Likert scale, with a total score ranging from 0 to 72 (worst condition). Medical charts were reviewed regarding JIA and uveitis features and outcome. Quantitative and qualitative variables were compared by means of Mann-Whitney U test or chi-square/Fisher exact test, as appropriate; correlations among quantitative non-parametric variables were evaluated by Spearman’s test.

**Results:** We describe results from 170 patients enrolled (72.9% female), with a median age at study time of 12.9 (9.3-16.4) years. Seventy-seven had JIA-U, 72 JIA without uveitis and 21 idiopathic uveitis. Uveitis was active in 22/98 patients (22.4%), with a median of uveitis duration of 8.0 years (3.7-13.0). Almost all children with uveitis were on systemic treatment (89/98, 90.8%) at the time of interview; 48.0% of patients presented an ocular damage, with 7.1% having a best corrected visual acuity (BCVA) < 4/10. Total score, VF and QoL scores resulted significantly higher in JIA-U patients compared to JIA without uveitis, while no differences were noticed among children with uveitis with or without JIA (Table 1). VF was significantly worse in patients with ocular damage and BCVA < 4/10 (p 0.0242 and 0.0039, respectively). In patients with uveitis, VF and QoL showed a significant correlation (r 0.47, p <0.0001) especially in patients with idiopathic uveitis (r 0.61, p <0.0001).

**Conclusion:** Visual function is a crucial component of QoL in children with uveitis and it correlates with ocular damage. Since eye involvement significantly affects QoL in patients with JIA, a specific tool widely validated and cross-cultural adapted is highly demanded in the clinical care of JIA-U patients.

**Patient Consent Received**

No

**Disclosure of Interest**

None declared

**Table 1 (abstract P132).** See text for description

	JIA-U n = 77	Idiopathic uveitis n = 21	JIA n = 72	p-value*	p-value^#^
Total score	4.0 (2.0-9.0)	4.0 (2.0-9.0)	2.0 (0-4.0)	0.7840	**<0.0001**
VF score	1.0 (0-3.0)	1.0 (0-5.0)	0 (0-0)	0.5801	**<0.0001**
QoL score	3.0 (1.0-5.0)	3.0 (2.0-4.0)	1.0 (0-4.0)	0.8607	**0.0019**

Numbers are medians (IQR). * JIA-U vs idiopathic uveitis; ^#^JIA-U vs JIA

### P133 The features of the articular course in JIA patients, who developed uveitis or not

#### M. Chakhalian, E. Gaidar, T. Nikitina, E. Isupova, I. Chikova, M. Dubko, V. Masalova, T. Likhacheva, L. Snegireva, M. Kaneva, O. Kalashnikova, V. Chasnyk, M. Kostik

##### Saint Petersburg State Pediatric Medical University, Saint-Petersburg, Russian Federation

###### **Correspondence:** M. Chakhalian

**Introduction:** juvenile idiopathic arthritis (JIA) is the commonest rheumatic disease of childhood and JIA-associated uveitis is the most frequent extra-articular manifestation [1]. There is little information on the characteristics of the articular status in JIA children with uveitis.

**Objectives:** our study aimed to describe joint involvement in JIA patients depending on the uveitis.

**Methods:** in the retrospective study 520 JIA children were included.

**Results:** Uveitis was in 116 (22.3%) patients. Patients with uveitis, were ANA-positive (OR=2.4 95% CI: [1.4; 4.0], p=0.001, the number of affected joints was less. The main affected joints in JIA patients with uveitis were knee (22.4%), ankle (19.1%), PIP (16.3%), foot joints (15.2%), MCP (13.5%), elbow (12.8%). The main predictors were oligoarthritis (OR= 2.5 [95%CI: 1.6; 3.9], p=0.00003) and relapsed JIA course (presence of flares) 31% vs 14.9% (OR=2.6 [95%CI: 1.6; 4.2], p=0.00008). Absence of arthritis of specific joints (table) had a protective effect against uveitis.

**Table:** 

Parameter	OR (95%CI)	р
Arthritis of the wrist joint	0,47 (0,26; 0,83)	0,0002
Arthritis of the metacarpophalangeal joint	0,47 (0,26; 0,83)	0,012
Arthritis of the distal interphalangeal joint	0,22 (0,1; 0,7)	0,006
Arthritis of the hip joint	0,37 (0,2; 0,72)	0,003
Talocalcaneal arthritis	0,53 (0,1; 0,83)	0,015
Cervical spine	0,42 (0,2; 0,88)	0,018
Temporomandibular joint	0,11 (0,01; 0,78)	0,007
Shoulder joint	0,17 (0,04; 0,7)	0,006
Elbow joint	0,46 (0,24; 0,88)	0,016

**Conclusion:** relapsed course of JIA, oligoarthicular category, ANA-positivity and involvement of specific joints were predictors of uveitits in JIA patients, whom precise ocular examination required.

**References**

1. Ethan S. Sen, A.V. Ramanan Clinical Immunology, Vol.211, February 2020, 108322.

**Trial registration identifying number:** This work supported by the Russian Foundation for Basic Research (grant № 18-515-57001)

**Patient Consent Received**

Yes

**Disclosure of Interest**

None declared

### P134 The profile of uveitis, required adalimumab treatment: single centre experience

#### M. Chakhalian^1^, E. Gaidar^1^, T. Nikitina^1^, E. Isupova^1^, I. Chikova^1^, M. Dubko^1^, V. Masalova^1^, T. Likhacheva^1^, L. Snegireva^1^, M. Kaneva^1^, O. Kalashnikova^1^, A. Kononov^2^, V. Chasnyk^1^, M. Kostik^1^

##### ^1^Saint Petersburg State Pediatric Medical University; ^2^North – Western State Medical University named after I.I. Mechnikov, Saint-Petersburg, Russian Federation

###### **Correspondence:** M. Kostik

**Introduction:** chronic autoimmune uveitis often associated with juvenile idiopathic arthritis (JIA) or could be alone. For patient whom topical corticosteroids fold, methotrexate (MTX) and subsequent adalimumab (ADA) are treatment options.

**Objectives:** to evaluate features and outcomes of uveitis, required treatment with adalimumamb.

**Methods:** in the retrospective study we used 280 charts of patients with uveitis, treated in the pediatric rheumatology department since 2008 to 2019 year, who had at least 2 years of observation period and had all necessary data for study inclusion. After selection we included 167 JIA patients with uveitis and 17 patients with chronic anterior uveitis (121 girls and 63 boys) whom metotrexate MTX (n=48) or ADA±MTX (n=136) were initiated due to failure of previous treatment. All patients initially received MTX, and ADA was added if MTX fold. Treatment with ADA±MTX might be initiated as first-line in the cases of vision-threatened complications.

**Results:** Uveitis types were: chronic anterior (n=149), peripheral (n=6), posterior (n=3) and panuveitis (n=26). Unilateral involvement was in 54 and 134 had bilateral involvement. JIA categories were distributed subsequently: 118 (70.7%) had oligoarthritis, 32 (19.2%) - polyarthritis, 10 (6.0%) - enthesytis-related arthritis, and 7 (4.1%) - psoriatic arthritis. In 151/167 (90.4%) arthritis preceded to uveitis and in 14 (9.6%) uveitis was before arthritis. ANA positivity was in 56/184 (30.4%) and HLAB27 was in 22/167 (13.2%). Uveitis de novo was in 11/167 (6.6%) patients after etanercept treatment. Uveitis remission achieved 140 (76.1%) in 1.5 (0.4; 4.9) years and 78/140 (55.7%) patients experienced at least one flare in 4.5 (3.2; 6.1) years. In 71 (38.6%) uveitis-related complications were detected during the first visit to our centre in 0.8 (0.2; 2.8) years after uveitis onset and following complications during the treatment developed 27 (14.7%) patients, so 98 (53.3%) patients developed at least one uveitis-related complication during the study. Eye surgery had 29 (15.8%) in 1.8 (0.4; 3.0) years. No differences in gender distribution, number of involvement eyes, ANA and HLA B27 positivity, achievement of the uveitis remission depending the ADA or MTX treatment. Prescribing of ADA was a predictor of uveitis, other than anterior (OR=4.6 [95%CI:1.3; 15.9], p=0.009) primary uveitis-related complications (OR=8.1 [95%CI: 3.0; 21.7), eye surgery (OR=5.7 [95%CI:1.3; 25.0]), oligoarthicular JIA category (OR=2.5 [95%CI:1.2; 5.1]). After ADA initiation the flare frequency, time before flares and frequency of secondary uveitis-related complications became equal.

**Table:** 

Parameter	MTX (n=48)	ADA±MTX (n=136)	p
Uveitis subtypes, n (%)			
anterior	45 (93.8)	104 (76.5)	0.062
peripheral	0 (0)	6 (4.4)	
posterior	0 (0)	3 (2.2)	
panuveitis	3 (6.2)	23 (16.9)	
Chronic uveitis withour arthritis, n (%)	0 (0)	17 (12.5)	0.011
JIA category, n (%)			
oligoarthritis	27 (56.3)	91 (76.5)	0.029
polyarthritis	16 (33.3)	16 (13.4)	
entesytis-related arthritis	3 (6.2)	7 (5.9)	
psoriatic arthritis	2 (4.2)	5 (4.2)	
Time, before remission, years	0.7 (0.1; 3.1)	2.2 (0.6; 5.0)	0.0006
Primary uveitis-related complications, n (%)	5 (10.4)	66 (48.5)	0.000003
Eye surgery, n (%)	2 (4.2)	27 (19.9)	0.010

**Conclusion:** ADA treatment was associated with poor-prognostic uveitis resistant to MTX treatment. Treatment with ADA encouraged to previously non-achievement remission.

**Trial registration identifying number:** This work supported by the Russian Foundation for Basic Research (grant № 18-515-57001)

**Patient Consent Received**

Yes

**Disclosure of Interest**

None declared

### P135 Childhood chronic idiopathic uveitis in a multicentre international cohort: a descriptive analysis

#### I. Maccora^1^, C. Guly^2^, C. De Libero^3^, A. V. Ramanan^4^, G. Simonini^5^

##### ^1^Neurofarba department, Rheumatology Unit, Meyer children’s University Hospital, Florence, Italy; ^2^Bristol Eye Hospital, Bristol, United Kingdom; ^3^Ophathalmology Unit, Meyer children's University Hospital, Florence, Italy; ^4^Paediatric Rheumatology, Bristol Royal Hospital for Children, Bristol, United Kingdom; ^5^Neurofarba department, Rheumatology Unit, Meyer Children's University Hospital, Florence, Italy

###### **Correspondence:** I. Maccora

**Introduction:** Nonetheless the most frequent form of uveitis in paediatric age, there is fair evidence regarding chronic idiopathic uveitis (CIU) in childhood

**Objectives:** To describe the demographic, clinical, laboratory and ophthalmological characteristics of children with CIU

**Methods:** This is a multicentre retrospective chart review observational study recruiting children affected by CIU, who attended the uveitis clinic of Bristol and Firenze Children Hospital (1^st^ Jan2019 - 31^st^ Jan 2020). Demographic, clinical and laboratory data of enrolled children were collected at disease onset, and at 3, 6, 12 months, and the last available follow-up

**Results:** Data of 126 (61 F) children with diagnosis of CIU were entered. The median age at diagnosis was 9.3 years (3-16 years), median time of follow-up 46 months (4-149 months). 111 (88.8%) were Caucasian, 5 (4%) African, 5 (4%) Asian, 3 (2.4%) Arab and 1 (0.8%) mixed. Uveitis was bilateral in 106 patients (84.1%). Sixty-eight patients had an anterior involvement (54%), 29 intermediate (23%), 15 anterior plus intermediate (11.9%), 13 panuveitis (10.3%) and one a posterior involvement (0.8%). Ocular signs and symptoms at onset have been reported in the 77.9% of patients (95), of whom 31.6% reported ocular pain (30), 57.9% ocular redness (50) and 55.8% blurred vision or floaters (53). The mean of ESR was 11.9 mm/h (SD ±12) and of CRP was 0.47 (SD ± 0.92). ANA positivity was reported in the 26.1% of patients, ANCA in the 5.5%. ANA positive patients were younger than ANA negative (F18.1, p<0.001), as well as ANA positivity was more frequently in female gender (χ^2^ 3.9, p 0.04). At the onset, the 54.7% of patients (47) had normal VA, while impaired visual acuity and blindness were reported respectively in the 31.4% (27) and 14% (12). The mean value of best corrected visual acuity (BCVA) was 0.425 LogMAR (SD ± 0.63). At the last available follow-up, the 83.2% had a normal VA (99) while impaired VA and blindness were reported in the 12.6% (15) and 4.2% (6) respectively. The mean value of VA in LogMar was 0.14 (SD ± 0.42). A significant improvement in VA logmar was observed in all patients at the last available follow-up (p <0.001). The 85.6% of patients (89) showed at least one complication at onset, while 46.8% (59) at the last follow-up. Stratifying by different anatomical subtypes, anterior uveitis was more frequently in Asians (χ^2^: 4.5 p= 0.034). ANA were significatively more frequently positive in patients with the anterior subtype (χ^2^: 15.1 p= 0.002). No differences have been detected in age at onset. At the onset, panuveitis had worse BCVA (0.9, p=0.03), an increased number of complications (4, p=0.011), and was associated with the presence of retinal vasculitis (χ^2^ 36.1, p<0.0001). Conversely, anterior uveitis presented more frequently posterior synechiae (χ^2^ 19.1, p <0.000) and band keratopathy (χ^2^15.8, p =0.001). At the last available follow-up, a significative proportion of cataract were observed in panuveitis (χ^2^ 12.3, p 0.006), whilst epiretinal membrane and multifocal choroiditis in intermediate uveitis (χ^2^19.5, p < 0.0001, χ^2^ 21.3 p < 0.0001) and band keratopathy in anterior uveitis (χ^2^ 8.7 p 0.033).

**Conclusion:** We report a large descriptive, retrospective cohort of idiopathic chronic uveitis in childhood. No predominance of gender was observed in idiopathic uveitis, that is in contrast with JIA associated uveitis

**Disclosure of Interest**

None declared

### P136 Effectiveness of Humira in unilateral complicated uveal cataract treatment in juvenile idiopathic arthritis (JIA). Case report

#### M. Lekishvili^1^, M. Gavura^2^, K. Mamamtavrishvili^2^, M. Ioseliani^1^

##### ^1^Rheumatology; ^2^New Hospitals, Tbilisi, Georgia

###### **Correspondence:** K. Mamamtavrishvili

**Introduction:** Juvenile idiopathic arthritis ( JIA) is the most common rheumatic disease of childhood. Ocular inflammation may occur at any time in the course of JIA. JIA associated uveitis its most common extra-articular manifestation. The treatment of the uveitis in children is often unsuccessful and can result in various complications.

**Objectives:** We present case of patient with JIA association uveitis. The patient is a 5 years old female with the unilateral uveitis in JIA diagnosed in 2020. With intact vision in the right and markedly reduced vision in the left eye (VIS OD 20/20, VIS OS 20/2000).

Slit lamp exam shows transparent cornea, anterior uveitis complicated cataract with posterior synechia. eye fundus not visible. IOP is normal in both eyes.

B. Scan Ultrasound (US) - Vitreous body clear, within normal limits (WNL), retina attached.

**Methods:** For 3month the patient was treated with the combination of Methothrexate, 7,5 mg orally once a week, Methilprednizolone 2 mg orally daily and Humira 20 mg per 2 weeks. 2weeks prior the surgery Methotrexate was discontinued. The operation,

Cataract phacoemulsification and intraoccular lens implantation in the

posterior chamber was performed on 5/20/21.

Surgical intervention consisted of the opening of the posterior synechia and the anterior vitrectomy.

**Results:** Marked improvement in vision without exacerbation of the anterior uveitis. Visual acuity was considerably improved to 20/40 in the left eye.

**Conclusion:** Success of the presented case is attributed to the above combination of Used of Methotrtexate, Methilprednizolone and Humira, resulting in marked improvement of vision without postsurgical complications.

**Patient Consent Received**

Yes

**Disclosure of Interest**

None declared

### P137 Predictive factors for lack of response to treatment in a long-term cohort of patients with juvenile idiopathic arthritis-associated uveitis

#### F. Minoia^1^, L. Marelli^2^, F. Pregnolato^3^, G. B. Beretta^1^, C. Mapelli^1^, G. Leone^1^, G. Cincinelli^3^, T. Giani^4^, P. Nucci^2,3^, R. Cimaz^3,5^, E. Miserocchi^6^ on behalf of on behalf of the Pediatric Rheumatology Associated Group of the Milan Area

##### ^1^Fondazione IRCCS Ca' Granda Ospedale Maggiore Policlinico; ^2^Ospedale San Giuseppe, IRCCS Multimedica; ^3^Università degli Studi di Milano, Milan; ^4^Università di Siena, Siena; ^5^ASST Gaetano Pini-CTO; ^6^San Raffaele Scientific Institute, Milan, Italy

###### **Correspondence:** F. Minoia

**Introduction:** Uveitis is the main extraarticular complication of juvenile idiopathic arthritis (JIA) with still a significant impact on JIA morbidity, despite continuous improvement in systemic treatment. Although antinuclear antibody positivity and early onset of JIA have been associated with a high risk of uveitis onset, no clinical features have been widely recognized as predictive factors for JIA-associated uveitis (JIA-U) lack of response to treatment, so far.

**Objectives:** To investigate clinical features associated with lack of response to systemic treatment in a long-term cohort of patients with JIA-U

**Methods:** Clinical records of patients with JIA-U were retrospectively reviewed with regard to clinical features, therapeutic choices and outcome. The role of potential predictors for lack of response to treatment has been assessed at bivariate and multivariate levels. Furthermore, a multivariable logistic model has been applied in order to estimate the strength of association between predictors and outcome, adjusting for potential confounders.

**Results:** Data from 152 JIA-U patients were analysed (82.2% female), with a median follow up of 12.0 years (IQR 9.9) and a median age at uveitis onset of 4.8 (4.1) years. In 72 patients (43.4%) at least one biologic DMARD (bDMARDs) to control uveitis was required. Compared to patients responsive to a monotherapy with a DMARD (n=38), children requiring a bDMARDs for uveitis had a lower median age at uveitis onset, a longer disease duration and a greater frequency of bilateral uveitis at onset (Table). No difference was observed in uveitis activity grade at onset. Despite similar frequency of ocular damage at onset, patients not responsive to DMARDs showed a higher percentage of ocular damage at last visit (66.7% vs 39.5% p=0.011). Multivariable analysis confirmed younger age at disease onset as an independent factor for lack of response to DMARDs (p 0.018). Male gender is associated with higher frequency of ocular surgery (33.3% vs 12.4%, p=0.043), and, despite the inaccuracy of the estimate due to limited sample size, acts as an independent factor in multivariable analysis with an almost 9 times higher risk to lack of response to DMARDs (p=0.049).

**Table:** 

	Monotherapy with DMARDs (n=38)	bDMARDs for uveitis (n=72)	p-value
Gender, %M (n)	7.9 (3)	25.0 (18)	0.055
Age at uveitis onset (yr), median (IQR)	6.0 (3.3)	4.1 (4.3)	0.008
Duration of disease (yr), median (IQR)	10.4 (8.0)	13.4 (10.7)	0.047
Oligoarthritis persistent course, % (n)	84.2 (32)	72.2 (52)	0.053
ANA positive, % (n)	89.5 (34)	94.4 (68)	0.444
Positive acute phase reactants at onset, % (n)	68.2 (15)	78.4 (29)	0.575
Active arthritis at uveitis onset, % (n)	57.6 (19)	72.7 (48)	0.197
Ocular damage at onset, % (n)	18.2 (6)	26.9 (18)	0.480
Bilateral uveitis at onset % (n)	50.0 (19)	77.8 (56)	0.006

**Conclusion:** Younger age at uveitis onset and male gender are predictors of a worse response to DMARDs, while the length of follow-up exerts a confounding effect on bilateral uveitis. Children resistant to conventional treatment need prompt recognition and additional strategies to improve long-term outcome

**Disclosure of Interest**

None declared

### P138 Paediatric non-infectious granulomatous uveitis: retrospective cohort study

#### A. T. Nguyen^1^, A. Rousseau^2^, B. Bodaghi^3^, P. Dusser^1^, L. Rossi^1^, C. Galeotti^1^, E. Da Cunha^2^, L. Eid^2^, M. Labetoulle^2^, E. Barreau^2^, C. Titah^4^, I. Koné-Paut^1^, C. Borocco^1^

##### ^1^Department of Paediatric Rheumatology, CeReMAIA, CHU Bicêtre, Assistance Publique - Hôpitaux de Paris, Université de Paris Saclay; ^2^Department of Ophthalmology, OPHTARA, CHU Bicêtre, Assistance Publique - Hôpitaux de Paris, Université de Paris-Saclay, Le Kremlin-Bicêtre; ^3^Department of Ophthalmology, Hôpital de la Pitié-Salpêtrière, Assistance Publique - Hôpitaux de Paris; ^4^Department of Ophthalmology, Fondation Ophtalmologique de Rothschild, Paris, France

###### **Correspondence:** A. T. Nguyen

**Introduction:** Paediatric-onset granulomatous uveitis (PGU) is rare. In addition, the lack of awareness often leads to diagnosis delay and poor visual outcome. Determining the underlying cause is essential and challenging. In addition, the care of these patients still lies on very little data.

**Objectives:** To evaluate the demographics, aetiologies, complications, treatments and visual prognoses of paediatric non-infectious granulomatous uveitis

**Methods:** Retrospective chart review of PGU occurring in children before 16 years of age and recruited from the paediatric rheumatology department at Bicêtre Hospital, France from 2001 to 2021. Our study excluded infectious uveitis. Our definition of inactive disease and remission followed the Standardization of Uveitis Nomenclature criteria. (1)

**Results:** We included 43 patients with 80 affected eyes: 24 had idiopathic uveitis, 13 had sarcoidosis-associated uveitis, 3 had *NOD2* mutation-associated uveitis, 2 had juvenile idiopathic arthritis-associated uveitis, and one had Vogt-Konayagi-Harada disease. The median age at diagnosis was 10.2 years. Sex-ratio M/F was 0.72. Features of PGU were mostly: panuveitis (63%), bilateral (84%), and evolving chronically (84%). Granulomatous features consisted in mutton-fat keratic precipitates (65%) in all aetiologies. Choroidal granulomas and iris nodules were present more in sarcoidosis-associated uveitis than in idiopathic uveitis (31% vs 17%, p=0.4 and 38% vs 8%, p=0.07). All but 5 patients with sarcoidosis had ocular symptoms before other manifestations, which included mainly lung damage in 38%, then liver damage (23%), lymph node involvement (23%), lymphocytic meningitis (23%), arthritis (15%), hearing loss (8%), and cutaneous lesions (8%). Features leading to diagnosis of sarcoidosis were mainly lymphopenia, hypergammaglobulinemia, elevated lysozyme and ESR. None of the investigations were relevant for the diagnosis of Blau syndrome, excepted screening for *NOD2* mutation. None of the patients with *NOD2* mutation exhibited neither granulomatous polyarthritis and dermatitis, nor extraocular symptom. Ocular complications at diagnosis and/or during follow-up were present in 62 eyes out of 80 (78%). The most used treatments were systemic corticosteroids (79%), methotrexate (79%), TNF-alpha monoclonal antibody (42%), and azathioprine (16%). Twenty-seven per cent of eyes were on remission at last follow-up, 67% were inactive and 5% remained active. The median duration of follow-up was 4.8 years.

**Conclusion:** We report herein the first significant cohort focused on paediatric non-infectious granulomatous uveitis. PGU are idiopathic in most cases. Sarcoidosis-associated uveitis are associated to laboratory tests abnormalities. A high rate of complications is associated with PGU. However, a significant proportion of uveitis became inactive or achieved remission, following the use of systemic treatments as corticosteroids, immunosuppressants and/or biologics.

1. Jabs DA, Nussenblatt RB, Rosenbaum JT, Standardization of Uveitis Nomenclature (SUN) Working Group. Standardization of uveitis nomenclature for reporting clinical data. Results of the First International Workshop. Am J Ophthalmol. sept 2005;140(3):509-16.

**Patient Consent Received**

Yes

**Disclosure of Interest**

None declared

### P139 Long-term experience of biological therapy in juvenile idiopathic arthritis associated with uveitis: single center experience

#### S. Arsenyeva^1^, I. Nikishina^1^, M. Kaleda^1^, A. Shapovalenko^1^, E. Denisova^2^, A. Panova^2^

##### ^1^Paediatric, V.A. Nasonova Scientific Research Institute of Rheumatology; ^2^Paediatric, Helmgoltz Moscow Research Institute of Eye Diseases, Moscow, Russian Federation

###### **Correspondence:** I. Nikishina

**Introduction:** Biological agents (BA) are high efficacy options for current therapy for patients (pts) with juvenile idiopathic arthritis (JIA). They are successfully used not only for the arthritis but also for JIA-associated uveitis.

**Objectives:** to evaluate the main clinical features of pts with JIA-associated uveitis, who needed to treat by BA, the spectrum of BA, reasons for indication and withdrawals.

**Methods:** retrospective cohort study included all JIA pts (1136) who were treated with BA in our clinic from 2002 to 2020. All cases of JIA-associated uveitis were collected in special study for the describing of their clinical features, JIA category, exposure to Methotrexate (MTX) and BA, presence of ANA, HLA B27.

**Results:** among of 1136 pts treated with different BA we identified 204 (18 %) pts (71 male/133 female; 35% and 65% respectively), included 36 (3.2%) pts (19 female /17 male) with uveitis *de novo* under BA. JIA subtypes were as follows: RF-neg polyarthritis 25 (12%), persistent oligoarthritis 76 (37%), extended oligoarthritis 92 (45%), enthesitis-related arthritis (ERA) - 9 (4%). Average age at JIA onset was 4.3 yrs (Me 3,0, min 0,3, max 15,3 yrs). Duration of disease before BA treatment was 5,1 yrs (Me 4,3, min 0,2 max 16,6 yrs). Average age of BA treatment start was 9,4 yrs (Me 9,4, min 1,7 max 18 yrs). 149/204 pts were ANA-positive (73%), 63/204 pts had HLA B27 (31%), including 24 pts who had the both features. 171/204 (84%) of pts received MTX. Due to high activity of arthritis and uveitis BA treatment was started, 305 treatment series in total, including 175 for Adalimumab (1^st^ line – 124(71%), 2^nd -^ 45, 3^rd^ - 6). Infliximab was indicated in 37 in 2002-2012, as a “historical cohort” (1^st^ line - 32, 2^nd -^ 4, 3^rd –^ 1). Abatacept was given in 31- mostly in patient without HLA B27 and not ERA subtypes, and in pts with high risk of infection, especially, tuberculosis (1^st^ line - 20, 2^nd -^ 9, 3^rd^ -1, 4^th^ -1). 15 pts were treated by Golimumab (1^st^ line - 0, 2^nd -^ 10, 3^rd –^ 4, 4^th^-1), 11 – Tocilizumab, mostly in severe polyarthritis and high laboratory activity (1^st^ line - 2, 2^nd -^ 2, 3^rd –^ 5, 4^th^ – 1), 2 - Sarilumab (3^rd –^ 2). Etanercept was not indicated in pts with uveitis, but we observed 34 pts, who developed uveitis *de novo* (1^st^ line - 26, 2^nd -^ 4, 3^rd –^ 4). There were 116 cases of withdrawals. Secondary inefficacy was as the reason for withdrawals in 47/116 (41%): Infliximab 15/37 (40 %), Adalimumab 22/175 (13%), Abatacept – 8/31 (26%), Tocilizumab – 2/11(18%). Withdrawals due to AE were found in 55/116 (47%): Etanercept – 34 cases of uveitis de novo; Infliximab – 13/37 (35%); Adalimumab – 4/175 (2%), Abatacept -4/31 (13%); others – 14 (12%), basically due to non-medical reasons, including difficulties in BA access in adulthood.

**Conclusion:** Our experience demonstrated that Adalimumab is still the preferred alternative for the treatment of JIA-associated uveitis with the best drug survival. In case of its secondary inefficacy Golimumab may be as the next option. Abatacept and Tocilizumab were indicated if there were additional reasons.

**Disclosure of Interest**

None declared

### P140 Demographic and clinical characteristics of children with refractory JIA-associated uveitis receiving biological therapy

#### E. Baranovskaya, A. Berbenyuk, L. Galstyan, E. Popova, E. Zholobova

##### Institute of Child's Health, Sechenov University, Moscow, Russian Federation

###### **Correspondence:** A. Berbenyuk

**Introduction:** Juvenile idiopathic arthritis (JIA) is the most common rheumatic disease among children. Uveitis is the extra-articular manifestation which occurs in approximately 8-30% of patients depending on the clinical form of JIA (1). JIA-uveitis is frequently anterior, often asymptomatic and is associated with oligoarthritis and rheumatoid factor (RF) negative polyarthritis. Disease-modifying antirheumatic drugs (DMARDs) normally used for uveitis management are showing positive effect in about 70% of cases (2). Biological agents are a second-line treatment for JIA-uveitis in case of DMARDs ineffectiveness. As JIA uveitis is a sight-threatening manifestation, it is important to discover the risk factors of severe disease refractory to DMARDs to start biological treatment timely.

**Objectives:** To address the features of refractory to DMARDs JIA-associated uveitis by evaluating demographic and clinical characteristics of children with JIA receiving biologics.

**Methods:** This open-label, single-center, observational (2020–ongoing) cohort study included 43 patients (4.5–17.5 y/o) with JIA and associated rheumatoid uveitis refractory to DMARDs who required biological treatment. A percentage ratio, Chi-square test for categorical data and Me (Q1, Q3) with preceding Shapiro-Wilk test for numerical data were used.

**Results:**

**Table:** 

Age, y/o	Number of patients, n	Percentage, %
Before 1	3	7
1 – 2	13	30.2
2 – 5	20	46.5
6 – 11	6	14
12 – 18	1	2.3

in this cohort of patients with JIA-associated uveitis (n=43) a median age was 11.7 (IQR 7.2, 14.3) y/o, with girl/boy ratio of 2/1. Oligoarthritis was seen in 62.8% (n=27), RF negative polyarticular – in 30.2% (n=13) of patients; 4.7% (n=2) of children were diagnosed with enthesitis-related arthritis, 2.3% (n=1) – with psoriatic arthritis. JIA manifested at the median age of 2.6 (IQR 1.6, 4.4): before 1 y/o – 7% (n=3), 1–2 y/o – 30.2% (n=13), 2–5 y/o – 46.5% (n=20), 6–11 y/o – 14% (n=6), 12–18 y/o – 2.3% (n=1) (Table 1). Articular syndrome preceded in 83.8% (n=36), uveitis – in 9,3% (n=4), and simultaneous joint and eye involvement was seen in 6.9% (n=3) of children. Uveitis OU occurred in 74.5% (n=32, girl/boy ratio 2.2/1), single-eye uveitis – in 25.5% (n=11; girl/boy ratio 1.75/1) of patients, though overall no statistically significant correlation was revealed between sex and number of eyes involved (R=0.04). Time between articular syndrome manifestation and eye involvement (and vice versa if uveitis preceded) was 23 (IQR 7, 48) months, time before biological treatment initiation – 36 (IQR 14, 60) months. Antinuclear factor (ANF) positivity prevailed (74.4 %; n=32), with no statistically significant correlation between sex and ANF positivity (R=0.066). Most patients were initiated with adalimumab (n=39), some of them – with etanercept (n=3) or abatacept (n=1).

**Conclusion:** The group of patients with JIA-associated uveitis refractory to DMARDs is characterized by female predominance, early manifestation of arthritis and oligoarthritis. These features could be considered as factors in favour of earlier biological therapy initiation in case of conventional therapy ineffectiveness.

References

1. Nordal E, Rypdal V, Christoffersen T, Aalto K, Berntson L, and for the Nordic Study Group of Pediatric Rheumatology (NoSPeR). Incidence and predictors of Uveitis in juvenile idiopathic arthritis in a Nordic long-term cohort study. Pediatr Rheumatol. 2017 г.;15(1):66.

2. Galstyan L.A., Zholobova E.S., Chebysheva S.N., Meleshkina A.V., Seraya V.A., Loskutova O.Yu. Uveitis associated with juvenile idiopathic arthritis. Ros Vestn Perinatol i Pediatr. 2019; 64:(2): 30–37

**Patient Consent Received**

Yes

**Disclosure of Interest**

E. Baranovskaya: None declared, A. Berbenyuk: None declared, L. Galstyan: None declared, E. Popova: None declared, E. Zholobova Speaker Bureau of: Pfizer, AbbVie, Novartis, Roche

### P141 The impact of selecting different outcome measure on the results of juvenile idiopathic arthritis associated uveitis treatment - the longitudinal observational study

#### M. Barisic Kutija^1^, M. Sestan^2^, S. Peric^1^, N. Kifer^2^, P. K. Ivkic^1^, M. Galiot Delic^1^, J. Knezevic^1^, M. Held^2^, M. Frkovic^2^, M. Jelusic^2^, N. Vukojevic^1^

##### ^1^University Hospital Centre Zagreb, Department of Ophthalmology, University of Zagreb School of Medicine; ^2^University Hospital Centre Zagreb, Department of Pediatrics, University of Zagreb School of Medicine, Zagreb, Croatia

###### **Correspondence:** M. Sestan

**Introduction:** The number of patients with juvenile idiopathic arthritis associated uveitis (JIA-U) on systemic immunomodulatory treatment (IMT) is relatively small, especially those who need IMT for disease control. Variabilities in the way patients are selected and the results presented in different studies on the effectiveness of IMT make it very difficult to compare studies with each other.

**Objectives:** The aim of this study was to show on the same sample of JIA-U patients how different the obtained levels of therapy efficacy are depending on the selected definitions of outcomes in the long-term management with IMT.

**Methods:** The longitudinal observational study with JIA-U patients treated with IMT was conducted at University Hospital Centre Zagreb in the period from 2011 to 2017.

**Results:** We included 38 JIA-U patients aged 2 to 15 years and 69 eyes respectively, since 7 patients had unilateral JIA-U. Median (range) time of follow up was 209 (19-381) weeks. At the first examination 46 (66.7%) eyes had grade ≤1+ of inflammation in anterior chamber (AC) according to Standardization of Uveitis Nomenclature (SUN) Working Group criteria, 11 (15.9%) had grade 2+, while 3 (4.4%) eyes had grade 3+ of inflammation. At baseline, 23 children (60.5%) had already received methotrexate (MTX) therapy and 8 (21.0%) biologics, while 4 (10.5%) children were treated with systemic glucocorticoids (GC). Topical glucocorticoids (TGC) in the form of drops were used to treat JIA-U in 61 (88.4%) eyes with a median of 4 daily doses. Most patients were treated concomitantly with GC ointment (75.4% of the eyes) with a median of 1 daily dose. Until the end of the follow-up, all children received MTX at least for some period, and 40% of patients were treated with biologics. The results of the effectiveness of IMT are presented according to the reduction of the need for TGC therapy and according to the achieved level of inflammation in AC. In the first 12 months of follow-up, among JIA-U patients treated with both biologics and MTX, in 65% of eyes there was no need for TGC therapy. Overall, in the 48th month of follow-up, in 50% of eyes there was no need for TGC therapy, and the rest required 1-2 daily doses of TGC. At the end of the first year, with MTX and biological therapy 75% of eyes had grade 0 of inflammation in AC and in 48th month 61.1% of eyes achieved grade 0 of inflammation. In the 12th month of application of biological therapy, in addition to MTX, in our study in 75% of eyes a grade 0 of inflammation was achieved with ≤2 doses of TGC, and in the 48th month in 61.1% of eyes. If the results are presented according to milder criteria, then in the 12th month of follow-up 90% of the eyes have a degree of inflammation ≤0.5+ with ≤2 doses of TGC, and in the 48th month all patients achieved a degree of inflammation ≤0.5+ with ≤2 doses of TGC.

**Conclusion:** It was shown that the results of treatment outcomes during follow-up largely depend on the selected outcome measures, i.e. the criteria for the effectiveness of therapy. This will be important for future research because it suggests caution that in the pursuit of better results, setting different limits can lead to a more favorable outcome.

**Patient Consent Received**

No

**Disclosure of Interest**

None declared

### P142 The influence of systemic immunomodulatory treatment on the intensity of topical glucocorticoid therapy in patients with juvenile idiopathic arthritis-associated uveitis - longitudinal observational study during 7 years

#### M. Barisic Kutija^1^, M. Sestan^2^, S. Peric^1^, N. Kifer^2^, P. K. Ivkic^1^, M. Galiot Delic^1^, S. Jandrokovic^1^, M. Held^2^, M. Frkovic^2^, M. Jelusic^2^, N. Vukojevic^1^

##### ^1^University Hospital Centre Zagreb, Department of Ophthalmology, University of Zagreb School of Medicine; ^2^University Hospital Centre Zagreb, Department of Pediatrics, University of Zagreb School of Medicine, Zagreb, Croatia

###### **Correspondence:** M. Sestan

**Introduction:** Juvenile idiopathic arthritis associated uveitis (JIA-U) is the most common and potentially most destructive extraarticular manifestation of JIA. The main goal of the treatment is to achieve complete suppression of intraocular inflammation, maintain visual acuity, prevent relapses and complications and avoid the side effects of systemic and topical medications.

**Objectives:** The aim of this research was to determine the need for topical glucocorticoid therapy (TGC) in patients with JIA-U on systemic biological therapy in comparison to patients treated with methotrexate (MTX) only.

**Methods:** We have conducted longitudinal observational study in which we included JIA-U patients in whom systemic immunomodulatory treatment (IMT: biologics and/or MTX) was introduced and who were followed at least 3 months in the period between 2011 and 2017. The data about the number of cells in the anterior chamber (AC) according to Standardization of Uveitis Nomenclature (SUN) Working Group criteria, about TGC and systemic therapy and JIA complications were collected on each examination. Generalized linear mixed models were used to analyze the relationships between treatment with biologics, MTX, TGC and the grade of inflammation in AC according to SUN criteria.

**Results:** 38 JIA-U patients (69 eyes) with median (range) age of 4.9 (2-15) years and follow up period of 209 (19-381) weeks were included. There were a total of 1205 examinations. At the first examination JIA-U was detected in 16 (42.1%) of patients, 59 (79.7%) of the eyes had ≤1+ cells in the AC, and in 19 (50%) of JIA-U patients complications were already present. MTX was introduced in 23 (60.5%) JIA-U patients before the inclusion in the study, 8 (21%) has already received biologics, while in 4 (10.5%) prior systemic glucocorticoids were also used. Until the end of the study, all patients received MTX and 40% JIA-U patients were treated with biologics. The average number of TGC doses decreased steadily in the first 12 months, from 3.74 doses at baseline, 0.95 doses at 12th month to 0.72 doses in the 48th month of follow-up. After Friedman and the post hoc test a statistically significant difference in the daily doses of TGC could be seen from the 12th month after application of systemic IMT. The number of daily doses of TGC per eye as well as the degree of inflammation in AC per eye decreased over time. Using generalized linear mixed models it was shown that the treatment with biologics, but not with MTX and systemic glucocorticoids, was associated with lower intensity of TGC therapy. Treatment with biologics and systemic glucocorticoids, but not with MTX, was associated with lower degree of inflammation in AC.

**Conclusion:** The results showed that the application of systemic biological therapy may result in less intensive TGC therapy, resulting in glucocorticoid-sparing potential, and reducing intraocular inflammation.

**Patient Consent Received**

No

**Disclosure of Interest**

None declared

### P143 Adalimumab in a cohort of children with juvenile idiopathic arthritis: a single-center experience

#### G. Tarantino^1^, D. Pires Marafon^1^, G. Zinzanella^2^, A. Uva^3^, A. Aquilani^1^, G. Marucci^1^, R. Nicolai^1^, D. Rigante^4^, F. De Benedetti^1^, S. Magni-Manzoni^1^

##### ^1^Rheumatology; ^2^Ophtalmology, Ospedale Pediatrico Bambino Gesù, Rome; ^3^Division of Paediatrics, Ospedale di Ravenna, Ravenna; ^4^Division of Paediatrics, Policlinico Agostino Gemelli, Rome, Italy

###### **Correspondence:** G. Tarantino

**Introduction:** Uveitis is the most serious extra-articular complication of juvenile idiopathic arthritis (JIA). Screening at-risk patients is essential as well as prompt treatment to minimize intra-ocular inflammation and preserve visual function with steroid-sparing immunomodulatory drugs (i.e. c-DMARDs) or TNF-α inhibitors (b-DMARDs).

**Objectives:** To describe demographic and clinical features in a single-center cohort of children with JIA treated with adalimumab (ADA), grouped according to the presence or absence of uveitis (JIA-U), and to observe the disease course in the JIA-U group during a 36-month-period of follow-up (FU).

**Methods:** Records of JIA patients treated with ADA throughout 2019 were retrospectively reviewed to assess joint involvement and ophthalmological examinations. Children with FU <12 months were excluded. Uveitis was diagnosed according to the SUN Working Group criteria. Data were analyzed through descriptive statistics.

**Results:** Of 109 patients with JIA, only 96 were included in the study (Table 1). None of them presented RF-positive polyarthritis, nor systemic JIA (sJIA). In JIA-U cohort, 60% (35/58) presented isolated joint involvement at onset. Median time from diagnosis to uveitis was of 19 months. Articular and ocular inflammation occurred simultaneously in 19 children; uveitis preceded arthritis in 6.9%. Each uveitis episode was initially treated with topical steroids and mydriatics. Most of JIA-U patients (87.9%) were already on c-DMARDs at ADA-start. Compared to the cohort without uveitis, they were younger both at onset and at baseline and started TNF-α inhibition later. Forty-five (77.6%) experienced recurrent uveitis (median frequency of 3 episodes) before starting ADA. Among JIA-U patients, there were no differences in gender, age at disease onset, ILAR JIA subtype and ANA positivity between active disease (AD) and clinical remission on therapy (CM) cohorts at 6 and 12 months from baseline. At baseline, 32 of the JIA-U children showed ocular complications, including posterior synechiae (n=29), visual loss (n=10, BCVA 20/40), cataract (n=9), band keratopathy (n=6) and glaucoma (n=1). Uveitis flares between 12-24 months were mostly bilateral, as well as at baseline; those occurred by 6 months from ADA start and after withdrawal were more frequently unilateral.

**Table:** 

	TOTAL (N=96)	WITH JIA-U (N=58)	WITHOUT JIA-U (N=38)
Female, n (%)	67 (69.8)	48 (82.8)	19 (50.0)
Age at disease onset (years), median (IQR)	3.7 (2.1-9.6)	2.5 (1.9-4.2)	10.0 (4.9-13.0)
Antinuclear antibody (ANA) positive, n (%)	69 (71.9)	54 (93.1)	15 (39.5)
JIA Classification (ILAR Criteria), n (%)			
Persistent oligoarthritis	41 (42.7)	33 (56.9)	8 (21.1)
Extended oligoarthritis	25 (26.0)	15 (25.9)	10 (26.3)
RF-negative polyarthritis	18 (18.8)	8 (13.8)	10 (26.3)
Enthesitis-related arthritis	8 (8.3)	1 (1.7)	7 (18.4)
Psoriatic arthritis	4 (4.2)	1 (1.7)	3 (7.9)

**Conclusion:** Results about real-life use of ADA at our center confirm its role as first-choice option among b-DMARDs for children with refractory JIA-U, according to a traditional “step-ladder” model. A targeted risk analysis of clinical and/or laboratories parameters was not available. Further prospective studies evaluating the appropriate withdrawal timing and determining the risk factors for relapse are warranted.

**Disclosure of Interest**

None declared

## e-Poster viewing: Immunodeficiency and infection related arthritis

### P144 Parasitic rheumatism – an unusual cause of chronic polyarthritis

#### A. Cristóvão Ferreira^1^, A. R. Claro^1^, J. Gil^1^, M. Cabral^2^, H. S. Sousa^3^

##### ^1^Pediatrics, Centro Hospitalar Universitário Lisboa Norte; ^2^Pediatrics, Hospital Professor Doutor Fernando Fonseca, Lisbon; ^3^Pediatrics, Hospital Vila Franca de Xira, Vila Franca de Xira, Portugal

###### **Correspondence:** A. Cristóvão Ferreira

**Introduction:** In Europe, parasites are an exceptionally rare etiology of infection-related arthritis.

**Objectives:** To recall the importance of excluding parasitic infestations in a child with chronic arthritis.

**Methods:** Description of a case report.

**Results:** A 12-year-old (yo) boy, born and raised in Sub-Saharan Africa (São Tomé and Príncipe), arrived in Portugal 5 days before admission, with no past medical report. Since 5-yo he presented with chronic asymmetric migratory polyarthritis with inflammatory pain and movement limitation of the cervical spine, elbows, wrists, knees, ankles, hands and feet’ fingers. Despite fluctuating, there were no symptom-free intervals, and, since its presentation, there was no disease progression. Some episodes of self-limited fever and tonsillitis, vomiting and diarrhoea, and two hospital admissions (gastroenteritis and malaria) were reported.

The national immunization program was updated, and both growth and neurodevelopment were regular. Familiar history was unremarkable.

On examination, he was slightly pale, apyretic, prepubertal, weight 29,5Kg, height 135cm (P3, WHO), BMI 16,8Kg/m2 (P15, WHO), with a limping gait. Pain and tenderness of cervical spine and ankles. Pain, swelling and decreased range of motion of the right elbow and wrist, both knees and small joints of the hand and feet. He also had sausage-like toes. The remainder examination was normal.

Laboratory investigation revealed an erythrocyte sedimentation rate of 86mm/h, C-reactive protein 3,35mg/dL, normal leucocyte count with eosinophilia (536/μL), normocytic anaemia (hemoglobin 11,2g/dL), 404000 platelets/uL. Routine biochemical tests, urinalysis, serum immunoglobulins and complement fractions were normal. Rheumatoid factor, antinuclear antibodies and HLA-B27 were negative. ASO and DNase-B were elevated and throat culture positive for *S. pyogenes*. Wide serological tests (HIV, B/C hepatitis, parvovirus B19, CMV, *M.pneumoniae, B.burgdorferi, B.henselae*), tuberculin test, IGRA, blood and stool cultures were negative. Stool ova and parasites exam revealed many larvae and adult forms of ***Ascaris Lumbricoides*** and ***Trichuris trichiura*** and positive ***Giardia*** antigen in stool. Abdominal ultrasound revealed impressive signs of parasitic infestation in different maturation phases and with variable size. Plain x-rays of affected joints revealed soft tissue swelling. There was no joint effusion on ultrasonography of the left knee and right wrist. Ophthalmologic and cardiac evaluations were normal. He was treated with non-steroidal anti-inflammatory drugs (NSAIDs) and on day 7 metronidazole was started with impressive clinical improvement. On day 12, a single dose of intramuscular benzylpenicillin was administered. One month later, he was asymptomatic and laboratory values were normal. When re-evaluated 3 years later, the patient was still free of symptoms.

**Conclusion:** Parasitic rheumatism is a rare condition characterized by inflammatory aseptic joint manifestations due to a parasitic infestation. The clinical presentation of this reactive arthritis may be very heterogeneous and can mimic the clinical picture of different inflammatory rheumatic diseases making it a challenging differential diagnosis. It seems to rely on genetic predisposition, which may explain its uncommon occurrence despite the high prevalence of parasitic infestation worldwide. The failure of NSAID’s, along with the notable efficacy of specific anti-parasitic treatment in a patient with a parasitic infestation, are the main hallmarks.

When caring for a patient coming from an endemic area of parasitosis with osteoarticular complaints, even with none or mild gastrointestinal manifestations, a high index of suspicion is suggested, and when correctly treated, the prognosis is excellent.

**Patient Consent Received**

Yes

**Disclosure of Interest**

None declared

### P145 Clinical features of paediatric HIV arthropathy

#### M. Harrison^1^, N. Brice^2,3^, K. Webb^2,3^, W. Slamang^2,3^, C. Scott^2,3^

##### ^1^Department of Paediatrics, Fort Beaufort Hospital, Fort Beaufort; ^2^Division of Paediatric Rheumatology, Department of Paediatrics and Child Health, Red Cross War Memorial Children’s Hospital; ^3^Department of Paediatrics and Child Health, University of Cape Town, Cape Town, South Africa

###### **Correspondence:** M. Harrison

**Introduction:** Advanced HIV infection is associated with an inflammatory arthritis, however few reports have described this disorder in children.

**Objectives:** This study aimed to describe the clinical features of HIV arthropathy in a case series of children in South Africa and compare these with features of JIA.

**Methods:** Retrospective data were collected from HIV-infected children with HIV arthropathy enrolled in a Paediatric Rheumatology clinic in Cape Town, South Africa. Data from a recently described, published cohort of children with JIA enrolled in the same clinic were included for comparison. Ethical approval was granted by the Human Research Ethics Committee of the University of Cape Town, with a waiver for consent.

**Results:** Eleven cases of HIV arthropathy were identified. Cases predominantly affected boys (8/11), and the median age of onset was 10.3 years (IQR 6.9 – 11.6). Most cases presented in the setting of advanced immunosuppression, with a median absolute CD4+ count of 389 cells/uL (IQR 322 – 449) and median CD4+ proportion of 19.5% (IQR 14.8 – 25.0) at presentation. The clinical presentation was variable, with both oligoarthritis (6/11) and polyarthritis (5/11) being prevalent. All cases exhibited large joint involvement, which was usually asymmetrical. In addition, four children had asymmetrical small joint involvement. Associated features included enthesitis (4/11) and dactylitis (1/11). The most consistent laboratory feature was elevated acute phase reactants, and typical ultrasonographic findings were joint effusions and synovial hypertrophy. JIA and HIV arthropathy presented at a similar age, with median age at HIV arthritis onset of 10.3 years (IQR 6.9 – 11.6) versus 9.25 years (IQR 4.5 – 12.3) at arthritis onset in the JIA subgroup. HIV arthropathy cases were predominantly male (M/F ratio 3.0), whereas JIA cases had an equal sex distribution (M/F ratio 0.9). Oligo-articular disease was more frequently described in children with HIV arthropathy (55%), compared to those with JIA (38%).

**Conclusion:** In this series, most cases of HIV arthropathy exhibited asymmetrical large joint oligoarthritis or polyarthritis, and presented in older boys with advanced immunosuppression. HIV arthropathy appears to present at a similar age to JIA, with a comparable pattern of joint involvement to oligo-articular and poly-articular JIA subtypes.

**Patient Consent Received**

No

**Disclosure of Interest**

None declared

## e-Poster viewing: Macrophage activation syndrome

### P146 Efficacy of emapalumab in chronic/relapsing macrophage activation syndrome in systemic juvenile idiopathic arthritis with lung and liver involvement

#### A. Arduini, M. Pardeo, A. De Matteis, G. Marucci, I. Caiello, G. Prencipe, F. De Benedetti, C. Bracaglia

##### Division of Rheumatology, IRCCS Ospedale Pediatrico Bambino Gesù, Roma, Italy

###### **Correspondence:** A. Arduini

**Introduction:** MAS is a severe complication of sJIA. IFNγ is a major driver of hyperinflammation and hypercytokinemia.

**Objectives:** To report the response to the anti-IFNγ antibody, emapalumab, in a patient with sJIA and chronic relapsing MAS.

**Methods:** Serum levels of IL-18, CXCL9 and neopterin were measured by ELISA.

**Results:** A 6 years old Caucasian girl, presented a first episode of HLH in April 2017, with persistent fever, hepatosplenomegaly, CNS involvement with seizures, marked hyperferritinemia and high CXCL9 levels (Table). She met the HLH-2004 primary HLH criteria and she was treated with emapalumab in the context of the primary HLH trial. She had complete remission with normalization of CXCL9. In June 2018 (after 10 months from last emapalumab) she presented a progressive increase in liver enzymes (ALT 1333 UI/l, AST 851 UI/l, LDH 822 UI/l), with no other HLH features. The liver biopsy was suggestive for autoimmune hepatitis. Treatment with oral prednisone (1 mg/kg/die) was started with progressive normalization of liver enzymes and continued for 8 months up to February 2020. During these months she presented recurrent episodes of fever with thrombocytopenia, increased levels of inflammatory markers, transaminases, LDH with mild hyperferritinemia (< 1500). All these episodes were managed with transient increase in GCs. In February 2020, she developed arthritis (wrists and knee) and she was referred to our attention. At this time she met the ILAR criteria for sJIA and the EULAR/ACR MAS criteria (ferritin 3.460, PLT 64.000, triglyceride 220; fibrinogen 385, AST 100). Abdominal CT showed splenomegaly and hepatomegaly, with disseminated round lesions. Chest CT showed initial signs of interstitial lung disease. Intravenous methylprednisolone (3 pulses of 30 mg/kg) followed by oral prednisone (0.5 mg/kg) and anakinra (5 mg/kg) were started without significant improvement. She then presented a further episode of MAS with severe marrow and liver involvement. CXCL9, neopterin and IL-18 levels were persistently elevated. Emapalumab was started, on compassionate use regimen. After 5 months of emapalumab she achieved complete clinical remission with normalization of laboratory parameters. GCs were tapered to <0.2 mg/Kg after 4 months and discontinued after 7 months. After 13 months of emapalumab, CT showed complete resolution of the liver nodular lesions, of splenomegaly and of lung involvement. CXCL9 and neopterin levels normalized, while IL-18 levels decreased, but remained persistently elevated.

**Conclusion:** This case shows that IFNγ neutralization may represent a valid therapeutic approach in patients with chronic/relapsing MAS with liver and marrow involvement.

**Patient Consent Received**

Yes

**Disclosure of Interest**

A. Arduini: None declared, M. Pardeo: None declared, A. De Matteis: None declared, G. Marucci: None declared, I. Caiello: None declared, G. Prencipe: None declared, F. De Benedetti Consultant for: for Abbvie, SOBI, Novimmune, Novartis, Roche, Pfizer, C. Bracaglia Consultant for: SOBI, Novartis

**Table 1 (abstract P146):** **.** Laboratory parameters and cytokine levels during disease course

	Range	26/04/2017	19/02/2018	13/06/2018	24/02/2020	08/04/2020	22/04/2021
		Onset primary HLH	Remission after emapalumab	Onset of liver involvement	sJIA critria and full-blown MAS	Start emapalumab	1 year after emapalumab
**GB (10^3 /uL)**	5.5-15	12,27	6,44	6,09	7,48	0,91	4,8
**PLT (10^3 /uL)**	150-450	46	438	206	64	11	207
**Ferritin (ng/ml)**	13-150	293.400	100	27	3.460	13.411	5
**AST (UI/L)**	<32	578	31	851	733	432	32
**IL-18 (pg/ml)**	<300	>125.000	24.470	41.399	51.510	50.000	12.370
**CXCL9 (pg/ml)**	<612	379.642	710	546	15.163	193.783	<50
**Neopterin (ng/ml)**	<2,5	11,05	2,97	4,20	16,3	8,9	0,75

### P147 Applicability of the hscore criteria in patients with macrophage activation syndrome

#### M. I. De La Cera Rodríguez, N. L. De la Rosa Encarnacion, H. F. Menchaca Aguayo, E. R. Mercedes Perez, P. P. Ramos Tiñini, R. E. Loor Chavez, S. Rodriguez Aguayo, E. Faugier Fuentes

##### Paediatric Rheumatology, Hospital Infantiil de México Federico Gómez, Mexico, Mexico

###### **Correspondence:** M. I. De La Cera Rodríguez

**Introduction:** Macrophage activation syndrome (MAS) is a secondary form of hemophagocytic lymphohistiocytosis (HLH), a potentially fatal complication of rheumatic diseases. This usually occurs in the context of systemic juvenile idiopathic arthritis (JIAs), it can also occur, although more rarely in systemic lupus erythematosus (SLE) and Kawasaki disease.

The HScore (hemophagocytic syndrome diagnostic score) was created to timely identify secondary HLH in adults. With a total of nine variables, where 3 are clinical, 5 biological and 1 cytological, each of them is given a specific score, in which a sum of 203-257 points with a mean of 230 points determines the posibility for the diagnosis of secondary hemophagocytic syndrome and a score of 125 confers negativity for it. The HScore has not yet been validated in the pediatric population, however it has been shown that a higher score cutoff >141 points provides greater sensitivity and specificity in children.

**Objectives:** The application of the HScore criteria in pediatric population with diagnosis of Macrophage Activation Syndrome

**Methods:** Retrospective, descriptive cross study

**Results:** A total of 17 patients were included by review of the physical and electronic medical record in a period of five years from 2016 to 2021, all of them aged between 0 to 18 years, with a distribution by female gender of 76.4% and male 23.6%. 100% of the patients have a diagnosis of underlying rheumatological disease. The main condition being Systemic Juvenile Idiopathic Arthritis (JIAs) with a total of 11 patients and Systemic Lupus Erythematosus (SLE) 6 patients.

Regarding the complete hematic biometry, the presence of cytopenias is describen in 1 line in two patients (11.7%), two cell lines in eight patients (47%) and three cell lines in seven patients (41.3%). A finding of hemophagocytes in bone marrow was evidenced in 12 patients (70.5%) The JIAs an SLE groups are compared by analyzing each of the criteria individually, finding statistical significance in the cytopenia criterion, with 100% of the patients with SLE presenting affection of three cell lines (p 0.001) codition explained by the pathophysiological bases of each of the diseases described above.

The score was given to each variable determined by de HScore with a mean of 272.9, median of 271 an mode of 297 points respectively. Concluding that it is possible to diagnose MAS with HScore variables in 100% of the studied patients.

When comparing the JIAs and SLE groups, no statistical significance is found in the final absolute value of the HScore, with which it is possible to conclude that the Hscore has no variation in terms of the associated base pathology, establishing then, that obtaining the necessary score allows to do diagnose of Macrophage Activation Syndrome and therefore its application is possible in the face of the different associated comorbidities.

**Conclusion:** The high mortality rate of these conditions highlights the importance of early and timely recognition to establish treatment strategies. This being the main reason why it seeks to establish new criteria that facilitate its application and that in turn have high sensitivity an specificity for the diagnosis. The objective of applying these criteria is to simplyfy laboratory tools using more widely available markers.

Considering that at this time, the HScore criteria have not yet been validated in the pediatric population, however, the study and application of them in our population establishes the possibility of diagnosing Macrophage Activation Syndrome from the initial or early stages, a situatioin that allows to implement early and aggressive treatment of the underlying disease.

**Patient Consent Received**

No

**Disclosure of Interest**

None declared

### P148 Traditional laboratory parameters and new biomarkers in macrophage activation syndrome (MAS) and secondary hemophagocytic lymphohistiocytosis (SHLH)

#### A. De Matteis^1^, D. Pires Marafon^1^, I. Caiello^1^, M. Pardeo^1^, G. Marucci^1^, E. Sacco^1^, F. Minoia^2^, F. Licciardi^3^, A. Miniaci^4^, I. Maccora^5^, C. Alizzi^6^, G. Prencipe^1^, F. De Benedetti^1^, C. Bracaglia^1^

##### ^1^Division of Rheumatology, IRCCS Ospedale Pediatrico Bambino Gesù, Rome; ^2^Pediatric Rheumatology, Fondazione IRCCS Ca’ Grande Ospedale Maggiore Policlinico, Milan; ^3^Department of Pediatrics and Infectious Diseases, School of Medicine, University of Turin, Regina Margherita Children’s Hospital, Turin; ^4^Department of Pediatrics, University of Bologna, S. Orsola-Malpighi Hospital, Bologna; ^5^Pediatric Rheumatology Unit, Meyer Children's University Hospital, Florence; ^6^University Department Pro.Sa.M.I. “G. D’Alessandro”, University of Palermo, Palermo, Italy

###### **Correspondence:** A. De Matteis

**Introduction:** Macrophage Activation Syndrome (MAS) and Secondary Hemophagocytic Lymphohistiocytosis (sHLH) are cytokine storm syndromes, in which IFNγ plays a pivotal role.

**Objectives:** Routine laboratory parameters of disease activity and severity were collected from 103 patients, 41 sHLH, 40 MAS in the context of sJIA, and 22 sJIA without MAS, from 6 Italian centers.

**Methods:** The samples were collected at three different time points: active disease (T0), 7-10 days from starting therapy (T1) and in clinical inactive disease on medication (from 1 to 3 months from onset) (T2). Serum levels of the IFN-γ related biomarkers (CXCL9, CXCL10, Neopterin) and IL-18 were measured by ELISA.

**Results:** 367 samples were collected. Laboratory characteristics at T0 are detailed in table. Using the 2016 classification criteria for MAS, we can confirm that platelet count is a specific parameter, only 4 patients with sJIA had a value <181x10^9^/liter; while ferritin is a sensitive parameter, 94.2% of patients with MAS had ferritin >684 ng/ml. We have found that lactate dehydrogenase (LDH) values were statistically higher in MAS and sHLH compared to sJIA. ROC curve of LDH values in MAS showed a statistically significant area under the curve (AUC= 0.78, p-value <0.0001). A cut-off of 681 U/L had a sensitivity of 72.6% and a specificity of 69.2%. CXCL9, CXCL10, neopterin and IL-18 values in T0 were significantly higher in MAS and sHLH patients compared to sJIA. IL-18 in MAS was significantly high than in sHLH (p<0.0001). The ROC curves performed for each biomarker showed a statistically significant AUCs (p<0.01), except for IL-18 in sHLH. We identified a cut off value for each biomarker in MAS (CXCL9 900 pg/ml, CXCL10 280 pg/ml, Neopterin 6.0 ng/ml, IL-18 78000 pg/ml) and sHLH (CXCL9 1900 pg/ml, CXCL10 270 pg/ml, Neopterin 8.0 ng/ml). CXCL9, CXCL10, neopterin and IL-18 levels lowered progressively at T1 with a normalization in T2. CXCL9 decreased faster compared to neopterin, similarly to the decrease of routine laboratory parameters.

**Conclusion:** Platelet count and ferritin have respectively high specificity and sensitivity to diagnose MAS in the context of sJIA. Even if LDH is not included in 2016 classification criteria for MAS in sJIA, we have found that this parameter could help to discriminate MAS in sJIA, in addition to the others. Moreover, our results confirm that the IFN-γ related biomarkers and IL-18 are significantly high in patients with MAS and sHLH and might be useful for diagnosis in addition to the traditional laboratory parameters. IL-18 could be also useful to distinguish sHLH from MAS and MAS from active sJIA.

**Disclosure of Interest**

A. De Matteis: None declared, D. Pires Marafon: None declared, I. Caiello: None declared, M. Pardeo: None declared, G. Marucci: None declared, E. Sacco: None declared, F. Minoia: None declared, F. Licciardi: None declared, A. Miniaci: None declared, I. Maccora: None declared, C. Alizzi: None declared, G. Prencipe: None declared, F. De Benedetti Consultant for: Abbvie, SOBI, Novimmune, Novartis, Roche, Pfizer, Employee of: SOBI, C. Bracaglia Consultant for: SOBI and Novartis

**Table 1 (abstract P148).** Laboratory parameters in T0. Values are shown as median (IQR); p-value: Mann-Whitney U test

	MAS (N=52)	sHLH (N=48)	sJIA (N=40)	MAS vs sHLH	MAS vs sJIA	sHLH vs sJIA
Ferritin (ng/ml)	4593 (1770-10842)	4098 (2075-16867)	728 (335-2786)	0.72	**<0.0001**	**<0.0001**
Platelet count (x10^9/l)	200 (114-310)	97 (47-184)	449 (275-559)	**0.0003**	**<0.0001**	**<0.0001**
AST (U/L)	86 (51-149)	150 (51-340)	33 (23-46)	0.07	**<0.0001**	**<0.0001**
Triglycerides (mg/dl)	188 (148-286)	226 (166-381)	108 (78-140)	0.11	**<0.0001**	**<0.0001**
Fibrinogen (mg/dl)	330 (225-437)	228 (137-331)	565 (441-691)	**0.0018**	**<0.0001**	**<0.0001**
LDH (U/L)	975 (666-1446)	1255 (701-2748)	599 (407-724)	0.11	**<0.0001**	**<0.0001**
CXCL9 (pg/ml)	1728 (798-11507)	4159 (1880-10016)	300 (300-1989)	0.15	**0.0010**	**<0.0001**
Neopterin (ng/ml)	9.4 (4.9-16.0)	20.3 (9.6-35.0)	4.3 (3.0-7.4)	**0.0023**	**<0.0001**	**<0.0001**
IL-18 (pg/ml)	159833 (50000-250000)	13640 (2230-83909)	36764 (8958-82714)	**<0.0001**	**<0.0001**	0.16

### P149 Central nervous system involvement as a predictor of early death in children with macrophage activation syndrome and infection-associated hemophagocytic syndrome

#### S. Harnchoowong, S. Vilaiyuk, S. Pakakasama, B. Lerkvaleekul, S. Soponkanaporn

##### Pediatrics, Ramathibodi Hospital, Mahidol University, Bangkok, Thailand

###### **Correspondence:** S. Harnchoowong

**Introduction:** Macrophage activation syndrome (MAS) and infection-associated hemophagocytic syndrome (IAHS) are life-threatening conditions and high mortality rate. Therefore, it is essential to identify the characteristics of patients and factors that affected outcomes.

**Objectives:** To evaluate outcomes and identify predictors of treatment outcomes in children with MAS and IAHS.

**Methods:** Fifty-nine pediatric patients diagnosed with MAS (n=21) and IAHS (n=38) were enrolled between January 2004 and December 2019 in Ramathibodi Hospital. We retrospectively reviewed medical records including, clinical and laboratory data, disease information, and parameters related to outcome. Treatment outcomes were classified as “early death” (death within 30 days after diagnosis) and “early treatment response” (resolution of clinical manifestations and some laboratory results within 4 weeks). Differences between characteristics of MAS and IAHS patients were compared. Logistic regression analysis was performed to identify predictors of the treatment outcomes.

**Results:** The age of patients at diagnosis of MAS was significantly older than IAHS (median [IQR]; 11.1 [6.7-11.8] vs. 4.4 [1.7-10] years, *p*=0.004). Clinical manifestations were not significantly different between MAS and IAHS except for splenomegaly which was less common in MAS (47.6% vs. 78.9%, *p*=0.014). The underlying diseases in MAS were systemic juvenile idiopathic arthritis (66.7%) and systemic lupus erythematosus (33.3%). Viral infections were the most common etiology in IAHS (44.7% Epstein-Barr virus, 23.7% Dengue, and 13.2% cytomegalovirus). The laboratory data were significantly different between MAS and IAHS as follows: the hemoglobin levels (9.8 [8.9-10.9] vs. 8.5 [7.8-9.8] g/dL, *p*=0.039), neutrophil counts (2,464 [1,447-6,490] vs. 1,176 [213-2,790]/mm^3^, *p*=0.011), platelet counts (89 [46.5-150] vs. 45 [23.8-87.3] x10^6^/mm^3^, *p*=0.012), total bilirubin levels (0.7 [0.3-1.8] vs. 1.8 [1.1-4.5] mg/dL, *p*=0.002), and direct bilirubin levels (0.4 [0.1-1.3] vs. 1.3 [0.4-3.3] mg/dL, *p*=0.009). The overall mortality rate was 28.8% (17/59), and 52.9% (9/17) of these were early death. There was no significant difference in early death rate and early treatment response between the two groups. For the predictors of treatment outcomes, the predictors of early death in univariate analysis were central nervous system (CNS) involvement (OR 15.9 [95%CI 2.8-89.9], *p*=0.002), baseline platelet counts <44x10^6^ (OR 9 [95%CI 1.7-48.7], *p*=0.011), partial thromboplastin time >40 seconds (OR 6.1 [95%CI 1.3-27.8], *p*=0.02), albumin level <23 g/L (OR 5.7 [95%CI 1.2-26.1], *p*=0.025), total bilirubin level >1.8 mg/dL (OR 6.8 [95%CI 1.3-36.3], *p*=0.025) and ferritin decline <35% from baseline during 1 week (OR 11.1 [95%CI 1.8-69.3], *p*=0.01). In multivariate analysis, CNS involvement was the only predictor of early death with an odds ratio of 15.8 (95%CI 1.6-156.7, *p*=0.018). As for predictors of early treatment response, no CNS involvement (OR 22.7 [95%CI 2.4-212.4], *p*=0.006) and platelet counts ≥47x10^6^/mm^3^ (OR 23.6 [95%CI 2.6-217.9], *p*=0.005) were significant factors in multivariate analysis.

**Conclusion:** CNS involvement was related to early death in children with MAS and IAHS. Patients without CNS involvement or patients who had initial platelet count more than 47,000/mm^3^ had a higher chance of early treatment response.

**Patient Consent Received**

No

**Disclosure of Interest**

None declared

### P150 Peripheral blood gene expression analysis in systemic juvenile idiopathic arthritis and macrophage activation syndrome reveals dominant innate immunity but no interferon-gamma signature

#### C. Hinze^1^, M. Saers^1^, C. Kessel^1^, F. De Benedetti^2^, D. Föll^1^, C. Bracaglia^2^

##### ^1^Department of Pediatric Rheumatology and Immunology, University Hospital Münster, Münster, Germany; ^2^Division of Rheumatology, Ospedale Pediatrico Bambino Gesù, Rome, Italy

###### **Correspondence:** C. Hinze

**Introduction:** Systemic juvenile idiopathic arthritis (SJIA) is a chronic, severe inflammatory condition that may be complicated by life-threatening macrophage activation syndrome (MAS) which is driven by activation of the interleukin (IL)-18-interferon (IFN)γ-axis. Thus, in MAS, high circulating levels of IL-18 and CXCL9 proteins are observed.

**Objectives:** To evaluate the pattern of peripheral blood innate immunity-driven gene expression signatures in a cohort of patients with SJIA in different disease states.

**Methods:** Whole-blood-derived RNA from 54 samples from 35 patients (median age at first sample 6.4 years) from a single center was analyzed using a 24 gene custom NanoString panel, including IL-1β-/NFκB-, type 1 IFN and IFNγ-driven genes (AIM2, CCL20, IL1A, IL1B, IL1RN, IL6, IL-18, NLRC4, S100A8, S100A9, S100A12, PTX3, TNFAIP3, IFI27, IFI44L, IFIT1, ISG15, RSAD2, SIGLEC1, CIITA, CXCL9, CXCL10, IDO, MRC1). Four different disease states were considered (12 clinically inactive disease [CID], 21 active disease [AD], 9 MAS [as defined by ACR/EULAR], and 12 pre-MAS, i.e. prior to the development of full-blown MAS) as well as data from 15 pediatric healthy controls (HC). Normalized NanoString counts were used for comparisons. Groups were compared using non-parametric statistics. Correlation analyses and hierarchical clustering analyses were performed. Gene expression signatures were derived by using the geometric mean of normalized NanoString counts of the respective gene sets.

**Results:** On an individual gene level, there were substantial differences in expression between HC and AD, pre-MAS and MAS samples for many genes, with highest expression levels in MAS samples. In contrast, for CIITA, lower expression levels were seen in these disease states, compared to healthy controls. Conspicuously absent were significant differences in CXCL9 expression between disease states. Using correlation analyses across different disease states and using hierarchical clustering analysis, distinct gene expression signatures were identified, which we termed type 1 IFN, IFNγ and innate immunity signatures. While there was a probable gradient of expression levels, with a more prominent type 1 IFN signature in MAS > pre-MAS > AD > CID > HC, and a consistently elevated innate immunity signature in AD, pre-MAS and MAS, differences were not seen for an IFNγ signature (Table).

**Table:** 

	HC	CID	AD	Pre-MAS	MAS	p-value*
Type 1 IFN score†, median (IQR)	424 (291-792)	742 (570-1401)	1014 (223-3036)	3295 (362-8490)	4966 (1487-13300)	0.02
IFNγ score‡, median (IQR)	81 (55-111)	95 (70-136)	107 (62-168)	115 (60-233)	114 (63-327)	0.54
Innate immunity score‖, median (IQR)	332 (264-415)	565 (501-650)	1367 (718-2712)	1187 (703-2520)	1260 (625-1879)	<0.0001

*Kruskal-Wallis test

†Geometric mean of IFI27, IFI44L, IFIT1, ISG15, RSAD2, SIGLEC1 normalized NanoString counts

‡Geometric mean of CIITA, CXCL9, CXCL10, IDO normalized NanoString counts

‖Geometric mean of AIM2, CCL20, IL18, IL1RA, IL1A, IL1B, IL6, NLRC4, PTX3, S100A12, S100A8, S100A9, and TNFAIP3 normalized NanoString counts

**Conclusion:** In patients with SJIA in different disease states, a marked dysregulation of peripheral blood gene expression is seen in multiple innate immunity-related genes, including IL-1β/NFκB- and type 1 IFN-related genes, most prominently in AD, pre-MAS and MAS. In light of the suggested dominance of IFNγ-driven pathology in MAS, the absence of an IFNγ signature in the context of high CXCL9 serum levels and points to cellular IFNγ and CXCL9 sources outside of peripheral blood.

**Disclosure of Interest**

None declared

### P151 Lysinuric protein intolerance mimicking lupus presenting as macrophage activation syndrome

#### B. Kasap-Demir, A. Kanık, M. Köse, M. Baran

##### Izmir Katip Çelebi University, Izmir, Turkey

###### **Correspondence:** B. Kasap-Demir

**Introduction:** Macrophage activation syndrome is a rare but potentially fatal complication seen in autoimmune rheumatic diseases, characterized by cytokine storm. In some cases, it may appear as the first sign of the disease, and in others it may be observed during follow-up. Differential diagnosis in terms of underlying disease should be made carefully.

**Objectives:** Here, we aimed to report a case with a metabolic disease presenting as lupus associated macrophage activation syndrome.

**Methods:** A 16-year-old male patient was brought to our hospital with complaints of high fever that started 6 days before admission, darkening of urine color for 3 days, and yellowish skin. His past medical history was remarkable for IVIg usage between 2 and 3 years of age for suspected infantile transient hypogammaglobulinema. Aortic root dilatation was foud at the age of 4. His had growth retardation and he was diagnosed with hypopituitarism and osteoporosis, and used somatostatin at the age of 7. He was being followed up for hepatosplenomegaly and high ferritin levels (500-750) thereafter without any specific diagnosis. On admission, he was icteric, dehydrated and had malnutrition. Body weight was 28 kg (<3p, -5.44 SDS), height was 160 cm (<3p, -1.81 SDS), heart rate was 128/min, and respiratory rate was 24/min, body temperature was 39^0^C, blood pressure was 114/77 mmHg. The liver and spleen were palpable 4 cm below the ribs. The abnormal laboratory tests were as follows: Hb: 4.1 gr/dL, WBC: 10.5/mm^3^, platelets: 386.000/mm^3^, AST: 197 IU/L, LDH: 7430 U/L, Ferritin: 6430 ng/ml, triglyceride: 246 mg/dL, D.Coombs: 4(+), reticulocyte: 8.8% and schystocytes were prominent in blood smear. All other parameters were in normal limits. Bone marrow examination revealed intense hemophagocytosis. In rheumatological examinations, anti-dsDNA: 93.85 (n<100), ANA: 1/640, anti-Ro52: ++, ant-histon antibodies: ++, C3: 38.4 mg/dL (N:79-152), C4: 8.6 (N: 16-38), antibodies against cardiolipin, and beta 2 glycoprotein 1 were negative. SLE presenting with macrophage activation syndrome was considered. Erythrocyte transfusion was performed, 30 mg/kg/day pulse methylprednisolone and IVIG therapy was initiated and cyclospoirne was added. Proteinuria was observed and in the kidney biopsy performed in the follow up revealed focal mesangial proliferation in some glomeruli and IgM(++), IgA(+/-), C3(+), which was not compatible with lupus nephritis. No other clinical findings related to SLE were detected in the follow-up, however, LDH and ferritin levels increased intermittantly that were responsive to high dose steroids. Meanwhile, the screening tests that have been sent for hepatosplenomegaly, revealed a homozygous mutation for late onset glycogen storage disease type 4. Since the clinical course of the patient could not be explained with lupus or glycogen storage disease type 4, whole exome sequencing was sent.

**Results:** The result was suggestive of a homozygous deletion on chromosome 14 spanning several exons of *SLC7A7* compatible with lysinuric protein intolerance that may explain the MAS and intermittant hyperferritinemia in our patient. The patient's diet was regulated and he has no MAS flare in the following 18 months.

**Conclusion:** Lysinuric protein intolerance should be considered in a patient presenting with MAS in the presence of severe growth retardation, osteoporosis, organomegaly, and high inflammatory markers. It may mimic the laboratory findings of lupus as well, which may cause a diagnostic challenge.

**Patient Consent Received**

Yes

**Disclosure of Interest**

None declared

### P152 Primary and secondary hemophagocytic lymphohistiocytosis (HLH) in pediatric intensive care: clinical characteristics, therapies and outcomes

#### M. Murciano^1^, G. Bottari^1^, D. Pires Marafon^2^, F. De Luca^3^, F. Chiusolo^4^, P. Merli^5^, C. Cecchetti^1^, F. De Benedetti^2^, M. Di Nardo^1^, C. Bracaglia^2^

##### ^1^Department of Emergency and Pediatric Intensive Care; ^2^Division of Rheumatology, IRCCS Ospedale Pediatrico Bambino Gesù (OPBG), Roma, Italy; ^3^Pediatric Clinic, Policlinico Umberto I, Sapienza University of Rome; ^4^Pediatric Intensive Care Unit; ^5^Department of Onchohematology, IRCCS Ospedale Pediatrico Bambino Gesù (OPBG), Roma, Italy, Rome, Italy

###### **Correspondence:** M. Murciano

**Introduction:** HLH is a severe, life threatening disease that can develop into multiple organ failure (MOF) and death in a still high percentage of cases. Some patients require intensive care assistance with advanced organ support techniques. Primary HLH (pHLH) is usually caused by mutation in genes involved in the cytolytic function while secondary HLH (sHLH) can be triggered by various agents, including rheumatological diseases. The latter form is also called macrophage activation syndrome (MAS). Nevertheless, in a high percentage of cases no trigger has found.

**Objectives:** To evaluate clinical characteristics, therapies and outcomes of a cohort of pediatric patients with pHLH and sHLH admitted in pediatric intensive care unit (PICU) of Ospedale Pediatrico Bambino Gesù.

**Methods:** Data of 48 patients with HLH who required admission in PICU from 2007 till 2019 were collected. Clinical features, laboratory parameters and supportive therapy were evaluated at two time points: at PICU admission (T1) and at the worst time during PICU admission, defined by the highest ferritin value (T2). At T1 the risk of mortality was also assessed by the Pediatric Index of Mortality (PIM2 and PIM3).

**Results:** Of the 48 patients, 26 males, with disease onset mean age of 6.3 years (range 0 to 17.5), 5 had pHLH, 37 sHLH and 6 MAS in the context of sJIA. Twenty-three (48%) out of 48 patients died: 4 pHLH (80%), 18 sHLH (48%) and 1 MAS (16%). Splenomegaly at onset was significantly more frequent in patients who died compared to those who survived (78.3% vs 40%; ***p*=0.007**), as well as the incidence of MOF (43.5% vs 28%; *p*=0.26) and acute renal failure (47.8% vs 36%; *p*=0.41). White blood counts and neutrophil counts were significantly lower in T1 in patients who died compared to those who survived (***p*=0.033** and ***p*=0.017**), while platelets count was significantly lower in patients who died only in T2 (***p*=0.0087**). Ferritin levels were higher in patients who died both in T1 and T2 but the difference was not significant. ExtraCorporeal Membrane Oxygenation (ECMO) was used equally in the two groups of patients but for a longer period of time in those who died (Table 1). As expected, the PIM2 and PIM3 were higher in patients who died but the difference was not significant.

**Conclusion:** Our data confirm that patients with MAS have a lower mortality rate compared to the other forms of HLH. Splenomegaly at onset, platelets count and ferritin levels at time of PICU admission seems to identify patients with the worst prognosis. Extracorporeal support therapies, in particular ECMO, have been used in a homogeneous manner in all patients but for a longer period of time in the most severe cases and therefore with higher mortality. To our knowledge, this is the largest pediatric series of patients with pHLH and sHLH treated in the PICU setting.

**Patient Consent Received**

Yes

**Disclosure of Interest**

M. Murciano: None declared, G. Bottari: None declared, D. Pires Marafon: None declared, F. De Luca: None declared, F. Chiusolo: None declared, P. Merli: None declared, C. Cecchetti: None declared, F. De Benedetti Consultant for: Consultant for Abbvie, SOBI, Novimmune, Novartis, Roche, Pfizer., M. Di Nardo: None declared, C. Bracaglia Consultant for: Consultant for SOBI and Novartis.

**Table 1 (abstract P152).** Extracorporeal support therapies and outcomes of patients.

	Died(N=23)	Survivors(N=25)	p-value
Mechanical ventilation^1^	22 (95,7)	20 (80,0)	0,19^F^
Duration (days)^2^	13 (7-27)	13 (6-23)	0,67
ECMO^1^	5 (21,7)	5 (20,0)	1,0^F^
Duration (days)^2^	25 (2-69)	7 (6-7)	0,63
CRRT^1^	17 (73,9)	11 (44,0)	**0,036**^**C**^
Plasma exchange^1^	8 (34,8)	8 (32,0)	0,84^C^
CytoSorb^1^	3 (13,0)	2 (8,0)	0,66^F^
Days in PICU^2^	30 (10-45)	17 (9-30)	0,13

^1^Number (%) - Chi-square test^C^/ Fisher exact test^F^; ^2^Median (1st-3rd quartile) - Mann-Whitney test

### P153 Macrophages activating syndrome in pediatric visceral leishmaniasis: about a series of 14 cases

#### H. Nassih, R. Elqadiry, A. Bourrahouat, I. Aitsab

##### Pediatrics, Mohammed 6 University Hospital Center of Marrakesh, Marrakesh, Morocco

###### **Correspondence:** H. Nassih

**Introduction:** Macrophages activating syndrome (MAS) is one of the most common complications in pediatric visceral leishmaniasis.

**Objectives:** To describe the phenotype, management and prognosis of MAS complicating visceral leishmaniasis in children.

**Methods:** We conducted a single center retrospective descriptive study of 14 children with visceral leishmaniasis and MAS.

**Results:** The mean age at diagnosis was of 8 years, with extremes of 6 months and 14-year-old. Boys were predominant with 65%. Telltale symptoms were protracted fever and a bone marrow failure syndrome in all cases. Meanwhile, hemorrhage and deterioration of general condition was present in half of the cases. Physical examination found a splenomegaly in 64%, an hepatomegaly in 50%, anasarca in 21%, cholestasis in 14%, and arthritis in 7% of cases. Complete blood count found pancytopenia in 71% and bicytopenia in 29% of children. Laboratory work-up found high LDH levels in all cases, high ferritin level in 71%, high triglycerides level in half of cases, hepatic cytolysis in 64%, and liver failure and acute kidney injury in 28.6% of cases. CRP and ESR were elevated in all cases. Myelogram found hemophagocytic lymphohistiocytosis and Leishman-Donovan bodies in all cases. Management of MAS was based on methylprednisolone pulses at 1.73 g/m2/day for three consecutive days followed by prednisone at 2 mg/kg/day. Prednisone tapering schedule was decided case by case and according to the evolution of the clinical symptoms as well as the levels of acute phase reactants, the cytopenia and the liver enzymes. The mean steroid duration was of 17 days. As for leishmaniasis treatment, it was mostly based on meglumine antimoniate at 60 mg/kg/day by IM route and for 28 days. However, in four cases with liver failure, liposomale amphotericine B was prescribed at 3 mg/kg/day for 5 days. Evolution was marked by gain of apyrexia in a mean time of 5 days, normalization of acute phase reactants in a mean time of 8 days and normalization of hemogram in a mean time of 21 days. Unfortunately, we deplore two deaths during the first 24 hours of admission, the cause of death being acute respiratory distress syndrome and multiple organ dysfunction syndrome respectively.

**Conclusion:** Prognosis of MAS complicating visceral leishmaniasis is good when management is early. Systemic steroids are very effective in inducing remission of MAS symptoms.

**Disclosure of Interest**

None declared

### P154 When macrophage activation syndrome presents at diagnosis

#### M. J. Pereira^1^, Í. R. Oliveira^1^, A. J. Fernandes^1^, R. J. Pereira^1^, F. M. Dias^1^, M. C. Conde^2^

##### ^1^Pediatric Department, Centro Hospitalar Universitário do Algarve - Unidade de Faro, Faro; ^2^Pediatric Rheumatology Unit, Centro Hospitalar e Universitário Lisboa Central – Hospital Dona Estefânia, Lisbon, Portugal

###### **Correspondence:** M. J. Pereira

**Introduction:** Macrophage activation syndrome (MAS) is a serious, potentially life-threatening hyperinflammatory disorder that belongs to the spectrum of hemophagocytic lymphohistiocytosis (HLH) and can complicate several immunologic disorders.^1^

**Objectives:** We aim to highlight the importance of having a high-grade suspicion of MAS when systemic juvenile idiopathic arthritis (sJIA) is suspected and to describe a case of recurrent MAS.

**Methods:** We present a case report.

**Results:** A 10-year-old male with no relevant past medical or familial history, with non-consanguineous parents, was admitted for dizziness and fatigue for 3 months, polyarthralgia for 10 days, prostration, myalgia, headache, anorexia, and high fever for 5 days (2-3 spikes/day). On the 5^th^ day of fever, he started a rash, more prominent during fever spikes. On physical examination, he presented a discrete micromacular rash and a cervical adenopathy. He had markedly elevated inflammatory markers [leukocytosis, elevated C-reactive protein and erythrocyte sedimentation rate (ESR)], with ferritin > 9 000 mg/dL. Abdominal ultrasound and thoracoabdominal pelvic CT scan showed splenomegaly and multiple lymphadenopathies. He received empiric antibiotics, without clinical or inflammatory markers improvement. The main suspected diagnosis was sJIA, while neoplasic and infectious etiology were being excluded.

On the 17^th^ day, the fever pattern changed and became persistent; hepatosplenomegaly became more evident, and he developed distributive shock. Analytically, there was a decrease in hemoglobin and leukocyte count, a slight decrease in platelet count, severe hyperferritinemia (> 54 000 ug/L), elevation of aspartate transaminase, elevated triglycerides, and a decrease in ESR. The diagnosis of MAS was made and he started methylprednisolone pulses. He completed 5 pulses of methylprednisolone, maintaining high dose corticotherapy and initiated anakinra 3 mg/kg/day with marked clinical, inflammatory and MAS markers improvement.

On the 8^th^ day of anakinra, the clinical and analytical condition worsened and, despite raising anakinra to 5 mg/kg/day, a second MAS was diagnosed. Due to raised liver enzymes (> 5 higher than the upper normal limit), anakinra was suspended two days later, he repeated pulses of methylprednisolone and started IV cyclosporine with clinical and laboratory improvement allowing to restart anakinra.

One year later, he is off steroids, symptom-free, maintains therapy with anakinra, and is suspending cyclosporine. HLH, primary and acquired immunodeficiency genetic panels revealed no pathogenic mutations.

**Conclusion:** Among pediatric rheumatic diseases, MAS is most commonly seen in patients with sJIA.^1,2^ It is characterized by the acute onset of persistent high fever, pancytopenia, hyperferritinemia, hepatosplenomegaly, liver dysfunction, and coagulation abnormalities.^1,2,3,4^

Regardless of the criteria used, if sJIA is suspected, sudden worsening of the clinical condition should raise a high suspicion of MAS.^5^ If left untreated, MAS can progress to multiorgan failure and be fatal.^1,3^ In this case, rapid recognition and prompt therapy were critical to this child's survival and good outcome.

Heterozygous perforin-pathway mutations and mutations of other immune pathways that result in cytokine storm syndrome have been described in MAS patients.^3,4^ Despite a difficult-to-treat MAS, no pathogenic mutation was found in this patient.

**Patient Consent Received**

Yes

**Disclosure of Interest**

None declared

## e-Poster viewing: Imaging

### P155 Ultrasound features of ankles with clinically active disease in children with new-onset juvenile idiopathic arthritis

#### F. Chironi^1^, O. De Lucia^2^, F. Pregnolato^2^, G. Filocamo^1^, S. Orsi^3^, G. Rogani^1^, T. Giani^4^, S. Costi^5^, C. Agostoni^1,5^, R. Cimaz^2,5^, S. Lanni^1^ on behalf of PRAGMA (Pediatric Rheumatology Associated Group of the Milan Area)

##### ^1^Paediatrics, Fondazione IRCCS Ca' Granda Ospedale Maggiore Policlinico di Milano; ^2^Rheumatology, ASST Centro Traumatologico Ortopedico G. Pini-CTO, Milano; ^3^Istituto Giannina Gaslini, Genova; ^4^Medical Biotechnology, University of Siena, Siena; ^5^Clinical Sciences and Community Health, University of Milano, Milano, Italy

###### **Correspondence:** F. Chironi

**Introduction:** The ankle is one of the most commonly affected sites in juvenile idiopathic arthritis (JIA). This region has a complex anatomical structure owing to the presence of multiple joint recesses and surrounding tendons. Ultrasound (US) is a useful tool to implement clinical examination, since this imaging modality allows to localize precisely the inflamed joints and tendons which are often difficult to distinguish clinically, especially in young children.

**Objectives:** To investigate US features of ankles with clinical disease activity in patients with JIA at disease onset.

**Methods:** The clinical charts of all consecutive patients with new-onset JIA between May 2018 and January 2020 at study centres (Policlinico and G. Pini Hospitals of Milan) and with clinically active ankle disease among the joints affected were reviewed retrospectively. Data on ankle US assessment were retrieved. Detailed information about joints and tendon compartments affected on US examination were then recorded.

**Results:** Forty-four ankles of 32 patients with new-onset JIA (12 boys and 20 girls) were included in the study. US showed inflammation in 34 (77.3%) tibiotalar (TT), 18 (40.9%) subtalar (ST), and 23 (52.3%) intertarsal (IT) joints. The TT was the only affected joint compartment in 9 (20.5%) ankles, whereas the ST and IT joints resulted inflamed without any other joint compartment affected on US in 1 (2.3%) and 5 (11.4%) ankles, respectively. Eight (18.2%) ankles displayed on US concomitant involvement of TT, ST and IT joints. Concerning tendons, US showed inflammation in 21 (47.7%) medial tendon (MT), 20 (45.5%) lateral tendon (LT), and 9 (20.5%) anterior tendon (AT) compartments. The MT compartment was the only site of tendon pathology in 6 (13.6%) ankles. The LT and the AT compartments were affected on US without concomitant involvement of other tendon compartments in 5 (11.4%) and 1 (2.3%) ankles, respectively. The MT, LT, and AT compartments were together affected on US in 4 (9.1%) ankles. Three ankles (6.8%) had isolated tenosynovitis, whereas 15 (34.1%) ankles had arthritis without concomitant tenosynovitis.

**Conclusion:** The TT joint and the MT compartment are the sites most commonly affected on US in ankles with clinical disease activity in patients with new-onset JIA. The majority of ankles with clinical disease activity at disease onset show on US inflammation in multiple joint and tendon compartments. Isolated arthritis is more common than tenosynovitis alone. The findings of the study highlight the utility of US in identifying precisely the location of pathology and in managing local injection therapy.

**Patient Consent Received**

Yes

**Disclosure of Interest**

None declared

### P156 Self-reported acceptability of whole-body MRI in young people

#### V. Choida^1,2^, C. Ciurtin^2^, T. J. Bray^1^, D. Sen^2^, C. Fisher^2^, M. Leandro^2^, M. Hall-Craggs^1^

##### ^1^Centre for Medical Imaging; ^2^Centre for Adolescent Rheumatology Versus Arthritis, University College London, London, United Kingdom

###### **Correspondence:** V. Choida

**Introduction:** Magnetic resonance imaging (MRI) can detect musculoskeletal inflammation in patients with juvenile idiopathic arthritis (JIA). Whole-body MRI (WBMRI) is a promising imaging technique as it enables us to assess multiple joints in one examination. However, less is known about young people’s experience of undergoing MRI scans and their willingness to have these tests.

**Objectives:** To describe the experience of young people who underwent a WBMRI scan for research purpose, designed to assess joints for inflammation, and their willingness to have this investigation again in the future.

**Methods:** Sixty patients, 47 JIA and 13 controls with non-inflammatory musculoskeletal pain, aged 14-24 were recruited in a tertiary hospital for a prospective study which measured the prevalence of subclinical synovitis on WBMRI. The scan was performed with gadolinium in a 3 Tesla scanner and lasted 45-60 minutes. All participants completed an anonymised questionnaire, immediately after the MRI examination. The age, gender and presence or absence of JIA diagnosis were recorded. Five-point smiley face Likert scales were used to rate anxiety and pain levels during the scan. The likelihood of agreeing to undergo a repeat WBMRI scan for clinical reasons in 6 months’ time was recorded as definitely not, not likely, likely, or definitely. The mean ± standard deviation was calculated for age, and absolute and relative frequencies (%) were calculated for ordinal data. The chi-square test was used to compare ratings and t-test for the comparison of numerical data between groups.

**Results:** Forty female and 20 male participants completed the survey. Two male patients with JIA did not complete the second page of the questionnaire, which included the question of having a repeat scan. The mean age of JIA patients and controls in years was 18.3 ± 2.4 and 16.5 ±1.2 (p=0.012), respectively. The pain and anxiety ratings in young people with JIA and controls are shown in the Table. A higher proportion of JIA patients rated their anxiety levels as ‘okay’ or ‘totally fine’ compared to controls (83% vs 54%, p=0.028). Male patients reported more frequently that they felt ‘totally fine’ in terms of anxiety, compared to female patients (65% vs 28%, p=0.005). There were no significant differences in the pain levels experienced during the scan between males and females, or between JIA patients and controls. Almost all patients (57/58, 98%) answered that they were ‘likely’ (24/58, 41%) or would ‘definitely’ agree (33/58, 57%) to have a repeat scan in 6 months if advised by their doctor.

**Conclusion:** The majority of young people, predominantly patients with JIA, who underwent a research WBMRI scan for the assessment of synovitis, reported either mild or absent symptoms of anxiety and pain during the scan. Almost all patients would likely or definitely be agreeable to undergo a repeat scan in 6 months, for clinical reasons. In conclusion, WBMRI appears to be an acceptable imaging technique amongst young people who received this test.

**Disclosure of Interest**

None declared

**Table 1 (abstract P156).** Frequency of reported pain and anxiety levels by young people undergoing a whole-body MRI, number of respondents (%)

**Pain**	**Respondents**	**Very severe pain**	**Severe pain**	**Moderate pain**	**Mild pain**	**No pain**
	**JIA (n=47)**	0	1 (2.1)	9 (19.2)	16 (34)	21 (44.7)
	**Controls (n=13)**	0	1 (7.7)	1 (7.7)	5 (38.5)	6 (46.2)
	**All (n=60)**	0	2 (3.3)	10 (16.7)	21 (35)	27 (45)
**Anxiety**	**Respondents**	**Totally overwhelmed**	**Stressed**	**A bit tense**	**Okay**	**Totally fine**
	**JIA (n=47)**	0	1 (2.1)	7 (14.9)	19 (40.4)	20 (42.6)
	**Controls (n=13)**	0	2 (15.4)	4 (30.8)	3 (23.1)	4 (30.8)
	**All (n=60)**	0	3 (5)	11 (18.3)	22 (36.7)	24 (40)

### P157 Application of computerized color telethermography in 225 children and adolescents with Raynaud's phenomenon

#### N. Skreb^1^, M. Held^1^, M. Sestan^1^, N. Kifer^1^, D. Turudic^1^, M. Frkovic^1^, J. Stipic^2^, M. Jelusic^1^

##### ^1^University Hospital Centre Zagreb, Department of Pediatrics, University of Zagreb School of Medicine; ^2^University Hospital Centre Zagreb, Department of Neurology, Zagreb, Croatia

###### **Correspondence:** M. Held

**Introduction:** Computerized color telethermography (CCTT) is an established diagnostic procedure in clinical practice that is used for assessment and follow-up of patients with microcirculatory disorders in adult population, such as Raynaud's phenomenon (RP). However, CCTT is still not validated in pediatric patients with RP.

**Objectives:** To analyze the CCTT findings in children and adolescents with clinically suspicious RP and its relationship with age, gender and season as well as with biochemical and immunological laboratory findings.

**Methods:** Retrospective study included pediatric patients with suspected RP who underwented CCTT diagnostic procedure at the University Hospital Centre Zagreb from 2010 to 2019. Laboratory findings included inflammatory parameters, complete blood count, renal function tests and immunological tests (ANA, ENA screen, ANCA, RF, antiphospholipid antibodies, serum IgG and complement levels). Differences between categorical variables were examined using Chi-Square test and among numerical using t-test, followed by logistical regression analysis.

**Results:** Out of 225 patients with suspected RP, 176 were females (78.2%) and 49 were males (21.7%) giving a female to male ratio bigger than 3:1. In 44 patients (19,6%) CCTT was compatible with the diagnosis of primary RP, 68 patients (30,2%) were classified as secondary RP, whereas 27 patients (12%) had CCTT results which where considered unspecific. In 86 patients (38,2%) CCTT findings were normal. Among the patients classificated as secondary RP using CCTT, the most of them, 28 (41.2%), were diagnosed with juvenile idiopathic arthritis, while 26 (38.2%) had no evident other disease. The median (range) age at the time of performing CCTT was 15.16 (13.50-16.75) years. According to their age, patients were divided into 3 groups: childhood (3-10 years), early adolescence (11-14 years) and late adolescence (15-18 years). The most of them were in late adolescence group (53,8%) and these patients were 2.4 times more likely to be diagnosed with primary RP (OR 0.41, CI 0.18-0.92, p=0.03 for early adolescence group). Seasonal influence was a statistically significant factor in CCTT confirmation of the primary RP, since the largest number of patients with primary RP were diagnosed during the winter. If CCTT was performed in the winter, there was 7.25 times higher chance to confirm the primary RP with CCTT compared to the spring time (OR 7.25, CI 2.25-23.25, p=0.03). However, such influence was not observed for secondary RP. Patients diagnosed as secondary RP on CCTT had statistically significantly lower leukocyte (p=0.03) and platelet count (p=0.04), as well as C3 levels (p=0.008), but higher creatinine levels (p=0.008) in comparison with patients with normal CCTT findings. Concerning the immunlogical findings, it was shown that females, regardless of age, with positive ENA screen had 4 times higher risk to be diagnosed with secondary RP (OR 0.25, CI 0.06-0.95, p=0.04 for females with negative ENA).

**Conclusion:** Patients with secondary RP in whom the underlying systemic disease had not yet manifested had the greatest benefit from CCTT. In these patients, no other diagnostic method can replace CCTT. In patients with suspected primary RP, it is best to perform CCTT in the winter months. We observed that female adolescents were referred to CCTT more often and that regardless of age females with positive ENA screen were more likely to be diagnosed as secondary RP on CCTT.

**Patient Consent Received**

No

**Disclosure of Interest**

None declared

### P158 Procedures for the conduct, content and format of eular/pres pediatric musculoskeletal ultrasound courses

#### V. Muratore^1^, E. Naredo^2^, J. Vojinovic^3^, M. A. D'Agostino^4^, S. Magni Manzoni^5^

##### ^1^Dipartimento Cure Primarie, ATS Pavia, Pavia, Italy; ^2^Department of Rheumatology, Joint and Bone Research Unit, Hospital Universitario Fundación Jiménez Díaz, Madrid, Spain; ^3^Faculty of Medicine, University of Niš, Niš, Serbia; ^4^Rheumatology Department, Gemelli Hospital; ^5^Rheumatology Department, IRCCS Children’s Hospital Bambino Gesù, Rome, Italy

###### **Correspondence:** S. Magni Manzoni

**Introduction:** Recently several Pediatric Musculoskeletal ultrasound (PedMSUS) courses have been held. However, content, conduct and format of PedMSUS courses have never been internationally agreed.

**Objectives:** To produce practical and educational recommendations for the conduct, content and format of EULAR/PReS PedMSUS courses.

**Methods:** The project consisted of a joined EULAR/PReS effort and was conducted in two separate phases. 1.Through a systematic literature review, including extensive search on websites and networks on educational projects/events regarding PedMSUS, a list of potential items for the content, conduct and format of basic, intermediate, advanced courses and Teach-The-Theachers (TTT) PedMSUS courses, respectively, was identified. Through two Delphi processes, a panel of experts (project Taskforce) found agreement on the items to be considered for each PedMSUS level of competency. 2. Consensus on the proposed items was reached among a broader group of physicians and health care professionals with interest in pedMSUS and/or involved in previous EULAR/PReS MSUS educational events (Consensus Group). Agreement or consensus was achieved on each topic if selected by at least 75% of the participants.

**Results:** Twenty-two out of 24 (92%) Taskforce members participated to the I Delphi-round, whereas 18/22 (82%) to the II round; 45/114 (39%) members of the Consensus Group answered the consensus survey. Agreement and consensus were reached on: format of three-level education model (basic, intermediate and advanced); courses placed prior to the annual PReS and EULAR or joined EULAR/PReS congresses; distribution between theoretical and practical part of 50%-50% for basic courses and 40%-60% for intermediate courses; a maximum of 4 participants per teacher in practical sessions; models at the basic courses should be represented by healthy children, whereas they should patients for intermediate and advanced courses; courses could be attended with by Pediatric Rheumatologists, Adult Rheumatologists and Radiologists; Faculty members/tutors should fulfil prerequisites and should have successfully attended EULAR MSUS TTT courses; TTT courses should be held just prior to the EULAR congress and linked to the EULAR/PReS PedMSUS course; the theoretical part should include how to prepare and deliver educational material, how to organize a PedMSUS course and how to conduct a practical session; practical and theoretical part in TTT should respectively cover 50% of the course; a certificate of attendance and a certificate of successful competency assessment should be provided for all the levels of competency.

**Conclusion:** Shared EULAR/PReS procedures for the conduct, content and format of PedMSUS basic, intermediate, advanced and TTT courses were identified and will allow homogeneous and high-level educational events on PedMSUS under EULAR/PReS umbrella. The potential impact of the COVID-19 pandemia, that spread in the meaningwhile, could not be investigated and may deserve additional insights.

**Patient Consent Received**

Yes

**Disclosure of Interest**

None declared

### P159 Ultrasound-detected tenosynovitis in ankles with clinically active disease of children with new-onset juvenile idiopathic arthritis does not affect the chance to achieve disease remission

#### S. M. Orsi^1,2^, O. De Lucia^3^, F. Pregnolato^3^, G. Filocamo^1^, F. Chironi^1^, G. Beretta^1^, T. Giani^4^, S. Costi^5^, C. Agostoni^5^, R. Cimaz^3,5^, S. Lanni^1^ on behalf of Pediatric Rheumatology Group of the Milan Area (PRAGMA)

##### ^1^Fondazione IRCCS Policlinico di Milano, Milan; ^2^Istituto Giannina Gaslini, Genoa; ^3^ASST G. Pini-CTO, Milan; ^4^University of Siena, Siena; ^5^University of Milan, Milan, Italy

###### **Correspondence:** S. M. Orsi

**Introduction:** The ankle region has a complex anatomical structure owing to the presence of multiple joints recesses and surrounding tendons. While the predictive value of ultrasound (US)-detected arthritis for subsequent flares has been investigated, the prognostic role of tenosynovitis in juvenile idiopathic arthritis (JIA) remains still unexplored.

**Objectives:** To investigate the predictive value of US-detected tenosynovitis in ankles with clinically active disease of children with new-onset JIA.

**Methods:** The clinical charts of all patients with new-onset JIA between May 2018-January 2020 at study centres (Policlinico and G.Pini Hospitals of Milan) and with clinically active ankle disease were reviewed. Data on ankle US assessment were retrieved and patients were stratified as follows: 1) patients with detection on US of isolated arthritis in at least one of the joints of the ankle; 2) patients with detection of tenosynovitis in at least one of the tendon compartments of the ankle irrespective of the presence of concomitant arthritis. In these two categories, estimation of patients who were able to achieve clinical disease remission at 12 months since disease onset was evaluated. Reliability of US was assessed on still images by the two sonographers who performed US examinations and was calculated using kappa (k) statistics. Any discrepancies on US findings were then resolved through a discussion between the sonographers before data analysis.

**Results:** Twenty-seven new-onset JIA patients were found to have clinical involvement of the ankle among the joints affected. Nine of them (33.3%) showed on US isolated arthritis of the ankle, whereas US-detected tenosynovitis with or without arthritis was found in 18 (66.7%) patients. The percentage of patients who were able to achieve disease remission at 12-months was the same (66.7%) for patients with and without US-detected tenosynovitis in the ankle (12/18 and 6/9 patients, respectively). Seventeen patients were not treated with biologics: also in this subgroup no difference was found in the rate of clinical remission at follow-up between patients who had isolated arthritis (8/17) and patients who had tenosynovitis (9/17) with or without arthritis at US baseline assessment (5/8 and 4/9 patients in clinical remission, respectively). In patients with US-detected tenosynovitis and clinical remission at 12 months, the lateral tendon compartment (LTC) was the tendon site more frequently affected by pathology (75%). Patients with US-detected tenosynovitis that did not achieve clinical remission at follow-up had the highest frequency of tendon pathology on US in the medial tendon compartment (MTC) (83.3%). The anterior tendon compartment was the less frequently affected in all patients (33.3% both in patients with and without clinical remission at the follow-up visit). Intraobserver reliability of US was excellent for both the sonographers (k=0.92 and k=1.00). Interobserver reliability of US was moderate (k=0.65).

**Conclusion:** US-detected tenosynovitis of the ankle is common in patients with new-onset JIA with ankle clinically active disease and is more frequent than the detection on US of isolated arthritis. The MTC and LTC are the tendon compartments more commonly affected. The detection on US of tenosynovitis at disease onset in ankles with clinical disease activity does not seem to affect the chance to achieve the overall clinical disease remission compared to patients without tendon pathology but with joint disease in the ankle region.

**Disclosure of Interest**

None declared

### P160 Synovial signal intensity on static contrast-enhanced MRI for evaluation of disease activity in juvenile idiopathic arthritis – a look at the bright side of the knee

#### F. Verkuil^1^, J. M. van den Berg^1^, E. C. van Gulik^2^, A. M. Barendregt^2^, A. Nassar-Sheikh Rashid^1^, D. Schonenberg-Meinema^1^, K. M. Dolman^3^, T. W. Kuijpers^1^, M. Maas^2^, R. Hemke^2^

##### ^1^Pediatric Immunology, Rheumatology and Infectious Diseases; ^2^Radiology and Nuclear Medicine, AMSTERDAM UMC, LOCATION AMC; ^3^Pediatrics, OLVG, Amsterdam, Netherlands

###### **Correspondence:** F. Verkuil

**Introduction:** Knowledge on the role of synovial signal intensity (SI) grading on static contrast-enhanced (CE) MRI of the knee for assessment of disease activity in juvenile idiopathic arthritis (JIA) is lacking.

**Objectives:** To assess the value of synovial SI on static CE-MRI of the knee for evaluation of disease activity in children with JIA.

**Methods:** Children with clinically inactive and clinically active JIA who underwent static CE-MRI of the knee were included. Synovial SI was evaluated on post-contrast T1-weighted fat-saturated images using a 0.02 cm^2^ region of interest drawn in the area of the synovium that contained visually the highest SI. To control for time-dependent post-contrast enhancement variability, a ratio between the SI of the synovium to the musculus gastrocnemius was calculated.

**Results:** We included 427 JIA patients (clinically inactive JIA: 150 [35,1%]; clinically active JIA: 277 [64.9%]), 65.3% female, with a mean age of 13.3 ±3.2 years. Mean SI synovium-to-muscle ratio was 2.1 ±0.7 in patients with clinically inactive JIA versus 2.2 ±0.8 in patients with clinically active JIA. Subgroup analysis showed no significant difference in SI synovium-to-muscle ratio between JIA patients with clinically inactive disease and JIA patients with clinically active disease (p-value 0.22).

**Conclusion:** Evaluation of the brightness of the synovium on static CE-MRI of the knee for assessment of JIA disease activity should be avoided, as this might lead to incorrect clinical conclusions.

**Disclosure of Interest**

None declared

### P161 Ultrasound in the initial diagnostic workup of paediatric large vessel vasculitis

#### D. Windschall^1,2^, C. Hinze^3^, T. Schwarz^1^, F. Gohar^1^

##### ^1^Clinic for Paediatric and Adolescent Rheumatology, St. Josef-Stift Hospital, Northwest German Center for Rheumatology, Sendenhorst; ^2^University Halle-Wittenberg, Halle; ^3^Clinic for Paediatric Rheumatology and Immunology, University Hospital Münster, Muenster, Germany

###### **Correspondence:** D. Windschall

**Introduction:** Prompt identification and diagnosis of large vessel vasculitis (LVV) is necessary to reduce associated morbidity and mortality. Clinical presentation is highly variable and there are ethnic and regional differences in the incidence and prevalence. Paediatric rheumatologists therefore require a high index of suspicion to establish the diagnosis early. Ultrasound (US) is a quick, non-invasive imaging tool for suspected LVV.

**Objectives:** To demonstrate the utility of US in the diagnosis of LVV.

**Methods:** We report two patients who presented acutely with joint pain and swelling, weight loss, generalized muscular atrophy and a marked acute phase reaction. Fast track ultrasound of the heart and aortic branches was performed as part of the initial diagnostic workup.

**Results:** US findings for Patient 1 (13 year-old female, Turkish ancestry) showed the halo sign in the area of the descending aorta combined with massive aortic dilatation and the aliasing phenomenon in the upper descending aorta. US results for Patient 2 (17 year-old male, Bulgarian ancestry) showed the halo sign in the abdominal aorta with increased aortic flow and stenosis at the outgoing upper mesenteric artery. Blood flow was reduced at the poststenotic site. Additionally, infiltration of the left carotid artery wall was detected. Both patients were transferred to a tertiary paediatric cardiology centre. MRI angiography and PET-MRI in Patient 1 demonstrated inflammatory changes in the wall of the aortic aneurysm. Cardiothoracic surgery with aortic arch grafting was performed. A diagnosis of Behçet’s disease was made based on the additional findings of recurrent major oral aphthous ulcers, soft tissue inflammation and HLA-B51 positivity. Patient 2 also underwent MRI and PET-MRI testing which demonstrated extensive inflammatory changes in the thoracic and abdominal aorta and multiple first branch vessels, leading to a diagnosis of Takayasu arteritis.

**Conclusion:** Ultrasound is a useful non-invasive tool for the screening of LVV. Typical findings are related to inflammation of the arteries including 1) intimal oedema forming a hypoechoic ring at the lumen periphery (the “halo sign”); 2) lumen stenosis with corresponding increased systolic blood flow velocity; and/or 3) vessel occlusion. Colour Doppler US may show the “aliasing phenomenon” post-stenosis. With full lumen occlusion, no colour signal will be detected. Whilst LVV is very rare in paediatric rheumatology, left undiagnosed or mismanaged LVV may result in serious adverse outcomes.

**Patient Consent Received**

Yes

**Disclosure of Interest**

None declared

### P162 Ultrasound imaging of the vascularization of salivary glands in paediatric patients with primary or secondary Sjögren’s syndrome

#### D. Windschall^1,2^, S. Schua^1^, S. Hardt^1^, F. Gohar^1^

##### ^1^Clinic for Paediatric and Adolescent Rheumatology, St. Josef-Stift Hospital, Northwest German Center for Rheumatology, Sendenhorst; ^2^University Halle-Wittenberg, Halle, Germany

###### **Correspondence:** D. Windschall

**Introduction:** Salivary gland ultrasound (SGUS) in symptomatic paediatric patients with systemic erythematosus lupus (JSLE), Sjogren’s disease (jSS) and mixed connective tissue disease (MCTD) show abnormalities in up to 96% of patients.^1^

**Objectives:** Semi-quantitative SGUS scoring tools of B-Mode (BM) SGUS findings have been shown to be feasible and accurate.^2^ We performed the first systematic evaluation of SGUS hypervascularisation as a marker of inflammation in paediatric patients, using the OMERACT scoring tool used so far in adult patients.^3^

**Methods:** We report 7 patients with JSLE, jSS or MCTD with or without sicca or parotitis symptoms who underwent SGUS with BM and PD with a linear high-frequency transducer. We used the following semiquantitative scores: Vascularization: Grade 0: no visible vascular signals; Grade 1: focal, dispersed vascular signals; Grade 2: diffuse vascular signals detected in less than 50% of the gland; Grade 3: diffuse vascular signals in more than 50% of the gland. B-Mode: Grade 0: normal parenchyma; Grade 1: mild inhomogeneity without anechoic or hypoechoic areas and hyperechogenic bands; Grade 2: moderate inhomogeneity with focal anechoic or hypoechoic areas; and Grade 3: severe inhomogeneity with diffuse anechoic or hypoechoic areas occupying the entire gland or a fibrous gland.

**Results:** Table 1 summarises the clinical findings and SGUS vascularity and BM findings. All patients (6 female, 1 male) had pathological SGUS findings, even in the absence of current symptoms. Additionally, SGUS vascularity and BM grading showed good correlation (correlation coefficient 0.91).

**Table:** 

Patient	Diagnosis	Sicca Symptoms	Age at diagnosis	Age at scan	Current medication	CRP mg/dL	ESR (BSG) mm/h	SGUS BM (max grade all SG)	SGUS PD Score (max grade all SG)
1	MCTD, JSS	yes	16	19	HCQ + AZA	0,16	**26**	3	2
2	MCTD	yes	12	14	MTX + AZA + Prednisolon	neg	5	1	0
3	MCTD, incomplete JSLE, jSS	yes	4	14	HCQ + MTX	neg	10	3	2
4	MCTD / jSS, DD JSLE	no	12	14		neg	13	2	1
5	MCTD	yes	6	7	MTX + HCQ + Prednisolon	neg.	8	3	3
6	jSS	no	15	16	HCQ	neg.	12	3	2
7	JSLE	no	12	16	HCQ + MTX	0,17	32	3	3

**Conclusion: Summary:** This preliminary investigation of SGUS-vascularisation in paediatric patients showed good correlation with BM findings even in the absence of clinical symptoms, suggesting a role for performing SGUS routinely as part of the diagnostic workup. Further studies in paediatric patients are necessary to confirm our findings.

**References**

1. Krumrey-Langkammerer and Haas JP. *Paediatric Rheumatology* 2020:18;44. 2. Hocevar A et al. Eur J Radiol. 2007;63(3):379-83. 3. Finzel et al. Rheumatology. 2021:14;60(5)

**Patient Consent Received**

No

**Disclosure of Interest**

None declared

### P163 Validation of normative sonographic data in JIA patients with knee arthritis – preliminary data from the imaging working groups of the PReS and GKJR

#### D. Windschall^1,2^, R. Trauzeddel^3^, F. Gohar^4^, S. Schua^4^, S. Hardt^4^, M. Krumrey-Langkammmerer ^5^, L. Fotis^6^, R. Berendes^7^, M. Haller^8^, P. Maschmeyer^9,10^, S. Magni-Manzoni ^11^ on behalf of Imaging working groups of the PReS and GKJR

##### ^1^Clinic for Paediatric and Adolescent Rheumatology, St. Josef-Stift Hospital, Northwest German Center for Rheumatology, Sendenhorst; ^2^University Halle-Wittenberg, Halle; ^3^Department of Paediatrics, Helios Klinik Berlin-Buch, Berlin; ^4^Clinic for Paediatric and Adolescent Rheumatology, St. Josef-Stift Hospital, Northwest German Center for Rheumatology, Sendenhorst; ^5^Clinic for Paediatric and Adolescent Rheumatology, German Center for Paediatric and Adolescent Rheumatology, Garmisch-Partenkirchen, Germany; ^6^ Department of Pediatrics, Attikon General Hospital, National and Kapodistrian University of Athens, Athens, Greece; ^7^Paediatric Rheumatology, Kinderkrankenhaus St. Marien gGmbH, Landshut; ^8^Kinder- und Jugendarztpraxis, Gundelfingen; ^9^Berlin Institute of Health at Charité, Charité - Universitätsmedizin Berlin; ^10^Berlin Institute for Medical Systems Biology, Max Delbrück Center for Molecular Medicine in the Helmholtz Association (MDC), Berlin, Germany; ^11^U.O. di Reumatologia,Dipartimento di Pediatrie Specialistiche, IRCCS Ospedale Pediatrico Bambino Gesù, Rome, Italy

###### **Correspondence:** D. Windschall

**Introduction:** Ultrasound B-Mode (BM) and vascularity (PD) can discriminate normal and pathological findings, identifying joint effusion, synovial proliferation and hypervascularity in patients with juvenile idiopathic arthritis (JIA). Physiological ultrasound (US) findings for the paediatric knee joint has been published in 2016 by the Imaging Working Groups of the GKJR and OMERACT.

**Objectives:** To compare knee joints of JIA patients with and without arthritis using pD and bM to determine the most sensitive US views in an international multicenter study.

**Methods:** Patients aged ≤ 18 years attending with clinical symptoms of arthritis of 1 or both knee joints in 6 participating centres were recruited. Standardized US examination was performed (Table 1) using a linear transducer and bM and pD findings were graded 0 (normal)-3 (severe) according to the adapted OMERACT-US score.

**Results:** A total of 75 individual knee joint examinations in 53 patients were performed, knees without arthritis were controls. Patient reported symptoms of arthritic knees were swelling (98%), loss of function (85%) and pain (83%). At the time of scanning, 57% of patients were using NSAR therapies, 23% methotrexate, 2% biological and 2% oral steroids. In BM, the commonly used anterior longitudinal standard view performed in 30° remained the most sensitive for assessing effusion when used as independent view. However, PD in lateral parapatellar and lateral longitudinal views were more sensitive than the typically evaluated anterior longitudinal and transverse in neutral view. The highest sensitivity for hypervascularization was seen in the parapatellar views, the lateral longitudinal and the anterior transverse and longitudinal views performed at 30°.

**Table:** 

	Cases (n=53)	
	PD Grade 1, 2 or 3 % (n)	BM Grade 1, 2 or 3 % (n)
Anterior longitudinal	44 (23)	89 (47)
Anterior longitudinal 30°	64 (34)	95 (51)
Anterior transverse	55 (29)	89 (47)
Anterior transverse 30°	63 (34)	88 (47)
Medial longitudinal	49 (26)	77 (41)
Lateral longitudinal	64 (34)	91 (48)
Medial parapatellar	62 (33)	81 (43)
Lateral parapatellar	69 (37)	85 (45)

**Conclusion:** These preliminary findings suggest US could be performed with knees in 30° flexion (anterior longitudinal and transverse) for maximal BM sensitivity. These views combined with the lateral parapatellar and longitudinal views as marker for hypervascularity provide the most sensitive US method.

**Patient Consent Received**

Yes

**Disclosure of Interest**

None declared

## e-Poster viewing: Disease outcome and transition

### P164 Long-term outcomes in adult patients with childhood-onset systemic lupus erythematosus

#### A. Mirguet^1^, N. Costedoat^2^, A. Hummel^3^, V. Le Guern^2^, I. Lemelle^4^, Z. Amoura^5^, R. Carlomagno^6^, A. Belot^7^, T. Remen^8^, B. Bader-Meunier^9^

##### ^1^Pediatric Nephrology and Rheumatology, CHRU Nancy, Vandoeuvre-lès-Nancy; ^2^Department of Internal Medicine, Cochin Hospital; ^3^Department of Nephrology, Necker Hospital, Paris; ^4^Department of Pediatric Onco-hematology, Children Hospital, University Hospital of Nancy, Nancy; ^5^Department of Internal Medicine, La Pitié-Salpétrière Hospital, Paris, France; ^6^Rheumatology, Immunology and Allergology Unit, Department of Pediatrics, Vaudois University Hospial, Lausanne, Switzerland; ^7^Department of Pediatric Rheumatology, Nephrology, and Dermatology, Hospital Femme-Mère-Enfant, Hospices Civils de Lyon, Lyon; ^8^Methodology, Data Management and Statistic Unit, MPI Department, University Hospital of Nancy, Nancy; ^9^Department for Immunology, Hematology and Pediatric Rheumatology, Necker Hospital, Paris, France

###### **Correspondence:** B. Bader-Meunier

**Introduction:** Systemic Lupus Erythematosus (SLE) is a chronic systemic autoimmune disease. Data regarding the long-term course of childhood-onset SLE patients are scarce.

**Objectives:** Aims of this study were to describe disease activity and damage in adulthood in juvenile-onset SLE patients, to identify the risk factors at the time of diagnosis for renal and neurological damage and to compare patient profiles according to follow-up duration.

**Methods:** We conducted a national retrospective multicenter study using data from the PEDIALUP registry of the JIRcohort database. Demographic characteristics, clinical, laboratory, radiological, histological data and treatments were collected from medical records during the follow-up, as well as clinical scores as SLICC-DI, used to assess irreversible damage linked to the disease, to treatments or to associated comorbidities.

**Results:** We analyzed 228 patients (90 % women and 63 % Caucasians) with a median follow-up of 16 years. At the last visit, 41 % of patients had a SLICC-Damage Index score ≥ 1. Musculoskeletal, neurological, cutaneous, cardiovascular and renal damage were the most frequent. SLICC-DI score was correlated with the number of renal flares (β = 0.19, p = 0.02). The proportion of patients with a SLICC-DI score ≥ 1 increased significantly with follow-up duration (p = 0.002). In univariate analysis : 1) non-Caucasian ethnicity (p = 0.03), class IV nephropathy (p = 0.03), change of class of lupus nephropathy (p = 0.01), number of renal flares (p = 0.003) and neurolupus at diagnosis (p = 0.04) were significantly associated with the presence of renal damage at the last visit; 2) antiphospholipid antibodies (p = 0.01), antiphospholipid syndrome (p < 0.0001) and neurolupus at diagnosis (p < 0.0001) were strongly associated with the presence of neurological damage at the last visit. At the last visit, 34 % patients still had active disease with a SLEDAI score ≥ 6.

**Conclusion:** Damages and iatrogenic complications are frequent in childhood-onset SLE patients after long-term follow-up. A large proportion of patients still has persistent active disease at the last visit. The management of complications remains a major issue in this severe and chronic disease.

**Patient Consent Received**

Yes

**Disclosure of Interest**

None declared

### P165 Health-related quality of life, continuity of care and patient satisfaction in rheumatic diseases: health care transition program tuebingen

#### L. Böker^1^, J. Kümmerle-Deschner^1^, J. Klotsche^2^, S. Hansmann^1^

##### ^1^Center for Pediatric Rheumatology and autoinflammation reference center Tuebingen (arcT), University Children's Hospital Tuebingen, Tuebingen; ^2^Programme area Epidemiology and Health Care Research, German Rheumatism Research Centre Berlin and Leibniz Institute, Berlin, Germany

###### **Correspondence:** L. Böker

**Introduction:** A significant number of pediatric rheumatic and inflammatory patients have ongoing disease activity into adulthood associated with limitations in daily life. Successful transfer from pediatric to adult rheumatology care maintains low disease activity and high health-related quality of life (HR-QoL)(1).

**Objectives:** To evaluate I) HR-QoL of patients after transfer to adult rheumatology compared to the German population norm and II) satisfaction with the transition process and continuity of care.

**Methods:** The Tuebingen rheumatology health care transition (HCT) program is a well-established holistic, interdisciplinary concept and comprises pediatric and adult rheumatologists, nursing, physiotherapy, and psycho-social care. A single-center cross-sectional study was performed, including patients of the HCT program between 2000 and 2020. In an iterative team process, a standardized questionnaire was generated, including HR-QoL (EQ-5D-5L), satisfaction (10 cm VAS) and questions regarding transfer-readiness, time of transfer, disease activity and continuity of care. Data was described using absolute and relative frequencies. A logistic regression analysis was performed to identify correlates.

**Results:** 85 of 295 patients completed the questionnaire (response rate 28.8%). 70.6% were female, median age was 24.1 years (range 19.1-40.5), 65 were diagnosed with Juvenile Idiopathic Arthritis (76.5%). The mean score in each dimension of the EQ-5D-5L was below 1.8. The EQ VAS (100 mm) had a mean score of 79.8 (SD 19.0). Pain/discomfort was the most frequent problem (52.9%). 91.8% reported no problems with self-care. Problems with usual activities (35.7%) and anxiety/depression (42.2%) were reported more often than in the general population (18.3%; 22.6%). 78.3% (65/83) reported regular care by an adult rheumatologist. Of the 18 respondents without such care, 14 cited a lack of disease activity as a reason. Mean satisfaction with pediatric care (8.4; SD 1,7) and transition clinic (7.9; SD 2.6) were higher than satisfaction with adult care (7.7; SD 2.2). Satisfaction with preparation for transfer (6.8; SD 3.0) was the lowest score but showed negative correlation with time since transfer (beta -0.34). Low physician global assessment (PGA) at time of transfer was associated with high HR-QoL after transfer.

**Conclusion:** The long-term HR-QoL in our cohort is lower than that of the general population, although problems with self-care (8.8%), mobility (31.6%) and pain/discomfort (56.1%) of JIA patients are comparable to literature (10.7%; 25.2%; 56.4%) (2). Continuity of care was higher than in similar studies (74.0%) (3). Satisfaction with the HCT program was high, including a recent significant increase in satisfaction with preparation for transfer. While the burden for rheumatic long-term patients remains elevated, a structured interdisciplinary HCT program can improve patient satisfaction and soon, hopefully, long-term HR-QoL.

**References**

1. Hilderson D, Moons P, Van der Elst K, Luyckx K, Wouters C, Westhovens R. The clinical impact of a brief transition programme for young people with juvenile idiopathic arthritis: results of the DON'T RETARD project. Rheumatology (Oxford). 2016;55(1):133-42.

2. Barth S, Haas JP, Schlichtiger J, Molz J, Bisdorff B, Michels H, et al. Long-Term Health-Related Quality of Life in German Patients with Juvenile Idiopathic Arthritis in Comparison to German General Population. PLoS One. 2016;11(4):e0153267.

3. Stringer E, Scott R, Mosher D, MacNeill I, Huber AM, Ramsey S, et al. Evaluation of a Rheumatology Transition Clinic. Pediatr Rheumatol Online J. 2015;13:22.

**Patient Consent Received**

No

**Disclosure of Interest**

None declared

### P166 Musculoskeletal diagnoses prior to cancer in children: a danish registry-based cohort study

#### N. Brix^1^, J. Amstrup^2^, M. Nørgaard^3^, S. Hagstrøm^2^, H. Hasle^1^, T. Herlin^1^

##### ^1^Department of Pediatrics and Adolescent Medicine, Aarhus University Hospital, Aarhus; ^2^Department of Pediatrics and Adolescent Medicine, Aalborg University Hospital, Aalborg; ^3^Clinical Epidemiology Department, Aarhus University Hospital, Aarhus, Denmark

###### **Correspondence:** N. Brix

**Introduction:** Children with cancer often present with diffuse signs which complicates the diagnostic process, especially if musculoskeletal symptoms are the initial manifestation of malignancy. Arthritis and arthropathy are the most frequent symptoms leading to cancer misdiagnosed as rheumatic disease. Joint involvement, although well-recognized in children with acute lymphoblastic leukemia, has only rarely been described in children with other types of cancer. Yet, limited data exist on the prevalence of musculoskeletal diagnoses prior to cancer diagnosis in children.

**Objectives:** Our objective was to identify the prevalence of musculoskeletal diagnoses leading to hospital contact within six months preceding cancer diagnosis in children. Secondarily, to evaluate whether preceding musculoskeletal diagnoses affected survival.

**Methods: Design, Setting, and Participants:** We used data from population-based medical registries covering all Danish hospitals to identify a cohort of children diagnosed with cancer over a 23-year period (1996–2018). We compared children with musculoskeletal diagnoses recorded within six months preceding the cancer diagnosis and children without musculoskeletal diagnoses using prevalence ratios and 95% confidence intervals (CI).

**Exposure:** A musculoskeletal diagnosis (ICD10 codes, M00-M99) recorded in the Danish National Patient Registry within six months before cancer.

**Main Outcome and Measures:** Survival measured as five-year overall survival, crude, and adjusted hazard ratios.

**Results:** Among 3,895 children with cancer, 7% (n = 264) had at least one musculoskeletal diagnosis recorded within six months preceding cancer diagnosis. These 264 patients had a total of 451 visits across Danish hospital departments with a median of two visits (range 1–10). The overall median physician’s diagnostic interval from first musculoskeletal diagnosis to cancer diagnosis was 15 days (IQR 7–47). The five-year overall survival did not differ for children with a prior musculoskeletal diagnosis 84.5% (95% CI 79.2–88.6) compared to those without 84.2 (95% CI 82.9–84.4), except for spinal tumors being favorable for those with a prior musculoskeletal diagnosis: adjusted hazard ratio: 0.23 (95% CI 0.05–0.98).

**Conclusion:** A preliminary musculoskeletal diagnosis occurred in 7% of children with cancer but did not affect overall survival. Furthermore, this study provides an insight into musculoskeletal diagnoses prior to cancer diagnosis.

**Patient Consent Received**

No

**Disclosure of Interest**

None declared

### P167 Disease persistence beyond age 16 in patients with juvenile idiopathic arthritis: a descriptive study using electronic health records in England

#### R. E. Costello^1^, L. Kearsley-Fleet^1^, J. E. McDonagh^1^, K. Hyrich^1,2^, J. H. Humphreys^1^

##### ^1^Centre for Epidemiology Versus Arthritis, University of Manchester; ^2^National Institute of Health Research Manchester Biomedical Research Centre, Manchester University NHS Foundation Trust, Manchester, United Kingdom

###### **Correspondence:** R. E. Costello

**Introduction:** It is estimated that juvenile idiopathic arthritis (JIA) persists into adulthood in at least one-third of patients, but it is not clear how frequently hospital services are used beyond age 16.

**Objectives:** To describe characteristics related to disease persistence in young people (YP) with JIA using electronic health records (EHR) in England.

**Methods:** YP with JIA were identified from primary care EHR (Clinical Practice Research Datalink GOLD and Aurum databases) between 2003 and 2018. JIA was identified if they had a Read code for JIA and either >=3 Hospital Episode Statistics (HES) outpatient specialist care (rheumatology/ophthalmology) appointments or a HES inpatient admission coded with JIA, prior to age 16. Further, cases needed to have linkage to HES data, registration at the same GP for >1 year beyond age 16. Cases were followed from the earliest of first Read code or first HES outpatient appointment until leaving their GP or end of 2018. YP with >=1 specialist care outpatient appointment beyond age 16 were considered to have “persistent disease”. YP were considered discharged from specialist care (a proxy for disease remission) if they had >=1 year between the last appointment and end of study follow-up. For both databases, patient characteristics and characteristics of discharged are presented, stratified by disease persistence.

**Results:** Of 192 and 903 YP eligible for the study, 135(70.3%) and 639(70.8%) had persistent disease in GOLD and Aurum, respectively. Those with persistent disease were older at first JIA code and more likely to be female compared to those without persistent disease (Table 1). Overall, 90/192(46.9%) and 453/603(50.2%), in GOLD and Aurum respectively, remained in specialist care after age 18. Of those with persistent disease, approximately 41% were discharged prior to the end of study follow-up, with 33% and 29% discharged between ages 16-18 in GOLD and Aurum respectively. The median age of the last visit was 14 for those without persistent disease and 18 for those discharged after the age of 16. Disease duration was longer in those discharged after age 16.

**Conclusion:** Over two-third of patients with JIA continue to have hospital visits beyond age 16. Of those, however, approximately 4 in 10 were discharged from specialist care by the age of 18. These data provide important information for YP with JIA and their families, as well as clinicians running, developing and planning young adult services in paediatric and adult rheumatology.

**Disclosure of Interest**

None declared

**Table 1 (abstract P167).** Characteristics of patients and remission, by disease persistence

		GOLD – Disease not persistent N = 57	GOLD – Disease persistent N = 135	Aurum – Disease not persistent N = 264	Aurum – Disease persistent N = 639
Duration of follow-up (years), median (interquartile range (IQR)		9.9(7.1, 12.5)	9.2(6.3, 12.0)	12.5(8.5, 15.4)	12.6(8.9, 15.6)
Age first code, n(%)	0-<4 years	8(14.0)	7(5.2)	41(15.5)	108(16.9)
	4-<9 years	13(22.8)	15(11.1)	79(29.9)	141(22.1)
	9-<16 years	36(63.2)	113(83.7)	144(54.5)	390(61.0)
Gender, n (%)	Male	26( 45.6)	49(36.3)	130( 49.2)	228(35.7)
	Female	31( 54.4)	86(63.7)	134( 50.8)	411(64.3)
Discharged, n(%)	16-18 years	-	45(33.3)	-	186(29.1)
	>18 years	-	10(7.4)	-	76(11.9)
Age last appointment if discharged, median (IQR)		13.9(12.6, 15.3)	17.8(17.0, 20.4)	13.7(11.9, 15.2)	18.2(16.9, 20.7)
Disease duration at last appointment if discharged, median (IQR)		2.9(1.4, 5.7)	6.3(3.7, 9.6)	3.4(1.6, 6.3)	7.6(4.3, 12.2)

### P168 Proportion of children and young people with juvenile idiopathic arthritis (JIA) stopping biologic therapy for remission

#### L. Kearsley-Fleet^1^, C. Ciurtin^2^, E. Baildam^3^, M. W. Beresford^3,4^, S. Douglas^5^, H. E. Foster^6^, T. R. Southwood^7^, K. L. Hyrich^1,8^ on behalf of UK JIA Biologic Registers (BCRD and BSPAR-ETN)

##### ^1^Centre for Epidemiology Versus Arthritis, Manchester Academic Health Science Centre, The University of Manchester, Manchester; ^2^University College London, London; ^3^Alder Hey Children's NHS Foundation Trust; ^4^University of Liverpool, Liverpool, United Kingdom; ^5^Scottish Network for Arthritis in Children (SNAC), Scotland, United States Minor Outlying Islands; ^6^Newcastle University, Newcastle; ^7^University of Birmingham, Birmingham; ^8^National Institute of Health Research Manchester Biomedical Research Centre, Manchester University NHS Foundation Trust, Manchester Academic Health Science Centre, Manchester, United Kingdom

###### **Correspondence:** L. Kearsley-Fleet

**Introduction:** Biologic therapies are common treatments used in children and young people with juvenile idiopathic arthritis (JIA). Concerns about their long-term safety in children and young people has prompted many clinicians to consider tapering or stopping these treatments in patients who have achieved remission, but currently it is unclear whether this is an effective decision and what proportion of children will flare and require further biologic therapy.

**Objectives:** This analysis aimed to estimate the proportion who stop biologic therapy for remission, how long they were on therapy prior to stopping, how many then re-start biologic therapy and after how long.

**Methods:** All children and young people with JIA registered on their first biologic without a history of uveitis into the UK JIA Biologic Registers from 1-Jan-2010 up to 1-Feb-2021. Systemic JIA patients were only included if they were starting either IL-1 or IL-6 inhibitors. Other JIA patients were only included if they were starting a TNF inhibitor. Time on drug prior to remission was calculated from the original start date of the biologic therapy, until the date the therapy was stopped for remission, regardless of interim episodes of stopping due to non-remission reasons. Tapering could not be identified, only stop of therapy. Results were stratified by systemic and non-systemic JIA to account for the differences between ILAR subtypes.

**Results:** A total of 878 children and young people with JIA were included.

Of the 793 non-systemic JIA patients starting TNF inhibitors – the majority polyarticular RF- (37%), extended-oligoarticular (19%), and ERA (15%) – 131 (17%) patients stopped their first biologic for remission after a median of 2.2 years (IQR 2.0, 2.9). However, 44% later re-started biologic therapy, usually the same biologic (84%), after a median of 4.7 months.

Of the 85 systemic JIA patients starting IL-1 or IL-6 inhibitors, 25 (29%) stopped their first biologic for remission after a median of 2.2 years (IQR 1.5, 3.2). However, 20% later re-started biologic therapy, usually the same biologic (80%), after a median of 4.4 months.

There was no evidence to support that more systemic JIA patients were stopping for remission; age and gender adjusted hazard ratio 1.3 (95% CI 0.8-2.0) compared with non-systemic JIA patients.

**Conclusion:** In this large UK study, around 1 in 5 children had their biologic stopped following achievement of remission. For children with non-systemic JIA, almost half restarted their biologic within 2 years, although this proportion was lower for systemic JIA. These data suggest that tapering biologics rather than stopping completely should be explored in some children to minimise biologic exposure without triggering a flare.

**Patient Consent Received**

No

**Disclosure of Interest**

L. Kearsley-Fleet: None declared, C. Ciurtin: None declared, E. Baildam: None declared, M. Beresford: None declared, S. Douglas: None declared, H. Foster: None declared, T. Southwood: None declared, K. Hyrich Consultant for: Abbvie

**Table 1 (abstract P168).** See text for description

	Non-Systemic JIA Starting TNF N=793	Systemic JIA Start IL-1/6 N=85
**Registered Biologic therapy**		
Etanercept	544 (69%)	-
Infliximab	28 (4%)	-
Anakinra	-	28 (33%)
Adalimumab	220 (28%)	-
Tocilizumab	-	57 (67%)
Golimumab	1 (<1%)	-
**Concomitant MTX**	450 (57%)	58 (68%)
**Female**	527 (66%)	49 (58%)
**Age, years**		
Median (IQR)	12 (8, 14)	7 (3, 12)
Min to Max	1 to 20	1 to 17
**Disease Duration, years**	N=782	N=85
Median (IQR)	1 (1, 4)	1 (0, 1)
Min to Max	0 to 18	0 to 9
**Remission (% of whole cohort)**	131 (17%)	25 (29%)
**Time to Remission, years**	2.2 (2.0, 2.9)	2.2 (1.5, 3.2)
**Time to end of follow-up, if >0**	1.5 (0.8, 2.5) N=123	1.4 (0.8, 2.5) N=24
**Re-started biologic therapy**	58 (44%)	5 (20%)
Time to re-start, years	0.4 (0.2, 0.7)	0.4 (0.1, 0.4)
Re-started same biologic	49 (84%)	4 (80%)

### P169 Description of work productivity of adulthood juvenile idiopathic arthritis patients

#### T. Kishi^1,2^, Y. Tani^2^, E. Tanaka^3^, T. Kawabe^2^, S. Nagata^1^, M. Harigai^3^, T. Miyamae^2^

##### ^1^Department of Pediatrics, Tokyo Women's Medical University, School of Medicine; ^2^Pediatric Rheumatology, Tokyo Women's Medical University, Institute of Rheumatology; ^3^Division of Rheumatology, Department of Internal Medicine, Tokyo Women’s Medical University, School of Medicine, Tokyo, Japan

###### **Correspondence:** T. Kishi

**Introduction:** The treatment and outcome of juvenile idiopathic arthritis (JIA) have become much improved in recent decades. Nevertheless, some patients still have difficulty in the daily living because of chronic synovitis or functional disability with long-term illness duration. It has been reported that the employment rate of adulthood JIA patients was not at a low level in recent years. However, the impact of the disease on work productivity, such as restrictions on choice of job and the place of work, employment status, or annual income, is not well understood.

**Objectives:** The aim of this study was to evaluate the impact of JIA on the work productivity of long-term follow-up patients.

**Methods:** A questionnaire survey was administered to patients with adulthood JIA (age of disease onset was under 16 years) who were younger than 60 years of age at the time of this study in the rheumatology department of Tokyo Women’s Medical University Hospital. The questionnaire included job status, job description, commuting methods, commuting time, and annual income.

**Results:** We included 55 (Female: 87%) adulthood JIA patients in this study, the median and interquartile (IQR) age at onset of disease was 14 [10-15] years, and at evaluation was 36 [28-43] years. Of the 55 cases, 45 (82%) were employed, four were students, four were mainly housekeeping with no remuneration, and the remaining two cases were unemployed. Of the 45 employed patients, 36 (80%) were full-time and 9 (20%) were part-time. The employment rate was 82%, which was similar to the women's rate in Tokyo for the same age group (76–81%). The type of jobs was categorized into three types. Patients who have mainly engaged in a sedentary job (e.g., administrative work, accounting work) were 35 (71%), a job that mainly a walkaround (e.g., salesperson, nurse) were 7 (14%), and a job that requires much standing (e.g., teacher, shop clerk) were 3 (6%). Significantly more patients with a disability certificate were engaged in sedentary work than those without (95% vs. 52%, p<0.01). The median [IQR] time since their first job was 15 [10-22] years, 28 (57%) patients had changed jobs at least once, and of these, 8 (16%) patients had changed jobs because of JIA. However, there was no difference[MH1] in the era of onset or disease duration between patients who changed jobs and not. The most common method of commuting was public transportation with 24 (49%) patients. Regarding commuting time, 23 (47%) patients took less than 15 minutes, 11 (22%) took 15 to 30 minutes, and 6 (12%) took more than 60 minutes. These results tended to be slightly shorter than the average commute time of 44 minutes in Tokyo. There were 27 (59%) patients with an annual income of fewer than 4 million yen. Among them, 11 (24%) had an annual income fewer than 2 million yen. Moreover, none had an annual income of more than 10 million yen. This result was similar to women's average annual income of 2.8 million yen in Tokyo. Regarding to academic background, 95% of patients had graduated high school, and 49% had a college degree.

**Conclusion:** In the long-term disease course, the majority of the adult JIA patients were working, and the impact of the disease on work productivity was limited, despite some patients changed jobs because of the JIA. The commuting time was shorter compared to the general population of the region. Furthermore, the patients tended to be engaged in mainly sedentary office work, especially patients with functional disability.

**Patient Consent Received**

Yes

**Disclosure of Interest**

None declared

### P170 Transition readiness in adolescents with juvenile idiopathic arthritis (JIA) and their parents - our expirience

#### D. Lazarević^1,2^, S. Đorđević^3^, D. Novaković^4^, G. Sušić^4^

##### ^1^Department of Pediatric Rheumatology, Clinic of Pediatrics, University Clinical Center Niš; ^2^Faculty of Medicine, University of Niš, Nis; ^3^University Children`s Hospital; ^4^Institute of Rheumatology, Belgrade, Serbia

###### **Correspondence:** D. Lazarević

**Introduction:** The transition from pediatric to adult care is a very vulnerable period for patients with juvenile idiopathic arthritis (JIA) and their families. Future study results and the formal transitional program could help to overcome existing difficulties.

**Objectives:** This study aimed to evaluate which JIA patient disease characteristics might lead to self-management skills improvement in the transition readiness process. We also wanted to explore the readiness of JIA patients and their families for the transition process into the adult health care system.

**Methods:** We have recruited different JIA patient subtypes and their parents from a single study center. Demografic data were collected and Transition Readiness Assessment Questionnaire (TRAQ) was applied to all patients and their parents at one time point.

**Results:** A total of 44 JIA patients (9 males and 35 females; median age 15.12 years, range 12.33 to 19.33 years; median disease duration 4.29 years, range 0.42 to 17.5 years) and their parents were enrolled. Fourteen (31.82%) of 44 JIA patients had a concomitant disease while 10 (22.73%) of them had uveitis. Eleven (25%) of them had a family history of autoimmune diseases. In total, 21(47.7%) of JIA patients were receiving biologics. There was a strong correlation between older patient age and total TRAQ score among patients (ρ=0.799, p<0.0001) and a moderate correlation between older patient age and total TRAQ score among parents (ρ=0.522, p<0.0001). Total TRAQ score has a strong correlation between patients and parents (ρ=0.653, p<0.0001). We could not find any association of JIA patient characteristics (JIA disease subtypes, disease duration, gender, concomitant diseases, uveitis, family history of autoimmune diseases, number of hospitalizations, and treatment with biologics) with TRAQ scores and JIA patients' and parents' readiness for transition.

**Conclusion:** Transition readiness of JIA patients increase with advancing age. There is no difference between transition readiness for JIA patients and their parents. Future studies with a larger sample size and new insights are needed in order to facilitate this challenging transition process.

**Patient Consent Received**

Yes

**Disclosure of Interest**

None declared

### P171 Outcome of sarcoidosis in children: about a series of five cases

#### H. Nassih, R. Elqadiry, A. Bourrahouat, I. Aitsab

##### Pediatrics, Mohammed 6 University Hospital Center of Marrakesh, Marrakesh, Morocco

###### **Correspondence:** H. Nassih

**Introduction:** Childhood sarcoidosis is a rare multi-systemic granulomatous disorder of unknown etiology. The long-term course and prognosis is not well established in childhood sarcoidosis, but it appears to be poorer in early-onset disease.

**Objectives:** To describe the clinical course and outcome of juvenile sarcoidosis.

**Methods:** A single center retrospective descriptive study of five cases of juvenile sarcoidosis.

**Results:** All of our patients were girls. The median age at diagnosis was of 11-year-old, with extremes of 9 and 14-year-old. The interval between onset of symptoms and diagnosis was of 2 months. One of the cases was treated at first as tuberculosis and another one was treated as systemic juvenile idiopathic arthritis. Prolonged fever and arthritis were a revealing pattern in all cases. Meanwhile, we found uveitis in one case, lymphadenopathies in 3 cases, hepatosplenomegaly in 2 cases, interstitial lung disease in 4 cases, and liver failure with cholestasis in one case. Macrophages activating syndrome was a revealing pattern in one case. Acute phase reactants were high in all cases, with medians of ESR of 23 mm the first hour, CRP of 31 mg/L, and ferritin of 340 ng/ml. Meanwhile, hematological involvement was in the form of microcytic anemia in one case, leucopenia in 3 cases, lymphopenia in 2 cases, thrombocytopenia in one case, and aregenerative normocytic anemia in one case. High angiotensin 2 conversing enzyme level was found in all cases, with a mean of 181 ui/L. Biopsy of the lymphadenopathies showed granulomas in one case. Treatment was based on steroids alone in one case. Meanwhile, associated immunosuppressive therapy was needed in the remaining cases. Azathioprine was prescribed in 2 cases, methotrexate in one case, and mycophenolate mofetil in one case. The mean treatment period was of 42 months. Evolution was marked by complete remission in one case, and recurrence of the symptoms after treatment withdrawal in the remaining cases. One child had blindness after a severe refractory uveitis.

**Conclusion:** Sarcoidosis symptoms are nonspecific and often lead to misdiagnosis. Management can be difficult in the pediatric population, and recurrence of the disease after treatment withdrawal is frequent.

**Disclosure of Interest**

None declared

### P172 A data science evaluation of the Juvenile Arthritis Multidimensional Assessment Report (JAMAR) questionnaire for improving management of JIA patients

#### H. Quesada-Masachs^1^, M. Lopez-Corbeto^2^, M. Faloutsos^1^, S. Ghose^3^, S. Marsal^4^, E. Quesada-Masachs^2^

##### ^1^Computer Science, University of California Riverside, Riverside, United States; ^2^Pediatric Rheumatology, Hospital Universitari Vall d'Hebron, Barcelona, Spain; ^3^Computer Science, University of California Berkeley, Berkeley, United States; ^4^Rheumatology Department, Hospital Universitari Vall d'Hebron, Barcelona, Spain

###### **Correspondence:** E. Quesada-Masachs

**Introduction:** The Juvenile Arthritis Multidimensional Assessment Report (JAMAR) is a questionnaire developed to comprehensively assess Juvenile Idiopathic Arthritis (JIA) patients. Despite being translated and available in 54 languages, there is still limited literature about it. The length of the questionnaire could have been influencing its clinical practicality.

**Objectives:** The purpose of this study was to answer the following questions:

1. Which are the most informative questions to infer patient’s disease activity?

2. Are these informative questions the same for JIA children and parents?

**Methods:** We included 71 children with JIA according to ILAR criteria, all of them receiving treatment and we followed them up for a year. JAMAR questionnaires were answered by both children and parents at baseline, 6 and 12 months. Also, a thorough clinical examination was performed in every visit: all the joints were clinically assessed for swelling, tenderness, and limited range of motion, Juvenile Arthritis Disease Activity Score (JADAS), disease activity state, parents and patients assessment through Visual Analogue Scale (VAS), physician’s VAS, Erythrocyte Sedimentation Rate (ESR) and C-reactive protein (CRP) were recorded. We applied state of the art machine learning methods in order to find the most relevant questions in JAMAR. The objective was to predict if a patient was clinically active or inactive according to the clinical criteria. Each question from the JAMAR was treated as a potential feature. Additionally, we utilized tensor decomposition to identify relevant patient clusters. Furthermore, we correlated these critical questions with clinical and biological parameters recorded.

**Results:** A total of 374 JAMAR questionnaires were analyzed with our Machine Learning algorithms. First, we trained and evaluated several classifiers and identified those that performed the best. Second, in order to understand better how our features (questions) are performing in our algorithm we calculated and plotted the weight of each question. Finally, we identified a small group of questions as the most relevant for patients and parents. The identified questions exhibited better correlations with the JADAS scores than the non-relevant ones. Not all the most informative questions were the same for JIA children and their parents, highlighting the need of considering these discrepancies when interrogating the parents or JIA patients.

**Conclusion:** In this study, we revised the JAMAR questionnaire by applying modern data mining techniques in a longitudinal dataset. Our results suggest that a small number of questions in the JAMAR questionnaire provide significant information and correlate well with the JADAS scores. We argue that this reduced set of questions could make the data collection easier by trading off the number of questions for frequency and ease of self-reported data collection. More work is needed to make the best use of the information and capabilities that JAMAR offers to physicians.

**Patient Consent Received**

Yes

**Disclosure of Interest**

None declared

### P173 What does the patient well-being vas tell us when the physician global assessment score is zero? Analysis of a large multinational dataset

#### F. Ridella^1^, C. Trincianti^1^, M. Spelta^2^, R. Naddei^2^, C. N. Herrera ^3^, C. Malagon^3^, O. Arguentes^3^, A. Ibanez Estrella^3^, A. Kondi^3^, N. Ruperto^4^, A. Ravelli^2^, A. Consolaro^5^

##### ^1^University of Genova; ^2^Istituto Giannina Gaslini; ^3^Paediatric Rheumatology International Trials Organisation (PRINTO); ^4^Istituto Giannina Gaslini, Paediatric Rheumatology International Trials Organisation (PRINTO); ^5^Istituto Giannina Gaslini, University of Genova, Genova, Italy

###### **Correspondence:** F. Ridella

**Introduction:** Parent- and child-reported outcomes (PCROs) are measures that reflect the parent and child perception of rheumatic disease course and effectiveness of therapeutic interventions. Among PCROs for the assessment of patients with juvenile idiopathic arthritis (JIA), the most widely adopted is the parent/patient global evaluation or well-being visual analogue scale (WB-VAS). Several studies in JIA have highlighted the discrepancies in the assessment of the disease status between the physician and the parent/patient. This difference might be due to the WB-VAS measuring a broader construct than the physician global assessment (PGA).

**Objectives:** To evaluate, in a large multinational sample of JIA patients, the disease characteristics of subjects considered as inactive by the physician with an increased WB-VAS score.

**Methods:** Data from the multinational dataset of patients enrolled in the Epidemiology Treatment and Outcome of Childhood Arthritis (EPOCA) study were analyzed. We have included only subjects with a PGA score of 0. PCROs were collected through the juvenile arthritis multidimensional assessment report (JAMAR). We compared demographic features, socio-economic status, level of education, subtype of JIA diagnosis and the main PCROs (pain level, presence of morning stiffness, count of joints with swelling or pain, functional ability, disease activity level, ongoing therapy, presence of medications side effects and health related quality of life measured with the pediatric rheumatology quality of life (PRQL) scale) between subjects with WB-VAS ≤ 1 and > 1.

**Results:** A total of 3537 patients were sorted into two groups according to the WB_VAS score: 2862 subjects were included in a first group (WB_VAS ≤1); 675 in a second one (WB-VAS >1). Respectively, 17,6% and 18,1% of families belonged to the lower socio-economic status, 70,5% and 71% to the intermediate, 11,9% and 10,8% to the higher. The percentages of patients in the three levels of education was not different in the two groups:20,2% and 22% in the lower, 48,9% and 50,1% in the intermediate, 30,8% and 27,9% in the higher level of education. No significant difference was observed in the distribution of JIA categories in the two groups. Subjects in first group were younger at disease onset (5.6 vs 6.4 years). Comparison of main PCROs results is presented in the table.

**Table:** 

PCROs	WB_VAS ≤1	WB_VAS >1	p
VAS_Pain (average)	0.3 (0.9)	2.4 (2.4)	<0.001
Presence of morning stiffness (%)	227 (8.0)	285 (42.4)	<0.001
Patients under treatment (%)	1919 (67.2)	540 (80.2)	<0.001
Reporting side effects (%)	421 (22.1)	236 (43.9)	<0.001
Number of adverse events to the therapy (average)	0.2 (0.7)	0.8 (1.5)	<0.001
Juvenile Arthritis Functionality Scale (JAFS) Total Score (average)	0.5 (1.6)	3.0 (4.4)	<0.001
JIA Quality of Life (JQL) Total Score (mean)	1.6 (2.3)	6.4 (4.4)	<0.001
VAS-Disease Activity (average)	0.4 (1.3)	2.3 (2.4)	<0.001
Count of active joints (average)	0.2 (0.7)	1.4 (2.2)	<0.001

**Conclusion:** We have analyzed the variables that might determine a difference between the physician’s assessment of inactive disease and the parent’s/patient’s perception of well-being. In particular, socio-economic status, level of education, and gender representation seem not to impact on the general perception of well-being, while pain seems to have the greatest influence on the parent/patient quality of life assessment. Finally, children with lower WB-VAS score were younger at disease onset.

**Patient Consent Received**

Yes

**Disclosure of Interest**

None declared

### P174 Systolic function of the right ventricle of the heart in adolescents with rheumatic diseases

#### T. S. Holovko^1^, N. Shevchenko^1,2^, L. Bohmat^1,2^, V. Nikonova^2^

##### ^1^Department of pediatrics № 2, V. N. Karazin Kharkiv National University; ^2^Department of rheumatology and comorbid states, SI Institute for Children and Adolescents Health Care of NAMS of Ukraine, Kharkiv, Ukraine

###### **Correspondence:** N. Shevchenko

**Introduction:** Rheumatic diseases (RD) are a group of diseases characterized by the development of a wide range of comorbid conditions, primarily of the cardiovascular system. In routine practice, the state of the heart is usually assessed by the work of its left ventricle. However fibrosis of the lung tissue often develops with the involvement of the pulmonary artery system in the process. Therefore, the study of the right ventricle is important in RD.

According to the consensus on the use of biomarkers, the N-terminal inactive fragment (NT-proBNP76), which accumulates in specific granules of cardiomyocytes, is currently of great importance in the diagnosis of heart failure.

In the presence of symptoms, in adults its level is more than 125 pg / ml and in children from 1 to 16 years old more than 83 pg / ml, should be regarded as a diagnostically important criterion.

**Objectives:** To study the systolic function of the right ventricle (RV) myocardium in adolescents with rheumatic diseases, taking into account the level of NT-proBNP in the blood.

**Methods:** 52 adolescents with RD at the age of 13.11 ± 0.89 years were examined. This group included 9 patients with systemic lupus erythematosus (SLE) and 43 with juvenile idiopathic arthritis (JIA). The control group consisted of 44 healthy peers aged 14.73 ± 0.32 years, comparable in age. In order to determine the functional state of the RV of heart, an ultrasound examination was carried out on the LOGIO V2 apparatus by General Electric (USA), with a 3Sc-RS transducer in M- ​​and B-modes.

The ejection fraction of the right ventricle (EFRV), minute (MV) volumes, heart rate (HR) were determined. The study of NT-proBNP in blood was carried out by the method of competitive immunoassay on the analyzer IMMULITE 2000, Siemens. Statistical processing of the obtained data was carried out using the SPSS17 software package (license 4a180844250981ae3dae-s/nSPSS17) on the PC / Pentium-4 computer.

**Results:** The level of EFRV in patients with RD was significantly lower than in adolescents in the control group (46.99 ± 2.92 % versus 58.51 ± 1.77 %; p < 0.001), but at the same time, the minute volume (MVrv) in children of this group was higher than in children in the control group (MVrv 1.05 ± 0.10 l/min versus 0.48 ± 0.03 l/min; p < 0.001). The increase of MVRV in adolescents with RD was due to their higher heart rate (81.67 ± 2.37 beats/min versus 66.61 ± 1.65 beats/min; p < 0.001). The level of NT-proBNP in the blood of adolescent patients was significantly higher than in the control group (45.71 ± 6.39 pg/ml versus 18.38 ± 0.94 pg/ml; p < 0.001). Correlation relationships between RV systolic function and NT-proBNP level have not been established.

**Conclusion:** In adolescents with rheumatic diseases a significant decrease in the pumping function of the right ventricle of the heart was established with an increase in its minute volume due to the higher heart rate. This occurs against the background of a significantly increased level of natriuretic peptide in them.

**Disclosure of Interest**

None declared

### P175 Outcomes of intraarticular triamcinolone acetonide injection in children with non-systemic juvenile idiopathic arthritis

#### M. Sukharomana, S. Charuvanij

##### Division of Rheumatology, Department of Pediatrics, Faculty of Medicine Siriraj Hospital, Mahidol University, Bangkok, Thailand

###### **Correspondence:** M. Sukharomana

**Introduction:** Intraarticular corticosteroids injection is an adjunctive therapy in children with juvenile idiopathic arthritis (JIA). There are various types of corticosteroids used, depending on the availability. Some countries, like Thailand, have access only to triamcinolone acetonide (TA).

**Objectives:** To study the outcomes and adverse events of intraarticular TA injections in children with non-systemic JIA.

**Methods:** A retrospective cohort study of children with non-systemic JIA who received intraarticular TA injections from August 2010 to August 2018, with at least 6 months of follow-up period. Arthritis was defined as objective findings of joint swelling, or 2 out of 4 of the following: limited range of motion, tenderness along joint line, stress pain on end range of motions, and warmth. Response to intraarticular TA injection was defined as an absence of arthritis at 6 months after receiving the procedure. Response to intraarticular TA injection and adverse events were evaluated at 6, 12, 18, and 24 months following each procedure.

**Results:** Forty-five children with non-systemic JIA received intraarticular TA injections; 12 oligoarthritis (6 extended, 6 persistent), 8 rheumatoid factor (RF) positive polyarthritis, 3 RF negative polyarthritis, 19 enthesitis-related arthritis (ERA), and 3 unclassified JIA. There were 20 males (44.4%) and the median (interquartile range, IQR) age was 10 (5.7,11.7) years. The median (IQR) time from diagnosis to first joint injection was 28 (5, 243) days. One hundred seventy-seven joints were injected (57 knee joints, 40 ankle joints, 37 wrist joints, 18 proximal interphalangeal joints, 10 elbow joints, 10 metacarpophalangeal joints, 4 metatarsophalangeal joints, and 1 subtalar joint). The median number of joints injected per patient was 2 (IQR 1, 4), and the median number of joint injection episodes was 2 (IQR 1, 3). The dosage of TA was 2 mg/kg in 106 (60%), and 1 mg/kg in 32 (18.1%). A response to intraarticular TA injections was observed in 118 (66.7%) at 6 months, 113 (63.8%) at 12 months, 95 (53.7%) at 18 months, and 42 (23.7%) at 24 months. Ninety-four of 177 (53.1%) joints had arthritis flare-up following intraarticular TA injection, with the median time at 4 (IQR 2.6, 8.6) months. Patients with ERA had a median (IQR) time to arthritis flare-up at 3 (IQR 0.9, 5.2) months. Patients with oligoarthritis had a median (IQR) time to arthritis flare-up at 5.9 (IQR 1.4, 10.4) months. There were significant differences of time to arthritis flare-up between ERA subtype (median 3 months, IQR 0.9, 5.2) versus non-ERA subtypes (median 5.1 months, IQR 1.5, 8.6) at *p*=0.007. Local adverse effects following intraarticular TA injections included pigmentary changes in 3 (1.7%), cutaneous atrophy in 2 (1.1%), and radiographic calcification in 1 (0.6%). There were no crystal-induced arthropathy, septic arthritis, or any systemic adverse reactions.

**Conclusion:** Intraarticular TA injection had a favorable response in two-thirds of injected joints at 6 months among children with non-systemic JIA. The ERA subtype had the earliest arthritis flare-up following the intraarticular TA injection. Adverse reactions from the procedure were uncommon.

**Disclosure of Interest**

None declared

### P176 Damage and disability in children with juvenile idiopathic arthritis: a study from Thailand

#### S. Tangcheewinsirikul, M. Sukharomana, S. Charuvanij

##### Division of Rheumatology, Department of Pediatrics, Faculty of Medicine Siriraj Hospital, Mahidol University, Bangkok, Thailand

###### **Correspondence:** S. Tangcheewinsirikul

**Introduction:** Juvenile idiopathic arthritis (JIA) is the most common cause of childhood-onset chronic arthritis. The chronic nature of JIA increases the likelihood of articular and extra-articular complications that can have damaging and debilitating effects in children with JIA.

**Objectives:** The aim of this study was to investigate the prevalence of different types of JIA-related articular and extra-articular damage and disability, and to identify factors independently associated with damage and disability in Thai children with JIA.

**Methods:** During June 2019 to June 2020, this prospective cross-sectional study enrolled Thai children aged ≤18 years who were diagnosed with JIA according to the International League of Associations for Rheumatology criteria and who had at least 6 months of available follow-up data. Patients were recruited from the Division of Rheumatology of the Department of Pediatrics, Faculty of Medicine Siriraj Hospital, Mahidol University, Bangkok, Thailand. Siriraj Hospital is Thailand’s largest adult and pediatric national tertiary referral center. Damage was evaluated using the Juvenile Arthritis Damage Index (JADI) and the modified-JADI (mJADI) assessment tools. Disability was assessed using the Child Health Assessment Questionnaire (CHAQ) and Steinbrocker classification criteria. Occurrence of macrophage activation syndrome (MAS) and infection were also reviewed.

**Results:** Eighty-two patients with JIA (42 female, 40 male) were included in this cohort. The median (interquartile range; IQR) age was 12 (8.7-14.3) years. The median (IQR) disease duration was 36.8 (16.3-62.1) months. Enthesitis-related arthritis (ERA) was the most common subtype (31.7%), followed by systemic JIA (28.0%) and oligoarticular JIA (15.9%). Twenty-eight of 82 patients (34.1%) had articular damage as evidenced by a JADI-articular (JADI-A) or mJADI-A score of ≥1. Limitation of motion at the lumbar spine was the most common site of damage (12.2%), followed by the wrist joint (11.0%). Extra-articular complications were observed in 23 (28.0%) patients as demonstrated by a JADI-extra-articular (JADI-E) or mJADI-E score of ≥1. Striae was the most commonly observed (9.8%) extra-articular complication, followed by growth failure (8.5%). Ocular damage was found in 1 patient with ERA. The prevalence of uveitis was a low 1.2%. Cataract and glaucoma were detected in 4.9% and 2.4% of JIA children, respectively. MAS was identified in 21.7% of patients with systemic JIA. Aortitis was documented in 1 patient with ERA. Infection occurred in 15 (18.3%) patients, and most of those had systemic JIA subtype. Most (86.6%) patients were classified as Steinbrocker functional class I. Moderate-to-severe disability (CHAQ score ≥0.6) was found in 15 (18.3%) patients. Multivariable logistic regression analysis revealed Steinbroker functional classification greater than class I (adjusted odds ratio [aOR]: 7.83, 95% confidence interval [CI]: 1.60-38.33; *p*=0.011), and delayed diagnosis longer than 6 months (aOR: 6.95, 95% CI: 2.30-21.04; *p*=0.001) to be factors independently associated with articular damage. Limited joint counts (*r*_s_=0.710, *p*<0.001) and duration of delayed diagnosis (*r*_s_=0.488, *p*<0.001) significantly correlated with JADI-A scores.

**Conclusion:** One-third of Thai children with JIA had disease-related damage, and restricted lumbar spine motion was the most common type of articular damage. Steinbroker functional classification greater than class I and duration of delayed diagnosis were the independent predictors of articular damage. The findings of this study highlight the need for improved early detection and diagnosis to prevent or reduce permanent damage in Thai children with JIA.

**Patient Consent Received**

No

**Disclosure of Interest**

None declared

### P177 How ready are adolescents and their parents for transition from pediatric rheumatology care to adult rheumatology care?

#### A. Vermé, J. Granhagen Jungner, K. Palmblad, E. Weidenhielm Broström, C. Bartholdson

##### Department of Women´s and Children´s Health, Karolinska Institutet, Stockholm, Sweden

###### **Correspondence:** A. Vermé

**Introduction:** In Sweden, approximately 1500-2000 children suffers from Juvenile Idiopathic Arthritis (JIA)^1^. The transfer from pediatric rheumatology care to adult rheumatology care is often perceived as difficult and creates a great deal of concern for the adolescents and their parents.^2^ To facilitate the transition process, it´s important that both the adolescents and their parents have good knowledge about the disease and treatment.^3^ If the adolescent has good knowledge about the disease and treatment it increases the opportunity to take an active role when visiting the rheumatology clinic and to take a greater reasonability for the disease and treatment^3^.

**Objectives:** The purpose of the study is to investigate how ready adolescents with Juvenile Idiopathic Arthritis (JIA) are to transfer to adult care and to take responsibility for their own health. The purpose is also to investigate parent’s perspectives in these issues.

**Methods:** Members, aged 14-18, of Young Rheumatic’s, Sweden was invited to participate in the study by responding to the questionnaire “Readiness for transition questionnaire”.^4^ All patients at the pediatric rheumatology clinic at Astrid Lindgren´s Children´s hospital in Stockholm Sweden aged, 14-18 also received an invitation to participate in the study. Parents of these adolescents were also invited to participate.

**Results:** During the period April 2020 to March 2021, 59 adolescents (girls n= 58, boys n= 10, did not state gender=1) and 58 parents (women n= 44, men n= 14) answered the questionnaire. Preliminary result demonstrates that about half of the adolescents responded that they were the ones taking the greatest responsibility for their health but only 24% of the parents experienced the same. Furthermore, adolescents take responsibility to book visits to the pediatric rheumatology clinic to a small extend, 52% of the adolescents and 54% of the parents stated that the adolescents didn’t take any responsibility at all for booking visits. Furthermore, the result show that 48% of the adolescents experienced that they took responsibility to talk to the staff when visiting the clinic, the parents had the same experience. Half of the parents felt that their adolescents was not at all ready to transfer to adult care compared to almost half (46%) of the adolescents. Very few of the adolescents (4%) felt completely ready to be transferred to adult care, which corresponded the parent’s experiences (2%).

**Conclusion:** The result demonstrates that very few of Swedish adolescents with juvenile arthritis felt ready to transfer to adult care. Adolescents need help and support to be able to increase their participation in their own care. To be able and to dare to participate knowledge is needed. One possible way to meet those needs is to introduce a structed ready for transition program in pediatric rheumatology care in Sweden.

**Patient Consent Received**

No

**Disclosure of Interest**

None declared

## e-Poster viewing: Treatment

### P178 Efficacy and safety of tocilizumab (Intravenous and subcutaneous) in patients with systemic and polyarticular juvenile idiopathic arthritis: short term results of the prospective study

#### S. Caglayan, K. Ulu, T. Coşkuner, F. Demir, B. Sözeri

##### Pediatric Rheumatology, Umraniye Training and Research Hospital, Istanbul, Turkey

###### **Correspondence:** S. Caglayan

**Introduction:** Juvenile idiopathic arthritis (JIA) is one of the most common chronic rheumatic diseases of childhood. Polyarticular JIA (pJIA) can cause joint damage in children, leading to serious disability. Systemic JIA (sJIA) is a subgroup of JIA that is characterized by autoinflammatory and autoimmune disease features, difficult to treat, and can lead to mortality. Tocilizumab (TCZ) is an interleukin 6 (IL-6) receptor antagonist used to treat moderate to severe active JIA. Both intravenous (IV) and subcutaneous (SC) routes are approved for the treatment of children.

**Objectives:** This study aimed to assess the efficacy and safety of SC TCZ versus IV TCZ, including switching formulations, in patients with juvenile idiopathic arthritis.

**Methods:** We prospectively reviewed all patients diagnosed with JIA and receiving at least one dose of TCZ in a single pediatric tertiary rheumatology center from Turkey between June 2016 and January 2021. Disease activity was assessed by Jadas-27 ESR at baseline, week 12 (W12), W24 and W48. Severe adverse events (SAE) were defined as a life-threatening event and/or an event requiring hospital admission, leading to permanent disability or treatment discontinuation.

**Results:** A total of 47 patients (34 females, 13 males ) who were 32 systemic JIA (68,1%) and 15 polyarticular JIA (31,9%) were included in the study. The mean age of the patients at diagnosis was 7.8 ± 4 years and the median age at the onset of TCZ was 10.4 years (IQR 6.2 - 14 years). All patients received disease-modifying antirheumatic drugs (methotrexate or sulphasalazine) at baseline. Most children had received prednisone (89.4%, N=42), and/or a biologic agent (51%, N=24) before TCZ. After TCZ treatment, 46.8% of all patients achieved inactive disease and steroid was discontinued in 11 (26,1%) of 42 patients who were taking steroids. Decrease in disease activity as measured by the JADAS scores was observed higher in biologic naive patients compared to biologic exposed patients at 3 months and 6 months median change: -14.3 vs-10.15 (p=0.032), and median change: -20 vs -12 (p=0.05), respectively. The median JADAS-27 score of 40 patients treated with tocilizumab-iv decreased from 21 at baseline (IQR, 17-24.6) to 4 (IQR, 0-8) at week 48. Twenty-three patients were used subcutaneous tocilizumab, 16 of them after intravenous therapy, and 7 of them directly. The median JADAS-27 score of these patients decreased from 7 at baseline (IQR, 0.5-15.2) to 0 (IQR, 0-5) at week 48. SAE was observed in 13 (27.6%) patients (SAE 6.7/100 patient-years), including infections, autoimmune disease, macrophage activation syndrome, and infusion reaction. No cases of malignancy or death were reported while under TCZ treatment. Throughout the treatment period, 20 patients experienced 28 exacerbations, mostly within the first 6 months (57.1%). At the last follow-up, 16 (34%) children remained on TCZ.

**Conclusion:** In this cohort, Tocilizumab (TCZ) is effective and well-tolerated in both SC and IV in the long-term treatment of children with both pJIA and sJIA, including switching formulations.

**Disclosure of Interest**

None declared

### P179 Safety of colchicine treatment in pediatric autoinflammatory patients, followed in a single center

#### T. Coşkuner^1^, F. Kavas Coşkuner^2^, K. Ulu^1^, N. Gerenli^2^, S. Çağlayan^1^, T. İbiş^2^, C. Çeltik^2^, F. Demir^1^, B. Sözeri^1^

##### ^1^Pediatric Rheumatology; ^2^Pediatric Gastroenterology, University of Health Sciences, Umraniye Training and Research Hospital, Istanbul, Turkey

###### **Correspondence:** K. Ulu

**Introduction:** Colchicine has been used for the management of, familial Mediterranean fever (FMF), other autoinflammatory diseases (AID)Behcet’s disease for decades in children. Colchicine therapy can result in diarrhoea and other gastrointestinal adverse effects. Long-term colchicine therapy is rarely associated with myopathy, polyneuropathy, transaminitis, and rhabdomyolysis.

**Objectives:** In this study, we aimed to investigate the frequency of colchicine side effects and risk factors in pediatric patients. We also analyzed the relationship between primary diseases and other drugs used for them.

**Methods:** We included 806 consecutive patients treated with colchicine for ≥ 6 months. The patients were obtained from database of autoinflammatory diseases, Umraniye Training and Research Hospital, Department of Pediatric Rheumatology, were included in the study. Laboratory investigation was performed before eachpatient’s last visit to the clinic. The clinical, demographic,genetic variables, and colchicine dose were analyzed for correlationwith colchicine-adverse reactions. The ethical committeeof our institute approved the study protocol.

Statistical Analyses

Results are given as a mean- SD or proportion as appropriate. Differences between the groups in discrete variableswere evaluated by chi-square or Fisher exact test. Comparisonsof continuous variables were done by unpairedStudent t test or Wilcoxon 2-sample test, as required. AllP values given are 2-sided. P values less than 0.05 were consideredsignificant.

**Results:** 806 patients (407 female, 399 male) were included in the study. A number of disease states were studied including FMF (n=721,89.5%), Behcet disease (n=49, 6.1%), other AIDs (n=36, 4.5%). There were CAPS (n=3), HIDS (n=2), TRAPS (n=2), and PFAPA syndrome (n=29) in other AIDs.

The mean age at diagnosis of disease was 7.15 ± 4.17 years (7 months-17 years), the mean initiation age of colchicine therapy was 17 ±7.29 years (7 months-6.73 years). In our cohort, colchicine resistance was determined in 44 patients (6.1%).

The median colchicum exposure timewas 41months (IQR, 24-71). Adverse events due to colchicine were detected in 8.6% of the patients (n=67). Severe adverse event was not seen in any patients. No relation was found between gender, presence of comorbid disease, use of other drugs and side effects in all patient group.

Main adverse events were as follows: diarrhea in 30 patients(44.7%) and elevation in transaminases in 23(34.3%). Neither GIS nor liver toxicity could be correlated with the dose of colchicine in the patients included in the study. Although and the median treatment initiation age of patients with colchicine-related adverse effects were lower than the others, there was no statistically significant difference in FMF patients (5 vs 6 years, P=0.06). In contrast, adverse effects were observed in the older age group in the Behcet disease group (median age =16.6 vs 12.3 years, P =0.03).

Among the patients with adverse effects, 21 patients (31.3%) did not change the treatment plan, while the dose was reduced in 21 patients (31.3%), colchicine was discontinued for a short time in 17 patients (25.4%) and permanently in 5 patients (7.5%). In 3 patients, they were switched to colchicine opocalcium.

**Conclusion:** Colchicine treatment (median 4 years) has been found to be very safe even in younger age groups. Regardless of pre-existing disease, gender and age, the most common side effects are diarrhea and transient elevation of transaminases, which can be controlled by a decrease in colchicine dose. The difference detected in Behçet's patients’ needs confirmation in larger study groups.

**Patient Consent Received**

Yes

**Disclosure of Interest**

None declared

### P180 Therapeutic drug monitoring in children with juvenile idiopathic arthritis (JIA) with or without uveitis and non-jia uveitis treated with adalimumab: a single centre retrospective study

#### T. Doudouliaki^1^, S. Compeyrot-Lacassagne^1^, C. Ciurtin^2,3^, A. L. Solebo^4,5,6,7,8^, H. Petrushkin^6,9^, L. Wedderburn^2,10^

##### ^1^Rheumatology, Great Ormond Street Hospital; ^2^Centre for Adolescent Rheumatology Versus Arthritis at UCL, UCLH/GOSH; ^3^Centre for Rheumatology Research, Division of Medicine, UCL; ^4^Population, Policy, Practice Research and Teaching Department; ^5^Ulverscroft Vision Research Group, UCL GOS Institute of Child Health; ^6^Great Ormond Street Hospital for Children NHS Trust; ^7^National Institute for Health Research Biomedical Research Centre, Great Ormond Street Hospital and UCL GOS ICH; ^8^National Institute for Health Research Biomedical Research Centre, Moorfields Eye Hospital NHS Foundation Trust and UCL Institute of Ophthalmology; ^9^Moorfields Eye Hospital NHS Foundation Trust; ^10^UCL Great Ormond Street Institute for Child Health (ICH), UCL, London, United Kingdom

###### **Correspondence:** T. Doudouliaki

**Introduction:** The use of biologic medications has revolutionized the management of autoimmune conditions in children, especially Juvenile Idiopathic Arthritis (JIA). Despite better disease control achieved for most patients, there are still a significant number that will not respond to treatment or will present with secondary loss of response. Therapeutic Drug Monitoring (TDM) comprises both the measurement of serum drug levels and antidrug antibodies (ADAb). In Paediatric Rheumatology, TDM is not yet widely implemented and drug levels are measured in a reactive manner, when the desired treatment outcome is not achieved.

**Objectives:** To describe data for patients who are or have been on treatment with Adalimumab (ADAL) and had drug and ADAb levels already measured in one large Paediatric Rheumatology centre, in order to investigate the association between TDM and clinical response.

**Methods:** Retrospective review of records of patients with a diagnosis of JIA, uveitis associated with JIA (JIAU) and non-JIA uveitis currently or previously on treatment with ADAL. Data were gathered on available serum drug and ADAb levels, disease activity, concomitant use of Disease Modifying Antirheumatic Drugs (DMARDs), patient age and disease duration at the time of serum sampling.

**Results:** Twenty-six out of 246 patients currently on ADAL and 7 out of 230 patients who had been on ADAL were identified to have available ADAL and ADAb serum levels measured at least once.

Seventeen patients had a diagnosis of JIA and JIAU, 11 patients had JIA only and 5 patients non JIAU. Twenty-three patients were on concomitant DMARDs [17 patients on Methotrexate (MTX), 5 on Mycophenolate Mofetil, (MMF), 1 on Sulphasalazine], while 10 patients were on ADAL monotherapy. Inactive JIA was defined as no active joint count and/or no EMS and no uveitis. Inactive uveitis was defined as quiet eyes on less than 1 daily steroid eye drop.

Twenty nine patients had active disease (87.9%). This suggests that drug monitoring was reactive. Median drug level was 7.9 mg/L (0.9-10.7), median antibody level was 6 AU/ml (0-200). 17 patients (51.5%) had detectable ADAb.

There was a significant correlation between low drug level and the presence of ADAb (p<0.05) and between the absence of DMARD and the presence of ADAb (p=0.032). Disease activity is also correlated with low drug level (p: 0.028).

**Conclusion:** This small real-life pilot study suggested a correlation between disease activity, and low anti-TNF drug levels with detectable ADAb. TDM may contribute to the optimisation of patient care, leading to a personalised medicine approach but for the time being, there are no standardized therapeutic drug levels to aim for. The next goal will be to set optimal drug levels to be targeted on induction and maintenance treatment for patients on ADAL.

**Disclosure of Interest**

None declared

### P181 Adalimumab-induced sapho syndrome: a case report

#### M. Fastiggi^1^, C. Sembenini^1^, M. E. Zannin^1^, F. Dell'Apa^1^, C. Giraudo^2^, A. Meneghel^1^, G. Martini^1^, F. Zulian^1^

##### ^1^Department of Women’s and Children’s Health; ^2^Institute of Radiology, University of Padua, Padua, Italy

###### **Correspondence:** M. Fastiggi

**Introduction:** TNF alpha inhibitors (TNFi), are widely used to treat juvenile idiopathic arthritis (JIA) and usually well tolerated. Among the side effects of these agents, many paradoxical events have been reported such as psoriasiform skin reactions, uveitis and granulomatous diseases (sarcoidosis and Crohn’s disease)^1^. Herein, we report the case of 16-years-old girl with oligoarticular JIA (oJIA) who developed SAPHO syndrome (synovitis, acne, pustulosis, hyperostosis and osteitis) as a paradoxical adverse event during adalimumab treatment.

**Objectives:** Describe the first case of ADA-induced SAPHO-like syndrome in a paediatric patient with JIA.

**Methods:** Case report.

**Results:** A 14 years-old girl affected by oJIA since the age of 2 years with recalcitrant uveitis was started on adalimumab (ADA) at a standard dose of 40 mg every 2 weeks with progressive improvement of ocular inflammation. Seven months later, the patient presented swelling and pain of the proximal part of left clavicle associated with palmar-plantar pustular rash. She had no history of trauma or recent infections. Laboratory test showed a mild elevation of CRP (7,5 mg/L) and ESR (62 mm/h) with normal white blood-cell count. X-rays showed enlargement of the medial third of the left clavicle with irregular structure with periosteal reaction and, in the same site, MRI showed a spongy bone oedema with osteitis and intense periosteal reaction. Tc^99^ bone-scan ruled out other possible sites of bone involvement. Considering the close temporal relationship with the start of ADA, the working diagnosis was ADA-induced SAPHO syndrome. ADA was therefore discontinued and the skin lesions were successfully treated with topical corticosteroids. The bone symptoms did not improve with non-steroidal anti-inflammatory drugs (Naproxen) therefore, 3 monthly infusions of pamidronate were administered with success. Six months later MRI showed a reduction of cortical bone thickening and oedema of the clavicular spongy bone. Because of poor control of ocular inflammation with just MTX, 15 months later, abatacept was added. Almost three year since Sapho syndrome onset, the patient is on stable articular, ocular and bone remission.

**Conclusion:** Since the introduction of biological agents, such as TNFi, treatment options have increased in children with JIA and its prognosis has significantly improved. However, unexpected side effects have been reported, particularly dermatological, intestinal and ophthalmological paradoxical adverse events (PAEs)^3^. To our knowledge, this is the first case of ADA-induced SAPHO-like syndrome in a paediatric patient with JIA. Our experience also suggests that Abatacept may represent an effective treatment option in patients requiring biological treatment in case of PAEs secondary to TNFi.

**References**

1) Wendling D et al. Paradoxical effects of anti-TNF-α agents in inflammatory diseases. Expert Rev Clin Immunol. 2014 Jan;10(1):159-69.

2) Kahn MF et al. The SAPHO syndrome. Baillieres Clin Rheumatol 1994;8:333–62.

3) Toussirot É et al. Paradoxical reactions under TNF-α blocking agents and other biological agents given for chronic immune-mediated diseases: an analytical and comprehensive overview RMD Open 2016 Jul 15;2(2):e000239.

**Patient Consent Received**

Yes

**Disclosure of Interest**

None declared

### P182 Review of corticosteroid induction protocols used for children with a new diagnosis of polyarticular course juvenile idiopathic arthritis (PJIA) in an east of england tertiary rheumatology service

#### C. Foley, P. Bale, K. Armon

##### Addenbrooke's Hospital, Cambridge, United Kingdom

###### **Correspondence:** C. Foley

**Introduction:** Children with pJIA present with five or more joints affected by pain, swelling and stiffness. Untreated, joint inflammation can lead to irreversible joint damage and disability. Corticosteroids have been used for treatment of JIA since the 1950s. Current clinical practice for treatment of pJIA involves high-dose corticosteroids for a limited period in order to decrease inflammation. The aim is to induce remission whilst systemic treatment, commenced alongside corticosteroids begins to work. No standardised, evidence-based approach currently exists to guide corticosteroid induction regimens in pJIA.

**Objectives:**

1. Describe corticosteroid regimens in children newly diagnosed with pJIA.
2. Compare disease activity at diagnosis, with follow-up review after treatment with corticosteroids.

**Methods:**

Retrospective chart review of children newly diagnosed with pJIA, January 2019 to December 2020, inclusive. Demographic data collected and steroid regimens documented. Disease activity recorded pre-instigation of corticosteroids, and then at a follow-up appointment on average 5.5 weeks into treatment (range 3-12 weeks). Modified JADAS-27 created using active joint count (AJC), C-reactive protein (CRP) and ESR, as physician and patient/parent global assessment scores were missing from the majority of charts. Total score achievable using modified JADAS-27 (mJADAS-27)=47.

**Results:** Sixteen-children were diagnosed with pJIA between January 2019 and December 2020, (male = 5,15%). Eleven-children (69%) had polyarticular RF-negative JIA (RF-), 3(19%) polyarticular RF-positive JIA (RF+), 1(6%) Psoriatic-JIA (PsA) and 1(6%) HLA-B27 positive enthesitis-related arthritis (ERA). All children were commenced on non-steroidal anti-inflammatory drugs (Naproxen, n=11; Ibuprofen, n=5) and subcutaneous Methotrexate (15mg/m^2^) alongside corticosteroids.

A three-day course of intravenous methylprednisolone (ivMP) was the initial corticosteroid of choice in 12/16(75%) children. Six children were given a dose of 30mg/kg (maximum 1gram), two children 20mg/kg and four 500mg (weight 30.9-44.2 kg; two children were on oral prednisolone (POPred) prior to admission for ivMP; one child had T1DM). Following 3-days of ivMP, all 12 children were commenced on POPred.

The children that did not receive ivMP were commenced on POPred at a dose of 0.5-1mg/kg, with an initial weaning plan of 5mg/week. Two of these children had RF+, one had ERA and the other PsA. AJC ranged from 5-12.

Starting dose of POPred following 3-days of ivMP ranged from 7.5-40mg, maximum dose 1mg/kg. Weaning instructions varied from 5mg/week (n=6), 2.5mg/week (n=4) or stay on low dose (<0.25mg/kg) until review (n=6).

Median mJADAS-27 pre-corticosteroids was 19.4 (5-43.5). Follow-up mJADAS-27 was calculated about 5.5 weeks (3-12) into corticosteroid treatment. Median follow-up mJADAS-27 was 3.5 (0-8). On average, mJADAS-27 improved by 81% (0-100%) following corticosteroids. Of the four children that did not receive ivMP, one child RF- had 100% improvement in mJADAS-27, the other experienced no improvement. The child with PsA experienced 86% improvement; the child with ERA, only a 20% improvement.

**Conclusion:**

Corticosteroids lead to improved disease activity in children with pJIA. However, treatment regimens employed vary. Development of a standard operating procedure for corticosteroid induction in pJIA is required. Longitudinal studies would enable evidence-based development of such protocols, and should consider optimal corticosteroid route of administration and dose to achieve maximal benefit, whilst minimising corticosteroid toxicity.

**Disclosure of Interest**

None declared

### P183 Haematological side effects due to anti-TNF therapy in pediatric rheumatology patients

#### M. Kasap Cuceoglu^1^, E. Sag^1^, D. D. Gumus^2^, S. Demir^1^, U. Kaya Akca^1^, E. Atalay^1^, S. Sener^1^, Z. Balik^1^, O. Basaran^1^, E. D. Batu^1^, Y. Bilginer^1^, F. Gumruk^3^, S. Ozen^1^

##### ^1^Pediatric Rheumatology; ^2^Department of Pediatrics; ^3^Pediatric Haematology, Hacettepe University, Ankara, Turkey

###### **Correspondence:** M. Kasap Cuceoglu

**Introduction:** Despite early intensive treatment with DMARDs (Disease -Modifying Antirheumatic Drugs) for the last twenty years, many pediatric patients are followed up with chronic active disease in the rheumatic diseases such as juvenile idiopathic arthritis (JIA), Chronic nonbacterial osteomyelitis (CNO), Uveitis. Therefore, biological drugs have been used in the treatment of childhood rheumatic diseases in order to decrease the frequency of chronic sequelae and achieve complete remission. Biological drugs are effective in various pathogenetic pathways. Therefore, the choice of medication should be based on the subgroup of the disease. Anti-TNF agents are one of the most commonly used drugs in practice. It is important to know the side effects of these drugs in order for the treatment to be effective and continuous.

**Objectives:** Anti-TNF agents are used for the treatment of various rheumatologic diseases. We aimed to study the hematological side effects of anti-TNF agents, including infliximab, adalimumab, and etanercept.

**Methods:** We evaluated the patients followed up between December 2019 and December 2020 in the Department of Pediatric Rheumatology in Hacettepe University, Ankara, Turkey, retrospectively. 93 pediatric patients who were treated with anti-TNFs were included in the study. The demographics, follow up duration on treatment, dose, drug side effects were analyzed. Patients with anemia, thrombocytopenia, and leukopenia (Hb<10.5 mg/dl, WBC<4000 /uL, plt<250.000/mm^3^) at drug initiation were excluded from the study.

**Results:** 93 pediatric rheumatology patients including 42 juvenile idiopathic arthritis, 29 enthesitis-related arthritis, four CRMO, six DADA2, one PAPA, two UAID, one Behçet' s disease, and 2 psoriatic arthritis were included in this study. The mean age of the study cohort was 14.7±4.1 years. Among our patients, 59 of them (63%) were treated with etanercept, 28 of them (30%) with adalimumab, and six patients (6%) were treated with infliximab. No allergic reactions to adalimumab and etanercept were detected. On the other hand, one patient developed a hypersensitivity reaction against infliximab; thus the treatment was switched to adalimumab.

Among all patients, 11 of them (11.8%) had thrombocytopenia, seven (7.5%) of them had leukopenia, and only one of them (1%) had anemia during anti-TNF treatment. 23.7 % of the patients treated with etanercept (15.2% had thrombocytopenia, 6.7% had leukopenia and 1.6% had anemia), %16.6 of the patients with infliximab (thrombocytopenia) and 14.2 % of the patients with adalimumab (10.7% had leukopenia and 3.5% had thrombocytopenia) developed drug-related hematological side effects during follow-up. The mean total duration of anti-TNF drug treatments were 4.6 ± 2.1 years, 5.1 ± 2.5 years, 3.7 ± 0.5 years, for etanercept, adalimumab, infliximab, respectively.

Among those who developed hematological side effects, the mean delay between the onset of biologic agent and the onset of the side effects were 11.5 months (range=3-33) for leukopenia and 29.4 months (range=13-48) for thrombocytopenia. And it was one month who developed anemia with etanercept.

**Conclusion:** Anti-TNF agents are being used effectively in daily pediatric rheumatology practice. In this study, we observed that anti-TNF drugs could affect the blood cells, with etanercept bearing the greatest risk in our population.

**Patient Consent Received**

No

**Disclosure of Interest**

None declared

### P184 Steroid intra-articular injection in children with oligoarticular onset juvenile arthritis: factors associated with a poor response

#### A. Kozhevnikov^1,2^, N. Pozdeeva^1^, S. Bogdanova^1^, G. Novik^2^

##### ^1^H.Turner National Medical Research Center for Сhildren's Orthopedics and Trauma Surgery; ^2^Saint-Petersburg State Pediatric Medical University, Saint-Petersburg, Russian Federation

###### **Correspondence:** A. Kozhevnikov

**Introduction:** Intra-articular corticosteroid injections are the first-line antirheumatic drugs of oligoarticular onset juvenile arthritis*.* Despite significant advances in treatment (anti-TNF, block IL6) the choice DMARDs of pediatric chronic joint disease still remains relevant. Pediatric rheumatologists and to this day there are no consensus on the best modality for treatment.

**Objectives:** The aim of this study was to search of predictive biomarkers of ineffective treatment by early intra-articular steroid injections in oligoarticular onset juvenile arthritis*.*

**Methods:** Efficacy of early isolated intra-articular injections (is-IAI) of triamcinolone acetonide in 120 children (met ILAR criteria for JA; 89% girls /11% boys) aged median (IQR) 4,2 (1,6 – 7,6) years with oligoarticular onset juvenile arthritis without extra-articular manifestations (oligo-JA) were collected retrospectively and analyzed*.* Clinical data, radiology and laboratory results (blood and synovial fluid) were evaluated. Triamcinolone acetonide (TA) was administered intra-articular at a dose of 20-40 mg with an injection interval of 3-6-12 months which was depended on the activity of the disease. All children were divided into two groups: active / inactive arthritis based on the effectiveness of is-IAI. The average follow-up was 48 [38; 62] months.

**Results:** 42 children (35%; all girls) were achieved remission oligo-JA after is-IAI of TA with mean of 2 injection per joint (inactive arthritis > 24 months)*.* The mean interval between two consecutive is-IAI was 7 [5,25; 10] months. Other children (54% girls; 11% boys) did not achieve inactive oligo-JA after is-IAI of TA with mean of 3 [2; 4] injection per joint. The mean interval between first two consecutive injection was 5,5 [4,25; 7] months and other injections *-* 2 [2; 3] months. All children who did not achieve remission of oligo-JA for is-IAI were treated by DMARDs*.* Statistical analyses were performed to determine the relationships between clinical, instrumental, laboratory signs and efficacy is-IAI of TA*.* In addition to routine studies measures were included cJADAS10 and biological inflammatory markers [interleukin 6 (IL6), tumor necrosis factor alfa (TNF-α) and calprotectin in serum and synovial fluid]. Efficacy is-IAI of TA was no associated significantly with number of active joint of onset oligo*-*JA*,* cJADAS10, titer of ANF, serum level of CRP mg/ml, ESR mm/h, IL6 pg/ml TNF-α pg/ml and Calprotectin. The mean inflamed synovial fluid of IL6 levels 2208 [710; 4564] / 3234 [1265; 16902] pg/ml and TNF-α levels 3,3 [2,5; 3,8] / 1,1 [0,6; 3,7] pg/ml at onset of inactive and active oligo-JA were not significantly differ. The analysis revealed a correlation between a short phase of beneficial effect after is-IAI of TA and risk of activity disease *(*with an inactive phase of arthritis less than 3 months, the risk activity was OR = 2.09, p <0.001; with an inactive phase less than 2 months - OR = 8.9, p <0.001).

**Conclusion:** TA is an effective and safety treatment in children with oligoarticular juvenile arthritis. Research was revealed that about a third of children with oligo-JA achieved inactive arthritis of average after two intra-articular injections of TA (all girls)*.* There are no biomarkers for prediction of poor treatment response in oligo-JA to early steroid injections. But a short phase of beneficial effect after is-IAI of TA may be sign of risk activity disease. In addition boys with oligoarticular onset juvenile arthritis may be considered like potentially ineffective for local steroid therapy*.*

**Disclosure of Interest**

None declared

### P185 Pigmented villonodular synovitis in children: experience of surgical treatment

#### A. Kozhevnikov^1,2^, N. Pozdeeva^1^, M. Nikitin^1^, V. Zorin^1^, Y. Proshenko^1^, G. Novik^2^

##### ^1^H.Turner National Medical Research Center for Сhildren's Orthopedics and Trauma Surgery; ^2^St. Petersburg State Pediatric Medical University, Saint-Petersburg, Russian Federation

###### **Correspondence:** A. Kozhevnikov

**Introduction:** Pigmented villonodular synovitis (PVNS) is a rare pseudoneoplastic origin of synovial membrane disease characterized by unrestrained proliferation of mononuclear cells. CSF-1/CSF-1R – dependent signaling drives mechanism of proliferation (Molena B. et al 2011, Takehiro Ota et al, 2015). Results of proliferative hyperactivity are formed multinucleated giant cells, foam cells and hemosiderophages with thickened synovial membrane to nodules and greatly elongated villious leading to damage joint. Usually only one joint is affected of PVNS, the knee is the most common site involved in 80 percent of cases. There are localized (tenosynovium, giant cell nodule) and diffuse form. There is not a standard method of treatment for PVNS, especially in pediatric patients. Use of radiotherapy in children is controversial due to the possibility of post-irradiation sarcoma.

**Objectives:** The aim of this study was to investigate of feasibility and efficacy of surgical treatment of PVNS in children.

**Methods:** Retrospectively were analyzed clinical history, histologic features and radiographic images of 25 children with PVNS (9-17 years old, boys ≈ girls). 10 children were with single nodular, 14 - diffusing form of PVNS. Chronic monoarthritis of knee had 87% (diffuse, nodular), elbow - 12% (diffuse), ankle – 4% (nodular) children. All children were insensitive to anti-rheumatic therapy (methotrexate, steroids, anti-TNF). Surgical treatment of PVNS was removal of the nodule by means of arthroscopy and total synovectomy or synovial capsulectomy in case of diffuse form. PVNS was confirmed by the results of the characteristic histological picture of the pathology.

**Results:** During 5-year follow-up period after orthopedic surgery relapse of PVNS was noted in two children with a diffuse form. A feature of these forms of PVNS was the total involvement of the synovium, including the posterior area of the knee. There were no relapses among children with nodular PVNS. A specific feature of the recovery period after orthopedic-surgical treatment of children with PVNS of the elbow joint was the formation of yearly arthrosis-arthritis with reduced range of motion.

**Conclusion:** PVNS is a rare condition in pediatric rheumatology. Arthroscopy - useful method of treatment nodular PVNS in children. The treatment choice of diffuse form PVNS is the synovectomy, despite frequent results with joint function impairement. Systemic targeting the CSF1/CSF1R axis like treatment may be proposed in children with diffuse, relapsed, or multifocal PVNS.

**Patient Consent Received**

No

**Disclosure of Interest**

None declared

### P186 Safety of mycophenolate mofetil in pediatric patients with chronic rheumatic disorders: a monocentric retrospective study

#### F. Barbati^1^, S. Ricci^1^, E. Marrani^2^, C. Azzari^1^, G. Simonini^2^

##### ^1^Pediatric Immunology; ^2^Pediatric Rheumatology, AOU Meyer, firenze, Italy

###### **Correspondence:** E. Marrani

**Introduction:** Mycophenolate Mofetil (MMF) is an immunosuppressive drug that in the last twenty years has been used for the treatment of different autoimmune rheumatological diseases, such as Systemic Lupus Erythematosus, Systemic Sclerosis, Polymyositis, Dermatomyositis, and Systemic vasculitis. Data on the immunological consequences of MMF therapy are still uncertain and not yet well identified.

**Objectives:** In order to test the hypothetical drug-induced hypogammaglobulinemia, the aim of this study was to report the trend of the immunoglobulin (Ig) values and of the infectious diseases in a cohort of children affected by systemic autoimmune diseases treated with MMF.

**Methods:** This observational study retrospectively evaluated a monocentric cohort of paediatric patients receiving MMF and affected by a chronic rheumatic disease, followed between December 2009 to February 2021 at the Pediatric Rheumatology Unit of Meyer Children’s University Hospital in Florence. Demographic, clinical and laboratory data were collected for each patient into a customized database.

**Results:** This observational study retrospectively evaluated a monocentric cohort of paediatric patients receiving MMF and affected by a chronic rheumatic disease, followed between December 2009 to February 2021 at the Pediatric Rheumatology Unit of Meyer Children’s University Hospital in Florence. Demographic, clinical and laboratory data were collected for each patient into a customized database.

**Conclusion:** In paediatric patients with chronic rheumatic diseases, immunological I level tests and serological analyses to screen the protection against the common childhood pathogens are suggested before starting an immunosuppressive drug. These patients should also complete the vaccination schedule.

According to our clinical observation, in patients treated with MMF a strict monitoring of Ig values is required during treatment and after discontinuation of the drug.

**Disclosure of Interest**

None declared

### P187 Alanine aminotransferase (ALT) elevation in methotrexate (MTX) treated juvenile idiopathic arthritis (JIA) patients

#### V. Mars, M. Doeleman, N. Wulffraat, J. Swart, S. de Roock

##### Pediatric Rheumatology, Wilhelmina Children's Hospital, Utrecht, Netherlands

###### **Correspondence:** V. Mars

**Introduction:** Most Juvenile Idiopathic Arthritis (JIA) patients use weekly methotrexate (MTX). The risk of MTX causing liver injury has been a great concern [1]. Due to potential hepatotoxicity, guidelines for monitoring MTX treatment were proposed by the American College of Rheumatology (ACR). The ACR guidelines recommend laboratory testing every 12 weeks. Especially alanine aminotransferase (ALT) is regarded as an important test to screen for liver damage. [2] The ACR guidelines have never been formally validated in children. Studies have suggested that hepatotoxicity and substantial abnormalities in liver enzymes are rare in JIA patients when compared to adults [3-5].

**Objectives:** To gain more insight into the outcomes of surveillance laboratory ALT testing in JIA patients and to assess predictors for ALT elevation in MTX treated JIA patients. This in order to progress towards a MTX monitoring guideline that reflects the specific risk of hepatotoxicity in JIA patients more accurately. Especially considering the burden of three monthly laboratory testing for pediatric patients and their families.

**Methods:** Analyses were made in a prospectively collected clinical database for 844 JIA patients. Follow-up was at least 3 months, regarded from the moment of first visit until the last available normal ALT test or the first ALT elevation. An ALT value higher than 1.5 times the upper limit of normal (ALT>1.5x ULN) was considered clinically relevant. The patient was marked as a MTX treated JIA patient, if they used MTX either orally or subcutaneously at the time of the first significant ALT elevation, or at the last normal ALT value if never significantly elevated. Mann Whitney U and Chi square tests were used for comparisons between patients with and without significant ALT elevation. Numbers needed to harm (NNH) were calculated using 2x2 contingency tables. Predictors for ALT>1.5×ULN were identified using univariate and multiple regression analysis in MTX treated JIA patients.

**Results:** 597 JIA patients were included with 6078 ALT tests. 452 (76%) of patients were ever treated with MTX and 253 (42%) patients were treated with MTX at the moment of an ALT test. ALT>1.5xULN was observed in 92 (15%) of all JIA patients and 65 (71%) of these were MTX treated JIA patients at the moment of ALT elevation. Therefore ALT>1.5xULN was observed in 65 (26%) of JIA patients treated with MTX at the moment of an ALT test. MTX treatment was significantly associated with ALT elevation (p=0.000). Associations were observed between ALT>1.5xULN and the female sex (p=0.005), younger age (p=0.018), lower BMI (p=0.005), oJIA and pJIA subtypes (p=0.021) and ANA positivity (p=0.042). The association with the female sex, JIA subtypes and ANA positivity were not found in the MTX treated patients subanalysis. A NNH of almost 6 JIA patients was calculated to be treated with MTX to achieve significant ALT elevation in one patient. No predictors could be identified for ALT>1.5×ULN in the MTX treated JIA patients subanalysis.

**Conclusion:** This study underlines the importance of the surveillance laboratory ALT testing in MTX treated JIA patients. A relatively high incidence of 15% in ALT elevation is found in all JIA patients, but with 26% in MTX treated JIA patients in particular. The NNH is almost 6 patients to be treated with MTX to achieve ALT elevation in one extra patient. No predictors for ALT elevation are identified in MTX treated JIA patients. The combination of high occurrence and difficult prediction, imply that surveillance ALT testing for all MTX treated JIA patients is more effective then hypothesized. Further research should be conducted to identify predisposing factors for elevated ALT and to explicitly evaluate the optimal frequency of ALT testing.

**Trial registration identifying number:** 1. Visser K, van der Heijde DM. Risk and management of liver toxicity during methotrexate treatment in rheumatoid and psoriatic arthritis: a systematic review of the literature. Clin Exp Rheumatol. 2009;27(6):1017-1025.

2. Singh JA, Saag KG, Bridges SL, et al. 2015 American college of rheumatology guideline for the treatment of rheumatoid arthritis. Arthritis Rheumatol. 2016;68(1):1226-26.

3. Lahdenne P, Rapola J, Ylijoki H, et al. Hepatotoxicity in patients with juvenile idiopathic arthritis receiving longterm methotrexate therapy. J Rheumatol 2002;29:2442-5.

4. Ortiz-Alvarez O, Morishita K, Avery G, et al. Guidelines for test monitoring of methotrexate toxicity in juvenile idiopathic arthritis. J Rheumatol. 2004;31(12):2501–6.

5. Kocharla L, Taylor J, Weiler T et al. Monitoring methotrexate toxicity in juvenile idiopathic arthritis. J Rheumatol. 2009;36(12):2813–8.

**Disclosure of Interest**

None declared

### P188 Therapy-dependant growth parameters in JIA patients

#### T. Marushko^1^, Y. Marushko^2^, O. Onufreiv^1^, Y.-E. Kulchytska^1^, Y. Holubovska^1^

##### ^1^Pediatrics-2, Shupyk National Healthcare University of Ukraine; ^2^Pediatrics postgraduate education, Bogomolets National Medical University, Kyiv, Ukraine

###### **Correspondence:** T. Marushko

**Introduction:** Growth and the skeletal system status are the factors that determine the prognosis of consequences in JIA course. Also, a series of recent studies has indicated that vitamin D deficiency and the presence of antibodies to adalimumab (ADA) can worsen the JIA patient condition.

**Objectives:** To evaluate the growth parameters, the skeletal system status, the vitamin D serum level, the anti-adalimumab antibodies (AAA) levels in JIA patients.

**Methods:** We assessed the data from 47 patients aged 3 to 17 years. All children were divided into 2 groups depending on the therapy type. Group I included patients treated exclusively with methotrexate (MTX) for 1 year or more (26), group II consisted of patients treated with MTX and ADA for at least 6 months (21). The frequency of growth disorders, muscle strength changes, serum calcidiol level, dual-energy X-ray absorptiometry (DXA) data, and AAA serum level in JIA patients were analyzed.

**Results:** 8.5% of our JIA patients demonstrated less than -1 SD score compared to 39% in previous years. According to manual dynamometry data, there was a decrease in muscle strength compared with healthy peers, that was especially prominent in patients with polyarticular (49.0%) and systemic (54.5 %) forms. Depending on group affiliation, it was 66.7 ± 9.4% in group I and 32.0 ± 9.5% in group II. Assessed vitamin D status revealed that 68% of patients with JIA required vitamin D level correction: 52% have mild vitamin D deficiency (serum 25(OH)D3 level is below 30 ng/ml), the remaining 16% have severe vitamin D deficiency (serum 25(OH)D3 level below 20 ng/ml). DXA data showed the signs of secondary osteopenic syndrome in 38% of patients from both study groups and significantly higher of bone mineral density (BMD) scores in children treated with MTX and ADA simultaneously (χ2 = 20.28; p <0.001). Also, in patients of group II (25.1 ± 0.9 ng/ml), the serum osteocalcin level was significantly higher (t = 4.22, p <0.001) compared with group I patients (19.4 ± 1.0 ng/ml) who took MTX exclusively. There was a positive, almost strong correlation (r-Spearman's = 0.69) between the ​​osteocalcin and BMD values. According to our multiple regression analysis results, it is the total disease activity indicator that reliably has an inverse correlation with the growth indicator expressed in SD (β = -0.45, p = 0.003) and the indicator of the relative growth rate per year (β = -0.62, p = 0.000009). It was revealed that patients with a pronounced decrease in BMD were more likely to have increased AAA level, with the highest detected level 1153.6 AU/ml.

**Conclusion:** Simultaneous treatment MTX and ADA in JIA patients is associated with a positive effect on both growth parameters and skeletal system. The decrease in bone mineral density is strongly associated with elevated titers of anti-adalimumab antibodies.

**Disclosure of Interest**

None declared

### P189 Is there a correlation between serum calprotectin and proinflammatory cytokine levels?

#### T. Marushko, Y. Holubovska, Y.-E. Kulchytska

##### Pediatrics-2, Shupyk National Healthcare University of Ukraine, Kyiv, Ukraine

###### **Correspondence:** T. Marushko

**Introduction:** One of the important tasks of modern rheumatology is the search for effective laboratory markers for making therapeutic decisions in the management of juvenile idiopathic arthritis (JIA).

**Objectives:** to conduct a correlation analysis between the levels of serum calprotectin and pro-inflammatory cytokines – IL-6 and TNF-α.

**Methods:** We evaluated serum calprotectin levels (sCal) in 28 patients (including 18 girls and 10 boys). The mean age was 11.1 ± 0.8 years, and the duration of the disease was 5.8 ± 0.7 years. IL-6 level was determined in 13 patients with systemic form of JIA (5 of them took MTX exclusively, 7 – tocilizumab, 1 - MTX and adalimumab) and in 15 patients with polyarticular form – TNF-α level in blood serum (10 patients who took MTX, 3 – adalimumab, 2 – adalimumab with MTX).

**Results:** The median serum IL-6 concentration was 23 [5; 125] pg/ml, respectively, are equal to the median sCal level of these patients – 3200 [1100; 25900] mg/l. Only in 2 patients from group II, who were in the stage of drug remission, IL-6 concentrations were within the reference values ​​(below 7.1 pg/ml). With regard to serum TNF-α levels, the median of its concentration was 8 [3, 34] pg/ml, and the median sCal level was 5500 [900; 24900] mg/l, respectively. In 8 children (4 from group I and 4 from group II), TNF-α levels were within the reference values ​​(below 8.1 pg/ml), although all these patients had the JIA active course (1^st^ or 2^nd^ stages of activity).

When conducting Spearman’s rank correlation, a significant direct strong correlation of moderate strength was found between the levels of serum calprotectin and IL-6 (r-Spearman's = 0.88, p = 0.00006), and there was found no significant correlation between the values ​​of sCal and TNF-α (r-Spearman's = 0.41, p = 0.13).

**Conclusion:** IL-6 concentration is an informative laboratory marker for assessing the inflammatory activity process in JIA patients.

**Patient Consent Received**

Yes

**Disclosure of Interest**

None declared

### P190 Multidisciplinary approach to JIA diagnosis in pediatric patients

#### T. Marushko^1^, L. Yakovenko^2^, N. Kiselova^2^, Y. Holubovska^1^, Y.-E. Kulchytska^1^

##### ^1^Pediatrics-2, Shupyk National Healthcare University of Ukraine; ^2^Department of Surgical Dentistry and Maxillofacial Surgery of Pediatric Age, Bogomolets National Medical University, Kyiv, Ukraine

###### **Correspondence:** T. Marushko

**Introduction:** Juvenile idiopathic arthritis (JIA) is the most common childhood rheumatological disease (1: 1000 children). In Ukraine, the prevalence of JIA among children 0-17 years old over the past 5 years was 0.32-0.42 cases per 1000 children. The involvement of the temporomandibular joints (TMJ), depending on the diagnostic methods, the studied population and the JIA subgroup, varies from 17% to 87%. Unfortunately, detection of TMJ arthritis in children with JIA is difficult due to the absence of early signs and symptoms. Therefore, the diagnosis of TMJ involvement often occurs late, when the growth disorders of the mandible are already evident.

**Objectives:** To determine the diagnostic measures according to a multidisciplinary approach in the early manifestations of JIA.

**Methods:** The analysis of clinical, laboratory data and TMJ MRI studies of 11 children with JIA (10 girls/1 boy) aged 8-18 years was carried out. All children are on medication.

**Results:** JIA manifested as polyarthritis (with positive rheumatoid factor (RF)) in 5 our patients, polyarthritis (with negative RF) – in 4 children, enthesitis – in 1 child, and undifferentiated arthritis in 1 child. According to radiological criteria, changes in joints were detected: stage I in 54.5%, stage II – 27.3%, stage III – 9.1%, and in 1 (9.1%) child with undifferentiated arthritis no radiological changes were found.

The manifestations of the TMJ arthritis were mouth opening limitation, pain in the TMJ and limitation of lower jaw movement in 91% of patients, which appeared already in the first year of illness in 36% of children and in 45% in the third year. Long-term absence of complaints of TMJ pain was identified in all children with positive RF. Changes in the TMJ tissues were found to correspond to complaints presented by children in 82% of cases. Adhesion of articular discs to the posterior slope of the articular tubercle was diagnosed in 27.3% (n = 3) children. Bilaminar zone with edema and intra-articular effusion - in 9.1% (n = 1) of the child. The MRI stage of changes in the TMJ coincided with the general stage in 36% (n = 4) cases and in 27.3% (n = 3) children it was higher than in other joints. No chronic changes in the TMJ structures were found in a child with undifferentiated arthritis.

The revealed changes in the muscular system of the TMJ in JIA showed that on the side of the affected joint m.pterygosdeus lateralis tended to shorten its length. M.pterygosdeus medialis lengthens from the side of the affected joint compared to the opposite side and the norm, according to 3.73 ± 0.11 cm; 3.64 ± 0.12cm and 2.91 ± 0.27cm. Such a compensatory-adaptive reaction of the masticatory muscles provides protection for the intra-articular disc, ligaments and capsules. The data obtained on changes in the structures of the temporomandibular joint and the masticatory muscles around it indicate that in the first year of the JIA disease course they are included in the symptom complex and are a component of the disease diagnostic measures.

**Conclusion:** Examination of the temporomandibular joint with the determination of anatomical and functional changes according to MRI indicators is a mandatory diagnostic test for correcting the treatment of chronic temporomandibular arthritis. Children with JIA need a multidisciplinary approach by rheumatologists and maxillofacial surgeons.

**Patient Consent Received**

Yes

**Disclosure of Interest**

None declared

### P191 Population pharmacokinetics of infliximab in children with juvenile idiopathic arthritis

#### A. Nassar-Sheikh Rashid^1^, D. Schonenberg-Meinema^1^, S. E. Berends^2^, J. M. van den Berg^1^, R. A. Mathot^2^

##### ^1^Department of Pediatric Immunology, Rheumatology and Infectious Diseases, Emma Children’s Hospital; ^2^Hospital Pharmacy, Amsterdam UMC, Amsterdam, Netherlands

###### **Correspondence:** A. Nassar-Sheikh Rashid

**Introduction:** Higher dosage regimes for Infliximab (IFX) have been described to be effective in partial- or non-responding adults and children with rheumatic disease and appear to be safe. To optimize IFX treatment in juvenile idiopathic arthritis (JIA) patients, therapeutic drug monitoring (TDM) might be beneficial. To support routine TDM of IFX and regimen optimization in JIA patients, more in-depth knowledge of the pharmacokinetic (PK) variability of IFX is needed. Ultimately, as soon as the optimal therapeutic drug ranges will be known, PK model-based simulation can be used to individualize drug dosing recommendations. In this study, a population PK model for IFX is described for JIA patients.

**Objectives:** Our hypothesis is that optimizing dosage and frequency of IFX administration for individual patients will improve treatment outcome. Individual dosages may be optimized by taking specific patient characteristics into account that explain inter-patient variability in pharmacokinetics (PK). Inter-patient variability can be quantified and investigated by the population approach. Ultimately, as soon as the optimal therapeutic drug ranges will be known, PK model-based simulation can be used to individualize drug dosing recommendations for these patients, based on their characteristics. In this current study, the population PK for IFX are described for JIA patients (which to our knowledge have not been described before).

**Methods:** Data including IFX trough concentrations and anti-IFX antibodies of 27 JIA patients on IFX maintenance treatment were retrieved from electronic charts. Three population pharmacokinetic models from literature were validated for our dataset using nonlinear-mixed effects modeling program NONMEM. A novel population pharmacokinetic model was developed based on our study data.

**Results:** A total of 65 obtained blood samples after a median of 32 days after the last IFX infusion (IQR 28-42) were analyzed. The three published models underpredicted the observed trough concentrations. A newly developed one compartment model best described the IFX serum concentration over time data in JIA patients.

**Conclusion:** Our study shows a novel and the first PK model for IFX in JIA patients. Our main finding was that a one- compartment model best described the IFX serum concentration over time data. Predictive performance of the literature models was insufficient for our patient data. Our data also show that different PK models are needed for different age categories (children or adults) and in different diseases.

**Patient Consent Received**

No

**Disclosure of Interest**

None declared

### P192 Successful experience of tofacitinib treatment in 9 patients with severe course of fibrodysplasia ossificans progressiva

#### I. Nikishina, S. Arsenyeva, V. Matkava, T. Pachkoria

##### Paediatric, V.A. Nasonova Scientific Research Institute of Rheumatology, Moscow, Russian Federation

###### **Correspondence:** I. Nikishina

**Introduction:** Fibrodysplasia ossificans progressiva (FOP) is an extremely rare genetic condition which causes by a ACVR1 gene mutation responsible for the activity of BMP and uncontrolled osteogenesis. According to its mechanism, this disease has a classic autoinflammatory pattern and after the manifestation of the first flare, usually provoked by mechanical trauma, a cascade of inflammatory reactions is triggered, ending with the development of large heterotopic ossifications

**Objectives:** To analyze the use of Janus-kinase inhibitor Tofacitinib (TOFA) as a new therapy approaches in most severe cases of FOP

**Methods:** 35 patients (pts) (17 males/18 females) with verified FOP for the period from 1998 to 2020 were analyzed. In 9 pts with severe course of FOP TOFA administration were evaluated

**Results:** We observed 35 pts with definite diagnosis FOP. Among them 9 pts (5 males/4 females), from 2 to 16 y.o. with extremely severe course of FOP and steroid addiction received TOFA. All pts have «classic» FOP stigmas and confirmed mutation in the ACVR1 gene (8 pts have classic mutation c.617 G> A (p.Arg206His), 1 pt has с.983 G>A (p.Gly 328 Glu). Multiple heterotopic ossifications were detected in all pts (neck, back, upper and lower limbs with significant limiting of motion in large joints). All pts had synovitis mostly of the low limb (hip -8, knee-7, ankle – 4) with ultrasound and MRI evidences. Active bilateral sacroiliitis with bone edema was detected in 2 of 3 patients who were able to perform MRI. In another patient sacroiliitis verified by CT. The most pts at the age after 5 years had ankylosis of the facet joints and vertebral bodies by the type of syndesmophytes (6 pts). Presence of axial skeletal damage with sacroiliitis and gradual development of ankylosis of facet joints of cervical spine, synovitis of large joints makes it possible to justify the diagnosis of juvenile ankylosing spondylitis (5 pts) or JIA (4 pts) as concomitant condition. It was done for the "organizational" reasons to facilitate the availability of the drug therapy. All patients received NSAIDs and steroids (9-per os, 5- intravenous pulse therapy), 6 pts -bisphosphonates (infusions of pamidronic acid 2-3 courses of 30-60 mg depending on weight). Due to the severe uncontrolled course of FOP with continuous flares, formation of new nodes and steroid addiction targeted anti-inflammatory therapy with the Janus kinase inhibitor was prescribed. We use the similar dose to randomized trial for JIA (up to 5 mg twice a day) after the approval of the local Ethic Committee. The first patient was 16 y.o. at the time of TOFA administration in December 2019, the age of the others pts was from 2 to 12 y.o. Duration of TOFA therapy is from 7 to 17 months. Compare to previous 6 months, the average number of new flares significantly decreased from 8,1 (Me 9) to 0,9 (Ме 0) in after 6 months of TOFA treatment. Only in one youngest patient of 2 y.o. the number of flares decreased from 10 to 4 per the same period. In the most of children was achieved an improvement of movements in large joints (shoulder, elbow). In one patient with severe course of FOP and localization of the inflammatory focus in the temporomandibular joint area, a rapid regression of changes with restoration of the mouth opening aperture was obtained. In all 9 pts we stopped the steroids. Drug tolerance was good in all pts, no AE were registered

**Conclusion:** The anti-inflammatory effect of TOFA allows to stop the recurrence of new flares of FOP and restrain the progression of heterotopic ossification. An additional advantage of TOFA is an oral method of use, taking into consideration contraindications for using of injections in patients with FOP.

**Disclosure of Interest**

None declared

### P193 An unusual case of systemic lupus erythematosus with Sjögren syndrome and recurring episodes of macrophage activation syndrome in a boy – difficulties to diagnose, difficulties to treat

#### M. Kaleda^1^, I. Nikishina^1^, V. Malievsky^2^

##### ^1^Pediatrics, V.A. Nasonova Research Institute of Rheumatology, Moscow; ^2^Pediatrics, Federal State Budgetary Educational Institution of Higher Education “Bashkir State Medical University” of the Ministry of Healthcare, Ufa, Russian Federation

###### **Correspondence:** I. Nikishina

**Introduction:** systemic lupus erythematosus with juvenile onset (jSLE), especially in boys, can occur with unusual manifestations, including Sjogren's syndrome (SS), accompanied by unexpected complications and difficulties in choosing therapy.

**Objectives:** to present a rare case of successfully application of abatacept in an jSLE+SS in a boy who had two episodes of macrophage activation syndrome (MAS).

**Methods:** Case report.

**Results:** 4-year-old boy admitted to our clinic for the first time in 2010 presenting the 6-month history of disease. There were fever up to 39°C, polyarthritis, anemia, trombocytopenia, hyperIgG-emia, increase TA (2N), CRP (20 mg/l), RF 30 mg/l at the disease onset. Initially JIA, polyarthicular sybtype, RF+ was diagnosed in regional hospital. Treatment with NSAIDs, GC iv 125 mg №3, methotrexati 7,5 mg weekly was started. During the next 3 weeks laboratory results were constantly getting worse (Нв 80 г/л, trombocytes 145000, leucocytes 1000, increase TA 10N) and new clinical symptoms occurred (maculopapular rash with itching; splenomegaly; febrile fever). He received GC iv + per os 1 mg/kg with a short-term effect. On 1^st^ admission in our clinic in September of 2010, he had general fatigue and tiredness, classic malar rash, severe cutaneous vasculitis (palpable purpura and digital capillaritis), Raynaud's syndrome, enanthema, myopathy, lymphadenopathy, hepatosplenomegaly, polyarthritis. Data of laboratory tests: Hb 96g/l, ESR 27 mm/h, ANA titer 1:640 h+sp+cytopl, anti-SS-A(Ro)>200 U/ml, RF 230 ME/ml, C4 0.07 g/l. The revision of bone marrow biopsy showed changes typical for MAS. So jSLE was diagnosed as with SLICC criteria, 2012 with MAS at onset according the preliminary diagnostic criteria for MAS Complicating SLE. The initial SLEDAI was 29. Patient failed to respond to GC iv repeating courses, GC per os max 0.7 mg/kg, IVIG repeating courses, DMARDs consequentially: cyclophosphamide iv, azathioprine, mycophenolate mofetil (MMF) + hydroxychloroquine. Rituximab (RTX) was introduced in November of 2013 due to inefficiency of prior therapy in dose 375mg/m2 weekly N2 on course. Concomitant therapy: GC per os 0.5 mg/kg, MMF 500 mg/day, hydroxychloroquine 200 mg/day. Treatment with RTX allowed to decrease the dose of GC to 0.2 mg/kg, to reduce the activity of the disease (SLEDAI=4), but relapses of the disease required a repeat of RTX therapy every 6 months (5 courses in total). Patient developed a recurrent episode of MAS on 6^th^ years of disease (the 8^th^ day after RTX – 5^th^ course, 1^st^ infusion). Therapy of RTX was discontinued. In June 2016 Sjogren's syndrome has been verified (according to parotid sialography, unstimulated sialometry in combination with clinical signs of dry mouth, anti-SS-A(Ro)+, RF+). Correction of therapy was carried out at the expense of the change a dose of GC, the dose of MMF was increased to 750 mg per day. Patient received repeat courses of IVIG due to recurrent infections of mouth. Since October 2017 flare due to fever, polyarthritis, skin and mucosal lesions. The dose of GC has been increased to 0.5 mg/kg. In November 2017, abatacept therapy with 10 mg/kg was started with good safety. Inactive status of the disease was achieved after 12 months of therapy. Now boy receives abatacept during 42 months, continues GC 5 mg per day, hydroxychloroquine 100 mg per day, MMF 750 mg per day. In November 2020, he underwent COVID-19 with minimal manifestations (runny nose, fatigue, positive PCR test) without reactivation of rheumatic disease.

**Conclusion:** This clinical case demonstrates efficacy and safety of abatacept in the rare combination of jSLE, SS and recurring episodes of MAS in boy with early onset.

**Patient Consent Received**

Yes

**Disclosure of Interest**

None declared

### P194 Safety of anakinra in patients with Cryopyrin Associated Periodic Syndromes (CAPS) using a graduated pre-filled syringe

#### R. Papa^1^, G. Giancane^2^, H. J. Lachmann^3^, P. Brogan^4^, E. Legger^5^, D. Lindqvist^6^, S. Cederholm^6^, F. Bagnasco^7^, N. Ruperto^2^, M. Gattorno^1^ on behalf of for the Paediatric Rheumatology International Trials Organisation (PRINTO) and the Eurofever registry

##### ^1^UOSD Centro Malattie Autoinfiammatorie e Immunodeficienze; ^2^Clinica Pediatrica e Reumatologia, IRCCS Istituto Giannina Gaslini, Genova, Italy; ^3^National Amyloidosis Centre, Royal Free Campus; ^4^Rheumatology, UCL GOS Institute of Child Health, London, United Kingdom; ^5^Pediatric Rheumatology, Beatrix Kinderkliniek, University Medical Center, Groningen, Netherlands; ^6^Sobi, Stockholm, Sweden; ^7^Servizio di Epidemiologia e Biostatistica, IRCCS Istituto Giannina Gaslini, Genova, Italy

###### **Correspondence:** R. Papa

**Introduction:** Cryopyrin associated periodic syndromes (CAPS) are a group of ultra-rare autoinflammatory diseases caused by mutations in the NLRP3 gene, leading to overproduction of IL-1β. CAPS includes the following subdiagnoses: familial cold autoinflammatory syndrome (FCAS), Muckle-Wells syndrome (MWS) and chronic infantile neurological cutaneous articular syndrome/neonatal-onset multisystem inflammatory disease (CINCA/NOMID), where FCAS is the mildest form of CAPS and CINCA/NOMID the most severe. A graduated pre-filled syringe of the IL-1 receptor antagonist anakinra was introduced to meet the need of smaller and varying doses when treating children with CAPS.

**Objectives:** To evaluate the safety of anakinra in CAPS patients using the novel pre-filled syringe.

**Methods:** Investigators managing patients with CAPS treated with anakinra were identified via the Eurofever registry. Follow-up data of at least 3 years for each patient were prospectively collected and analyzed (EUPAS6366). The primary study endpoints were the occurrence of adverse events (AEs) with focus on serious infections, malignancies, injection site reactions, allergic reactions and medication errors, including re-use of the syringe. Secondary endpoints included dose at specific time points, discontinuations, and switch to other IL-1 blocking treatment.

**Results:** 12 patients with CAPS were included in the study, 8 with MWS, 2 with FCAS and 2 with CINCA/NOMID. The majority were male (75%) and white (83.3%). At baseline, all but one patient were already using the graduated prefilled syringe with anakinra. 5 patients required a median dose of 2 to 3 mg/kg/day and 6 patients below 2 mg/kg/day. During a total of 26.1 patient years of treatment, there were 7 AEs with rate of 26.8 (95% CI 4.2-169.6) per 100 patient years. All AEs were infections from 1 patient with CINCA/NOMID and were considered unrelated to anakinra by the investigator. Two of the AEs were considered serious as hospitalization was required (1 tonsillitis and 1 urinary tract infection). No AEs were severe, 6 of the AEs were of moderate severity and 1 was of mild severity. In total, 6 patients discontinued anakinra permanently, 2 during the first year of treatment, 3 during the second year and 1 after the third year of follow-up. The reasons for discontinuation included switch to canakinumab (4 patients), switch to canakinumab and inefficacy (1 patient) and non-compliance (1 patient). The remaining 5 patients continued anakira until the end of the study. 1 patient discontinued anakinra temporarily during the second year of treatment due to non-compliance. No deaths, malignancies, injection site reactions, allergic reactions or medication errors, including re-use of the pre-filled syringe, were observed.

Results are shown as number (%) unless stated otherwise.

**Conclusion:** The results of the present study confirm the safety profile of anakinra treatment in CAPS patients using the graduated pre-filled syringe. No new safety findings were identified.

**Trial registration identifying number:** EUPAS6366

**Patient Consent Received**

Yes

**Disclosure of Interest**

None declared

**Table 1 (abstract P194).** Study results

Patients	12 (100)
Age at baseline, median (range), years	25.3 (1.4-54.9)
Disease duration, median (range), years	15.5 (1.4-51.3)
Anakinra dose at baseline, median (range), mg/kg/day	1.7 (1.1-2.5)
Total duration of anakinra exposure, median (range), years	1.3 (0.5-7)
Patients with AEs	1 (8.3)
Any AE	7 (100)
Urinary tract infection	1 (14.3)
Upper respiratory tract infection	1 (14.3)
Tonsillitis	5 (71.4)

### P195 A rare case of alopecia areata in the presence of TNF-α inhibitors in a patient with juvenile idiopathic arthritis

#### Y. Spivakovskiy^1^, A. Y. Spivakovskaya^2^, Y. Chernenkov^2^, A. Kandrina^2^

##### ^1^Department of Faculty Pediatrics; ^2^Depertment of Hospitality Pediatrics, Saratov State Medical University, Saratov, Russian Federation

###### **Correspondence:** A. Y. Spivakovskaya

**Introduction:** Juvenile idiopathic arthritis (JIA) is one of the most common and potentially disabling diseases in pediatric rheumatology. An important role in the pathogenesis of JIA is played by TNF-α and, therefore, biological preparations aimed at blocking it are extremely important. The use of biological therapy makes it possible to achieve stable remission, however, suppression of a number of biological effects of TNF-α can also lead to the development of unwanted drug reactions. The most common adverse drug manifestations of biological therapy are widely known, but in practice, rare side effects may occur.

**Objectives:** presentation of a clinical case of a rare side effect during the use of a TNF-α blocker in a patient with JIA.

**Methods:** A male patient ten years old with a diagnosis of JIA of the oligoarticular variant verified according to the ILAR criteria is under observation at the University Clinical Hospital from 2019 to the present. The duration of the disease at the time of the description of the clinical case was 3 years.

**Results:** At the onset of the disease, articular pathology prevailed in the patient; against the background of the use of NSAID and DMARDS, no pronounced positive clinical effect was obtained. There were indications for the name of biological therapy. The addition of uveitis made it possible to select a representative of the group of TNF-α inhibitors - recombinant monoclonal antibodies to TNF-α - adalimumab as a starting drug, in the standard dose. On the background of treatment within one calendar year, the patient achieved a stable positive clinical effect, according to the criteria ACR Pedi 70 and ACR Pedi 90 were obtained by the end of the third and sixth months, respectively. In the second year of biological therapy, against the background of maintaining a persistent effect within the framework of the minimal clinical and humoral activity of the disease, the patient was verified to have focal hair loss on the head. The child was comprehensively examined in accordance with clinical guidelines in cases of development of unspecified alopecia, including an immunological and endocrinological examination. Taking into account the peculiarities of the course of the underlying pathology, further decisions on the genesis of alopecia were made within the framework of a consultation of university clinics with the participation of rheumatologists, dermatologists and a clinical pharmacologist. After excluding other causes, the genesis of alopecia was verified as a side effect of the ongoing biological therapy with recombinant monoclonal antibodies to TNF-α. The biological therapy was changed to a drug with a different effect. Against this background, over the next 6 months, regression of the pathological symptom, partial restoration of the hairline was noted.

**Conclusion:** Anti-TNF-α antibodies are the first line of choice when using biological therapy in patients with JIA. When using biological therapy, in addition to a positive effect on the course of the underlying disease, the risk of developing a number of side effects, including alopecia areata, remains. The EULAR consensus presents the most general ideas about the development of side effects in the presence of biological therapy. At the moment in Russia there are isolated mentions of such complications of biological therapy, all of them are predominantly in adult patients. At the moment, there are no unified principles of therapeutic measures for the development of alopecia, as a side effect of biological therapy. The general therapeutic consensus of rheumatologists and other specialists will make it possible to develop a unified approach to treatment and diagnostic tactics in real clinical practice.

**Patient Consent Received**

Yes

**Disclosure of Interest**

None declared

### P196 Comparison of the efficacy and safety of original and biosimilar adalimumab molecules in childhood rheumatic diseases

#### K. Ulu, F. Demir, T. Coşkuner, S. Çağlayan, B. Sözeri

##### Department of Pediatric Rheumatology, University of Health Sciences, Umraniye Training and Research Hospital, Istanbul, Turkey

###### **Correspondence:** K. Ulu

**Introduction:** The TNF-α inhibitor adalimumab is a biological disease modifying anti-rheumatic drug (bDMARD) that has been used in different rheumatic diseases with a resistant course. ABP-501 is a biosimilar product (BP) of adalimumab, recently approved by the FDA and EMA. To our knowledge, there is no study assess and compare the efficacy and safety of the original and biosimilar adalimumab product on pediatric patients.

**Objectives:** We aimed to compare the efficacy and safety of the original and biosimilar adalimumab (ABP-501) molecules in childhood rheumatic diseases.

**Methods:** This non-interventional, retrospective, single-centre analysis carried out in Umraniye Training and Research Hospital, Pediatric Rheumatology Clinic, Istanbul, Turkey. The study group consisted of patients who were followed due to chronic rheumatic disease between January 1, 2016 and June 1, 2020, and received reference or biosimilar adalimumab therapy for at least three months. Demographic and clinical data of patients were collected at baseline, 3rd, 6th, and 12th months of treatment. Disease activity assessment was made with JADAS-27 in JIA patients, with SUN criteria in uveitis patients, and with Behçet's Disease Activity Index in BD patients. During the screening visit to exclude tuberculosis, all subjects were evaluated for tuberculosis using the QuantiferonTB gold test and a chest X-ray (if not done in the last 6 months). Efficacy and safety of treatments were compared between reference adalimumab (RA) and biosimilar adalimumab (BA) groups.

**Results:** A total of 98 patients (72 with original and 26 with biosimilar molecule) treated with adalimumab, were included in the study. There were 50 female and 48 male in the study, and the median age at the initiation of the adalimumab was 140 months (min-max: 36-231) for RA group and, 178 months (min-max: 96-225) for BA group. Of the 98 patients evaluated, the primary diagnoses of 68 were juvenile idiopathic arthritis, 18 were idiopathic uveitis, seven were Behçet's disease, two were Blau syndrome, two were chronic recurrent multifocal osteomyelitis and one was Vogt-Koyanagi-Harada syndrome. All patients received concurrent DMARDs in at least one interval in the treatment period with adalimumab. There were no substantial differences in terms of the types of previous DMARDs used between each RA and BA group. 72 of the patients were biologic-naïve, and 13 were switched from etanercept, 10 from infliximab, and three from other bDMARDs. The median exposure time of adalimumab was 17 months (min-max:4-70) in RA and 18 months (min-max: 5-24) in BA. All patients had active disease before treatment. In the group treated with RA, inactive disease was achieved in 69.4%, 81.7% and 76.7% of the patients at the 3rd, 6th and 12th months, respectively. Also, inactive disease was achieved in 57.7%, 62.5% and 72.7% of the patients at the 3rd, 6th and 12th months in the group treated with BA, respectively. There was no statistically significant difference in efficacy between the groups at the 3rd, 6th and 12th months (p=0.33, 0.09 and 0.77). Mean changes from baseline in JADAS-27 were similar between RA and BA at each study visit. Serious adverse events (AE) were seen in one patient in each groups (lymphoma in RA group, tuberculous meningitis in BA group). Non-serious AE were observed in eight patients (11.1%) in the RA group and in two patients (7.7%) in the BA group, without statistically significant difference between groups (p=0.3).

**Conclusion:** No significant difference was observed between the biosimilar adalimumab ABP-501 and referance adalimumab in terms of efficacy and safety. Biosimilars could increase patients' access to bDMARDs due to lower drug prices, provide savings for healthcare systems and patients.

**Trial registration identifying number:**

(approval no: B10.1TKH.4.34.H.G.P.0.01).

**Patient Consent Received**

Yes

**Disclosure of Interest**

None declared

## e-Poster viewing: Bone in rheumatic diseases

### P197 JIA patients do not have more osteoporosis than healthy age and sex-matched controls but lower bone mass density is found at total hip and cortical compartment

#### M. Lopez Corbeto^1^, L. Martínez-Mitjana^1^, E. Moreno-Ruzafa^1^, M. Barceló^2^, M. Pascual-Pastor^2^

##### ^1^Pediatric Rheumatology Department; ^2^Bone Metabolism Department, Hospital Universitari Vall d'Hebron, Barcelona, Spain

###### **Correspondence:** M. Lopez Corbeto

**Introduction:** Achievement of a normal peak bone mass is an especially important consideration in young adults with juvenile idiopathic arthritis (JIA), because interference with attainment of peak bone mass may not be repaired later in life. It has been suggested that children with JIA cortical appendicular skeletal bone is more affected by disease activity than axial trabecular bone. To evaluate bone mineral density (BMD) DXA measurements are widely used. Although, DXA is limited to two-dimensional evaluation of integral BMD and cannot differentiate between trabecular and cortical bone compartments. Alternatively, 3D-DXA analysis is a new method based on DXA scans of proximal femur that provides accurate estimates of trabecular and cortical volumetric BMD.

**Objectives:** The aim of the study was to analyze the trabecular and cortical bone using 3D-DXA in young adults with JIA compared to age-matched healthy controls.

**Methods:** This cross-sectional study was aimed to analyze the differences in 3D-DXA proximal femur compartments. The patients were recruited from the specialized transitional unit of the Vall d’Hebron University Hospital. JIA patients older than 18 yo without previous bisphosphonates intake were included. Age and sex-matched healthy controls were selected. Risk factors for OP were assessed in all participants, suptype of JIA and disease activity was evaluated. DXA scans (Lunar Prodigy, General Electric Medical Systems, v.15) were acquired to both patients and controls. OP was defined according to the WHO criteria. The 3D-DXA software was used to assess in 3D the trabecular density and cortical thickness from DXA scans as reported previously [1]. Quantitative variables were described using means and standard deviations (SD) whereas frequencies and percentages were reported for qualitative variables. The 3D-DXA and DXA measurements were compared using Student’s *t* test. Categorical variables were compared using Chi-squared test. All hypothesis tests with p value lower than 5% were considered significant.

**Results:** Forty-six patients and 46 age and sex-matched controls were evaluated. The mean time of disease’s duration was 7.37 years, and the most frequent JIA subtype was Oligoarticular ANA positive. More than 21% of the patients had an active disease. OP was present in 5 patients and 1 control. Despite not finding significant differences in the prevalence of osteoporosis, JIA patients had lower aBMD at total hip by DXA (0.932 ± 0.02 g/cm^2^ in patients and 1.001 ± 0.01 g/cm^2^ in controls, p-value=0.008) and and lower cortical sBMD by 3D-DXA than controls (147.23 ± 3.41 mg/cm^2^ in patients and 161.52 ± 2.89 mg/cm^2^ in controls, p-value= 0.002).

**Conclusion:** JIA patients do not have more OP than controls in our cohort but there are differences in BMD in the different locations and greater involvement of the cortical bone in JIA.

**Patient Consent Received**

Yes

**Disclosure of Interest**

None declared

### P198 Biomarkers in juvenile idiopathic arthritis associated with inflammation and bone health

#### A. Lundestad^1^, L. E. Cetrelli^2^, P. Frid^3^, M. Hoff^4^, G. B. Hoftun^1^, A. Bletsa^5^, K. Tylleskär^6^, E. B. Nordal^7^, A. J. Feuerherm^2^, A. Sen^2^, M. Rygg^1^

##### ^1^Department of Clinical and Molecular Medicine (IKOM), and Department of Pediatrics, NTNU - Norwegian University of Science and Technology, and St. Olavs Hospital; ^2^Center for Oral Health Services and Research, Mid-Norway (TkMidt), Trondheim; ^3^Institute of Clinical Dentistry, and Department of ENT, UiT The Artic University of Norway, and University Hospital of North Norway, Tromsø; ^4^Department of Neuromedicine and Movement Science (INB), and Department of Rheumatology, NTNU - Norwegian University of Science and Technology, and St. Olavs Hospital, Trondheim; ^5^Department of Clinical Dentistry, Oral Health Centre of Expertise in Western Norway, and University of Bergen; ^6^Department of Pediatrics, Haukeland University Hospital, Bergen; ^7^Department of Clinical Medicine, and Department of Pediatrics, UiT The Artic University of Norway, and University Hospital of North Norway, Tromsø, Norway

###### **Correspondence:** A. Lundestad

**Introduction:** Very little is known about biomarkers associated with inflammation and bone health in juvenile idiopathic arthritis (JIA). Studies on factors predicting disease activity and long-term outcomes among children with JIA are needed.

**Objectives:** To study proteins related to inflammation and bone health in children with JIA compared to controls, and to identify biomarkers that may characterize certain subgroups of JIA with high risk of adverse outcome.

**Methods:** In the NorJIA study https://norjia.com, 228 children with JIA from three different regions in Norway and age- and sex-matched controls participated in a multi-center, longitudinal cohort study. Clinical examination, patient/parent-reported questionnaires, imaging, and blood tests were performed. Inactive disease was defined according to the ACR criteria (1). Bone mineral density (BMD) was measured with dual-energy X-ray absorptiometry (DXA). We selected participants from this cohort with active or inactive disease, low or high BMD Z-score, and controls. Serum samples were analyzed with a multiplex immunoassay with 92 inflammation-related proteins, of which several are known to interact in bone metabolism (Olink Proteomics, Inflammation panel). The results are presented as mean normalized protein expression (NPX) values (arbitrary log2 scale units). A mean NPX difference of 1.0 means a doubling of protein concentration.

**Results:** Median age of the 43 children with JIA (67% girls) was 13.9 (IQR 10.6 -15.1) years, while median age of the 29 controls (72% girls) was 14.3 (IQR 12.7-16.4) years. Median disease duration was 4.7 (IQR 2.4-8.1) years, and 49% had active disease at the study visit. In the JIA group, 13 children had lumbar BMD Z-score ≤-1. In children with JIA *versus* controls, we observed a ≥0.4-fold increase in serum levels of TNF-α, S100A12 (EN-RANGE), FGF-21, CCL20, IL-6, SIRT2, 4E-BP1 and a ≥0.4-fold decrease in levels of OSM, IL-13 and TGF-alpha. A similar pattern was found in the subgroup with active disease *versus* controls with ≥0.4-fold increase in TNF-α, S100A12, FGF-21, CCL20, IL-6, SIRT2, 4E-BP1 and a ≥0.4-fold decrease in IL-13. In the subgroup with low *versus* high BMD Z-score, TNF-α and TNF-β were ≥0.4-fold increased, while OSM, S100A12 and IL-5 were ≥0.4-fold decreased.

**Conclusion:** In this explorative study on inflammatory biomarkers, we found characteristic changes in several inflammation-related proteins in JIA *versus* controls. We also found similar characteristics in JIA with active disease *versus* controls, while children with low *versus* high BMD Z-score had a slightly different profile. Several of these proteins have not previously been studied in JIA.

**References**

1. Wallace CA, Giannini EH, Huang B, Itert L, Ruperto N; Childhood Arthritis Rheumatology Research Alliance et al. American college of Rheumatology provisional criteria for defining clinical inactive disease in select categories of juvenile idiopathic arthritis. Arthritis Care Res. 2011;63(7):929-936

2. Nishida M, Saegusa J, Tanaka S, Morinobu A. S100A12 facilitates osteoclast differentiation from human monocytes. PLoS One.2018 Sep 20;13(9):e0204140

3. Ganeva M, Fuehner S, Kessel C, Klotsche J, Niewerth M, Miden K et al. Trajectories of disease courses in the inception cohort of newly diagnosed patients with JIA (ICON-JIA): the potential of serum biomarkers at baseline. Pediatr Rheumatol Online J. 2021 May 1;19(1):64

**Trial registration identifying number:** https://clinicaltrials.gov/ Identifier: NCT03904459

**Patient Consent Received**

Yes

**Disclosure of Interest**

None declared

### P199 Bone fragility risk factors in a pediatric rheumatology department - data from a single-center

#### A. T. Melo^1,2,3^, C. Tenazinha^2,3^, F. O. Ramos^1,2,3^, P. C. Reis^1,3,4^, J. E. Fonseca^1,2,3^, R. C. Marques^1,2,3^

##### ^1^Unidade de Reumatologia Pediátrica; ^2^Serviço de Reumatologia e Doenças Ósseas Metabólicas, Hospital de Santa Maria, Chuln; ^3^Unidade de Investigação em Reumatologia, Instituto de Medicina Molecular, Faculdade de Medicina, Universidade de Lisboa; ^4^Serviço de Pediatria, Hospital de Santa Maria, Chuln, Lisbon, Portugal

###### **Correspondence:** A. T. Melo

**Introduction:** Low bone mineral density (BMD) is an under-recognized complication of chronic illness in childhood. Reducing the frequency of fragility fractures requires increased attention to risk factors, early intervention, and additional research to optimize therapy and potentially prevent their occurrence.

**Objectives:** To identify and characterize children with risk factors for low BMD followed in a Paediatric Rheumatology unit.

**Methods:** A retrospective observational study was conducted by consulting all the clinical records of patients followed at a Pediatric Rheumatology Unit, from January 1st 2020 to April 30th 2021. The following criteria were considered risk factors for low BMD: active chronic inflammatory disease (defined as moderate or high disease activity for at least 3 months duration), corticotherapy (>3month duration), reduced mobility, puberty disorders, metabolic diseases and previous fractures.

**Results:** Five hundred and sixty-six patients were analyzed, 71 (12.5%) of whom had risk factors for low BMD. Forty patients (56.3%) were female, with a median age of 13.7 [2-21] years. Nine patients with osteogenesis imperfecta (OI) were identified (2 of whom had also reduced mobility). Four other patients had reduced mobility (all had Duchenne muscular dystrophy diagnosis and all medicated with corticosteroids); a malabsorption syndrome was identified in 6 patients (half of them had a metabolic genetic disease diagnosis). Active chronic inflammatory diseases were identified in 41 patients. From those, 37 were also exposed to corticosteroids. The most frequent active chronic inflammatory diseases identified were juvenile idiopathic arthritis (n=12) and juvenile systemic lupus erythematosus (n=12), followed by idiopathic uveitis (n=3), juvenile dermatomyositis (n=3) autoimmune hepatitis (n=2), mixed connective tissue disease (n=2), and ANCA vasculitis (n=2).

If possible, patients were stimulated to practice physical exercise. An adequate dietary calcium intake and vitamin D supplementation were advised. Nineteen patients (26.7%) had at least one fragility fracture identified of whom 10 patients had a Z score of -2. Four of these patients were currently treated with alendronate, 5 with zoledronic acid (4 biannually and 1 annually) and 1 patient was treated with denosumab. None of these patients had subsequent fractures, except a girl with OI type VI that had previously been treated with pamidronate, but maintained repetitive fractures and for that reason was started on denosumab.

**Conclusion:** In our cohort, inflammatory chronic diseases were the main risk factors for low BMD, demonstrating the impact of inflammation on bone, potentiated with steroid intake. An adequate treatment of the underlying illness, exercise, a sufficient calcium intake and vitamin D status are essential to prevent osteoporosis. If general measures fail to prevent further bone loss and fracture, pharmacologic therapy may be considered.

**Disclosure of Interest**

None declared

### P200 A rare cause of paediatric wrist pain unmasked by minor trauma

#### F. Patel, on behalf of Consultant supervisor -Dr Arani Sridhar, Radiology advice from Dr Ahmed Sharaf - University Hospitals of Leicester

##### Paediatric Rheumatology, University Hospitals of Leicester NHS trust, Leicester, United Kingdom

###### **Correspondence:** F. Patel

**Introduction:** A 10 year-old girl was referred to paediatric rheumatology with a six-month history of a painful, swollen left wrist associated with functional limitation and disturbed sleep. She initially had a minor fall, X-rays at the time showed no bony abnormalities.

**Objectives:** Blood tests were normal including inflammatory markers and autoimmune screen. Wrist MRI showed significant synovial thickening and avascular necrosis of the left lunate (figure 1).

**Methods:** Our patient received non-steroidal anti-inflammatories and physiotherapy, but a year later continued to have chronic regional pain with allodynia and hyperalgesia. Despite this, she remains upbeat and continues to live a normal childhood and has coped well with the recent increase in computer usage and typing associated with remote-schooling as a consequence of closures during the COVID-19 pandemic.

**Results:** Kienböck disease; an eponym for avascular necrosis of the lunate bone, is of unknown aetiology and incidence^[1]^. The proposed trigger is trauma in those with a susceptibility due to natural skeletal and vascular variations^[2]^. It is the commonest cause of adult aseptic osteonecrosis of the upper extremity, usually in dominant hands of men aged 20-40^[3]^. Paediatric Kienböck is rare; presenting as pain, stiffness, swelling and reduced power often after an innocuous fall. Diagnostically this is challenging because the mechanism suggests a soft-tissue injury whereas the chronicity mimics Juvenile Idiopathic Arthritis.

Radiographic severity is defined by Lichtman classification and used to guide non-curative surgical or conservative management^[4]^. This aims to relieve pressure on the lunate bone and restore perfusion. Anti-inflammatory medications are offered prior to surgical joint-levelling to reduce pain, swelling and deformity^[5]^.

**Conclusion:** We emphasise that clinicians consider this rare, destructive pathology in their differential diagnosis for paediatric chronic wrist pain and swelling, especially in those presenting weeks after a seemingly innocuous hand trauma.

1. Kienböck R. Über traumatische Malazie des Mondbeins und Ihre Folgezustände: Entartungsformen und Kompressions frakturen. Fortschr Geb Rontgenstr 1910. 16:77–103.

2. Irisarri C. Aetiology of Kienbock’s disease. Journal of hand surgery. 2004;29(3):281–7.

3. Azar MS, Kowsarian SAS, Mohseni-Badnpei, MS, Hadian A. Kienbock's Disease in a Child. Iran J Med Sci 2011; 36(2): 133-135.

4. Allan CH, Joshi A, Lichtman DM. Kienböck’s disease: diagnosis and treatment. J Am Acad Orthop Surg. 2001;9:128–136

5. Innes L, Strauch RJ (2010) Systematic review of the treatment of Kienbock’s disease in its early and late stages. J Hand Surg Am 35(5), 713–717, 717 e711-714.

**Patient Consent Received**

Yes

**Disclosure of Interest**

None declared

Figure 1 - Coronal T1-weighted (A) and coronal T2-weighted fat supressed (B) MRI of the left wrist. The lunate bone has low T1 signal (white arrow) and high T2 signal (blue arrow) due to bone marrow oedema and bony changes associated with avascular necrosis. The synovium exhibits the same pattern of signal change due to thickening and reactive inflammatory changes

### P201 Non-inflammatory arthropathy in children referred to pediatric rheumatology clinic for joint pains and deformities: our experience from Chandigarh, North India

#### P. Patra^1^, V. Pandiarajan^1^, A. Kaur^2^, S. Guleria^2^, M. Sudhakar^2^, A. K. Jindal^2^, D. Suri^2^, A. Rawat^2^, A. Gupta^2^, G. S. L. Bhavani^3^, A. Shukla^3^, K. M. Girisha^3^, S. Singh^1^

##### ^1^Pediatrics; ^2^Post Graduate Institute of Medical education and Research, Chandigarh; ^3^Medical Genetics, Kasturba Medical College, Manipal, India

###### **Correspondence:** P. Patra

**Introduction:** Noninflammatory arthropathies are groups of a rare autosomal recessive skeletal dysplasia of childhood, mistaken as juvenile idiopathic arthritis (JIA)

**Objectives:** To study the clinico-laboratory characteristics of children, diagnosed with noninflammatory arthropathies and was followed up in the Pediatric Rheumatology Clinic in a tertiary care institute in North-West India.

**Methods:** Case records of 23 children with noninflammatory arthropathy were reviewed in retrospect. Their demographic data, clinical features, radiological features, and genetic analysis reports were analyzed.

**Results:** Out of 23 children, 16 (76.1%) were diagnosed as progressive pseudorheumatoid dysplasia (PPRD), 4 patients had Camptodactyly-arthropathy-coxa vara-pericarditis (CACP) syndrome, 2 patients had Multicentric osteolysis, nodulosis, and arthropathy (MONA) syndrome and one had epiphyseal dysplasia.

The age of onset of symptoms in children with PPRD was 4 (IQR; 2.25-5.75) years and median delay in establishing definitive diagnosis was 5.5 years (IQR; 4.13-8.0). Small joints involvement was noted in 18 (80.95%) patients. Wrist, elbow, shoulder joints were involved in 8 (50%), 14 (87.54%), 3 (18.75%) of patients. Likewise, knee, ankle joint, hip joints were affected in 11 (68.75%), 9 (56.25%) and 4 (19.4%) children. In 3 (18.75%) children there was involvement of cervical spine. Bilateral symmetry of joint involvement was noted 12 (75%) children. Radiological changes like platyspondyly, platybasilli, osteopenia, or widening of joint space were evident in all cases. Before referral to our institute, 40% of children were misdiagnosed as cases of JIA and were managed with anti-inflammatory and immunomodulatory drugs. In 11 (68.75%) patients, mutation in WNT1- inducible- signaling pathway protein 3 (*WISP3*) gene was detected. We also noted a novel pathogenic variant in one patient.

Four patients with CACP syndrome were identified based on typical clinical findings – non-inflammatory nature of joint pain and synovial enlargement, camptodactyly, and constrictive pericarditis. All had pathogenic variation in the plasticity-related gene (PRG) gene. One patient was misdiagnosed as rheumatic fever and had been on penicillin prophylaxis for 6 months. Pericardiectomy was required in two patients to relive the features of congestive cardiac failure.

Two patients with MONA syndrome were identified based on extensive symmetric joint deformities involving small and large joints and presence of osteopenia in radiographs. Pathogenic variant in *MMP2* gene was detected in both of them.

In one patient with progressive joint stiffness and flexion contracture of hips, autosomal dominant epiphyseal dysplasia was identified based on positive family history and skeletal radiographs. Mutation in Exon 53 of *COL2A1*gene was documented in this patient.

Fifteen (65.21%) patients already had joint deformities at the time of presentation. Twenty- one patients were alive at the times of this study whereas 3 patients were lost to follow-up.

**Conclusion:** Noninflammatory arthropathies are distinct group of musculoskeletal disorders. PPRD is the most common noninflammatory arthropathy found in our cohort. As these group of musculoskeletal disorders mimic JIA, and there is a lack of awareness among clinicians, a considerable delay in diagnosis was observed. There is also a need of increase awareness among clinicians and rheumatologists about these groups of musculoskeletal disorders in order to initiate appropriate therapeutic and rehabilitative measures.

**Patient Consent Received**

No

**Disclosure of Interest**

None declared

### P202 Use of intravenous pamidronate in pediatric leukemia patients with osteonecrosis (ON) resulted in reduced pain, stabilization of on lesions, and avoidance of arthroplasty

#### P. Miettunen^1^, S. L. Stephenson^1^, S. Haider^2^, V. Moorjani^2^, R. P. Anderson^3^

##### ^1^Pediatrics; ^2^Diagnostic Imaging; ^3^Pediatric Oncology, Alberta Children's Hospital and University of Calgary, Calgary, Canada

###### **Correspondence:** P. Miettunen

**Introduction:** Osteonecrosis (ON) is a disabling complication of chemotherapy, especially corticosteroids, for acute lymphocytic leukemia (ALL), in children and adolescents. Hip and knee joint surface involvement by >50% can result in severe joint collapse which may require arthroplasty within 2 years of ON onset. There are few reports in the literature of non-surgical management of ON.

**Objectives:** Pediatric ALL patients (pALL) with chemotherapy related ON, treated with intravenous pamidronte (PAM), were analyzed for clinical and radiologic outcome.

**Methods:** This was a prospective study. All consecutive pALL patients (0-18 years), between 2004-2019 at a single institute, who developed bone pain, were evaluated for ON with a whole body MRI (WB-MRI) and for osteoporosis by bone mineral density (BMD) and fracture history, according to the International Society for Clinical Densitometry criteria. Patients with confirmed ON received two 9-month courses of once/monthly IV-PAM (1 mg/kg/dose, maximum dose 60 mg/dose). Visual analogue scale for pain (VAS), with “0” being “no-pain” and “10” being “the worst possible pain” was administered at baseline, 3, 6 and 12 months and yearly. The radiologic outcome was assessed by serial WB-MRIs and serial joint radiographs. All patients with ON were evaluated for bone turnover and BMD at set intervals.

**Results:** Out of 40 pALL patients with bone pain, all 40 met criteria for osteoporosis and 24/40 (60%) had ON (9F:15M). ON was diagnosed at mean 12.8 (median 6.1) months after ALL diagnosis. Thirteen patients (55%) were > 10 years at ON diagnosis. Twenty patients (83%) had ON lesions in both upper and lower extremities and 4 patients (17%) in lower extremities only. ON affected 26 large joints (shoulders [10], hips [4] and knees [12]). All 4 hips and 4/12 knee joints had >50% of joint surface involvement by ON.

The mean duration of follow-up was 7 (range 3-15) years. The mean pain VAS pre-PAM was 8.5/10 (range 8-10/10). The maximum pain VAS after first PAM was 1/10. The mean VAS was 0. 08/10 (range 0-1/10) at 12 months, sustained at final follow-up. All patients were off pain medications at 6 months.

No patient required arthroplasty. Two femoral heads developed minor collapse (both patients > 10 years), while other ON lesions remained stable or resolved on imaging. All patients returned to baseline physical activities.

BMD analysis showed increase in lumbosacral BMD z-score after 1 year. None of the patients with ON developed bone fractures during the followup.

**Conclusion:** PAM was well tolerated and improved joint and bony pain in all patients. Apart from minor collapse of 2 femoral heads, all patients demonstrated improved or stable ON lesions radiologically. No patient had progressive joint destruction requiring arthroplasty, including those with large juxta-articular lesions. The bone density improved in all patients. All patients returned to normal physical function. Novel treatment strategies with PAM should be considered to treat and to prevent progression of this debilitating complication in survivors of childhood cancer.

**Patient Consent Received**

No

**Disclosure of Interest**

None declared

## e-Poster viewing: Genetics and environment

### P203 First case report of RAG1 gene mutation diagnosis in 7 year-old libyan male with a previous diagnosis of vasculitis

#### A. M. A. Abushhaiwia^1^, Y. AElfawires^2^, N. Abushhiwa^3^

##### ^1^ Paediatric Rheumatology, Tripoli children's Hospital, faculty of Medicine, university of Tripoli; ^2^Paediatric Rheumatology, Tripoli Children's Hospital,faculty of Medicine,university of Tripoli; ^3^Pathology, faculty of Medicine, university of Tripoli, Tripoli, Libya

###### **Correspondence:** Y. AElfawires

**Introduction:** RAG deficiency is emerging as one of the leading causes of SCID and leaky SCID. Mutations of the recombination activating genes RAG 1 and RAG 2 are associated with a range of clinical presentations including, SCID and autoimmunity. In this report, we describe the application of whole exome sequencing for arriving at a molecular diagnosis in a child suffering from B- T- NK+ SCID and early onset autoimmunity

**Objectives:** A case report of RAG1 gene mutation diagnosis in 7 year- old male with a previous diagnosis of vascuiltis

**Methods:** Whole exome sequencing analysis revealed a homozygous mis-sense variation (c.2522G>A) p. (Arg841Gln) (chr11: 36597376;hg19) in the RAG1 gene

**Results: Case Report:**We present a 7yrs old Arab Libyan male child born to a third degree healthy consanguineous marriage, recently diagnosed with RAG1 mutation. the family had lost two boys, 1st at 6months of age due to severe pneumonia, 2^nd^ at one yr due to AI enteropathy presenting with chronic intractable diarrhea. He was followed at haematology clinic since infancy with the diagnosis of AIHA, At 17 months of age, he presented to our Rheumatology clinic as a case of vasculitis, with history of severe anaemia, history of digital ischemia, absent peripheral pulses and high BP, Rt hand weakness, levideoretularies, breathlessness, spenomegaly, laboratory tests showed HGB 2.6, WBC leukocytosis 20, thrombocytosis PLAT 812, HCT 47.8%7 elevated that showed hyperviscosity, high elevated ESR 110ml\hr, blood film for eosinophil only 1% was normal . Immune profile lab tests excluded CVID, including immunoglobulin assay all were normal. CD3 25.7 was normal CD4 was low 15.667, CD8 was normal 10.6, CD4\CD8 ratio was normal 1.46, CD19 was normal 35.41,CD56 (NK) was normal 7.86, ANA, ANCA, antidsDNAab were negative, C3 was low, C4 was low 11.4, d.dimer was elevated 894.78, Doppler ultrasound showed stenosis of right axillary and right subclavin, high resolution CTscan chest revealed micro nodular infiltration. Based on presentation, laboratory tests and images he was treated as a case of polyarteritis nodosa by steroid IV, IVIG, immunosuppressant drug (azathioprin). At age of 4 he started to lose his hearing as a consequence of, recurrent otitis media complicated by bilateral labryinthitis ossifican and bilateral chronic otomastoditis causing conductive deafens. In 2017 he required ICU admission because of thrombocytopenia and high BP, MRA scan upper and lower extremities showed right renal artery stenosis based on that takaysue arterities was considered and managed with steroid IV, IVIG, IV antibiotics and Rituximab infusion once per week for 4 doses, methotrexate SC,Bactrim syrup alternative day. In 2018 Laboratory evaluation was notable for decreased IGg 507, IgM 11 but IgA, IgD, IgE were normal, DADA2 was ruled out with normal enzyme activity assay, CT-angiogram for aorta and it’s branches was normal .Since 2013, he had about 17 admissions, around 4/ year between ICU and ward because of recurrent Pneumonia, AIHA, recurrent otitis media, left ankle arthritis, recurrent small skin nodules biopsy showed pannculitis, chronic fungal eye lids infection, recurrent mouth trush, mild clubbing fingers, hepatosplenomegally, frequent using oral and IV antibiotics, elevated inflammatory markers (ESR,CRP) leuckocytosis .The clinical diagnosis of SCID and family history of siblings death prompted us to investigate the molecular genetic correlates of the disease. Since over 13 genes are implicated in SCID, we resorted to whole exome sequencing.

**Conclusion:** Here we report for the first time RAG1 gene mutation in Libya. We also demonstrate the importance of genetic analysis for the final diagnosis in children with complex clinical sceenario

**Patient Consent Received**

Yes

**Disclosure of Interest**

None declared

### P204 Allelic polymorphism of interleukin-6 and tumor necrosis factor-α genes and features of juvenile idiopathic arthritis

#### A. Artsymovych^1^, O. Oshlianska^1^, Z. Rossokha^2^, O. Kryvova^3^, O. Okhotnikova^1^

##### ^1^pediatrics #1, Shupyk National Healthcare University of Ukraine; ^2^State Institution Reference Center for Molecular Diagnostics of the Ministry of Health of Ukraine; ^3^Medical Information Systems, International Research and Training Center for Information Technologies and Systems under NAS and MES of Ukraine, Kyiv, Ukraine

###### **Correspondence:** A. Artsymovych

**Introduction:** The function of the leading proinflammatory cytokines interleukin 6 (IL6) and tumor necrosis factor-α (TNF-α) in patients with JIA may depend on their structure, which is due to the characteristics of allelic polymorphism of their genes, which in the case of autoimmune inflammation may lead to pathomorphosis of disease.

**Objectives:** To study the allelic polymorphism of IL6 and TNF-α genes in JIA depending on the peculiarities of its course.

**Methods:** 52 patients with JIA (14 oJIA, 2 pJIA RF +, 11 pJIA RF-, 15 entJIA, 2 psJIS, 5 sJIA, 3 undifferentiated) were examined. In addition to standard examination, allelic polymorphism of TNF-α G308A & IL-6 G-174C genes using polymerase chain reaction (PCR) using specific oligonucleotide primers and subsequent analysis of restriction fragment length polymorphism, and of IL-6 and TNF in blood serum using chemiluminescence immune assay (CLIA) and the electro-chemiluminescence immune assay (ECLIA) were determined.

**Results:** The population of JIA patients with GG (wild allele) of IL-6 G-174C gene was characterized by a higher frequency of registration of hyperthermia, anemia and osteoporosis, GC (heterozygous mutation) IL-6 included all cases of pJIA RF+, CC (homozygous mutation) - predominance of ANA-positive cases, uveitis, hip joint damage, cardiometabolic changes and higher level of IL6 and immunoglobulin M in the serum. A significant statistical relationship was found between the unfavorable course of the disease and the allelic variant of the IL-6 GC gene (Pearson Chi-square = 6,165, df = 2, p = 0,045).

Significant differences in JIA activity depending on the allelic polymorphism of the TNF-α G308A gene (JADAS GA (heterozygous mutation) = 4.5 vs. GG (wild allele) = 12.71) were revealed by the t - test (p = 0.027). Patients with GG TNF-α are more likely to be ANA-positive, carriers of GA to have sacroiliitis. It should be noted that carriers of the GG allele were found three times more often than carriers of the GA allele, and there was only one carrier of AA (homozygous mutation) allele, which could affect the results.

One third of JIA patients were children with a combination of GC IL-6 and GG TNF-α (mainly oJIA and entJIA), of which 76% had an unfavorable course of disease, 73% had changes on standard 12-channel ECG, 53% secondary osteoporosis, 17.6% gastrointestinal lesions. The combination of GG IL6 + GG TNF-α had 21.15%, half - fever at the onset of the disease, 40% polyarthritis. 7 children were included in the group with GC IL6 + GA TNF-α (all with an unfavorable course, included all pJIA RF + cases, sacroiliitis, 57% received biological DMARDs).

Patients with CC IL6 + GG TNF-α were characterized by a higher incidence of uveitis, hip joint damage, ANA-positivity (70%) and HLAB27 + (56%).

**Conclusion:** Changes in the sequence of nitrogenous bases in the genes of proinflammatory cytokines can lead to changes in the sequence of amino acids in the cytokine, changes in its functional ability, which leads to changes in course of JIA. The presence of a mutant allele of the IL-6 GC gene causes a more severe course of JIA, the wild GG allele of the TNF gene - higher inflammatory activity. Cluster analysis of the data showed that the combination of two heterozygous gene mutations leads to the least favorable course of disease.

**Patient Consent Received**

Yes

**Disclosure of Interest**

None declared

### P205 Leg pain in a 4 year-old child with selective diet, think about scurvy!

#### M. F. Gicchino, S. Cioffi, E. Miraglia del Giudice, A. N. Olivieri

##### Department of Woman, Child and General and Specialistic Surgery, University of the Study of Campania Luigi Vanvitelli, Naples, Italy

###### **Correspondence:** M. F. Gicchino

**Introduction:** Scurvy is the disease generated by the lack of vitamin C, although it is considered a rare and past disease, scurvy continues to be detected in children with neurodevelopmental disorders and with restricted and selective diet habits. Clinical features are hypertrophy, swelling and bleeding of the gums, follicular hyperkeratosis, lower limbs swelling and tenderness. Identifying scurvy could be demanding due to the perceived rarity of the condition, and it could become a tricky diagnostic question given to the variety of nonspecific symptoms, including gingival manifestations.

**Objectives:** Here we present a case report of Scurvy in a 4 year-old child.

**Methods:** A 4 year-old child presented to our Department with lower limbs pain and refusal to walk for about 4 months. The symptoms were partially managed with Non Steroidal antinfiammatory Drugs. Patient’s mother did not refer ferver, skin rash or weight loss. Patient psychomotor development was referred in the norm up to 7-8 months of age, then patient presented a delay of psychomotor and language development. Dietary history reveals highly selective eating since the second year, based exclusively on ham and white meat homogenized, with refusal of fruits and vegetables. Since about 1 year the mother reports difficulty in eating, and several episodes of gengivitis with antivirals and topical antifungals with partial benefit. When patient came to our department he was in fair general conditions, his weight was 17 kg (25-50 th), his height was 105 cm.(25-50 th). Physical examination althought difficult because of the patient’s developmental delay revealed :pale and dry skin, corkscrew hair, signs of follicular hyperkeratosis in the lower and upper limbs. Child refused to walk, with legs fixed in flexion at hips and knees (“Frog leg-position”). Both legs were diffusely tender to palpation. The child was uncooperative for oral examination but erythematous, hemorrhagic, and swollen gums in maxillary anterior region were noted. Blood examinations revealed low iron and vitamin D level, inflammatory parameters (C-reactive ptotein and erythrocyte sedimentation rate) were normal. Hips, knees and ankle ultrasound did not revealed joints effusion. X-ray of the lower limbs did not revealed fractures, but generalized osteopenia and typical features of malnutrition, including a ground glass appearance, Pelkan spur, which represents a healing metaphyseal pathologic fracture, and a Wimberger ring sign, which denotes a thin sclerotic cortex surrounding a lucent epiphysis. Periosteal new bone formation secondary to subperiosteal, hemorrhage, a dense provisional calcification immediately adjacent to the physis (Frankel line), and an adjacent lucent band more, diaphyseal in location (Trummerfeld line).

**Results:** In consideration of personal history, result of blood examinations and of lower limbs X-ray, we hypothesized a state of vitamin C deficiency. This clinical suspicion was confirmed by the finding of low levels serum vitamin C <2.4 micromol / L (normal value 26.1-84.6). Supplementary treatment with oral vitamin C (300 mg daily) and D (800 UI daily) was started . Patient clinical condition improved with recovery of walking. One month after discharge, the boy had normal vitamins’ levels: 35,6 micromol/L (normal value 26.1-84.6).

**Conclusion:** Scurvy is rare, but it still occurs among children with autism and developmental disorders, so this condition should be keep in mind in a clinical constellation of lower extremity pain, limp, recurrent gengivitis, fatigue, anemia, in particular in children with an history of selective diet. The adoption of a detailed dietary anamnesis is fundamental to the early recognition of nutritional deficiency diseases in order to avoid invasive procedures and/or their severe complications.

**Patient Consent Received**

Yes

**Disclosure of Interest**

None declared

### P206 The study of association of STAT4, CRP and IRF5 gene polymorphisms and systemic lupus erythematosus with juvenile onset

#### I. Guseva^1^, M. Kaleda^2^, M. Krylov^1^, I. Nikishina^2^, E. Samarkina^1^

##### ^1^Immunology and molecular biology of rheumatic diseases; ^2^Pediatrics, V.A. Nasonova Research Institute of Rheumatology, Moscow, Russian Federation

###### **Correspondence:** I. Guseva

**Introduction:** Systemic lupus erythematosus (SLE) is a chronic autoimmune disease with strong genetic component, which determines the relevance of research on the contribution of various genetic factors to this disease. These studies are of particular importance in the SLE with juvenile onset (jSLE).

**Objectives:** To study the hypothesis of the association of some polymorphisms of the STAT4, CRP, and IRF5 genes with susceptibility to jSLE and the effect of the revealed correlation on the severity of the disease.

**Methods:** The case-control pilot study included 50 patients (pts) with jSLE (38 girls) with an average age of 12.1±2.85 years at onset and 103 healthy individuals (controls). Diagnosis of SLE was reviewed based on 2012 SLICC criteria. 80.0% of pts had acute cutaneous lupus at the onset, 46.0% - nonscarring alopecia, 76.0% - arthritis, 22.0% - oral and nasal ulcers, 22.0% - serositis, 44.0% - renal involvement, 28.0% –neuropsychiatric disorders. Leucopenia was found in 68.0% of pts, thrombocytopenia – in 28.0%. ANA were detected in 100% pts, anti-dsDNA – in 90.0%, anti-Sm – in 22.0%, antiphospholipid antibodies - in 20.0%, hypocomplementemia – in 38.0%, positive direct Coombs test – in 30.0 %. STAT4 (rs7574865, G/T), CRP (rs1205, C/T) and IRF5 (rs2004640, G/T) gene polymorphisms were genotyped using allele-specific RT-PCR assay.

**Results:** The distribution of GG, GT and TT genotypes of the STAT4 gene polymorphism rs7574865 differed statistically between jSLE and controls (36.0%, 54.0%, 10.0% and 63,1%, 33.0%, 3,9 respectively, χ ^2^=10.42, p=0.005). The minor T allele was associated with the development of jSLE (OR=2.3 [CI 1.3-4.0], p=0.002, p_C_ = 0.01). Moreover, this polymorphism was associated with the development of arthritis (OR=7.0 [CI 1.7-28.3], p=0.006, p_C_ = 0.018). There were no significant differences between jSLE pts and controls in the genotypic and allelic distributions of the CRP gene polymorphism C/T (rs1205). At the same time, a pronounced association of this polymorphism with the development of alopecia was revealed (OR=8.2 [1.9 - 34.3], p=0,004, p_C_ = 0.012). The polymorphism G/T (rs2004640) of the IRF5 gene was associated with the development of jSLE (OR=1.9 [CI 1.2-3.1], p=0.004, p_C_ = 0.012). The association of IRF5 gene polymorphism with clinical and laboratory parameters was not identified.

**Conclusion:** Our preliminary study in small groups of jSLE pts revealed that STAT4 (rs7574865) and IRF5 (rs20004640) gene polymorphisms are associated with susceptibility to jSLE. At the same time, we did not find a relationship between the CRP gene polymorphism (rs1205, C/T) and the risk of developing jSLE. The rs7574865 STAT4 gene polymorphism had a pronounced relationship with the development of arthritis in our pts with jSLE. The rs1205, C/T CRP gene polymorphism had a pronounced relationship with the development of nonscarring alopecia. Further investigations are required to clarify the role of genetic markers in jSLE as markers of the risk of developing SLE and its clinical and laboratory phenotypes in large studies in different ethnic and population groups.

**Disclosure of Interest**

None declared

### P207 A pilot study investigating the influence of apoptosis-related gene single-nucleotide polymorphisms on course and outcomes of juvenile arthritis

#### A. Kozhevnikov^1,2^, N. Pozdeeva^2^, M. Sogoyan^2^, S. Khalchitsky^2^, S. Vissarionov^2^, G. Novik^1^

##### ^1^St.Petersburg state pediatric medical university; ^2^H.Turner national medical research center for children's orthopedics and trauma surgery, Saint-Petersburg, Russian Federation

###### **Correspondence:** A. Kozhevnikov

**Introduction:** Juvenile arthritis (JA) is a chronic inflammatory disease that is characterized by long progressive course leading to development of contractures and loss of joint function. Multifactorial nature of JA is considered fact. Reduced sensitivity cell receptor to proapoptotic signals is one of the possible mechanisms maintaining synovial hyperplasia. It is generally believed that G allele of IL6 gene is associated with higher transcription activity than the C allele (A. Sen et al., 2011; F. Zehsaz et al., 2014; A. J. Ruiz-Padilla et al., 2016). Pro allele of gene P53 is associated with represses cells apoptotic effect than the Arg allele (K.Proestling et al., 2012; Ana Coelho et al., 2018).

**Objectives:** To study whether polymorphisms in apoptosis genes predict course and outcomes of juvenile arthritis.

**Methods:** We conducted for potentially functional single-nucleotide polymorphisms in Fas ligand (CD95L), TRAIL, IL6 and TP53. A total of seven functional SNPs were identified. In the first step, to study whether SNPs in apoptosis genes predict course and outcome of JA were analyzed 116 children. A second step was to assess gene polymorphisms interact with clinical predictors JA. All children were classified according to the ILAR criteria (ILAR 1997; 2001; the Edmonton revision 2004). 89 children were with ANF-positive JA (oligo-polyarthritis: ♂25, ♀66), 27 ANF-negative JA (entheso-JA: ♂18, ♀9). 60 healthy children without family history of any autoimmune disease were controls. Statistic analysis was carried out in groups of girls and boys with long active arthritis and remission.

**Results:** For juvenile arthritis genetic predictors there are no identified. Distribution of IL6 C174G, FasL-124A/G, FasL-843C/T, TRAIL-1525G/A, TP53 Arg72Pro genotypes did not differ significantly between the study groups. We identified that 67,6% of ANF-positive girls with long phase of “active disease” (n=34) were with IL6 -174 GC/GG (G allele) genotype and P53 -72 GC/CC (Pro / C allele) genotype (p<0,01; Odd's ratio 9,2; 95%CI: 2,7490 - 30,7896). All girls with IL6 -174 GG and P53 -72 CC genotype (n=9) were with severe erosive polyarthritis. 89% ANF-positive girls which achieved early “inactive disease” (n=27) were with IL6 -174 GC/GG (G allele) genotype and P53 -72 GG (Arg / G allele) genotype (p<0,01; Odd's ratio 16,9; 95%CI: 3,347- 85,54). Correlation with FasL-124A/G, FasL-843C/T, TRAIL-1525G/A polymorphisms in ANF-positive girls with outcomes of JA didn’t revealed. The difference in ANF-positive boys distribution across the genotypes was not significant. ANF-negative boys and gilrs with enteso-JA had a high frequency of P53 -72 GC/CC (Pro / C allele) genotype but this SNP was not associated with the course and outcomes of disease.

**Conclusion:** We observed that polymorphisms of IL6 C174G and TP53 Arg72Pro influence to the course and outcomes of juvenile arthritis. The strongest association was seen P53 -72 GC/CC (Pro / C allele) genotypes and ANF-positive girls. The influence of IL6 -174 G allele on course of JA evidence like doubtful fact and requires clarification. (Khalda Amr et al., 2016; Sawsan Jassim Al-Harbi et al., 2019). However ongoing studies in a large group of children should be able to reveal the effect of apoptosis-related gene single-nucleotide polymorphisms in luminal of juvenile arthritis.

**Disclosure of Interest**

None declared

### P208 Whole exome sequencing of MIS-C patients: analysis of genes involved in inborn errors of type I IFN immunity, hemophagocytic lymphohistiocytosis (HLH), kawasaki disease (KD) and TLR7 gene

#### E. Lovšin^1,2^, B. Jenko Bizjan^3^, M. Zajc Avramovič^1^, J. Kovač^3^, M. Debeljak^2,3^, T. Avčin^1,2^

##### ^1^Department of Allergology, Rheumatology and Clinical Immunology, University Medical Center Ljubljana Division of Pediatrics; ^2^Faculty of Medicine, University of Ljubljana; ^3^Clinical institute for Special Laboratory Diagnostics, University Medical Center Ljubljana Division of Pediatrics, Ljubljana, Slovenia

###### **Correspondence:** E. Lovšin

**Introduction:** After spring 2020, a series of reports from Europe and USA described clusters of children, presenting life-threatening multisystem inflammatory syndrome in children (MIS-C), associated with antecedent exposure to SARS-CoV-2 (1). In patients with life threatening COVID-19 3.5% were found to have inborn errors in type I IFN signalling pathway (2). A case series of 4 young patients with severe COVID-19 reported rare loss-of-function variants in the *TLR7* gene associated with impaired type I IFN responses (3). Clinically, MIS-C shares features with secondary hemophagocytic lymphohistiocytosis (HLH) and Kawasaki disease (KD), which were also associated with possible infectious trigger and might share a common genetic cause (4).

**Objectives:** We analysed whether MIS-C patients have an underlying presence of genetic variants in exomes associated with inborn errors of type I IFN immunity, HLH, KD and presence of variants in *TLR7* gene.

**Methods:** Blood was drawn from 17 MIS-C patients upon submission into the hospital, DNA from peripheral blood was isolated and whole exome sequencing was performed. Variants in the following genes were investigated: type I IFN immunity (*TLR3*, *UNC93B1*, *TRAF3*, *TBK1*, *IRF3/9*, *IRF7*, *IFNAR1/2*, *STAT1*/*2*, *IKBKG*, *TRIF*), HLH (*AP3B1*, *CD27*, *FADD*, *FAS*, *FASLG*, *HPLH1*, *ITK*, *LYST*, *MAGT1*, *MYO5A*, *NLRC4*, *PRF1*, *RAB27A*, *RECQL4*, *SH2D1A*, *STX11*, *STXBP2*, *UNC13D*, *XIAP*, *TNFRSF9*, *CDC42*), KD (*ITPKC*, *CD40*, *FCGR2A*, *BLK*, *CASP3*, *TRX-CAT1-7*, *PGBD1*, *LTA*, *TSBP1*, *HLA-DQB1/2*, *HLA-DOB*, *IGHV1-69*) and *TLR7* genes. Analysis was focused on rare (GnomAD<0.01) exonic or splicing variants.

**Results:** No common genetic denominators were found in analysed genes. Five rare variants were observed in four patients (4/17). According to ACMG classification variants of uncertain significance (VUS) were found in *LYST* (2), *IKBKG* (1), *IRF3* (1) and *NLRC4* (1) in heterozygous genotype. No clinical evidence was found in ClinVar database for any of the variants, except for one variant in *LYST* (c.3931A>G:p.M1311V) with uncertain significance for Chédiak-Higashi syndrome and medium prediction scores. Variants in *LYST* (c.5990C>G:p.A1997G), *NLRC4* (c.772T>C:p.C258R) and *IRF3* (c.325G>C: p.G109R) have high CADD, Mutation Taster, Polyphen and SIFT prediction scores. And *IKBKG* (c.325C>G:p.L109V) variant had medium prediction scores.

**Conclusion:** Our findings suggest that MIS-C patients do not share a rare loss-of-function variant in type I IFN immunity genes, *TLR7* gene or genes associated either with HLH or KD. Despite numerous clinical, immunological and genetic research of the MIS-C patients, the syndromes pathogenesis and etiologic cause remain elusive.

1. Syrimi E, Fennell E, Richter A, Vrljicak P, Stark R, Ott S, et al. Single-cell RNA-seq reveals profound monocyte changes in Paediatric Inflammatory Multisystem Syndrome Temporally associated with SARS-CoV-2 infection (PIMS-TS). medRxiv. 2020 Jan 1;2020.08.06.20164848.

2. Zhang Q, Liu Z, Moncada-Velez M, Chen J, Ogishi M, Bigio B, et al. Inborn errors of type I IFN immunity in patients with life-threatening COVID-19. Science (80- ). 2020;370(6515).

3. Van Der Made CI, Simons A, Schuurs-Hoeijmakers J, Van Den Heuvel G, Mantere T, Kersten S, et al. Presence of Genetic Variants among Young Men with Severe COVID-19. JAMA - J Am Med Assoc. 2020;324(7):663–73.

4. Sancho-shimizu V, Brodin P, Cobat A, Biggs CM, Toubiana J, Lucas CL, et al. SARS-CoV-2-related MIS-C: A key to the viral and genetic causes of Kawasaki disease? J Exp Med. 2021 Jun;218(6):1–16.

**Patient Consent Received**

Yes

**Disclosure of Interest**

None declared

### P209 Structural and functional condition of the bone tissue in children during the growth spurt depending on the VDR gene polymorphism

#### N. Osman, T. Frolova, N. Stenkova, E. Atamanova

##### Kharkiv National Medical University, Kharkiv, Ukraine

###### **Correspondence:** N. Osman

**Introduction:** The period of intensive growth in children is associated with active changes in the bone tissue architecture. A high level of bone mass accumulationis observed. Whether such processes are adequate depends on numerous factors, however, all of them are based on a genetic component. Gene expression affects all processes in the body, including bone tissue. The Fokl polymorphism of the VDR gene responsible for the activity of cell receptors for vitamin D3 is explored in association with bone pathology, autoimmune diseases, diseases of the central nervous, cardiovascular and other systems.

**Objectives:** The research is aimed at establishing the structural and functional features of bone tissue in children during the growth spurt, taking into account the Fokl polymorphism of the VDR gene.

**Methods:** The examination covered 205 children aged from 9 to 17, they were divided into three groups depending on the presence or absence of growth spurt (GS) and its intensity: group 1 included 50 children whose height increased by 8-12 cm for the current year; group 2 consisted of 46 children who grew by 12 cm for the present year, group 3 included 109 children without growth spurt. The eligibility criteria were as follows: no chronic somatic or endocrine pathology. The research required the collection of clinical data, medical history and an objective assessment of the level of physical (according to WHO guidelines "Child Growth Standards", 2007), molecular diagnosis of the Fokl polymorphism of the VDR gene (PCR method, Realtime), ultrasound (Sonost-2000, Korea) and X-ray densitometry (DXA) (HOLOGIC QDR W Explorer, USA). The criterion for diagnosing a decrease in the bone mineral density was considered to be BMDZ-score ≤-2 in accordance with the recommendations of The International Society For Clinical Densitometry (ISCD), 2019.

**Results:** A decrease in the bone mineral density (BMD) as observed through ultrasound densitometry (UD) was diagnosed in 24 children (48.0%) of group 1, in 28 children (60.87%) of group 2, and in 43 children (39.45%) of group 3. DXA was used to examine 32 children with a decrease in BMD. In 18 of them (56.25%) a decrease in BMD was diagnosed as shown by ultrasound. The number of children with a decreased BMD in group 1 reached 38.9% while in group 2 it was 50.0% (p<0.05). Spearman's correlation analysis showed that children in groups 1 and 2 revealed a positive relationship between the intensity of the growth spurt and BMD (r=0.40). The frequency of BMD reduction in children of group 2 was significantly higher (p<0.05) than in group 1. The following variants of the Fokl polymorphism of the VDR gene were detected in all of the examined children: a normal genotype was found in 27.81% of children, 61.95% of children showed a heterozygous mutation, a homozygous mutation was detected in 10.24% of children. A heterozygous mutation of the Fokl polymorphism in the VDR gene proved to bethe most widespread (72.48%) in the group of children without GS with a decreased BMD. Children of groups 1 and 2 showed no significant differences in the prevalence of the heterozygous mutation of the Fokl polymorphism in the VDR gene.

**Conclusion:** The main reason for a decreased BMD in school children during the growth spurt is a delayed bone mass accumulation associated with an intensive linear skeletal growth. Fokl polymorphism mutations of the VDR gene are also relevant, but their negative impact is more pronounced in children without GS. Thus, this process is not of a pathological nature and does not require any treatment.

**Patient Consent Received**

Yes

**Disclosure of Interest**

None declared

### P210 Autoimmunity across 3-generations in a family with CTLA4 haplo-insufficiency!

#### R. Shanbhag Mohite, S. Bhattad

##### Pediatric Immunology and Rheumatology, Aster CMI hospital, Bangalore, India

###### **Correspondence:** R. Shanbhag Mohite

**Introduction:** Cytotoxic T lymphocyte antigen-4 (CTLA-4) is a critical and a very potent inhibitor of T-cell proliferation that serves as a “immune checkpoint”. Patients with CTLA4 haploinsufficiency with autoimmune infiltration (CHAI) present with autoimmune cytopenias, hypogammaglobulinemia, organ-specific autoimmunity, lymphadenopathy/splenomegaly and lymphocytic infiltration of non-lymphoid organs.

**Objectives:** To describe a family with CHAI involving multiple members across 3-generations.

**Methods:** Retrospective review of clinical records was performed. A detailed clinical history including the age of presentation, symptoms, family history, findings on physical examination, laboratory findings and treatment details were recorded. Whole exome sequencing was performed on Next generation sequencing platform.

**Results:** A 10-year-old boy, born to a non-consanguineously married Indian couple presented with history of passing blood in stools from the age of 4-years. He also had had recurrent episodes of wheeze associated lower respiratory tract infections and had received multiple courses of antimicrobials. At the age of 9, he developed chronic diarrhea and was treated with antibiotics on multiple occasions with no relief. He had a strong family history involving his father and paternal grandmother.

Father, a 35-year old, developed severe anemia at the age of 13 years and was diagnosed with autoimmune hemolytic anemia (AIHA) that responded to steroid therapy. He has had three relapses so far and the recent episode was treated with intravenous immunoglobulin. At age of 22 years, he developed chronic diarrhea and was treated as a probable case of celiac disease with gluten free diet with no relief.

Paternal grandmother, 55-year old, developed AIHA at the age of 42 years and has had multiple relapses requiring steroid therapy. At the age of 45, she developed chronic diarrhea with significant weight loss, possibly autoimmune enteropathy that has partially responded to steroids.

On examination, index case was found to be underweight and had an enlarged right axillary lymph node. Computed tomography abdomen showed multiple enlarged intra-abdominal lymph nodes and endoscopy of the small and large bowel revealed diffuse nodular hyperplasia. Immunological evaluation showed normal serum immunoglobulins. In view of the strong family history,an autosomal dominant pattern of inheritance was suspected,and genetic evaluation was carried out. A pathogenic heterozygous mutation c.416A>G (p. Tyr139Cys) was identified in exon 2 of the *CTLA4* gene by whole exome sequencing. A diagnosis of CTLA-4 haploinsufficiency with autoimmune infiltration (CHAI) was established. The index case has been started on steroids and sirolimus and is under close follow-up.

A review of literature of 222 CHAI patients reported the median age of onset of symptoms to be 10-years (6-16 years) with median age at diagnosis being 23-years (17–40). Family history of immune deficiency was reported in 62% and autoimmunity was observed to be the most common presentation in these patients. The age of onset of symptoms in the index case was 4 years and age at diagnosis was 9 years, however, it was much delayed in both his father and paternal grandmother. Non-malignant lymphoproliferation as reported in previous cases series was also noted in the index patient. Initially patients with CHAI were treated with rapamycin, however various studies have reported a promising role of abatacept, belatacept and sirolimus as a first-line therapy to control manifestations of immune dysregulation. However, HSCT offers a more definitive therapy in patients with severe disease manifestations or poor control with immuno-modulatory drugs.

**Conclusion:** Poly-autoimmunity with lymphoproliferation is a known presentation of CHAI. We hereby present a family with CTLA-4 haplo-insufficiency involving members from 3-generations.

**Patient Consent Received**

Yes

**Disclosure of Interest**

None declared

## e-Poster viewing: Immunoregulation and basic science

### P211 Patient stratification through molecular immune phenotyping in psoriatic JIA

#### A. Carvalho^1^, A. Charras^1^, E. Carlson^1^, P. J. Ferguson^2^, C. M. Hedrich^1,3^

##### ^1^Department of Women's and Children's Health, Institute of Life Course and Medical Sciences, University of Liverpool, Liverpool, United Kingdom; ^2^Rheumatology, Allergy and Immunology Department, University of Iowa Stead Family Children’s Hospital, Iowa City, Iowa, United States; ^3^Department of Paediatric Rheumatology, Alder Hey Children's NHS Foundation Trust, Liverpool, United Kingdom

###### **Correspondence:** A. Carvalho

**Introduction:** Childhood skin psoriasis and psoriatic juvenile idiopathic arthritis (psJIA) are poorly understood. Approximately 50% of psJIA patients develop joint disease prior to skin involvement, making the diagnosis challenging. Because psJIA poorly responds to first-line treatments for other forms of JIA, delayed diagnosis frequently contributes to prolonged disease activity.

**Objectives:** Aiming at the definition of biomarkers and new treatment targets, this study investigated molecular phenotypes of immune cells in psoriasis/psJIA as compared to controls (enthesitis-related arthritis (ERA-)JIA, healthy individuals) using DNA methylation mapping and cytokine expression profiling.

**Methods:** Using biospecimen from patients with psJIA (5), ERA-JIA (6), and healthy controls (5) DNA and RNA were isolated from CD4+ T cells. DNA Methylation was assessed using Illumina EPIC BeadChips; gene expression was investigated using NanoString Inflammation panels.

**Results:**

Disease-specific gene expression profiles were found in CD4+ T cells from psJIA and ERA JIA when compared to healthy controls.

**Table:** 

Gene expression profiles						
ERA JIA Vs Healthy controls			psJIA Vs Healthy controls	ERA JIA Vs psJIA		
*Over-expressed*		*Down-regulated*	*Down-regulated*	*Over-expressed*		*Down-regulated*
SOCS3	BCL3	TNFSF10	KLRK1	SYK	PRKCD	PDCD1
BCL6	PTK2	XCR1	S100A8	EGR1	HLA-DRB3	DUSP4
TLR2			HLA-DRB3	HLA-DMA	CLEC4A	
CFP			XCR1	ITGAM	TNFRSF1B	
CLEC4A			C6	LGALS3	NCF4	
				BCL6		

A number of differentially regulated genes are controlled by the transcription factor cAMP-responsive element modulator (CREM)α (*SYK*, *PDCD1* and *DUSP4*). This suggests dysregulation of CREMα signalling in CD4+ T cells from psJIA patients, which has been recently reported for (adult) psoriasis and psoriatic arthritis patients.

DNA methylation profiles cluster with patient groups. Integration of gene expression profiles with methylation status of CpG positions within differentially expressed genes delivered two previously unknown targets. Expression of Killer Cell Lectin Like Receptor K1 (KLRK1) was reduced in the presence of *KLRK1* cg24995855 hypermethylation in psJIA patients when compared with healthy controls. KLRK1 belongs to the NKG2 family of C-type lectin-like receptors and are overexpressed in infected, transformed, senescent and stressed cells. Thus, reduced gene expression may result in prolonged survival of autoreactive T cells. As compared with ERA-JIA, sampled from psJIA patients exhibited increased expression of Protein Kinase C Delta (PRKCD) in the presence of *PRKCD* cg01828740 and cg08176244 hypomethylation. PRKCD is involved in lymphocyte signalling and in the regulation of growth, apoptosis, and differentiation. Increased expression may affect immune cell activation and/or increase tumour risk.

**Conclusion:** DNA Methylation patterns differentiate psJIA from ERA-JIA and healthy controls. T cells form patients with psJIA exhibit gene expression patterns associated with effector lymphocytes and CREMα signalling. Integration of DNA methylation and gene expression profiles revealed additional, not previously explored, psJIA specific targets genes *KLRK1* and *PRKCD*. Molecular immune cell phenotyping may be exploited for biomarker development and the identification of new treatment targets. Findings will be confirmed in larger cohorts using targeted approaches.

**Disclosure of Interest**

None declared

### P212

**Withdrawn**

### P213 LAG-3 is a central immune receptor in oligoarticular juvenile idiopathic arthritis

#### E. Sag^1,2,3^, S. Demir^2^, M. Aspari^3^, M. A. Nielsen^3^, C. Skejø^3^, M. Hvid^3,4^, E. Turhan^5^, Y. Bilginer^2^, S. Greisen^3^, S. Ozen^1,2^, B. Deleuran^3,6^

##### ^1^Pediatric Rheumatology Unit, Hacettepe University, Translational Medicine Laboratories; ^2^Division Of Pediatric Rheumatology, Hacettepe University Faculty Of Medicine, Department Of Pediatrics, Ankara, Turkey; ^3^Department Of Biomedicine; ^4^Department of Clinical Medicine, Aarhus University, Aarhus, Denmark; ^5^Department of Orthopedics and Traumatology, Hacettepe University Faculty of Medicine, Ankara, Turkey; ^6^Department of Rheumatology, Aarhus University Hospital, Aarhus, Denmark

###### **Correspondence:** E. Sag

**Introduction:** Oligoarticular juvenile idiopathic arthritis (o-JIA) is a common inflammatory joint disease in children, driven by continuous T cell activation. T cell activation can be counter-balanced by signals generated by inhibitory receptors (IRs) such as CTLA-4, PD-1, LAG-3, and TIM-3.

**Objectives:** We investigated the role of IRs and especially LAG3 in the pathogenesis of o-JIA.

**Methods:** O-JIA patients was enrolled at a time of a flare with at least one swollen joint from which joint fluid could be drawn. Paired samples of synovial fluid (SF) and plasma and peripheral blood mononuclear cells (PBMCs) and synovial fluid mononuclear cells (SFMCs) were collected from o-JIA patients along with their clinical data (n = 24). Plasma from healthy controls (HC) (n = 14) and paired SF and plasma samples from 5 non-arthritic juvenile orthopedic patients (OC) (n = 5) served as controls. Soluble levels of IRs (PD-1, LAG-3, TIM-3, CTLA-4) were analyzed by ELISA and their cellular expression by flow cytometry. Spontaneously differentiated fibroblast-like synoviocytes (FLSs) from SFMCs were co-cultured with autologous PBMCs/SFMCs and used as an *ex vivo* disease model. The neutralizing and agonistic antibodies against IRs were used in the *ex-vivo* disease model for the functional analysis.

**Results:** In patients with o-JIA, the median age was 11.2 (6.7–14.2) years at time of inclusion; they had a median JADAS-27 score of 7.4 (5.2–9.6) and median disease onset of 3.6 (2.4–8.6) years. Most of them were off-treatment (n = 16) at the time of sampling while 6 received methotrexate treatment for less than 3 months and 2 patients was treated with anti-TNF agents for less than 6 months. The median age of the HCs was 9.0 years (7.3–11.0) and in the OCs it was 14.0 (14.0–17.5).

The levels of the soluble IRs, PD-1, LAG-3 and TIM-3, were all significantly increased in the SF compared with plasma in o-JIA patients. Plasma sLAG-3 (p < 0.05) levels in o-JIA patients were significantly higher than the plasma levels in HCs. Soluble CTLA-4 was in all cases below the detection limit of 0.13 ng/ml in both plasma and SF.

As observed for the soluble factors, PD-1, LAG-3 and TIM-3 expressing CD3+CD4+CD45RO+ T cells were highly increased in the SF, compared with the blood. Similar to sCTLA-4, the cells expressing CTLA4 were not increased in o-JIA SF.

MHC class-II expression was induced on FLSs when these were co-cultured with autologous PBMCs and SFMCs, together with an increased MCP-1 production. The neutralizing antibodies against CTLA-4, PD-1, LAG-3, and TIM-3 were added to the cultures. Only anti-LAG-3 antibodies significantly increased MCP-1 secretion in PBMC monocultures (p < 0.01) and FLS+PBMC co-cultures (p < 0.01).

Addition of an agonistic LAG-3 antibody (IMP761) resulted in a decreased effector cytokine secretion and that of IL-10, IL-12, IL-1b, IL-4, and IL-6 reached the level of significance. (p < 0.01)

**Conclusion:** This is the first report comparing the effects of different IRs in o-JIA. Our study revealed that both the soluble levels and the surface expressions of the co-IRs are increased at the site of inflammation in o-JIA. Co-cultures of autologous FLSs and PBMCs/SFMCs may serve as an important *ex-vivo* arthritis model addressing also the stromal compartment recently discovered to be important in autoimmunity. The findings of our study support LAG-3 as a potential target in o-JIA. However, further in vivo studies are necessary to validate its role in JIA.

**Disclosure of Interest**

None declared

## e-Poster viewing: Juvenile dermatomyositis

### P214 The clinical utility of a positive antinuclear antibody test result

#### M. Kaleda, E. Fedorov, S. Salugina

##### Pediatrics, V.A. Nasonova Research Institute of Rheumatology, Moscow, Russian Federation

###### **Correspondence:** S. Salugina

**Introduction:** Symptoms and signs of a wide range of diseases in the practice of rheumatologists are caused by a systemic autoimmune response, which, in laboratory research, is primarily characterized by the expression of antinuclear antibodies (ANA). Characterization of AHA titers and patterns in a patient's serum can be of great help in diagnosis.

**Objectives:** To study the frequency, titers and patterns of ANA in all consequently patients (pts) with various rheumatic diseases in single center.

**Methods:** In this retrospective analytic study we assessed the results of the expression of ANA in all pts who were admitted to our clinic from January 2021 to March 2021 inclusive. ANA test was performed by an indirect immunofluorescence (IIF) technique with HEp-2 cells.

**Results:** Out of the 226 pts investigated for ANA. The median age at the moment of observation was 16.6 years [interquartile range (IQR) 10.5; 16.0] without difference between ANA positive/negative pts. 114 pts (50.4%) were positive, among which 82.4% were female (in group with negative ANA – 53.6% were female). 29.8% were pts ≤ 10 years of age. No ANA-associated rheumatic disease was identified in pts with an ANA ≤ 1:160. 68.4% ANA positive pts had different subtypes of JIA (39/78 RF- polyarticular, 5/78 RF+ polyarticular, 21/78 oligoarticular, 12/78 enthesitis-related arthritis, 1/78 psoriatic arthritis), 28.9% - connective tissues diseases (CTD) (19/33 – systemic lupus erythematosus, 8/33 – systemic sclerosis, 3/33 – juvenile dermatomyositis, 3/33 – Sjogren's syndrome), 2 pts – chronic recurrent multifocal osteomyelitis, 1 – TINU-syndrome. Uveitis was diagnosed in 29.5% pts with JIA. Total 35.1% pts had ANA titer 1:320, 37.7% - 1:640, 17.5% - 1:1280, 9.7% >1:1280 without difference between pts ≤ 10 years of age or older. 23.7% pts had isolated patterns (12.3% - speckled (sp), 7.9% - homogeneous (h), 2.6% - nucleolar (nucl), cytoplasmic (cytopl) – 0.9%). 76.3% pts had several mixed patterns: h+sp – 34.2%; sp+cytopl – 12.3%, h+sp+cytopl – 16.7%, sp+discrete nuclear dots (dots) – 3.5%, h+sp+dots – 2.6%, h+sp+dots+cytopl – 2.6%, h+cytopl – 1.8%, dots+cytopl – 0.9%, nucl+cytopl – 0.9%, h+centromeric (centr) – 0.9%). Pts with different subtypes of JIA were more likely to have h pattern and h+sp pattern than pts with CTD (h – 10.25% and 3.0%, respectively; h+sp – 41.0% and 18.2%, respectively). Pts with CTD were more likely to have sp pattern and sp+cytopl pattern than pts with JIA (sp – 18.2% and 7.7%, respectively; sp+cytopl – 21.2% and 9.0%, respectively). There were no statistically significant differences between JIA and CTD for other patterns. Titer 1:320 was observed more often in pts with JIA (39.7% and 21.2%, respectively), but titer ≥1:1280 was observed more often in pts with CTD (39.4% and 23.1%, respectively). Pts with JIA and ANA titer ≥1:1280 were ≤ 10 years of age or had a history of TNF-inhibitors.

**Conclusion:** Among the ANA-positive pts, there were more female. The most common pattern identified was nuclear, subpattern speckled and homogeneous (both isolated and mixed patterns). Cytoplasmic pattern was rare. The majority of pts had various mixed patterns, which confirms the trend towards the formation of a wide range of autoantibodies in RD with juvenile onset. In our study, pts with JIA were more likely to have h and h+sp patterns, whereas pts with CTD were significantly more likely to have sp and sp+cytopl patterns. A prospective study is needed to understand better the predictive value of ANA IIF in clinical setup.

**Disclosure of Interest**

None declared

### P215 Clinical profile of juvenile dermatomyositis in children with disease onset ≤3 years of age is different compared to those who had disease onset >3 years of age: a 28 years’ experience from a tertiary care centre in Northern India

#### G. Anjani, P. Vignesh, M. Sudhakar, N. Johnson, H. Chaudhary, A. Jindal, D. Suri, A. Rawat, A. Gupta, S. Singh

##### Pediatric Allergy Immunolgy Unit, Department of Pediatrics, Advanced Pediatrics Centre, Postgraduate Institute of Medical Education and Research, Chandigarh, India, Chandigarh, India

###### **Correspondence:** G. Anjani

**Introduction:** The course and outcome of juvenile dermatomyositis (JDM) in children with disease onset at or below three years of age could be different from that in the children with disease onset at greater than three years of age.

**Objectives:** To compare 2 groups of children with JDM–those with onset of symptoms on or before 3 years of age (group 1) versus those with disease onset after 3 years of age (group 2) and to assess differences in disease presentation, laboratory investigations, therapy, complications and outcomes.

**Methods:** A single-centre retrospective analysis of case records of JDM over a period of 28 years from 1993 to 2021 April was performed.

**Results:** Of the 131 children with JDM, 34 children (26%) had disease onset ≤3 years. The male to female (M: F) ratio in group 1 and group 2 were 1: 1.4 and 1.2:1 respectively. Median age at onset of symptoms were 2.4 years (range:1.6-3 years) and 7.9 years (range:6-10.5years) in group 1 and 2 respectively. Median delay in diagnosis was comparable between both groups.

Among the, clinical features, there was no statistical difference between groups 1 and 2 in terms of presence of fever (19 vs 41,p=0.230), heliotrope rash (24vs65,p=0.832);Gottron papules(26 vs 76, p=0.814),inverse Gottron(4 vs 15, p=0.779),rash(24 vs 61, p=0.532). However, the number of patients with periorbital swelling at presentation was higher in group 2 compared to group 1(49 vs 10, p=0.045). There was no statistical difference between the two groups in relation to neck flexor weakness, pharyngeal muscle weakness, and proximal muscle weakness. While none of the patients in group 1 had severe respiratory muscle weakness requiring mechanical ventilation, 5 patients(6.3%) in group 2 had severe respiratory muscle weakness. Presence of Raynaud phenomenon and interstitial lung disease have not been noted in group 1.Amyopathic forms (1/34 cases,2.9%) are lesser in group 1 as compared to group 2 (7/97,7.2%.Similarly,overlap syndromes (5/34,14.7%) are lesser in group 1 as compared to group 2 (20/97, 20.6%).

Twelve children underwent testing for myositis specific autoantibodies in ≤3 years group. Positivity for anti-TIF-gamma positivity, NXP-2 positivity, anti-Jo-1, anti-TIF-gamma with PM/Scl-100, anti-Sm with snRNP with anti SS-A were seen in 3, 2, 2, 1 and 1 respectively.

Patients in group 1 took longer time to achieve remission than patients in group 2(median time to achieve remission: 8 months vs 6 months,p-0.011).However,the number of patients receiving methotrexate,pulse steroids,pulse cyclophosphamide, intravenous immunoglobulin and mycophenolate mofetil were comparable between the two groups.

The median follow-up duration was 6.35 years and 3.4 years in group 1 and 2 respectively. The number of patients with relapses and episodes of relapses, presence of lipodystrophy and calcinosis were comparable between two groups. In group 1, there was only 1 (2.9%) death (aspiration) as compared to 7 cases (7.2%) in group2.

**Conclusion:** The highlight of the study is that none of the younger children (≤3 years) had severe respiratory muscle weakness or ventilator support requirement. Amyopathic forms of JDM, ILD, overlap syndromes, and Raynaud’s phenomenon are much less common in ≤3 years age group. Older children had higher incidence of periorbital edema at presentation as compared to younger ones. Younger patients in our cohort took significantly longer time to achieve remission but mortality rate is less in younger children as compared to older children.

**Patient Consent Received**

No

**Disclosure of Interest**

None declared

### P216 Clinical manifestations and serologic profile in mexican patients with juvenile inflammatory myopathies

#### R. C. Calderon Zamora, N. E. Rubio Pérez, A. V. Villarreal Treviño, F. García Rodríguez, K. V. Jirón Mendiola

##### Reumatología Pediátrica, Hospital Universitario "Dr. José E. González", Monterrey, NL, Mexico

###### **Correspondence:** R. C. Calderon Zamora

**Introduction:** Juvenile inflammatory myopathies (JIM) are a heterogeneous group of diseases characterized by muscle weakness, skin manifestations, and involvement to different organs^1^. They comprise four groups: dermatomyositis, polymyositis, inclusion body myositis, and immune mediated necrotizing myopathy. Overlap myositis has recently been recognized as an autonomous entity^3^. Juvenile dermatomyositis covers 80% of patients, with an incidence of 4.1 cases per million children per year^1^.

Laboratory tests are fundamental to the diagnosis of JIM, more than 80% of patients present increase in muscle enzymes (creatine kinase, lactate dehydrogenase, aspartate and alanine aminotransferase, aldolase) and antinuclear antibodies (ANAs) are found in more than 76% of patients^1^. Myositis-specific antibodies (MSA) and myositis-associated antibodies (MAA) can be present in different groups of JIM and their presence is associated with various clinical phenotypes^2^. There is a lack of information related to serologic profile in Latin-American patients with JIM.

**Objectives:** To describe the clinical and serologic characteristics of patients with inflammatory myopathies in a single Mexican center.

**Methods:** A single-center observational cross-sectional study was carried out, analyzing the clinical records of patients diagnosed and followed with JIM in the pediatric rheumatology clinic of our hospital between May 2008 and April 2021. General demographics, clinical and laboratory criteria at diagnosis, serologic (ANAs, MSA, and MAA), and complementary studies information were collected in order to perform descriptive analysis.

**Results:** Twelve patients were included in the study, 66% female (n = 8), median age of onset 8.5 (2 – 14) years, males were younger at diagnosis, diagnosis delay 2.9 (1 – 9) months. All patients presented muscular features, 92% (n = 11) cutaneous manifestations, 25% (n = 3) Raynaud's phenomenon, and 33% (n = 4) calcinosis. None of them presents gastrointestinal, neurological, heart, or kidney disease. Three patients presented as overlap with scleroderma.

ANA was positive in 6 patients (50%), with fine speckled (n = 3), fine speckled + centriole (n = 1), filamentous (n = 1), homogeneous nucleolar (n = 1) immunofluorescence patterns. Eight patients had a MSA and MAA panel, 3 were NXP2+, 2 MDA5+, 1 PMScl+, 1 Mi2a+ and 1 TIF1g+. The pattern associated with younger age at onset was NXP2, a higher risk of relapses occurs in MDA5 positive patients, calcinosis was frequently found in patients with FIF1g and NXP2, extrapulmonary calcifications in MDA5 and TIF1g.

**Conclusion:** This is one of the firsts reports on clinical and serological findings from JIM Latin-American patients. Presentation has a defined variability in terms of the positivity of antibodies, and there is a need to collect more data to understand MSA and MAA relevance in outcomes of this patients.

**Patient Consent Received**

No

**Disclosure of Interest**

None declared

### P217 Characteristics and outcomes of juvenile dermatomyositis in Thai children: an experience from a tertiary referral center

#### P. Khaosut^1,2^, C. Sitthi^1^

##### ^1^Department of Paediatrics, Faculty of Medicine, Chulalongkorn University; ^2^Paediatric Allergy and Clinical Immunology Research Unit, Division of Allergy and Immunology, King Chulalongkorn Memorial Hospital, Bangkok, Thailand

###### **Correspondence:** P. Khaosut

**Introduction:** Juvenile dermatomyositis (JDM) is the most common autoimmune myositis in children. However, there is a paucity of data on the clinical characteristics of JDM patients in Thailand.

**Objectives:** To describe the clinical manifestations, investigations, treatments, complications and outcomes of JDM in Thailand.

**Methods:** A bidirectional descriptive study based on past medical record review**.** All children aged < 15 years old diagnosed with JDM by Peter and Bohan criteria at King Chulalongkorn Memorial Hospital during January 2010 to December 2020 were enrolled.

**Results:** Twelve cases were identified: 67% female. The median age (IQR) at diagnosis and median time to diagnosis were 3.4 (2-6.5) years and 3.5 (1.2-8) months, respectively. The common clinical presentations were weakness (100%), Gottron papules (75%) and heliotrope rash (42%). Most patients had polyphasic disease course. More than half of the patients (67%) developed calcinosis. Lactate dehydrogenase was elevated in all cases. Muscle inflammation was found in 88% of biopsy, 75% of electromyography and 100% on MRI. The most common myositis specific Ab was anti-NXP2 (60%). Anti-NXP2 positive patients were more likely to have calcinosis. Five of 12 cases received recommended treatment with prednisolone and methotrexate at diagnosis. One patient with calcinosis had a good response with infliximab.

**Conclusion:** Our review underscores that JDM patients with younger age at diagnosis will need to be monitored closely and are likely to need aggressive long-term treatment. Prompt treatment should be managed according to the severity of symptoms in order to improve clinical outcome and prevent serious complication.

**Trial registration identifying number:** Ethical approval was obtained from hospital Institutional Review Board, Faculty of Medicine, Chulalongkorn University, Bangkok, Thailand (COA No.324/2020, IRB No.017/63).

**Patient Consent Received**

Yes

**Disclosure of Interest**

None declared

### P218 Pres juvenile dermatomyositis working party (JDM WP) achievements from 2020-2021

#### M. G. L. Wilkinson^1^, C. Papadopoulou^1^, R. Campanilho-Marques^2^, H. Sanner^3^, S. Röstlund ^4^, M. Noergaard^5^, J. Swan^6^, S. Veldkamp^7^, J. Wienke^8^, L. McCann^9^, on behalf of JDM working group

##### ^1^UCL Great Ormond Street Institute of Child Health, London, United Kingdom; ^2^Hospital de Santa Maria, Centro Hospitalar Universitário Lisboa Norte, Centro Académico de Medicina de Lisboa, Lisbon, Portugal; ^3^Department of Rheumatology, Oslo University Hospital, Oslo, Norway; ^4^Women´s Health and Allied Health Professionals Theme, Medical Unit Occupational therapy and Physiotherapy, Karolinska University Hospital, Solna, Sweden; ^5^Department of Physiotherapy (Pediatrics), Aarhus University Hospital, Aarhus, Denmark; ^6^Specialist Public Health Family Nurse, Family Nurse Partnership, Wallacetown Health Centre, Dundee, United Kingdom; ^7^Center for Translational Immunology, University Medical Center Utrecht; ^8^Center for Translational Immunology, University Medical Center, Utrecht, Netherlands; ^9^Alderhey Children's NHS Foundation Trust, Liverpool, United Kingdom

###### **Correspondence:** L. McCann

**Introduction:** The PReS JDM WP aims to bring together clinicians and researchers to improve knowledge, promote good practice and facilitate research in JDM.

**Objectives:** To provide an update of the workings of the group from September 2020-April 2021.

**Methods:** The PReS JDM working party has 236 active members. A core group of elected / co-opted representatives meet every 2 months. 3-monthly meetings are open to all members; one of which takes place during the PReS annual conference.

**Results:** Since the PReS conference 2020, work has been on-going in line with the PReS pillars, as shown in Table 1:

**Table:** 

Clinical care	Science & research	Education & training	Collaborative working
Development of a study of sleep / fatigue in JDM.	Collaborative opportunities in basic science and translational research within the PReS JDM WP group.	Narrated PowerPoint presentation on Myositis Specific Antibodies available on PReS website JDM WP tab.	CARRA / PReS JDM WP SIG: Rehabilitation and Exercise.
Survey of practice in JDM.	Updates given during meetings.	Training tools for muscle strength testing: MMT8 / CMAS, available on PReS website JDM WP tab.	CARRA / PReS JDM WP SIG: Telemedicine in JDM.
Position statement for transitional care during the Covid-19 pandemic for JDM, further developed to encompass all childhood-onset Rheumatic and Musculoskeletal Diseases, available on PReS website. Review submitted to PROJ 2021.	New tools assessing JDM activity – presentation by Prof Ravelli, JDM WP meeting, January 2021.	2021 update of JDM module for PReS/EULAR online course.	IMACS / PReS JDM WP SIG: Extension of the Single Hub and Access point for paediatric Rheumatology in Europe (SHARE) consensus guidance.

Abbreviations: CARRA - Childhood Arthritis & Rheumatology Research Alliance; IMACS - International Myositis Assessment & Clinical Studies Group; SIG – Special Interest Group.

**Conclusion:**

The PReS JDM WP has been active in collaborative projects to enhance clinical care, translational research and education / training on an international platform.

**Patient Consent Received**

No

**Disclosure of Interest**

None declared

### P219 Not just another calcinosis clinical case …

#### A. T. Melo^1,2^, I. Cordeiro^1,2^, O. Moldovan^3^, S. M. Gomes^4^, R. C. Marques^1,2^

##### ^1^Serviço de Reumatologia e Doenças Ósseas Metabólicas, HOSPITAL DE SANTA MARIA, CHULN; ^2^Unidade de Investigação em Reumatologia, Instituto de Medicina Molecular, Faculdade de Medicina, Universidade de Lisboa; ^3^Serviço de Genética Médica, Hospital de Santa Maria, Chuln, Lisbon, Portugal; ^4^Great Ormond Street Institute of Child Health, University College London, London, United Kingdom

###### **Correspondence:** A. T. Melo

**Introduction:** Calcinosis cutis is described as the deposition of calcium salts in the skin, subcutaneous tissue, muscles and visceral organs. Calciphylaxis, metastatic, dystrophic, idiopathic, and iatrogenic are the 5 subtypes of calcinosis cutis.

**Objectives:** To present a severe widespread calcinosis clinical case.

**Methods:** Data were collected from the hospital clinical records.

**Results:** We describe the case of a 21-year-old man previously healthy until the age of 2 years old when he started with stony subcutaneous nodules, initially located in the lower limbs but with rapid progression to upper limbs, trunk and neck. He had no evidence of myositis, dysphagia, dysphonia or skin rashes. He had normal serial blood tests including creatine-kinase and aldolase. The results of several lesion biopsies revealed deep calcifications underlined by an inconstant inflammatory lymphohistiocytic infiltrate, compatible with calcinosis. Calcinosis lesions continued to disseminate, despite sequential treatment with calcium channel blockers, oral bisphosphonates and sodium thiosulfate. Immunosuppression with steroids, methotrexate, cyclosporine, intravenous immunoglobulin and etanercept didn´t improve the calcinosis progression. Infliximab was also started but had to be suspended due to an allergic reaction. Whole exome sequencing was performed as a trio (patient and parents) but no relevant mutations were found thus far, including in skeletal dysplasia and interferonopathy related genes. The calcinosis lesions continued to progress in terms of number and size, involving skin and muscles of the limbs, neck, trunk and abdomen with recurrent episodes of skin infections due to calcinosis ulceration and several lipodystrophy areas with severe joint limitation and secondary osteoarthritis. Continuous blood test monitoring showed no relevant results except for a positive NXP2 myositis specific antibody.

**Conclusion:** The treatment of calcinosis cutis is challenging. Disseminated calcinosis often requires systemic treatment and several treatment options have already been tried. Several rare genetic diseases were seemingly discarded by exome sequencing, however the presence of mutations affecting intronic regions or in potential novel genes cannot be excluded.

**Disclosure of Interest**

A. Melo: None declared, I. Cordeiro Employee of: Bristol Myers Squibb, O. Moldovan: None declared, S. Gomes: None declared, R. Marques: None declared

### P220 Single center retrospective study of the juvenile idiopathic inflammatory myopathies

#### M. Kaleda, I. Nikishina, S. Arsenyeva

##### Pediatrics, V.A. Nasonova Research Institute of Rheumatology, Moscow, Russian Federation

###### **Correspondence:** I. Nikishina

**Introduction:** Juvenile idiopathic inflammatory myopathies (JIIM) include juvenile dermatomyositis (JDM), the most common form of JIIM, juvenile polymyositis (JPM) and juvenile myositis overlapping (OM) with another rheumatic diseases (RD), whose have not been well characterized.

**Objectives:** to summarize clinical phenotypes, spectrum of autoantibodies, therapeutic options in patients with JIIM based on the data from the single pediatric rheumatological center.

**Methods:** The retrospective study included all patients (pts) of our center with different kinds of JIIM diagnosed over the last 5 years (2016-2020).

**Results:** 37 pts were identified as having JIIM: 23 - JDM, 1 –JPM and 13 – OM. OM included 3 pts with systemic lupus erythematosus (SLE) (23%), 5 with mixed connective tissue disease (38.5%), 2 with overlap JIA+JDM (15.4%), 2 – JIA+JDM+ Sjogren's syndrome (15.4%), 1 – SLE+JDM (7.7%). The overall sex ratio was M: F = 1:1.7 without difference between JDM and OM (p›0.05). The median age at the onset was 6.9 years [interquartile range (IQR) 3.25; 8.4] in pts with JDM, 11.3 years [4.0;14.0] in pts with OM. The median disease duration at the onset of myopathy was 3.0 months [0.25; 6.0] in pts with JDM, 9.0 months [6.0;14.0] – OM. A boy diagnosed with JPM got sick at the age of 7. All pts had proximal myopathy. Dysphagia observed in 12 (52.2%) pts with JDM, 2 (15.4%) – OM. Only 5 (21.7%) pts with JDM had nasal intonation. The classical rash (heliotrope rash and/or Gottron’s papules) was in all pts with JDM, 4/13 (30.8%) - OM. HRCT abnormalities were found in 5 (21.7%) pts with JDM, in 5 (38.5%) pts with OM. The restrictive lung involvement was found in 8(34.8%) pts with JDM and 5(38.5%) pts with OM. Serum muscle enzymes were increased as follows: CK 235.5 U/L [156; 1345.25] in pts with JDM, 170.0 U/l [121.0; 230.0] – OM; ALT – 51.0 U/L [35.0;94.0] in pts with JDM, 80.0 [54.0; 160.0] – OM; AST –72.0 U/L [50.0;120.0] in pts with JDM, 99.0 [78.0; 125.0] – OM; LDH – 538.0 U/L [425.0;840.0] in pts with JDM, 398.0 [328.0; 568.0] – OM. EMG abnormalities were observed in 69.6% of pts with JDM, 30.8% - OM. Muscle MRI revealed myositis in 10 pts with JDM, 2 pts with OM. In a patient with JPM the diagnosis was confirmed by muscle biopsy. Typical for JIIM nailfold capillaroscopic changes had 20/21 pts with JDM, 7/13 – OM. Calcinosis developed in 7(30.4%) pts, whose diagnosis was verified late. Antinuclear antibodies (ANA) were positive in 13 pts (56.5%) with JDM, 12 pts (92.3%) with OM. 1 patient had anti-Jo-1 autoantibodies (meets the criteria for antisynthetase syndrome), 1 – anti-PM-Scl. 1 patient with SLE had anti-Rib-P autoantibodies. Patient with JPM had anti-SRP autoantibodies. All pts received glucocorticoids (GC), 81.0% - methotrexate, 18.9% - hydroxychloroquine, 8.1% - cyclophosphamide, 8.1% - cyclosporine, 2.7% - mycophenolate mofetil, 2.7% - azathioprine, 67.6% - IVIG. Biologics (B) were started due to insufficient efficacy of previous therapy in 26% pts with JDM, all pts – with OM (total 51.3% with JIIM). The median age at start of B was 12.3 years [IQR 9.5; 15.0]. The median disease duration prior to treatment with B was 2.25 years [IQR 0,8; 3.6]. 58.8% of pts received rituximab, 41.2% - abatacept. All pts have reached inactive status of the disease. Anti-Jo-1 antibodies normalized in a patient with antisynthetase syndrome, who received abatacept for 17 months.

**Conclusion:** The spectrum of JIIM in our study seems to be similar to other studies. Pts with JDM, as the most frequent subtype, had a younger age at onset, more significant increase the level of CK and LDH, more often had classical rash and typical nailfold capillaroscopic changes. However, pts with OM more often had lung involvement and were more likely to require intensive therapy, included B.

**Disclosure of Interest**

None declared

### P221 Was it COVID19 or the vaccine? a rare case of SARS-COV2 infection or post-vaccine triggered flare in juvenile dermatomyositis

#### E. Potts, L. PattersonBrown, A. Jones, R. Florida-James, V. Shivamurthy, N. Wilkinson

##### Paediatric Rheumatology, Evelina London Children's Hospital, Guy's and St Thomas' NHS Foundation Trust, London, United Kingdom

###### **Correspondence:** E. Potts

**Introduction:** Vaccines and infection can cause flares of inflammatory conditions.

**Objectives:** We describe a rare, rapid flare of Juvenile Dermatomyositis (JDM) preceded by confirmed Sars-Cov2 antibodies (asymptomatic infection or first vaccination).

**Methods:** Data was extracted from electronic medical records and a literature review undertaken.

**Results:** We present a 16 year old female of Bangladeshi origin with JDM diagnosed August 2019 (CK 29691, CMAS 23; Mi-2b, Ku, Mi-2a positive). On a background of suboptimal control with low grade inflammation (CK 659; CMAS 51) medications were altered in May 2020 adding Adalimumab and Mycophenolate Mofitil (MMF), to Methotrexate and Intravenous Immunoglobulin (IVIG). CK rose in August 2020 (15969). IVIG continued and physiotherapy input increased. After a stable 5 months (CK 100-300), CK grew to 2307 but as CMAS remained 50 treatment was not altered. Four weeks prior (Jan 2021) the patient received their first dose of Pfizer SARS-CoV2 vaccination. A family member was confirmed to be COVID-19 positive 8 weeks prior and the family isolated together. Our patient did not report any known COVID-19 symptoms.

In March 2021 she presented with a 4 day history of acute active inflammation as demonstrated by malaise, increased rash, muscle pain and reduced function (CMAS 3, CK 52848). She was admitted for Multidisciplinary management of an acute flare and found to be SARS-CoV2 IgG positive consistent with prior infection. Adalimumab and MMF were swopped with tacrolimus alongside intravenous methylprednisolone, oral prednisolone and physiotherapy. CMAS scores remained considerably low (5) over the following 6 weeks, with a rapid rise (34) at week 7 with the patient discharged home.

Literature review has identified a case of Anti-MDA5 JDM increased interstitial lung disease associated with active SARS-CoV2 infection^1^. Similarly there is a case presentation of Macrophage Activation Syndrome in Systemic Onset Juvenile Idiopathic Arthritis temporally associated with SARS-CoV2 infection^2^. There are no reported cases of vaccine mediated hyperinflammation of muscle or skin disease in JDM or Paediatric Inflammatory Multisystem Syndrome Temporally associated with COVID-19 (PIMSTS)^3^.

**Conclusion:** This was a rare case of a rapid deterioration in function and hyperinflammation at a time of PIMSTS and other similar reactions in adolescents with Rheumatic diseases. We hypothesise that this such case may have been triggered by recent asymptomatic COVID-19 infection or following SARS-Cov2 vaccination.

Quintana-Ortega C, Remesal A, Ruiz de Valbuena M, de la Serna O, Laplaza-González M, Álvarez-Rojas E, Udaondo C, Alcobendas R, Murias S. Fatal outcome of anti-MDA5 juvenile dermatomyositis in a paediatric COVID-19 patient: a case report. Mod Rheumatol Case Rep. 2021 Jan;5(1):101-107. doi: 10.1080/24725625.2020.1832755. Epub 2020 Oct 20. PMID: 33019894

Akturk H, Tanyildiz M, Erbey F, Tasdemir M, Celikyurt A, Gonen E, Bilge I. Macrophage Activation Syndrome in a Child with Juvenile Idiopathic Arthritis Secondary to SARS-CoV-2. J Trop Pediatr. 2021 May 24:fmab049. doi: 10.1093/tropej/fmab049. Epub ahead of print. PMID: 34028559

Harwood R, Allin B, Jones CE, Whittaker E, Ramnarayan P, Ramanan AV, Kaleem M, Tulloh R, Peters MJ, Almond S, Davis PJ, Levin M, Tometzki A, Faust SN, Knight M, Kenny S; PIMS-TS National Consensus Management Study Group. A national consensus management pathway for paediatric inflammatory multisystem syndrome temporally associated with COVID-19 (PIMS-TS): results of a national Delphi process. Lancet Child Adolesc Health. 2021 Feb;5(2):133-141. doi: 10.1016/S2352-4642(20)30304-7. Epub 2020 Sep 18. Erratum in: Lancet Child Adolesc Health. 2021 Feb;5(2):e5. PMID: 32956615; PMCID: PMC7500943

**Patient Consent Received**

Yes

**Disclosure of Interest**

None declared

### P222 Interferon score in patients with juvenile dermatomyositis

#### R. Raupov^1^, E. Suspitsin^1,2^, R. Mulkidzhan^2^, M. Kostik^1,3^

##### ^1^Saint-Petersburg State Pediatric Medical University; ^2^Molecular diagnostics, N.N.Petrov Institute of Oncology; ^3^Almazov National Medical Research Centre, Saint-Petersburg, Russian Federation

###### **Correspondence:** R. Raupov

**Introduction:** two main mechanisms of pathogenesis have been proposed for juvenile dermatomyositis (JDM), namely, interferon type I- (IFN-I) and antibody-centered. The activity of IFN-I signaling pathway can be measured using IFN-score.

**Objectives:** to evaluate IFN-score in patients with JDM.

**Methods:** 15 patients (5 boys and 10 girls) were enrolled in the study. Clinical and laboratory parameters including disease activity (CMAS-childhood myositis assessment tool, aCAT- abbreviated cutaneous assessment tool) and treatment efficiency were assessed. IFN-score was assessed by RT-PCR quantitation of 5 IFN I-regulated transcripts; median relative expression of ≥2 was considered as a cut-off.

**Results:** The median age of patients was 9.0 (5.9-10.8) years (Table 1). One of the patients (#15) had widespread soft-tissue calcinosis, another one (#10) had combination of JDM and systemic sclerosis. The median time to IFN-score assessment was 21 (4; 51) months. Patients were divided into groups with high (n=12) and normal IFN-score (n=3). The first group had higher aCAT activity index - 3 (2-6) vs 0 (0-0), p=0.012. Positive correlations with IFN-score value were found for aCAT activity (p=0.014) and arthritis (p=0.018). IFN-score was 11.6 (3.1; 27.7) in patients with active disease vs 1.8 (1.6; 2.7) in those in remission (p=0.040).

Two patients (#2, #3) with recurrent skin activity were treated with jak-inhibitors (Tofacitinib). Patient #2 had complete response, while patient#3 had a partial response.

In 5 patients (# 2-6) IFN-score was assessed in dynamics. The value decreased during the treatment from 8.8 (2.0; 15.5) to 4.2 (1.3; 6.8), p=0.043.

**Conclusion:** in a small group of JDM patients IFN-score was associated with disease activity, specifically with skin involvement.

Limitations of the study: number of patients, widely variable time from disease onset to IFN-score measurement, no anti-TIF1 and anti-NXP2 antibodies assessment performed.

**Acknowledgments**

This work was supported by the RSF grant № 20-45-01005

**Patient Consent Received**

No

**Disclosure of Interest**

None declared

**Table 1 (abstract P222).** See text for description

	Sex/age	Time to IFN-score eval. (m)	IFN- score	aCAT (activity/damage)	CMAS	CS (mg/kg)
1	m/10y3m	45	1.98	1/1	-	0,2
2	f/6y7m	10	33.75	6/0	45	0,7
3	f/5y3m	21	10.8	6/1	-	1
4	m/13y4m	93	15.52	3/1	34	0
5	m/4y10m	3	11.62	2/0	-	0
6	f/8y9m	3	44.57	9/0	10	0
7	m/16y1m	22	27.72	2/1	46	0
8	f/3y5m	16	1.26	0/1	4	0,85
9	f/10y10m	56	3.45	2/0	-	0,1
10	f/ 10y8m	51	1.54	0/1	48	0
11	f/9y6m	28	1.67	0/1	46	0,1
12	f/ 6y2m	8	3.13	3/0	38	1,1
13	f/7y10m	3	2.53	5/0	18	1
14	f/3y6m	4	8.88	5/0	17	0
15	m/9y4m	36	15.06	3/0	34	0

### P223 Onset and relapse of juvenile dermatomyositis following SARS-COV-2 infection

#### M. Rodero^1^, S. Pelleau^2^, V. Bondet^3^, C. Gitiaux^4^, A. Fayand^1^, M. White^2^, D. Duffy^3^, I. Melki^5,6,7^, B. Bader-Meunier^7,8,9^

##### ^1^Chimie & Biologie, Modélisation et Immunologie pour la Thérapie (CBMIT), UMR8601, CNRS; ^2^Infectious Diseases Epidemiology and Analytics Unit, Department of Global Health; ^3^Translational Immunology Lab, Institut pasteur; ^4^Department of Paediatric Neurophysiology, Necker-Enfants Malades Hospital, AP-HP; ^5^Laboratory of Neurogenetics and Neuroinflammation, Imagine Institute, Paris University; ^6^General Paediatrics, Department of Infectious Disease and Internal Medicine, Robert Debré, Hospital, AP-HP; ^7^Reference center for Rheumatic, AutoImmune and Systemic diseases in children (RAISE); ^8^Laboratory of Immunogenetics of Paediatric Autoimmunity, Imagine Institute, Inserm; ^9^Department of Paediatric Hematology-Immunology and Rheumatology, Necker-Enfants Malades Hospital, AP-HP, Paris, France

###### **Correspondence:** M. Rodero

**Introduction:** A working hypothesis is that juvenile dermatomyositis (JDM) is a type 1 interferon driven inflammatory response, triggered by one or more environmental stimuli, such as infection.

**Objectives:** We aimed to test the hypothesis that SARS-CoV-2 infection could promote JDM onset or relapse.

**Methods:** We studied SARS-CoV-2 infection history in all JDM patients seen in our center for disease onset (n=6) or relapse (n=4) since the start of the pandemic. IgG and IgM directed against whole spike protein, spike Receptor Binding Domain (RBD), spike S2 subunit, nucleocapsid protein (NP) and Membrane glycoprotein (ME) were measure in the plasma by multiplex bead-based assay at diagnosis. IFNα2 level in the plasma was measure by digital ELISA.

**Results:** Out of the 10 patients we identified concomitant infection by SARS-CoV-2 with disease onset in one patient, and concomitant infection by SARS-CoV-2 with disease relapse after 8 years out of treatment in one other. IFNa2 dosages in the plasma of these two patients revealed abnormally elevated concentrations (73476 fg/ml and 4612fg/ml respectively, median active JDM: 491fg/ml).

**Conclusion:** Our results strongly suggest that SARS-CoV-2 infection could trigger the development of JDM, possibly through induction of IFNα.

**Patient Consent Received**

Yes

**Disclosure of Interest**

None declared

### P224 Juvenile dermatomyositis with anti-MDA5 antibodies and lung involvement

#### I. Sanjurjo-Jimenez^1^, E. Barral Mena^2^, E. Calvo Aranda^3^, A. Navas Carretero ^2^, M. Á. Martín Díaz^4^, P. S. Tirado Zambrana^5^

##### ^1^Pediatrics, Infanta Leonor University Hospital. Madrid, Spain; ^2^Pediatrics; ^3^Rheumatology; ^4^Dermatology; ^5^Anatomical Pathology, Infanta Leonor University Hospital., Madrid, Spain

###### **Correspondence:** I. Sanjurjo-Jimenez

**Introduction:** Juvenile dermatomyositis is an inflammatory autoimmune disease that mainly affects the skin and muscles. Some autoantibodies may help predict damage to other organs and establish the prognosis.

**Objectives:** To present a case of juvenile dermatomyositis with anti-MDA5 and lung involvement.

**Methods:** Case report.

**Results:** A 9-year-old boy presented with constitutional symptoms of one month of evolution and skin rash (face, hands, elbows, knees). He also had aphthous stomatitis and limiting pain in the knees, ankles, wrists and fingers. No gastrointestinal or respiratory symptoms. Physical examination revealed a heliotrope rash, Gottron’s papules and inverse palmar Gottron sign over the finger flexor tendons. Wrists and thumbs were swollen, with limitation in range of motion. Only neck flexion weakness was found, with no findings in the rest of the muscle and joint examination.

In the laboratory test we found mild lymphopenia and elevated aldolase, CK, LDH, ferritin, and transaminases levels. CRP 1.8 mg/L. Weak positive anti-nuclear (ANA 1/80) and anti-MDA5 antibodies were detected. His pulmonary function tests were normal as well as his chest x-ray. When anti-MDA5 antibodies positivity was confirmed and because of its association with interstitial lung disease, a high-resolution computed tomography was performed, and ground-glass opacities were revealed.

The skin biopsy showed vacuolar interface, perivascular infiltrate, and leukocytoclasia, compatible with dermatomyositis. The magnetic resonance of the shoulder girdles showed patchy muscle involvement, and the electromyogram demonstrated a myopathic pattern. The capillaroscopy showed a group of capillaries with dilation (4th finger, left hand). Synovitis during the ultrasound assessment was observed, with effusion and Doppler signal in radiocarpal joints and in the left 4th metacarpophalangeal.

After confirming the diagnosis of juvenile dermatomyositis treatment was started with hydroxychloroquine, corticosteroids, methotrexate and tacrolimus. The PNEUMOVAX 23 was completed and infection prophylaxis was started with Trimethoprim/sulfamethoxazole.

Two months later the arthritis and blood test were resolved, he continues with skin inflammatory activity. Without any respiratory symptoms, the pulmonary function test remains normal.

**Conclusion:** The diagnosis of juvenile dermatomyositis is based on the Bohan and Peter criteria. Our patient presented muscle weakness, typical skin rash, elevated muscle enzymes and a myopathic electromyographic pattern. It may be accompanied by constitutional symptoms, skin ulcers, calcinosis, arthralgia/arthritis or periungual capillary alterations (1,2).

Anti-MDA5 antibodies are associated with a poor prognosis, with the development of skin ulcers and rapidly progressive lung interstitial disease. Its prevalence in Europe reaches 12-40% in adults and children with dermatomyositis. In Great Britain it is estimated to occur in 7% of juvenile dermatomyositis (3).

Chest CT is essential for the early detection of lung involvement, even in the absence of respiratory symptoms (3, 4).

The treatment is based on high-dose corticosteroids and immunosuppressants. The therapeutic response is determined by the symptomatic improvement and the laboratory (muscle enzymes) and pulmonary tests (1,2). There is no clear consensus on the radiological follow-up of lung involvement.

**Patient Consent Received**

Yes

**Disclosure of Interest**

None declared

### P225 Anti-NXP2 is the most common type of autoantibody in a North Indian cohort of children with juvenile dermatomyositis – our preliminary experience from a tertiary care centre in North-West India

#### M. Sudhakar, P. Vignesh, G. Anjani, R. Kumrah, N. Johnson, A. Jindal, D. Suri, A. Rawat, A. Gupta, S. Singh

##### Department of Pediatrics, Postgraduate Institute of Medical education and Research, Chandigarh, Chandigarh, India

###### **Correspondence:** M. Sudhakar

**Introduction:** Juvenile Dermatomyositis (JDMS) is a multisystem autoimmune vasculopathy characterised by symmetrical skeletal muscle weakness, typical skin changes, and variable internal organ involvement. Identification of predictive factors for severe disease at onset of disease would be helpful to judge the duration and level of immunosuppression needed. Several clinical, laboratory markers for predicting disease phenotype, and course were available, however, none proved to be most useful. Myositis specific, and associated antibodies are one such biomarkers recently that could classify idiopathic inflammatory myopathies into different classes, and possibly predict the clinical course also.

**Objectives:** Descriptive study on myositis specific antibodies in patients with JDMS.

**Methods:** A single centre retrospective study compiling data of patients with JDMS for a period of 3 years (2018-21) who underwent immunodot assay for myositis antibodies by a 16-antigen kit (Euroline Autoimmune Inflammatory Myopathies 16 Ag, Euroimmun, Lübeck, Germany). The following antigens were included Mi2; myeloma differentiation-associated gene 5 (MDA5), transcription intermediary factor 1 gamma (TIF1γ), NXP2, small ubiquitin-like modifier 1 activating enzyme (SAE), aminoacyl- tRNA synthetase antibodies (Jo1, PL7, PL12, EJ, OJ), and anti- signal recognition peptide (SRP) antibodies. The following myositis associated antibodies (MAA) (anti-Ro, anti-Ku, and anti-PM-Scl antibodies) were also included in the panel. Analysis was done comparing the clinical, laboratory, and outcome variables.

**Results:** We analysed 30 children diagnosed with JDMS in this study. Twenty- four patients (80%) had positive myositis antibodies, and of these 8 had > 1 myositis antibody (33.3%) positivity. Six patients (20%) were negative for myositis antibodies. The frequency of antibodies include: anti-NXP-2 (n=6, 25%); anti- MDA5 antibody (n=5, 20.8%); anti-Ro antibody (n=4, 16.6%); PM- Scl antibodies (n=4, 16.6%); Mi-2 antibody (n=3, 12.5%); anti- TIF1γ antibodies (n=3, 12.5%); anti- Ku antibody (n=2, 8.3%); anti- PL antibody (n=2, 8.3%); anti- SAE antibodies (n=2, 8.3%); and anti- SRP antibody (n=1, 4.1%). Two patients (2/5 patients, 66.6%) with MDA5 had interstitial lung disease (ILD), when compared to 1 patient (4%) without MDA5 had ILD. Both the patients were asymptomatic clinically. A positive MDA-5 had a 40% chance of predicting a positive imaging for ILD. Both presence of MDA-5, and inverse Gottron were predictive of ILD in 50% of patients. None of the patients in our cohort had amyopathic JDMS presentation. Three of 5 patients (60%) with MDA-5 positivity had inverse Gottron whereas only 32% of patients without MDA-5 positivity had inverse Gottron papules. Two patients with NXP-2 positivity (33%) had calcinosis whereas only 12.5% of patients without NXP2 positivity had calcinosis.

**Conclusion:** We report 80% positive rate of myositis antibodies in children with JDMS. Antibodies against NXP-2 antigen followed by MDA-5 were the commonest antibodies in our cohort. A positive anti-MDA-5 antibody had predicted for ILD, and a positive NXP-2 antibody was associated with higher frequency of calcinosis.

**Patient Consent Received**

No

**Disclosure of Interest**

None declared

### P226 Thrombocytopenia at diagnosis is a potential biomarker to predict severe disease course in children with juvenile dermatomyositis: our experience from Chandigarh, North India

#### M. Sudhakar, P. Vignesh, G. Anjani, N. Johnson, A. Jindal, D. Suri, A. Rawat, A. Gupta, S. Singh

##### Pediatrics, Postgraduate Institute of Medical education and Research, Chandigarh, Chandigarh, India

###### **Correspondence:** M. Sudhakar

**Introduction:** Juvenile Dermatomyositis (JDMS) is a multisystem autoimmune vasculopathy with less predictable clinical course. Identification of predictive factors for severe disease at onset of disease would be helpful to judge the duration and level of immunosuppression needed.

**Objectives:** To evaluate whether thrombocytopenia at diagnosis of JDMS predict severity and disease course.

**Methods:** A single centre retrospective study compiling data of 131 patients with JDMS for last 30 years was carried out. Analysis was done comparing clinical, laboratory, and outcome variables between patients who had thrombocytopenia (<150x10^9^/L), and patients with normal platelets at time of diagnosis of JDMS.

**Results:** Fourteen patients (10.7%) with JDMS had thrombocytopenia, and rest 117 patients (89.3%) had normal platelets at diagnosis. None of 14 patients had associated sepsis, overlap syndrome or macrophage activation syndrome. Patients with thrombocytopenia had later age of onset of symptoms and diagnosis (9.9 vs 6 years, p0.008; 10.1 vs 7 years, p-0.031). However, median delay in diagnosis was less in thrombocytopenia group (4.5 vs 6 years, p 0.016). Number of patients with heliotrope rash (9 vs 80, p-0.767); other rash (9 vs 79, p-1.000); Gottron papule (9 vs 93, p-0.303); calcinosis (1 vs 32, p-0.188), were comparable between groups. Periorbital swelling [10 (71%) vs 49 (41.8%), p-0.047] and fever [10 (71%) vs 50 (42.7%), p-0.050] were seen more with thrombocytopenic patients.

Number of patients with respiratory muscle weakness [5 (35.7%) vs 6 (5%), p-0.002], and pharyngeal muscle weakness [9 (64.2%) vs 40 (34.1%), p-0.040] were also significantly high in thrombocytopenic group. Though the number of patients with proximal muscle weakness were comparable, median manual muscle test score was low in thrombocytopenic group (28 vs 48, p-0.001). Moreover, a high proportion of children in thrombocytopenic group had anasarca at presentation [3 (21%) vs 2 (1.7%), p-0.009]. Patients with gastrointestinal vasculopathy (hematemesis, melena) were more in thrombocytopenic group [4 (28.5%) vs 1 (0.8%), p-0.001]. One patient in the thrombocytopenic group had biopsy-proven IgA nephropathy, and another had concomitant autoimmune hemolytic anaemia. Platelet counts rapidly improved with institution of immunosuppressive therapy for JDMS.

Regarding treatment, both the groups were comparable in terms of frequency and duration of corticosteroid usage; frequency of methotrexate, cyclophosphamide, mycophenolate mofetil and intravenous immunoglobulin. Two patients with severe respiratory involvement in thrombocytopenic group was also administered with rituximab due to severity of illness. Though treatment details were comparable, the median time required to achieve remission was more in thrombocytopenic group (8 months vs 4.5 months, p 0.011).

Rate of relapse was more in thrombocytopenic group (35% vs 15%). Three patients (21.4%) in thrombocytopenic group had succumbed to illness over a median follow-up period of 5.5 years, and 7 patients (5.9%) in normal platelet group had died during illness course.

**Conclusion:** Children with JDMS who had thrombocytopenia at diagnosis had an older age of disease onset and had lesser delays in diagnosis compared to children with normal platelet counts. These children also had increased incidence of GI vasculopathy and respiratory muscle involvement requiring mechanical ventilation. Rate of future disease relapses and mortality were more in children who had low platelets at the time of diagnosis. Thus, thrombocytopenia could be considered a potential laboratory marker in patients with JDMS to predict the clinical course, relapse, and outcome.

**Patient Consent Received**

No

**Disclosure of Interest**

None declared

### P227 Ipomiopathic anti-MDA5 juvenile dermatomiositis with periostitis in treatment with mycophenolate mophetile: a case report

#### A. Uva, L. Mambelli, M. Mainetti, A. Iacono, F. Marchetti

##### Pediatric Unit, "Santa Maria delle Croci" Hospital- Ravenna, Ravenna, Italy

###### **Correspondence:** A. Uva

**Introduction:** Juvenile dermatomyositis (JDM) is the most common paediatric inflammatory myopathy and it is characterized by distictive skin rashes and symmetrical proximal muscle weakness. The most widely used diagnostic criteria are those suggested by Bohan and Peter, although they have not been tested for sensitivity or specificity against all appropriate disease confounders. Recently, myositis specific antibodies (MSA) have been detected in patients with dermatomyositis. Among them, the presence of anti-MDA5 antibodies is associated with an increased risk of skin and oral ulceration, arthritis, milder muscle disease and interstitial lung disease (ILD). However, few data concerning presentation, treatment and follow up are available on anti-MDA5 antibodies JDM patients.

**Objectives:** To describe the first patient with anti-MDA5 antibodies JDM and associated periostitis

**Methods:** We performed a full blood count, liver and muscle enzymes. MSA has been detected by immunofluorescence assay. A whole body (WB) MRI was used to highlight muscle inflammation. A CT-chest scan was required to rule out the presence of Interstitial Lung Disease (ILD)

**Results:** A 10 years old boy was admitted to our Pediatric Unit because of a three month story of pain of the wrists and legs and asthenia associated to the presence of erythematous-xerotic lesions of hands, elbows, knees and ear auricles. On physical examinations, he presented both hand arthritis, pain at digitopression of the left tibia, Gottron’s papules, heliotropic rash and erythematous-xerotic lesion of elbows. No muscle weakness was noted (MMT8 78/80, CMAS 48/52). His blood count revealed mild lymphopenia (L 1260/mmc) and an increase in liver exams (ALT 180 U/I), with normal values of CPK and LDH. CRP, ESR, C3 and C4 were in ranges too. Infective and paraneoplastic causes were ruled out; a punch biopsy of the skin of the elbow was obtained to exclude psoriasis and cutaneous sarcoidosis. The autoimmune panel was negative for ANA antibodies. Due to the presence of Gottron’s papule, MSA were performed, revealing the positivity of anti-MDA5 auotoantibodies. The boy underwent a WBMRI that enlightened the presence of inflammation of both peroneal muscles and left tibia periostitis, which was very painful. No signs of calcinosis were found. A diagnosis of anti-MDA5 autoantibodies JDM was held. Because of a suspicious of ILD, a CT chest scan was made, but it was negative. He started three glucocorticoid pulses (20 mg/kg) later tapered orally, intravenous immunoglobulin (2 g/kg) and subcutaneous methotrexate, which was dismissed 2 weeks later due to an increase in liver enzymes. Mycophenolate mophetile (600 mg/mq every 12 hours) was preferred with good response on both arthritis and skin lesions.

**Conclusion:** Anti-MDA5 autoantibodies identify a distinct clinical phenotype of children affected by JDM. Data from the Japanese and English cohort differ in prognosis: Caucasian children seem to have less likely ILD than the East-Asians. In particular, English JDM patients reached remission according to PRINTO criteria after 2 years of treatment, although this is not clearly defined. To the best of our knowledge, this is the first case of anti-MDA5 positive JDM patient with associated periostitis. We described periostitis as a potential novel feature of anti-MDA5 autoantibodies JDM. Moreover, mycophenelate could be used in children as a first line therapy, even in those that present with arthritis. Our case expands the phenotype of anti-MDA5 autoantibodies JDM and supports the evidence that mycophenolate could represent a good therapeutic choice in these patients.

**Patient Consent Received**

Yes

**Disclosure of Interest**

None declared

### P228 Anti-MDA5 antibody-positive juvenile dermatomyositis complicated with progressive interstitial lung disease: a case successfully treated with mycophenolate mofetil and tacrolimus

#### K. Yamazaki^1^, T. Yamazaki^2^

##### ^1^Division of Rheumatology and Allergology, Department of Internal medicine, St. MariannaUniversity School of Medicine, Kawasaki; ^2^Pediatrics, Tokyo Medical University, Tokyo, Japan

###### **Correspondence:** K. Yamazaki

**Introduction:** Anti-Melanoma differentiation associated gene 5 (MDA5) autoantibody (Ab) was identified in a small population of patients with juvenile dermatomyositis (JDM) in Caucasians and was associated with skin ulceration and arthritis. Whereas anti-MDA5 Ab was reported to be related to rapidly progressive (RP)-ILD and a poor prognosis in East-Asian patients with JDM.

**Objectives:** We aim to report a case of 4- year-old boy diagnosed with JDM complicated with RP-ILD who was successfully treated with Mycophenolate mofetil (MMF) and Tacrolimus (Tac).

**Methods:** A case report and a literature review. Parental informed consent was obtained.

**Results:** A 4-year-old boy was referred to our hospital for eruption and fever. At the first visit, he exhibited Gottron’s sign on the dorsum of his hands, periungual erythema, auricular ulcer, and symmetric muscle weakness of the proximal lower extremities. The level of anti-MDA5 Ab was high (138 index), where the cut-off value is 32 index in the EIA. His chest CT demonstrated ground-glass opacities in the right upper lung lobes. He was diagnosed as JDM complicated with interstitial lung disease, and we initiated treatment with methylprednisolone (PSL) pulse, cyclosporine (CyA), and intravenous pulse administration of cyclophosphamide (IVCY). Just after the 2nd IVCY, he had syndrome of inappropriate secretion of antidiuretic hormone and posterior reversible encephalopathy syndrome, and so we had to alternatively treat him with PSL, MMF, and Tac. His myositis and interstitial pneumonia significantly improved six months after the first administration of MMF. Forty-eight months later, he achieved drug-free remission.

**Conclusion:** A standard treatment for DM/JDM complicated with RP-ILD has not yet been established. The treatment with a combined immunosuppressive regimen of high dose PSL, CyA, and IVCY has been proposed and administered beginning in the early phase of this disease in Japan. The combined immunosuppressive regimen brought a significant improvement in the mortality of JDM complicated with RP-ILD. Our result suggests that MMF can be considered a new therapeutic option for JDM complicated with progressive ILD, particularly when unfavorable conditions are anticipated due to IVCY.

**Patient Consent Received**

Yes

**Disclosure of Interest**

None declared

## e-Poster viewing: Systemic lupus erythematosus and antiphospholipid syndrome

### P229 Coexistence of tumid lupus erythematosus with systemic lupus erythematosus: a report first case of tumid lupus in a libyan female child

#### A. M. A. Abushhaiwia^1^, N. Abushhiwa^2^, Y. AElfawires2^3^, A. A. Abdullah^4^, A. M. Kouklah^4^

##### ^1^Paediatric Rheumatology, Tripoli Children's Hospital,Faculty of Medicine, Tripoli university; ^2^Pathology, Faculty of Medicine: University of Tripoli; ^3^Paediatric Rheumatology, Tripoli Children's Hospital, Faculty of Medicine,University of Tripoli; ^4^Paediatric Rheumatology, Tripoli Children Hospital, Tripoli, Libya

###### **Correspondence:** A. M. A. Abushhaiwia

**Introduction:** Lupus erythematosus tumidus (LET) is a rare type of cutaneous lupus erythematosus (CLE), which has commonly, been under- estimated, and neglected in clinical practice. LET is generally, an independent disease, although it has been reported to co-exist with discoid lupus erythematosus (DLE), and SLE. Herein, report a case of 11-year-old female child diagnosed as of LET associated with alopecia significantly improved after treatment with hydroxychloroquine in combination with oral corticosteroids and MMF.

**Objectives:** We report a case of LET converted from DLE with diverse clinical manifestations and unusual pathologic findings.

**Methods:** case report study

**Results:** An 11-year-old female child born of consanguineous marriage was brought to our Rheumatology clinic with the complaint of erythema and rashes present on the face her cheeks, bridge of the nose and forehead. Subsequently, spread too much of the similar lesions were present over the extremities since 3 months. Gradually, which healed with hyper-pigmentation. Lesions occur in hairy areas such alopecia. Along with the skin lesions, the child also developed painful oral ulcers, which caused considerable difficulty in eating, poor appetite and weight loss, there was a significant history of worsening of facial skin lesions during sun-exposure and discoloration of both hands and feet on prolonged cold exposure. She later developed low-grade fever, which was not associated with chills or rigor along with joint pain over large joints (fingers of her both hands, knee and ankle). She had undergone some local treatment but was not relieve. On general examination, the child was thin-built (30 kg). Generalized pallor and no cervical lymphadenopathy were present. Her developmental milestones were normal. Cutaneous examination showed diffuse hair loss (lupus hair) and at some places patchy alopecia Erythematous rash was seen all over the face with (malar rash/butterfly rash) involving both cheeks and bridge of the nose and forehead. Showed post hyperpigmentation, Photosensitivity and Reynaud’s patches were seen on the both hands and foot. Laboratory investigations revealed WBC = 3.600cells/mm3 (58% neutrophils, 28% lymphocytes), hemoglobin = 10.5g/dL, platelets = 313,000/mm3, erythrocyte sedimentation rate (ESR) was elevated 90 mm/h, CRP was negative; specific tests –ANA was positive with ANA titer = 1:160 though anti- double stranded DNA (dsDNA) was positive with titer 554 IU\ml, anti-Sm AB was positive >330 U\ml (REF <7), U1-snRNP was positive 88U\ml (REF <5), Scl70 was positive 10 U\ml (REF <7), anti-Ro/SS-A, and anti-La/SS-B antibodies were undetectable serum complement levels were normal. LFT and renal function tests and urinalysis were within normal limits. Chest X-ray and electrocardiography revealed no abnormality. Ophthalmologic tests were also normal. Systemic examinations were within normal limits. The histopathological examination of skin biopsy specimen showed dermis thinking in the blood vessels wall due to fibrin, mild papillary oedema, perifollicular and interstitial inflammatory cell infiltrate predominantly of lymphocytes and histocytes. On base of the clinicopathologic findings, a diagnosis of LET was made. Treatment was initiated with prednisolone (1 mg/kg. d), combined with hydroxychloroquine (6.5mg/kg/d) MMF and vitamin D supplements were further added to the patient

**Conclusion:** Considering the history of LET being transformed from DLE, we speculate that a continuous spectrum may include DLE, LET, and SLE, and these 3 entities could potentially, convert between each other.

**Patient Consent Received**

Yes

**Disclosure of Interest**

None declared

### P230 Winning the battle after one year of suffering: a case of systemic lupus erythematosus with coexistent psoriasis vulgaris treatment challenge: a first child case report in Libya

#### A. M. A. Abushhaiwia^1^, Y. AElfawires1^2^, R. A. Algariani1^2^

##### ^1^Paediatric Rheumatology, Tripoli Children's Hospital, Faculty of Medicine,University of Tripoli; ^2^Paediatric Rheumatology, Tripoli Children's Hospital, Faculty of Medicine, University of Tripoli, Tripoli, Libya

###### **Correspondence:** A. M. A. Abushhaiwia

**Introduction:**

Systemic lupus erythematosus (SLE) is the prototypic multisystem autoimmune disorder with apparent shift to Th2 immune responses leading to B cell hyperactivity and production of autoantibodies. Psoriasis, on the other hand, is a predominantly Th1 immune response. The coexistence of SLE and PS is considered unusual and very rare. However, treatment is also challenging, as medications used to treat one condition exacerbate or even trigger the symptoms of the other. Worldwide, therefore. We herein report first Libyan female a child with pSLE who develops Ps; illustrate the dilemma of diagnosis and therapeutic management in such case.

**Objectives:** to present the clinical problem of SLE and Ps coexistence

**Methods:** case report presentation

**Results:**

We have a 12-year-old Arab Libyan female child born by parents of non-consanguineous marriage. There was no family history of neither autoimmune nor metabolic diseases. She had history of progressive dyspnoea, periorbital edema, and fatigue over the past few months. Her physical examination showed pericardial rub, short for her age height 113.5cm (<0.4 percentile). Investigations showed marked hypothyroidism with echocardiogram confirming severe pericardial effusion and a tamponade phenomenon. Urgent pericardiocentesis relieved her acute symptoms and prompt treatment with thyroxine replacement .one month later was referred to our Rheumatology clinic with history of malar rash and mild alopecia,diagnosed as SLE at the age of 10 years old according to American college of Rheumatology (ACR) criteria. The diagnosis of SLE was based on the presence of constitutional symptoms, malar rash, photosensitivity, serositis, hepatomegally, ANA was positive, antids-DNAab was positive 144.8 IU\ml, antiSMA was negative, anticardiolipin IgG positive 15.2 normal range <10, anticardiolipin IgM was positive 340 MPL-U\l, lupus anticoagulant was positive, C3 was low 0.3 g\l, C4 was normal 0.1, AST was very high 93.0 IU\L, ALT was very high 185 IU\L,TSH 0.09 uIU\ml very low (normal range 0.27-5) free T4 was high 31.9 pmol\l (normal range 12-22), serology test for autoimmune hepatitis and anti-tissue transglutaminase IgA,IgG were negative,).She had been in prednisone intake 10mg\day, L-thyroxin, added hydroxychloroquine 200mg since March 2019 prior to appearance of lesions and subsequent one-year history of severe skin lesions over her body. Which presented as erythematous, well-defined scaly plaques on the scalp, behind ears, trunk, extremities and buttock. The dermatologists were treated her as infectious eczematous dermatitis however, the malar rash persisted. Psoriatic skin changes became more frequent and worsening. Skin biopsy revealed the presence of psoriasis vulgaris. Other laboratory parameters (CBC, ESR, CRP, urinalysis, chemistry) were normal. ANA was positive 1:160; anti-dsDNA was negative, ENA was negative, APL-antibodies were negative, anti thyroid peroxidise AB was negative 9 IU\ml normal ranges <35. C3 was normal, C4 8 mg/dl (N: 14-44), C1q level: 15.1 (5-25), CH50: 80: (N: 60-130). Since her C4 level is low while C3 level is normal and the absence of lupus nephritis, we considered the possibility of C4 mutation. WES test has not resulted yet. Considering her SLE is inactive and her psoriasis is stable after discounted hydroxycholoquine, immunosuppressant (MMF, azathioprin that used for a while without any benefits) and prednisone was slowly tapered until discontinuation. The goal was to manage her with methotrexate if she will have flare skin lesions

**Conclusion:** We need to consider the potential development of GPP when administering HCQ to patients with SLE even though it is rare; it is a severe adverse event.

**Patient Consent Received**

Yes

**Disclosure of Interest**

None declared

### P231 Rhupus syndrome in children: a case report from Libya

#### A. Abushhaiwia^1^, A. Ateeq^2^, Y. AElfawires^3^, A. Ahlam Nuwayji ^4^

##### ^1^Paediatric Rheumatology, Tripoli Children Hospital,Faculty of Medicine, University of Tripoli; ^2^Paediatric Rheumatology, Tripoli Children Hospital; ^3^Paediatric Rheumatology, Tripoli Children Hospital, Faculty of Medicine, University of Tripoli; ^4^Paediatric Rheumatology, Tripoli Children's Hospital, Tripoli, Libya

###### **Correspondence:** A. Abushhaiwia

**Introduction:** Overlapping JIA and JSLE is a rare clinical condition in children. RHUPUS syndrome has been defined as a Rhupus is an overlap syndrome of rheumatoid arthritis and systemic lupus erythematous (SLE) in adult patients. To diagnosis a patient with Rhupus present with clinical symptoms of SLE with positive ANA, antiDNA or antiSm, associated with clinical symptoms of JIA

**Objectives:** Describe clinical and serological findings of a Libyan male child diagnosed with Rhupus

**Methods:** case report

**Results:** An 8 year-old Libyan black boy was referred with history with clinical manifestation (fever, fatigue, pallor, jaundice, and LL oedema), hepatosplenomegally, ascites and arthritis in the left elbow and left shoulder. At that time, laboratory results revealed anemia (Hb: 9.7 g/dL), thrombocytopenia (30,000/mm3) and normal leukocytes (WBC count 8000/mm3) (predominant lymphopenia) high elevated ESR 100ml\hr, positive CRP . Coomb’s test was negative, elevated liver enzymes AST was 134 U\L, ALTI was 136 U\L, TB 4.2, BD 2.5, total protein 8, S. Albumin was low 3.2, viral screen (HIV, HBSAg, HCV) was negative, serology tests to exclude Wilson disease was negative, serology tests for AIH were negative, CTscan chest, abdomen pelvis revealed hepatomegaly with normal parencyhmal density, moderate ascites with minimal right pleural effusion, bone marrow aspiration was normal, ANA was positive (1/60, anti-dsDNA level was positive 1/80, anti-SM was negative, rheumatoid factor level (RF) was 7.88 IU/mL (0–14IU/mL), and C3 (65.7mg/dL) and C4 (4.4mg/dL) levels were low. Antiphospholipid antibodies (aPL) level was negative and urinalysis was negative. He was diagnosed as JSLE, with more than five criteria,. Treatment was started with new diagnosis of SLE with Prednisolone (1mg/kg/day), Azathioprine (50 mg/d), and Hydroxycholoroquine (200/mg/day). After two years of ongoing chronic polyarthritis MRI both hips revealed a vascular necrosis of both femoral heads with subsequent changes, that needs to doing hip total or subtotal replacement in future. The main symptom lately was erosive arthritis, polyarticular, and morning stiffness and decreased joint range of motion; there was evidence of multiple joints erosion in radiologic evaluation of elbows, shoulders, hips and both knees. After 5-year followup, he is 17 years old and he is under observation and he was on low dose prednisolone (0.5 gr/kg/day), azathioprine (50 mg/d), and hydroxycholoroquine (400/mg/day) and methotraxte. The patients met 6 classification ACR 1997 criteria for SLE. All of the patients met ILAR 2001 criteria for JIA diagnosis. Joint manifestations were treated with methotrexate, the patient required etanercept therapy for persistent joint disease but unfortunately not available in Libya

**Conclusion:** The coexistence of two JIA and JSLE in the same patient is a very uncommon finding. It is very important to recognize the Rhupus in order to make a prompt diagnosis and intervention that assures a better outcome for patients

**Patient Consent Received**

Yes

**Disclosure of Interest**

None declared

### P232 Dysregulated endothelial markers in systemic lupus erythematosus: a systematic review and meta-analysis

#### S. C. Bergkamp^1^, M. Wahadat^2,3^, A. Salah^1^, T. Kuijpers^1^, V. Smith^4,5,6^, S. Tas^7,8^, J. van den Berg^1^, S. Kamphuis^2^, D. Schonenberg - Meinema^1^

##### ^1^Paediatric Immunology, Rheumatology and Infectious Diseases, Emma Children’s Hospital, Amsterdam University Medical Centres (AUMC), University of Amsterdam, Amsterdam; ^2^Paediatric Rheumatology, Sophia Children's Hospital, Erasmus University Medical Center; ^3^Immunology, Erasmus University Medical Center, Rotterdam, Netherlands; ^4^Internal Medicine, Ghent University; ^5^Rheumatology, Ghent University Hospital; ^6^Unit for Molecular Immunology and Inflammation, VIB Inflammation Research Centre (IRC), Ghent, Belgium; ^7^Rheumatology & Clinical Immunology; ^8^Experimental Immunology, Amsterdam University Medical Centres (AUMC), location AMC, Amsterdam, Netherlands

###### **Correspondence:** S. C. Bergkamp

**Introduction:** Systemic Lupus Erythematosus (SLE) patients are at risk for premature atherosclerosis at relatively young age. Endothelial dysregulation is one of the pathophysiologic mechanisms that lead to higher risk for cardiovascular disease in SLE.

**Objectives:** To perform a systematic literature review on endothelial markers that are dysregulated in SLE and investigate potential associations with disease activity.

**Methods:** Search terms were entered into Embase, MEDLINE, Web of Science, Google Scholar and Cochrane. Inclusion criteria were 1) studies published after 2000 reporting measurements of endothelial cell (EC) markers in serum and/or plasma of SLE patients (diagnosed according to ACR/SLICC criteria), 2) English language peer reviewed articles, and 3) disease activity measurement.

**Results:** From 1892 hits, 124 eligible articles were selected. The identified endothelial markers were involved in endothelial cell (EC) activation, endothelial cell apoptosis, disturbed angiogenesis, defective vascular tone control, immune dysregulation and coagulopathy. Meta-analyses of primarily cross-sectional studies showed significant associations between marker levels and disease activity for the following endothelial markers: Pentraxin-3, GAS6, Thrombomodulin, VEGF, ICAM-1 and MCP-1. Other dysregulated markers, without associations with disease activity, were: Angiopoeitin-2, Neopterin, vWF, P-Selectin, VCAM-1, TWEAK, IP-10 and E-Selectin

**Conclusion:** We provide a complete literature overview for dysregulated endothelial markers in SLE comprising a wide range of different endothelial functions. SLE-induced endothelial marker dysregulation was seen with and without association with disease activity. Longitudinal data on endothelial markers in SLE are now needed to guide us in unravelling the pathophysiology of premature atherosclerosis and cardiovascular events in SLE patients in young adulthood.

**Patient Consent Received**

No

**Disclosure of Interest**

None declared

### P233 Pulmonary manifestations in childhood-onset systemic lupus erythematosus: a national multicentric retrospective cohort

#### E. Bernard^1^, A. Hadchouel Duverge^2^, A. Kheniche^3^, A. Faye^1^, F. Hofer^4^, C. Rebelle^5^, M. Caillez^6^, A. Carbasse^7^, Y. Hatchuel^8^, H. Reumaux^9^, I. Lemelle^10^, A. Belot^11^, Y. Uzunhan^12^, B. Bader-Meunier^13^, I. Melki^1^

##### ^1^Pédiatrie Générale, Hôpital Robert Debré; ^2^Pneumologie Pédiatrique, Hôpital Necker Enfants-malades; ^3^Radiologie Pédiatrique, Hôpital Robert Debré, Paris, France; ^4^JIRcohorte, Lausanne, Switzerland; ^5^Pédiatrie, Hôpital Saint Joseph; ^6^Néphrologie pédiatrique, Hôpital de la Timone, Marseille; ^7^Urgence et Post-urgence pédiatriques, Hôpital Arnaud de Villeneuve, Montpellier; ^8^Pédiatrie, Maison de la Femme, de la Mère et de l'Enfant. CHU de Martinique, Fort-de-France; ^9^Rhumatologie Pédiatrique, Hôpital Jeanne de Flandre, Lille; ^10^Hémato-oncologie Pédiatrique, Hôpital d'Enfant - CHRU de Nancy, Nancy; ^11^Néphrologie-Rhumatologie-Dermatologie Pédiatrique, Hôpital Femme-Mère-Enfant, Hospices Civils de Lyon, Lyon; ^12^Pneumologie, Hôpital Avicenne, Bobigny; ^13^Unité d'Immuno-Hématologie et Rhumatologie Pédiatrique, Hôpital Necker Enfants-Malades, Paris, France

###### **Correspondence:** E. Bernard

**Introduction:** While well described in adults, there is little data regarding pulmonary involvement in systemic lupus erythematosus in pediatric population.

**Objectives:** To describe characteristics of lung features in a cohort of childhood-onset systemic lupus erythematosus (cSLE) patients, to evaluate their prevalence and characteristics associated with severe manifestations.

**Methods:** A national retrospective multicenter cohort study was performed between 2007 and 2020, including cSLE patients who presented symptomatic pulmonary features.

**Results:** Forty-six patients, with a median age at diagnosis of 12.7 years, were included from 10 French centers. Lung features prevalence in cSLE, estimated from available data (38 of the 46 included patients, from 5 centers), was 10.1%. Acute manifestations (78%) included: pleuritis (39%), diffuse alveolar hemorrhage (DAH) (22%), acute lupus pneumonitis (22%), infections (20%). Chronic manifestations (33%) associated: interstitial lung disease (17%), pulmonary hypertension (13%) and shrinking lung syndrome (9%). Severe forms, defined as the need for intensive care or third-line therapies, were frequent (n=15/46, 33%) but no death from pulmonary manifestation was observed. Children with severe lung features had a lower female to male ratio (1.1:1 versus 5.2:1, p=0,038), presented more renal manifestations (73% versus 45%, p=0,047) and less serositis (13% versus 52%, p=0,013) in comparison to non-severe patients. DAH was more prominent in the severe group (47% versus 10%, p=0,008), while pleuritis was more frequently reported in non-severe patients (7% versus 42%, p=0,018).

**Conclusion:** With one of the largest cohorts reported, we observed that pulmonary features in cSLE are varied and often severe. These manifestations should actively be assessed, with extensive paraclinical tests such as pulmonary function tests (associated with DLCO), computed tomography or bronchoalveolar lavage, if necessary. Early diagnosis enables adequate, aggressive treatment of severe forms, and long-term follow-up is necessary for those patients.

**Disclosure of Interest**

None declared

### P234 Acquired angioedema in juvenile systemic lupus erythematosus: a case of therapeutic success with the use of rituximab

#### M. Bernardo^1^, I. S. Graça^2^, S. Lopes da Silva^3^, P. Costa-Reis^1^

##### ^1^Pediatric Rheumatology Unit, Santa Maria Hospital, Centro Hospitalar Universitário Lisboa Norte; ^2^Pediatric Cardiology Department, Santa Cruz Hospital, Centro Hospitalar Lisboa Ocidental; ^3^Allergy and Immunology Department, Santa Maria Hospital, Centro Hospitalar Universitário Lisboa Norte, Lisbon, Portugal

###### **Correspondence:** M. Bernardo

**Introduction:** Angioedema is a rare phenomenon in patients with Lupus. It usually results from acquired C1-inhibitor deficiency due to antibody formation directed against this molecule. Some reports also describe patients with severe forms of Systemic Lupus Erythematous, classical pathway–mediated hypocomplementemia and presence of anti-C1q antibodies.

Herein we report a case of Juvenile Systemic Lupus Erythematosus presenting with recurrent angioedema, normal C1q and C1 inhibitor antigen levels and positive anti-C1q antibodies, with an excellent response to rituximab.

**Objectives:** -

**Methods:** -

**Results:** A previously healthy fifteen-year-old girl was referred to a Paediatric Rheumatology clinic due to recurrent episodes of facial and acral angioedema without urticaria or pruritus, accompanied by asthenia and painless cervical lymphadenopathy with 2 months duration. She had no family history of angioedema or rheumatic/autoimmune diseases. Type I and type II hereditary angioedema, lymphoproliferative and infectious causes were excluded. Complementary investigation identified hypocomplementemia (C3 - 41mg/dl, C4 - 6 mg/d and CH50 – 23.8 U/ml), positive antinuclear antibodies (1/160, fine speckled pattern), positive lupus anticoagulant antibodies and anti-C1q antibodies (237 U/mL; normal <15), with normal C1q (23.7 mg/dl) and C1 inhibitor antigen (23.5 mg/dl) and functional levels (99,5%). It was not possible to test for anti-C1 inhibitor antibodies in our institution.

Acquired angioedema, associated with systemic lupus erythematous, was strongly suspected. However, the patient lacked clinical criteria for systemic lupus erythematous thus far. Hydroxychloroquine and oral prednisolone (0.75 mg/kg/day) were initiated with a fairly good response (less frequent and milder episodes).

One year later, daily, severe headaches, without response to painkillers, started. Neuroimaging (MRI) was normal. *Pseudotumor cerebri* was excluded and a mild elevated protein CSF level was found (92 mg/dl), with negative CSF cultural results and negative anti-NMDA, anti-AQ4 and anti-MOG antibodies. Mycophenolate mofetil (600 mg/m^2^/dose) was added to therapy with a very significant clinical response.

A formal diagnosis of systemic lupus erythematous was finally established two years after angioedema presentation, with the addition of the following clinical and immunological manifestations: alopecia, malar erythema, Raynaud phenomenon, cutaneous vasculitis, polyarthritis, lymphopenia, persistent microscopic haematuria, non-nephrotic proteinuria, leukocyturia and positive anti-dsDNA antibodies. Blood pressure and renal function were normal.

Due to increased frequency of angioedema episodes, poor lupus disease activity control (SLEDAI>10) with optimized conventional therapy, and inability to taper glucocorticoids, rituximab was started with excellent results (375mg/m^2^, weekly, four doses). After the first dose, the patient remained asymptomatic with sustained remission of angioedema, allowing safe tapering of glucocorticoids.

**Conclusion:** There are scarce reports of angioedema as a presenting symptom of systemic lupus erythematous.

Our patient had several atypical findings for acquired angioedema, including a normal C1-inhibitor antigen and its functional activity. Patients with classical pathway-mediated complement consumption, presence of autoantibodies against C1q and systemic autoimmune manifestations have been seldom described. The pathophysiology in most of these cases is still poorly understood and diagnosis is usually established on clinical grounds.

Rituximab seems to be a safe and effective treatment in cases of Systemic Lupus Erythematosus with angioedema.

**Patient Consent Received**

Yes

**Disclosure of Interest**

None declared

### P235 Juvenile systemic lupus erytematosus experience of a tertiary hospital in South of Spain

#### A. Capilla Miranda, L. Fernandez-Silveira, M. Camacho-Lovillo, M.-I. G. Ruiz-Santa Quiteria, O. Neth

##### Division of Pediatrics Infectious Diseases, Rheumatology and Immunology, Hospital Universitario Virgen del Rocío, Sevilla, Spain

###### **Correspondence:** M. Camacho-Lovillo

**Introduction:** Juvenile systemic lupus erythematosus (jSLE) is a chronic multisystem autoimmune disease. Around 15 to 20% SLE patients are diagnosed before the age of sixteen. Disease severity and outcome are more serious in childhood.^1^

**Objectives:** To analyze the pattern of disease expression, laboratory data, treatments used and outcome in pediatric patients diagnosed with jSLE, followed in a tertiary care Hospital in Spain.

**Methods:** Medical charts of all the pediatric patients younger than 16 years old, diagnosed with jSLE between January 2008 and December 2020, according to revised 1997 American College of Rheumatology (ACR) criteria^2^ and followed at the pediatric rheumatology clinic in our hospital were retrospectively reviewed.

**Results:** We describe a cohort of 17 patients with a mean age at disease onset of 10.8 ± 2.63 years (mean ± 1SD, range 3-14 years), thirteen were female (76%), follow-up time was a mean of 3,5 ± 2 years (mean ± 1SD, range 0,6-7 years). 24% had first-degree relatives with autoimmune diseases.

Arthralgia was the most prevalent clinical feature with a frequency of 76%, followed by mucocutaneous involvement (malar rash and oral aphthosis) and constitutional manifestations like fever or asthenia.

Ten (59%) developed renal involvement, eight of which showed the latter at the time of diagnosis. Renal biopsy was performed in all of them, showing nephritis class II in 3/10 (2/3 evolved to IV), class III in 3/10, class IV in 3 and class V in 1/10.

Neuropsychiatric manifestations developed in 6 patients. Headache (4 patients) was the most common symptom. One patient presented paresthesia and one suffered from seizures due to cerebral ischemia.

Serositis was found in three (18%) patients, and pulmonary involvement appeared in two, as pulmonary hemorrhage and decreased lung function.

Hematologic abnormalities were found in 76% patients. Anemia (53%) followed by thrombocytopenia (41%) were the most common findings at diagnosis.

Antinuclear antibodies (ANA) were detected in all patients, but anti-dsDNA and anti-ENA were both found in 82%. Hypocomplementemia (low C3 and C4) was detected in 15 patients (88%) at diagnosis.

Some type of antiphospholipid antibody was positive in 7 patients (41%) and aCL IgM was the most antiphospholipid antibody detected at diagnosis. At the follow-up period, one patient developed renal venous thrombosis and other developed cerebral venous thrombosis.

All patients received hydroxychloroquine and oral corticosteroid. Ten (59%) were treated with intravenous pulse corticosteroid, mycophenolate mofetil in 71%, cyclophosphamide in 35%, methotrexate in 41%, rituximab in 24%, tacrolimus in 12% and azathioprine in 6%. 59% received antiplatelet or anticoagulants.

At the end of the study, 59% patients had an SLEDAI score of 0 (complete remission) whilst receiving treatment. 13% (2 patients) had a mild SLEDAI score (≤3), 13% had a moderate SLEDAI (>3 and ≤12) score and 13% had a severe SLEDAI score (>12)^3^. One patient required a renal transplant and two had dialysis requirement. Two patients died, one because of cerebral venous thrombosis and other died of cause nonrelated of jSLE.

**Conclusion:** Our patients were similar to jSLE patients from other series^3,4^.

Although jSLE is uncommon in childhood, disease activity tends to be greater in pediatric patients, increasing morbidity. A multidisciplinary approach including early treatment approaches is necessary to offer the best possible outcome of this patient group.

**Patient Consent Received**

No

**Disclosure of Interest**

None declared

### P236 Time to diagnosis depends on the age at disease onset in a monocentric cohort of 177 patients with juvenile systemic lupus erythematosus

#### A. Petaccia^1^, C. Chighizola^2,3^, I. Pontikaki^3^, G. Rogani^1,2^, F. Corona^1^, M. Gattinara^3^, T. Giani^4^, R. Cimaz^2,3^, on behalf of on behalf of Pediatric Rheumatology Associated Group of the Milan Area (PRAGMA)

##### ^1^Fondazione IRCCS Ca' Granda, Ospedale Maggiore Policlinico; ^2^Department of Clinical Sciences and Community Health, University of Milan; ^3^Pediatric Rheumatology Unit, ASST G Pini, Milan; ^4^University of Siena, Siena, Italy

###### **Correspondence:** C. Chighizola

**Introduction:** Juvenile Systemic Lupus Erythematosus (jSLE) is a rare systemic autoimmune inflammatory disease that usually presents in early adolescence and is characterized by a severe clinical course.

**Objectives:** The aim of this work is to investigate how the clinical features at disease onset impact the time to diagnosis in a monocentric cohort of patients with jSLE.

**Methods:** The clinical records of patients were retrospectively reviewed. Patients were included in the study in case of i) diagnosis of SLE according to 1997 American College of Rheumatology (ACR) or the 2012 SLICC classification criteria for SLE and ii) disease onset before 18 years of age. Clinical data at disease onset were retrospectively collected. Continuous data are expressed as median (interquartile range [IQR]). Potential differences in continuous variables between two or more subgroups were investigated using Mann-Whitney U test or Kruskal-Wallis (KW) test, respectively. Dunn’s correction was applied for multiple comparisons. Statistical analysis was performed using GraphPadPrism v6. P values <0.05 were regarded statistically significant.

**Results:** One hundred seventy-seven patients (155 females, 87.6%) fulfilling the inclusion criteria were recruited in the study. The median age at disease onset was 12.2 years (10-14.5). The diagnosis of jSLE was made at a median age of 12.9 years (10.6-15.2), with a median delay of 2 months (1-8). A significant difference in the time to diagnosis emerged when patients were classified in 3 groups upon the age at diagnosis (before 10 years, between 10 and 15 years, after 15 years of age; KW=7.65, p=0.021). In particular, patients diagnosed after 15 years had a time to diagnosis significantly longer compared to those receiving a diagnosis of jSLE before 10 years (median 1.4 and 4.0 months, respectively; p=0.01). No difference in the time to diagnosis (months) emerged when patients were subgrouped upon the presence of renal, articular, cutaneous, neurological and haematological involvement at disease onset. In particular, patients with anemia (hemoglobin<12g/dL), thrombocytopenia (platelets<100,000/mm^3^), leukopenia (white blood cells<4,000/mm^3^), had a time to diagnosis not statistically different from patients with normal hemoglobin, white blood cell or platelet count, respectively. Time to diagnosis was similar in patients stratified upon anti-dsDNA antibody positivity, increased ESR or CRP levels, low serum C3 or C4.

**Conclusion:** Age at disease onset is the main determinant of the time to diagnosis in patients with jSLE in our large cohort. In particular, subjects presenting the first clinical manifestations when aged more than 15 years were diagnosed significantly later.

**Patient Consent Received**

No

**Disclosure of Interest**

None declared

### P237 Disease manifestations at onset in a monocentric cohort of 177 patients with juvenile systemic lupus erythematosus

#### C. Chighizola^1,2^, A. Petaccia^3^, I. Pontikaki^2^, M. Gattinara^2^, T. Giani^4^, F. Chironi^3^, M. Torcoletti^3^, R. Cimaz^1,2^, on behalf of on behalf of the Pediatric Rheumatology Associated Group of the Milan Area (PRAGMA), Milan, Italy

##### ^1^Department of Clinical Sciences and Community Health, University of Milan; ^2^Pediatric Rheumatology Unit, ASST G Pini; ^3^Fondazione IRCCS Ca' Granda, Ospedale Maggiore Policlinico, Milan; ^4^University of Siena, Siena, Italy

###### **Correspondence:** C. Chighizola

**Introduction:** Systemic Lupus Erythematosus (SLE) is a systemic autoimmune inflammatory disease that in 10-20% of cases can present in pediatric age, usually in early adolescence. Juvenile SLE (jSLE) is characterized by a severe clinical course.

**Objectives:** The aim of this work is to describe the clinical manifestations and the laboratory features at disease onset in a monocentric cohort of patients with jSLE.

**Methods:** The clinical records of patients were retrospectively reviewed. Patients were included in the study in case of i) diagnosis of SLE according to the 1997 American College of Rheumatology (ACR) or the 2012 SLICC classification criteria for SLE and ii) disease onset before 18 years of age. Demographic data, details about clinical manifestations and laboratory features at disease onset were collected. Continuous data are expressed as median (interquartile range [IQR]) and categorical data as percentages. The association between categorical variables was assessed by chi-squared test or Fisher exact test, as appropriate. Statistical analysis was performed using GraphPadPrism v6. P values <0.05 were regarded as statistically significant.

**Results:** One hundred seventy-seven patients (155 females, 87.6%) fulfilling the inclusion criteria were recruited in the study. The median age at disease onset was 12.2 years (10-14.5). The diagnosis of jSLE was made at a median age of 12.9 years (10.6-15.2), with a median delay of 2 months (1-8). Hematologic involvement was the most common disease manifestation (133 patients, 75%), followed by joint and cutaneous involvements (124 patients, 70% and 101 patients, 57%, respectively). Renal disease was observed in 58 patients (32.8%) while neurological complications were diagnosed in 26 cases (14.7%). Table 1 reports the immunological profile and laboratory data of enrolled patients. The only difference in disease manifestations between genders related to the higher prevalence of anemia observed in males (χ^2^: 10.4; p=0.0012). Patients with positive anti-dsDNA antibodies had a higher rate of renal involvement (χ^2^: 21.4; p<0.0001), neurological manifestations (χ^2^: 6.58; p=0.010), skin lesions (χ^2^: 54.1; p<0.0001), joint involvement (χ^2^:7.64; p=0.0057), leukopenia (χ^2^: 14.8; p=0.0001), low C3 (χ^2^: 13.08; p=0.0003) and low C4 (χ^2^: 42.95; p<0.0001). Patients with positive anti-Sm antibodies had a higher rate of leukopenia (χ^2^:4.96; p=0.0259). Complement levels were inversely related with leukopenia (χ^2^: 6.9; p=0.0086) and joint involvement (χ^2^:5.42; p=0.02).

**Conclusion:** These data on manifestations at onset of jSLE in a large monocentric cohort allow togain further insights into a rare disease with a still high morbidity burden.

**Patient Consent Received**

No

**Disclosure of Interest**

None declared

**Table 1 (abstract P237).** Immunological profile and laboratory data of enrolled patients

Test	Number of patients (%)
Positive ANA > 1:80 in IFI	172 (97.2%)
Positive anti-dsDNA antibodies	133 (75.1%)
Positive anti-Sm antibodies	16 (9%)
Lupus anticoagulant	29 (16.4%)
Leukopenia (<4,000 leukocytes/mm^3^)	62 (35%)
Thrombocytopenia (<100,000 platelets/mm^3^)	35 (19.8%)
Anemia (Hb < 12 g/dL)	115 (65%)
Raised ESR	132 (74.6%)
Raised CRP	21 (11.9%)

### P238 Different patterns of organ involvement at disease onset in a monocentric cohort of 177 patients with juvenile systemic lupus erythematosus

#### C. Chighizola^1,2^, A. Petaccia^3^, I. Pontikaki^2^, S. Costi^1^, F. Baldo^3^, T. Giani^4^, C. Agostoni^1,3^, R. Cimaz^1,2^, on behalf of on behalf of Pediatric Rheumatology Units of the Pediatric Rheumatology Associated Group of the Milan Area (PRAGMA), Milan, Italy

##### ^1^Department of Clinical Sciences and Community Health, University of Milan; ^2^Pediatric Rheumatology Unit, ASST G Pini; ^3^Fondazione IRCCS Ca' Granda, Ospedale Maggiore Policlinico, Milan; ^4^University of Siena, Siena, Italy

###### **Correspondence:** C. Chighizola

**Introduction:** Juvenile Systemic Lupus Erythematosus (jSLE) is a rare systemic autoimmune inflammatory disease that usually presents in early adolescence. jSLE is characterized by a severe clinical course and by a higher prevalence of lupus nephritis, haematological anomalies, neurological and cutaneous involvements.

**Objectives:** The aim of this work is to investigate the different patterns of organ involvement in a monocentric cohort of patients with jSLE.

**Methods:** The clinical records of patients were retrospectively reviewed. Patients were included in the study in case of i) diagnosis of SLE according to 1997 American College of Rheumatology (ACR) or the 2012 classification criteria for SLE and ii) disease onset before 18 years of age. Clinical data at disease onset were retrospectively collected.

**Results:** One hundred seventy-seven patients (155 females, 87.6%) fulfilling the inclusion criteria were recruited in the study. The median age at disease onset was 12.2 years (10-14.5), with only one child being diagnosed before the age of 5 years. Anti-nuclear antibodies tested negative in 5 subjects (2.8%). Complement serum levels were decreased in 138 patients (78%, C3 in 128 patients and C4 in 110). In 128 patients (74.6%), ESR was raised, in 21 cases with a concomitant CRP increase. Patients presented most commonly with 3 disease manifestations (58, 32.8%), while 2 organ involvements were identified in 54 patients (30.5%) and 4 in 25 subjects (14.1%). As presented in Table 1, the most frequent pattern of disease manifestation consisted in the combination of hematological, cutaneous and articular involvements (29 patients, 30.5%). In 26 cases (14.7%), the disease started with the association of hematological abnormalities and joint involvement. Nineteen patients (10.7%) had at disease onset 4 lupus manifestations consisting in hematological, skin, neurological and renal involvements. Only few patients (6, 3.4%) had a SLE onset with 5 manifestations (hematological, cutaneous, neurological, articular and renal involvements).

**Conclusion:** The combination of hematological and joint involvements, alone or in association with other manifestations, provides the most frequent pattern of disease presentation in our large monocentric cohort of jSLE, being identified in more than half of cases (94 patients, 53%).

**Patient Consent Received**

No

**Disclosure of Interest**

None declared

**Table 1 (abstract P238).** Most frequent disease patterns in our monocentric cohort of jSLE

Disease pattern	Number of patients (%)
Hematological + articular + skin involvements	29 (75%)
Hematological + articular involvements	26 (70%)
Hematological + articular + skin + renal involvements	19 (57%)
Hematological + skin involvements	17 (32.8%)
Articular involvement	11 (14.7%)
Hematological involvement	10 (75.1%)
Hematological + articular + renal involvements	8 (35%)
Hematological + skin + renal involvements	7 (19.8%)
Hematological + articular + skin + renal + neurological involvements	6 (65%)

### P239 Pediatric automated neuropsychological assessment metrics as a screening tool for neuropsychiatric syndromes in Turkish children with systemic lupus erythematosus

#### S. Demir^1^, M. Kasap Cüceoğlu^1^, E. S. Akbaş Aliyev^2^, P. Avar Aydın^3^, E. Aliyev^1^, T. Cak^2^, S. Ozen^1^, E. Çengel Kültür^2^, Y. Bilginer^1^

##### ^1^Pediatric Rheumatology; ^2^Child and Adolescent Psychiatry, Hacettepe University; ^3^Pediatric Rheumatology, Ankara University, Ankara, Turkey

###### **Correspondence:** S. Demir

**Introduction:** Childhood-onset systemic lupus erythematosus (cSLE) is a multisystem autoimmune disease characterized by a wide range of clinical manifestations. Neuropsychiatric systemic lupus erythematosus (NPSLE) is one of the challenging involvement due to the lack of specific diagnostic tests. The Pediatric Automated Neuropsychological Assessment Metrics (PedANAM) is a computerized battery that measures cognitive ability, mental processing speed, and memory.

**Objectives:** We compared the PedANAM cognitive performance scores of cSLE patients and controls to determine whether we can detect neurocognitive dysfunction (NCD) in Turkish cSLE patients via PEDANAM.

**Methods:** We performed Ped-ANAM and formal neuropsychological testing on 23 children with SLE and on 12 healthy children. All participants were assessed by a child and adolescent psychiatrist with Schedule for Affective Disorders and Schizophrenia for School-Age Children Present and Lifetime Version, DSM-5 November 2016 -Turkish Adaptation (K-SADS-PL-DSM-5-T). Participants with any current psychiatric diagnosis on the K-SADS-PL-DSM-5-T and intellectual deficits were excluded clinically. PedANAM is a computerized battery which is consisted of 10 subtests and 4 performance parameters. Cognitive Performance Scores (CPS) were adjusted for age and sex. Higher CPS_PCA_ levels indicate a lower probability of NCD and higher CPS_multiscore_ kevels indicate a higher probability of NCD. If a lupus patient’s CPS_PCA_ < 0.25 and CPSmultiscore > 4.62 during follow-up, a formal neurocognitive test and neuroimaging are suggested. The software can be obtained from Vista Life Sciences (Parker CO, USA).

**Results:** Twenty-three patients with cSLE and 12 healthy children were recruited into the study. There was no statistical difference between 2 groups in the mean of age and gender (p>0.05). When we compared the median (min_max) of PedANAM-CPS indices of the two groups; SLE patients had lower CPS_PCA_ than control group [0.73 (-12.86 - 2.69) vs. -0.16 (-2.31 - 2.10), p = 0.824] and CPS_multiscore_ was higher in cSLE than healthy children [5.43 (1.77 – 17.30) vs. 4.66 (2.14 – 6.66), p = 0.363]. However, the differences did not reach to statistical significance. In the SLE group the abnormal results were most frequently seen in learning (62.5%), spatial working memory (50%), attention/precessing speed (43.8%), spatial processing/matching grids (43.8%), delayed memory (47.7%). In the control group, abnormal results were most commonly seen inattention/processing speed (57.1%), and learning (71.4%).

**Conclusion:** Our preliminary results show that PEDANAM could detect scores indicating cognitive impairment in Turkish cSLE patients, who had no clinically evaluated neuropsychiatric disorders.

**Patient Consent Received**

Yes

**Disclosure of Interest**

None declared

### P240 Clinical significance of anti-C1Q antibodies positivity in non-renal juvenile systemic lupus erythematosus

#### G. Dobson^1,2,3^, C. Papadopoulou^1^, A. Al Zaabi^1^, C. Valentine^4^, Z. A. Siwji^1^, C. A. Pilkington^1^, M. Al Obaidi^1^, E. Moraitis^1^

##### ^1^Rheumatology, Great Ormond Street Hospital; ^2^Infection, Immunity and Inflammation Programme Research and Teaching Department, UCL Great Ormond Street Institute of Child Health; ^3^Centre for Adolescent Rheumatology Versus Arthritis at UCL UCLH and GOSH, University College London; ^4^Medical School, Imperial College, London, United Kingdom

###### **Correspondence:** G. Dobson

**Introduction:** In systemic lupus erythematosus, the presence of antibodies against complement C1q (anti-C1q) in combination with anti-dsDNA and low complement has a strong serological association with lupus nephritis [1,2,3]. There is paucity of data on the clinical significance of anti-C1q in juvenile systemic lupus erythematosus (jSLE), particularly with non-renal lupus.

**Objectives:** This retrospective study aimed to identify clinical and laboratory associations of anti-C1q in a large single centre UK based paediatric cohort with jSLE and determine the value of anti-C1q as a marker in patients without lupus nephritis.

**Methods:** Patients seen in the connective tissue disease (CTD) clinic at Great Ormond Street Hospital from May 2014 to July 2019 were included. The following characteristics were collected: sex, age, ethnicity, organ system involvement at presentation and follow-up, presence of anti-C1q, anti-dsDNA antibodies, anti-nuclear antibodies (ANA), antibodies to extractable nuclear antigens (ENA) and its subtypes, antiphospholipid antibodies, complement C3 and C4 levels, immunoglobulins levels, urine albumin/creatinine ratio. Therapies used were also recorded. Measurement of anti C1q is part of the routine assessment at presentation and follow-up for all patients with a possible or established diagnosis of CTD in our centre.

Data were analysed using Statistica. Differences between groups were tested using Mann–Whitney U-test for non-parametric variables and Chi-square and Fischer Exact for parametric variables. P-values less than 0.05 were considered statistically significant.

**Results:** 128 patients were included (20% male, 80% female), the median age at diagnosis was 10.0 years (range 0.9-15.9 years). 73% met the 1997 American College of Rheumatology criteria for jSLE diagnosis, and 27% had other CTD diagnoses.

46% of patients tested positive for anti-C1q and 54% tested negative. Anti-C1q positive patients were more likely to have a diagnosis of jSLE compared to negative patients (75% and 39%, respectively; P=0.01).

Nephritis, anti-dsDNA positivity, low C3, low C4 were more common in anti-C1q positive compared to negative patients: 42% compared to 12%, P=0.0001; 67% compared to 32%, P=0.0002; 62% compared to 15%, P=<0.0001; 65% compared to 34%, P=0.001, respectively.

Positive anti-C1q patients were more likely than negative patients to have arthritis, (71% compared to 44%, P=0.002), oral or nasal ulcers (47% compared to 29%, P= 0.03) and they were less likely to have neurological disorder (18% compared to 42%, P=0.004).

85% of the 46 cases with jSLE had positive anti-C1q and polyarthritis at presentation; of this subgroup 46% had no nephritis at any time during follow-up. From that group of patients, 22% had anti-C1q positivity, arthritis and no renal involvement, despite negative anti-dsDNA antibodies at presentation.

Other differences between groups of patients did not meet the significance threshold once remaining clinical and laboratory features were considered.

**Conclusion:** Previous paediatric studies investigated the association of anti-C1q with lupus nephritis and SLE disease activity markers and we supported this association. In addition, our results highlight an association between anti-C1q positivity and arthritis and mouth ulcers in jSLE. Interestingly, the anti-C1q antedated the anti-dsDNA positivity for a minority of patients with arthritis.

Larger prospective studies are needed to confirm our observations. Nevertheless, these data support screening for these antibodies in paediatric patients presenting predominantly with arthritis.

**Disclosure of Interest**

None declared

### P241 Caregiver burden and related factors in caregivers of patients with childhood-onset systemic lupus erythematosus

#### G. Durcan^1^, S. Uzuner^2^, S. Sahin^3^, K. Bahali^4^, K. Barut^3^, A. G. Kilicoglu^5^, A. Adrovic^3^, A. Bilgic^6^, O. Kasapcopur^3^

##### ^1^Department of Child and Adolescent Psychiatry, University of Health Sciences, Bakirkoy Research and Training Hospital for Psychiatry, Neurology and Neurosurgery; ^2^Department of Pediatrics, Medical Faculty, Bezmialem Vakif University; ^3^Department of Pediatric Rheumatology, Medical Faculty of Cerrahpasa, Istanbul University-Cerrahpasa, Istanbul; ^4^Department of Psychology, Istanbul Gelisim University; ^5^Department of Child and Adolescent Psychiatry, Medical Faculty, Bezmialem Vakif University, Istanbul; ^6^Department of Child and Adolescent Psychiatry, Medical Faculty of Meram, Necmettin Erbakan University, Konya, Turkey

###### **Correspondence:** S. Sahin

**Introduction:** Having a child with a chronic disease is stressful for all members of the family, and especially for the primary caregiver (in most cases, the mother). Caregiver burden is used to identify physical, psychological, social or financial problems that may occur while providing care to another person with a physical or mental disability.

**Objectives:** There is no documentation about the association between caregiver burden and psychological status in caregivers of patients with childhood-onset systemic lupus erythematosus (SLE). The aim of this study was to evaluate the associations between caregiver burden and both the caregiver’s and child's psychological symptoms in a cohort of children with SLE.

**Methods:** Thirty-four patients (aged 9-18 years) with childhood-onset SLE and their caregivers participated in this study. The control group (n=34) was composed of healthy children and their caregivers. The Zarit Burden Interview (ZBI), Beck Depression Inventory (BDI), and Beck Anxiety Inventory (BAI) were used to assess caregiver burden and the caregiver's psychological state. The Strengths and Difficulties Questionnaire (SDQ)-parent form was completed by the caregiver to assess the psychological state of children and adolescents.

**Results:** The mean age of the children and caregivers in the patient group was 14.35 ± 2.83 and 39.91 ± 5.19 years, respectively, and it was 15.62 ± 1.46 and 42.44 ± 7.12 years in the control group. There was no difference between the groups in terms of the ages of the children and caregivers. No significant difference was found between the study and control groups for caregiver burden, anxiety and depression in parents, and psychological status in children. Caregiver burden was positively correlated with parent's depression (r = 0.648, p < 0.001), anxiety (r = 0.396, p = 0.03), and behavioral (r = 0.383, p = 0.025) and peer problems (r = 0.394, p < 0.05) of the children, and it was negatively correlated with the children’s prosocial behaviors (r = -0.473, p = 0.005). According to regression analyses, the parents' depression and children's peer relationship had a positive effect on caregiver burden scores.

**Conclusion:** Physicians should be aware of the presence of psychological symptoms in patients with childhood-onset SLE and their caregivers because it can affect caregiver burden and the caregiver's psychological state. The psychological status of children with SLE and their caregivers should be evaluated periodically during the diagnosis and follow-up process, and at-risk caregivers and their children should be referred to mental health professionals.

**Patient Consent Received**

Yes

**Disclosure of Interest**

None declared

### P242 A Patient engagement project- improving communication of the concept of 'treat-to-target' in JSLE with young people

#### R. Elliott^1^, E. Taylor^1^, E. Smith^2,3^

##### ^1^School of Medicine, University of Liverpool; ^2^Department of paediatric rheumatology, Alder Hey Children's NHS Foundation Trust; ^3^Institute of Life Course and Medical Sciences, University of Liverpool, Liverpool, United Kingdom

###### **Correspondence:** R. Elliott

**Introduction:** Treat to target (T2T), has become part of routine clinical care in many areas of medicine, and proposed as a strategy to improve management of juvenile-onset systemic lupus erythematosus (JSLE). The TARGET LUPUS research programme: ‘Targeting disease, Agreeing Recommendations and reducing Glucocorticoids through Effective Treatment, in LUPUS’ has been established in order to develop a JSLE T2T study. Children and young people (CYP) participating in TARGET LUPUS related studies have expressed difficulty in understanding the concept of T2T, causing concerns for informed consent and participant recruitment within a future T2T clinical trial.

**Objectives:** To explore, in-depth, the views of CYP on a proposed animation and voice-over, which will help to explain the concept of T2T to eligible JSLE patients in a future T2T clinical trial.

**Methods:** An illustrated animation story board was developed on PowerPoint, to be used alongside a contemporaneous voiceover which allowed the animation to be simulated for CYP participating in three established young person’s research advisory groups (Generation R, Lupus UK young person’s group, and YOUR RHEUM). Topic-guided discussion was used to generate feedback on both the storyboard and voiceover. Meetings were recorded, transcribed and analysed for key areas of improvement. Changes were made iteratively to the resources, based on participant feedback. Participants completed pre/post workshop questionnaires to assess the impact of the resources on their understanding of T2T. Descriptive statistics (median values, interquartile ranges, IQR) reported. Paired t-test compared pre/post workshop questionnaire results.

**Results:** 40 CYP were consulted. 16/40 (40%) from Generation R (median age 15 years [IQR 12-15]), 12/40(30%) from Lupus UK (median age 27 [IQR 22-30]), and 12/40(30%) from YOUR RHEUM (median age 17 [IQR 16-21]). 62% of respondents had an underlying rheumatic condition. 39/40(98%) and 37/40(93%) CYP completed the pre/post-workshop surveys respectively. In the pre-workshop survey, the median participant understanding of T2T was 2 [IQR 1-4], on a 1-10 scale. After viewing the resources, participant understanding improved to a median of 9 [IQR 8-10], p<0.0001). The majority of CYP (76%) felt that the concept of T2T is best communicated using an animation and accompanying participant information sheet. 19% felt the animation could be used alone, and 5% stated they would prefer a written description alone.

Participants liked that the animation followed the journey of a patient participating in a T2T study. They liked the use of a graph which showed progress towards the target. CYP made the following suggestions for improvement; (1) a slide showing a clearer side by side comparison T2T and standard care, using key words and pictures to demonstrate the differences, (2) involving different characters in the voiceover (patient, doctor, narrator), (3) inclusion of a parent in the animation rather than a patient alone, (4) emphasising that it isn’t the patients fault if they haven’t met their target, (5) changes to terminology.

**Conclusion:** Involvement of CYP in research is crucial to help improve the design and delivery of studies, ensuring that it is relevant to CYP/families. In the current study, involvement of CYP has greatly improved the animation explaining the concept of T2T, leading to important changes to the visuals, terminology and content, and significant improvements pre/post-test survey scores. These resources will be of benefit in a future JSLE T2T study, supporting study recruitment.

**Trial registration identifying number:**

**Disclosure of Interest**

None declared

### P243 Primary systematic lupus erythematosus combined with wilson’s disease in a male child: a rare case report

#### D. Hadef^1^, S. Slimani^2^, F. Lahouel^3^, B. Benlahcen^4^, N. Bouchair^5^

##### ^1^Department of Pediatrics, University hospital center of Batna, Faculty of Medicine, Batna 2 University; ^2^Atlas Clinic of Rheumatology; ^3^Department of Nephrology, University hospital center of Batna, Faculty of Medicine, Batna 2 University; ^4^Private Practitioner, Batna; ^5^Faculty of Medicine, Badji Mokhtar Annaba University, Annaba, Algeria

###### **Correspondence:** D. Hadef

**Introduction:** Wilson’s disease (WD) is a rare, recessively inherited disorder of copper metabolism with its accumulation in multiple organs particularly in the liver and brain. Systematic lupus erythematosus (SLE) is an autoimmune disease, and like WD, it involves multiple organs and systems. The combination of WD and SLE is not usual apart from iatrogenism.

**Objectives:** we present a pediatric case of concurrent WD and primary SLE not induced by penicillamine. After extensive research in the literature, this is the only male case described so far. The other seven reported cases are female.

**Methods:** Case presentation:

A previously well 12-year-old boy was admitted at the University Hospital Center of Batna (Algeria) with acute haemolysis (pallor, subictereus, and red urine). There was no consanguinity, or family history. Physical examination revealed normal development and growth, no fever, and no signs of swelling in the liver, lymph nodes and spleen. A complete blood count (CBC) revealed normochromic normocytic anemia, Thrombocytopenia, and Leucopenia. His blood biochemistry showed Hepatic cytolysis, and Hepato-cellular insufficiency. WD was suspected because of the combination of the impaired liver function, hemolytic anemia, and normal alkaline phosphatase levels. Serum ceruloplasmin and copper levels were decreased, while urinary copper was elevated confirming the diagnosis of WD. There was no neurological or ophthalmological involvement. Family investigation revealed WD with cirrhosis in a 9-year-old brother.

The onset of nephrotic syndrome and the presence of inflammatory syndrome cannot be explained by WD. The kidney biopsy histopathology revealed nephritis lupus class II (WHO classification). Subsequent serum analysis also revealed positive native anti-DNA and anti-PCNA antibodies verified on a second sample. Based on all the findings, the final diagnosis for this patient was Wd combined with SLE. We started therapy with bolus of corticosteroids and Cyclophosphamide, relayed by Mycophenolate Moftil and hydroxychloroquine Cooper chelation has also been initiated.

Improvement in renal and even hepatic damage was noted. Unfortunately, after two years, the patient presented abnormal movements with dysarthria. Brain MRI showed abnormal signals of the basal ganglia consistent with neurological damage in WD.

**Results:** Discussion :

Concomitant SLE and WD without penicillamine treatment is rare (7 cases reported in the literature with 3 children). To our knowledge, this is the first report of an association between WD and SLE in a male. In our patient, SLE and WD were diagnosed simultaneously as 4 other described cases.

WD was first suspected due to unexplained impaired liver function with hemolytic anemia. Copper Tests confirming the diagnosis. At that time, there was no neurological or ophthalmological impairment.

In our patient, the worsening of the hematological involvement (pancytopenia) in an inflammatory context, with installation of a nephrotic syndrome cannot be explained by WD. SLE was evoked despite the fact that it was a child and male. Renal biopsy as well as the immunological workup were in favor of SLE disease.

Treatment of SLE improved symptoms but later chelation could not prevent the usual neurologic complication of WD at this age. Neurological involvement appeared at the age of 14 as described in the literature with the common sign of dysathria, followed by the installation of abnormal movements due to the impairment of the basal ganglia seen on MRI.

**Conclusion:** WD and SLE not induced by penicillamine can co-exist. As there is no pathophysiological explanation, it’s probably a simple fortuitous association.

**Disclosure of Interest**

None declared

### P244 A scoping review of treat to target clinical trial study designs - to inform development of a juvenile-onset lupus (JSLE) treat-to-target study

#### E. Ingram^1^, E. M. Smith^2,3^

##### ^1^School of Medicine; ^2^Institute of Life Course and Medical Sciences, University of Liverpool; ^3^Department of Paediatric Rheumatology, Alder Hey Children's NHS Foundation Trust, Liverpool, United Kingdom

###### **Correspondence:** E. Ingram

**Introduction:** Treat to target (T2T) has been proposed as a way to improve management of juvenile-onset systemic lupus erythematosus (JSLE). The TARGET LUPUS^Ó^ research programme: ‘Targeting disease, Agreeing Recommendations and reducing Glucocorticoids through Effective Treatment, in LUPUS’ has been established to develop a JSLE T2T study. The majority of evidence for T2T is in the context of Rheumatoid Arthritis (RA), with recent studies on Psoriatic Arthritis (PsA) and Inflammatory Bowel Disease (IBD).

**Objectives:** This scoping review aims to evaluate methods used in T2T studies carried out in RA, IBD and PsA, considering how these methods could be applied to JSLE.

**Methods:** The search strategy included treat to target, Rheumatoid Arthritis, Psoriatic Arthritis, Ulcerative Colitis and Crohn’s Disease (including variations/abbreviations). Inclusion criteria included; randomised controlled trial, clinical trial, clinical study, clinical trial protocol, controlled clinical trial, pragmatic clinical trial, human species. Papers not on treat-to-target, non-English, reviews, case reports, case series, observational studies, non-human, not on RA, PsA or IBD were excluded. Titles and abstracts screened by two reviewers. Data extracted from full-text papers using a piloted proforma (recording study design, target, target assessor, follow-up frequency, treatment escalation, primary/secondary outcome measures, primary outcome met).

**Results:** 2789 papers were identified via Pubmed and 9 through grey literature. 964 duplicates removed. 1834 titles/abstracts screened for eligibility. 30 studies met the inclusion criteria. 27/30 (90%) were RA, 2/30 (7%) IBD, and 1/30 (3%) PsA related. 6/30 (20%) compared T2T to usual care, 10/30 (33%) compared two T2T regimes with same target, 5/30 (17%) compared different targets. 3/30 (10%) were single arm studies, 3/30 (10%) T2T implementation/sustainability studies and 3/30 (10%) were T2T protocols.

15/30 (50%) studies use the Disease Activity Score (DAS) as a target, 3/30 (10%) ACR/EULAR remission, 1/30 (3%) imaging targets, 2/30 (7%) endoscopic mucosal healing. In 8/30 (27%) studies, targets were DAS-based with another clinical/imaging target. In 1/30 (3%) study the target was physician adherence. In 25/30 (83%) studies the assessor was a rheumatologist, in 3/30 (10%) a nurse, and in 2/30 (7%) an endoscopist. The most common follow-up times were monthly or 3-monthly, however some studies changed follow-up frequency over time.

18/30 (60%) studies met their primary outcome. DAS measures were most commonly used (13/30, 43%), followed by ACR/EULAR remission (4/30, 13%), imaging scores (3/30, 10%), and psychosocial measures (2/30, 7%). Other primary outcomes such as adherence were used in 4/30 (13%). A co-primary outcome (DAS & image-score) was used in 4/30 (13%). Secondary outcomes included damage scores, function scores, imaging and HRQOL scores.

**Conclusion:** T2T is used as part of routine care in RA with many studies supporting its use. Early studies tended to compare T2T to usual care, whereas recent studies compared different aspects of T2T to further refine the approach. This scoping review has provided important insights to help design a future JSLE T2T study.

**Disclosure of Interest**

None declared

### P245 Critical illness and outcome of children with sle admitted in PICU in a tertiary care centre in South India

#### M. Janarthanan^1^, I. Thayammal^2^, S. T.K.^2^, R. P.S.^2^, S. S^2^

##### ^1^Rheumatology; ^2^Paediatrics, Sri Ramachandra Institute of Higher Education & Research, Chennai, India

###### **Correspondence:** M. Janarthanan

**Introduction:** SLE is an autoimmune disease resulting in damage to any organ due to immune dysregulation.the disease course is unpredictable and the outcome of critical illness in adults requiring ICU admission is well established while the data of paediatric counterpart are lacking. Hence we decided to do this study in our hospital.

**Objectives:** To analyse the causes of critical illness in children with lupus and to study the outcome of PICU admissions in children with SLE

**Methods:** This is a retrospective observational study of SLE children requiring PICU stay, over a time period from January 2010 to August 2109. Demographic, clinical, investigatory and management data were collected from casesheets and studied.

**Results:** Of total 108 patients with SLE, 14 patients required 16 episodes of PICU admission. The range of duration of PICU stay was 3-29days. The median age requiring PICU admission was 13. There was female preponderance with F:M ratio of 11:3. Analysis of the data revealed, lupus nephritis with complications were present in 43.7%, while sepsis affected 25%. Macrophage Activation syndrome and severe hemolytic anemia were seen in 18.75% and 12.5% children respectively.

Respiratory manifestations noted in these children include ARDS, pulmonary edema, pulmonary thromboembolism, pulmonary haemorrhage and H1N1 pneumonia. CNS vasculitis and cerebral haemorrhage leading to hemiparesis were the neurological manifestations. Cardiovascular complications included refractory hypertension, cardiac tamponade with obstructive, hypotension shock and severe pulmonary hypertension. Out of 14 children, 4 succumbed to the complications. 1 child died due to late presentation with pulmonary haemorrhage, 1 child with macrophage activation syndrome, 1 child with cardiac complications and 1 due to infection.

**Conclusion:** Infection and disease progression are common causes of morbidity and mortality in children with lupus. Management of complications and infections can be challenging in these children. Early referral to tertiary care and prompt identification and treatment of complications may improve outcome in these children.

**Trial registration identifying number:**

**Disclosure of Interest**

None declared

### P246 Lupus nephritis in children- experience from a tertiary hospital in South India

#### B. Gunabooshanam^1^, S. Geminiganesan^2^, I. Thayammal ^2^, M. Janarthanan^3^

##### ^1^Pathology; ^2^Paediatrics; ^3^Rheumatology, Sri Ramachandra Institute of Higher Education & Research, Chennai, India

###### **Correspondence:** M. Janarthanan

**Introduction:** Childhood SLE is a chronic autoimmune disease with wide spectrum of disease manifestations. Compared to adults, majority of children may present with nephritis at the time of diagnosis or subsequently develop renal involvement over the course of their illness.

**Objectives:** To study the demographic, clinical, laboratory and serological data of biopsy proven lupus nephritis cases and correlate with corresponding histopathologic data. To study the outcome of patients with nephritis at 6 months

**Methods:** A retrospective, cross sectional study was conducted in tertiary care hospital in Southern India . 38 biopsy proven lupus nephritis cases of children between 1m-18yrs of age, diagnosed between January 2010 –December 2020 were included. Demographic data like age, sex, clinical data like symptoms, laboratory data including CBC, urine routine analysis, ANA, dsDNA, 24hr urine protein, urine protein creatinine ratio, BUN, Creatinine, S. Albumin etc were collected. Renal biopsies were examined, immunofluorescence microscopy done and results compared with corresponding laboratory and clinical data.

**Results:** 38 renal biopsies were performed over a period of 10 years in children suspected to have lupus nephritis. The male to female ratio was 2:7.5. Nephritis was present at the time of diagnosis in 8 (21%) and the remaining patients developed nephritis subsequently during follow up. Hypocomplementemia was present in all children with a mean C3 level of 47.19 (mg/dl) and mean C4 level of 8.36(mg/dl). ANA and dsDNA were positive in all children. Based on ISN-RPS classification 7.9% had class I, 23.7% had class II and III each, and 44.7% had class IV nephritis. 26/38 (68.4%) patients had lupus nephritis with activity and the remaining 12( 68.4%) had lupus nephritis without activity. Patients were treated with intravenous steroids followed by oral steroids, hydroxychloroquine, calcium supplements, and either cyclophosphamide or mycophenolate mofetil. At 6 months follow up 4/38 had persistent proteinuria and 3/38 continued to have elevated creatinine. One patient succumbed to illness due to cardiac and renal complications.

**Conclusion:** Lupus nephritis is one of the major determinants of long term outcome in children with SLE. Histopathology and immunofluorescence are helpful in assessing the extent of renal disease and helps in making treatment decisions.

**Disclosure of Interest**

None declared

### P247 Coexistence of systemic lupus erythematosus and rasmussen’s encephalitis in a 8 year-old child

#### Z. Kizildag^1^, R. Isguder^1^, U. Yis^2^, B. Makay^1^, S. A. Hiz Kurul^2^, E. S. Unsal^1^

##### ^1^Pediatric Rheumatology; ^2^Pediatric Neurology, Dokuz Eylul University Faculty of Medicine, Izmir, Turkey

###### **Correspondence:** Z. Kizildag

**Introduction:** Rasmussen’s encephalitis (RE) is an immune-mediated disease characterized by resistant focal epileptic seizures, progressive hemiplegia, cognitive impairment, and unilateral inflammation of the cerebral cortex. Both RE and systemic lupus erythematosus (SLE) have common autoimmune markers suggesting the existence of common immune mechanisms in the pathogenesis.

**Objectives:** To describe the coexistence of RE and SLE.

**Methods:** Case report

**Results:** An eight-year-old girl was admitted to the hospital with a right-sided focal motor seizure and right-sided weakness. Electroencephalography (EEG) showed rare sharp wave discharges in the left frontocentral region. Brain magnetic resonance imaging (MRI) and computerized tomography were normal. The patient was treated with levetiracetam. However, focal seizures and unilateral deficits persisted for two months. A repeat MRI revealed a signal increase in the left frontal lobe, the cortical-subcortical area of the insula and the putamen, and volume loss in the left frontal lobe on the FLAIR sequences. With MRI and the EEG findings of slowing bioelectric activity in the left hemisphere, a diagnosis of RE was made. Despite treatment with levetiracetam, oxcarbazepine and topiramate, seizures persisted. At the pediatric intensive care unit, 20 mg/kg pulse methylprednisolone for three consecutive days and 2 g/kg intravenous immunoglobulin (IVIG) were administered. Since the patient’s mother had SLE and her aunt had lupus nephritis, the patient was searched for other autoimmune disorders that may accompany RE. ANA, which was tested before IVIG infusion, was positive.

A detailed history concerning SLE revealed constitutional complaints such as hair loss, fatigue, anorexia, and cognitive changes such as excessive aggression and introversion. She also had intermittent arthritis of hands with morning stiffness in the last year. ANA was positive at a titer of 1/1000 - 1/3200 with a speckled pattern, anti-RNP/ Sm, and direct coombs were positive. The patient was diagnosed as SLE.

The treatment was aimed to cover both disorders. Methylprednisolone 2 mg/kg and monthly IVIG infusions for RE were continued, and cyclophosphamide (500 mg/m2/month for six courses) with hydroxychloroquine was added. Cyclophosphamide induction therapy was switched to azathioprine. No new organ or system involvement developed, and the frequency of seizures decreased.

**Conclusion:** This case presents the coexistence of common immune mechanisms in the pathogenesis of SLE and RE.

**Patient Consent Received**

No

**Disclosure of Interest**

None declared

### P248 Childhood systemic lupus erythematosus: an update on clinical profile and complications in children from tertiary care centre in sub-himalayan region of North West India

#### H. Kothari, S. Verma, A. Sharma, S. Guleria, R. Jaswal, A. Rafi, M. Sharma, S. Sharma, P. Gautam, J. Sharma, N. Chaudhary

##### Paediatrics, Dr RPGMC Medical College Tanda at Kangra (HP), Kangra (HP), India

###### **Correspondence:** H. Kothari

**Introduction:** SLE is a chronic autoimmune disease characterized by multisystem inflammation and abnormal immunological function. Almost all published research suggests a higher frequency of renal, neurological, and haematological involvement with paediatric SLE than with adult SLE at the time of diagnosis.

**Objectives:** The study was done to find the clinical profile and complications in a cohort of children with SLE admitted in a tertiary care centre in a resource limited setting in Sub-Himalayan region of North west India.

**Methods:** We did a retrospective observational study of all children diagnosed as SLE from July 2017-December 2020 in a tertiary care hospital in north India. Demographic data, clinical profile, laboratory investigations and follow up details of the patients were collected and analysed from Paediatric Rheumatology Register of our centre. Diagnosis of SLE was based on Systemic Lupus International Collaborating Clinics (SLICC) classification criteria.

**Results:** A total of 17 children (female: male- 7.5:1) with SLE were analysed. Median age of symptom onset was 13 years (range 8-17 years). The initial manifestations at the time of presentation were fever(59%), joint involvement(59%), malar rash(47%), renal involvement(35%), photosensitivity(30%), alopecia(19%), headache(19%), body ache(19%) Raynaud’s phenomenon(12%) and gastrointestinal complaints(12%). Atypical presentation included isolated peripheral arthritis(6%) and B/L pleural effusion(6%). Lupus nephritis was present in 6 (35%) children. Renal function tests were deranged in only 1 case. Out of 6, 66.67% (4) patients with lupus nephritis were diagnosed to have hypertension. Urine examination revealed proteinuria and microscopic haematuria in 6 patients. Renal biopsy was done in 5 patients, 2 had class III and other three had class II, class IV and class V lupus nephritis respectively. ANA, Anti dsDNA positivity and hypocomplementemia were present in 94%, 45% and 75% of patients respectively. One patient had positive Anti Smith,U1RNP,SSA,RO 52,SSB antibodies. AVN of hip joint was detected in 2 of our SLE patients (12.5%).Both the patients required core decompression of femoral head. One patient developed osteonecrosis B/L hip joints on prolonged steroid therapy 7 years after diagnosis of disease whereas the other patient developed symptoms 1 year after diagnosis and steroid intake. In our study, there was an association between intravenous pulse methylprednisolone and AVN. It highlights the value of screening lupus patients by MRI to rule out the presence of AVN.

All patients were started on hydroxychloroquine and photoprotection. Children with renal involvement were given pulse methylprednisolone followed by tapering doses of oral prednisolone and intravenous, monthly cyclophosphamide. Azathioprine or Mycophenolate Mofetil were used as maintenance therapy in all. Subcutaneous weekly methotrexate was used in 2 patients. Two children died during the disease course. Cause of death was Macrophage Activation Syndrome (MAS). The case fatality rate was 12% in our centre. Disease continues to be in remission in rest.

**Conclusion:** The current study reveals complications and few atypical presentations in children with SLE. There is a significant female preponderance in our study group. Joint involvement followed by renal was the commonest presentation. We believe that the aetiology of AVN in SLE is multifactorial. Corticosteroid is the main predisposing factor for AVN in SLE patients and its use should be judicious. Early recognition of SLE is critical for timely initiation of appropriate treatment.

**Disclosure of Interest**

None declared

### P249 Prevalence and clinical association of anti C1Q antibodies in pediatric SLE

#### S. Kumar, G. Ipe

##### Pediatric Rheumatology, Department of Pediatrics, Department of Pediatrics, Christian Medical College, Vellore, India

###### **Correspondence:** S. Kumar

**Introduction:** Anti-C1q has been associated with systemic lupus erythematosus (SLE) as well as in other connective tissue diseases. They have been considered as a marker for disease activity and presence of nephritis in previous studies

**Objectives:** Aim of this study was to determine the prevalence of anti- C1q antibodies in the pediatric SLE population and to determine clinical associations of elevated anti- C1q antibody levels especially with lupus nephritis

**Methods:** Sera of 150 pediatric SLE patients who fulfilled ACR criteria for SLE were recruited. After obtaining informed consent, blood samples were tested for anti- C1q antibody by commercially available ELISA kit. Prevalence of anti-C1q and its association with lupus nephritis were determined

**Results:** Out of total 150 children with SLE, anti- C1q positivity was present in 95 children (64%), at a cut off value of 20U/ml. Children with proteinuria, low C3, low C4 and anti dsDNA positivity had were significantly more likely to have anti- C1q antibody positivity. Children with lupus nephritis were significantly more likely to have anti C1q antibodies positive than children without renal involvement (74% vs. 51%, p= 0.02). Among the children with lupus nephritis, children with active renal disease were more likely to have anti- C1q positivity than in children with quiescent disease (88% vs. 53%, p= 0.002). Anti-C1q antibodies had a sensitivity of 74% and specificity of 54% at a cut off value of 22U/L, for renal disease in pSLE

**Conclusion:** Out study confirms previous findings of the association of anti-C1q antibodies with nephritis and disease activity in pSLE. Anti-C1q antibody titers were found to have positive correlation with renal disease in children with pediatric SLE, and could be used as an adjunctive biomarker in monitoring disease activity in children with lupus nephritis

**Disclosure of Interest**

None declared

### P250 Neuropsychiatric systemic lupus erythematosous: monocentric study and novel biomarkers

#### M. Labouret^1^, V. Trebossen^2^, A. Ntorkou^3^, S. Bartoli^1^, V. Bondet^4^, D. Duffy^4^, M. Elmaleh^3^, L. Seabra^5^, B. Bader-Meunier ^6^, M.-L. Frémond^5^, I. Hau^7^, V. Hentgen^8^, E. Le Roux^9^, U. Meinzer^1^, C. Parmentier^10^, J.-F. Benoist^11^, Y. Crow^5^, P. Ellul^2^, I. Melki^1,5,6^

##### ^1^General Paediatrics, Infectious Disease and Internal Medicine; ^2^Child and Adolescent Psychiatry; ^3^Paediatric Radiology Department, Robert Debré Hospital; ^4^Translational Immunology Lab, Pasteur Institute; ^5^Laboratory of Neurogenetics and Neuroinflammation, Imagine Institute; ^6^Paediatric Hematology-Immunology and Rheumatology, Necker-Enfants Malades Hospital, Paris; ^7^General Paediatrics, Centre Hospitalier Intercommunal de Créteil (CHIC), Créteil; ^8^General Paediatrics, Versailles Hospital, Versailles; ^9^Clinical Research Unit, Robert Debré Hospital; ^10^Paediatric Nephrology, Trousseau Hospital; ^11^Reference Centre for Inherited Metabolic Diseases, Necker-Enfants Malades Hospital, Paris, France

###### **Correspondence:** M. Labouret

**Introduction:** Juvenile systemic lupus erythematosus (j-SLE) is a rare pediatric chronic auto-immune disease affecting multiple organs. Neuropsychiatric SLE (NPSLE) is frequent in (j)SLE and is associated with increased mortality compared to the general population and to SLE patients without neuropsychiatric involvement. Furthermore, NPSLE morbidity and impact on quality of life is considerable. Although NPSLE classification criteria and activity scores have been developed, attributing neuropsychiatric features to SLE remains a major challenge, as no specific reliable NPSLE biomarkers have been validated.

**Objectives:** Describe thoroughly j-NPSLE features and try to identify specific markers.

**Methods:** Retrospective (January 2017 to December 2020) monocentric study including any patient followed for j-SLE (onset <16 years) meeting the 2019 European League Against Rheumatism (EULAR) American College of Rheumatology (ACR) Classification Criteria for SLE. Neuropsychiatric events were attributed to SLE using ACR NPSLE criteria (isolated headaches excluded) after multidisciplinary evaluation by a psychologist, a child psychiatrist, and a paediatric rheumatologist.

**Results:** Among 39 patients included, 44% (n=17) presented with j-NPSLE. J-NPSLE was diagnosed at the onset of j-SLE in 59% and was most often associated with active lupus disease. Patients with j-NPSLE had frequent kidney involvement (76%). All had psychiatric symptoms: hallucinations (71%), psychomotor retardation (71%), cognitive symptoms (59%), depressed mood (35%), catatonia with agitation (12%). Headache (53%) and hyperreflexia (41%) were often associated. The main signs noted on brain MRI were T2/FLAIR white matter hypersignals (71%), often considered as non-specific, and cerebral atrophy (88%), sometimes following corticotherapy. Neopterin and interferon-alpha (IFN-α, quantified by Simoa) in CSF were higher at j-NPSLE diagnosis than during partial or complete remission of j-NPSLE (respectively p=0.01 and p=0.001). During the acute phase steroid shots followed by oral corticotherapy were initiated in 15 patients (88%), cyclophosphamide in 9 patients (53%) while 13 patients (76%) were treated by mycophenolate mofetil in the maintenance phase. Under immunosuppressive treatment, clinical improvement of neuropsychiatric symptoms was observed in all patients.

**Conclusion:** Clinicians should be aware of the unique presentation of j-NPSLE (predominantly psychiatric manifestations). CSF IFN-α (Simoa) and neopterin levels may be new significant biomarkers. Nevertheless, prospective investigations are warranted to assess the specific link between these biomarkers and active NPSLE.

**Patient Consent Received**

No

**Disclosure of Interest**

None declared

### P251 Success with rituximab in 2 patients with unusual forms of presentation in pediatric systemic lupus erythematosus

#### F. O. Liñan^1^, J. E. Leiva^2^, M. N. Miranda^2^, C. E. Leiva^2^, M. K. Miñano^2^, K. L. Varas^2^, J. C. Rodriguez^2^, D. A. Corro^2^, C. R. Otoya^2^

##### ^1^Reumatología, Hospital Víctor Soles, Viru; ^2^Reumatología, Hospital Victor Lazarte, Trujillo, Peru

###### **Correspondence:** F. O. Liñan

**Introduction:** Pediatric SLE (pSLE) represents 15% of all patients with SLE. Renal and neuropsychiatric involvement is more aggressive in pSLE, with single-organ involvement being the most common clinical form of appearance. Forms of presentation such as cerebral infarction and serositis are uncommon presenting manifestations in pSLE. Treatment of pSLE does not differ from that of adults and the therapeutic arsenal is the same as in adult forms. Rituximab (RTX) is a biological agent used worldwide in SLE with excellent results, however, there is still no consensus on its real efficacy in SLE.

**Objectives:** Presentation of 2 cases of pSLE with infrequent forms of presentation (cerebral infarction and serositis), which did not respond to conventional therapy but did respond to rituximab.

**Methods:** Case report, with a description of the clinical picture, diagnostic method and form of treatment.

**Results:** First case: a 16-year-old woman presented with a progressive headache with a tonic-clonic seizure. The brain scan shows a left frontoparietal cerebral infarction. On physical examination, he found livedo reticularis in the lower limbs, joint pain, hair loss, and oral ulcers. Tests reveal normocytic anemia, thrombocytopenia, decreased complement, 1/320 ANA with homogeneous pattern, and 3,200 mg of protein in a 24-hour urine sample. Diagnosis of pLES is made and it is pulsed with methylprednisolone 1 g EV daily for 3 days, then cyclophosphamide 1 g EV monthly. After 3 months, proteinuria, fatigue and arthralgia persist. For this reason, it was decided to use rituximab at a dose of 375 mg / m2 on days 1 and 15 every 6 months. After 4 infusions, the proteinuria disappeared, as well as the arthralgias and general malaise. She currently maintains SLEDAI-2K in remission score, maintaining low doses of prednisone.

Second case: a 10-year-old boy, diffuse abdominal pain with associated distension begins more or less abruptly. Tiredness, shortness of breath, and palpitations are added. A plain abdominal X-ray does not show air-fluid levels, but the chest plate shows bilateral pleural effusion with enlargement of the cardiac silhouette. An abdominal echocardiogram and ultrasonography show pericardial effusion and ascites respectively. Physical examination reveals general paleness, translucent lower limb edema, pericardial rub, and decreased vesicular murmur in both lung bases. Laboratory tests show leukopenia, lymphopenia, normocytic anemia, elevated acute phase reactants, ANA 1/560, anti-DNA 280 U / mL, decreased complement, elevated urea, creatinine, and transaminases. SLE is diagnosed and it is pulsed with methylprednisolone 30 mg / kg / dose for 4 days, to then go on to mycophenolate 600 mg / m2 daily. Initially there is improvement, but after 2 months initial serositis reappears. It was decided to use rituximab 375 mg / m2. After the second infusion, serositis improved, but maintained elevated creatinine levels. He is currently in low activity, with lupus nephritis and slightly elevated creatinine values.

**Conclusion:** Cerebral infarction and serositis are rare forms of debut in pSLE. the cases presented showed elevated ANA titers and decreased complement. Both cases improved with rituximab after failure of cyclophosphamide and mycophenolate. Informed consent was obtained from the parents and the patients. Disclosures: none declared.

**Patient Consent Received**

Yes

**Disclosure of Interest**

None declared

### P252 Diagnostic delay in juvenile-onset systemic lupus erythematosus: insights from a British paediatric surveillance unit study

#### H. Lythgoe^1^, E. M. Smith^1,2^, O. G. Killeen^3^, R. Murphy^4^, C. Pilkington^5^, C. Pain^1^, M. Beresford^6,7^, on behalf of in association with the British Paediatric Surveillance Unit

##### ^1^Department of paediatric rheumatology, Alder Hey Children’s NHS Foundation Trust; ^2^Institute of Life Course and Medical Sciences, University of Liverpool, Liverpool, United Kingdom; ^3^Department of paediatric rheumatology, Our Lady’s Children’s Hospital, Crumlin, Ireland; ^4^Department of dermatology, Sheffield Teaching Hospitals NHS Foundation Trust, Sheffield; ^5^Department of paediatric rheumatology, Great Ormond Street Children’s Hospital, London; ^6^Institute of Life Course and Medical Sciences; ^7^Department of paediatric rheumatology, Alder Hey Children's NHS Foundation Trust, Liverpool, United Kingdom

###### **Correspondence:** H. Lythgoe

**Introduction:** Juvenile-onset systemic lupus erythematosus (JSLE) is a rare, multi-system autoimmune disease. Its heterogenic nature means that diagnosis can be challenging and delay is recognised(1).

**Objectives:** This study aimed to examine time from symptom onset to diagnosis in children and young people (CYP) with a new diagnosis of JSLE in the UK and Ireland and consider potential impacting factors.

**Methods:** British Paediatric Surveillance Unit (BPSU) methodology was used to identify new diagnoses of JSLE (09/2017-09/2019) in children <18 years of age, meeting either American College of Rheumatology (ACR-1997) or Systemic Lupus International Collaborating Clinics (SLICC-2012) classification criteria in the UK and Ireland. Time to diagnosis was quantified in months from symptom onset to diagnosis. Exploratory factors which may impact time to diagnosis including age at onset, gender, ethnicity, overnight admission at diagnosis, ACR and SLICC classification data were included in a Cox regression survival analysis. Variables with hazard ratios (exp(β)s) >1 are associated with higher probability of being diagnosed with JSLE earlier. Factors which reached significance (p<0.05) in the univariate model were combined into a multivariate model.

**Results:** There were 112 patients included in this study with a median time from symptom onset to diagnosis of 4 months (interquartile range 2–10 months). Time from symptom onset to diagnosis was <1 month for 18/112 (16%), between 1-12 months for 70/112 (63%) and over 12 months for 24/112 (21%). Patients were diagnosed by rheumatology (74/112, 66%), nephrology (8/112, 7%), general paediatrics (17/112, 15%), dermatology (4/112, 4%) or by a multi-speciality team (all involving rheumatology or nephrology) (9/112, 8%). Most patients (82/112, 73%) were seen by at least one other hospital team prior to diagnosis and 25/112 (22%) were seen by two or more other hospital teams prior to diagnosis. The diagnosing clinicians felt that patients had not been referred appropriately, despite seeking medical review, in 15/112 (13%).

Table 1 shows the five individual factors reaching statistical significance as potential predictors of shorter time to diagnosis. When combined within a multivariate model, the only factor that retained significance was overnight admission at diagnosis. Interestingly, length of stay did not reach statistical significance, perhaps implying that the importance of admission is not solely related to the patient being more unwell at diagnosis.

**Conclusion:** There remains a marked delay in achieving diagnosis for CYP presenting with JSLE. In the UK and Ireland, overnight admission at diagnosis appears to be the strongest predictor of shorter time to diagnosis. This may relate to the patient being more unwell (and therefore warranting admission) or by accessing a more expedient diagnostic work-up through elective admission to hospital. Future studies should focus on understanding whether novel care pathways, including routine admission for patients with possible JSLE to facilitate diagnostic work-up could lead to earlier diagnosis and treatment.

**Patient Consent Received**

No

**Disclosure of Interest**

None declared

**Table 1 (abstract P252).** Results of univariate Cox regression analysis

Factor associated with delay	Exp(β)	P-value	Confidence interval
Overnight admission at diagnosis	1.708	0.006	1.17 – 2.50
Anti-dsDNA positive	1.708	0.027	1.06 – 2.74
ACR immunological criterion met	2.593	0.028	1.11 – 6.07
ACR score	1.147	0.033	1.01 – 1.30
SLICC score	1.119	0.005	1.03 – 1.21

### P253 Is there a difference in clinical picture of systemic lupus erythematosus in male and female juvenile patients?

#### V. Matkava, I. Nikishina, M. Kaleda, Z. Kolkhidova, T. Pachkoria

##### Pediatric Department, V. A. Nasonova Research Institute of Rheumatology, Moscow, Russian Federation

###### **Correspondence:** V. Matkava

**Introduction:** Systemic lupus erythematosus (SLE) is usually more common in women. There is evidence of sex differences in the clinical picture and course of SLE. It seems that sex dimorphism may affect to clinical features of SLE in children and adolescents.

**Objectives:** To clarify whether there are differences in the clinical features and disease course between male and female patients with juvenile onset of SLE (jSLE).

**Methods:** The retrospective study included 138 pts with SLE, who were admitted to our center for the last 10 years (2011-2020). Diagnosis of SLE based on 2012 SLICC criteria. Age at onset, family history, clinical, hematological and immunological manifestations of SLE, the therapy were evaluated. SLEDAI 2К was used for disease activity assessment. The data obtained were compared depending on the sex.

**Results:** In described study group the male pts was 15% (boys/girls ratio = 1:5). The median age at the onset of jSLE was 10.0 y.o. [interquartile range (IQR) 8.2; 12.5] in boys, 13.3 y.o. [10.25; 15.0] – in girls. 42.8% of boys and 20.5% of girls got sick before the age of 10 (p = 0.047). Family history of various immuno-inflammatory diseases had 24 girls (20.5%), 7 boys (33.3%) (p = 0.25). There was no difference of the median disease duration at the time of jSLE verification between male and female pts. The boys more often had acute cutaneous lupus at the onset (95% boys and 68% girls, respectively) (p = 0.0084). We did not find a statistically significant difference between other manifestations of jSLE depending on sex, but boys were relatively more likely to have non-scarring alopecia (33% and 16%, respectively), involvement of nervous system (38% and 20%) and leukopenia (81% and 60%). Assessment of the immunological features had no statistically significant differences depending on sex, but anti-Smith antibodies (24.4% and 14.3%) and hypocomplementemia (53.3% and 33.3%) were more presented among girls. Evaluation of disease activity at onset did not reveal sex differences (12 [8; 17] in girls and 14 [8; 19] in boys). Therapeutic options were comparable depending on sex. The use of biologics (B) (rituximab/belimumab/abatacept), including sequential therapy of 2 B, was equally required (14.2% of boys, 13.9% of girls).

**Conclusion:** According to our results, boys had an earlier onset of SLE, especially before puberty. Acute cutaneous lupus was observed more often in boys. There was also a tendency without statistically significant differences for boys in relation to alopecia, leukopenia, CNS involvement, but for girls in relation to immunological features (higher level of anti-Smith antibodies and hypocomplementemia). There is no established preferred choice of therapy regimen in male and female pts.

**Patient Consent Received**

Yes

**Disclosure of Interest**

None declared

### P254 Juvenile systemic lupus erythematous: about a series of 35 cases

#### H. Nassih, R. Elqadiry, A. Bourrahouat, I. Ait Sab

##### Pediatrics, Mohammed 6 University Hospital Center of Marrakesh, Marrakesh, Morocco

###### **Correspondence:** H. Nassih

**Introduction:** Systemic Lupus Erythematous (SLE) is a chronic autoimmune disease that can involve any organ system with a wide range of disease manifestations, and can lead to significant morbidity and even mortality.

**Objectives:** This series describes the epidemiology, common clinical features, complications of disease, and the management of SLE in children.

**Methods:** A single center retrospective descriptive study of 35 children with SLE.

**Results:** There was 27 (77%) girls and 8 (22.9%) boys. The mean age at diagnosis was of 9±2 years, with extremes of 3 and 15-year-old. Systemic involvement was in the form of protracted fever in 62.8%, nephrotic syndrome in 37%, nephritic syndrome in 40%, thrombotic microangiopathy in 8.6%, isolated proteinuria in 25.7%, acute kidney injury in 25.7%, malar rash in 48.6%, arthritis in 57%, neurolupus in 17%, serositis in 11.4%, and hemolytic anemia in 28.6%. Autoimmune conditions associated with SLE were systemic sclerosis, antiphospholipid antibodies syndrome and Gujerat-Sjogren syndrome in one case each. Two cases had severe pancreatitis as a revealing pattern. Posterior uveitis was present in one case. Meanwhile, pulmonary involvement was seen in two cases (interstitial lung disease and recurrent pneumonia). Proliferative lupus nephritis was found in kidney biopsy in 48.6%. Four cases had an end stage kidney disease and were put under hemodialysis. Management consisted on hydroxychloroquine at 5 mg/kg/day and oral steroids at a starting dose of 2 mg/kg/day in all cases, while pulses of methylprednisolone were prescribed in 57% of our cases. Other immunomodulatory treatments used were IV cyclophosphamide in 34.4%, mycophenolate mofetil in 51.4%, azathioprine in 8.6%, tacrolimus in 5.7%, rituximab in 11.4%, and methotrexate in 2.8%. Evolution was marked by stabilization of the disease activity in 42.8% and recurrent flares in 28.6%. Meanwhile, six cases were lost to follow-up, and four cases died. The main reason of death was the disease activity (seen in two cases), followed by infection and macrophages activation syndrome (in one case each).

**Conclusion:** Management of juvenile SLE can be challenging, especially when there is severe systemic involvement. Disease damage, drugs side effects and frequent hospitalization have major effect on the quality of life of pediatric patients.

**Disclosure of Interest**

None declared

### P255 Neuropsychiatric involvement in childhood systemic lupus erythematosus: a multicenter study in Turkey

#### A. Paç Kısaarslan^1^, S. Özdemir Çiçek^1^, E. D. Batu^2^, S. Şahin^3^, M. K. Gürgöze^4^, S. Balcı Çetinkaya^5^, M. Kışla Ekinci^5^, B. Atmış^6^, K. Barut^3^, A. Androvic^3^, B. Esen Ağar^7^, N. Şahin^1^, F. Demir^8^, E. Bağlan^9^, M. Akbalık Kara^10^, S. Zırhlı Selçuk^11^, S. Özdel^9^, E. Çomak^12^, G. Otar Yener^13^, D. Gezgin Yıldırım^14^, K. Öztürk^15^, M. Yıldız^3^, F. Haşlak^3^, S. Şener^2^, H. Kısaoğlu^16^, Ö. Baba^16^, Z. Kızıldağ^17^, R. İşgüder^17^, S. Çağlayan^8^, R. B. Güven Bilgin^18^, G. Aytaç^18^, B. Bozkaya Yücel^19^, A. Tanatar^20^, H. E. Sönmez^21^, M. Çakan^22^, A. Kara^7^, A. T. Elmas^11^, B. Demircioğlu Kılıç^10^, N. Aktay Ayaz^20^, B. Kasap^23^, B. Çelikel Acar^24^, O. Ozkaya^25^, S. Yüksel^26^, S. Bakkaloğlu^27^, Ö. Aydoğ^19^, G. Aksu^18^, S. Akman^12^, M. Bülbül^9^, M. Büyükçelik^10^, Y. Tabel^11^, B. Sözeri^8^, M. Kalyoncu^16^, Y. Bilginer^2^, M. H. Poyrazoğlu^1^, E. Ünsal^17^, Ö. Kasapçopur^3^, S. Ozen^2^, R. Düşünsel^1^

##### ^1^Pediatric Rheumatology, Erciyes University Faculty of Medicine, Kayseri; ^2^Pediatric Rheumatology, Hacettepe University Faculty of Medicine, Ankara; ^3^Pediatric Rheumatology, Istanbul University, Cerrahpaşa Faculty of Medicine, Istanbul; ^4^Pediatric Nephrology and Rheumatology, Fırat University Faculty of Medicine, Elazığ; ^5^Pediatric Rheumatology; ^6^Pediatric Nephrology, Çukurova University Faculty of Medicine, Adana; ^7^Pediatric Nephrology, Fırat University Faculty of Medicine, Elazığ; ^8^Pediatric Rheumatology, Ümraniye Research and Training Hospital, Istanbul; ^9^Pediatric Rheumatology, Dr. Sami Ulus Maternity and Child Health and Diseases Research and Training Hospital, Ankara; ^10^Pediatric Nephrology, Gaziantep University Faculty of Medicine, Gaziantep; ^11^Pediatric Nephrology, İnönü University Faculty of Medicine, Malatya; ^12^Pediatric Nephrology, Akdeniz University Faculty of Medicine, Antalya; ^13^Pediatric Rheumatology, Şanlıurfa Research and Training Hospital, Şanlıurfa; ^14^Pediatric Rheumatology, Diyarbakır Training and Research Hospital, Diyarbakır; ^15^Pediatric Rheumatology, Istanbul Medeniyet University, Göztepe Prof. Dr. Süleyman Yalçın City Hospital, Istanbul; ^16^Pediatric Rheumatology, Karadeniz Technical University Faculty of Medicine, Trabzon; ^17^Pediatric Rheumatology, Dokuz Eylül University Faculty of Medicine; ^18^Pediatric Rheumatology, Ege University Faculty of Medicine, Izmir; ^19^Pediatric Rheumatology, Ondokuz Mayis University Faculty of Medicine, Samsun; ^20^Pediatric Rheumatology, Istanbul University Faculty of Medicine, Istanbul; ^21^Pediatric Rheumatology, Kocaeli University Faculty of Medicine, Kocaeli; ^22^Pediatric Rheumatology, University of Health Sciences, Zeynep Kamil Women and Children’s Diseases Training and Research Hospital, Istanbul; ^23^Pediatric Nephrology, Katip Çelebi University, Izmir; ^24^Pediatric Nephrology and Rheumatology, Ankara City Hospital, Ankara; ^25^Pediatric Nephrology and Rheumatology, İstinye University Faculty of Medicine, Istanbul; ^26^Pediatric Nephrology and Rheumatology, Pamukkale University Faculty of Medicine, Denizli; ^27^Pediatric Nephrology and Rheumatology, Gazi University Faculty of Medicine, Ankara, Turkey

###### **Correspondence:** A. Paç Kısaarslan

**Introduction:** The childhood neuropsychiatric SLE(c-NPSLE) is a challenge for clinicians. NPSLE is more frequent while there are fewer studies in children than adults.

**Objectives:** To evaluate c-NPSLE in Turkey.

**Methods:** Demographic features, laboratory, imaging findings, and the features according to the ACR 2019 case definitions were evaluated.

**Results:** 143/1059 c-NPSLE patients (F/M: 8/1) were involved from 23 centers. The c-NPSLE prevelance was 13.6%. The median (min-max) age at SLE diagnosis and NP involvement were 159(15-215) and 168(15-248) months,respectively. The median (min-max) SLEDAI score was 20 (2-61) at NP involvement. The most frequent CNS involvements were headache(48.3%),seizure(39.2%),acute confusional state(33.6%),mood disorder(30.8%), anxiety disorder(30.1%),cognitive dysfunction(21.7%),movement disorder(18.2%), psychosis(18.2%),cerebrovascular disease(14.7%). The most frequent peripheral nervous system involvements were polyneuropathy(6.3%),chronic inflammatory demyelinating polyneuropathy(3.5%),Guillain-Barre syndrome(2.8%),isolated optic neuritis(2.8%). The other manifestations were PRES(9.1%), idiopathic intracranial hypertension(5.6%), neuromyelitis optica(2.8%). The most frequently administered therapies were oral corticosteroids(97.9%) and hydroxychloroquine(89.5%) while cyclophosphamide was the most frequently used immunosuppressant(55.2%). Non-NPSLE involvements were hematologic (79.7%) and skin (77.6%) involvements, lupus nephritis(47.6%), serositis(68.5%). Permanent neurological sequelae was present in 11.9% (n=17),7 (4.9%) patients died.

**Conclusion:** This study provides real-life data for c-NPSLE with a high number of patients from Turkey, and shows that c-NPSLE can have very different phenotypic patterns and may cause significant morbidity. We believe that it will significantly contribute to the literature on c-NPSLE, for which little information is available.

**Disclosure of Interest**

None declared

### P256 Case report: a catatonic teenager

#### L. Paterson-Brown^1^, A. Jones^1^, N. Scally^2^, D. Imeson^3^, V. Shivamurthy^4^

##### ^1^Paediatric Rheumatology, Evelina London Children's Hospital; ^2^Paediatrics, Croydon University Hospital; ^3^Liason Child and Adolescent Psychiatry, Evelina London Children's Hospital; ^4^Paediatric Rheumatology, Evelina London's Children Hospital, London, United Kingdom

###### **Correspondence:** L. Paterson-Brown

**Introduction:** Juvenile Systemic Lupus Erythematosus (JSLE) presents with a wide range of symptoms affecting many organs.

**Objectives:** We present a 14 year old girl with acute confusion and psychosis who was diagnosed with Neuropsychiatric JSLE.

**Methods:** A case report.

**Results:** Our patient was referred with 48-hours of behavioural change: confusion, agitation and aggression. There were no physical symptoms or history of infection and her physical examination was normal. Baseline investigations were unremarkable and the following morning her symptoms had largely recovered. The Child and Adolescent Mental Health Service (CAMHS) team’s initial diagnosis was acute stress reaction. It was not until she presented with catatonia 2 months later and previous investigations reviewed, that they noted an unexplained raised ESR so further autoimmune bloods were sent, including Anti-dsDNA which was positive. The patient required inpatient psychiatric treatment and then was safe for transfer to a medical paediatric hospital to begin JSLE treatment.

During her follow up, the patient continued to have psychiatric symptoms however bloods normalised with an unremarkable repeat MRI brain. As a result, it is difficult to establish how best to monitor response to treatment in this patient.

Recent studies state that catatonia should be considered a red flag for autoimmune conditions(1) and may also be useful as a marker of severity(2). As a result we continue to monitor closely. The most helpful clinical information has been from reported changes in behaviour by her mother and psychiatric reassessment. There are also side effects of psychiatric medications to consider and at times it has been difficult to differentiate behavioural changes secondary to medication changes or increasing inflammation. Ongoing care requires close work alongside psychiatry to monitor symptoms and her response to any medication changes with regular mental state examinations.

**Conclusion:** This is a rare presentation with primary psychiatric symptoms, posing many challenges to the team throughout. It is not uncommon for JSLE patients to have neurological involvement but this is not common at presentation(3) and is usually accompanied by signs in other organ systems. It is rare to have pure psychiatric symptoms, therefore in these patients we must look carefully for associated physical symptoms or signs for evidence of wider systemic inflammation. We must also look further into the required follow up for patients where imaging and serology do not correlate with our clinical suspicions of ongoing inflammation. How can we best measure ongoing inflammation and inform the next treatment decisions? It is our responsibility as a paediatric rheumatology community to share knowledge with our colleagues in other specialties, learn from each other’s experiences and build strong cross team working relationships so that we support each other with the problem solving approach required for difficult or rare presentations.

**References**

1. Ferrafiat V, Riquin E, Freri E, Granata T, Nardocci N, Medjkane F, et al. Psychiatric autoimmune conditions in children and adolescents: Is catatonia a severity marker? Progress in Neuro-Psychopharmacology and Biological Psychiatry. 2021 Jan 10;104:110028.

2. Cohen D, Ferrafiat V, Raffin M, Consoli A. 2.6 CATATONIA AND AUTOIMMUNE CONDITIONS IN CHILDREN AND ADOLESCENTS: A DIAGNOSTIC AND THERAPEUTIC CHALLENGE. Journal of the American Academy of Child & Adolescent Psychiatry. 2019 Oct 1;58(10, Supplement):S132.

3. Olfat MO, Al-Mayouf SM, Muzaffer MA. Pattern of neuropsychiatric manifestations and outcome in juvenile systemic lupus erythematosus. ;23(5):395-9. doi: 10.1007/s10067-004-0898-3. Epub 2004 Jul 23. PMID: 15278752. Clin Rheumatol.

**Patient Consent Received**

Yes

**Disclosure of Interest**

None declared

### P257 Childhood systemic lupus erythematosus (CSLE) damage measured using systemic lupus international collaborating clinics/american college of rheumatology damage index (SDI) in a cohort of patients from Mumbai, India

#### V. Shah^1^, P. Pimpale Chavan^2,3^, R. Khubchandani^1^

##### ^1^Section of Pediatric Rheumatology; ^2^Former Fellow Pediatric Rheumatology, Jaslok Hospital & Research Centre, Mumbai, India; ^3^Current : Inflammatory Disease Section, National Human Genome Research Institute, National Institutes of Health, Bethesda, United States

###### **Correspondence:** P. Pimpale Chavan

**Introduction:** SDI score measures irreversible damage in 12 organ systems, present for at least 6 months resulting from cSLE or its treatment.

**Objectives:** To measure SDI and analyse some associated factors which may contribute to long term damage.

**Methods:** After ethics approval 50 patients with cSLE regularly following up for *at least* a year at the first study visit were identified and damage was measured using SDI *at two separate visits six months apart.* Patients with monogenic lupus, mixed connective tissue disease, drug-induced lupus, were excluded.

**Results:**

Demography of our patients, SDI and its damage associations and comparison with other cross-sectional studies appear in Table 1. 28% (14/50) of patients had damage in one or more organ systems, with 20% showing damage in 1, 6% in 2 and 2% in 3 organ systems. Damage was observed in ocular (9/50;18%), neuropsychiatric (NPS) (7/50;14%), renal (2/50;4%) and in musculoskeletal (1/50;2%) systems respectively; and was absent in pulmonary, cardiac, gastrointestinal, peripheral vascular, and cutaneous systems. Growth failure was observed in 9 (18%) patients and none had delayed puberty. A high SDI score was associated with NPS involvement (p-value 0.002), prolonged steroid usage (p-value 0.04) and growth failure (p-value 0.04). No corelation was observed with gender, age at diagnosis, time to diagnosis, disease duration, number of diagnostic criteria, renal disease and cyclophosphamide use.

**Conclusion:** A striking feature is the progressive reduction in the number of patients with damage since Ravelli’s first study in 2003 with the rest of the studies comparing similar numbers and similar or even longer times to SDI evaluation. SDI scores were lower in our patients compared to other studies probably due to inclusion of only regularly visiting (and thus probably adherent) patients. Using steroids in lowest possible doses, greater vigilance towards patients with NPS involvement, and growth monitoring are crucial for best outcomes. Our study additionally examined diagnostic delay as an associated factor and did not find corelation with SDI.

**Disclosure of Interest**

None declared

**Table 1 (abstract P257).** Demographic data, SDI and its association comparison across various cross-sectional studies

Author. citation	Ravelli et al. Arthritis Care Res. 2003 Aug 15;49(4):501-7	Lilleby et al. Clin Exp Rheumatol. 2005;23(2):261-9	Brunner et al. Arthritis Rheumatol. 2008 Feb;58(2):556-62	Salah et al. Rheumatol Int. 2009 Oct 1;29(12):1463	Sit JK et al. Pediatric Rheumatol. 2018 Dec 1;16(1):56	Our study 2019
**Sample size (N)**	387	71	67	148	59	50
**Gender (Females %)**	85.27	76	85	69.6	84.8	74
**SDI >0 (%)**	50.5	61	56.1	43.9	33.9	28
**Median SDI (IQR)**	1 (NM)	1 (NM)	1.7 (2.67)*	0.93 (1.37)*	0 (NM)	0 (0-1)
**Median age at diagnosis (years) (IQR)**	12.4 (NM)	14.5 (NM)	12.7 (2.5)*	10.5 (2.75)*	13 (12-15)	11.8 (10-13.6)
**Median age at study visit (years) (IQR)**	16.6 (NM)	26.5 (NM)	NM	17.1 (3.8)*	NM	18.8 (15.8-20.6)
**Median diagnostic delay (months) (IQR)**	NM	NM	NM	NM	NM	5 (2-10)
**Median disease duration (years) (IQR)**	4.6 (NM)	9.8 (NM)	NM	6.57 (3.59)*	7.8 (5.5-10.1)	6.6 (4.4-8.6)
**Damage associations**	Disease duration, NPS,CYC	Disease duration, Hypertension, Steroids	Disease activity, Steroids	Diagnostic criteria, age at onset, disease duration, NPS	Age at diagnosis, NPS, major organs involved, disease flares /infection	NPS, Steroids (>0.3mg/kg/day), growth failure

* = mean (SD), *NM* not mentioned, *NPS* neuropsychiatric system, *CYC* Cyclophosphamide

### P258 IFN-I score evaluation and genetic analysis in 40 pediatric sle patients: the preliminary data of cross-sectional study

#### R. Raupov^1^, E. Suspitsin^1,2^, R. Mulkidzhan^2^, E. Kalashnikova^1^, N. Lubimova^3^, E. Kuchinskaya^3^, A. Kosmin^2^, M. Kostik^1,3^

##### ^1^Saint-Petersburg State Pediatric Medical University; ^2^Molecular diagnostics, N.N.Petrov Institute of Oncology; ^3^Almazov National Medical Research Centre, Saint-Petersburg, Russian Federation

###### **Correspondence:** R. Raupov

**Introduction:** systemic lupus erythematosus (SLE) is a heterogenic multisystem autoimmune disease. Hyperactivation of interferon type I (IFN-I) system is one of basic pathogenic mechanisms in SLE. Genetic studies revealed a substantial number of genomic loci linked to SLE susceptibility.

**Objectives:** to evaluate IFN-I score and lupus-associated genetic variants in pediatric SLE patients.

**Methods:** 40 SLE patients (33 girls, 7 boys) under 18 years old were enrolled in the study. The data on clinical manifestations, laboratory findings at the onset of the disease and at the point of interferon signature assessment were evaluated. Interferon score was assessed by RT-PCR quantitation of 5 IFN I-regulated transcripts; median relative expression of ≥2 was considered as a cut-off. Genetic analysis was performed in 21 patients using clinical exome sequencing (CES, Roche IDP panel).

**Results:** 31 (77.5%) patients had increased IFN-I score, which was associated with nephritis (28% vs 0%, p=0.036) and ANA-positivity (87% vs 56%, p=0.043). Patients with increased IFN-I score needed more aggressive treatment (rituximab or cyclophosphamide, 71.0% vs 33%, p=0.040). There was no correlation between IFN-score and disease activity indexes (SELENA-SLEDAI, ECLAM). CES revealed rare pathogenic/likely pathogenic variants in 11 (52.5%) patients. Of those, 7 children had mutation in genes associated with nucleic acid sensing and IFN-signaling, two carried variants of genes associated with apoptotic and immune complex (IC) clearance and two had mutations in both groups (Table 1). All the patients with mutations had elevated IFN I score.

**Conclusion:** the majority of pediatric SLE patients have hyperactivation of IFN-I pathway. IFN-I score was associated with nephritis and aggressive treatment, but not with disease activity. Half of the patients had pathogenic/likely pathogenic mutations in SLE-related genes.

**Acknowledgements**

This work was supported by the RSF grant № 20-45-01005

**Disclosure of Interest**

None declared

**Table 1 (abstract P258).** See text for description

Nucleic acid sensing and IFN-signaling	Apoptotic and IC clearance	Variants found in combination
**RNASEL** NM_021133 c.1880A>G (p.K627R)*	**CR1** NM_000573 c.313C>T (p.R105C) **CR1** NM_000573 c.4681G>A (p.V1561M)	**CR1** NM_000573 c.1716G>T (p.Q572H) **TLR1** NM_003263 c.893C>T (p.S298F)
**DDX58** NM_014314 c.2590_2591insTTCT (p.C864Ffs*9)	**C1QA** NM_015991 c.334C>T (p.Q112X) homozygous	**DNASE1** NM_005223 c.460C>G (p.P154A); **CR1** NM_000573 c.1716G>T (p.Q572H)
**TLR1** NM_003263 c.2031G>C (p.K677N)		
**RNASEH2B** NM_024570 c.295C>T (p.H99Y)		
**TLR3** NM_003265 c.1311C>A (p.D437E)		

*mutation detected in monozygous twins

### P259 Acquired von willebrand disease in a child as early manifestation of systemic lupus erythematosus

#### K. Ruchti^1^, F. Achini-Gutzwiller^2^, M. Albisetti^2^, S. Palmer Sarott^1^

##### ^1^Rheumatology; ^2^Hematology, University Children's Hospital, Zurich, Switzerland

###### **Correspondence:** K. Ruchti

**Introduction:** Acquired von Willebrand disease (AvWD) is a bleeding disorder, which is extremely rare in children. Unlike patients with inherited von Willebrand disease, these patients typically have no personal or family history of bleeding. AvWD is associated with a variety of underlying disorders.

**Objectives:** Based on clinical and laboratory findings we present a case of paediatric AvWD associated with juvenile systemic lupus erythematosus (jSLE).

**Methods:** A previously healthy 12-year-old female with no personal or family history of bleeding disorders presented with hemodynamically relevant anemia (hemoglobin 48 g/l) due to menorrhagia and moderate thrombocytopenia. Laboratory investigations revealed markedly reduced Factor VIII, reduced von Willebrand Factor (vWF) ristocetin cofactor, reduced vWF activity and vWF antigen leading to the diagnosis of AvWD. Screening for underlying disease was negative for malignancy and primary or acquired immunodeficiencies. Further investigations found non-neutralizing anti-vWF IgG and a mildly elevated antinuclear antibody (ANA) titer (1:640; <1:320).

Initial response to infusion of plasma-derived vWF and immunoglobulins was poor, and menorrhagia was controlled with a combined hormonal contraceptive pill and tranexamic acid.

Six weeks later, the patient developed proteinuria in the nephrotic range while maintaining normal renal function leading to consultation of the rheumatology department. Further investigations showed decreased complement, increased ANA titer (1:1280) and positive double stranded DNA antibodies. Renal biopsy confirmed mesangial proliferative lupus nephritis (ISN/PRS Class I/II). Retrospectively, the patient described a malar rash.

**Results:** jSLE was diagnosed, based on the ACR/EULAR as well as the SLICC criteria. First-line treatment with intravenous steroid pulse and oral maintenance therapy with mycophenolate mofetil and steroids was introduced. Four weeks later, the proteinuria completely resolved and vWF levels increased significantly.

**Conclusion:** This case illustrates once more that jSLE can present with a wide variety of symptoms – in this patient with menorrhagia due to an acquired bleeding disorder caused by autoantibodies against vWF. AvWS likely occurs in association with an underlying disorder, and search for an immune-mediated pathogenesis should be included in the workup. Keeping the differential diagnosis of jSLE in mind, even when faced with unusual clinical symptoms, can facilitate early recognition of the disease. Early specific jSLE treatment can decrease the risk of long-term organ damage and, as seen in this case, it can lead to normalization of vWF.

**Patient Consent Received**

Yes

**Disclosure of Interest**

None declared

### P260 Mucocutaneous manifestations of systemic lupus erythematosus - case series from India

#### N. S^1^, S. Ravindran^2^, A. Romana^1^, S. J^1^, V. Gornale^1^, J. Raghuram^3^, A. P. Rao^2^

##### ^1^Pediatrics, Indira Gandhi Institute of Child Health; ^2^Pediatrics, Manipal hospital; ^3^Pediatrics, Columbia Asia hospital, Bangalore, India

###### **Correspondence:** N. S

**Introduction:** Cutaneous involvement is seen in 60-85% of patients of Pediatric Systemic Lupus Erythematosus (pSLE) and can be the earliest manifestation of the disease in 25% of patients.

**Objectives:** This study aims to analyze the mucocutaneous manifestations in a cohort of pSLE patients attending a pediatric rheumatology clinic in the southern part of India.

**Methods:** A retrospective data collection of pSLE patients attending a pediatric rheumatology clinic in Bangalore, India was carried out. A total of 133 patients of pediatric SLE fulfilling Systemic Lupus International Collaborating Clinic (SLICC) criteria were enrolled.

**Results:** The male: female ratio was 1:4. The mean age at presentation was 12.16(+/-2.35) years. The frequency of various cutaneous manifestations is listed in the table. 113(84.96%) patients had mucocutaneous manifestations. Acute Cutaneous Lupus (ACLE) was the most common cutaneous manifestation (79.69%). Rarer manifestations like toxic epidermal necrolysis (TEN) like ACLE, bullous lupus, lupus panniculitis and chilblain lupus was noted in one patient each. Among the patients with mucocutaneous manifestations, hematological involvement was noted in 100(88.49%) patients in the form of either or combination of anemia (81.41%), thrombocytopenia (25.64%) and leukopenia (19.46%). Renal involvement was seen in 51(45.13%) patients, neurological manifestations in 29(25.6%) patients. Hypocomplementemia in the form of low C3 noted in 57(50.44%) and low C4 in 39(34.51%) patients. Antinuclear antibodies were positive in all patients and dsDNA was positive in 68(60.17%) patients.

**Table:** 

**ACLE**	106(79.69%)
Maculopapular lupus rash	60(45.11%)
Photosensitive lupus rash	58(43.60%)
Malar rash	56(42.10%)
**SCLE**	5(3.75%)
**CCLE**	
Discoid rash	17(12.78%)
**LE non specific skin lesions**	
Oral ulcer	57(42.85%)
Cutaneous vasculitic rash	51(38.34%)
Non scarring alopecia	25(18.79%)

**Conclusion:** Mucocutaneous manifestations are one of the most common manifestations of pSLE and help diagnose pSLE. Skin rashes mirror systemic disease activity. It is important for a clinician to identify the clues these provide and titrate treatment to achieve remission.

**Patient Consent Received**

Yes

**Disclosure of Interest**

None declared

### P261 Capillary scleroderma pattern and disease damage in childhood-onset systemic lupus erythematosus: important lessons from longitudinal follow-up

#### D. Schonenberg-Meinema^1^, S. Bergkamp^1^, A. Nassar-Sheikh Rashid^1^, M. Gruppen^1^, W. Armbrust^2^, K. Dolman^3^, P. Hissinkmuller^4^, J. Swart^5^, S. Kamphuis^6^, V. Smith^7^ on behalf of EULAR study group on Microcirculation in Rheumatic Diseases, J. M. van den Berg^1^

##### ^1^Pediatric Immunology, Rheumatology and Infectious Diseases, AMSTERDAM UMC, Amsterdam; ^2^Department of Pediatric Rheumatology and Immunology, University Medical Center Groningen, Broningen; ^3^Department of Pediatrics, Onze Lieve Vrouwe Gasthuis, Amsterdam; ^4^Department of Pediatric Rheumatology, Leiden University Medical Centre, Leiden; ^5^Department of Pediatric Immunology, University Medical Center Utrecht, Utrecht; ^6^Department of Pediatric Rheumatology, Erasmus University Medical Centre, Rotterdam, Netherlands; ^7^Department of Rheumatology, University Hospital Ghent, Ghent, Belgium

###### **Correspondence:** D. Schonenberg-Meinema

**Introduction:** Abnormalities in nailfold capillaries from systemic lupus erythematosus (SLE) patients, visualized by capillaroscopy, have been described in many studies. A capillary scleroderma pattern has been described in SLE with percentages varying from 3 to 26%. It has been suggested that SLE-patients with a capillary scleroderma pattern might be patients with (subclinical) overlapping features from other connective tissue diseases (CTD), such as SSc and dermatomyositis. Also, it has been stated that the finding of a scleroderma pattern in SLE patients might be associated with the occurrence of Raynaud’s phenomenon and the presence of anti-RNP antibodies and that these patients are at risk for pulmonary arterial hypertension or pulmonary fibrosis. Our recent cross-sectional study in childhood-onset SLE (cSLE) patients showed that a scleroderma pattern in nailfold capillaries can be observed in patients with typical SLE symptoms (such as lupus nephritis, butterfly rash, serositis, aphtous ulcers, leukopenia, thrombocytopenia) without any clinical signs of SSc.

**Objectives:** To observe, longitudinally, if nailfold capillary patterns in childhood-onset SLE (cSLE) change over time and if a scleroderma pattern is associated with higher disease activity, -damage or scleroderma-like features.

**Methods:** Prospective data from cSLE-patients (onset < 18 years) were analyzed. Disease activity was defined by SLEDAI score and disease damage by SDI index. A scleroderma pattern was defined according to the ‘Fast track algorithm’ from the EULAR Study Group on Microcirculation in Rheumatic Diseases. An abnormal capillary pattern, not matching a scleroderma pattern, was defined as ‘microangiopathy’.

**Results:** We studied 53 cSLE patients with median disease onset of 14 years (IQR 12.5-15.5 years), median SLEDAI score at diagnosis was 11 (IQR 8-16). N=9 (17%) showed a scleroderma pattern (ever) and n=37 (70%) showed microangiopathy. N=30 patients had follow-up capillaroscopy of which 24/30 (80%) showed no changes in capillary pattern over time. Median SLEDAI score at diagnosis did not differ between capillary pattern groups. A scleroderma pattern in cSLE was significantly associated with the presence of anti-RNP antibodies and with occurrence of disease damage. Patients with a scleroderma pattern did not develop clinical features for systemic sclerosis during follow-up (range 1-10 years after diagnosis), but showed significantly increased risk for SLE-related disease damage (Hazard ratio 9.4, p=0.003).

**Conclusion:** This prospective study shows that the majority of abnormal capillary patterns in cSLE do not change over time, irrespective of treatment and SLE disease activity. Capillary scleroderma pattern in cSLE might reflect a more severe disease course and seems a prognostic red flag for disease damage.

**Disclosure of Interest**

None declared

### P262 Review of the worldwide epidemiological data of systemic lupus erithematosus

#### V. Sevostyanov, I. Razumov, E. Zholobova

##### I.M. Sechenov First Moscow State Medical University (Sechenov University), Moscow, Russian Federation

###### **Correspondence:** V. Sevostyanov

**Introduction:** Systemic lupus erythematosus (SLE) is a systemic autoimmune disease of unknown etiology, which is based on a genetically determined disorder of immune regulation with the development of immune inflammation in the tissues of various organs.

**Objectives:** A review of new epidemiological data of the worldwide incidence and prevalence of SLE in children.

**Methods:** Studies searched in the electronic databases PubMed.

**Results:** 218 studies were screened, 8 epidemiological studies of SLE since 2011 was undertaken, 8 countries included in the review. Prevalence. Europe: Italy - prevalence was 15.3; The Russian Federation - prevalence was 0,7. Asia: Singapore - prevalence was 6.3; Oman: Sharqiya - prevalence was 12.3, Dhofar - prevalence was 3.61, Dakhiliyah - prevalence was 3.17, Wusta - prevalence was 3.05, Dhahirah - prevalence was 2.71, Batinah - prevalence was 2.49, Muscat - prevalence was 2.45; The Republic of Korea - prevalence was 5.35. North America: USA - prevalence was 9.73 per 100,000 children. Incident. Europe: Italy - incidence was 3.8. Asia: The Republic of Korea - incidence was 2.20; Vietnam - incidence was 0.4. Oceania: New Zealand - incidence was 0.52; Australia - incidence was 0.32. North America: USA - incidence was 2.22 per 100,000 children.

**Conclusion:** An analysis was made of articles on morbidity. The data is heterogeneous across countries, requiring further in-depth study.

**Patient Consent Received**

No

**Disclosure of Interest**

None declared

### P263 Vitamin D deficiency in patients with systemic lupus erythematosus (SLE)

#### L. Bohmat^1,2^, I. Bessonova^3,4^, N. S. Shevchenko^3,5^, I. Khadzhynova^3,5^

##### ^1^Department of rheumatology and comorbid states, V.N.Karazin Kharkiv national university; ^2^Department of pediatrics № 2, V.N. Karazin Kharkia National University; ^3^Department of rheumatology and comorbid states, SI Institute for Children and Adolescents Health Care of NAMS of Ukraine; ^4^Department of pediatrics; ^5^Department of pediatrics № 2, V. N. Karazin Kharkiv National University, Kharkiv, Ukraine

###### **Correspondence:** N. S. Shevchenko

**Introduction:** The modern literature discusses the role of vitamin D as an immune and inflammatory mediator involved in the pathogenesis of a number of autoimmune diseases, including systemic lupus erythematosus (SLE). A number of studies have shown a link between low levels of vitamin D and higher disease activity, damage of organs and systems.

**Objectives:** To determine the status of vitamin D in children and adolescents with SLE, to analyze the dynamics of the status of vitamin D against the background of supplementation with vitamin D.

**Methods:** 32 patients with SLE (28 girls and 4 boys) aged 7-18 years were examined. The study was conducted twice: the first one- in the absence of additional intake of vitamin D, the second one - after its 3 months supplementation in a dose of 2000 IU. Serum 25-hydroxyvitamin D [25(OH)D] levels were measured using chemiluminescent method (Cobas 6000, Roche Diagnostics, Switzerland).

**Results:** At the first examination the average level of vitamin D was 19.00 ± 1.32 ng / ml corresponding to the deficit. The concentration indicators varied in the range of 4.64 – 37.81 ng / ml. No differences in vitamin D levels from gender were found.

Re-examination revealed positive changes in the status of vitamin D: the mean values ​​of 25 (OH) D increased significantly (28.14 ± 3.03 ng / ml ; p <0.01) and varied in higher reference intervals (from 12,14 ng / ml to 47,83 ng / ml).

Vitamin D deficiency (concentration of 25(OH)D of less than 20 ng / ml) was diagnosed in the majority of patients (65,63%) during the first examination and much less frequently during the second examination (30.77%; p <0.01).

The optimal level of 25(OH)D (exceeding 30 ng / ml) was recorded in isolated cases during the first examination (9.38%) and much more often during the re-examination (46.15%, p <0.01).

Inverse correlation was found between the level of 25(OH)D and albuminuria values ​​(r = -0.805; p <0.05), which allows us to think about the relationship between vitamin D deficiency and the development of renal disorders in children with SLE.

A repeated examination of children and adolescents with SLE who received 25(OH)D drugs for a long time did not reveal a similar association.

**Conclusion:** The majority of children and adolescents with SLE are deficient in vitamin D. The results of the study suggest an important role of vitamin D in the development of renal impairment and the need to add vitamin D to the complex of therapy for this category of patients.

**Disclosure of Interest**

None declared

### P264 Spondyloenchondrodysplasia in a female toddler presenting as systemic lupus erythematosus

#### M. Tsinti^1^, V. Dermentzoglou^1^, A. Ntokou^2^, E. Tsitsami^1^

##### ^1^Pediatric Rheumatology Unit, First Department of Pediatrics, University of Athens, Medical School, Children's Hospital Aghia Sophia; ^2^Second Department of Pediatrics, Children's Hospital Aghia Sophia, Athens, Greece

###### **Correspondence:** M. Tsinti

**Introduction:** Systemic lupus erythematosus (SLE) onset during toddlerhood is very rare and a monogenic etiology should be suspected.

**Objectives:** To present the disease course of a case of spondyloenchondrodysplasia presenting as SLE with nephritis and thrombocytopenia.

**Methods:** Case presentation.

**Results:** A 14-month female toddler was hospitalized with sudden onset fever (39 °C), petechial rash and hyposphagma. Initial laboratory investigation revealed leukopenia, thrombocytopenia, nephrotic range proteinuria, glomerular hematuria and casts, increased ESR and positive Direct Antiglobbulin Test. On the diagnosis of immune thrombocytopenia Intravenous Immunoglobulin was initiated. The patient developed nephritic syndrome necessitating intensive antihypertensive treatment with ramipril, propranolol and felodipine. Fever persisted. Infectious causes and malignancies were excluded. Renal biopsy on the 11th day of persistent nephritic-nephrotic syndrome revealed diffuse global proliferative lupus nephritis with active lesions class IV-G (A). IgG, IgA, IgM values were normal, C3 and C4 profoundly decreased; C1, CH50 and alternative complement pathways were normal. Antinuclear Antibodies titre was 1/640, diffuse pattern; antiphospholipid antibodies and

Extractable Nuclear Antigen Antibodies were absent. Brain CT performed for persistent irritability revealed bilateral and symmetrical calcifications of the basal ganglia. Intravenous Methylprednisolone pulses and mycophenolate mofetil (MMF) were initiated. Fever subsided and White Blood Cells and platelet counts improved. Nephritis persisted after 5 Intravenous methylprednisolone pulses followed by methylprednisolone 2 mg/kg/d. MMF was changed to IV cyclophosphamide pulses fortnightly leading proteinuria into remission. After 4 cycles, cyclophosphamide was changed to MMF again due to persistent leukopenia. In 6 months the disease remitted. Periodically the patient had microscopic glomerular hematuria and leukopenia treated with mild increases of steroid dose. Stepdown of antihypertensives was impossible. Normalization of C3 and C4 levels excluded the diagnosis of primary hypocomplementemia. At the age of 25 months while SLE into remission, she developed walking difficulty, gradually progressing to spastic paraplegia, with normal spinal cord, brain MRI and lower limb muscle imaging. Whole Exome Sequencing revealed homozygocity for the likely pathogenic mutation 721G>A:p.D241N in the *ACP5* gene, causative for spondyloenchondrodysplasia with immune dysregulation. Skeletal survey revealed transverse sclerotic metaphyseal bands with irregular borders involving proximal and distal femur, proximal tibia and fibula, distal ulna and radius, bilaterally and platyspondyly of the vertebra throughout axial skeleton. During a 3 year follow-up the disease flared twice. Periods of high disease activity were complicated by urinary tract infection with bacteremia. Her height gradually fell below the 3^rd^ percentile despite initial improvement on the 1^st^ year of treatment, possibly a primary manifestation of spondyloenchondrodysplasia.

**Conclusion:** Spondyloenchondrodysplasia is a defect of IFNa signaling, associated with skeletal, neural and immune manifestations. Mutations in the *ACP5* gene have been associated with autoimmune manifestations however SLE as the initial disease manifestation, during toddlerhood has not been previously described.

**Patient Consent Received**

Yes

**Disclosure of Interest**

None declared

### P265 DNASE1L3 deficiency, new phenotypes and evidence for a transient type I interferon signaling

#### M. Tusseau^1^, E. Lovsin^2^, C. Samaille^3^, R. Pescarmona^4^, A. L. Mathieu^1^, M. C. Maggio^5^, V. Selmanovic^6^, M. Debeljak^7^, A. Dachy^3^, A. Janin^8^, L. Januel^8^, J. B. Gibier^9^, E. Chopin^10^, I. Rouvet^10^, D. Goncalves^11^, N. Fabien^11^, G. Rice^12^, G. Lesca^13^, A. Labalme^13^, P. Romagnani^14^, T. Walzer^1^, S. Viel^1^, M. Perret^11^, Y. Crow^15^, T. Avcin^2^, R. Cimaz^14^, A. Belot^1^

##### ^1^The International Center of Research in Infectiology, LYON, France; ^2^University Children's Hospital University Medical Center Ljubljana, Ljubljana, Slovenia; ^3^Nephrologie pediatrique, Hôpital Jeanne de Flandre, Lille; ^4^Immunology Laboratory, Hospices Civils de Lyon, Lyon Sud Hospital, LYON, France; ^5^University Department Pro.Sa.M.I. "G. D'Alessandro," University of Palermo, PALERMO, Italy; ^6^Clinical center University of Sarajevo, Sarajevo, Bosnia and Herzegovina; ^7^University Children's Hospital University Medical Center Ljubljana,, Ljubljana, Slovenia; ^8^Cardiogenetics Laboratory, Biochemistry and Molecular Biology Department, Lyon University Hospital, LYON; ^9^Univ. Lille, UMR9020-U1277 - CANTHER - Cancer Heterogeneity Plasticity and Resistance to Therapies, LILLE; ^10^Hospices Civils de Lyon, Centre de Biotechnologie Cellulaire et Biothèque, BRON; ^11^Immunology Department, Hospices Civils de Lyon, Centre Hospitalier Lyon Sud, LYON, France; ^12^Division of Evolution and Genomic Sciences, School of Biological Sciences, Faculty of Biology, Medicine and Health, University of Manchester, Manchester Academic Health Science Centre, MANCHESTER, United Kingdom; ^13^Genetics Department, Lyon University Hospital, LYON, France; ^14^ASST G. Pini, Milano Italy and Department of Clinical Sciences and Community Health, University of Milano, MILANO, Italy; ^15^Laboratory of Neurogenetics and Neuroinflammation, Institut Imagine, Université de Paris, PARIS, France

###### **Correspondence:** M. Tusseau

**Introduction:** Deoxyribonuclease 1 like 3 (DNASE1L3) is a secreted enzyme that has been shown to digest the extracellular chromatin derived from apoptotic bodies, and *DNASE1L3* pathogenic variants have been associated to a lupus phenotype. It is unclear whether interferon signaling is sustained in DNASE1L3 deficiency in humans.

**Objectives:** Here, we report on four patients with pathogenic variations in *DNASE1L3*, including 2 previously undescribed causal variants, and expand the phenotype from SLE to vasculitis with gut involvement. To explore whether or not the interferon cascade was strongly and sustainably induced, Interferon stimulated genes (ISGs) expression was assessed for each patient. We also review previous reports highlighting the spectrum of DNASE1L3 deficiency.

**Methods:** Identification of disease-causing variants was based on NGS sequencing in 3 out of 4 patients, and for one patient, coding regions of the *DNASE1L3* gene were directly sequenced by Sanger sequencing.

Type I interferon signature was determined using either quantitative reverse transcription polymerase chain reaction or nanostring technology, and serum IN-α2 concentrations was measured using simoa assay.

**Results:** Disease in one patient was characterized by lupus nephritis and skin lesions, while two others exhibited hypocomplementemic urticarial vasculitis syndrome. The fourth patient presented with early-onset inflammatory bowel disease. Contrary to canonical type-I interferonopathies, we noticed a transient increase of ISGs in blood, which reverted to normal with disease remission.

**Conclusion:** Reviewing previous reports, DNASE1L3-related disease appears to carry a significant risk of lupus nephritis and a poor outcome together with the presence of anti-neutrophil cytoplasmic antibodies (ANCA). DNASE1L3 deficiency may share the pathogenesis with C1q deficiency by affecting efferocytosis, and this report suggests that interferon production is not directly driven by *DNASE1L3* pathogenic variants.

**Patient Consent Received**

Yes

**Disclosure of Interest**

None declared

### P266 Disease activity, remission and flare in a Dutch childhood-onset systemic lupus erythematosus cohort: a pilot study

#### L. van den Berg^1^, J. Wahadat^1,2^, D. Timmermans^1^, K. van Rijswijk^1^, A. van Dijk^1^, S. Bakx^1^, M. Verkaaik^1^, S. Kamphuis^1^

##### ^1^Department of Pediatric Rheumatology, Erasmus University Medical Center; ^2^Department of Immunology, Erasmus University Medical Center Rotterdam, Rotterdam, Netherlands

###### **Correspondence:** L. van den Berg

**Introduction:** Childhood-onset systemic lupus erythematosus (cSLE) is a lifelong and potential life-threatening multisystem autoimmune disease. cSLE generally has a more severe course than SLE in adults with higher cumulative disease activity and earlier accrual of damage over time. Therefore, achieving and maintaining disease remission is specifically important for children with cSLE. Lupus Low Disease Activity State (LLDAS) is a disease index describing a state of low lupus activity in adults and could be a useful tool for a treat-to-target approach in cSLE as well.

**Objectives:** To investigate the course of disease activity in a prospective cohort of Dutch cSLE patients focused on achieving disease remission and medication use.

**Methods:** All patients fulfilled the Systemic Lupus International Collaborating Clinics classification criteria. Clinical characteristics and medication use were prospectively collected. Disease activity was measured with BILAG-2004, SELENA-SLEDAI and Physician Global Assessment (PGA). In short, LLDAS is defined by SELENA-SLEDAI ≤ 4 without an increase since the previous visit, SELENA-SLEDAI-PGA ≤1.0, and treatment with maintenance dose of immunosuppressants and/or prednisone ≤ 7.5 mg/day. Clinical remission (CR) is defined by PGA and SELENA-SLEDAI equal to 0. For CR with treatment (Tx), patients are allowed hydroxychloroquine, low dose prednisone (≤5 mg/day), maintenance dose of immunosuppressants and biologicals. For CR without Tx, patients are allowed hydroxychloroquine. Disease flare is defined as increase of 1 point in the PGA and/or score ≥12 or increase of >4 points in the SELENA-SLEDAI and/or having A or B in any domain of the BILAG-2004 since the previous visit.

**Results:** 54 patients (85% female, 52% white) with a total of 550 visits (median 10 per patient) were included. Organ system involvement was most often found in the hematological (69%) and mucocutaneous (67%) domains, 33% had renal involvement. BILAG-2004, SELENA-SLEDAI and PGA at diagnosis had a median of 10 (2-42), 9 (1-29) and 2.0 (0.5-3.0). After 6 months of follow-up, BILAG-2004, SELENA-SLEDAI and PGA had decreased to a median of 2 (0-13), 2 (0-16) and 0.3 (0.0-2.0). 49 children ever achieved LLDAS in a median of 179 days. 20 children ever achieved CR with Tx in a median of 406 days, and 12 ever achieved CR without Tx, median of 485 days. 28 children ever had a disease flare, median of first flare at 237 days. 87% of children were treated with hydroxychloroquine within 3 months after diagnosis. Mycophenolate mofetil (MMF) was the most frequently used immunosuppressant, 55% of children were treated with MMF within 6 months after diagnosis. 50% of children were treated with prednisone at diagnosis, median dose 0.76 mg/kg/day, 51% still had prednisone after 6 months but with reduced doses (median dose 0.16 mg/kg/day). At 1 year after diagnosis, 21% used prednisone (median dose 0.21 mg/kg/day).

**Conclusion:** In this cSLE cohort, LLDAS was a realistic goal, achieved in almost all children within 6 months after diagnosis and a useful (additional) guide for a treat-to-target approach. In concordance with European guidelines, prednisone use could be decreased significantly within 6 months after diagnosis, however paralleled with equal increase in use of immunosuppressants. We are currently extending this study in multiple Dutch centers to validate these results and relate achieving disease remission and prednisone use to damage accrual over time.

**Patient Consent Received**

Yes

**Disclosure of Interest**

None declared

## e-Poster viewing: New diseases

### P267 A novel presentation of a hereditary periodic syndrome: which variant is responsible?

#### S. Al-Mayouf, L. Akbar

##### Pediatric Rheumatology, King Faisal Specialist Hospital and Research Center, Riyadh, Saudi Arabia

###### **Correspondence:** L. Akbar

**Introduction:** Recently, plasminogen, its activators, and its receptors have gained more attention in inflammation regulatory processes, including release proinflammatory signaling molecules, and thus its role has implications for a wide spectrum of clinical manifestations.

**Objectives:** To present a case of homozygous plasminogen variant managed initially as periodic fever syndrome.

**Methods:** A retrospective report of a child who presented with a constellation of findings; which cannot fully be explained by one classified autoinflammatory disease.

**Results:** A 9-year-old boy presented at the age of 18-months with periodic fevers and vomiting every 3 weeks, lasting for 48-72 hours. Heterozygous variant of *MEFV* gene was detected (c.442G>C; p.E148Q). Over a period of 7 years he developed recurrent headaches, abdominal pain, dysphagia, failure to thrive, eosinophilic esophagitis, recurrent otitis media, pneumonias, bronchial asthma like exacerbations, eye redness, tearing, delayed wound healing, periodontitis, and loss of teeth. Also, he was found to have Pseudotumor cerebri. Immunological and infectious work-up were inconclusive. Colchicine and short courses of steroids were initiated. However, anakinra was added with a partial response. It is worth mentioning that his parents are first-degree cousins; he has a twin sister with severe cyclic GI manifestations, with remarkable response to anakinra. Other siblings had sickle cell disease suffered from similar complaints as the index case with varying degrees in severity. With the constellation of findings and the inadequate response to therapies, whole-exome sequencing was obtained revealing homozygous variant in plasminogen gene; coding for plasminogen. However, his twin was found to have heterozygous plasminogen variant.

**Conclusion:** Our case had a constellation of findings cannot fully be explained by one classified autoinflammatory disease. Although we have no definite diagnosis; we believe that plasminogen gene variant, with the coexistence of *MEFV* gene variant might contribute to the clinical manifestations. Further studies needed to confirm this finding and allow more definitive conclusion.

**Patient Consent Received**

Yes

**Disclosure of Interest**

None declared

### P268 ARPC1B gene mutation: a syndrome combining immunodeficiency and autoinflammation

#### M. Marti Masanet^1^, B. López Montesinos^2^, L. Lacruz Perez^2^, M. I. Fernández González^1^, J. I. Aróstegui Gorospe^3^, A. Mensa Vilaró^3^, I. Calvo Penadés^2^

##### ^1^Pediatric Rheumatology Unit, Medical Research Institute Hospital La Fe; ^2^Pediatric Rheumatology Unit, HOSPITAL Universitari i Politecnic La Fe, Valencia; ^3^Immunology. Biomedical Diagnostic Center, Hospital Clínic, Barcelona, Spain

###### **Correspondence:** M. Marti Masanet

**Introduction:** ARPC1B deficiency is a rare syndrome caused by mutations in this gene, which consists in an actinopathy and which clinically combines immunodeficiency, allergy and autoinflammation.

**Objectives:** To present the case of a child with a rare disease such as ARPC1B deficiency.

**Methods:** Male with consanguineous parents and disease onset at 27 days of life with deep oral ulcers that progressively worsened, fever and papulo-purpuric skin lesions that appeared spontaneously or in the context of trauma and that subsequently were ulcerated. He associated progressive anemia, receiving several transfusions, and severe bloody liquid stools. Blood tests showed elevated CRP, procalcitonin and ferritin, platelets in the lower limit and high leucocytosis with neutrophilia. It was performed lumbar puncture (with negative cultures), brain ultrasound and cardiology study (without significant pathological findings), abdominal ultrasound (findings suggestive of colitis were observed), skin biopsy (with pathological result compatible with leukocytoclastic vasculitis) and bone marrow aspirate that ruled out malignancy.

Given the significant inflammatory component (and no response to antibiotic treatment), it was decided to administer intravenous immunoglobulin doses and systemic corticosteroid therapy was initiated. Then, the patient improved clinically and didn’t required new transfusions. But when reducing corticosteroid dose, there was a point when the symptoms got worse again.

It should also be noted the growth failure that didn’t improve as the weeks went by and the presence, during the admission, of several infections, such as a P. aeuroginosa abscess in a vaccination area and CMV primary infection.

In one of the skin flares, a biopsy was repeated, and the result was compatible with polyarteritis nodosa. Immunological study was extended: the study for chronic granulomatous disease, leukocyte adhesion deficit and functional study of the IL-10 pathway (related to neonatal Crohn's disease) were normal. An angio-MRI was also performed, in which no other vascular alterations were observed, ADA2 enzyme activity was normal and an interferon signature was also negative. At this time, due to suspicion of a possible autoinflammatory disease, a targeted clinical exome was requested and, given the important vasculitic component of the clinical manifestations, mycophenolate mofetil was associated to the treatment. The patient remained stable with this treatment.

**Results:** It was done an exhaustive genetic study. First, nucleotide variants for recessive and X-linked pattern variants were tested. And later, a copy number variation analysis was made. This last study showed a big deletion in the ARPC1B gene, affecting the exomes 2,3 and 4.

During the clinical evolution, the patient has continued presenting serious infections (like skin abscess and CMV reactivations), it was impossible to reduce corticosteroid dose lower than 1.2 mg/kg/day, he needed a nasogastric catheter to improve nutrition and, actually, the hematopoietic stem cell transplantation has been turned on.

**Conclusion:** ARPC1B defiency is characterized by a very early onset and clinical presentation includes growth failure, fever, vasculitic skin lesions, infections and gastrointestinal symptoms. Platelet alterations are characteristic of actinophaties. The autoinflammatory manifestations seem to respond to treatment with corticosteroids, mycophenolate mofetil and sirolimus, although hematopoietic stem cell transplantation is considered the only curative option.

**Patient Consent Received**

Yes

**Disclosure of Interest**

None declared

## e-Poster viewing: Scleroderma and related syndromes

### P269 The impact of COVID-19 on clinical course and treatment among patients with juvenile systemic sclerosis

#### A. Adrovic, M. Yildiz, F. Haslak, S. Sahin, O. Koker, K. Barut, O. Kasapcopur

##### Pediatric Rheumatology, Istanbul University-Cerrahpasa, Cerrahpasa Medical Faculty, Istanbul, Turkey

###### **Correspondence:** A. Adrovic

**Introduction:** The Corona virus disease -2019 (COVID-19) caused by the novel Severe Acute Respiratory Syndrome Coronavirus-2 (SARS-CoV-2) made a sustainable impact on health care worldwide. Till know, there are limited data regarding the impact of SARS-CoV-2 pandemic on patients with juvenile systemic sclerosis (JSS).

**Objectives:** We aimed to explore the influence of SARS-CoV-2 pandemic among patients with JSS. In this context, we performed a web-based survey by focusing on patients’ complaints, accessibility to health care and compliance of routine treatment.

**Methods:** The questionnaire including 28 questions was send by the telephone or by the e-mail to patients diagnosed with JSS. The first part of the form included questions regarding the demographic characteristics of patients. The second part included multi-choice of questions regarding the clinical characteristics of patients, treatment they used for the JSS and the continuation and/or discontinuation of regular follow up during the pandemic.

**Results:** Of the 27 responders, 22 (81.5%) were females. The mean age at the time of the study was 17.3 ±3.5 years. Five patients (18.5%) responded that they had deterioration of the disease during the last 6 months. Six patients (22.2%) reported the irregular usage of their routine scleroderma treatment during pandemics. Nine patients (33.3%) missed their routine clinic control since the proclamation of the SARS-CoV-2 pandemics: 2 patients due to a lockdown, 4 patients were afraid to be infected by the SARS-CoV-2 if they go to hospital, 1 patient was feeling well so she decided to postpone the visit and 1 patient took the advantage of telemedicine. Seven patients (25.9%) had a contact with family member diagnose as COVID-19. A total of 4 patients (14.8%) were diagnosed as COVID-19 and only 1 (3.7%) of them was hospitalized. All of those 4 patients had symptoms of fever, fatigue, myalgia and headache but they completely recovered. Nine patients were under biological treatment (tocilizumab) but only one of them was diagnosed as COVID-19.

**Conclusion:** This is the first study evaluating the impact of COVID-19 on patients with JSS. The COVID-19 hasn’t significantly disrupted the medical care in JSS patients. The telemedicine could be an acceptable option for patients disenabled to come to hospital due to different reasons.

**Patient Consent Received**

Yes

**Disclosure of Interest**

None declared

### P270 Juvenile systemic sclerosis: about 9 cases

#### K. Danaoui^1^, H. Nassih^1^, K. Oujennane^2^, R. Elqadiry^3^, A. Bourrahouat^3^, S. Amal^2^, I. Aitsab^3^

##### ^1^B Pediatric Ward, Mohamed VI University Hospital Center Marrakesh, Morocco; ^2^Dermatology Ward; ^3^B Pediatric Ward, Mohammed VI University Hospital, Marrakesh, Morocco

###### **Correspondence:** K. Danaoui

**Introduction:** Juvenile systemic sclerosis is a rare chronic and autoimmune rheumatic disease ; whose symptoms begin on average at age 11 affecting the skin and other organs. It has a high morbidity and mortality, being a disease of which there is no consensus of treatment, so it becomes a challenge for the specialists who manage it .

**Objectives:** To report clinical manifestations, management and outcome of systemic sclerosis in children.

**Methods:** We conducted a retrospective descriptive study including 9 cases of juvenile systemic sclerosis.

**Results:** All our patients were girls. The mean age at onset was of 10-year-old, with extremes of 6 and 14-year-old. The mean interval between disease onset and diagnosis was of 9 months. Telltale symptoms of systemic sclerosis were tightening of the skin in all cases, protracted arthritis in 6 cases, lower-limb functional capacity impairment in two patients, recurrent skin ulcers in one patient, dysphagia in one patient, and prolonged fever in 2 patients. Skin biopsy was performed in all cases and diagnosis was made on the basis of the ACR-EULAR classification criteria for systemic sclerosis. Laboratory work-up found high acute phase reactants (ESR, CRP) in all patients. Meanwhile, three children had microcytic anemia. Electromyogram found peripheral neuropathy in one case. In the other hand, esogastroduodenal fibroscopy found significant thickening of the walls of the upper gastrointestinal tract in one patient. As for the immunological assessment, the antinuclear antibody test was positive in two patients, the anti-double stranded DNA test was positive in one patient, and the anti Scl-70 antibody was present in one case. Systemic lupus erythematosus was associated with systemic sclerosis in one girl. At last, one patient presented with proteinuria above 500 mg/24h, and for whom a kidney biopsy was performed and showed focal segmental glomerulosclerosis. Management was based on steroids associated with methotrexate as a first line treatment, and for a minimal period of 24 months in all cases. In one case, mycophenolate mofetil was latter added to obtain remission. All our patients were under physiotherapy and psychological support. Evolution was marked by complete remission in 5 patients and partial remission in 3 patients. Unfortunately, we deplore the death of one of our patients following a septic shock.

**Conclusion:** the delay in the diagnosis and management can lead to permanent sequalae in children affected by systemic sclerosis, as well as a major impact on their quality of life. That is why, more awareness is needed among pediatricians to improve outcome of this disease.

**Patient Consent Received**

Yes

**Disclosure of Interest**

None declared

### P271 Male juvenile systemic sclerosis patients have more severe disease: results from the international juvenile scleroderma inception cohort. www.juvenile-scleroderma.com

#### I. Foeldvari^1^, J. Klotsche^2^, O. Kasapcopur^3^, A. Adrovic^3^, K. Torok^3^, M. T. Terreri^3^, A. P. Sakamoto^3^, F. Sztajnbok^3^, B. Feldman^3^, V. Stanevicha^3^, J. Anton^3^, R. Khubchandani^3^, E. Alexeeva^3^, S. Johnson^3^, M. Katsicas ^3^, S. Sawhney^3^, V. Smith^3^, S. Appenzeller^3^, T. Avcin ^3^, M. Kostik^3^, T. Lehman^3^, E. Marrani^3^, D. Schonenberg-Meinema^3^, W.-A. Sifuentes-Giraldo^3^, N. Vasquez-Canizares^3^, M. Janarthanan ^3^, H. Malcova^3^, M. Moll^3^, D. Nemcova ^3^, A. Patwardhan^3^, M. J. Santos^3^, C. Battagliotti^3^, L. Berntson^3^, B. Bica^3^, J. Brunner^3^, R. Cimaz^3^, P. Costa Reis^3^, D. Eleftheriou^3^, L. Harel^3^, G. Horneff^3^, D. Kaiser^3^, T. Kallinich^3^, D. Lazarevic^3^, S. Nielsen^3^, K. Minden^2^, F. Nuruzzaman^3^, S. Opsahl Hetlevik^3^, Y. Uziel ^3^, N. Helmus^1^

##### ^1^Hamburg Center for Pediatric and Adolescent Rheumatology, Am Schoen Klinik Eilbek, Hamburg; ^2^German Rheumatism Research Center, Berlin; ^3^jSSc collaborative group, Hamburg, Germany

###### **Correspondence:** I. Foeldvari

**Introduction:** Juvenile systemic sclerosis (jSSc) is a rare disease with a prevalence of around 3 in 1,000,000 children. To better capture the clinical manifestations of jSSc the juvenile systemic sclerosis inception cohort (jSScC) has been prospectively enrolling patients with predetermined clinical variables over the past 12 years. One of the goals is to study the demographic, clinical features, and physician and patient reported outcome differences between male and female patients, to determine if characteristics are similar or different.

**Objectives:** To compare organ involvement and disease severity between male and female patients with juvenile onset systemic sclerosis (jSSc).

**Methods:** Demographics, organ involvement, laboratory evaluation, patient reported outcomes and physician assessment variables were compared between male and female jSSc patients enrolled in the prospective international juvenile systemic sclerosis cohort (jSScC) at their baseline visit.

**Results:** 175 jSSc patients were evaluated, 142 female and 33 male. Race, age of onset, disease duration, and disease subtypes (70% diffuse cutaneous) were similar between males and females. Active digital ulceration, very low body mass index, and tendon friction rubs were significantly more frequent in males. Physician global assessment of disease severity and digital ulcer activity was significantly higher in males. The composite pulmonary involvement was also more frequent in males, though not statistically significantly.

**Conclusion:** In this cohort, jSSc had a more severe course in males. This reflects the adult-onset SSc cohort data and parallels it in regards to increased digital ulcers, interstitial lung disease, and global severity. Differences from adult findings include no increased signal of pulmonary arterial hypertension or heart failure in male pediatric patients. While monitoring protocols of organ involvement in jSSc need to be identical for males and females, our findings suggest a higher index of suspicion of certain organ involvement in males.

Supported by the "Joachim Herz Stiftung"

**Disclosure of Interest**

None declared

### P272 How is pulmonary function assessed in juvenile systemic scleroderma patient? Do we have a good clinical standard? Results from the juvenile scleroderma inception cohort- www.juvenile-scleroderma.com

#### B. Hinrichs^1^, I. Foeldvari^2^, E. Alexeeva^3^, J. Anton^3^, S. Appenzeller^3^, L. Berntson^3^, J. Brunner^3^, P. Costa Reis^3^, B. Feldman^3^, G. Horneff^3^, M. Janarthanan ^3^, T. Kallinich^3^, O. Kasapcopur^3^, R. Khubchandani^3^, M. Kostik^3^, T. Lehman^3^, E. Marrani^3^, S. Nielsen^3^, F. Nuruzzaman^3^, A. Patwardhan^3^, M. J. Santos^3^, D. Schonenberg-Meinema^3^, V. Smith^3^, V. Stanevicha^3^, F. Sztajnbok^3^, M. T. Terreri^3^, K. Torok^3^, Y. Uziel ^3^, N. Helmus^2^

##### ^1^Children's pulmonology, Asklepios Klinik Nord – Heidberg; ^2^Hamburg Center for Pediatric and Adolescent Rheumatology, Am Schoen Klinik Eilbek; ^3^jSSc Collaborative Group, Hamburg, Germany

###### **Correspondence:** I. Foeldvari

**Introduction:** Juvenile systemic scleroderma (jSSc) is a rare, but potentially life-threatening disease. Around 45% of the patients develop interstitial lung disease and this can lead to relevant mortality. Pulmonary function tests (PFT). Including forced vital capacity (FVC) and lung carbon monoxide diffusion (DLCO), are key for the screening of interstitial lung disease. PFT require an optimal patient cooperation and coordination. For example, the patient has to hold the breath for 10 seconds. Additionally, the graph of the breath holding maneuver has to be carefully observed, if there is a premature expiration, numbers have to be judged with suspicion. We were interested how in the real world these problems are addressed in children.

**Objectives:** to assess how PFT is assessed in the participating pediatric rheumatologic centers in the juvenile scleroderma inception cohort

**Methods:** We conducted a survey among the pediatric rheumatologists and associated pediatric pulmonologists, who are participating in the prospective juvenile scleroderma inception cohort (jSScC). We asked them some simple questions about the standard of care regarding assessment of the PFT.

**Results:** 65% (26/40) of the surveyed participants of jSScC responded. From the participating centers 96%(25/26) assess the pulmonary function test in a specific pediatric setting. 65% (17/26) regularly assess DLCO. In 77% (20/26) of the centers the respiratory technician that conducts the PFT judges the level of cooperation of the child and the pediatric pulmonologist, who evaluates the results, also makes a judgement on the reliability of the test. We asked a specific question regarding the duration of the breath holding time setting in the lung function system. 32% (8/25) set the breath holding time under 7 seconds and 8% (2/26) at 7 seconds and 20% (5/25) between 7 and 10 seconds and 28% (7/25) exactly 10 seconds.

**Conclusion:** We could demonstrate that in 96% of the responding centers PFT are carried out in specialized pediatric pulmonology departments, which are aware of the methodological problems and change their breath holding time to an appropriate time to gain as many reliable tests as possible. There is still a wide range of the applied breath holding time and even in centers carrying for jSSc patients, 23% do not assess DLCO.

**Disclosure of Interest**

None declared

### P273 Patients with juvenile systemic sclerosis have a distinct pattern of organ involvement. Results from the juvenile systemic sclerosis inception cohort. Temic sclerosis inception cohort. www.juvenile-scleroderma.com

#### I. Foeldvari^1^, J. Klotsche^2^, O. Kasapcopur^3^, A. Adrovic^3^, K. Torok^3^, M. T. Terreri^3^, A. P. Sakamoto^3^, F. Sztajnbok^3^, B. Feldman^3^, V. Stanevicha^3^, J. Anton^3^, R. Khubchandani^3^, E. Alexeeva^3^, S. Johnson^3^, M. Katsicas ^3^, S. Sawhney^3^, V. Smith^3^, S. Appenzeller^3^, T. Avcin ^3^, M. Kostik^3^, T. Lehman^3^, E. Marrani^3^, D. Schonenberg-Meinema^3^, W.-A. Sifuentes-Giraldo^3^, N. Vasquez-Canizares^3^, M. Janarthanan ^3^, H. Malcova^3^, M. Moll^3^, D. Nemcova ^3^, A. Patwardhan^3^, M. J. Santos^3^, C. Battagliotti^3^, L. Berntson^3^, B. Bica^3^, J. Brunner^3^, R. Cimaz^3^, P. Costa Reis^3^, D. Eleftheriou^3^, L. Harel^3^, G. Horneff^3^, D. Kaiser^3^, T. Kallinich^3^, D. Lazarevic^3^, S. Nielsen^3^, K. Minden^2^, F. Nuruzzaman^3^, S. Opsahl Hetlevik^3^, Y. Uziel ^3^, N. Helmus^1^

##### ^1^Hamburg Center for Pediatric and Adolescent Rheumatology, Am Schoen Klinik Eilbek, Hamburg; ^2^German Rheumatism Research Center, Berlin; ^3^jSSc collaborative group, Hamburg, Germany

###### **Correspondence:** I. Foeldvari

**Introduction:** Juvenile systemic sclerosis (jSSc) is a rare disease with a prevalence of around 3 in 1,000,000 children. To better capture the clinical manifestations of jSSc the juvenile systemic sclerosis inception cohort (jSScC) has been prospectively enrolling patients with predetermined clinical variables over the past 12 years. One of the goals is to study the demographic, clinical features, and physician and patient reported outcome differences between those with juvenile limited cutaneous (lc) compared to diffuse cutaneous (dc) disease subtypes, to determine if characteristics are similar or different between dc and lc jSSc.

**Objectives:** Evaluation of the baseline clinical characteristics of jSSc patients in the jSScC. Compare clinical phenotype between diffuse (dcjSSc) and limited cutaneous (lcjSSc) subtypes.

**Methods:** Demographic, physical examination, organ system evaluation, autoantibody profile, treatment, and patient and physician reported outcome variables were evaluated from the jSSc Inception cohort and summary statistics applied using chi-square test and Mann Whitney U-test comparing lcjSSc and dcjSSc subtypes.

**Results:** At the time of data extraction, 187 jSSc patients were enrolled in the cohort, 80% were Caucasian and 80% female. Diffuse cutaneous jSSc subtype predominated (72%). Median Disease duration was 2.5 years (1 – 4.4). Median age at Raynaud´s was 10.4 years (7.2 – 13.1) and median age of first non-Raynaud´s was 10.9 (7.4 – 13.5). Significant differences were found between dcjSSc versus lcjSSc, regarding several clinical characteristics. Patients with diffuse cutaneous subtype had significantly higher modified Rodnan skin score (p<0.001), presence of sclerodactyly (p=0.003), presence of Gottron’s papules (p=0.008), presence of telangiectasia (p=0.005), history of digital tip ulceration (p=0.001). Cardiac involvement was significantly higher in limited cutaneous jSSc subtype (p=0.001). Diffuse cutaneous jSSc patients had significantly worse scores for Physician Global Assessment of disease activity (35 vs 20; p<0.001) and disease damage (30 vs 15; p<0.001).

**Conclusion:** Results from this large international cohort of jSSc patients demonstrate significant differences between dcjSSc and lcjSSc patients. According to the general organ involvement and physician global scores, the dcjSSc patients had significantly more severe disease. These observations strengthen our previous findings of the unique organ pattern of pediatric patients.

Supported by the "Joachim Herz Stiftung"

**Disclosure of Interest**

None declared

### P274 A progressive rash with myositis and associated bony changes

#### A. Jones^1^, L. Paterson-Brown^1^, D. Greenblatt^2^, C. Lloyd^3^, V. Shivamurthy^1^

##### ^1^Paediatric Rheumatology; ^2^Paediatric Dermatology; ^3^Paediatric Radiology, Evelina Children's Hospital, London, United Kingdom

###### **Correspondence:** A. Jones

**Introduction:** Juvenile localised scleroderma is a rare autoimmune disorder causing functional disability and aesthetic sequelae^(1,2)^. It is usually considered a disease confined to skin and subcutaneous tissue but can extend to bones and has been linked to extracutaneous features, without development of systemic sclerosis^(3)^.

**Objectives:** We present a rare case of juvenile localised scleroderma with acute myositis and bony changes on magnetic resonance imaging (MRI) who initially presented with blaschkoid hyperpigmentation.

**Methods:** A case report is described.

**Results:** A 4 year old girl was referred with knee swelling and a limp. The mother described a 2 year history of rash spanning her right leg with review by dermatology in 2019 suggesting a diagnosis of atrophic lichen planus. Over time, the rash became increasingly hyperpigmented, was spreading and her right knee appeared more prominent with a limp. There were no systemic features.

Examination revealed extensive linear skin changes to her right leg from the dorsum of her foot over her buttock and hip, classical of linear scleroderma. There was accompanying muscle and fat loss, reduced strength and restriction of movement at the right ankle, knee and hip.

The clinical diagnosis prompted a range of investigations revealing the extent of fibrous bony change, acute myositis, muscle and fat atrophy.

**Table:** 

Inflammatory markers	CRP <1 ESR 3
Autoantibodies	Rheumatoid Factor 40 Antinuclear Antibody 1/1280 Ku antibody positive
Creatine Kinase	62
MRI	Reduction in muscle bulk and subcutaneous fat to the right thigh, calf and gluteals. Muscle oedema of the gluteal, anterolateral thigh and anterolateral lower leg muscles. Extensive bone marrow signal abnormality in the right hemipelvis, right femur and fibula.
Xrays	Chondroid calcifcation in the right proximal femur, no focal lucency. Right iliac sclerosis.
Histology	Mild hyperkeratosis, papillomatosis and acanthosis of the epidermis. perivascular and periadnexal inflammatory cell infiltrate. Subcutaneous fat appears fibrotic

Treatment was initiated with 3 days pulsed intravenous methylprednisolone (IVMP) followed by weekly IVMP for 4 weeks alongside subcutaneous methotrexate. Despite initial spread of cutaneous disease, treatment has halted disease progression and her strength is improving. Follow up MRI is planned to assess bone disease.

**Conclusion:** Studies following patients with juvenile onset localised scleroderma found approximately 50% of patients reported permanent sequelae, including contractures, deep tissue atrophy and limb length discrepancies^(4)^. Due to the progressive nature of this disease, early recognition and treatment has been proven to have better long-term outcomes^(1,4)^. In this patient, delay in diagnosis led to significant contractures, weakness, muscle and fat wasting which may cause complex functional and aesthetic sequelae into adult life. We are currently unsure of the relevance of the positive Ku antibody and bone changes.

1.Giorgia Martini et al. Disease course and long-term outcomes of juvenile localised scleroderma: Experience from a single pediatric rheumatology centre and literature review. *Autoimmun Rev*. 2018. 17(7): 727-734

2.Zulian F, Athreya BH et al. Juvenile scleroderma working group of the pediatric rheumatology European Society (PRES). Juvenile localized scleroderma: clinical and epidemiological features in 750 children. An international study. *Rheumatology*2006; 45(5): 614-20

3.Francesco Zulian, Cristina Vallongo, Patricia Woo et al. Localized scleroderma in childhood is not just a skin disease. *Arthritis & Rheumatism* 2005; 52(9): 2873-2881

4.Stephanie Saxton- Daniels, Heidi Jacobe. An evaluation of long term outcomes in adults with pediatric- onset morphea. *Arch. Dermatology* 2010; 146(9): 1044 - 1045

**Patient Consent Received**

Yes

**Disclosure of Interest**

None declared

### P275 Effectiveness of tocilizumab in an 11-year-old girl with localized scleroderma: a case report

#### T. Kawabe^1,2^, Y. Kawaguchi^2^, K. Urayama^3^, K. Mizuochi^4^, Y. Kaburaki^4^, S. Nagata^4^, N. Ishiguro^3^, M. Harigai^2^, T. Miyamae^1,2^

##### ^1^Pediatric Rheumatology, Institute of Rheumatology, Tokyo Women’s Medical University School of Medicine; ^2^Division of Rheumatology, Department of Internal Medicine; ^3^Department of Dermatology; ^4^Department of Pediatrics, Tokyo Women’s Medical University School of Medicine, Tokyo, Japan

###### **Correspondence:** T. Kawabe

**Introduction:** Localized scleroderma (LSc) is a chronic inflammation in the skin and soft tissues associated with autoimmunity, which subsequently leads to fibrosis. Fibrosis of the skin of the extremities induces joint deformity and contracture and inhibits normal growth of the affected limb. Although the clinical features of childhood-onset LSc have been well studied, optimal treatment has not been established at present. In recent years, several case reports have suggested the effectiveness of biological agents for LSc. It has been reported that interleukin-6 acts on fibroblasts to promote synthesis of collagen and differentiation into myofibroblasts, which strongly induces fibrosis. We experienced a case of LSc successfully treated with tocilizumab (TCZ), a humanized monoclonal anti-IL-6 receptor antibody.

**Objectives:** To evaluate the effectiveness of TCZ on skin sclerosing lesions and secondary dysfunction in a pediatric case of LSc during the adolescent growth spurt.

**Methods:** Case report.

**Results:** An 11-year-old girl presented with skin sclerosis of body trunk and lower extremities. Especially severe sclerosis spread from the thigh to the lower left leg at the age of three. The findings of skin biopsy were consistent with LSc. She also manifested limited flexion in her left knee, and MRI revealed arthritis in her left knee joint beneath the sclerotic lesion. Methotrexate ameliorated sclerosis, however joint symptoms and observations did not improve. Therefore, adalimumab and tacrolimus were applied at the age of four, and were very effective against arthritis. She achieved medication-free remission after the age of 8, and both joint and skin symptoms were stable without treatment.

At the age of ten, the sclerotic lesion on the right thigh enlarged, and her left knee joint pain and limited flexion flared up. Knee MRI and ultrasound findings did not dipict any results that would explain arthritis this time, and her arthralgia and limited flexion were attributed to skin sclerosis around the knee. There was an 18 x 6 cm of skin sclerosis on the right thigh and brown spots with atrophy from the left thigh to the lower leg. In addition, multiple skin sclerosis on the left axilla, back, left side abdomen, left upper arm, and right dorsum of the foot was newly identified. Skin re-biopsy of her right thigh showed increased collagen fibers in all layers of the dermis and fatty tissue, lymphocytic infiltration to blood vessels and skin appendages. These findings suggested relapse of LSc. There were no serological markers reflecting the disease activity. After methylprednisolone pulse therapy, intravenous TCZ (8 mg/kg/4 weeks) was started and her pain disappeared. She has received treatment with TCZ for seven months, and no new skin lesions appeared so far. The patient's height is increasing at a high rate of 1 cm per month; nevertheless, the skin sclerosis lesions showed improvement and reduction in size, her skin sclerosis have been gradually softened, the right thigh lesion diminished to 15 x 5.5 cm, and no secondary leg length difference appeared. Although limited flexion of left knee remained slightly, she can now crouch with her knees folded and ride a bicycle sitting down instead of standing up.

**Conclusion:** TCZ is widely used in juvenile idiopathic arthritis patients who require a biological agent in Japan. IL-6 activates innate immunity via T cells and B cells, which is involved in the pathogenesis of LSc. It is desirable to confirm the effect of blocking activity of IL-6 on skin lesions of LSc in vivo and to establish biomarkers that reflect the pathophysiology and therapeutic effects of TCZ.

**Patient Consent Received**

Yes

**Disclosure of Interest**

None declared

### P276 Sjögren disease and Sjögren syndrome in children

#### S. Olga, P. Nadejda, O. Maria, S. Valentina, K. Julia, A. Elena, N. Maria

##### Pediatric departament, I.M.Sechenov First Moscow State Medical University, Москва, Russian Federation

###### **Correspondence:** S. Olga

**Introduction:** Sjögren disease/syndrome (SD/SS) is uncommon in children. The standard clinical criteria used in diagnosis of adult Sjögren syndrome do not allow to make a diagnosis in children at an early stage. Xerostomia and xerophthalmia are the basic criteria of SD/SS more often revealed in late stage of the disease in children.

**Objectives:** To analyze and clarify the clinical and laboratory signs that indicates early stage of disease

**Methods:** Analysis of 28 childhood cases SD/SS was done. All patients (pts) had satisfacted for disease criteria (**Sjogren’s International Collaborative Clinical Alliance = SICCA, 2012г).** All children carried out physical examination, salivary glands ultrasound investigation, sialometry, sialography, tests for xerophthalmia. Lab test included the measuring of CBC, total protein (TP), α-, β- and γ-globulins, IgA, IgM, IgG, rheumatoid factor (RF), ANA, ENA-profile with anti-SSA/Ro and anti-SSB/La detection

**Results:** we observed 28 pts, aged from 3.5 to 17 years, the mean age 10.5 years, 24 girls and 4 boys (girls/boys = 7:1). SD was diagnosed in 13 girls. SS was diagnosed in 15 pts. Systemic lupus erythematosus (SLE) and SS – 11 pts ( 9 girls, 2 boys), systemic scleroderma (SSD) and SS – 2 girls, juvenile rheumatoid arthritis (JRA) and SS – 1 boy, juvenile dermatomyositis (JDM) and SS – 1 girl. Pts with SD (13) more often had mild course of the disease. Arthralgia, fatigue and low subfebrile temperature after acute respiratory infection was common complaints in all 13 pts (100%). Two girls had hemorrhagic nonspecific rush. Lab investigations revealed increased ESR in all pts (100%), leukopenia in 6 (46%) pts. Due to prolonged arthralgia, fatigue and subfebrile fever subsequent studies revealed hyper γ-globulinaemia (100%), ANA and RF in unexplained high titers (100%), and SSA/SSB antibodies (100%). Only two girls complained of dry mouth. And also two girls had episodes of recurrent parotitis. So the diagnosis was made on average 6-12 months after the onset by excluding other systemic diseases and by instrumental investigations. Salivary glands ultrasound investigation revealed initial stage of parotitis in all pts (100%) that was suggested by sialometry and sialography. As to xerophthalmia no one of patients complained of dry eyes. Low lacrimation was detected in 5 girls (38%). Low dose of prednisone and micophenolate mofetil (MF) have been used for treatment in all patients. In compare with SD course SS depend on associated rheumatic diseases and seems more aggressive. Strong fever up to 38C had all pts with SLE, one of with SSD and one pts with JRA. This two pts demonstrated high level of TP, significant hyper γ-globulinaemia with polyclonal secretion that required excluding lymphoma. Body weight loss had 6 children (40%). Generalized lymphoadenophaty had 8 pts (40%). Purpura and nonspecific rash registered in 9 (60%) children. Seven pts (46%) demonstrated Raynaud phenomenon. Six pts complained of dry mouth and 5 pts complained of dry eyes in disease onset. Twenty six pts (90%) with SS/SD demonstrated good response for immunosupressive treatment with reducing of clinical symptoms (fever, arthralgia, rush, function of salivary glands, lacrimation) after six month of treatment but not for lab parameters. Increased level of RF, ANA, anti-SSA/Ro and anti-SSB/La persisted in all patients for six month of follow up. In children with SS persistence of RF, ANA, anti-SSA/Ro and anti-SSB/La was strong despite more intensive immunosuppression. Two patients with SSD and JRA were not responsible for therapy.

**Conclusion:** SD/SS in children is characterized with late complain of xerophthalmia and xerostomia. Persistence of RF, ANA, anti-SSA/Ro and anti-SSB/La required the investigations for revealing of SD or SS

**Disclosure of Interest**

None declared

### P277 RAG 1 mutation: combined immunodeficiency and autoimmunity

#### P. P. Ramos, E. Faugier, H. Menchaca, E. Mercedes, M. De la Cera, N. De la Rosa, S. Rodríguez

##### Reumatología Pediátrica., Hospital Infantil de México Federico Gomez, Mexico, Mexico

###### **Correspondence:** P. P. Ramos

**Introduction:** Primary immunodeficiencies (PIDs) are no longer defined only by the tendency to infection, but also by characteristics of "immune dysregulation" including autoimmunity. Which represents a diagnostic challenge.

**Objectives:** Describe the case of a patient with overloap: SLE and scleroderma; and detection of the RAG 1 gene mutation.

**Methods:** Descriptive and observational case. Data obtained from the clinical record

**Results:** A 4-year-old female with a history of chronic dermatosis beginning in the first year of life, refractory to multiple treatments. Referred to the institution with a diagnosis of vitiligo. Skin biopsy: morphea variety scleroderma. Methotrexate started. He completed the immunological approach, integrating overlap syndrome by criteria of generalized scleroderma and systemic lupus erythematosus (SLE). Pulmonary high-resolution computed tomography (HRCT): cicatricial atelectasis in apical-posterior, lingular, and posterior basal segments; thickening of the interlobular interstitium, cylindrical bronchiectasis. Lung scan: left lung hypoperfusion. DMARD therapy was modified to mycophenolate mofetil, corticosteroid, and cyclophosphamide. Subsequently, she presented gastrointestinal symptoms and pulmonary HRCT without improvement, for which tocilizumab therapy was escalated. With the approach for PID by the association of autoimmune diseases; met European Society for Immunodeficiencies (ESID) criteria for combined immunodeficiency. He started monthly immunoglobulin replacement therapy. Exome sequencing: RAG 1 gene mutation. Currently under the protocol for allogeneic cell transplantation.

**Conclusion:** The particularity of the case of an autoimmunity association is described, manifested with an overlap of SLE and scleroderma; with combined immunodeficiency; with detection of the RAG 1 gene mutation and vitiligo that belongs to the spectrum of genetic alteration. The patient has criteria for allogeneic cell transplantation due to disease association, refractoriness to treatment, age, and pulmonary involvement. The exceptional association has not been described in the literature. The meticulous clinical and paraclinical approach documented the association of overlapping autoimmune diseases with immunodeficiency, which meets the criteria for allogeneic cell transplantation.

**Disclosure of Interest**

None declared

### P278 Evaluation of patients with localized scleroderma: a single center experience

#### S. N. Taskin, S. Dogantan, A. Paç Kisaarslan, M. H. Poyrazoglu

##### Pediatric Rheumatology, Erciyes University School of Medicine, Kayseri, Turkey

###### **Correspondence:** S. N. Taskin

**Introduction:** Scleroderma is a chronic connective tissue disease that causes widespread microvascular damage and excessive collagen deposition in the skin and internal organs. This disease, which is rare in children, is divided into two main forms as systemic scleroderma and localized scleroderma. Although it is expected in patients with systemic scleroderma, extra-skin involvement can also be seen in localized scleroderma patients.

**Objectives:** It was aimed to evaluate the demographic, clinical, and laboratory data of patients with a diagnosis of localized scleroderma followed in the Pediatric Rheumatology Department of Erciyes University Children's Hospital.

**Methods:** The medical records of 0-18 years old, who were followed up with the diagnosis of Localized Scleroderma in Erciyes University Children's Hospital Pediatric Rheumatology Department between 1 January 2000 and 31 December 2020 were retrospectively reviewed and the demographic, clinical, and laboratory characteristics of the patients were evaluated.

**Results:** The data of 15 cases of localized scleroderma was accessed 3 (20%) of the patients were male, 12 (80%) were female and the average age was 12.3 years. The average age of onset of the disease was 7.1 years, and the time from the onset of the complaints to the diagnosis was approximately 11 months, and the follow-up period after diagnosis was approximately 4.5 years.

Six (40%) of the cases were plaque morphea, 2 (13.3%) pansclerotic scleroderma, 1 (6.6%) generalized morphea, 6 (40%) mixed subtype. All of the mixed subtypes consisted of Linear scleroderma and plaque morphea. 5 (33.3%) patients had 'en coup de sabre'. Lesions were in the face or neck region in 8 (53.3) patients, on the trunk in 7 (46.6%) patients, in the upper extremity in 7 (46.6%) patients, and in the lower extremity in 10 (66.6%) patients.

In addition to localized scleroderma, one (6.6%) patient had puberty precox, one (6.6%) patient had juvenile idiopathic arthritis and Reynaud's phenomenon, one (6.6%) patient had vesicoureteral reflux and renal scar. Systemic lupus erythematosus developed in the follow-up of two (13.2%) patients. One (6.6%) patient also had Crouzon syndrome and vitiligo diagnoses. In addition, growth retardation was detected in the follow-up of one (6.6%) patient.

Joint involvement such as arthritis and arthralgia were detected in 6 (40%) patients, gastrointestinal (gastroesophageal reflux) findings in 1 (6.6%), and neurological findings (headache) in 1 (6.6%) patient. One patient (6.6%) with vesicoureteral reflux also had a renal scar. Ocular, respiratory, and cardiological involvement was not seen in any of the patients.

In the laboratory examinations, no patient was found to have an acute phase reactant (white blood cell, sedimentation, C-reactive protein) elevation in the follow-up. Antinuclear antibody was positive in nine (60%) patients, anti-dsDNA in two (13.3%) patients, and rheumatoid factor in one (6.6%) patient. Thyroid function tests (TSH, T3, T4), thyroid antibodies were normal in all patients.

Ten (66.6%) patients were using disease-modifying antirheumatic drugs (methotrexate or mycophenolate mofetil), and two patients were using biological agents (Etanercept, Tocilizumab). The treatment of five (33.3%) patients was continued with local treatment (Calcipotriol, Mometasone furoate, Pimecrolimus, steroid). Two (13.3%) patients had received ultraviolet phototherapy treat during their follow-up.

**Conclusion:** In localized scleroderma patients, involvement may not be limited to the skin. Patients' complaints about other systems should be carefully evaluated. With detailed evaluation, system involvement can be detected early and the development of sequelae can be prevented with appropriate treatment.

**Disclosure of Interest**

None declared

### P279 Identifying current assessment and treatment strategies in juvenile systemic sclerosis: an international effort to improve patient outcomes

#### N. Vasquez-Canizares^1^, C. Pain^2^, A. Adrovic^3^, J. Anton^4^, J. Chaitow^5^, I. Foeldvari^6^, O. Kasapcopur^7^, M. M. Katsicas^8^, V. Leone^9^, A. Minuti^10^, B. Stevens ^11^, K. Torok^12^, M. Twilt^13^, F. Zulian^14^, S. Li^15^

##### ^1^Pediatrics, Division of Pediatric Rheumatology, Children's Hospital at Montefiore, Albert Einstein College of Medicine, Bronx, United States; ^2^Paediatric Rheumatology, Alder Hey Children's NHS Foundation Trust, Liverpool, United Kingdom; ^3^Pediatric Rheumatology, Cerrahpasa Medical School, Istanbul University, Istanbul, Turkey; ^4^Pediatric Rheumatology, Hospital Sant Joan de Déu. Universitat de Barcelona, Barcelona, Spain; ^5^Rheumatology, Sydney Children's Hospital Network, Randwick and Westmead, Sydney, Australia; ^6^Pediatric Rheumatology, Hamburger Zentrum für Kinder- und Jugendrheumatologie associated to Asklepios Campus der Semmelweis-Universität, Hamburg, Germany; ^7^Pediatric Rheumatology, Istanbul University-Cerrahpasa, Istanbul, Turkey; ^8^Pediatric Rheumatology, Hospital de Pediatria J. P Garrahan, Buenos Aires, Argentina; ^9^Paediatric Rheumatology, Ismett, UPMC, Palermo, Italy; ^10^D. Samuel Gottesman Library Research and Education, Albert Einstein College of Medicine, Bronx; ^11^Pediatrics, Division of Pediatric Rheumatology, Indiana University School of Medicine, Riley Hospital for Children, Indianapolis; ^12^Pediatrics, Division of Pediatric Rheumatology, University of Pittsburgh, UPMC Children’s Hospital of Pittsburgh, Pittsburgh, United States; ^13^Pediatric Rheumatology, Alberta Children’s Hospital, Cumming School of Medicine, University of Calgary, Alberta, Canada; ^14^Woman's and Child's Health, University Hospital of Padova, Padua, Italy; ^15^Pediatrics, Division of Pediatric Rheumatology, Joseph M. Sanzari Children’s Hospital, Hackensack University Medical Center, Hackensack, United States

###### **Correspondence:** N. Vasquez-Canizares

**Introduction:** Juvenile Systemic Sclerosis (jSSc) is a rare autoimmune and fibrosing disease associated with significant morbidity and mortality risk. Data on treatment strategies is limited and primarily based on adult data. Because jSSc has many differences from adult SSc, optimal care requires jSSc treatment studies. A strong international collaboration to develop jSSc consensus tools and definitions to enable comparative effectiveness studies is needed

**Objectives:** To identify currently used assessments and treatments for the different organ manifestations of jSSc. To develop consensus agreement on outcome measures for specific organ manifestations of jSSc and consensus definitions needed for clinical trials. Needed definitions include disease severity levels, and organ specific improvement and worsening criteria for defining response

**Methods:** This is an international collaborative effort between pediatric rheumatologists (PRs) in the Childhood Arthritis and Rheumatology Research Alliance (CARRA) and the Pediatric Research European Society (PReS), led by a core group of 2 CARRA and 2 PReS members (NV, SL, CP, FZ) with collaboration from a consensus working group (WG) of 20-30 CARRA/PReS members to act as country representatives. Steps include a literature review on SSc outcome measures, development of consensus surveys to understand current treatment strategies, and Delphi consensus meetings. A stepwise process focusing on one organ system at a time is intended, initially cardiopulmonary, followed by Raynaud phenomenon, and skin/musculoskeletal systems. Questions will help understand what tools are available, and what methods are routinely used by PRs and considered ideal to assess for organ involvement. To understand current treatment practices and what factors are used by PRs in their stratification of care to help define severity level parameters for different organs, case-based surveys will be developed. Survey data will be collected in REDCap, and will be reviewed along with literature review data in consensus WG meetings to generate consensus outcome measures and definitions for defining treatment response. The study will be supported by experienced collaborators and online tools including Qualtrics, Covidence and Mentimeter to facilitate with survey development, literature review, and consensus meeting process

**Results:** 14 CARRA and PReS members from 9 countries are currently collaborating. An initial survey was generated aimed at understanding the jSSc prevalence in our community, and most commonly used assessments and treatments for cardiopulmonary disease. It will be soon widely distributed amongst CARRA and PReS members and will serve as an opportunity for members, including fellows and junior investigators, to engage in this international collaborative effort at a range of levels

**Conclusion:** A wide gap on defining optimal care for jSSc patients exists worldwide. Developing consensus tools to assess patients and evaluate treatment response is essential for conducting treatment studies to enable generation of jSSc evidence-based recommendations and improve outcomes. An international collaborative effort is needed, expanding beyond the 9 countries and 14 collaborators currently involved

**Disclosure of Interest**

None declared

### P280 Cardiovascular involvement as a clue for the diagnosis of juvenile systemic sclerosis sine scleroderma

#### F. Zulian^1^, G. Lanzoni^1^, A. Meneghel^1^, B. Castaldi^1^, F. Tirelli^1^, E. Zanatta^2^, G. Martini^1^

##### ^1^Department of Woman and Child Health; ^2^Department of Medicine-DIMED, University of Padova, Padova, Italy

###### **Correspondence:** F. Zulian

**Introduction:** Juvenile Systemic Sclerosis (JSSc) is a rare condition in childhood and its variety with no skin involvement, systemic sclerosis *sine scleroderma* (ssJSSc) is anecdotal as only two cases have been reported to date^1,2^. We describe a series of five patients from our Center and compare these seven patients with a cohort of patients with standard JSSc.

**Objectives:** We describe a series of five patients from our Center with the two reported in the literature and compare these seven patients with a cohort of patients with classical JSSc.

**Methods:** Patients with Juvenile Systemic Sclerosis (JSSc) followed at our Center were retrospectively evaluated. For every patient, we collected demographic, clinical, instrumental and laboratory data, autoantibody profile and treatment. According with the proposed ssSSc Criteria^3^, patients with no skin involvement but with all of the following features: 1) Raynaud’s phenomenon or a peripheral vascular equivalent, 2) positive ANA titer (≥1:160), 3) any one of the following: documented intestinal dysmotility, interstitial lung disease or pulmonary arterial hypertension, cardiac or renal involvement typical of scleroderma were defined as having ssJSSc and were compared with those with classical JSSc^4^.

**Results:** Among 52 JSSc patients seen in 20 years, 5 (9.6%) presented with ssJSSc*.* The clinical features of these five patients and of other two, reported in the literature^1,2^, were compared with a group of 32 classical JSSc patients with complete clinical data available. Six patients had cardiovascular involvement as presenting feature, 3 primary myocardiopathy, 3 secondary to pulmonary arterial hypertension (PAH). Two patients (33.3%) died after a quite brief disease course and one rapidly underwent a heart transplantation. ANA was positive in all except one and scleroderma-specific antibodies were present in 4/6 tested patients. Altered EKG and cMRI were present in all tested patients; cEcho parameter ejection fraction (EF) was reduced in 5/7 patients. In comparison with classical JSSc patients, ssJSSc showed a significantly longer diagnostic delay (20.1 vs 8.3 months, p 0.017), higher cardiac involvement (85.7 vs 15.6%, p 0.001) and worse outcome, intended as mortality or end-stage organ failure rate (42.9% vs 6.2%, p<0.001).

Values are numbers (%) or mean (+ SD)

**Conclusion:** To the best of our knowledge, this is the first case series of patients with ssJSSc reported so far. Cardiovascular involvement represents the most important clinical feature of this subtype and still carries a very high morbidity and mortality rate. These preliminary findings and, in particular, the longer diagnostic delay, confirms the crucial role of complete rheumatologic work-up, including the search for autoantibodies and capillaroscopy, in pediatric patients presenting with isolated myocardiopathy or PAH and Raynaud phenomenon.

REFERENCES

1. Navon P et al. *Acta Paediatr*. 1993 Jan;82(1):122-3.

2. Zloof Y, et al. *Pediatrics*. 2020;145(5)

3. Poormoghim H, et al. *Arthritis Rheum* 2000;43:444_51.

4. Zulian F, et al*., Arthritis Rheum.* 2007;57(2):203-12.

**Patient Consent Received**

No

**Disclosure of Interest**

None declared

## e-Poster viewing: Vasculitides

### P281 A case report of refractory Kawasaki disease

#### I. Bona^1^, C. Marino^1^, C. Cavallaro^1^, L. Di Pasquale^1^, M. Genco^1^, A. Alaimo^2^, S. Spoto^2^, F. Centineo^2^, M. A. Garofalo^2^, D. Poli^3^, C. Di Mambro^3^, S. Accomando^4^, M. C. Maggio^4^, C. Alizzi^4^, F. Cardella^4^

##### ^1^School of Specialization in Pediatrics, Università di Palermo; ^2^Pediatric Cardiology Unit, ARNAS Civico Di Cristina, Palermo; ^3^Mediterranean Pediatric Cardiology Center “Bambino Gesù” San Vincenzo Hospital, Taormina; ^4^Department of Pediatrics, ARNAS Civico Di Cristina, Palermo, Italy

###### **Correspondence:** I. Bona

**Introduction:**

**Objectives:**

**Methods:**

**Results:** A four-months old infant was referred for fever for two days and dyspnea. He had bilateral conjunctival hyperemia; bilateral cervical lymphadenopathy; truncal exanthem; perineal erythema; dry cough; normal cardiac, thoracic, and abdominal examination. Nasopharyngeal swab was negative for SARS-CoV-2. His routine analysis showed: Hb 9,9 g/dl; leukocytes 12000/mm3 (neutrophils 60%; lymphocytes 13%); platelets 430000/mm3; CRP 19 mg/dl (normal values < 0,5 mg/dl); ESR 94; Serum Amyloid A 1430 mg/l; ferritin 883 ng/ml. His chest X-ray showed an interstitial pattern. His first echocardiographic evaluation only showed a minimal pericardial effusion, and normal coronary arteries.

The patient received two consecutive intravenous infusions of immunoglobulins (2 g/kg), associated with 2 mg/kg/day methylprednisolone, with consequent apyrexia and reduction of CRP (4,5 mg/dl). He also started 3 mg/kg/day aspirin.

After two days from the second infusion, fever reappeared together with coronary dilatation: diameter was 2,4 mm for LCA (z-score 3,16); 2,4 mm for LAD (z-score 4,49); 2,5 mm for RCA (z-score 4,13). Exams showed CRP 6,57 mg/dl; leukocytes 62860/mm3 (73% neutrophils); platelets 1532000/mm3. The child then received Intravenous pulses of 30 mg/kg/day methylprednisolone in three consecutive days, and started 4 mg/kg/day anakinra.

The pulses were followed by apyrexia, with CRP 6,54 mg/dl. Four days later CRP was 8,96 mg/dl, so anakinra was interrupted, and 5 mg/kg infliximab was started.

Echocardiogram showed rapidly evolving giant coronary aneurysms: diameter was 6 mm for LCA (z-score 15,79); 6,5 mm for LAD (z-score 16,41); 5 mm for RCA (z-score 11,72).

Due to the rapid evolution of aneurysms and an elevated z-score (> 10), 1 mg/kg/dose enoxaparin was started, according to the Kawasaki disease guidelines of the American Heart Association (AHA) (*Circulation*, 2017). The patient also started ECG, troponin and BNP monitoring. Three days later, the coronary arteries showed further dilatation. Seven days after infliximab infusion, RCA developed a “rosary bead - like” pattern.

There was a gradual improvement of general clinical conditions with reduction of inflammatory markers (negative CRP, ESR 22, leukocytes 28580/mm3, platelets 800000/mm3 after ten days from infliximab infusion), so steroid tapering was started. Because of an increased thromboembolic risk (due to thrombocytosis and to the accelerated blood flow in stenotic coronary traits), clopidogrel 0,5 mg/kg/day was added, according to AHA guidelines.

Thirty days after symptom onset, also LDA developed a “rosary bead - like” pattern. CRP was 1,27 mg/dl and ESR was 37. The patient was then transferred to Pediatric Cardiac Surgery, started 5 mg/kg/day cyclosporin and, after further coronary dilatation (LDA 11 mm; RCA 9 mm), 3 mg/kg/day propranolol and warfarin. A CT angiogram confirmed the aneurysms, and excluded any aortic dilatations. Epiaortic vessels were spared. An increase of cyclosporin dose to 8 mg/kg/day was followed by stabilization of coronary aneurysms.

**Conclusion:** The extreme refractariety of coronary disease, despite aggressive therapy and biologics, is rather clear. The rapid evolution could be explained, though only in part, by a few risk factors such as age below 12 months and elevated CRP. These factors are well known, even though they are not mentioned in guidelines.

Written informed consent for publication was obtained.

**Patient Consent Received**

Yes

**Disclosure of Interest**

None declared

### P282 Chronic polyarthritis in an adolescent with cutaneous polyarteritis nodosa

#### T. Castro^1,2^, L. Malat^2^, D. Mikhaela^2^, P. Vara^2^

##### ^1^Pediatrics, São Camilo Hospital; ^2^Universidade Cidade de São Paulo - Unicid, São Paulo, Brazil

###### **Correspondence:** L. Malat

**Introduction:** Polyarteritis nodosa (PAN) is a necrotizing vasculitis of medium/small arteries. It is a rare condition, especially in the pediatric age group. Cutaneous PAN (cPAN) is recognized as a separate entity. It is characterized by disease affecting the skin with no major organ system involvement.

**Objectives:** Chronic polyarthritis is much less observed and can simulate juvenile idiopathic arthritis, which can delay diagnosis, as was the case with this patient.

**Methods:** We report a 14-year-old boy, who presented to us with polyarthritis and myalgia as a first manifestation of cPAN.

**Results:** This 14-year-old male adolescent presented with a history of arthritis in right shoulder, ankles, knees, elbows, fingers and toes, associated with myalgia, with partial improvement after the use of non-hormonal anti-inflammatory drugs. Two months later, he was diagnosed with juvenile idiopathic arthritis. Corticosteroids were prescribed in a dose of 20 mg/day, methotrexate 7.5 mg/week and folic acid. There was no clinical improvement. At this time, he start with fever and the appearance of a painful erythematous lesions in the lower limbs and arms, difficulty in walking, which were progressively getting worse, anorexia and weight loss during this period. He also had a history of oropharyngeal infection prior to beginning of these symptoms. At that time, he was treated with azithromycin for 5 days. As there was no improvement in his clinical condition, he sought the Emergency Room where he was admitted for investigation 3 months after the beginning of his symptoms. On physical examination, we found arthritis in the elbows, knees, ankles and joints of the hands, maculopapular rash in the ankles, muscle atrophy in the arms and legs, palpable and painful nodules on the right leg and right foot.

Several laboratory tests were requested. Blood investigations showed anemia, leucocytosis, elevated CRP, ESR and ASLO titres, with normal liver and kidney functions, and normal urine 1. Syphilis, HIV, serology for *Mycoplasma* pneumoniae, leishmaniasis, *Paracoccidioides brasiliensis*, Epstein-Barr virus, cytomegalovirus, Zika and Chikungunya viruses, dengue, and parvovirus B19 were negative. HBsAg, hep c were negative. CRP and serology was negative for Coronavirus 19 (Covid-19). Chest X-ray, echocardiogram, and ultrasound of the abdomen were normal. Lupus anticoagulant, and FR were negative. Other autoantibodies were negative. p-ANCA and c-ANCA were negative. Myelogram with normal immunophenotyping was found. A skin biopsy was suggestive of PAN showing perivascular lymphomononuclear inflammatory infiltrate, sometimes permeating the vascular wall. The presence of eosinophils and neutrophils with outbreaks of leukocytoclasia was noted, an absence of malignancy was also shown.

With infectious, hematological and oncological causes aside, pulse therapy with methylprednisolone was started. After the first infusion, a significant improvement in myalgia and arthritis was observed, in addition to the disappearance of febrile peaks. Then, he started a monthly pulse therapy with cyclophosphamide and methylprednisolone. Four months after start this therapy, we observed improvement of skin lesions and in laboratory exams.

**Conclusion:** Cutaneous PAN is a rare disease, especially in the pediatric age group. Its clinical manifestations are quite varied, making early diagnosis difficult. Joint involvement, when it occurs, is characterized by an acute and oligoarticular pattern in the knees and ankles. Chronic polyarthritis is much less observed and can simulate juvenile idiopathic arthritis, which can delay diagnosis, as was the case with this patient. We must consider the diagnosis of PAN in those patients with chronic polyarthritis, associated with cutaneous vasculitic manifestations and increased ASLO.

**Patient Consent Received**

Yes

**Disclosure of Interest**

None declared

### P283 Kawasaki disease. Importance of early diagnosis and evidence-based treatment. Case report

#### M. I. De La Cera Rodríguez, N. L. De la Rosa Encarnacion, H. F. Menchaca Aguayo, E. R. Mercedes Perez, P. P. Ramos Tiñini, S. Rodriguez Aguayo, E. Faugier Fuentes

##### Rheumatology, Hospital Infantiil de México Federico Gómez, Mexico, Mexico

###### **Correspondence:** M. I. De La Cera Rodríguez

**Introduction:** Kawasaki disease is an acute vasculitis of childhood that carries a risk of developing coronary artery aneurysms in up to 25% of cases without treatment. Prompt treatment with IVIG and additional therapies can lower risk to less than 4%.

In the past decade, the bibliography reports the contraindication of IVIG administration after the acute phase of disease. Currently, the therapeutic goal of treating Kawasaki disease with IVIG even after day 10 of evolution, is to reduce inflammation limit arterial damage, and prevent thrombosis.

**Objectives:** To describe the presentation of a clinical case that describes the complications associated with the previous indication for not administering IVIG after the acute phase of the disease.

**Methods:** Case Description: a 3-year-old male sent to a third-level hospital for a 30-day fever, meets the criteria for incomplete Kawasaki disease, so an echochardiogram was performed to corroborate the presence of a 6 mm aneurysm in the right coronary artery. Because it is in the subacute phase of the disease, IVIG administration is deferred. During his followup, progression to a giant saccular aneurysm was documented and in the last control of myocardial perfusion tomography, data of mild ischemia in the affected artery territory was reported; being a candidate for management with interventional hemodynamics.

**Results:** Not administering IVIG and additional therapies, even after the acute phase of the disease, entails an increased risk of aneurysm development and their progression, which determines the need for invasive revascularization procedures.

**Conclusion:** 10 years ago IVIG was indicated only in the acute phase of the disease, currently the benefit of its administration is documented, as well as additional therapies even after this time has elapsed to improve prognosis and function. Because of the development of coronary disease in the acute and subacute phase of the disease, the administration of IVIG is currently indicated during this period and not limit its indication to the first ten days of the disease, since this situation conditions the presence of coronary lesions and/or progression thereof.

**Patient Consent Received**

No

**Disclosure of Interest**

None declared

### P284 Kawasaki disease shock syndrome (KDSS)

#### N. L. De La Rosa Encarnación, M. I. De la Cera Rodriguez, H. F. Menchaca Aguayo, E. R. Mercedes Perez, P. P. Ramos Tiñini, E. Faugier Fuentes, S. Rodriguez Aguayo

##### Reumatología pediátrica, Hospital Infantil de México Federico Gómez, Ciudad Mexico, Mexico

###### **Correspondence:** N. L. De La Rosa Encarnación

**Introduction:** Kawasaki disease (KD) is a systemic vasculitis, it is known as the main cause of heart disease acquired in childhood. Heart disease can cause coronary artery occlusion, ischemia, and rupture, even disability or death. Shock is not a common presentation of the disease; it rarely occurs in the acute phase. In recent years, cases of patients with KDSS have been reported, which is characterized by presenting hemodynamic instability during the acute phase of the disease. Despite the continuous advances underdiagnosed by pediatricians, due to erroneous diagnoses.

**Objectives:** Describe a case of shock syndrome due to Kawasaki disease (KDSS)

**Methods:** We present the case of a 4-month-old boy who presented KD complicated by shock syndrome (KDSS), treated with intravenous immunoglobulins (IVIG) and methylprednisolone pulses.

**Results:** 4-month-old male. Healthy preview. Clinical picture: persistent fever for 20 days (40 ° C), changes in the oral mucosa with cheilitis, conjunctival injection, maculopapular rash, edema of the hands and feet, cervical adenopathy> 1.5cm, for which he was hospitalized outside the institution, receiving multiple treatments of antibiotics for a week. Referred to our center for treatment of long-standing fever. At his evaluation drowsy, irritable, with shock data (tachycardia of 220 beats per minute, generalized paleness, diminished peripheral pulses and capillary filling 3 seconds), seizure. Kawasaki disease shock syndrome was integrated, he was admitted to the emergency room where he required oxygen, loads with crystalloids and aminergic support with dobutamine, he was transferred to intensive care (PICU). It has a HARADA score of 5 points. Laboratories: leukocytosis, thrombocytosis, anemia, hyponatremia, hypoalbuminemia. High acute phase reactants (CRP and ESR). Electrocardiogram with sinus tachycardia, an echocardiogram was performed with aneurysm of the left coronary 4.4x5.3 mm, right coronary 4.8x4.6 mm and 2.5x3.2 mm anterior descending coronary artery. Renal and liver function without alteration, negative blood and CSF cultures. Treatment: gamma globulin (2gkgd), methylprednisolone (20mgkgd) for 3 days, acetylsalicylic acid (10mgkgd) and enoxaparin (1mgkgd). Cefepime during his hospital stay. He was discharged with a decrease in the aneurysms and without neurological sequelae, with an antiplatelet dose of ASA (3mgkgd) due to clinical improvement after 7 days of hospitalization.

**Conclusion:** We report a case of Kawasaki disease shock syndrome (KDSS). This entity is characterized by tachycardia, hypotension, and signs of poor perfusion. The relevant clinical finding in this case was hemodynamic instability, as well as the typical KD clinical presentation. Shock, a rare but serious complication of Kawasaki disease, should be considered as a differential diagnosis of toxic shock. Timely diagnosis is imperative to initiate adequate treatment, thus reducing the risk of developing coronary artery aneurysm. We emphasize that fever for more than 5 days with no apparent cause can be suggestive of Kawasaki disease and according to the 2017 AHA recommendations, it is imperative to perform an echocardiographic study, a situation that can avoid sequelae such as KDSS in this patient.

**Disclosure of Interest**

None declared

### P285 Is there an increased cardiovascular risk in children with vascular Behçet’s disease?

#### S. Demir^1^, A. Düzova^2^, O. Çimen^1^, E. Sağ^1^, B. Oğuz^3^, T. Karagöz^4^, S. Ozen^1^, Y. Bilginer^1^

##### ^1^Department of Pediatric Rheumatology; ^2^Department of Pediatric Nephrology; ^3^Department of Radiology; ^4^Department of Pediatric Cardiology, Hacettepe University School of Medicine, Ankara, Turkey

###### **Correspondence:** S. Demir

**Introduction:** Behçet’s disease (BD) is a polygenic multisystemic autoinflammatory disorder and vascular involvement is one of the major causes of morbidity and mortality of the disease.

**Objectives:** We aimed to define whether vascular involvement of pediatric BD is a risk factor for hypertension and cardiovascular dysmorphology.

**Methods:** Thirty-one BD patients who followed at pediatric rheumatology clinic, were enrolled to the study. The pediatric patients (<16 years of age at disease onset and diagnosis) were classified as having BD according to the Pediatric Behçet’s Disease (PEDBD) classification criteria. Demographic data, clinical manifestations, laboratory and radiological findings and outcomes were documented from patient charts. Patients with other known risk factors for cardiovascular disease as obesity and hypertension were excluded. During the same week, carotid intima-media thickness (cIMT) measurement and echocardiography and 24-hour ambulatory blood pressure monitoring (ABPM) were performed. Physical examination and blood tests were also performed on the same day with cIMT.

**Results:** Thirty-one children with pediatric BD (16 female, 51.6%; F/M: 1.06) were enrolled in the study. Based on the cumulative disease characteristics; oral ulcer was the most common clinical finding (100%), followed by skin involvement (78%, n=25), arthritis (56%, n=18), genital ulcers (47%, n=15), ocular involvement (28%, n=9) had and vascular involvement (18%, n=6). We grouped patients into two groups as patients with and without vascular involvement. The mean age at disease onset and at the time of BD diagnosis was 8.79 ± 4.23 years, and 11.62 ± 3.22 years, respectively. The median follow-up duration was not statistically different between two groups (59.63 months (IQR: 58.38-63.57) vs 40.28 months (IQR: 11.30-75.47)). All patients underwent cIMT measurement, echocardiography and 24-hour ABPM. The mean value of right cIMT, left cIMT and echocardiograpy measurements were not different between two groups. Although the prevalence of abnormal ABPM, non-dipping, and ambulatory hypertension was higher in patients with vascular involvement, they did not reach to statistical significance (Table).

**Table:** 

	Patients with vascular involvement (n=6)	Patients without vascular involvement (n=25)	p-value
**TAPSE z score**	-2.25 ± 1.62	-0.80 ± 1.69	0.501
**LVMass z score**	-0.82 ± 0.42	-0.79 ± 2.12	0.255
**IVSD z score**	1.07 ± 0.48	1.68 ± 0.95	0.111
**LVIDD z score**	-0.92 ± 1.23	-0.45 ± 1.59	0.393
**LVPWTD z score**	0.51 ± 0.76	0.79 ± 0.87	0.452
**LVIDS z score**	-0.84 ± 1.14	-0.65 ± 0.95	0.851
**cIMT mean (mm)**	0.43 ± 0.05	0.42 ± 0.07	0.841
**Abnormal ABPM,** n (%)	3 (50.0%)	7 (30.4%)	0.633
**ABPM-hypertension,** n (%)	2 (33.3%)	3 (13.0%)	0.269

Results for continuous variables are presented as mean ± standard deviation LVMass: left ventricular mass, TAPSE: tricuspid annular plane systolic excursion, LVIDD: left ventricular internal diameter end diastole, LVIDS: left ventricular internal diameter end-systole, IVSD: interventricular septal end-diastole, LVPWTD: left ventricle posterior wall thickness in diastole cIMT: carotid intima-media thickness, ABPM: ambulatory blood pressure monitoring

**Conclusion:** The cIMT and cardiac morphology were similar between BD patients with and without vascular involvement. We observed that vascular BD patients had more abnormal ABPM though it did not reach statistical significance. Long-term follow-up studies in larger series may clarify the value of ABPM.

**Disclosure of Interest**

None declared

### P286 Distribution of adverse prognostic factors in adult onset and childhood onset IgA vasculitis – nephritis, our experience from a tertiary care centre

#### S. A. Ganu^1^, P. Chickermane^1^, S. N. V.^2^, S. Balan^1^

##### ^1^Department of Clinical Immunology and Rheumatology; ^2^Department of Pathology, Amrita Institute of Medical Sciences, Kochi, India

###### **Correspondence:** S. A. Ganu

**Introduction:** A multitude of studies have described adverse risk factors concerning various aspects of renal involvement like severity & prognosis in IgA vasculitis nephritis. The adult-onset IgA vasculitis– nephritis (a-IgAVN) and childhood onset IgA vasculitis nephritis (c-IgAVN) have different severities and prognosis. Therefore, it is likely that these two groups might differ in prevalence of these risk factors.

**Objectives:** To describe the comparative distribution of risk factors associated with renal disease in a-IgAVN and c-IgAVN.

**Methods:** Retrospective chart review of all patients visiting our hospital between May 2013 and May 2021 was done. Those with a diagnosis of IgA vasculitis or Henoch Schoenlein Purpura were further screened for IgA vasculitis by using EULAR/PReS/PRINTO (2010) classification criteria for IgA vasculitis. Among the selected patients, those having renal involvement were selected for analysis.

**Results:** A total 53 patients had a IgAVN with a median disease duration of 63 (43-87) month and median follow up duration of 14 (3-24) month. These were further classified into c-IgAVN (n= 29) and a-IgAVN (n=24).

We did not have any patient with disease onset before 4 year of age, 23 (79%) of c-IgAVN had onset after 10 years of age and in 2 patients (8.3% of a-IgAVN group) the disease started after 65 years of age. The proportion of patients in whom renal involvement started within 3 months of first symptom was same in both groups.

We noticed that the c-IgAVN group had a higher percentage of patients with GI involvement than the a-IgAVN group [25 (86.2%) vs 14(58.3%), p = 0.048]. Most of the GI involvement was in the form of abdominal pain alone. Prevalence of GI bleed was not different in two groups [11 (37.9%) vs 5 (20.8%), p 0.177]. Adults had a higher prevalence of central distribution of skin rash [1 (3.4%) vs 10 (41.7%), p 0.002] and bullous skin involvement [0 vs 3 (12.5%)]. A notable number of patients in each group had a persistent purpura [2 (6.9) vs 3 (12.5), p 0.824].

The renal insufficiency as a presenting manifestation was found in a significantly higher proportion of patients with a-IgAVN [1 (3.4%) vs 6 (25%), p 0.058]. The percentage of various types of the clinical renal involvement at presentation is shown in the following table.

**Table:** 

Type of clinical renal involvement	c-IgAVN	a-IgAVN
Haematuria, n(%)	4 (13.8)	2 (8.3)
Mild proteinuria +/- haematuria, n(%)	4 (13.8)	5 (20.8)
Acute nephritic syndrome, n(%)	9 (31)	9 (37.5)
Nephrotic syndrome, n(%)	10 (34.5)	6 (25)
Mixed nephrotic- nephritic syndrome, n(%)	2 (6.9)	2 (8.3)

Renal biopsy was performed in 8 patients in each group (c-IgAVN and a-IgAVN). Five patients in each group had undergone an upfront renal biopsy before commencing immunosuppressive treatment, while in 3 patients it was done after failure of initial treatment. We found that ISKDC class 3a and 3b were the most common ISKDC classes found in both groups. There was no significant difference in individual scores (MEST-C scoring) for mesangial proliferation, endothelial proliferation, segmental sclerosis, tubular atrophy, or crescents between the two groups.

We did not have any patient with CNS involvement or angioedema. There was no significant difference in other traditional laboratory markers.

**Conclusion:** In our study, we found that among all the prognostic factors analysed, most of the risk factor like GI bleed were present in both groups; however, the central distribution of purpuric lesions, bullous skin lesions and renal insufficiency at presentation were more prevalent in a-IgAVN patients as compared to c-IgAVN patients. These findings concur with more aggressive nature of adult-onset IgA vasculitis-nephritis.

**Patient Consent Received**

No

**Disclosure of Interest**

None declared

### P287 Lupus panniculitis in a 9 year-old girl: a case report

#### M. F. Gicchino, F. Di Domenico, A. Barlabà, M. Bartiromo, E. Miraglia del Giudice, A. N. Olivieri

##### Department of Woman, Child and General and Specialistic Surgery, University of the Study of Campania Luigi Vanvitelli, Naples, Italy

###### **Correspondence:** M. F. Gicchino

**Introduction:** Lupus panniculitis (LP) is a rare variant of cutaneous lupus erythematosus. LP commonly presents in the third-to-sixth decades of life, with female predilection. The most common cutaneous manifestations are tender, painful, erythematous subcutaneous indurated nodules or plaques on fatty body areas. Profound lipoatrophy often occurs, potentially leading to severe disfigurement. While LP is frequently isolated, coexistence with discoid lupus erythematosus (DLE) or systemic lupus erythematosus (SLE) may occur. In children diagnosed with LP, the risk of progression to SLE is unknown. Diagnosis is often delayed, increasing the risk for sequalae, such as atrophy and calcinosis. Differential diagnosis for LP includes many diseases such as erythema nodosum, pancreatic panniculitis, morphea profunda, cold-panniculitis and subcutaneous panniculitis-like T-cell lymphoma (SPTCL), which may be clinically identical with LP. LP is commonly treated with antimalarial drugs, immunosuppressants and corticosteroids.

**Objectives:** To report a rare case of a 9 year old girl diagnosed with LP.

**Methods:** A nine year old girl came to our department because of painful erythematosus nodules in her left leg from six months. It was not referred history of fever or trauma. Patient examination revealed erythematous, nodular lesion with microulcerations affecting the left leg. The left limb was flexed and extra-rotated. Patient presented intense pain and lameness on walking. The rest of clinical examination was unremarkable. Complete blood count, inflammatory parameters, liver, kidney function, iron levels was in the norm. Both Antinuclear antibodies and ENA were negative. Virological screening (including Sars-Cov2) was negative. Quantiferon test was negative. Urinalysis was in the norm. Faecal calprotectin was negative. Both heart and abdomen ultrasound were in the norm. Patient underwent to magnetic resonance of left leg and skin biopsy of the lesion.

**Results:** MRI revealed: signal hypertensity of skin tissue and subcutaneous adipose tissue in the left thigh. A further area of ​​hyperintensity in correspondence with the cutaneous plane at the level of the popliteal fossa and the subcutaneous tissue and two areas of signal hyperintensity affecting the semitendinosus and sartorius muscle were also detected. Skin biopsy showed: superficial and deep perivascular lymphocytic dermatitis (prevalence of CD3 + T lymphocytes with predominance of CD4 +) with lobular panniculitic involvement prevailing at the dermo-hypodermic interface.

**Conclusion:** According to clinical features, laboratory, radiological and histopathological findings Lupus Panniculitis was diagnosed and treatment with prednisone and methotrexate was started. At a one month follow up visit patient was in good general conditions, she was able to walk. The erythematous nodules were in regression. Lupus panniculitis is rare and difficult to diagnose, particularly in patients without SLE or DLE at presentation. An early diagnosis is very important in order to start an adequate treatment avoiding complications, such as pain and irreversible atrophy.

**Patient Consent Received**

Yes

**Disclosure of Interest**

None declared

### P288 Ischemic stroke as an initial presentation of Takayasu arteritis in a 16 year old

#### M. Hafeez

##### Rheumatology, University Hospital of Leicester, Leicester, United Kingdom

**Introduction:** Takayasu Arteritis is a panarteritis affecting Aorta and its main branches(1). It affects mainly females in second to thrid decade of life(2). Neurological manifestation of TA are variable including stroke, TIA and visual disturbances resulting from either stenosis or thromboembolism(3).

**Objectives:** We present a case of 16 year old female admitted with 1 day history of left sided weakness, sensory loss,dysphagia and speech disturbance. She was reviewed by stroke team with resolved speech and swallowing symptoms but persistent left sided weakness.

On examination blood pressure was 105/75mmHg in right arm and 109/72mmHg in left arm. Pulses were regular bilaterally.

MRI head was done which showed high T2 signal intensity in the territory of the right MCA involving the Caudate and Puteman which demonstrates diffusion restriction in keeping with acute infarction.

Initial treatment was with aspirin as patient was outside thrombolysis window, followed by dual anti-platelet therapy as evidence of occluded left common carotid artery and nearly occluded right common carotid and internal carotid artery on Carotid Ultrasound.

**Methods:** Further investigation with CT angiogram aorta and carotid revealed evidence of large vessel vasculitis involving the aortic arch, its branches and descending thoracic aorta,which would support a diagnosis of Takayasu's arteritis. No evidence of truncal end-organ ischaemia or other sites of arterial occlusion apart from common carotid arteries was seen.

**Results:** Multidisciplinary team approach was adopted.

Upon discussion no surgical intervention was advised by vascular surgery.

Clopidogrel was advised to stopped after 4 weeks and aspirin to continue long term.

Rheumatology team was involved and patient was started on IV methylprednisolone for 3 days followed by weaning dose of oral prednisolone with view of starting on Rituximab as outpatient.

Physiotherapy input was valuable and helped her with improving her mobility.

**Conclusion:**

Takayasu arteritis can have stroke as its rare initial manifestation in young people(2).

In dealing with young people, neurological symptoms should be taken seriously and acted promptly upon, followed by urgent investigation to rule out any underlying serious problem.

Diagnosis of stroke in young patient should raise concern of ruling of Takayasu arteritis as a potential cause.

References

1. Di Santo M, Stelmaszewski EV, Villa A. Takayasu arteritis in paediatrics. Cardiol Young. 2018;28(3):354–61.

2. Shah B, Chhetri R. Malignant ischemic stroke in a young female: A rare primary manifestation of Takayasu arteritis. Case Rep Neurol Med. 2019;2019:7942825.

3. Kim HJ, Suh DC, Kim JK, Kim SJ, Lee JH, Choi CG, et al. Correlation of neurological manifestations of Takayasu’s arteritis with cerebral angiographic findings. Clin Imaging. 2005;29(2):79–85.

**Patient Consent Received**

Yes

**Disclosure of Interest**

None declared

### P289 The role of single nucleotide polymorphisms of genes HMGB1 and ager in the susceptibility and clinical features of patients with IgA vasculitis

#### M. Held^1^, M. Batnozic Varga^2^, M. Sestan^1^, M. Sapina^3^, N. Kifer^1^, D. Grguric^1^, K. Crkvenac Gornik^4^, M. Frkovic^1^, N. Arvaj^5^, J. Wagner^5^, M. Jelusic^1^

##### ^1^University Hospital Centre Zagreb, Department of Pediatrics, University of Zagreb School of Medicine, Zagreb; ^2^University Hospital Centre Osijek, Department of Pediatrics, Josip Juraj Strossmayer University of Osijek, Faculty of Medicine; ^3^University Hospital Centre Osijek, Department of Pediatrics, Josip Juraj Strossmayer University of Osijek, Faculty of Medicine, Faculty of Dental Medicine and Health, Osijek; ^4^University Hospital Centre Zagreb, Department of laboratory diagnostics, University of Zagreb School of Medicine, Zagreb; ^5^Department of Biology and Medical Genetics, Faculty of Medicine, Josip Juraj Strossmayer University of Osijek, Osijek, Croatia

###### **Correspondence:** M. Held

**Introduction:** The pathogenesis of IgA vasculitis (IgAV) is complex and still insufficiently elucidated. It is a multifactorial disease in the development of which, in addition to numerous environmental factors, the genetic background also plays an important role. Previous genome-wide association study studies have established an association between IgAV susceptibility and the HLA class II genes, although many small studies have indicated the importance of variants in various non-HLA genes in the manifestation of different disease phenotypes.

**Objectives:** The aim of this research was to investigate single nucleotide polymorphisms (SNPs) of genes *HMGB1* and *AGER* encoding for high mobility group box-1 (HMGB1) and receptor for advanced glycation endproducts (RAGE), both acting as mediators of inflammation, in the susceptibility and clinical features of patients with IgAV.

**Methods:** Genomic DNA was extracted from whole blood samples after which the HMGB1 and RAGE gene polymorphisms were genotyped using a real-time polymerase chain reaction. The presence and frequency of polymorphisms in HMGB1 (rs2249825, rs1045411, rs1060348, rs1412125 and rs41369348) and RAGE (rs1800625, rs1800624, rs2070600 and rs3134940) were determined. Clinical data were collected from database with systematic analysis of patients with IgAV in Croatian population from two Croatian University Centers for pediatric rheumatology and nephrology care.

**Results:** The research included 81 pediatric IgAV patients, of whom 45 were boys and 36 girls, as well as 150 age- and sex-matched healthy controls without any history of autoimmune disease. The median (range) age of IgAV patients was 6.25 (4.60-8.20) years, and among them 71.6% had joint involvement, 29.62% had gastrointestinal manifestations, while 27.16% patients developed nephritis. The purpuric rash which extended from lower extremities to the trunk, upper extremities and face (generalized rash) was present in 43.20% of patients and 27.16% had at least one relapse. Among the analyzed polymorphisms, only in the rs1412125 there was a deviation from the Hardy Weinberger equilibrium. There was no statistically significant association of the analyzed polymorphisms with the IgAV susceptibility, compared to healthy controls. However, the two polymorphisms proved to be linked with a well-defined clinical phenotype. Polymorphism rs2070600 was significantly related with the development of nephritis in IgAV, while rs1412125 was associated with gastrointestinal involvement. The IgAV patients carrying the T allele (rs2070600) of the *AGER* had significantly increased risk of nephritis development compared with the IgAV patients with homozygous CC genotype in dominant (OR 4.05, CI 1.09-15.03, p = 0.037) and additive genetic models (OR 3.95, CI 1.16-13.47, p = 0.049). The minor C allele (rs1412125) of the *HMGB1* was found to significantly increase the risk of gastrointestinal involvement in overdominant model with an allelic odd ratio of 2.78 (CI 1.04-7.43, p = 0.04).

**Conclusion:** Although neither of analyzed HMGB1 and RAGE polymorphisms was not associated with IgAV susceptibility, our results indicated that these polymorphisms may be involved in the pathogenesis of IgAV with possible effect on different disease phenotypes.

SUPPORT: Croatian Science Foundation project IP-2019-04-8822

**Trial registration identifying number:**

**Patient Consent Received**

No

**Disclosure of Interest**

None declared

### P290

**Withdrawn**

### P291 Cogan’s syndrome: report of two pediatric cases

#### M. Kasap Cuceoglu^1^, O. Basaran^1^, E. D. Batu^1^, U. Kaya Akca^1^, E. Atalay^1^, S. Sener^1^, Z. Balik^1^, R. Gocmen^2^, S. Kadayifcilar^3^, M. U. Akyol^4^, Y. Bilginer^1^, S. Ozen^1^

##### ^1^Pediatric Rheumatology; ^2^Department of Radiology, Section of Neuroradiology; ^3^Department of Ophthalmology; ^4^Department of Otolaryngology, Hacettepe University, Ankara, Turkey

###### **Correspondence:** M. Kasap Cuceoglu

**Introduction:** Cogan Syndrome is defined as a chronic inflammatory disease characterized by interstitial keratitis associated with audio-vestibular symptoms. It is characterized by a sudden onset of vertigo, tinnitus, and usually rapid development of bilateral sensorineural hearing loss. The etiology is still unknown. Diagnosis is often missed or delayed because it is a rare disease, and there is no specific diagnostic test. Early diagnosis and appropriate management improve the prognosis. Steroid treatment should begin immediately to prevent irreversible lesions of the eyes and inner ear.

**Objectives:** Cogan Syndrome is a rare inflammatory disease characterized by interstitial keratitis or uveitis, vestibular impairment, and progressive hearing loss, commonly bilateral. Although glucocorticoids are fundamental treatment options, in most cases, hearing loss gradually worsens. Herein, we report two pediatric cases with Cogan syndrome.

**Methods:** Case Report

**Results:** Case 1, A previously healthy 14-year-old boy presented with acute bilateral red and painful eyes. He had ataxia, vertigo, vomiting, and tinnitus without any fever. He was diagnosed with allergic conjunctivitis and started steroid eye drops. The fourth day after the presentation, otolaryngological evaluation was requested because of the consistent severe vomiting, tinnitus, and vertigo. Case 2, A 14-year-girl presented in the ophthalmology department with acute bilateral red and painful eyes. There were no oral aphthae, morning stiffness, dry mouth, dry eyes, and joint swelling in her past medical history. First patient had a cochlear implant, the hearing of the other patient improved with medical treatment.

**Conclusion:** Cogan's syndrome should be in our differential when a young patient presents with sudden onset hearing loss. The major morbidity of the disease is hearing loss, which will become irreversible if treatment is delayed. Early diagnosis and treatment can prevent irreversible hearing loss.

**References**

1. Cogan dg. Syndrome of nonsyphilitic interstitial keratitis and vestibuloauditory symptoms. Arch ophthalmol. 1945;33(2):144-149.

2. Pagnini I, Zannin ME, Vittadello F, et al. Clinical features and outcome of Cogan syndrome. J Pediatr. 2012;160(2):303-307.e1.

3. Bacciu A, Pasanisi E, Di Lella F, Guida M, Bacciu S, Vincenti V. Cochlear implantation in patients with Cogan syndrome: long-term results. Eur Arch otorhinolaryngology Off J Eur Fed Oto-Rhino-Laryngological Soc Affil with Ger Soc Oto-Rhino-Laryngology - Head Neck Surg. 2015;272(11):3201-3207.

**Patient Consent Received**

Yes

**Disclosure of Interest**

None declared

### P292 Serum biomarker profiling among KAWAKINRA study participants identifies different response groups to IL-1Ra treatment

#### C. Kessel^1^, I. Koné-Paut^2^, S. Tellier^3^, A. Belot^4^, L. Rossi-Semerano^5^, P. Dusser^6^, I. Marie^6^, N. Boukhedouni ^7^, C. Piedvache^8^, D. Foell^1^

##### ^1^Pediatric Rheumatology & Immunology, University Children's Hospital Muenster, Muenster, Germany; ^2^Division of Pediatric Rheumatology and CEREMAIA, Bicêtre Hospital, APHP, University of Paris sud Saclay, Le Kremlin-Bicêtre; ^3^Department of Pediatrics, Divisions of Nephrology, Rheumatology and Internal Medicine, University of Toulouse, Toulouse; ^4^Departments of Pediatrics, Division of Rheumatology, Dermatology and Nephrology, University of Lyon, Lyon; ^5^Division of Pediatric Rheumatology and CEREMAIA,, Bicêtre Hospital, APHP, University of Paris sud Saclay, Le Kremlin-Bicêtre; ^6^Division of Pediatric Rheumatology and CEREMAIA, Bicêtre Hospital, APHP, University of Paris sud Saclay, Le Kremlin-Bicêtre; ^7^APHP, Paris Saclay, Clinical Research Unit Paris-Sud, Bicêtre Hospital; ^8^APHP, Clinical Research Unit Paris-Sud, Bicêtre Hospital, Le Kremlin-Bicêtre, France

###### **Correspondence:** C. Kessel

**Introduction:** Kawasaki disease (KD) is an acute vasculitis of unknown etiology that affects small- and medium-sized arteries of infants and children. It is the main cause of acquired heart disease during childhood in developed countries. Studies in preclinical models and on human material suggest IL-1 signaling to have a critical role in KD pathophysiology.

**Objectives:** To monitor selected serum biomarker levels in course of IL-1 blockade in IVIG-resistant Kawasaki disease (KD) patients enrolled in the KAWAKINRA trial^1^.

**Methods:** Sixteen KAWAKINRA patients with KD that failed to respond to one or more courses of IVIG received daily subcutaneous injections of anakinra for a total treatment duration of 14 days^1^. Serum samples for biomarker analyses were acquired during screening visit prior to first anakinra treatment as well as on days 3 and 14 in course of the study. Biomarker levels in serum or cell culture supernatant were assessed by multiplexed bead array assay. S100A12 serum levels were quantified by in-house ELISA.

**Results:** Levels of most analyzed inflammatory serum markers predominantly linked to either innate (IL-1b, IL-6, IL-10, IL-18, TNFa, MCP-2, S100A12) or adaptive (IFNg, IFNg-signaling: CXCL9, CXCL10; IL-17A) immune mechanisms declined significantly in course of anakinra treatment but remained elevated over healthy pediatric controls. Similarly, serum concentrations of markers linked to immune cell or endothelial cell activation (sICAM-1, soluble VCAM-1, LRG1) were reduced upon IL-1 blockade. Hierarchical clustering analysis of all quantified serum biomarkers detected prior to anakinra treatment revealed the cohort to separate into two distinct clusters, which was predominantly driven by differing levels of IL-1b, endogenous IL-1Ra and LRG1. Following 3 days of anakinra treatment, we still observed a distinct clustering of patients, primarily driven by LRG1 serum levels. Increased concentrations associated with the necessity to adjust anakinra dosage. Following 14 days of daily anakinra injections a clustering of patients according to significant differences in serum biomarker levels was no longer evident. Throughout, LRG1 blood levels were tightly associated with circulating IL-1b and inversely correlated to hemoglobin concentrations. In line with these *in patient* observations, LRG1 release from human coronary artery endothelial cells or monocytes following inflammatory *in vitro* challenge was quenched by the inhibition of IL-1 signaling, but promoted by stimulation of cells with IL-1b.

**Conclusion:** In the investigated KD study population LRG1 as possible trigger of endothelial activation, neovascularization and cardiac re-modelling^2^ appeared tightly associated with IL-1 signaling. Besides a potential pathomechanistic implication of these findings our data suggest monitoring of LRG1 serum levels alongside with blood leukocyte counts to support steering of IL-1R1 blocking KD treatment.

**References**

1. Kone-Paut et al., Arthritis Rheumatol, 2020

2. Wang et al., Nature, 2013

**Disclosure of Interest**

None declared

### P293 Macrophage polarization in Henoch-Schönlein’s purpura nephritis

#### N. Kifer^1^, M. Sestan^1^, M. Held^1^, D. Kifer^2^, M. Frkovic^1^, E. Babarovic^3^, S. Bulimbasic^4^, M. Coric^4^, A. Gagro^5^, G. Laskarin^6^, M. Jelusic^1^

##### ^1^Department of Paediatrics, Division of Rheumatology and Immunology, University Hospital Centre Zagreb, University of Zagreb School of Medicine; ^2^Department of Biophysics, Faculty of Farmacy and Biochemistry; ^3^Department of General Pathology and Pathological Anatomy, University of Rijeka, Faculty of Medicine; ^4^Department of Pathology and Cytology, University Hospital Centre Zagreb, University of Zagreb School of Medicine; ^5^Department of Paediatrics, Division of Rheumatology and Immunology, Children's Hospital Zagreb, Univesity of Osijek Faculty of Medicine; ^6^Department of Physiology, Immunology and Pathophysiology, University of Rijeka, Faculty of Medicine, Zagreb, Croatia

###### **Correspondence:** N. Kifer

**Introduction:** Macrophages frequently infiltrate injured glomerular and tubulointerstitial tissue, and it is possible that the degree and subtype of macrophage infiltration (M1 macrophages, which show proinflammatory features, and M2 macrophages, with their immunosuppressive features) varies depending on the type and severity of renal injury. Although renal involvement in patients with Henoch-Schönlein's purpura (HSP) is the main cause of morbidity and mortality and a significant prognostic determinant for the disease outcome, a reliable prognostic factor for severe forms of HSP nephritis (HSPN) is yet to be determined.

**Objectives:** The aim of this research was to determine macrophage subclasses in renal biopsy specimens of HSPN patients and to analyze their quantity in regard with patients' clinical parameters and histologic features.

**Methods:** We performed an immunohistochemical study on renal tissue samples of patients with HSPN, diagnosed by EULAR/PRINTO/PRES criteria and followed for at least 6 months. Patient clinical and laboratory data was retrieved from hospitals' medical records. Renal biopsy samples were marked with antibodies for CD68, iNOS and arginase. The number of immunoreactive cells was counted in each glomerulus by two independent experts.

**Results:** Laboratory and histologic data for 25 patients with HSPN was evaluated in regard with macrophage infiltration of the renal tissue. The median glomerular M1 and M2 counts (q1, q3) were 2.3 (0.9, 12.2) and 7.6 (4.4, 13.9), respectively. M1 macrophages were found statistically significantly less frequent in the glomeruli in comparison with M2 macrophages (p < 0.001, b = -0,289 ± 0.053). Collected laboratory data included inflammatory markers and markers of kidney function. There was no significant correlation between M1 and M2 macrophage count and laboratory parameters. Four pathohistological classifications were used: ISKDC, Haas classification, Oxford classification, and SQC classification. Classification stages/total classification scores and all histological variables were evaluated for possible correlation with macrophage count. Statistically significant negative correlation was found between segmental glomerulosclerosis (Oxford classification) and M2 macrophages (p = 0,001, b = -1,050 ± 0,275). An indication of negative correlations was also noted between M2 macrophages and segmental sclerosis, and M2 macrophages and adhesions (SQC classification).

**Conclusion:** Glomeruli in HSPN showed predominant M2 polarization of macrophages. M2 macrophage infiltration of glomeruli was highlighted as a possible negative predictor for segmental glomerulosclerosis.

Grant/research support: Croatian Science Foundation project PURPURAPREDICTORS IP-2019-04-8822 and University of Rijeka, Croatia grant No. Uni-ri-biomed-18-110.

**Patient Consent Received**

No

**Disclosure of Interest**

None declared

### P294 Plasmapheresis therapeutic effect on refractory Kawasaki disease

#### E. Mercedes, E. Faugier, S. Rodriguez, P. P. Ramos, H. Menchaca, N. De La Rosa, M. De La Cera

##### Reumatologia Pediatrica, Hospital Infantil de México Federico Gómez, Ciudad de México, Mexico

###### **Correspondence:** E. Mercedes

**Introduction:** Kawasaki disease (KD) is a self-limiting medium vessel vasculitis of unknown etiology. Refractory Kawasaki disease occurs in 10-20% of patients. The AHA defines patient’s refractory to IVIG treatment as patients who develop persistent fever or recrudescence of fever at least 36 hours after the end of IVIG infusion and an increased risk of developing coronary aneurysms.

In these situations, administration of a second dose of immunoglobulin is generally recommended, which can achieve high success rates. The dilemma arises when they also fail to respond to this second dose.

**Objectives:** To describe the therapeutic effect of plasmapheresis in refractory Kawasaki disease.

**Methods:**

MATERIAL AND METHODS

Description of a clinical case and review of the literature.

CLINICAL CASE

Infant younger than 7 months, previously healthy, with diagnosis of refractory Kawasaki. Harada 6 points. Did not respond to two doses of intravenous immunoglobulin, three boluses of methylprednisolone, infliximab. Persists with elevated C-reactive protein 3, thrombocytosis, increased aneurymes. Refractory to cyclophosphamide, azathioprine; in addition to second scheme of 3 boluses of methylprednisolone and infliximab. When remission was not achieved, treatment was escalated to 3 sessions of plasmapheresis. Subsequent evolution was favorable.

Translated with www.DeepL.com/Translator (free version)

MATERIAL AND METHODS: Description of a clinical case and review of the literature.

CLINICAL CASE: Infant younger than 7 months, previously healthy, with diagnosis of refractory Kawasaki. Harada 6 points. Did not respond to two doses of intravenous immunoglobulin, three boluses of methylprednisolone, infliximab. Persists with elevated C-reactive protein 3, thrombocytosis, increased aneurymes. Refractory to cyclophosphamide, azathioprine; in addition to second scheme of 3 boluses of methylprednisolone and infliximab. When remission was not achieved, treatment was escalated to 3 sessions of plasmapheresis. Subsequent evolution was favorable.

**Results:**

**Conclusion:** Therapeutic management was in accordance with the AHA 2017, SHARE 2019 and Japanese 2020 guidelines for refractory Kawasaki. Given the evidence of persistent inflammatory activity documented by progressive increase in acute phase reactants and aneurysm size, it was decided to use azathioprine, cyclophosphamide for being a medium caliber vasculitis and giving long-term therapeutic effect. Affection in other blood vessels was ruled out by CT angiography.

To immediately stop the inflammatory effect, it was decided to use plasmapheresis. This therapeutic procedure has been described as a therapeutic option in refractory Kawasaki and other autoimmune diseases to stop the inflammatory process immediately. Obtaining satisfactory results. Individualized therapeutic decision taken with the experience of the medical staff and accessibility of hospital resources. Situation referred to in the literature.

The present case demonstrates the efficacy of plasmapheresis in refractory Kawasaki as a therapeutic option.

**Patient Consent Received**

Yes

**Disclosure of Interest**

None declared

### P295 Gastrointestinal involvement in childhood IgA vasculitis and the role of the neutrophil

#### F. Milton^1^, L. Oni^1,2,3^

##### ^1^Women's and Children's Health, University of Liverpool; ^2^Department of Paediatric Nephrology, Alder Hey Children's NHS Foundation Trust; ^3^Liverpool Medical School, Univeristy of Liverpool, Liverpool, United Kingdom

###### **Correspondence:** F. Milton

**Introduction:** Gastrointestinal (GI) involvement in IgA vasculitis (IgAV) can present with a range of clinical features including abdominal pain, vomiting, diarrhoea, GI bleeding and intussusception. Previous studies have suggested acute phase neutrophil concentrations may distinguish patients with GI involvement.

**Objectives:** The aim of this study was to describe the GI manifestations in a cohort of children with IgAV and to evaluate the neutrophil concentration as a marker of GI involvement.

**Methods:** A single centre, retrospective cohort study using data from electronic medical records of patients with a clinical diagnosis of IgAV over a 5-year period (2015-2019). Data was recorded on clinical features, management, investigations and remission status. In a subgroup of patient’s the acute phase neutrophil concentrations were compared between children with GI involvement and children without.

**Results:** 153 children were included in the study; 54 (35%) had GI involvement. GI symptoms included abdominal pain (96%), vomiting (67%), GI bleeding (26%), intussusception (15%) and diarrhoea (7%). Most GI symptoms developed during the acute phase of the disease (<7 days from disease onset). 17 (31%) patients received corticosteroids and 2 (4%) had surgery for intussusception. At the time of the study 93% of the patients were in complete remission. No significant difference was found in the absolute neutrophil count (ANC) (6.93 vs 5.71; p=0.732) or neutrophil:lymphocyte ratio (NLR) (4.13 vs 1.93; p=0.493) for patients with IgAV and GI system involvement compared to those without.

**Conclusion:** GI system involvement occurred in 35% of the children in this cohort. Severe complications, such as intussusception were uncommon and often resolved without the need for surgery. Abdominal pain was the most common GI symptom and usually occurs during the acute phase of the disease. The overall complete remission rate for children with GI involvement and IgAV was high. Finally, there was no significant difference in neutrophil concentrations for patients with GI involvement in IgAV.

**Patient Consent Received**

No

**Disclosure of Interest**

None declared

### P296 Nationwide clinical epidemiological study of Takayasu arteritis in Japan 2017

#### T. Miyamae^1,2^, N. Konda^2^, K. Saeki^3^, S. Ito^4^, N. Iwata^5^, N. Nakano^6^, Y. Nakamura^7^, Y. Nakaoka^8^, M. Harigai^2^

##### ^1^Pediatric Rheumatology, Institute of Rheumatology, Tokyo Women’s Medical University; ^2^Department of Rheumatology, Tokyo Women’s Medical University School of Medicine, Tokyo; ^3^Department of Epidemiology, Nara Medical University School of Medicine, Kashihara; ^4^Department of Pediatrics, Yokohama City University, Yokohama; ^5^Department of Infection and Immunology, Aichi Children’s Health and Medical Center, Obu; ^6^Pediatric Medical Center, Ehime Prefectural Central Hospital, Matsuyama; ^7^Department of Public Health, Jichi Medical School, Shimotsuke; ^8^Department of Vascular Physiology, National Cerebral and Cardiovascular Center, Suita, Japan

###### **Correspondence:** T. Miyamae

**Introduction:** The recent advances in imaging technologies have enabled early detection of the large-vessel vasculitis, allowing for early diagnosis and treatment. Approvement of tocilizumab for Takayasu arteritis (TAK), 2017 in Japan, has led to a significant change in the treatment strategy and clinical course.

**Objectives:** This study aimed to investigate current prevalence and clinical characteristics in TAK by a nationwide survey in Japan.

**Methods:** The first survey was designed to estimate the number of patients with TAK at each institution in 2017. The second survey collected clinical characteristics of the patients with TAK who were reported from the medical institutions in the first survey. Young- and adult-onset TAK were defined as onset age < 18 and >= 18 years of age. These comparisons were made between the two age groups.

**Results:** Among 14,291 medical institutions in Japan, 3,495, 1,696 internal medicine, 890 cardiology, and 909 for pediatrics, were selected. The collection rate was 56.1%. Considering that sampling rate was 20%, the number of TAK patients with the clinical diagnosis and those with the criteria-based diagnosis (95% CI) was estimated to be 5,320 (4,810–5,820) and 4,880 (4,410–5,360). The numbers of reported patients in the second survey were 1,571 with TAK in total, including 263 with young-onset (16.7% of overall TAK), and 1308 with adult-onset TAK. The young- and adult-onset groups were predominantly female, but the former group tended to have a slightly higher ratio of males (1:5.6) than the latter group (1:7.3). The median [interquartile range, IQR] age at onset and disease durations at the survey were 24.5 [20 to 47] and 10 [4 to19] years. The proportions of patients who met the diagnostic criteria were 96.1%, 98.4%, and 95.6% for overall TAK, young- and adult-onset TAK, respectively. Compared with adult-onset TAK, young-onset TAK showed a significantly higher proportion of vascular lesions in the common carotid artery to the internal carotid artery, abdominal descending aorta, renal artery, celiac, and superior mesenteric artery. Methylprednisolone pulse therapy was applied in 39.2% of young-onset TAK patients, a significantly higher rate than in 4.8% of adult-onset TAK patients. Immunosuppressants and biologics were also selected at a higher rate in young-onset cases, 64.3% and 40.0%, respectively. In particular, tocilizumab was used in 31.7% of all young-onset cases and 79.4% of young-onset cases in which biologic agents were selected. The surgical procedure was performed in 19.9% of young-onset TAK. Disease-associated complications in patients with young-onset TAK were reported in 57.8%: aortic regurgitation 36.9%; ulcerative colitis 7.5%; stroke, 5.6%; and ischemic heart disease, 5.4%. Of the young-onset TAK patients, 93.7% achieved remission by investigator assessment after six months of treatment, a rate similar to that of adult-onset patients. However, after achieving remission, 51.8% of patients with young-onset TAK relapsed at some point during the follow-up period, a significantly higher rate than the 43.8% of adult-onset patients.

**Conclusion:** The clinical features of young-onset TAK became clearer when compared to adult-onset cases in this study. There was a significant tendency for subdiaphragmatic arterial lesions to be more common in young-onset cases. Despite aggressive immunosuppressive therapy, including biologics, the relapse rate after achieving remission was significantly higher in young-onset TAK than in adult-onset, suggesting that young-onset TAK is more challenging to treat.

**Disclosure of Interest**

None declared

### P297 Childhood Behçet disease: clinical features, management and outcome of 13 cases

#### H. Nassih, R. El Qadiry, A. Bourrahouat, I. Ait Sab

##### Pediatrics, Mohammed 6 University Hospital Center of Marrakesh, Marrakesh, Morocco

###### **Correspondence:** H. Nassih

**Introduction:** Behçet's disease is a rare vasculitis that involves median and small caliber arteries. Multi-systemic involvement is possible. Few are the papers that provide information about its clinical course, and outcome in the pediatric population. Also, the management is mainly based on adult guidelines.

**Objectives:** To shed light on the clinical course and outcome of juvenile Behçet disease.

**Methods:** A single center retrospective descriptive study of 13 children with Behçet disease. The mean follow-up period was of 3 years.

**Results:** We had 8 boys and 5 girls. Four of our patients were consanguineous. The mean age was of 9-year-old. The mean interval between onset and diagnosis was of 3 months. All of our patients had recurrent aphthous stomatitis with a median of 6 episodes per year. The other symptoms at diagnosis were concomitant genital ulcers in 4 cases, pseudo-folliculitis in 7 cases, urticaria rush in 4 cases, severe headache in 2 patients, stroke in one patient, prolonged and/or recurrent fever in 9 patients, uveitis in 4 cases, episcleritis in one case, arthritis in 5 cases, pulmonary hemorrhage in one case, and pyoderma gangrenosum in one case. The pathergy test was performed in all the patients, and was positive in 3 of them. Diagnosis was based on the International Criteria for Behçet's disease. Acute phase reactants (ESR and CRP) were high in all patients. Meanwhile, granulocytosis was found in 10 patients. As for HLA 51 testing, it was positive in 8 patients, from whom three were siblings. Colchicine was prescribed in 5 cases. All our patients were put on steroids, that was associated with azathioprine in 7 cases, methotrexate in 2 cases, cyclophosphamide in 2 cases, etanercept in 3 cases, and tocilizumab in one case. Complete remission was obtained in 8 cases in a mean time of 15 months, while five children had partial remission, and one boy lost sight of his left eye after a severe retinitis.

**Conclusion:** Bechet disease can cause severe sequalae if not diagnosed and treated on time. More studies are needed to determine what is the right management protocol to adopt in the pediatric patients.

**Disclosure of Interest**

None declared

### P298 Difficulties in managing a patient with ANCA-associated vasculitis and diabetes

#### O. A. Oshlianska^1^, A. G. Artsymovych ^1^, V. M. Nepomniashchyi^2^, T. G. Nadtochii ^3^

##### ^1^1.Department of Pediatrics № 1, 2. Department of Connective Tissue Disorders in Children, 1. Shupyk National Medical Academy of Postgraduate Education, 2. State Institute of Pediatrics, Obstetrics and Gynecology, Academy of Medical Sciences of Ukraine, Киев; ^2^Pathomorphology Laboratory, SI “Institute of Nephrology NAMS of Ukraine”; ^3^Department for Older Children, State Institute of Pediatrics, Obstetrics and Gynecology, Academy of Medical Sciences of Ukraine, Kyiv, Ukraine

###### **Correspondence:** A. G. Artsymovych

**Introduction:** Systemic vasculitis is a rare and extremely severe pathology in childhood. The proposed EULAR/ERA-EDTA recommendations for the management of ANCA-associated vasculitis do not contain guidelines for the management of patients with comorbid conditions such as diabetes mellitus, including corticosteroid (CS) dosage regimens.

**Objectives:** to analyze the disease in a child with SV and diabetes

**Methods:** an analysis of the disease hystory

**Results:** Patient J., boy, diabetes mellitus was diagnosed in 1.5 y, suboptimal glycemic control with insulin therapy. At the age of 13, manifestated with hemorrhagic rash, abdominal and joint syndrome, diagnosed with SV, prescribed CS followed by complete withdrawal, which led to exacerbation of the disease: joint, abdominal ischemic, peptic ulcer syndromes, weight loss, ESR 56mm/h, WBC 20.2^^9^/L, Hgb 69g/l, protein 51g/l, received pulse CS+cyclophosphamide (CP). Hemorrhagic and articular syndromes persisted, respiratory distress syndrome developed, widespread edema, hypertension, glycemia 18.9μmol/l. Repeated combined pulse therapy was performed, heparin therapy was added. After 1 month necrosis of the toes, persisted joint, edema and urinary syndrome. In the hemogram Hgb 75g/l, WBC 12.6^^9^/L, PLT 693^^9^/L, ESR 24mm/h, according to biochemical blood tests CHO 9.4μmol/l, urea 7.98μmol/l, creatinine 0.102μmol/l, cystatin C 1.32mg/l. Immunological parameters: ANCA Pt 3 IgG >8 IU/μl (n<1), IgG 11.7, IgA 2.6, IgM 1.7g/l; CD19+ 19.09% (1022 cells/μl); B1A CD5+ 18.34%. INR 4.83-1.05; PTI 10.1-92%, APTT 40.5-20.1sec. Massive erythrocyturia. Microalbuminuria 288.5 mg/24h, albumin/creatinine 98.1. Cardiac ultrasound: moderate left ventricular myocardial hypertrophy. MSCT: the transparency of the lung parenchyma is significantly reduced, single foci of significant acinar seal up to 10 mm due to hemorrhage. Diagnosed with ANCA-associated SV (MPA? GPA?), BVAS 32. Added CP to 2 mg/kg/d, CS rised to 1.5 mg/kg/d, infusion therapy with the FFP, rituximab 500 mg №2. There was a positive trend in a month: urinary and edematous syndrome were gone; Hgb 100g/l, ESR 3 m/h, biochemical and coagulogram parameters were normal, CD19+ 0.16%, however, glycemia and glucosuria were observed. Started reducing CS. Received infusions of IVIG for substitution purposes, the dose of CP was reduced due to leukopenia, necrotized areas auto-amputated. Six months later on the background of CS 16 mg/d with CP 50 mg/d revealed a progression of urinary syndrome (proteinuria >3.0g/l/d, microalbuminuria 1309.0 mg/d). However, cystatin C was 1.21mg/l, GFR 73ml/min, microalbumin/creatinine 170.6 mg/mmol. Since the total disease activity was not observed (IgG PT3 8 IU/μl, IgG 3.8 g/l, IgA 0.5 g/l, IgM 0.3g/l, ESR 7mm/h, CRP neg, coagulogram and blood biochemistry within normal limits) performed a biopsy of the kidney by video-assisted puncture. According to the results of nephrobiopsy: A. E. Berden et al. prognostic class was mixed, invented focal global glomerulosclerosis 9/35 (26%, SI95% 14-42%), fibrous crescents - 11% (SI95% 5-26%), cellular crescents 3% (SI95% 0.5-14%), focal segmental glomerulosclerosis 11% (SI95% 5-26%), tubular atrophy and interstitial fibrosis - 15-20%. The total score of chronic kidney damage was 5. It was decided to refrain from increasing CS. After 5 months (2 years from onset) there is remission of the disease (BVAS 4), microalbuminuria 208 mg/d.

**Conclusion:** It is clinically impossible to distinguish SV-associated nephrotic lesions from diabetic nephropathy. The use of nephrobiopsy and steroid-preserving treatment tactics may be helpful in cases of a combination of SV and diabetes.

**Patient Consent Received**

Yes

**Disclosure of Interest**

None declared

### P299 Monocyte activation markers in children with Kawasaki disease: a preliminary study from a tertiary care centre in North India

#### V. Pandiarajan, A. Gupta, K. Sairam, K. Arora, R. Kumrah, J. Das, J. K. Shandilya, D. Suri, A. Rawat, S. Singh

##### Allergy Immunology Unit, Department of Pediatrics, Postgraduate Institute of Medical Education and Research, Chandigarh, India

###### **Correspondence:** V. Pandiarajan

**Introduction:** Kawasaki disease (KD) is a childhood vasculitic disorder that preferentially involves coronary arteries. Monocyte/ macrophage activation is an integral part of pathogenesis of KD. Monocyte activation markers may serve as surrogate laboratory markers for early diagnosis of KD.

**Objectives:** To analyse the monocyte/ macrophage activation markers in children with KD and compare it with age-matched febrile and healthy controls.

**Methods:** Our study assessed different subsets of monocytes (Classical [CD14+CD16], intermediate [CD14++CD16+], and non-classical [CD14+CD16++] monocytes in 11 children diagnosed with KD and compared them with healthy and febrile age-matched controls. Early and late activation markers (CD69 and HLA-DR, respectively) in different monocyte subsets was also assayed using flow cytometry. Plasma levels of monocyte activation markers (sCD14, sCD163, MCP-1/ CCL2) in children with KD (n=16), age and sex-matched febrile (n=16) and healthy controls (n=16) were assayed using Quantikine® ELISA kits (R&D Systems® USA). Analysis was performed in a fully-automated ELISA machine (Infinite200 pro) and results were read in the ELISA reader (Tecan). Monocyte activation markers were also assessed after intravenous immunoglobulin (IVIg) infusion and 3 months after the acute episode of KD.

**Results:** Median (IQR) duration of fever among children with KD and febrile controls were 9 (7-15) and 7.5 (6.5-18) days, respectively. Children with acute KD and febrile controls had a significant elevation in absolute monocyte counts as compared to healthy controls (p-0.007). Children with acute KD also had higher absolute numbers of classical (p-0.056) and intermediate monocytes (p-0.013) as compared to healthy controls. Absolute numbers of monocytes that show positivity for early activation marker (CD69) were significantly higher in cases and febrile controls as compared to healthy controls (p-0.001). Significantly higher absolute counts of CD14+ monocytes having HLA-DR expression were seen in acute KD as compared to healthy controls (p-value 0.019).

Median (IQR) plasma sCD14 levels were higher in febrile controls 2754.10 (2249.50-3226.74) ng/mL, compared to children with acute KD 2228.84 (1899.34-2425.22) ng/mL and healthy controls 2252.64 (1375.07-2737.17) ng/dL (p-0.038). There was no significant difference in sCD163 values between cases, febrile, and healthy controls. Median (IQR) CCL2 levels were high in children with KD 318.85 (184.29-449.23) pg/mL. However, the differences were not statistically significant between cases, febrile, and healthy controls (p-0.075). Levels of CCL2 were higher in children with CAAs – however, the difference was not statistically significant (p-0.082).

Absolute counts of classical and intermediate monocytes were higher in acute KD and it progressively decreased after administration of IVIg and at 3 months follow-up. A significant decrease in both early (p-0.007) and late activation (p-0.022) in CD14+ monocytes was also noted in children with KD at 3^rd^ month follow-up. Levels of CCL2 significantly dropped after IVIg infusion in children with KD (p-0.47). There were no significant fluctuations in levels of sCD14 and sCD163 over time in children with KD.

**Conclusion:** Both classical and intermediate monocytes are elevated in acute stages of KD. We also documented an activated status of both classical and intermediate monocytes in acute phase of KD that subsided on follow-up. Our results suggest that IVIg may decrease monocyte activation in KD, thereby controlling systemic inflammation. We did not observe significant elevation in sCD14 and sCD163 levels, probably due to the prolonged duration of fever in our cohort of children with KD.

**Patient Consent Received**

Yes

**Disclosure of Interest**

None declared

### P300 Early, but not, late activation of lymphocytes is noted in acute stages of Kawasaki disease: our experience from a tertiary care centre in North-West India

#### V. Pandiarajan, P. Sharma, K. Arora, R. Kumrah, K. Sharma, J. Das, J. K. Shandilya, D. Suri, A. Rawat, S. Singh

##### Allergy Immunology Unit, Department of Pediatrics, Postgraduate Institute of Medical Education and Research, Chandigarh, India

###### **Correspondence:** V. Pandiarajan

**Introduction:** Autopsy studies have shown that T-lymphocytes play a major role in pathogenesis of Kawasaki disease (KD). Previous studies on T-lymphocyte activation in KD have shown conflicting results.

**Objectives:** To analyse lymphocyte activation markers in children with KD and compare it with age and sex-matched febrile and healthy controls.

**Methods:** We enrolled 10 children during the acute stage of KD and 19 age and sex-matched controls (10 healthy controls; 9 febrile controls) after obtaining consent. We studied both early (CD69) and late (HLA-DR) activation markers of T-cell activation by flow cytometry. T-cell activation profiles were assayed after 24 hours and 3 months of intravenous immunoglobulin (IVIg) administration. We estimated soluble CD25 levels in serum by Sandwich ELISA. mRNA expression of HLADRA and HLADRB was assessed using RT-PCR.

**Results:** Among patients with KD, 4 had complete KD and 6 had incomplete presentation, and none had coronary artery abnormalities or IVIg-resistant KD. Compared to healthy controls [median (IQR): 1.74% (0.78-2.29)], children with KD [median (IQR): 2.65% (1.81-4.74)] and febrile controls [median (IQR): 3.57% (2.68-4.48)] showed increased expression of CD69 on CD3+CD4+ T cells (p-0.04). CD69 expression on CD3+CD8+ T cells was also increased in children with KD [median (IQR): 4.84% (3.07-6.7)] and febrile controls [median (IQR): 6.33% (3.32-7.93)] as compared to healthy controls [median (IQR): 2.7% (1.86-3.27)], (p-0.056). No difference was found in HLA-DR expression between KD, febrile, and healthy controls. No significant difference in mRNA expression of HLADRA and HLADRB was observed between KD and healthy controls. Soluble CD25 was significantly elevated in KD [median (IQR): 3681.5 pg/mL (2026.75-4542)] as compared to febrile [median (IQR) 2755 pg/mL (2026-4098)] and healthy controls [median (IQR): 514 pg/mL (503.75-534)]. Levels of soluble CD25 in children with KD decreased on follow-up after IVIg administration.

**Conclusion:** To conclude, we document early but not late activation of T lymphocytes in children with KD. Markers of lymphocyte activation show a decrease with subsidence of systemic inflammation following IVIg therapy in KD.

**Patient Consent Received**

Yes

**Disclosure of Interest**

None declared

### P301 Can we predict the development of nephritis in pediatric IgA vasculitis patients?

#### E. Sag^1,2^, S. Demir^2^, Y. Bilginer^2^, S. Ozen^1,2^

##### ^1^Pediatric Rheumatology Unit, Translational Medicine Laboratories, Hacettepe University; ^2^Division of Pediatric Rheumatology, Hacettepe University Faculty of Medicine, Department of Pediatrics, Ankara, Turkey

###### **Correspondence:** E. Sag

**Introduction:** IgA vasculitis (IgAV) is the most common systemic vasculitis of childhood, characterized by palpable purpura, arthritis, gastrointestinal and renal involvement^1^ . It is a relatively self limited disease apart from the renal involvement which is associated with the long term morbidity.

**Objectives:** We aimed to define a marker at disease onset to predict the renal involvement.

**Methods:** In this pilot study, we analyzed a targeted panel of vascular inflammation markers (sT2, RAGE, TIE-2, sCD40L, TIE-1, sFlt-1, LIGHT, TNF-a, PlGF, IL6, IL18, IL10 and MCP-1) in the plasma samples of eight patients IgAV at the onset of the disease, before any treatment was initiated. At the time of sample collection, none of the patients had renal involvement; four of these patients subsequently developed nephritis and were defined as the IgAVN group. The levels of the markers were studied by a cytometric bead-based multiplex assay panel according to manufacturer’s instruction (LEGENDplex HU Vascular Inflammation panel 2 (13-plex); cat:740966, Biolegend) and analyzed by Novocyte 3005 flow cytometer.

**Results:** There were no significant differences in gender, age, clinical manifestations, and laboratory findings between IgAV and IgAVN patients (Table 1). sCD40L levels were higher (median 1938.1 vs 754.9 pg/mL, p=0.04) whereas sST2 levels were lower (median 862.8 vs 2302.8 pg/mL, p=0.02) in the patients who developed IgAV nephritis. sRAGE levels were higher and IL18 levels were lower in IgAVN patients but did not reach statistical significance, probably due to the low number of patients. The other parameters did not show any specific pattern.

Soluble CD40 ligand (sCD40L) is expressed on platelets and released on activation. It is proinflammatory for endothelial cells and promotes coagulation by inducing expression of tissue factor on monocytes and endothelial cells.^2^ It was investigated in acute coronary syndrome and was suggested as a risk factor for vascular damage.^2^ Furthermore, the levels of sCD40L were shown to be correlated with disease activity in ANCA-associated vasculitis.^3^ Thus in IgAV, sCD40L may be associated with the involvement of the kidney vasculature, which represents the more severe form of the disease. Receptor for advanced glycation endproducts (RAGE) is another marker for endothelial damage and vasculitis which was higher in IgAVN, as well. IL33 is expressed by epithelial and endothelial cells and overexpression of IL33 have been shown in large vessel vasculitis.^4^ sST2 acts as a decoy receptor and inhibits IL33 signalling.^4^ It was interesting to see that the levels of the IL-18 and sST2, which are members of IL-1 family, were lower in IgAVN patients.

**Conclusion:** Although the number of the patients were limited in this pilot study, we suggest that sCD40L and sRAGE might be used to predict the development of renal disease in IgAV patients. Further studies with larger number of patients are needed to confirm our findings.

**References**

1. Sag E, et al *Best Practice & Research Clinical Rheumatology 2017;31(4):558-75.*

2. Heeschen C, et al. *NEJM 2003;348(12):1104-11.*

3. Tomasson G, et al. *The Journal of Rheum 2011;38(6):1048-54.*

4. Desbois AC, et al. *Sci Rep 2020;10(1):6405.*

**Patient Consent Received**

Yes

**Disclosure of Interest**

None declared

**Table 1 (abstract P301).** Clinical and laboratory features of the patients

	IgAV (n=4)	IgAVN (n=4)	p value
**Gender**	2F, 2M	1F, 3M	0.49
**Age at disease onset**	9.0±5.4	10.3±3.4	0.68
**Arthralgia/arthritis**	50%	75%	0.49
**GIS involvement**	75%	50%	0.49
**White blood cells, x10**^**9**^**/L**	11150±1933	8525±1497	0.08
**Platelets x10**^**9**^**/L**	290250±87328	269250±38741	0.68
**Erythrocyte sedimentation rate (mm/hr)**	10.5±9.1	21.0±14	0.26
**C-reactive protein (mg/dL)**	0.63±0.03	1.16±0.96	0.39

**Data are given as mean±SD**

*IgAV* Immunoglobulin A vasculitis, *IgAVN* Immunoglobulin A vasculitis nephritis, *GIS* Gastrointestinal system

### P302 How useful is machine learning in predicting childhood IgA-vasculitis relapses?

#### M. Sapina^1^, M. Sestan^2^, N. Kifer^2^, M. Batnozic Varga^3^, M. Held^2^, S. Srsen^4^, A. Ovuka^5^, M. Frkovic^2^, A. Gagro^6,7^, M. Jelusic^2^

##### ^1^University Hospital Centre Osijek, Department of Pediatrics, Josip Juraj Strossmayer University of Osijek, Faculty of Medicine, Faculty of Dental Medicine and Health, Osijek; ^2^University Hospital Centre Zagreb, Department of Pediatrics, University of Zagreb School of Medicine, Zagreb; ^3^University Hospital Centre Osijek, Department of Pediatrics, Josip Juraj Strossmayer University of Osijek, Faculty of Medicine, Osijek; ^4^University Hospital Centre Split, Department of Pediatrics, University of Split School of Medicine, Split; ^5^University Hospital Centre Rijeka, Department of Pediatrics, University of Rijeka School of Medicine, Rijeka; ^6^Department of Pediatrics, Children's Hospital Zagreb, Zagreb; ^7^Josip Juraj Strossmayer University of Osijek, Faculty of Medicine, Osijek, Croatia

###### **Correspondence:** M. Sestan

**Introduction:**

IgA vasculitis (IgAV) is the most common systemic vasculitis in children. The mandatory clinical feature of the disease is purpuric rash, which predominantly affects the lower extremities, accompanied by diffuse abdominal pain, joint involvement, nephritis and/or IgA deposition in biopsy specimen (skin, intestinal tract, kidney). In most cases, IgAV is a self-limiting disease with favorable outcomes, however, relapses are not uncommon.

**Objectives:** The aim of this study is to evaluate the usefulness of supervised machine learning (ML) algorithms in the identification of patients which could develop IgAV relapses.

**Methods:** A large set of predictive variables related to demographic variables, clinical history, symptoms, laboratory values, and medications were used in the initial data collection for developing a predictive ML model. After preparing the dataset, handling missing values and data imbalances, a random forest (RF) decision tree and support vector machine (SVM) model with polynomial kernel were trained, crossvalidated and tested.

**Results:** This pilot study included 539 IgAV patients (260 males, and 279 females), with a median age of 6.17 (4.42 to 8.75) years. Among them, 78.11% had joint involvement, 44.53% had gastrointestinal involvement, and in 18.92% nephritis has developed. Atypically distributed rash (not affecting lower limbs as well as rash that had been generalized from the onset of the disease) was found in 5.19% IgAV patients, while 8.91% had persistent purpura for a month or more. The incidence of IgAV relapses was 10.2%. The RF model produced an overall accuracy of 95%, with a sensitivity of 100%, and specificity of 94.48%. The SVM model produced an accuracy of 87.58%, with a sensitivity of 100%, and specificity of 86.21%. The most useful predictor variable was the presence of persistence rash in both models. Other common predictors in both models included the presence of rash on atypical locations, age, and nephritis.

**Conclusion:** The results of this pilot study show promising applications of ML as an useful aid in predicting vulnerable patients who developed IgAV.

Support: Croatian Science Foundation project IP-2019-04-8822.

**Patient Consent Received**

No

**Disclosure of Interest**

None declared

### P303 Risk factors for coronary arterial involvement in Turkish children with Kawasaki disease: a multicenter retrospective study

#### S. Turkucar^1^, U. Akca Kaya^2^, F. Cakmak^3^, F. Haslak^4^, F. Demir^5^, E. Karabulut^6^, B. Makay^1^, Y. Bilginer^2^, N. A. Ayaz^3^, B. Sozeri^5^, O. Kasapcopur^4^, T. Karagoz^7^, N. Unal^8^, S. Ozen^2^, E. Unsal^1^

##### ^1^Departments of Pediatrics, Pediatric Rheumatology, Dokuz Eylul University Faculty of Medicine, Izmir; ^2^Departments of Pediatrics, Pediatric Rheumatology, Hacettepe University, Ankara; ^3^Departments of Pediatrics, Pediatric Rheumatology, Istabul University; ^4^Departments of Pediatrics, Pediatric Rheumatology, Istabul University, Carrahpasa School of Medicine; ^5^Departments of Pediatrics, Pediatric Rheumatology, Umraniye Training and Research Hospital, Istanbul; ^6^Department of Biostatistics; ^7^Department of Pediatric Cardiology, Hacettepe University, Ankara; ^8^Department of Pediatric Cardiology, Dokuz Eylul University Faculty of Medicine, Izmir, Turkey

###### **Correspondence:** S. Turkucar

**Introduction:** Coronary arterial lesions (CALs) are the major complication of Kawasaki Disease (KD) with significant morbidity and affects a substantial proportion of patients despite proper treatment. Previous studies in the literature defined several risk factors such as younger age, male gender, treatment delay, resistance to initial IVIG, high acute phase reactants and low albumin levels regarding increased risk of CALs. On the other hand, different geographical regions of the world such as Europe, Middle-East, Far-East and America define their self-risk-scoring systems, since current risk scoring systems are incapable for predicting CALs for all populations due to genetic and environmental differences.

**Objectives:** The aim of this study was to define the risk factors for CALs in Turkish children with KD.

**Methods:** Medical records of 399 KD patients from five pediatric rheumatology centers in Turkey were reviewed, retrospectively. Demographic, clinical (including duration of fever before IVIG and resistance to IVIG), laboratory and echocardiographic data were noted.

**Results:** The medical records of 233 boys (58.3%) and 166 girls (41.7%) were included in this study. Patients with CALs positive group had younger age, male predominance and longer duration of fever before treatment when compared to CALs negative group (p<0.05 for all parameters). In terms of laboratory parameters prior to first IVIG treatment, CALs positive group had lower hemoglobin (p=0.006) and higher lymphocyte (p=0.048) values than CALs negative group.

The predictive value of the variables revealed **male gender (*p<0.001*), age (*p=0.002*) and duration of fever prior to first IVIG treatment (*p*=0.036)** as independent predictors of coronary arterial lesions. When we applied ROC analysis to numerical variables, the best cut-off values were calculated as duration of fever ≥ 9.5 days and age ≤ 12 months. Based on these cut-off values, binary logistic regression analysis was applied for each parameter, and odds ratios were calculated as 2.252 for male gender, 3.112 for age ≤ 12 months and 2.084 for duration of fever before initial IVIG ≥ 9.5 days, respectively (Table). A new scoring system was defined with area under curve of 0.671 based on two of these three clinical parameters. The patients fulfilling at least two of these three criteria had an increased risk, with 83.1% specificity and 38.8% sensitivity.

**Conclusion:** Based on the demographic and clinical features, we established an easily applicable risk-scoring system for predicting CALs in Turkish children with KD. This may be useful for choosing optimal treatment and appropriate follow-up for KD to prevent coronary artery involvement. Further studies will show whether these risk factors can be used in other Caucasian populations as well.

**Patient Consent Received**

No

**Disclosure of Interest**

None declared

**Table 1 (abstract P303).** Multiple logistic regression analysis of predicting factors for CALs

	*Cut-off value*	*β*	*SE*	*Wald*	*df*	*Sig*	*Exp B*	*%95 Confidence Interval*	
								*Lower*	*Upper*
*Gender (Male)*		0.926	0.243	14.547	1	<0.001	2.525	1.569	4.065
*Age*	***≤12 Months***	1.135	0.308	13.572	1	<0.001	3.112	1.701	5.693
*Duration of fever before IVIG*	***≥ 9.5 Days***	0.735	0.241	9.270	1	0.002	2.084	1.299	3.343

### P304 Henoch–Schönlein purpura (IgA vasculitis): clinical profile and long-term renal outcome in children from a single centre in North-West India

#### S. Verma, H. Kothari, A. Sharma, A. Rafi, S. Guleria, R. Jaswal, S. Sharma, P. Gautam, J. Sharma, N. Chaudhary, M. Sharma

##### Pediatrics, Dr Rajendra Prasad Govt .Medical College,Tanda, Kangra, HP,India-176001, Kangra, India

###### **Correspondence:** S. Verma

**Introduction:** Henoch-Schönlein purpura (HSP), now aptly named as IgA vasculitis is one of the commonest vasculitic disorder of childhood, characterized by nonthrombocytopenic purpura, arthritis, abdominal pain and renal involvement. Renal damage is the most serious long-term complication of HSP.

**Objectives:** This study was done to evaluate the clinical profile, complications and long-term outcome of pediatric HSP patients

**Methods:** This retrospective study was done at a tertiary-care centre in North-India which is government run, tertiary-care medical institute. We collected the demographic data, the clinical manifestations, laboratory findings, outcomes and follow-up data of patients registered in Pediatric Rheumatology Clinic of the institute over last three and half years. Data were also analysed according to the presence of nephritis and without nephritis.

**Results:** There were 14 children (9 male) diagnosed to have HSP, during study period with M:F ratio of 1.8:1. Mean age of presentation was 12.3 years (range 3-16 years). Time to seek consultation ranges from 1 day to 1.5 months after appearance of first symptom/sign with mean duration of 13 days. Pain abdomen was the initial manifestation in 50% of patients followed by skin rash (29%) and arthritis (21%). However skin rash which was present in all brought them to hospital. Gastrointestinal involvement was seen in 57%, in form of pain abdomen (57%), bloody stools (29%), hematemesis (7%) and intussusception in 8 years old male child (7%). Renal involvement was seen in 43% in form of combination of hematuria and proteinuria. Hypertension was present in 21%. Other manifestations were arthralgia (40%), arthritis (29%), fever (29%), scrotal swelling (14%), swelling hands and feet (14%). None of our patients had nervous system involvement. There was significant difference in clinical presentation of patients with and without nephritis. The mean age of presentation was higher in patients with nephritis (12 years) as compared to patients without nephritis (8 years). Pain abdomen with bloody stools was main presentation (83%) in patients of HSP nephritis while other patients without nephritis mainly had arthritis (62.5%) and fever (50%). Recurrence of skin rash was present in 83% of children with HSP nephritis. Older age of presentation with abdominal symptoms were found as risk factor for HSP nephritis. Renal biopsy was done in 2 patients with renal involvement and showed fibrocellular crescents in one and mesangioproliferation in another patient. Patients with HSP nephritis were treated with high dose pulse steroid therapy followed by oral prednisolone and azathioprine or mycophenolate mofetil and were followed up. 83% of these patients continued to have proteinuria and hematuria on follow up (range11 months -25 months). However no abnormality was detected in their renal function tests. Presently 50% of these patients are on antihypertensive drugs.

**Conclusion:** Pain abdomen may be the initial symptom of HSP which may be a risk factor for development of HSP nephritis along with older age of presentation. In our study we found persistent renal involvement even after early use of high dose pulse corticosteroids along with immunosuppressive drugs. So renal monitoring for prolonged periods is required along with renal biopsy.

**Disclosure of Interest**

None declared

## e-Poster viewing: Miscellaneous rheumatic diseases

### P305 Chronic nonspecific constrictive pericarditis in camptodactyly-arthropathy-coxa vara-pericarditis syndrome

#### A. M. A. Abushhaiwia^1^, Y. AElfawires^2^

##### ^1^Paediatric Rheumatology, Tripoli Children's Hospital, Faculty of Medicine, Tripoli University; ^2^Paediatric Rheumatology, Tripoli Children Hospital, Faculty of Medicine, Tripoli University, Tripoli, Libya

###### **Correspondence:** A. M. A. Abushhaiwia

**Introduction:** Camptodactyly-arthropathy-coxa vara-pericarditis (CACP) syndrome is a genetic disorder caused by mutation in the Proteoglyacn PRG4 gene on chromosome 1 (Online Mendelian Inheritance in Man OMIM number 208250). The syndrome is characterized by congenital or early-onset camptodactyly and childhood-onset of non-inflammatory arthropathy, coxa vara deformity, or other dysplasia associated with progressive hip disease and non-inflammatory pericardial effusion. It has an autosomal recessive mode of inheritance and the causative gene is located on chromosome band 1q25-31.We present a case of a female child who presented with early onset camptodactyly, non-inflammatory arthropathy, and short femoral necks, with pericardial effusion.

**Objectives:** We describe a case of a 6-year -old female with features of CACP syndrome and with chronic nonspecific constrictive pericarditis.

**Methods:** Molecular genetic sequencing analysis were enriched using ROCH\KAPA sequence captured technology and sequenced on an illumina system (NGS) identified the homozygous variant c.3507T>Gp.Tyr1169 in the RAG4 gene is classified as likely pathogenic

**Results:** A 6-year -old female child,born of consanguineous healthy parents. Presented with a 3-month history of swelling of both knees and wrists. The parents also stated that they had noticed bent fingers for nearly and 4 years. On examination, the child had camptodactyly of the fingers with swelling of both knees, ankles and elbows without restricted motion or signs of inflammation like erythema or tenderness. Systemic examination showed no evidence of fever, lymphadenopathy, skin rash, or other systemic features. Her sibling and parents were normal on physical examination. Routine blood work-up revealed normal hemogram, Erythrocyte Sedimentation Rate (ESR) and C-reactive protein (CRP) with negative Antinuclear Antibody (ANA). Anteroposterior (AP) radiograph of pelvis revealed a broad short femoral neck and coxa vara. The articular surfaces were smooth with no erosions. X-ray of the hand was not obtained. X-ray of the knee revealed bilateral effusions with osteopenia and no erosions. Knee MRI showed large amount of joint fluid Knee MRI showed large amount of joint fluid with mildly thickened enhancing synovium**.** The electrocardiogram was normal. Echocardiography showed moderate pericardial effusion, suggesting constrictive pericarditis. Molecular genetic sequencing analysis identified the homozygous variant c.3507T>Gp.Tyr1169) chr1: 186280173 in the RAG4 gene is classified as likely pathogenic. The girl was given a clinical diagnosis of CACP syndrome. Subsequent extensive genetic testing was per formed to confirm the clinical diagnosis. Exome sequencing identified a homozygous pathogenic mutation

**Conclusion:** We recommend regular evaluation of clinical symptoms of constrictive pericarditis in children with CACP syndrome

**Patient Consent Received**

Yes

**Disclosure of Interest**

None declared

### P306 Bell’s palsy with facial bone involvement: a rare presentation of chronic non-bacterial osteomyelitis with literature review

#### H. Ailumerab^1^, C. Aguiar^2^

##### ^1^Pediatrcis, Children’s Hospital of The King’s Daughters; ^2^Pediatric Rheumatology, Children’s Hospital of The King’s Daughters, Norfolk, United States

###### **Correspondence:** H. Ailumerab

**Introduction:** Chronic non-bacterial osteomyelitis (CNO) is a chronic, sterile, inflammatory disease. It primarily presents with nonspecific bone pain and swelling, but ultimately can cause bone destruction and deformities, if left untreated [1]. CNO usually affects the metaphyses of tubular bones of lower extremities, followed by other parts of the body [1]. The involvement of the cranial and facial bones is rare in CNO. The mandible is the most reported facial bone in the literature [2].

**Objectives:** We present a rare case of CNO affecting facial and cranial bones (apart from the mandible) presenting as facial palsy with a review of the literature about similar affection.

**Methods:** A 10-year-old female with left side facial swelling and pain associated with elevated inflammatory markers initially assumed due to infectious osteomyelitis. Her imaging of the face and brain showed a destructive osseous process involving multiple cranial and facial bones. Bone biopsy of the left maxilla showed fibrous dysplasia with abscess formation. On empirical antibiotic treatment (Clindamycin), she developed a right-sided facial palsy. Toward the end of the antibiotic course, she developed another skeletal bone lesion at the right radius bone. Imaging showed multifocal lesions with mixed sclerosis and lucency. Bone biopsy of the right radius showed similar findings of destruction with no evidence of malignancy which helped towards the diagnosis of (CNO). Non-steroidal anti-inflammatory drug (NSAID) was started with regular follow up. Over more than a year of follow-up, the patient's inflammatory markers remain normal and joint swelling/limitation have remained in remission.

**Results:**

PubMed was used to search for articles using specific keywords. We found 5 cases in addition to our case

(Table 1).

**Conclusion:** The affection of the cranial & facial bones in CNO is very rare, but awareness of such presentation by the clinician is an important aspect of reaching the diagnosis.

**Trial registration identifying number:** None.

**References**

1. Iyer RS, Thapa MM, Chew FS. Chronic Recurrent Multifocal Osteomyelitis: review. AJR Am J Roentgenol. 2011;196 (6 Suppl): S87-91.

2. Borzutzky A, Stern S, Reiff A, Zurakowski D, et al. Pediatric Chronic Nonbacterial Osteomyelitis. Pediatrics. 2012;130(5): e1190-7.

3. Watanabe T, Ono H, Morimoto Y, Otsuki Y, et al. Skull involvement in a pediatric case of chronic recurrent multifocal osteomyelitis. Nagoya J Med Sci. 2015;77(3):493-500.

4. Wedman J, & van Weissenbruch R. Chronic Recurrent Multifocal Osteomyelitis. Ann Otol Rhinol Laryngol. 2005;114(1 Pt 1):65-8.

5. Barrani M, Massei F, Scaglione M, Paolicchi A, et al. Unusual onset of a case of chronic recurrent multifocal osteomyelitis. Pediatr Rheumatol Online J. 2015; 13: 60.

6. Psarelis S, Panayotidis I, Nikiphorou E. Osteosclerotic bone lesions of the skull. Arthritis Rheumatol. 2018;70(11):1819.

7. Koizumi H, Utsuki S, Oka H, Shimizu S, Fujii K. Chronic Recurrent Multifocal Osteomyelitis involving the skull. The Internet Journal of Neurosurgery. 2012;8(1).

**Patient Consent Received**

Yes

**Disclosure of Interest**

H. Ailumerab Shareholder of: No conflicts of interest, Grant / Research Support from: No conflicts of interest, Consultant for: No conflicts of interest, Employee of: No conflicts of interest, Paid Instructor for: No conflicts of interest, Speaker Bureau of: No conflicts of interest, C. Aguiar Shareholder of: No conflicts of interest, Grant / Research Support from: No conflicts of interest, Consultant for: No conflicts of interest, Employee of: No conflicts of interest, Paid Instructor for: No conflicts of interest, Speaker Bureau of: No conflicts of interest

**Table 1 (abstract P306).** Cases of CNO/CRMO associated with cranial/facial bones involvement (other than the mandible)

Patient age in years [Ref]	Gender	Cranial bones affected	Other parts of the body	ESR (mm/h)	CRP (mg/d)	Bone Biopsy	Imaging	Treatment offered	Country
10 [our case]	F	Maxillary, Zygomatic & Sphenoid	Radius, Facial nerve	104	7.5	(Maxilla) Fibrous dysplasia, reactive inflammatory fibro-osseous tissue with abscess formation. (Radius) similar chronic Inflammatory findings	Destructive process of the left maxillary, zygomatic, sphenoid bones, & the clivus. Mixed sclerosis and lucency of the distal right radial metadiaphysis.	NSAID	US
11 ^[3]^	M	Occipital	sacro-femoral	41	0.4	Lymphocytic infiltration.	Heterogeneous high intensity area on the right side of the sacrum, osteolytic lesions in the occipital bone with increased signal intensity	NSAID	Japan
12 ^[4]^	F	Frontal & Sphenoid	Tibia, Femur, Ribs	44	NR*	Non-granulomatous active osteomyelitis.	signs of osteomyelitis (destructive, increased signaling, etc).	NSAID	Netherland
12 ^[5]^	F	Frontal, Zygomatic & Sphenoid	Clavicle	49	3.8	Osteoblastic/osteoclastic remodeling. Granulomatous tissue rich of lymphocytes, neutrophils, & histiocytes	Area of osteolysis in the clavicle. Rearrangement & thickening of the cranial bones.	NSAID, bisphosphon-ates.	Italy
19 ^[6]^	M	Neurocranium	NR	95	150 mg/l **	NR	Osteosclerotic lesions in the cranial bones.	Steroid, colchicine & monthly tocilizumab	UK
21 ^[7]^	M	Neurocranium	Tibia	44	1.9	Marrow fibrosis and inflammatory cell invasion.	Thick skull, heterogeneous diploe with outer table erosion appearing as “worm-eaten” spots	NSAID	Japan

* NR: Not reported. ** Each mg/l is equivalent to 0.1 mg/dl

### P307 A review and retrospective case series of paediatric Sjögren’s syndrome from Southern Africa

#### S. Akhalwaya, K. Webb, C. Scott

##### Paediatric Rheumatology, University of Cape Town, Cape Town, South Africa

###### **Correspondence:** S. Akhalwaya

**Introduction:** Paediatric Sjögren’s syndrome (pSS) is an uncommon autoimmune paediatric disease, rarely reported in children in Africa. It remains an important consideration in a child with parotid swelling.

**Objectives:** Here, we present a retrospective case-series of four patients from a paediatric rheumatology clinic in South Africa and discuss some of the difficulties of diagnosis in our context.

**Methods:** We performed a retrospective analysis of patients who attend the Red Cross War Memorial Hospital Paediatric Rheumatology clinic between 2010 and 2019.

**Results:** The four patients diagnosed with Sjögren’s all had varied presentations and disease courses. There were 3 females and 1 male and the ages at diagnosis ranged from 6 to 19 years old. The time to diagnosis was prolonged and ranged from 1 month to 10 years. Two patients with primary pSS presented with extra-articular manifestations of arthritis, abdominal pain and fatigue, followed by dry mouth, dry eyes, parotid swelling and suggestive histopathology. The remaining 2 patients had a secondary pSS due to juvenile onset SLE and tuberculosis respectively.

**Conclusion:** In less resourced settings the diagnosis of pSS is often delayed. Both patients with primary pSS had preceding extra-glandular manifestations. In less resourced settings, infectious diseases may present as pSS and associated rheumatic diseases causing secondary pSS must be considered.

**Patient Consent Received**

No

**Disclosure of Interest**

None declared

### P308 Kikuchi-Fujimoto disease: a case report of a pediatric patient

#### M. Bernardo^1^, A. Lança^1^, C. Quadros^2^, J. Gonçalo Marques^1^, P. Costa-Reis^3^

##### ^1^Infectious Diseases and Immunodeficiency Unit, Pediatrics Department; ^2^Pathology Department; ^3^Pediatric Rheumatology Unit, Santa Maria Hospital, Centro Hospitalar Universitário Lisboa Norte, Lisbon, Portugal

###### **Correspondence:** M. Bernardo

**Introduction:** *Kikuchi-Fujimoto* disease is usually a self-limited cause of lymphadenitis. It is a prevalent disease amongst Asian individuals, but rare in other parts of the world. It usually affects young women, with limited cases described in paediatric literature.

This disease is characterized by focal and tender lymphadenopathy, mostly cervical, accompanied by fever and, less commonly, systemic manifestations. In some patients, it is associated with systemic lupus erythematous.

**Objectives:** -

**Methods:** -

**Results:** A previously healthy 7-year-old boy presented with prolonged fever (10 days), asthenia, anorexia, weight loss, an erythematous and evanescent maculopapular rash, polyarthritis (with large and small joint involvement), cervical lymphadenopathy and hepatosplenomegaly. There was leucocytosis and highly elevated inflammatory markers (maximum erythrocyte sedimentation rate 92 mm/h, maximum serum ferritin level 11 121 ng/ml). Other relevant investigations included: serum triglycerides 264 mg/dl, fibrinogen 326 mg/dl and AST 48 U/L. The *HScore* was 215, which was highly suspicious for macrophage activation syndrome.

Differential diagnosis included systemic idiopathic juvenile arthritis, juvenile systemic lupus erythematous, *Kikuchi-Fujmoto* disease, infectious diseases and malignancy.

Infectious causes were broadly excluded, including virus and intracellular microbes. No changes were seen on the echocardiogram. Antinuclear antibodies and hypocomplementemia were not identified.

A bone marrow aspiration and bone biopsy were performed and the results were unremarkable. A cervical node biopsy was then performed. The pathology exam showed nodal paracortical expansion resulting in general architecture derangement, presence of T lymphocytes in different maturing stages, histiocytes and areas of necrosis and intense karyorrhexis. No granulomas, neutrophil infiltration or microorganisms were found. The histopathological diagnosis was histiocytic necrotizing lymphadenitis and these findings supported the clinical diagnosis of Kikuchi-Fujimoto disease.

The patient was started on oral prednisolone (2mg/kg/day) due to marked systemic symptoms and a high suspicion for macrophage activation syndrome. After the first dose, he remained afebrile, with rapid clinical and laboratorial improvement. Prednisolone was tapered and the patient was regularly followed on the paediatric rheumatology clinic. After more than one year of follow-up, the patient is asymptomatic, without any medication and remains with no anti-nuclear antibodies.

**Conclusion:** This case report shows an atypical course of *Kikuchi-Fujimoto* disease and the diagnostic dilemmas clinicians face when dealing with such presentations. In fact, the co-occurrence of *Kikuchi Fujimoto* disease with reactive macrophage activation syndrome is extremely rare.

Definitive diagnosis of *Kikuchi-Fujimoto* disease is challenging and requires histopathological confirmation, with the finding of histiocytic necrotizing lymphadenitis.

Currently, no therapeutic guidelines exist and most patients do well with anti-inflammatory drugs. Corticosteroids and other immunomodulatory drugs may be useful, particularly in recurrences and more severe cases.

Prognosis is excellent, although some patients may recur and a minority go on to develop autoimmune diseases, mainly systemic lupus erythematosus. This accentuates the importance of appropriate referral for close monitoring.

Finally, *Kikuchi-Fujimoto* disease should be considered in the differential diagnosis of paediatric patients with lymphadenopathy and systemic symptoms. This might help to prevent misdiagnosis and inappropriate treatment.

**Patient Consent Received**

Yes

**Disclosure of Interest**

None declared

### P309 Eosinophilic fasciitis with visceral involvement in a young girl

#### I. Cardoso^1^, M. Rodrigues^2,3^, S. Miranda^4^, B. Santos^5^, F. Aguiar^2,3^, I. Brito^2,3^

##### ^1^Centro Hospitalar de Vila Nova de Gaia/Espinho, Gaia; ^2^Centro Hospitalar Universitário São João; ^3^FMUP, Porto; ^4^Hospital de Braga, Braga; ^5^Centro Hospitalar do Baixo Vouga, Aveiro, Portugal

###### **Correspondence:** B. Santos

**Introduction:** Case report

Eosinophilic fasciitis (EF) is a rare scleroderma-like connective tissue disease with unclear etiopathogenesis. The onset of EF is often sudden, developing over a few days or weeks. The main symptoms are symmetrical, full-circumference swelling and plate-like hardness of the lower limbs and sometimes in the upper limbs. Many cases exhibit systemic symptoms such as fever. En bloc biopsies from the skin to the fascia show marked fascial thickening and inflammatory cell infiltration.

Most patients are in their third to sixth decades, however pediatric cases also have been reported.

A previously healthy 3-year-old girl, presented to her local hospital due to generalized pitting edema, which started in the lower limbs and progressed centripetally over one week, associated with pain, stiffness and hepatosplenomegaly. Her mother and maternal grandfather had vitiligo, and family history was otherwise unremarkable.

Workup showed hypereosinophilia (maximum absolute count 7200/uL), microcytic anemia, hypofibrinogenemia, mildly increased troponins and BNP, hypoalbuminemia and non-nephrotic proteinuria. ESR was normal and CRP was mildy elevated. IgG was increased, with positive ANA (1/640 homogeneous, DFS70 +), negative ANCA, negative myositis-specific antibodies and normal complement levels. There was no evidence of hemolysis or abnormal cells in the blood film. She was transferred to a tertiary hospital for further investigations.

Serologies showed IgM positivity for EBV (confirmed by PCR), all other microbiological studies were negative.

Bone marrow and liver biopsies showed eosinophilia and inflammation. Thoracoabdominopelvic CT revealed mild polyserositis and hepatosplenomegaly. Echocardiography and cardiac MRI were unremarkable.

Painful limb, face and trunk edema persisted, with normal muscle strength. Upper limb MRI showed prominent fascial thickening with increased signal intensity and contrast enhancement, mild diffuse muscle edema like signal; this was most prominent in the forearm than on the arm but also visible in the chest wall, highly suggestive of EF.

Pending biopsy, corticosteroids (CS) were started with immediate clinical improvement and hypereosinophilia resolution. En bloc forearm biopsy performed a week later showed mild dermis fibrosis, subcutaneous tissue hialine sclerosis with lymphohistyocytic infiltrates, and skeletal muscle with mild inflammatory infiltrates; no eosinophils were seen. This was likely due to the timing of biopsy and/or tissue sampling since little fascia was seen.

During follow-up, limb swelling and pain subsided, and skin thickening mostly over the forearms, forehead, feet and trunk became increasingly apparent in the first months, with mild hand contracture. Methotrexate (MTX) was added and these features improved. Raynaud´s is absent, muscle strength is normal. Laboratorial changes and systemic manifestations have subsided. Vitiligo became apparent 4 months later.

15 months later, the patient remains on MTX and low-dose CS with mild skin thickening over the forearms and forehead and no contractures. She maintains normal growth and development.

We report a case of EF with systemic involvement, a rare entity that presents diagnostic challenges and requires multidisciplinary management. Fascial biopsy has classically been considered the diagnostic gold standard, but MRI has been increasingly used for both diagnosis and therapeutic monitoring purposes. Systemic CS remain first-line treatment and MTX has emerged as the leading CS-sparing agent. EBV infection might have been the trigger.

Parental consent was obtained.

**Objectives:** NA

**Methods:** NA

**Results:** NA

**Conclusion:** NA

**Patient Consent Received**

Yes

**Disclosure of Interest**

None declared

### P310 The nail fold capillaroscopic findings of adolescents with anorexia nervosa and bulimia nervosa

#### M. Kasap Cuceoglu^1^, M. Pehlivanturk Kizilkan^2^, E. Sag^1^, S. Akgul^2^, O. Derman^2^, Y. Bilginer^1^, N. Kambur^2^, S. Ozen^1^

##### ^1^Pediatric Rheumatology; ^2^Department of Pediatrics Division of Adolescent Medicine, Hacettepe University, Ankara, Turkey

###### **Correspondence:** M. Kasap Cuceoglu

**Introduction:** The nail fold video capillaroscopy (NVC) is usually performed on patients with microcirculation problems, such as Raynaud's phenomenon. It is also used to distinguish between primary and secondary Raynaud's Phenomenon and identify the scleroderma pattern.

**Objectives:** To describe the acute phase[ES1] nail fold capillaroscopic findings of adolescents with anorexia nervosa (AN) and bulimia nervosa (BN) and to compare these findings with adolescents diagnosed with primary Reynaud’s phenomenon (RP).

**Methods:** We included 17 AN, 2 BN patients and 6 adolescents with primary RP as a control group. The nail fold video capillaroscopy (NVC) data of three study groups were compared. AN and BN patients were classified according to DSM-5. The participants in these two groups were assessed for the presence of Reynaud’s phenomenon/acrocyanosis and the weight loss history (amount and duration), daily calorie intake, vital signs, hydration status, amenorrhea presence and time, the presence and frequency of compensatory behaviors, and drug usage were recorded. Adolescents with primary RP were diagnosed according to ‘International consensus criteria for the diagnosis of RP. All of the participants were thoroughly examined for the presence of additional conditions that might cause peripheral vascular impairment. The initial NVC was performed at the acute phase of AN and BN. For AN acute phase is defined as the period where the nutritional rehabilitation has not yet taken place. For BN, it is defined as the period with excessive vomiting, laxative, or diuretic usage. The NVC analysis was be performed with a digital USB microscopy by an expert blinded to the participant’s clinical status and diagnosis. Eight fingers were evaluated for each patient and average of all fingers’ scores were used as quantitative measures.

**Results:** Among adolescents with AN, 14 of them had enlarged capillaries (capillary diameter 20-50 ) and 6 of them had mild tortuosity (<50%) which were considered as minor capillaroscopic changes. 5 patients had at least one giant capillary (>50), 3 of them had microhemorrhages, 4 of them had capillary ramifications, and 1 of them had capillary disorganization which were considered as major capillaroscopic changes. Two adolescents had capillary loss (6 capillaries/mm). None of them had active or late phase scleroderma findings. In the primary RP group, there were minor findings as five adolescents had mild capillary dilation (capillary diameter 20-50 ), and 5 had mild tortuosity (<50%). However, none had scleroderma (early-active-late phase) findings. Microangiopathy assessment scores revealed no difference between the AN patients with and without RP and primary RP patients. (Table 1) A positive correlation was found between capillary ramification scores and initial daily calorie intake (r: -0.47; p=0.04).

**Conclusion:** Preliminary results of our study suggest that adolescents with AN are at risk for vasculopathy especially during the acute phase of the disease.

**Acknowledgements**

Muserref Kasap-Cuceoglu and Melis Pehlivanturk-Kizilkan contributed equally.

**Patient Consent Received**

No

**Disclosure of Interest**

None declared

**Table 1 (abstract P310).** Measurements of capileroscopy findings according to patient groups

	AN+RF+ n=4	AN+RF- n=13	BN n=2	Primer raynoud kontrol n=6	p
**Microangiopathy score**	0.84 (IQR 0.98)	0.92 (IQR 0.96)	0.71	0.65 (IQR0.23)	0.62
- **Capillary loss score**	0.22 (IQR 0.62)	0.06 (IQR 0.41)	0.15	0.0 (IQR 0.26)	0.58
- **Capillary ramification score**	0.18 (IQR 0.25)	0.13 (IQR 0.25)	0.09	0.09 (IQR 0.18)	0.88
- **Disorganized capillaries score**	0.38 (IQR 0.48)	0.56 (IQR 0.68)	0.46	0.43 (0.32)	0.98
**Enlarged capillaries score (20-50 )**	0.56 (IQR 0.78)	0.31 (IQR 0.72)	0.73	0.58 (IQR 0.42)	0.60
**Giant capillaries score (>50)**	0.06 (IQR 0.22)	0.00 (IQR 0.03)	0.18	0.03 (0.14)	0.14
**Microhaemorrhage score**	0.00	0.00	0.00	0.01	0.67

### P311 Maculopapular rash and fever: a diagnostic challenge to the paediatrician in the COVID-19 pandemic

#### C. Alizzi^1^, F. Cardella^1^, D. Romano^2^, C. Giambrone^2^, A. M. Burgio^2^, M. C. Maggio^2^

##### ^1^Children Hospital “G. Di Cristina”, ARNAS; ^2^University Department PROMISE “G. D’Alessandro” - Children Hospital “G. Di Cristina”, ARNAS, University of Palermo, Palermo, Italy

###### **Correspondence:** M. C. Maggio

**Introduction:** Several clinical conditions can manifest with fever and a maculopapular rash in paediatric age. Although some presentations are benign, others may be medical emergencies, which demand a prompt diagnosis and treatment. Some of the more common causes of fever and maculopapular rash include infectious diseases (Sars-CoV-2, Parvovirus B19; Coxsackie; Epstein-Barr virus infection, Mycoplasma Pneumoniae, etc), hypersensitivity reactions, Autoinflammatory syndromes, vasculitis, Kawasaki disease (KD), autoimmune diseases.

**Objectives:** In the COVID-19 pandemic era these symptoms need a well-organized hospital strategy to rapidly exclude Sars-CoV-2 infection and to distinguish severe and rapidly developing patients.

**Methods:** We evaluated the medical records of children admitted to a paediatric tertiary centre in the years 2020-202, excluding children with suspected or documented COVID-19 infection.

**Results:** We retrospectively identified 21 patients (13M; 9F), age: 0.7-12 years, admitted with the diagnosis of fever and rash and with a definite diagnosis. 10 children had a documented infection (2 Mycoplasma; 2 Parvovirus; 5 EBV; 1 Adenovirus); 3 patients had a KD; 4 had an autoimmune disease; 3 had an Autoinflammatory syndrome, 1 a vasculitis; 1 had a Macrophage Activation Syndrome (MAS). Distribution of the rash, a persistent/vanishing rash, the associated lymphadenopathy did not contribute to the differential diagnosis. Haemoglobin levels were significantly lower in KD (8.3-11.2). CRP was significantly higher in KD (3.23-34) vs autoimmune diseases and Autoinflammatory syndromes. The other laboratory parameters did not contribute to the differential diagnosis, otherwise reached by specific IgM and PCR. In children with clinical signs of suspicion of Autoinflammatory syndromes, the genetic approach permitted to reach the treat-to-target.

**Conclusion:** The numerous viral skin diseases that affect children present a diagnostic challenge to the clinicianIn some situations, viral rash may be difficult to clinically differentiate from nonviral diseases; extensive laboratory evaluation isolates the virus. Otherwise, autoimmune diseases must be excluded and, in this suspicion, the alert must be high to promptly diagnose a MAS. A most severe presentation can hide the first attack of an Autoinflammatory syndrome: hence, the genetic study of these condition is a milestone in the differential diagnosis and avoid a diagnostic delay.

**Patient Consent Received**

Yes

**Disclosure of Interest**

None declared

### P312 Primary Sjögren’s syndrome: description of a monocentric pediatric cohort with focus on the interferon pathway activation

#### C. Morreale^1^, S. Ancona ^1^, S. Volpi^2,3^, P. Bocca^2^, M. Gattorno^1,2^, C. Malattia^1,3^

##### ^1^Clinica Pediatrica e Reumatologia; ^2^Centro Malattie Autoinfiammatorie e Immunodeficienze, IRCCS Giannina Gaslini; ^3^Department of Neurosciences, Rehabilitation, Ophthalmology, Genetic and Maternal Infantile Sciences (DINOGMI), University of Genoa, Genoa, Italy

###### **Correspondence:** C. Morreale

**Introduction:** Primary Sjögren’s Syndrome (pSS) is a chronic autoimmune disease that can involves both exocrine glandular and extra-glandular systems. This condition is rare in children in whom diagnosis may be less straightforward due to the absence of well-established diagnostic criteria. Compelling evidence suggests a significant role for type I interferon (IFN) in the pathogenesis of SS [1].

**Objectives:** 1) To describe clinical and diagnostic features of a cohort of children with pSS;

2) To examine IFN pathway activation in pSS.

**Methods:** We included retrospectively 16 pSS children with disease onset until age 16 years that referred to our Unit. For each patient we collected: clinical data including the disease activity indexes (*ESSDAI -* EULAR Sjögren's Syndrome Disease Activity Index, *ESSPRI* - EULAR Sjögren’s Syndrome Patient Reported Index) [2], laboratory tests and US examination of salivary glands. In addition, expression of 6 type I IFN-related genes (IFI27, IFI44, IFIT1, ISG15, RSAD2, SIGLEC1) was tested in blood samples by standard Real-Time PCR techniques.

**Results:** Almost all patients (15/16) were females. The mean age at onset was 10.08 years (range 5.1-15.7); the mean time interval between onset of symptoms and diagnosis was 2.03 ± 1.6 years; the mean follow up time was 3.36 years. *Table 1* shows the clinical features at disease onset. Rheumatoid factor (RF), antinuclear antibodies (ANA), anti-SSA, anti-SSB were positive in the majority of cases (68.7%, 68.7%, 75%, 62.5% respectively); hypergammaglobulinemia and high erythrocyte sedimentation rate (ESR) were present in 10/16. We found type I IFN pathway activation in 9/14 (64.3%) patients. Schirmer’s test and break-up time (BUT) test were positive in 5/16 (31%) and 4/16 (25%), respectively, while unstimulated whole salivar flow (UWSF) was abnormal in 11/16 (68.7%). Salivary gland ultrasound (SGUS) was positive in 14/16 at onset; labial salivary glands biopsy was performed in 9/16 patients and in all of cases typical histopathological features of SS have been observed. Six out of 16 patients received oral corticosteroid, 10/16 hydroxychloroquine, 8/16 methotrexate, 2/16 sirolimus. The mean ESSDAI score was 6.5 at baseline and 4.7 at last follow-up visit; the mean ESSPRI score was 2.5 at disease onset and 1.5 at last visit.

**Conclusion:** SS is a rare condition in children and diagnosis can be often difficult due to the lack of child-specific diagnostic criteria. This is the first study demonstrating type I IFN signature in pediatric patients with pSS. Our results suggest that type I IFN dysregulation and overexpression might be involved in disease pathogenesis. In this perspective, the inhibition of this pathway could be considered an attractive therapeutic target in pediatric pSS.

**References**

**1.** Marketos N et al. Type I interferon signature in Sjögren's syndrome: pathophysiological and clinical implications. *ClinExpRheumatol.* 2019;37 Suppl 118(3):185-191.

**2.** Seror R et al. Validation of EULAR primary Sjögren's syndrome disease activity (ESSDAI) and patient indexes (ESSPRI) *Annals of the Rheumatic Diseases* 2015;74:859-866.

**Disclosure of Interest**

None declared

**Table 1 (abstract P312).** Clinical features at disease onset

Parotitis	10/16 (62.5%)
Dry mouth	10/16 (62.5%)
Arthralgia/arthritis	7/16 (43.7%)
Dry eyes	6/16 (37.5%)
Lymphadenopathy	6/16 (37.5%)
Caries	4/16 (25%)
Fatigue	4/16 (25%)
Fever	3/16 (18.7%)
Vaginal dryness	2/16 (12.5%)
Associated autoimmune diseases*	2/16 (12.5%) *ALPS-like syndrome

### P313 Spectrum of rheumatic diseases in 4065 children from a single pediatric rheumatology center in India

#### D. B. Pandya, M. Aggarwal, S. Sawhney

##### Pediatric Rheumatology Department, Institute of Child Health, Sir Gangaram Hospital, New Delhi, India

###### **Correspondence:** D. B. Pandya

**Introduction:** There is limited published data about the profile of paediatric rheumatic diseases seen at centers across India^1, 2^.

**Objectives:** We aim to look at the spectrum of diseases at a tertiary level paediatric rheumatology centre (PRC).

**Methods:** This is a retrospective analysis of all the children presenting for the first time to the PRC at Sir Ganga Ram hospital, New Delhi from 1st January 2013 to 31st March 2020. Our data included : place of residence, referral physician, demographic details and final diagnosis updated from their subsequent visits.

**Results:** 4065 children were seen,2189(54%) boys & 1876(46%) girls, median age 8.7 years. 2246(55%)patients were from the local national capital region (Delhi, Noida and Gurugram), 1714(42%) patients from other Indian states and 105(3%) patients from other countries. Patientswere referred by paediatricians(48%), rheumatologists(20%), orthopaedicians(9%), internet(6%), patients’ relatives(3.5%) andother(13.5%) paediatric and adult specialties.

The recent small scale studies from other parts of India3& Africa4 revealed polyarticular & systemic arthritis as a common subtype in JIA, lupus in CTD and Kawasaki disease in primary vasculitis. ERA was found to be the commonest JIA subtype in one latest study from singapore5 while OligoJIA in the study from Canada6.

**Conclusion:** Our unit was established in 2001 and is part of a tertiary level teaching hospital. 45% of patients travelled long distances, the most common referral was from paediatricians. Referrals are predominantly inflammatory rheumatic diseases (49%)followed by infections (9%).Non-inflammatory conditions were strikingly rare at 7%. Additionally, ERA was the commonest category of JIA, lupus the most common CTD and Kawasaki disease the most frequently primary vasculitis. This is perhaps the largest data from a single centre in a large metropolitan area and may not be reflective of other peripheral centres across India.

**Trial registration identifying number:** 1.Review.Indian J Pediatr2010 Sep;77(9):993-6.doi: 10.1007/s12098-010-0134-x. Epub 2010 Sep 3.

The Place of Pediatric Rheumatology in India

Sujata Sawhney 1, Prudence Manners

2. International Journal of Advanced Medical Health & Research (JIPMER)

Pediatric rheumatology: An under-recognized subspecialty in India

Year : 2017 | Volume : 4 | Issue : 2 | Page : 47-53

A k h i l a K a v i r a y a n i 1, S u m a B a l a n 2

3. Journal of Natural Science, Biology and Medicine, 2018

Clinico-epidemiological profile of pediatric rheumatology disorders in Eastern India

Pratap Kumar Patra, Manish Kumar

4. Pediatric Rheumatology volume 18, 2020

Spectrum of paediatric rheumatic disorders at a tertiary hospital in Tanzania

Francis F. Furia, Evance Godfrey, Naomi Mwamanenge & Peter Swai

5. Journal of Clinical Rheumatology April 2020

Juvenile idiopathic arthritis in Southeast Asia: the Singapore experience over two decades

Manasita Tanya & Kai Liang Teh & Lena Das & Sook Fun Hoh & Xiaocong Gao

6. Rheumatology 2020

A new Canadian inception cohort for juvenile idiopathic arthritis: The Canadian Alliance of Pediatric Rheumatology Investigators Registry

Michelle Batthish1,*, Roberta Berard 2,*, David Cabral3, Roxana Bolaria

**Patient Consent Received**

Yes

**Disclosure of Interest**

None declared

**Table 1 (abstract P313).** Spectrum of Rheumatic Diseases in 4065 children presented first time at our centre

Disease (Number of Patients)	Number of Patients(%)
**Juvenile Idiopathic Arthritis-JIA** **Enthesitis-Related Arthritis(578)**>Systemic-JIA(276)>Oligoarticular JIA(186) > Polyarticular-JIA(116)>Undifferentiated-JIA(21)>Psoriatic-JIA(6) ? JIA(21) Inflammatory Bowel Disease-associated arthritis(23)	**1227(30%)**
**Connective Tissue Diseases-CTDs** **Systemic Lupus Erythematosus(208)**>Juvenile Dermatomyositis(61) >Scleroderma(18)>Undifferentiated-CTD(14)>Overlap Syndrome(14)> Mixed-CTD(9)>Sjogren’s syndrome (2)	**326(8%)**
**Primary Vasculitides** **Kawasaki Disease(188)** >IgA-Vasculitis(126)>Behcet’s Disease(19)> Polyarteritis Nodosa(16)>Takayasu Arteritis(5)>Small Vessel Vasculitis(5)	**359(9%)**
**Inflammatory Eye Diseases :** Uveitis(51)>Scleritis(3)>Episcleritis(2)	**56(1.5%)**
**Reactive Arthritides and Infections** Reactive-Arthritis(128),Streptococcal-associated infections(77) including PSRA(10) and ARF(15), Viral infections(62), Transient synovitis of Hip(39), Tuberculosis including poncet’s disease(47) & Bone-Joint infections(17)	**370(9%)**
**Non–Inflammatory Musculoskeletal Diseases** Mechanical conditions(152)>Pain-amplification(58)>Fibromyalgia(52)>Joint Hypermobility(19)>Reflex Sympathetic dystrophy(11)	**292(7%)**
**Miscellaneous Diseases**	**537(13.5%)**
- Ø ?Primary Immunodeficiencies	28
- Ø ?Autoinflammatory Syndromes	34
- Ø Non-specific inflammatory conditions	45
- Ø Suspected IGG4-related disease	5
- Ø Sarcoidosis	9
- Ø Skin Diseases	101
- Ø Haematological disorders including malignancy	76
- Ø Metabolic/Endocrine Disorders including Vitamin D Deficiency	59
- Ø Genetic diseases	38
- Ø Neuromuscular disorders	41
- Ø Secondary Vasculitis	31
- Ø Pyrexia of Unknown Origin	52
- Ø Psychiatric disorders	18
Children with no abnormality detected	**414(10%)**
Children whose diagnosis has not been made due to single visit	**484(12%)**

### P314 Joints in a cold war- a picture essay on two mimics

#### A. Prabhudesai^1^, H. Panwala^2^, A. Khan^1^, N. P. Maldar^1^, R. Khubchandani^1^

##### ^1^Paediatric Rheumatology; ^2^Paediatric Radiology, NH SRCC Children’s Hospital, Mumbai, India

###### **Correspondence:** A. Prabhudesai

**Introduction:** Multicentric Osteolysis Nodulosis and Arthropathy (MONA) (OMIM- #259600; 46 cases/28 families to date) and Camptodactyly-arthropathy-coxa vara-pericarditis (CACP) (OMIM- #208250; 98 cases to date) are extremely rare autosomal recessive syndromic arthropathies which can easily mimic commoner inflammatory rheumatologic diseases owing to their progressive and deforming nature.

**Objectives:** We saw one case of each in a span of 1 month both of whom had been labelled as Juvenile idiopathic arthritis (JIA) and had received escalating immunosuppressive treatment to no avail. Through a picture essay we depict their clinical and radiological features.

**Methods: Case 1:** A 6-year-old developmentally normal girl, weight 15kg (10^th^ centile), height 102cm (<3^rd^ centile) born of a non-consanguineous marriage presented with progressive, deforming, remarkably symmetric arthropathy of large and small joints of upper and lower extremities for 2 years. On examination however the joints were *cold* and her past acute phase reactants were always normal. Besides short stature examination revealed hallux valgus, multiple subcutaneous nodules on elbows, right knee and soles, thickened skin over the limbs, anterior bowing of femora and tibiae and non-inflammatory joints with no other systemic signs. Skeletal survey showed generalized osteopenia in all bones, resorption and osteolysis of carpal and tarsal bones and progressive arthropathy of interphalangeal joints of both hands and great toes.

**Case 2:** A 19-year-old boy weighing 55.3kg (25^th^- 50^th^ centile), height 174cm (50^th^- 75^th^ centile) with a BMI of 18.4, born of non-consanguineous marriage was first found to have bent fingers at the age of 4 years. He subsequently developed swollen large joints at age 7 years and was treated as refractory JIA for 3 years. Examination revealed camptodactyly of the right third, both fifth fingers, all toes and symmetrical cold and bulky swollen joints with no tenderness and full range of motion. X rays of the hips showed short horizontally placed femora and an MRI showed the classic smooth synovial outpouchings which have been characteristically described. Systemic exam was normal. A younger sister is affected with similar but milder features with onset at age 10 years.

**Results: Case 1:** A clinic-radiological diagnosis of MONA was confirmed on whole exome sequencing (WES) that showed a homozygous 2-base pair deletion in exon 1 of the MMP2 gene (c.79_80delGC) that results in a frameshift and premature truncation of the protein 61 amino acids downstream to codon 27 (p.Ala27ArgfsTer61) and is designated likely pathogenic by ACMG classification.

**Case 2:** A clinical suspicion of CACP syndrome was confirmed with a positive WES in the index case that showed a homozygous 2-base pair deletion in exon 7 of the PRG4 gene (c.1910_1911delCT) that results in a frameshift and premature truncation of the protein 9 amino acids downstream to codon 637 (p.Pro637ArgfsTer9) and is designated pathogenic by ACMG classification.

**Conclusion:** The arthropathy of both MONA and CACP can mimic JIA owing to the progressive and deforming nature described in both conditions. The subcutaneous nodules of MONA can be mistaken for calcium deposits (as we did initially) while the combination of camptodactyly with bulky swollen large joints in CACP can mimic Blau syndrome. *Cold* joints, *normal acute phase reactants*, other *syndromic features*, affected *family members* – all elicited on the bedside with some quick radiology can make these conditions ‘single visit spotters’ and prevent diagnostic and therapeutic odysseys.

**Patient Consent Received**

Yes

**Disclosure of Interest**

None declared

### P315 Renal tubular acidosis in pediatric Sjogren’s syndrome: case series from India

#### I. Rajarathinam^1^, L. B^2^, V. K. Gornale^2^, J. Raghuram^3^, A. P. Rao^4^

##### ^1^Pediatrics, Vijaya Children's clinic; ^2^Pediatrics, Indira Gandhi Institute of Child Health; ^3^Pediatrics, Columbia Asia Hospital Whitefield; ^4^Pediatrics, 1st Main Rd, Arekere MICO Layout 2nd stage, 1st Stage, Araka Mico Layout, Arekere, Bengaluru, India

###### **Correspondence:** I. Rajarathinam

**Introduction:** Sjogren's syndrome (SS) is an autoimmune exocrine disorder characterized by Keratoconjunctivitis sicca. Primary SS is a rare pediatric rheumatological disorder with approximately 260 cases being reported in world literature. However, a manifestation which seems to be unique to pediatric is renal tubular acidosis (RTA).

**Objectives:** Renal manifestations in primary SS have been rarely described in literature. We describe the renal manifestations (RTA) in 4 patients with primary SS.

**Methods:** Retrospective chart review of patients with pSS with onset before 18 years of age and complicated by renal tubular acidosis in a tertiary children’s hospital, Bangalore, India were studied.

**Results:** Four female patients with pSS with RTA are being reported. The mean age at presentation was 15 (±1.87) years and mean duration to diagnosis from the disease onset was 18( ±13) months respectively. Symptoms related to RTA in the form of hypokalemic periodic paralysis were noted at the onset of pSS in 3 cases. Keratoconjunctivitis sicca complex was present in two patients. Arthritis was present in all the patients. Antinuclear antibodies were positive in all the patients as were antibodies against extractable nuclear antigens (SS-A and SS-B antibodies). Rheumatoid factor was positive in all the patients. All the patients were started on immunosuppressants including steroids with hydroxychloroquine along with renal supplements.

**Table:** 

	Patient A	Patient B	Patient C	Patient D
Age/Gender	17/Female	15/Female	16/Female	12/Female
Sjogren’s syndrome type	Primary	Primary	Primary	Primary
Age of presentation	14 years	12 years	16 years	11 years
Mean duration to diagnosis	3 years	2 years	2 months	10 months
RTA type	Distal	Distal	Proximal	Proximal
Presenting complaints	Episodic weakness of both legs	Inability to bear weight due to bone pains	Episodic weakness of both legs	Episodic weakness of both legs
Oral symptoms	Yes	No	Yes	No
Ocular	No	No	Yes	Yes
Skin dryness	No	No	Yes	No

**Conclusion:** Renal tubular acidosis can be the presenting feature of pSS. Hence, it is important to screen all children with RTA, particularly adolescents with elevated inflammatory parameters, for presence of antinuclear antibodies (ANA), anti Ro (anti SSA) and anti La (anti SSB), even in the absence of keratoconjunctivitis sicca symptoms.

**Patient Consent Received**

Yes

**Disclosure of Interest**

None declared

### P316 Pulmonary manifestations of the rheumatic diseases in childhood

#### S. Rodríguez Aguayo, E. Faugier Fuentes, M. I. De La Cera Rodriguez, E. R. Mercedes Pérez, N. L. De La Rosa Encarnación, P. P. Ramos Tiñini, H. F. Menchaca Aguayo

##### Paediatric Rheumatology, Hospital Infantil de México Federico Gómez, Ciudad de México, Mexico

###### **Correspondence:** S. Rodríguez Aguayo

**Introduction:** Diffuse lung disease (DLD) in children with rheumatological disease (RD) represents a heterogeneous group of autoimmune disorders that lead to significant morbidity and mortality due to chronic inflammation and immune dysregulation.^(1)^ Data on the incidence and prevalence of pulmonary involvement in children with RD is scarce. ^(2)^

**Objectives:** Describe the experience of the “Hospital Infantil de México Federico Gómez” regarding the pulmonary manifestations of rheumatological diseases in pediatric patients.

**Methods:** We carried descriptive retrospective study of 43 patients with pulmonary manifestations of the rheumatological diseases in childhood in "Hospital Infantil de Mexico Federico Gomez", from March 1st, 2015 to December 31st, 2020. Information included patients´ demographics, pulmonary manifestations, treatment used, and outcomes.

**Results:** We reported 43 patients. 72% of the patients were women. 24 patients (56%) with a diagnosis of systemic lupus erythematosus, six patients (14%) with ANCA-associated vasculitis, five (12%) with systemic sclerosis, four (9%) with juvenile dermatomyositis, three (7%) with overlap syndrome and a patient with Takayasu's arteritis. The mean age of the patients was 12 years (range 6 to 18). The main pulmonary manifestation in the patients was serositis (pleural effusions) in 11 patients (25%), followed by ground glass in seven patients (16%), fibrosis in five patients (12%), nodules in four (9%), two patients with hemorrhage (5%), two with pulmonary embolism, two with pulmonary arterial hypertension, one patient with emphysema and spontaneous pneumothorax, one patient with pulmonary infarction and eight patients (17%) with mixed pattern. The patients received treatment according to the activity of the RD and the type of pulmonary manifestation they presented. All patients received as initial therapy intravenous pulses of methylprednisolone at 30 mg / kg for 3 to 5 doses and subsequently prednisone 2 mg / kg per day with gradual reduction until its suspension. The DMARD most used was mycophenolate mofetil in 25 patients (58%), followed by methotrexate in 10 patients (23%) and azathioprine in 8 patients (19%). 38 patients (88%) also received cyclophosphamide 750 mg / m2 monthly for 6 to 9 doses as multi-target therapy. 7 patients (16%) required biological therapy (5 patients with anti-CD20 and 2 with anti-IL-6), 16 patients (37%) received 2 g / kg of intravenous immunoglobulin and 9 required plasma exchange as a second and third line of treatment. Two underwent autologous hematopoietic stem cell transplantation for refractory disease. 33 patients (77%) are in partial remission. 5 patients died (12%); the main cause was pulmonary hemorrhage (80%).

**Conclusion:** Diffuse lung disease in children with rheumatologic diseases represents a heterogeneous group of severe autoimmune disorders. Pulmonary manifestations of rheumatologic require a multidisciplinary and collaborative approach for optimal diagnosis and management.

**Disclosure of Interest**

None declared

### P317 Phalangeal microgeodic syndrome – a rare vascular acrosyndrome in childhood

#### P. B. Santos^1^, I. Cardoso^2^, S. B. Miranda^3^, F. Aguiar^4,5^, M. Rodrigues^4,5^, I. Brito^4,5^

##### ^1^Rheumatology, Centro Hospitalar do Baixo Vouga, Aveiro; ^2^Paediatrics, Centro Hospitalar Vila Nova de Gaia/Espinho, Vila Nova de Gaia; ^3^Paediatrics, Hospital de Braga, Braga; ^4^Pediatrics and Young Adult Rheumatology Unity, Centro Hospitalar Universitário São joão; ^5^Faculty of Medicine, Porto University, Porto, Portugal

###### **Correspondence:** P. B. Santos

**Introduction:** Phalangeal microgeodic syndrome (PMS) is a rare disease of unknown etiology, which affects fingers and/or toes of children.

**Objectives:** N/A

**Methods:** N/A

**Results: Case Presentation**

The authors describe the case of a 9-year-old girl, who presented to the Pediatric Rheumatology outpatient clinic in November 2020 with painful swelling and cyanosis of all fingers for 3 weeks which had not improved with oral ibuprofen. She had no history of previous COVID-19 and, besides mild IgA vasculitis in 2015, was otherwise healthy. On physical examination, painful spindle-shaped swelling with functional limitation and cyanosis of fingers, associated with chilblain-like lesions were observed. She was started on pentoxifylline 400 + 200 mg/day and, later, nifedipine 0.5 mg/kg/day, and advised to avoid exposure to cold. Five months later she had marked improvement of pain and swelling, maintaining mild cyanosis.

Laboratory workup revealed normal complete blood count, normal erythrocyte sedimentation and C-reactive protein, normal biochemical liver and renal parameters. Immunology tests showed normal IgG, IgA and IgM, positive antinuclear antibodies 1/100 and negative antidsDNA, ANCA, ACPA, rheumatoid factor, anti-ENA, anticentromere and antiphospholipd antibodies. Hand radiographs showed multiple small, round radiolucent images at the edge of the metaphysis of several phalanges. Hand MRI showed bone marrow edema in middle and distal phalanges of all fingers, and distal part of proximal phalanges (more diffuse and prominent in the second and fifth, bilaterally); there were no relevant changes in joints or tendons. These findings are compatible with PMS.

**Conclusion:** This is a rare disease that should be included in the differential diagnosis of patients presenting with vascular acrosyndromes complaints. It can be misinterpreted as an infectious or post-infectious (namely COVID-19-related), inflammatory, or even malignant condition. It usually has a benign course and resolves with conservative treatment. Pediatric rheumatologists should be aware of this entity, as a timely and precise diagnosis prevents further investigations and complications.

**Patient Consent Received**

Yes

**Disclosure of Interest**

None declared

### P318 Paediatric task force for global musculoskeletal health: towards better health for all

#### C. Scott^1^, C. J. Tiderius^2^, M. B. Dobbs^3^, H. E. Foster^4^

##### ^1^Paediatric Rheumatology, University of Cape Town, Cape Town, South Africa; ^2^Department of Orthopaedics Skane University Hospital, Lund University, Lund, Sweden; ^3^Washington University School of Medicine, St Louis Children’s Hospital, Missouri, United States; ^4^Paediatric Rheumatology, Newscastle University, Newcastle, United Kingdom

###### **Correspondence:** C. Scott

**Introduction:** The Paediatric Task Force for Musculoskeletal Health (TF) was established in 2018 in order to foster global co-operation and address gaps in education and care delivery, especially in underserved communities. TF activities have focused on creating a network of regional leaders who work “better together” in order to achieve the aims of the task force.

**Objectives:** To describe recent TF activities and initiatives and report on growth in membership. In addition, we will highlight models of best practice and propose new projects for the future.

**Methods:** The TF has created a network of regional MSK leaders accross the globe. Newsletters and ad hoc communications are used to identify projects which have the potential to influence global MSK health. Task Force working teams are established on a volunteer basis to collaborate on tasks and complete objectives.

**Results:** The TF has grown in terms of membership and influence, including novel partnerships in areas of the globe where engagement with global rheumatology has been suboptimal. The total number of countries included now number 44 (Fig 1). Our ‘call to action’ has been published to address the needs of children with MSK conditions. The regular TF e-newsletter has highlighted regional and country specific updates on rheumatology services, including sharing models of care and education, conference and funding opportunities as well as educational clinical vignettes. TF activities include applications to amend the WHO Essentials Medicine List for Joint Diseases in children and include medications integral to JIA treatment (Triamcinolone, Tocilizumab and Anakinra). Future plans include a bespoke virtual rolling paediatric rheumatology Continuing Medical Education (CME) program catering for the needs of healthcare workers in underserved areas; entitled SPRINT (Sharing Paediatric Rheumatology INternationally Together) as well as an overlapping multi-media awareness and “branding” campaign entitled RUN (Rheumatology Unmet Needs).

**Conclusion:** The TF has developed a robust and enthusiastic network of paediatric MSK caregivers across the globe who are collaborating to develop tangible pragmatic outputs and improve access to better care for children with MSK diseases.

**Patient Consent Received**

No

**Disclosure of Interest**

None declared

### P319 Evaluation of cases with primary and secondary Raynaud’s phenomenon in children

#### M. Sezer, E. Çelikel, F. Aydın, Z. Ekici Tekin, C. Karagöl, S. Coşkun, M. M. Kaplan, B. Acar

##### Pediatric Rheumatology, University of Health Science, Ankara City Hospital, Ankara, Turkey

###### **Correspondence:** M. Sezer

**Introduction:** Raynaud’s phenomenon (RP) is a vasospastic disorder characterized by recurrent transient vasospasm in the small arteries of the fingers and toes that results in triphasic color changes. According to etiology, RP can be primary (%80 of cases) or secondary. There is no associated disease in primary RP. Secondary RP is attributed to an underlying disease, most commonly connective tissue diseases. The difference of secondary RP from primary RP is related to its severity and complications. Antinuclear antibody (ANA) positivity, changes in capillaroscopy and digital ulceration are suggestive of secondary RP. Patients with primary RP may show signs of collagen tissue disease over time.

**Objectives:** The aim of this study is to evaluate the clinical, laboratory and capillaroscopic findings of patients with primary and secondary RP.

**Methods:** Patients who were diagnosed RP in the pediatric rheumatology department between January 2014 and January 2021, were retrospectively reviewed. Demographic data of patients, laboratory parameters and capillaroscopic findings were recorded. Capilleroscopy findings of the patients were classified as normal (capillary count> 7, capillary morphology normal, no capillary enlargement, no avascular area), nonspecific abnormalities (one of them; decrease in capillary number, capillary enlargement, abnormal morphology or microhemorrhage) and scleroderma pattern (presence of giant capillaries or the combination of abnormal shapes with an extremely lowered number of capillaries).

**Results:** Ninety-five patients with RP, of which 68 (71.6%) were females (female-to-male ratio= 2.51:1) were enrolled. The median age was 15.5 years (range 13.9-16.5 years) at the onset of disease. Color changes were biphasic in 69 (72.6%) patients, and triphasic in 26 (27.4%) patients. Twenty-one (22.1%) of the patients had pain and 40 (42.1%) had numbness. Primary RP was present in 84 (88.5%) patients and secondary RP in 11 (11.5%). In patients with secondary RP, the associated disease was systemic lupus erythematosus in 3 (27.2%) patients, scleroderma in 2 (18.2%) patients, juvenile dermatomyositis in 1(%9.1) patient, and juvenile idiopathic arthritis in 5 (%45.5) patients. Arthralgia, arthritis, rash and recurrent fever were significantly more common in secondary RP cases (*p*=0.001, *p*=<0.001, *p*=0.01, *p*=0.035, respectively). Pattern of color changes, age of onset hemogram parameters, acute phase reactants do not show a significant difference in primary and secondary RP cases. ANA was positive in 40 (42.6%) patients. ANA positivity >1/320 was significantly higher in patients with secondary RP (p=0.01). Two patients had scleroderma pattern, 19 patients had nonspecific changes, and 19 patients had normal capillaroscopy findings of 40 patients who could be reached capillaroscopic findings. Capillary irregularity, tortuous capillaries and increased branching were significantly higher in secondary RP cases (*p*=0.015, p=0.015, p=0.003, respectively).

**Conclusion:** In addition to capillaroscopy findings, patients with joint complaints, rash, fever, high titer ANA positivity should be examined in detail in terms of underlying diseases.

**Disclosure of Interest**

None declared

### P320 Identifying barriers to vaccination in immunocompromised children

#### V. Sivaraman^1^, K. Wise^2^, B. Boyle^1^, M. Ardura^1^

##### ^1^Pediatrics, Nationwide Children's Hospital and The Ohio State University; ^2^Specialty Pharmacy, Nationwide Children's Hospital, Columbus, United States

###### **Correspondence:** V. Sivaraman

**Introduction:** Immunocompromised children (ICC) are at increased risk for vaccine preventable infections and complications from these infections. However, for unclear reasons, vaccination of these high-risk patients remains suboptimal.

**Objectives:** Our primary aim was to assess vaccine knowledge, comfort, and vaccination practices among parents of and primary care physicians caring for children with c-SLE and IBD. In order to better identify barriers preventing the vaccination in this patient population, we performed a needs assessment survey.

**Methods:** We created and administered two needs assessment surveys: One was distributed to community primary care providers (PCP) at their monthly hospital meeting followed by an email survey to PCPs not present at the meeting. The second survey was administered to patients with c-SLE or IBD receiving care at the rheumatology and gastroenterology clinics at NCH or their caregivers, between January 1 to August 31, 2018, during routine scheduled visits for disease management. Surveys were completed by patients 18 years or older or by their parent or caregiver. We reviewed electronic medical records of the surveyed patients for demographics, medication exposure, and diagnosis. Responses were recorded in a Redcap database for statistical analysis. Since the study was performed as an internal quality improvement project to improve processes of care, it was deemed exempt from review by the Institutional Review Board.

**Results:** We surveyed 31c-SLE and 26 IBD patients. Patient characteristics and medications are noted in Table 1. Most patients received their vaccines from their primary care provider (PCP) or the Health Department and a minority (16%) stated they usually received vaccines from their subspecialist. The vast majority of patients (96%) felt that their PCP was well informed about vaccines. Notably, 91% of patients reported that their subspecialist discussed vaccines in the past year, most commonly Influenza, Human Papilloma Virus, pneumococcal vaccine and Hepatitis B. Only 2 parents expressed concerns for vaccine adverse effects and triggering a disease flare.

Among the 30 PCP who responded to the survey, 70% had over 20 years’ experience and 50% preferred to provide all vaccines. However, barriers to completing vaccines included: 14 of 16 (85%) stated they did not stock the 23-valent pneumococcal vaccine. Further, PCPs felt “very confident” about providing vaccines in their ICC) only 40% of the time. Practitioners cited not feeling well informed about their patient’s immunosuppressive medications and reported concern for exacerbating the patients underlying illness as the main reason for their lack of confidence.

SLE (n=31) IBD (n=26) All subjects (n=57)

Age: Median (Range) 16 (8-21) 16 (4-22) 16 (4-22)

Caucasian (%) 11 (35) 21 (81) 32 (56)

Other Race (%) 20 (65) 5 (19) 25 (44)

Female Gender (%) 28 (90%) 13 (50) 41 (72)

Medications

Daily Systemic Steroids 18 (58) 0 (0) 18 (32)

Non-Biologic DMARDs 31 (100) 14 (54) 44 (77)

Biologic DMARDs 4 (13) 9 (35) 13 (23)

**Conclusion:** In our survey of patients and PCPs, there was discordance between patient’s feeling confident in their PCP being aware of vaccine recommendations and PCP comfort in vaccinating their ICC patients, due to lack of knowledge of medications and concern for triggering a disease flare. While most ICC received vaccines at their PCP’s office, most of these offices did not carry the 23-valent pneumococcal vaccine and did not routinely recommend the vaccination of other household members. Access to recommended vaccines and lack of education about ICC vaccination schedule remain significant barriers and continued areas for improvement.

**Disclosure of Interest**

None declared

### P321 UK Research priorities in paediatric rheumatology: results of a multidisciplinary consultation and consensus exercise

#### E. M. Smith^1,2^, N. Egbivwie^3^, K. Cowan^4^, A. V. Ramanan^5^, C. E. Pain^6^, on behalf of on behalf of the UK NIHR CRN: Children/Versus Arthritis Paediatric Rheumatology Clinical Studies Group

##### ^1^Institute of Life Course and Medical Sciences, University of Liverpool; ^2^Paediatric Rheumatology Department, Alder Hey Children's Hospital; ^3^Institute of Life Course and Medical Sciences, University of Liverpool, Liverpool; ^4^Katherine Cowan Consulting Limited, Sussex; ^5^Department of Paediatric Rheumatology, University Hospitals Bristol NHS Foundation Trust & Bristol Medical School, Bristol; ^6^Department of Paediatric Rheumatology, Alder Hey Children’s NHS Foundation Trust, Liverpool, United Kingdom

###### **Correspondence:** E. M. Smith

**Introduction:** The evidence base underlying the management of children and young people (CYP) with paediatric rheumatic diseases (PRD’s) is inadequate, with many outstanding questions.

**Objectives:** The main objective of this study was to elicit PRD’s research priorities, through collaborative consultation of patients, carers and healthcare professionals.

**Methods:** This study was led by the UK NIHR CRN Children/Versus Arthritis Paediatric Rheumatology CSG (‘the CSG’) and its Topic Specific Groups (TSG’s)^1. The CSG is^ a multidisciplinary group with strong patient/parent representation, supporting the development of UK clinical studies. Research priority ideas were sought from paediatric rheumatologists, trainees, AHP's, nurses, patients, parents and charities, through on-line surveys and face-to-face meetings. Research ideas were categorised as disease specific or broad/general. They were grouped into sub-themes, duplicates, previously answered questions, and similar submissions combined. A modified on-line Delphi survey was initally used for research priority ranking, followed by an on-line consensus workshop to derive top research priorities.

**Results:** The initial consultation yielded 304 research priority ideas; 25% from patients/parents, 22% from the CSG, 18% from TSG’s, 13% from AHPs, 11% from trainees, 11% from nurses. 55 disease specific and 37 broad/general research priorities were voted upon in Delphi survey 1. A top 11 general broad research priorities were agreed. The top 10 disease specific priorities were discussed at the online Delphi workshop, and two online surveys held to determine their final ranking (see Table).

**Table:** 

Broad/general priorities	Disease specific priorities
**1.** How can we predict disease course (including flares, remission, treatment response) in individual patients? **2.** Understanding and predicting the long term outcomes of PRD’s (physical, psychological, social). **3.** What are the long term effects and safety profile of drugs including biologics and biosimilars in PRD’s? **4.** How can tolerability of methotrexate be improved for CYP?* **5.** What is the best treatment for each individual patient? (right drug, right patient, right time?)* **6.** How best do we assess and manage the impact of PRD’s through psychological support/intervention? **7.** Why do patients with PRD’s get fatigue and how do we best assess and manage it?	**1.** Trial of anti-TNF vs JAK inhibitors treatment JIA. **2.** Interventional trial of novel agents **and** standard of care treatment vs standard of care (methotrexate) using a targeted treatment pathway in newly diagnosed polyarticular JIA. **3.** Establish clinical trials in JDM to evaluate the effect of novel treatments (e.g. JAK inhibitors), biologics (e.g. TNF blockers) and other treatments currently used to improve disease control and steroid sparing. **4.** How can we best recognise CYP at risk from persistent pain, to ensure early intervention? **5.** Clinical trials of biological and other agents (e.g. bisphosphonates) in CRMO. **6.** Trial of mycophenolate versus methotrexate in localised scleroderma. **7.** In CYP with lupus, does implementation of a treat to target management strategy, as opposed to standard care lead to improvements in disease activity and damage, steroid sparing and improving quality of life and fatigue?

Top 7 are shown due to space limitations. *equal number of votes.

**Conclusion:** UK consensus-based PRD research priorities are presented, underpinned by collaboration with patients, carers and healthcare professionals, helping to guide funding bodies to improve the JSLE evidence base in PRD’s.

**Patient Consent Received**

No

**Disclosure of Interest**

None declared

### P322 Dress syndrome- ‘a masquerade in pediatric rheumatology clinic’

#### H. T. Reddy^1^, A. P. Rao^2^, S. Shamarao^3^, B. Shenoy^4^

##### ^1^DNB Paediatrics Resident doctor - 2nd year; ^2^Consultant in Paediatric Rheumatology; ^3^HOD -Paediatric Intensive Care Unit; ^4^HOD- Department of Paediatrics, Manipal Hospital, Hal Road, Bangalore, Bangalore, India

###### **Correspondence:** H. T. Reddy

**Introduction:** INTRODUCTION: Drug reaction with eosinophilia and systemic symptoms (DRESS) is a severe, cutaneous adverse reaction (SCAR) characterized by a spectrum of systemic manifestations with multiple organ involvement which can masquerade connective tissue disorders like Systemic Lupus Erythematosus (SLE) and vasculitis like Kawasaki disease (KD) due to overlapping manifestations.

**Objectives:** To analyze the clinical presentation, laboratory profile and outcomes of 4 patients with DRESS syndrome seen over a decade.

**Methods:** Retrospective data collection of children with DRESS syndrome seen in pediatric rheumatology clinic in between January 2009 and January 2021 was done and the data was analysed.

**Results:**

**Table:** 

	PATIENT A	PATIENT B	PATIENT C	PATIENT D
History	3 year 3 month old female child was diagnosed to have atypical febrile seizure, started on phenytoin .	13 year old male child who had an episode of left focal seizure was diagnosed to have neurocysticercosis and was started on phenytoin .	15 year old female child who was diagnosed to have small vessel vasculitis and was started on dapsone .	14 year old male child, with childhood epilepsy with breakthrough seizures was started on lamotrigine
Duration between starting of new drug and symptoms:	2 weeks	3 weeks	3 weeks	2 weeks
Clinical features:				
High spiking fever	+	+	+	+
Generalised maculopapular rash	+	+	+	+
Generalised lymphadenopathy				
Hepatosplenomegaly	+	+	+	+
Neurological involvement	+	+	+	+
Decompensated liver disease		+ (2 episodes of GTCS)	+(icterus, ascites, pedal edema)	
Investigations:				
Total leukocyte	Leucopenia	Leucopenia	Leukocytosis	Leukocytosis
Differential count	Normal	Neutropenia	Neutrophilia	Eosinophilia
Platelets	Normal	Normal	Thrombocytopenia	Normal
Elevated ESR	+	+	+	+
Elevated CRP	+	+	+	+
Elevated Ferritin	+	+	+	+
Elevated Triglycerides	+	+	++	+
Elevated transaminases	++	+	++	+
Hypoalbuminemia	-	-	++	-

The mean age of patients was 11½ years (+/-5years). There was no sex predisposition. The clinical details of the patients are given in Table 1. Dermatological involvement (generalized erythematous rash) along with generalized lymphadenopathy were seen in all the patients. 3 patients out of 4 (75%) had hepatic dysfunction (hypertransaminasemia) and 1 patient had acute fulminant hepatitis. One of the patients had seizures as a manifestation of neurological involvement. The mean duration from the time of exposure to development of symptoms was 2½ weeks (±1 week). The offending drug was phenytoin in 2 patients, dapsone and lamotrigine in one each. All patients fulfilled the RegiSCAR criteria for diagnosis of DRESS and were categorised as probable cases based on validation score. Only 25% of patients had evidence of eosinophilia and 75% had other haematological abnormalities which include leucopenia, thrombocytopenia, and neutrophilia. The mean duration of hospital stay was 10 days. The mean Hb-10.4g/dL^.^, mean leukocyte counts were 9010 cells/cu mm, mean ESR was 33.5 mm/hour, mean CRP-94.7mg/dL, mean serum triglyceride levels were 400mg/dL and mean AST/ ALT was 706.7/571.2 IU/L. Management included withdrawal of offending drug and corticosteroids (intravenous followed by oral).

**Conclusion:** DRESS can have overlap with regards to clinical presentation with more common pediatric rheumatological disease including connective tissue disorders like Systemic lupus erythematosus and vasculitis like Kawasaki disease. It is extremely important to enquire into the history of drug intake in any febrile child who comes with history of skin rashes with presence of visceral organ involvement as this could be DRESS.

**Patient Consent Received**

Yes

**Disclosure of Interest**

None declared

### P323 Frequency of clinical manifestations in Mexican children with rheumatic diseases

#### A. Villarreal-Treviño^1^, I. Peláez-Ballestas^2^, F. García-Rodríguez^3^, E. Faugier-Fuentes^4^, S. Mendieta-Zerón^5^, G. Reyes-Cordero^6^, J. Guadarrama-Orozco^4^, N. Rubio-Perez^1^ on behalf of COLaborativa de Investigación en Beneficio de la Reumatología Infantil (COLIBRI)

##### ^1^Universidad Autónoma de Nuevo León, Monterrey; ^2^Hospital General de México, Ciudad de México; ^3^Hospital Universitario "Dr. José E. González", Monterrey; ^4^Hospital Infantil de México, Ciudad de México; ^5^Instituto de Seguridad Social del Estado de México y Municipios, Toluca; ^6^Hospital Infantil de Especialidades del Estado de Chihuahua, Chihuahua, Mexico

###### **Correspondence:** A. Villarreal-Treviño

**Introduction:** Pediatric rheumatic diseases (PRD) are a heterogeneous group of disorders characterized by inflammation of connective tissue, especially the joints, blood vessels and skin. Variability on clinical manifestations presents regarding the PRD itself and between regions and ethnicity.

**Objectives:** To describe the frequency of clinical manifestations in Mexican Children with Juvenile Idiopathic Arthritis (JIA), Juvenile Dermatomyositis (JDM), and Systemic Lupus Erythematosus (SLE).

**Methods:** A multi-center cross-sectional study was carried out, analyzing the data provided by a survey to patients with JIA, JDM, and SLE, and their caregivers between April and November 2019. Data were obtained through interviews with the participants and/or review of the clinical records of the patients. The study used descriptive statistics with frequencies and measures of central tendency and dispersion.

**Results:** We recruit 200 participants, 109 (54.5%) with JIA, 28 (14%) with JDM and 63 (31.5%) with JSLE. Median age was 13 (IQR 10-15) years, most of them female (134, 67%). The predominant symptoms during the course of PRD were musculoskeletal (161/193, 83.4%), while articular were the most prevalent in JIA (109, 100%), cutaneous (28, 100%) and myositis (27, 96%) in JDM; and cutaneous (39/56, 69.6%), and hematological (29/56, 51.8%) in JSLE (Table).

**Table:** 

	Total 200 n (%)	JIA 109 n (%)	JDM 28 n (%)	JSLE 63 n (%)	P value*
Arthritis	144 (74.6)	109 (100)	10 (35)	25 (44)	< 0.001
Neuropsychiatric	15 (8)	0 (0)	0 (0)	15 (27)	< 0.001
Pulmonary	4 (2)	0 (0)	0 (0)	4 (7)	0.007
Cardiovascular	14 (7)	4 (3.7)	1 (4)	9 (16)	0.010
Gastrointestinal	8 (4)	1 (1)	3 (11)	4 (7)	0.028
Hematological	32 (17)	3 (3)	0 (0)	29 (52)	< 0.001
Ocular	1 (0.5)	1 (1)	0 (0)	0 (0)	0.679
Muscular	28 (14)	0 (0)	28 (100)	0 (0)	< 0.001
Renal	15 (8)	0 (0)	0 (0)	15 (24)	< 0.001

**Conclusion:** Frequency of clinical manifestations in our population were similar to those reported elsewhere with some differences in proportions. As expected, there are substantial characteristics related with each diagnosis.

**Patient Consent Received**

Yes

**Disclosure of Interest**

None declared

## e-Poster viewing: Psycho-social aspects and rehabilitation

### P324 Results of the PedsQLTM 4.0 generic core scales in children with juvenile idiopathic arthritis

#### L. Bohmat^1,2^, A. Fadieieva^1^, N. Shevchenko^1,2^

##### ^1^Department of Rheumatology and Comorbid States, SI Institute for Children and Adolescents Health Care of NAMS of Ukraine; ^2^Department of pediatrics № 2, V. N. Karazin Kharkiv National University, Kharkiv, Ukraine

###### **Correspondence:** L. Bohmat

**Introduction:** According to the “Treat to target” strategy, the aim of JIA management in children is both clinical remission/low disease activity and reaching the best indicators of the patient`s quality of life that it determines his social status in the future^1^. However, there is no optimal tool for assessment and control quality of life in this category of patients.

**Objectives:** To identify the quality of life in patients with JIA according to the results of the PedsQLTM 4.0 Generic Core Scales taking into account the variant of the disease.

**Methods:** 41 children with JIA were investigated ( 22 with polyarthritis – 53,66%, 14 with oligoarthritis – 34,17%, and 5 with undifferentiated – 12,19%), 3 - 17 years old (26 girls and 16 boys), 37 were treated by methotrexate, 5 – by sulfasalazine. The duration of the disease was 40,22± 6,21 month. Disease activity was determined corresponded to JADAS27, and functional state to CHAQ. Validated for Ukraine questionnaire PedsQLTM 4.0 Generic Core Scales was used for quality life evaluation during the last week. The questionnaire consists of 4 scales (physical, emotional, social, and school functioning), 33 questions, and has 4 age versions (for children from 2 to 18 years). The results were evaluated according to the method of the Likert scale^2^ (the highest score of 100 points demonstrates the best quality of life). The survey was conducted by children from 8 years and older and parents of children up to 8 years.

**Results:** It was found that the activity of JADAS27 in the children with polyarthritis was 11,20 ± 7,04, with oligoarthritis – 9,80 ± 4,23. High degree of JIA activity was in 12 patients with polyarthritis and 9 persons with oligoarthritis. Functional status (CHAQ) was estimated as 0.23 ± 0.17 indicating minimal functional impairment (0.22 ± 0.14 in children with polyarthritis and 0.18 ± 0.12 with oligoarthritis).

The overall level of quality of life in patients with JIA was 50,11 ± 6,20. This indicator had a strong relationship with the value of the score on the JADAS27 (r=0,784) and a weak relationship with the duration of the disease (r=0,272), p<0,05.

The overall level of quality of life in patients with polyarthritis was lower (38,41 ± 3,40) than with oligoarthritis (54.1 ± 2,81; p<0,05).

The physical scale of the questionnaire showed the highest results among other scales (corresponding to the minimum functional impairment according to CHAQ) – 76,21 ± 3,41; in children with polyarthritis – 62,71 ± 8,61 and with oligoarthritis – 80,51 ± 6,20. The lowest indicators were the results of subscales of emotional and school functioning (58.35 ± 3,67 and 44,24 ± 6,11 respectively), the same in children with polyarthritis (57,61 ± 7,34 and 42,42 ± 6,21) and with oligoarthritis (68,21 ± 8,71 and 64,60 ± 4,51 respectively). At the same time, the results of social functioning were quite high in both groups (82,45 ± 4,11).

**Conclusion:** The indicators of the quality of life of children with JIA were reduced, especially according to the data on the emotional and school functioning subscales. The results of the PedsQLTM 4.0 Generic Core Scales allow assessing the additional social and emotional sphere of children's life, which is important for their active socialization. This problem requires further study to understand possible actions to improve the situation in the cohort of children with JIA.

**Disclosure of Interest**

None declared

### P325 JIA phenotype in a multi-ethnic community: cohort study of 993 patients

#### A. Bouraoui, C. Fisher, J. Glanville, D. Sen

##### Adolescent and Young Adult Rheumatology Department, University College London Hospitals, London, United Kingdom

###### **Correspondence:** A. Bouraoui

**Introduction:** Juvenile idiopathic arthritis (JIA) is the most common rheumatic disease in childhood, predominantly affecting Caucasians. [1] London is the most diverse region in the UK with 40% of residents Black, Asian, Minority Ethnics (BAME).[2]

**Objectives:** In this single centre retrospective cohort study, we aim to understand the epidemiological and clinical characteristics of JIA focused on disease phenotype and ethnicity.

**Methods:** A retrospective cohort analysis was conducted within the department of Adolescent and Young Adult (AYA) rheumatology at University College London Hospital. We extracted demographic, clinical and laboratory data. Household incomes were estimated using the Office of National Statistics database. [3] R-Software was used for analysis. We calculated median and interquartile range (IQR) for continuous variables and frequency and percentage for categorical variables. Chi-squared-test and Wilcoxon rank-sum were used for Comparisons between Caucasians and BAME groups P values < 0.05 were considered significant and < 0.01 as highly significant.

**Results:** We identified 1001 patients with JIA, 993 fulfilling International League of Associations for Rheumatology Criteria. The age range was 14-75 with a median of 22 years (IQR:19-27) and Female/ Male ratio of 1.66 (621/372). 15% of patients (148) were referred directly to the AYA rheumatology from primary care and 85% transitioned from Paediatric Rheumatology clinics. 70% of patients were Caucasians (702), 27% (271) BAME, 10% Asian, 5% Afro-Caribbean and undocumented 2% (20). The majority of patients lived in above average income areas and 9% (90) in a low-income area. The predominant subtype was Enthesitis Related Arthritis at 22% (223) of all patients, followed by rheumatoid factor negative Polyarticular (RF-PJIA) 16% (159), Extended oligoarticular (EOJIA) 15% (157), RF positive Polyarticular (RF+PJIA) 10% (97), psoriatic (PsJIA) 7% (67), systemic onset (SOJIA) 6% (61), persistent oligoarticular (POJIA) in 5% (49) and undifferentiated JIA in 17%. 13% (131) of patients have a history of anterior uveitis and 17% were positive for antinuclear antibody (ANA). The median disease onset age was 10 years (4-13) and median disease duration 14 years (10-20). Comparison between Caucasians and BAME groups revealed a higher rate of POJIA in the BAME group (8% vs 4%, p:0.006), whereas a higher rate of EOJIA and RF+PJIA was seen amongst Caucasians (18% vs 11%, p:0.007) and (12% vs 4%, p:0.003) respectively. Despite similarities in disease duration and onset, greater diagnostic delay was seen in the BAME group compared to Caucasians (12(IQR 8-14) vs 10 (IQR3-13) months, p<0.0001). 70% of our BAME cohort were originally from London (p<0.0001). Estimated household income showed higher income prevalence in Caucasians 60% vs 3% of the BAME group (p <0.0001).

**Conclusion:** JIA is commonest in Caucasians in keeping with the literature**.** We show a difference in disease phenotype between age groups, ERA more common in AYA compared to POJIA in paediatrics cohorts.[4] BAME subjects were from poorer backgrounds with greater diagnostic delay. Our observations raise the question of referral bias and socioeconomic factors influencing access to specialist services amongst BAME populations potentially affecting the metrics of JIA.

*References*

*1. Saurenmann et al. (2007). Epidemiology of Juvenile Idiopathic Arthritis in a Multiethnic Cohort. Arthritis and rheumatism. 56. 1974-84. 10.1002/art.22709.*

*2.* *https://census.ukdataservice.ac.uk*

*3.* *https://www.ons.gov.uk*

*4. Thierry S, Fautrel B, Lemelle I, Guillemin F. Prevalence and incidence of juvenile idiopathic arthritis: a systematic review. Joint Bone Spine. 2014 Mar;81(2):112-7.*

**Disclosure of Interest**

None declared

### P326 Economic impact of juvenile idiopathic arthritis

#### S. Jiménez Hernández^1^, F. García Rodríguez^1^, A. V. Villarreal Treviño^1^, V. A. Barrientos Martínez^2^, A. A. Gamboa Alonso^2^, M. De la O Cavazos^2^, L. Ochoa Alderete^2^, N. Rubio Pérez^1^

##### ^1^Pediatric Rheumatology; ^2^Pediatrics, Hospital Universitario "José Eleuterio González", Monterrey, Mexico

###### **Correspondence:** F. García Rodríguez

**Introduction:** Juvenile Idiopathic Arthritis (JIA) is a heterogeneous group of diseases, which are characterized by chronic arthritis before the age of 16, and require prolonged treatment and follow-up. The treatment is multidisciplinary, with multiple appointments, laboratory studies and medications, this approach represents a high economic cost.

**Objectives:** The objective of this study is to systematically review the relevant literature on the socio- economic burden on direct and indirect costs of the JIA.

**Methods:** The PRISMA recommendations were followed, an unlimited search was made in different databases from January 2000 to July 2019, updated on May 2020. Reviewers worked independently and in duplicate, with good agreement (kappa 0.61) . The reported costs were converted from the original currency to US dollars (USD) for the year of publication, and inflation adjustment was made as of December 2019.

**Results:**

**Table:** 

REFID	Original	Total Cost		Direct costs related to medical care		Direct costs not related to medical care		Indirect Cost	
		Reported	Ajusted*	Reported	Ajusted*	Reported	Ajusted*	Reported	Ajusted*
278	EUR 2012	$31,546	$44,832	$14,508	$20,618	$8,323	$11,828	$8,715	$12,385
423	CAD 2009	$1,063	$1,122	$674	$711	$34	$37	$355	$375
705	EUR 1999	$3,471	$5,591	$1,821	$2,933	$78	$126	$1,571	$2,530
19‡˟	GBP 2016	$15,980	$23,117						
283	EUR 2012	$30,034	$42,682						
132	EUR 2014			$3,631	$5,450				
614	EUR 2018	$4,663	5,312	$4,172	$4,752				
171^	USD 2016	$1,130	$1,204	$969	$1,033				

15 articles were found, mostly from Europe. Total costs ranged from USD $1,122 to $44,832 / year, direct costs related to health services varied between USD $711 to $20,618/ year, direct non-health related costs varied from $37 to $11,828/ year, and indirect costs in 5 studies ranged from USD $139 to $ 12,385 / year.

**Conclusion:** There is great variability between studies when defining costs and how to measure them. It is necessary to standardize the reports and generate information from developing countries to obtain a more accurate analysis of the impact of the disease.

**Disclosure of Interest**

None declared

## e-Poster viewing: e-health and digital health applications

### P327 Global adaptation of a pediatric rheumatology educational resource relies on team collaboration

#### M. A. Alessi^1^, T. Tanner^2^, D. O'Leary^3^, M. Chan ^4^

##### ^1^King Fahad Hospital of The University, AL Khobar, Saudi Arabia; ^2^Children's Hospital at Montefiore, Bronx, New york, United States; ^3^UCD Centre for Arthritis Research, Dublin, Ireland; ^4^BC Children's Hospital and University of British Colombia, Vancouver, Canada

###### **Correspondence:** M. A. Alessi

**Introduction:** Pediatric rheumatology (PR) is under-resourced internationally. High clinical workload leads to challenges in developing, maintaining and regularly updating sustainable educational resources locally. Few clinical faculty and small numbers of trainees also pose challenges to structuring high-level education at the level of a PR trainee. Existing resources are often region-specific and cannot be easily applied to international contexts.

The PR Learning Modules were created to serve as a fellowship training curriculum at the University of British Columbia in 2015 . There has been interest in adapting these for PR trainees around the world with the input of global expertise.

**Objectives:** To update and adapt the existing evidence-based PR learning modules for use by the international PR community through a global collaboration; and to make it accessible through a sustainable platform online free of charge.

**Methods:** A virtual working group was established based on discussion of the project at a PR medical education workshop at the 2020 ACR meeting. Collaborators are pediatric rheumatologists and PR trainees with different experiences, levels of training, and cultural backgrounds from Canada, USA, Ireland and Saudi Arabia. Two collaborators have medical education training. We continue to recruit interested contributors and meet regularly to update and globalize established learning modules. Modules were updated based on a framework of ongoing user feedback for clarity, content and relevance; current literature; and guided by the Textbook of Pediatric Rheumatology (8th Edition).

Globalization was based on existing frameworks for internationalizing medical school curricula. Content was rephrased to ensure clear, simple language as many future users may have English as a second or additional language. Other adaptation strategies include: removing cultural identifiers, using generic drug names, and including reflection and conversation prompts around practice in specific healthcare systems and cultures. These working strategies were discussed and agreed upon by the team.

Our strategies were refined after we piloted our updating and adaptation processes with an initial review of 3 learning modules (out of 37). Each member did an initial review of one module, provided feedback, updated content and learning objectives, as well as references. Proposed changes were then independently reviewed by each group member; if universally agreed upon, they were accepted prior to a monthly meeting. Discrepancies were discussed by the group with all final accepted changes requiring 75% consensus.

All work has been facilitated through online platforms including monthly virtual meetings, cloud-based file sharing, and real-time group file editing.

**Results:** To date the working group has met five times and fourteen topics have been reviewed and updated. Project completion is expected in the coming months with plans for dissemination thereafter. Our collaboration allows team members to challenge each other professionally and academically, share practice and cultural insights, and develop both clinical and cultural competency.

**Conclusion:** Our virtual working group is an example of a successful cross-cultural collaboration to develop a high- quality evidence-based educational resource to address the needs of diverse stakeholders. Making this resource freely available may help address inequities in PR education worldwide. Evaluation of this resource and its applicability in different contexts will further inform the development of educational materials relevant to other healthcare systems and cultures.

**Disclosure of Interest**

None declared

### P328 The shift to pediatric rheumatology telemedicine practice impacts the ability to assess and document critical JIA outcome data: a survey within the PR-COIN network

#### F. Barbar-Smiley^1^, S. Akoghlanian^1^, M. E. Ryan^2^, J. G. Harris^3^, S. S. Vora^4^, R. Pooni^5^, D. R. Bullock^6^, S. M. Tse^7^, T. C. Lee^8^, Y. I. Goh^7^, on behalf of Pediatric Rheumatology Care Outcomes Improvement Network (PR-COIN)

##### ^1^Pediatric Rheumatology, Nationwide Children’s Hospital, Columbus; ^2^Pediatric Rheumatology, Allergy, &Immunology, University of Minnesota, Minneapolis; ^3^Pediatric Rheumatology, Children's Mercy, Kansas City; ^4^Pediatric Rheumatology, Atrium Health Levine Children’s Hospital, Charlotte; ^5^Rheumatology, Stanford Children’s Hospital, Palo Alto; ^6^Pediatric Rheumatology, Allergy, & Immunology, University of Minnesota, Minneapolis, United States; ^7^Rheumatology, The Hospital for Sick Children, Toronto, Canada; ^8^Pediatric Rheumatology, Stanford Children's Health, Palo Alto, United States

###### **Correspondence:** F. Barbar-Smiley

**Introduction:** The COVID-19 pandemic disrupted the traditional in-person healthcare delivery model, prompting a shift to telemedicine to ensure continuity of care for pediatric rheumatology patients. The change to virtual practice affected healthcare provider’s assessments of disease activity in patients with juvenile idiopathic arthritis (JIA) as they were unable to perform hands-on physical assessments. Understanding the impact of this shift is critical to help address any care gaps that are faced during virtual visits for patients with JIA.

**Objectives:** The objectives of the survey were four-fold: a) understand the impact of the switch from in-person to telemedicine visits from the healthcare provider perspective; b) identify the barriers and facilitators to collecting critical data elements that are important in monitoring JIA disease activity and outcomes; c) identify tools that providers are using during their telemedicine visits to perform disease activity assessments; and d) examine the impact of the telemedicine healthcare delivery on clinical research.

**Methods:** A cross-sectional survey sent to members from all Pediatric Rheumatology Care and Outcomes Improvement Network (PR-COIN) centers (n=21) with total number of targeted respondents of 121. The survey was sent out for completion between 08/17/2020 - 09/02/2020. Quantitative responses were analyzed using descriptive statistics. Qualitative responses were analyzed by content and theme.

**Results:** Survey ersponse rate was 98% (n=119) 90% fully completed. Most respondents (99%) indicated that they documented six critical data elements [CDE] (physician global assessment, patient global assessment, active joint count, morning stiffness, arthritis-related pain, and completion of uveitis screen) in 75% of telemedicine visits. Most respondents (74%) indicated that they documented active joint count over 70% of the time, while 30% of respondents reported barriers to documenting active joint count such as inability to palpate joints and the inability to visualize all joints on virtual examination. Identified barriers to assessment and visit documentation included challenges with assessing joint disease activity and platform technical issues. Ten percent of the respondents reported they often forgot to document CDE during telemedicine visits, indicating that setting up automated reminders in their electronic medical records may help with increasing their likelihood of documentation. A few centers reported having processes to assist with the collection of patient data in advance of the visit, such as pre-visitquestionnaires and planning. The ability to perform research activities was significantly impacted with only 37% of centers reported participating in research activities via telemedicine, and 29% reported their ability to consent patients via telemedicine visits.

**Conclusion:** There are multiple barriers and facilitators to conducting successful clinical visits as well as performing clinical research over telemedicine. Our data suggests variation in telemedicine practice and process across centers, as well as within each center, reflecting the need to standardize the process of telemedicine visits. Given that a portion of patients with JIA will likely continue to be serviced over telemedicine post-pandemic, teams need to adapt their existing practices to continue providing quality care and integrating clinical research over this platform where appropriate.

**Patient Consent Received**

No

**Disclosure of Interest**

None declared

### P329 The possibilities of telemedicine in pediatric rheumatology in the Russian federation

#### A. Fetisova^1^, E. Alexeeva^1,2^, T. Dvoryakovskaya^1,2^, R. Denisova^1^, I. Kriulin^1,3^, A. Babayan^4^, G. Vershinin^4^

##### ^1^Rheumatology, National Medical Research Center of Children's Health, Moscow, Russian Federation; ^2^Pediatric, Sechenov First Moscow State Medical University, Moscow, Russian Federation; ^3^Pediatric, Sechenov First Moscow State Medical University, Moscow, Rwanda; ^4^National Medical Research Center of Children's Health, Moscow, Russian Federation, Moscow, Russian Federation

###### **Correspondence:** E. Alexeeva

**Introduction:** The past decade in rheumatology has seen tremendous innovation in digital health technologies. With a limited number and distribution of pediatric rheumatologists, telemedicine has been proposed as one way to meet this mission, yet the adoption of this modality has been slower than expected. The current global COVID 19 pandemic has triggered a paradigm shift in many centers to use telemedicine more widely

**Objectives:** We report on the implementation of a telemedicine program in Russian Federation for the evaluation and treatment of patients with rheumatic diseases. Provide an overview of the use of telemedicine consultations in rheumatology department at the Federal Center.

**Methods:** The telemedicine consultations department was established on the basis of the Federal Center on 7^th^ of September in 2018. It is equipped with all the necessary equipment for organizing telemedicine consultations in real time, as well as holding deferred consultations on documents and selecting for hospitalization.

**Results:** In 2020 433 requests were received (122 - emergency, 81 - urgent, 230 - planned), rheumatic disease were excluded in 48 patients, 196 patients were received the recommendations of investigations and correction of therapy, 189 patients were admitted (18 of them in the ICU).

In 2021 (4 months) - 199 applications (52- emergency, 46 - urgent, 101- planned), rheumatic disease were excluded in 10 patients, 86 patients were admitted in the rheumatology department (5 of them in the ICU).

In 2021 (from January to April) the number of applications increased 3 times in comparison with 2020.

From April 2020 to April 2021, 44 initial applications and 19 repeated applications were received with a referral diagnosis of COVID 19, multisystem inflammatory syndrome. COVID 19 were confirmed in 10 patients. Other diagnoses were exposed: 6 - sepsis, 2 - PID, 1 - thrombophilia, 1 - relapse of leukemia, 5 - infection and 2 - deaths. 17 patients were admitted: sJIA were diagnosed in 8 patients, SLE -2, large B-cell lymphoma-1, colitis-1, Kawasaki-4, polyarthritis-1.

There were patients from the remote regions: 30 were from Sakhalin Republic, 25 - Republic of Bashkortostan, 25- KhMAO, 28 -Orenburg Region, 14- Amur Region.

**Conclusion:** Telemedicine is becoming more widespread, and the number of applications is increasing every year. We report the successful use of this service to assess the solution to the issue of hospitalization of children with rheumatic diseases for the initiation of DMARD in a region with limited access to rheumatological care.

**Disclosure of Interest**

None declared

### P330 Video-based, marker-less gait-analysis in children and adolescents based on transfer learning with deep neural networks

#### A. Behl^1^, S. Mayer^1^, A. Bevot^2^, D. Pauser^1^, D. Papies^1^, S. Hansmann^3^

##### ^1^Faculty of Economics and Social Sciences, University of Tuebingen; ^2^Department of Pediatric Neurology; ^3^Center for Pediatric Rheumatology, University Children's Hospital Tuebingen, Tuebingen, Germany

###### **Correspondence:** S. Hansmann

**Introduction:** Juvenile idiopathic arthritis (JIA) as a chronic inflammatory joint disease impairs body function and limits physical activity and social participation. Differences in gait pattern and functional limitations are small and quantitative recording and analysis of movement is currently only possible with expensive motion capture systems (1).

**Objectives:** This is the first study to investigate the potential and feasibility of video-based, marker-less 2D gait-analysis with deep neural networks to detect changes in gait mechanics in children and adolescents with JIA.

**Methods:** Sagittal plane gait videos from 40 individuals were obtained with standard consumer cameras on varying backgrounds. Using the DeepLabCut toolbox (2), pixel locations of six anatomical landmarks of each lower limb on randomly selected video images were labelled.

A body part detector called DeeperCut (3) was trained to track the location of the individuals’ anatomical landmarks in the videos. It is based on a deep Residual Network (ResNet) adapted to predict the probability for each marker being located at a specific pixel. We initialized ResNet with parameters pre-trained on ImageNet (4), a large image recognition dataset, and leveraged transfer learning to adapt the pre-trained model with our 750 images manually labelled with anatomical landmarks. Based on the position of the landmarks and thereby defined alignment of the individual segments, their relative position (knee and ankle joint angles), velocities and accelerations over time was determined.

This information can be used subsequently for gait analysis in videos without motion capture markers.

**Results:** Videos from 38 individuals (12 with JIA, median age 11.7 years (range 3.8-16.1); 26 healthy, 9.9 years (2.8-17.3)) were used for training and two test videos for subsequent evaluation of the marker detection and angle estimation.

Percentage of Correct Parts (PCP) (5) was applied as metric to evaluate the performance of our proposed approach. PCP measures a part prediction as correct if the pixel distance between the predicted part location and the manually labelled part is less than a fraction of the limb length in pixels. Despite the large age range of participants, we can correctly track pixel location of 99.6% of the body parts in the test videos according to the PCP metric with threshold set to 0.1.

Furthermore, we evaluated our joint angle estimations by computing the mean absolute error (MAE) between angles estimated from predicted landmarks and those from manually labelled markers. For the front limb, MAE for the knee and ankle was 3.9° and 3.2°, for the rear limb, MAE was 6.4° and 3.5° respectively.

**Conclusion:** Our approach represents a marker-less, robust, low-cost and adaptable method for gait analysis at any age. It will therefore help to understand gait in a broader spectrum of disease courses and can efficiently increase our knowledge of the functional gait and movement abnormalities in individuals with JIA, thereby improving further outcomes.

**Literature**

1. Kuntze et al. Gait Adaptations in Youth with Juvenile Idiopathic Arthritis. Arthritis Care Res. 2020; 72(7):917-24.

2. Mathis et al. DeepLabCut: Markerless Pose Estimation of User-defined Body Parts with Deep Learning. Nat Neurosci, 2018; 21(9):1281-9.

3. Insafutdinov et al. DeeperCut: A Deeper, Stronger, and Faster Multi-person Pose Estimation Model*.* Computer Vision – ECCV, 2016; 9910:34–50.

4. Russakovsky et al. ImageNet Large Scale Visual Recognition Challenge*.* Int J Comput Vision, 2015; 115(3):211-252.

5. Eichner et al. 2D Articulated Human Pose Estimation and Retrieval in (Almost) Unconstrained Still Images. Int J Comput Vision, 2012; 99(2):190-214.

**Disclosure of Interest**

None declared

### P331 SAIApp: a web application of the university of palermo dedicated to children with autoinflammatory syndromes

#### R. Pirrone^1^, G. Corsello^2^, I. Pirrone^2^, M. C. Maggio^2^

##### ^1^Department of Engineering; ^2^University Department Promise “G. D’Alessandro”, University of Palermo, Palermo, Italy

###### **Correspondence:** M. C. Maggio

**Introduction:** The recent epidemic strongly evidenced the necessity of reorganizing physician work, patients’ access to clinics and patient support, especially for chronic diseases.

In this field, a good strategy must consider the integration between department pediatricians, multidisciplinary specialists and pediatricians in private practice. Besides, adequate standards of care must be ensured especially for children affected by chronic diseases, as Autoinflammatory syndromes.

**Objectives:** These children need to monitor clinical manifestations, attacks-free intervals, treatment adherence and response, onset of short- and long-term complications. This surveillance is a fundamental step to guarantee the best and personalized therapeutic choice, to update the therapeutic plan and to monitor adverse events.

In this framework, a cutting-age strategy must adopt web applications, running on personal devices as tablet or smartphone.

**Methods:** SAIApp is a web application written in Python using the Django framework. The app’s data layer relies on a relational DBMS implemented in SQLite, while its presentation layer makes use of Bootstrap to guarantee full responsiveness even for small-screen devices. The mobile version has been designed just as a “webapp” that is a mobile app that simply shows a frame where HTML browsing still takes place. This choice is due to the very low computational load involved in SAIApp: it does not use any sensor of the mobile device, and no particular time constraints are required for the user interaction thus allowing for the use of web GUI widgets.

The application considers three main actors: the patient or her/his family, the department pediatrician, and the pediatrician in private practice that has been appointed by the patient as her/his reference physician. All of them are registered users of the application but with different roles and grants on the database.

The patient can only specify her/his health status by answering a series of simple Yes/No questions about the presence of some symptoms like fever, headache, vomit, constipation, and so on. Moreover, the patient can provide a qualitative evaluation of the overall health status through an easy GUI made by a list of emoj expressing increasing levels of satisfaction. Each health status report has a timestamp, and it is the main source of information to be stored in the database.

**Results:** The department pediatrician acts as the administrator of the system. He registers the new users belonging to the other two groups and inserts the clinical data. The department pediatrician fills the records related to the first diagnosis, to therapy along with the results of clinical examinations. Finally, the department pediatrician owns a GUI to perform search in the patients list according to several criteria. The result can be exported as a csv file for further analysis.

**Conclusion:** The pediatrician in private practice supports the work of the department pediatrician as she/he can insert and search for all the clinical data, related only to her/his patients. In this way, we want virtually extend the assistance to children with Autoinflammatory syndromes and guarantee adequate standards of care.

**Disclosure of Interest**

None declared

## e-Poster viewing: Pain, fatigue, disease experience and quality of life

### P332 Applicability and effectiveness of the back and forth schoolbooklet, a shared self management instrument for young children with juvenile idiopathic arthritis (JIA) at school

#### J. Cappon^1^, M. van Rossum^2^, E. Littooij^1^, M. van der Leeden^1^

##### ^1^Reade Center for Rehabilitation and Rheumatology; ^2^Amsterdam Rheumatology and Immunology Center | Reade, Amsterdam, Netherlands

###### **Correspondence:** J. Cappon

**Introduction:** Young children with JIA have to cope with pain and fatigue during schooldays, facing problems with writing, climbing stairs, physical education and playing outside.^1^ They need to develop age-appropriate self management skills, encouraged by their parents and teachers in a therapeutic alliance with the health professional team.^2^ For this purpose a personal plan for managing pain, fatigue and limitations is made during outpatient rehabilitation treatment in Reade by the physiotherapist and the occupational therapist and noted in a shared management tool, called the “Back and Forth Schoolbooklet”(B&FS). This tool contains diary pages with 1) a colour-in puppet for expressing location and amount of pain, 2) spaces for suggested alternatives for limited activities, 3) feedback spaces for comments of parents and teachers and 4) a self-evaluation scale of general well-being for the child. Children, parents and teachers are educated and instructed how to use the booklet in the context of the child's personal pain and fatigue management plan at school. Structured evaluation of the use of the instrument is necessary to improve its applicability and effectiveness during rehabilitation treatment.

**Objectives:** To study the feasibility, defined as practical and experienced applicability and effectiveness, of the B&FS.

**Methods:** Pilot feasibility study with a mixed-method design. Parents, teachers, therapists and children with JIA(4-8 years) were invited to fill in questionnaires after using the booklets in school during their rehabilitation treatment. Practical applicability was assessed by multiple choice questions on duration and frequency of use. Used diary items and pages were counted in returned booklets. Experienced applicability and effectiveness were assessed by open-ended questions. Topics were experienced barriers, facilitators and benefits using the mentioned items of the booklets. Practical applicability was analysed descriptively. Atlas-ti8 was used for analysing and coding the answers on the open-ended questions using a thematic approach.

**Results:** Eight children used the booklets. Six booklets were returned. Six parents of six children, four therapists and four teachers signed informed consent and answered questionnaires. Practical applicability: Five children used booklets for a period of 2 to > 12 week, almost every day. One child stopped in the first week. Counting diary pages confirmed every day or every second day appropriate use of the colour-in puppet and spaces for parents and teachers. Experienced applicability: Identified themes were: child-friendly, easy and providing a clear guide for managing symptoms in the daily school situation. Themes as: daily obligation, unwillingness of the child, lack of motivation or time of the parents or teachers and insufficient instruction illustrated experienced barriers for the use of the booklet. Effectiveness: Identified themes: 1) Children express themselves better about feelings of pain and fatigue, 2) Parents and teachers appreciate more insight into how the child feels and 3) Teachers feel provided with guidance in the interaction with the child 4) Children feel more secure to express themselves at school and 4) Parents are more relaxed about the school situation.

**Conclusion:** The Back & Forth School booklet is a feasible shared management instrument to support a personal plan for managing JIA symptoms of young children in the school situation. A less rigid daily routine and sufficient instruction can improve the experienced applicability.

**Disclosure of Interest**

None declared

### P333 Perceived health and social life in chronic pediatric inflammatory rheumatic diseases

#### R. El Haddad^1,2,3^, K. El Asmar^2^, C. Hascoet^3^, L. Rossi-Semerano^3,4^, P. Dusser^3,4^

##### ^1^Department of Epidemiology and Statistics, Faculty of Public Health Lebanese University, Fanar, Lebanon; ^2^Institut National de la Santé et de la Recherche Médicale UMR-1178, University Paris-Sud, Le Kremlin Bicêtre; ^3^Réseau Rhumatismes Inflammatoires Pédiatriques (RESRIP), Bourg-La-Reine; ^4^Rheumatology-Pediatrics Department and CeRéMAIA (Reference Centre for Autoinflammatory Diseases and Inflammatory Amyloidosis), APHP - Bicêtre Hospital, Le Kremlin Bicêtre, France

###### **Correspondence:** P. Dusser

**Introduction:** Chronic inflammatory rheumatic (CIR) diseases may affect perceived health and social life in children and adolescents.

**Objectives:** The aim of this study was to identify the (1) sociodemographic/clinical characteristics, (2) ongoing medications and (3) school layouts associated with perceived health status and social life and to evaluate its evolution over time in patients included in RESRIP (Réseau pour les Rhumatismes Inflammatoires Pédiatriques), a French healthcare network for children/adolescent with CIR diseases.

**Methods:** Patients >3 year-old enrolled in RESRIP (2013-2020) were included in the study. At enrollment visit (EV): sociodemographic/clinical characteristics, ongoing medications were inquired from the patients or their parents. Actions to be implemented by RESRIP (paramedical and school level) were also collected. Perceived physical health and social life were reported using a standardized questionnaire set up by RESRIP for the follow-up of patients, at EV and every 6 months. A “well-being score” was then calculated ranging from 0 to 18, with 0 corresponding to absolute well-being. Patients were followed up from their inclusion till June 2020.

**Results:** A total of 406 patients were enrolled and followed up for 36 months in average: 205 juvenile idiopathic arthritis, 68 connective tissue diseases, 81 auto-inflammatory diseases and 52 other diseases. The well-being score did not differ between the groups of diseases. At EV, use of homeopathy, need for implementation of hypnosis or psychological support and need for adjustment of school exams were associated with worse well-being score, and this, whatever the diagnosis. Over time, well-being score improved significantly by 0.04 score unit every 6 months (95%IC [-0.06; -0.03] p<0.001).

**Conclusion:** Well-being appears to be associated more with the impact of chronic illness than with the type of disease itself. Chronic pain, psychological suffering or functional failure are probably factors associated with this uneasiness, as described in the literature. This result underlines the importance of a healthcare network such as RESRIP who is responsible for the overall care of patients. In the future, it would be interesting to compare these results with patients with CIR not included in a healthcare network.

**Patient Consent Received**

Yes

**Disclosure of Interest**

None declared

### P334 Influence of psychological well-being on participation in physical activity and pain in children with juvenile idiopathic arthritis compared to controls

#### B. Mahler^1^, S. Esbersen^1^, J. J. Lomholt^2^, M. Nørgaard^3^, T. Herlin^1^

##### ^1^Department of Pediatrics, Aarhus University Hospital, Aarhus N; ^2^Department of Psychology and Behavioral Sciences, Aarhus University, Aarhus; ^3^Department of Physiotherapy, Aarhus University Hospital, Aarhus N, Denmark

###### **Correspondence:** B. Mahler

**Introduction:** Modern targeted therapy has caused a paradigm shift in the management of patients with juvenile idiopathic arthritis (JIA). This has allowed a motivating approach to promote physical activity (PA) and participation in sport aiming to reduce functional impairment.

**Objectives:** To examine how pain and psychological well-being influence the participation in PA in children with JIA and healthy controls.

**Methods:** JIA patients were consecutively recruited from the outpatient clinic and healthy age-related controls were recruited from different public schools in the Aarhus area during 2016 to 2018. During 7 consecutive days JIA patients and controls were asked to complete a pain app daily, called How-R-You, scoring pain intensity and pain location on a VAS score (0-10). The children and one of their parents completed questionnaires regarding anxiety symptoms in Spence Children’s Anxiety Scale (SCAS), wellbeing in WHO-5 Wellbeing Index (WHO-5)), and positive and negative mood in The Positive and Negative Affect Schedule (PANAS). Moreover, a questionnaire about PA and exercise was completed. Exclusion criteria for patients were JIA in remission off medication, co-morbidity, or non-Danish speaking children or parents. The study has been approved by the Danish National Committee on Health Research Ethics and the Danish Data Protection Agency (1-16-02-297-16, 1-10-72-153-17).

**Results:** 57 children with JIA and 174 age-related healthy children completed the study. Of the children with JIA 56.1% participated always in school-based physical education (edu-PA, compared to 90.4% of the healthy controls (p<0.001). Using How-R-You, JIA patients registered 65 days with no pain out of 464 days recorded (14%) compared to 363 out 1177 (30.8%) in the control group (p<0.0001). The mean total VAS, mean VAS/day with pain and mean of maximal VAS registered for JIA patients was significantly higher than for controls (all p<0.001).

Among JIA patients, a significantly higher negative affect score in PANAS was found compared to controls (mean 22.1 vs. 18.9, p=0.009). However, no difference was seen in the positive affect score, nor the scores in total SCAS and WHO-5 well-being index. A significantly higher positive affect score and lower negative affect score were found in children participating in club sports or always participating in edu-PA, regardless of study group. However, the anxiety score and WHO-5 well-being index were not significantly different between JIA patients whether or not participating in edu-PA or club sport, as it was for the control subjects. In controls recording zero days with pain, a significantly higher positive affect score, lower negative affect score, lower anxiety score (SCAS-C) and higher WHO-5 were found, compared to those with pain. However, in the JIA group we observed no significant difference in these scores, regardless the presence of pain.

**Conclusion:** By using the pain app How-R-You, we found that subjects with JIA recorded pain more often and more intense than healthy age-related controls. Psychological well-being was positively related to participation in educational PA and club sport in both study groups. Psychological well-being was also positively related to pain-free days in the control group, but not in the patient group.

**Disclosure of Interest**

None declared

### P335 Developing, implementing and evaluating a team-based one-year support-program for children newly diagnosed with JIA and their parents

#### K. Mördrup, J. Granhagen Jungner, K. Palmblad, E. Wiedenhielm Broström, C. Bartholdson

##### Department of Womens and Childrens Health, Karolinska Institutet, Stockholm, Sweden

###### **Correspondence:** K. Mördrup

**Introduction:** When a child is diagnosed with childhood rheumatism, such as Juvenile Idiopathic Arthritis (JIA), it is often a turbulent time with a lot of anxiety and stress, not only for the child but also for the parents.^1^ Information given directly after the notification of the diagnose can be difficult to comprehend and remember. ^1,2^ The child and parents often need repeated information to understand the disease to ensure compliance with treatment.^3^ Therefore, we anticipate that it is important that the child and the parents establishes a contact in care enabling close communication and supportive care.^4^

**Objectives:** To improve quality of care for children who is newly diagnosed with JIA and their parents by developing and implementing a team-based one-year support-program. Furthermore, the objectives are to investigate satisfaction levels of children and parents by comparing experiences between care received during the support-program and standard care.

**Methods:** The support-program has been developed by our rheumatology team including physicians, registered nurses (RNs), physiotherapists, occupational therapists and an external organizational coach. Children diagnosed with JIA and their parents are offered to participate in the support- program from the time of diagnose and one year ahead. During the program the children and their parents are invited six times to structured appointments with the team. The appointments are person-centered, thus flexible according to individual needs. For example, one visit may focus on physical activity and is led by the physiotherapist and the RN. Continuous telephone contact with the RN on demand, is scheduled over the whole year. To measure satisfaction-levels with care the participants (children/parents) in the support-program and children/parents who have received the standard care are invited to answer a study-specific questionnaire. For children younger than 8 years old the parents are invited to answer the questionnaire. For children 8-17 years old both the child and the parents are invited to answer the questionnaire.

**Results:** So far, totally 42 children and their parents have been included in the program, none have declined to participate. Of these, 14 have completed the support-program and 8 children and 6 parents have answered the questionnaire. Four children and 6 parents who have received standard care have answered the questionnaire enabling comparative data. Preliminary results show that most participants included in the support-program where satisfied with the care. Answers to the question regarding “Do you know where to turn to for help?” indicate that the support-program assists the child in everyday life. Almost all children and their parents (93%) in the support-program answered that they did know where to turn to for help, compared to 50% in the group of children and parents receiving standard care. Moreover, almost all parents (87%) in the program answered that they had received information about how the disease can affect their child’s everyday life, compared to 40 % in the group receiving standard care.

**Conclusion:** There seems to be a great interest in and need for the support-program. Participants in the support-program are satisfied with the team-based person-centered care during the first year ahead of diagnoses. Overall, children and parents included in the support-program are more satisfied than children and parents receiving standard care.

**Patient Consent Received**

No

**Disclosure of Interest**

None declared

### P336 Incidence and impact of gastrointestinal symptoms in juvenile fibromyalgia

#### A. Naim^1^, S. Arrigo^2^, E. Drago^1^, I. D'Elia^1^, L. Marasini^1^, C. Morreale^1^, C. Malattia^1,3^

##### ^1^Clinica Pediatrica e Reumatologia; ^2^Gastroenterology and Digestive Endoscopy Unit, IRCCS Giannina Gaslini; ^3^Department of Neurosciences, Rehabilitation, Ophthalmology, Genetic and Maternal Infantile Sciences (DINOGMI), University of Genoa, Genova, Italy

###### **Correspondence:** A. Naim

**Introduction:** Juvenile fibromyalgia syndrome (JFS) is a chronic disabling condition characterized by widespread musculoskeletal pain associated with several somatic symptoms including fatigue, non restorative sleep and headaches. Although gastrointestinal (GI) symptoms are common in fibromyalgia (FM), there is a lack of studies primarily focusing on GI disturbances in children with JFS.

**Objectives:** 1) To describe prevalence of GI symptoms and functional gastrointestinal disorders (FGID) in patients with JFS

2) To explore the impact of GI involvement on the global JFS severity.

**Methods:** We included 26 patients (24 females, 2 males, median age 15) diagnosed with JFS according to the 2010 criteria of the American College of Rheumatology. 23 patients had a primary JFS while the remaining had secondary JFS. Gastrointestinal symptoms were explored using a specifically devised questionnaire. The GI manifestations were classified according to Rome IV criteria of FGID. A 0-10 visual analogue scale was used to quantify the severity of GI symptoms. The global JFS severity was assessed using the J-FIMAR which includes comprehensive patients self-report questionnaires and numerical rating scales to quantify musculoskeletal pain, fatigue, headache, sleep quality, physical function, psychological state, health-related quality of life, and satisfaction with illness course. Correlations between GI symptoms and global JFS severity were tested using the Spearman’s rank order correlation coefficient (rs).

**Results:** Twenty out of 26 (77%) patients complained of GI symptoms and 11 (42%) perceived them as invalidating. None of the 3 patients with secondary JFS showed GI involvement. Gastrointestinal reflux disease (GERD) was present in 11/26 (42.3%) patients. The incidence of FGID is reported in Table 1.

Significant correlations were found between the intensity of GI involvement and the Widespread Pain Index (WPI) (rs = 0.54; p=0.046) and the musculoskeletal pain severity (rs = 0.52; p0.045). FGID were not associated with higher rates of sleep problems, chronic fatigue, headache and comorbid anxiety, and/or depression. Thirteen out of 26 (50%) patients presented a stool consistency indicating moderate/severe constipation according to Bristol stool charts; 10/26 (38.4%) referred inconstancy in daily number of evacuations.

**Conclusion:** GI symptoms are common features in primary JFS patients and may impair patient’s activities of daily living. Irritable bowel syndrome and functional dyspepsia are the most frequently reported FGID. In our patients GI symptoms significantly correlated with the extent and intensity of musculoskeletal pain. Our results confirm the link between FM and the gastrointestinal system and highlights the need of a multidisciplinary approach for optimal management of JFS.

**Disclosure of Interest**

None declared

**Table 1 (abstract P336).** See text for description

FGID	Prevalence (N=26)	Prevalence (%)
Irritable bowel syndrome	10	38.4
Functional dyspepsia	8	30.7
Functional nausea	3	11.5
Abdominal migraine	3	11.5
Functional dysphagia	3	11.5
Functional Constipation	2	7.7
Functional abdominal pain	2	7.7
Other	3	11.5

### P337 Health-related quality of life and function in adults with juvenile idiopathic arthritis – comparison with adult-onset rheumatic diseases

#### F. Oliveira-Ramos^1,2^, A. M. Rodrigues^3^, F. Martins^4^, A. Melo^1^, F. Aguiar^5^, L. Brites^6^, S. Azevedo^7^, A. C. Duarte^8^, J. Melo-Gomes^9^, C. Furtado^10^, A. F. Mourão^11^, G. Sequeira^12^, I. Cunha^13^, R. Figueira^14^, M. J. Santos^2,8^, J. E. Fonseca^1,2^

##### ^1^Pediatric Rheumatology Unit, Rheumatology Department, Centro Hospitalar Universitário Lisboa Norte; ^2^Faculdade de Medicina, Universidade de Lisboa; ^3^Centre for Chronic Diseases (CEDOC), Nova Medical School; ^4^Sociedade Portuguesa de Reumatologia; ^5^Centro Hospitalar Universitário São João, Lisbon; ^6^Centro Hospitalar e Universitário de Coimbra, Coimbra; ^7^Unidade Local de Saúde do Alto Minho, Ponte de Lima; ^8^Hospital Garcia de Orta, Almada; ^9^Instituto Português de Reumatologia, Lisbon; ^10^Hospital Divino Espírito Santo, Ponta Delgada; ^11^Centro Hospitalar Lisboa Ocidental, Lisbon; ^12^Centro Hospitalar Universitário do Algarve, Faro; ^13^Centro Hospitalar do Baixo Vouga, Aveiro; ^14^Hospital Central do Funchal, Funchal, Portugal

###### **Correspondence:** F. Oliveira-Ramos

**Introduction:** We currently know that health related quality of life (HRQoL) of Juvenile Idiopathic Arthritis (JIA) patients in adulthood is lower than for the general population. However, less is known how HRQoL of adult JIA patients compare to patients with other rheumatic diseases with adult-onset.

**Objectives:** To evaluate functional disability, mental health, fatigue and HRQoL among different JIA categories in adulthood and to identify differences with adult-onset rheumatic diseases.

**Methods:** Cross-sectional analysis nested in a cohort study of JIA patients registered in the Rheumatic Diseases Portuguese Register (Reuma.pt). Functional disability (Health Assessment Questionnaire - Disability Index (HAQ-DI)), mental health symptoms (Hospital Anxiety and Depression Scale (HADS)), fatigue (Functional Assessment of Chronic Illness Therapy – Fatigue Scale (FACIT-F) and health-related quality of life (EuroQol-5D (EQ5D) and Medical Outcomes Study 36-item Short Form (SF-36)) were compared among JIA categories. Adult patients with polyarticular JIA course and with enthesis related arthritis (ERA) JIA were compared respectively to patients with rheumatoid arthritis (RA) and adult-onset spondylarthritis (SpA), matched for gender and age with adjustment for disease duration and activity.

**Results:** A total of 585 JIA patients were included, mean disease duration 22.8 ± 12.7 years, 38% had active disease and 8.7% had severe disability at the time of the last visit. 10.5% and 4.8% presented anxiety and depression symptoms, respectively, with no differences among all JIA categories. Fatigue was less reported in ERA patients when compared to undifferentiated JIA patients (FACIT-F score 45 [39; 49.5] vs 34 [25; 40]; p=0.045). Persistent oligoarthritis and ERA patients scored higher in EQ5D and in physical component of the SF-36 when compared to other JIA categories. When we compared JIA to adult-onset rheumatic diseases, we found less disability in polyarticular course JIA when compared to RA (median HAQ of 0.25 (0; 1) vs 0.63 (0.13;1.13); p<0.001) and in ERA JIA when compared to adult-onset SpA (median HAQ 0 (0;0.44) VS 0.75 (0;1,5); p=0.041). Adult-onset SpA patients had more depression and anxiety symptoms than ERA patients (respectively 14.8% vs 0%; p=0.003 and 21.3% vs 9%; p=0.002). JIA patients had less fatigue symptoms when compared to control patients with adult-onset diseases (FACIT-F score 42 [33.5; 47] vs 40 [29; 47.5]; p=0.041 and 45 [39; 49.5] vs 41 [29; 46]; p=0.010). JIA patients with polyarticular course had better scores on EQ5D and all domains of SF36, than control patients with RA.

**Conclusion:** Persistent oligoarticular JIA is the category with less functional impairment and with better HRQoL in adulthood. Overall, polyarticular and ERA JIA have lower functional impairment and better quality-of-life in adulthood than adult-onset RA and SpA.

**Patient Consent Received**

Yes

**Disclosure of Interest**

None declared

### P338 Understanding the role of glucocorticoid medication on the health-related quality of life of families: a secondary analysis of qualitative data in patients with JIA, JSLE and JDM

#### S. Singhal^1,2^, L. Roper^3^, E. M. D. Smith^1,2^, F. Sherratt^3^, S. Gorst^3^, P. Livermore^4^, C. E. Pain^1,2^, M. W. Beresford^1,2^

##### ^1^Institute of Life Course & Medical Sciences, University of Liverpool; ^2^Department of Paediatric Rheumatology, Alder Hey Children's NHS Foundation Trust; ^3^Institute of Population Health, University of Liverpool, Liverpool; ^4^Great Ormond Street Hospital, London, United Kingdom

###### **Correspondence:** S. Singhal

**Introduction:** Glucocorticoid medication remains the mainstay of treatment for a wide range of conditions in paediatric rheumatology, including JIA, JSLE and JDM. They offer potential for rapid clinical improvement, but frequently lead to side effects that impact significantly upon health-related quality of life (HRQOL). This study seeks to better understand how this commonly used medication can affect HRQOL for children and young people (CYP) and their parents.

**Objectives:** To explore the role of glucocorticoid medication on the health-related quality of life of CYP with JIA, JSLE and JDM.

**Methods:** The study undertook secondary analyses of three primary qualitative datasets *(N=60)*, comprising semi-structured interviews exploring patient/parent perspectives in JIA *(N = 21)*, JSLE *(N = 24)* and JDM *(N=15)*. Families had experience of oral, intraarticular, intravenous and intramuscular glucocorticoid administration route. Thematic analysis was undertaken using a combination of inductive and deductive approaches. This involved repeat reading of the transcripts to gain familiarity with the datasets, generation of initial codes, identification of preliminary themes and iterative refinement of themes.

**Results:** The thematic analysis revealed six broad categories of impact on HRQOL: *1. Negative body image 2. Change in mood/cognition 3. Mobility & the body 4. Participating in daily life 5. Experiences around medication administration route 6. Tolerability of side effects.*

Both parents and young people identified a harmful effect on body image from glucocorticoid medication. Side effects such as facial swelling, weight gain and increased appetite were repeatedly identified as the most severe. This influenced self-esteem and perception from peers. The effect became more evident around puberty and was noted by some families to be of particular relevance in adolescent girls: *“At her age, she was worried about what people thought of her.”*

Changes in mood & cognition included difficulty concentrating, hyperactivity and mood swings. Parents reported difficulty in managing behavioural effects, particularly in younger children.

Families reported glucocorticoid treatment led to a rapid improvement in mobility and pain, *“After he was put on the steroid drip, he went, “Look mummy, I can run,” and I just cried.”* However, families reported toxicity from the physical effects of treatment e.g. poor immunity, skin changes and muscle weakness.

Glucocorticoid medication changed the way CYP participated in daily life, including social relationships. Families reported poor body image and feelings of isolation related to “being different”, resulting in withdrawal from peers.

Some effects were related to particular administration routes, e.g. anxiety with general anaesthesia. Tolerability related to duration and effectiveness of glucocorticoid medication in controlling the primary condition. Many families felt rapid onset of action was tempered by limited duration of effect, with concern about long-term toxicity.

**Conclusion:** Glucocorticoid medication has a diverse and marked impact on HRQOL for patients with JIA, JSLE and JDM. The benefits result in rapid improvement of pain and mobility, but the varied psychosocial impact of glucocorticoid toxicity remains concerning. Potential disparity between patient and physician priorities for glucocorticoid medication in relation to HRQOL needs to be addressed in order to improve acceptability and tolerability of glucocorticoid medication in patients.

**Disclosure of Interest**

None declared

### P339 Which item of the checklist individual strength-8 best reflects fatigue in juvenile idiopathic arthritis?

#### S. A. Musterd^1^, A. Vroegindeweij^1^, S. L. Nijhof^2^, M. M. Nap-van der Vlist^2^, E. M. van de Putte^2^, N. M. Wulffraat^1^, J. F. Swart^1^

##### ^1^Department of Pediatric Rheumatology/Immunology and Infectious Diseases; ^2^Department of Pediatrics, Wilhelmina Children's Hospital, University Medical Center Utrecht, Utrecht, Netherlands

###### **Correspondence:** A. Vroegindeweij

**Introduction:** Since up to 60-76% of patients with (inactive) juvenile idiopathic arthritis (JIA) suffers from fatigue, leading to significant impairments including increased school absences and decreased physical functioning, an item to screen specifically for fatigue in JIA patients should be added to the Juvenile Arthritis Multidimensional Assesment Report (JAMAR).

**Objectives:** The first aim is to examine whether two items of the JAMAR “Evaluation of Quality of Life” (JQL) section are sufficiently able to detect fatigue, namely item 3 “difficulty carrying out activities that require a lot of energy such as running, playing football, dancing etc.” and item 9 “any difficulty concentrating or paying attention”. If not, the second aim is to discover which item of the Checklist Individual Strength-8 (CIS-8), a validated questionnaire for measuring fatigue, is most associated with the presence of fatigue in JIA patients. The most distinctive item will be recommended as addition to the JAMAR to assess whether further evaluation of fatigue would be required.

**Methods:** Data was derived from the JIA cohort and overlapping PROactive cohort at Wilhelmina Children’s Hospital in Utrecht, the Netherlands. 120 patients aged 8-18 filled out the CIS-8 and the JAMAR as part of routine clinical care. Patients were identified as fatigued if CIS-8 score ≥35 and severely fatigued if CIS-8 ≥40. ROC curves were used to determine discriminative values and cut-off points for both JQL items and each CIS-8 item. The aim was to identify an item that performed excellently for the entire cohort (AUC >.90). Spearman’s rank correlation was calculated to explore the possible correlation of items with disease activity reflected by the Active Joint Count (AJC). Stratification based on age and gender was performed. The CIS-8 was transformed from a 7-Likert scale to the 4-Likert scale of the JAMAR (never=0, sometimes=1, often=2, everyday=3).

**Results:** 40 patients were identified as fatigued (CIS ≥35) and 28 patients as severly fatigued (CIS ≥40). For the entire cohort, JQL item 3 (CIS ≥35; AUC .827, CIS ≥40; AUC .871) and JQL item 9 (CIS ≥35; AUC .792, CIS ≥40; AUC .824) could not detect (severe) fatigue excellently, whereas CIS-8 items "physically I feel exhausted" (CIS ≥35; AUC .939, CIS ≥40; AUC .953) and "I feel weak" (CIS ≥35; AUC .939, CIS ≥40; AUC .956) could. Ultimately, the most distinctive item was CIS-8 item "I feel weak", given that this item was least associated with disease activity. Conversion to 4-Likert scale did not alter sensitivity and specificity of the cut-off value (≥1; i.e. “sometimes”) (CIS ≥35; sensitivity 92.5% specificity 85.0%, CIS ≥40; sensitivity 100% specificity 77.2%). Similar results were found when stratifying based on age and gender (see Table 1).

**Conclusion:** The two JQL items could not detect fatigue excellently for the entire cohort, contrary to the CIS-8 items. CIS-8 item "I feel weak" would be most suitable to recommend as addition to the JAMAR to detect fatigue. Scores of ≥1 would indicate that the CIS-8 should be completed by JIA patients to assess to which extent fatigue is present.

**Disclosure of Interest**

None declared

**Table 1 (abstract P339).** Discriminative value of CIS-8 item "I feel weak" for (severe) fatigue after conversion to 4-Likert scale and stratification based on age and gender

CIS-8 item	AUC (95% CI)	P-value	Sensitivity (%)	Specificity (%)
*CIS-8 ≥35*				
Weak, aged 8-11	.947 (.847-1.000)	.042*	100	89.5
Weak, aged 12-18	.904 (.840-.969)	.000*	92.1	83.6
Weak, female	.886 (.831-.960)	.000*	91.9	79.2
Weak, male	.979 (.934-1.000)	.007*	100	93.7
*CIS-8 ≥40*				
Weak, aged 12-18	.922 (.872-.971)	.000*	100	76.1
Weak, female	.899 (.837-.962)	.000*	100	69.5
Weak, male	.977 (.920-1.000)	.025*	100	90.9

Note. *AUC* area under curve, *CI* confidence interval. *P < .05

### P340 Syndrome of noninflammatory musculoskeletal pain in paediatric rheumatology practice: how to control and limit panic

#### Y. Vyzhga

##### National Pirogov Memorial Medical University, Vinnytsya, Vinnitsya, Ukraine

**Introduction:** Presence or development of the pain syndrome, that standardly localized in extremities, muscles, near joint regions is very common cause of the patient’s admission to the general practitioners, orthopaedics, surgeons and rheumatologists as well. This pain usually presents spontaneous sudden onset and makes parents to find reason of its development and start treatment anyhow, even if its not needed with a situation above. The most common reason of such pain episodes is musculoskeletal syndrome and not an onset of the infectious disease or manifestation of the rheumatological process.

**Objectives:** The goal of the study is to analyse the most common reasons of patient’s admission to the paediatric rheumatologist with noninflammatory musculoskeletal pain syndrome in children.

**Methods:** To evaluate the meanings we analysed literature data, summarized experience of the outpatient consultation within passed 2 years of work in Vinnytsya regional children’s hospital.

**Results:** The most common reason of the doctors admission (up to 35%) is a development of the periodic arthralgias, “knocking” in the joints in patients with hypermobility syndrome. For the differential diagnose we used modified criteria of Carter and Wilkinson. In such patients very important to remember that cause of their pain at the periarticular tissues is increased stiffness of the ligaments. That’s why optimal treatment strategy is modification of physical activity with stretching training instead of nonevidential usage of NSAIDs. The next frequent cause of the arthralgias and stiffness in the knee joints (22 %) after physical activity that occurs with patella-femoral syndrome. Typical patient is a girl of 10 – 14 y.o. with complains on pain after physical activity, climbing stairs, prolonged sitting during classes. Additional investigations are sufficient for differential diagnose and final its confirmation, that allows to start rehabilitation in time and exclude unnecessary drugs prescription. Up to 15% of all cases are met in young sportsmen with iliotibial tract syndrome that manifests with pain in knees and calve muscles. As well 11% of sport active boys shows signs of Osgood-Schlatter that doesn’t require any special treatment, just lifestyle and sport activity managing. And the separate group of the conditions that require special attention is growth pain that causes majority of the stressful situation, associated with restless leg syndrome, that are very common (40 %) in Ukrainian paediatric population at the age 3 – 12 y.o. All the mentioned diagnoses can be ruled out after thorough differential diagnose and exclusion of other possible organic and structural causes. They may require short-term anti-inflammatory therapy, modification of the sport and rest activity.

**Conclusion:** Routine work of the paediatric rheumatologist in developing counties may include up to 30 – 35% of the patients that don’t require specific observance even though they present syndrome of musculoskeletal pain. Theoretical knowledge and practical skills allow provide correct differential diagnose and avoid polypharmacy prescription and quickly resolve situation with adequate physical and social adaptation.

**Disclosure of Interest**

None declared

### P341 Predictors of chronic pain in juvenile idiopathic arthritis: a systematic review

#### B. Zweers, M. Doeleman, J. Swart, S. de Roock

##### Immunology, Universitair Medical Center Utrecht, Utrecht, Netherlands

###### **Correspondence:** B. Zweers

**Introduction:** Despite the improved disease management of juvenile idiopathic arthritis (JIA), chronic pain is still common in pediatric patients with JIA. To date, it is not clear which patients are prone to develop chronic pain syndromes and which are not.

**Objectives:** To determine from recent literature which clinical factors are associated with the development of chronic pain in children with JIA.

**Methods:** A systematic review was conducted in accordance with the PRISMA guidelines for Scoping Reviews. PubMed and Embase were searched from inception to April 20th, 2020. Inclusion criteria were studies in children (≤18 years) with established JIA on clinical factors associated with the development of chronic pain. Pain had to be measured at multiple moments in time Studies focusing on psychological behavior and coping rather than on clinical factors were excluded. Primary outcomes were Visual analog scale for pain (VAS), Numerical Rating Scale pain (NRS) and pain trajectories described in terms of VAS or NRS.

**Results:** Search resulted in 292 references. After title & abstract screening, 49 articles were read in full-text and five studies were eventually included in the review. Baseline characteristics are presented in Table 1. Due to large variability in methodology and outcome measurements, meta-analyses of combined results were not valid. Therefore, a qualitative review was conducted, including results and individual statistical models. Three studies showed a positive relation with female gender and chronic pain: Shiff et al. comparing high and low pain trajectories: odds ratio (OR) 3.62 (95%CI 1.38-9.52), Taxter et al.: female gender adding 0.85 point to the 0-10 (NRS) of pain (95% CI 0.41-1.28) p<0.05 and Learoyd et al.: comparing high pain trajectory with low pain trajectory OR 2.92 (0.53-16.15) p=0.22. Regarding disease activity, active joint count (AJC) was the most used measurement. Two studies found a positive association between AJC and either chronic pain course OR 1.87, (95%CI, 1.32- 2.66)^3^ or pain at a single visit^1^.

Association of age at onset of JIA, disease duration, JIA subtype and treatment with chronic pain were non-significant or contradictory across included studies.

**Conclusion:** Despite diverse outcome measures and limited studies available, female patients with juvenile idiopathic arthritis seem to be at a higher risk for developing chronic pain than their male counterparts. Higher disease activity is likely to increase the risk of chronic pain for children with JIA. Thus, controlling disease activity appears the most important preventative measure regarding the development of chronic pain. No specific treatment was significantly associated with the development of chronic pain. We hypothesize that a treat-to-target approach would be well-suited in this regard. For age, age at onset, and disease duration, evidence remains inconclusive.

**Patient Consent Received**

No

**Disclosure of Interest**

None declared

**Table 1 (abstract P341).** Characteristics of included articles

Study	Design	Primairy outcome	Patients (n)	Female (%)	Disease duration at study enrollment, months means (SD)	JIA subtypes (%)
Malleson et al. 2004^1^	Cross-sectional	Prediction model, VAS pain	301	77	Median: 73,2	Poly:31 Oligo: 56 Systemic:13
Taxter et al. 2015^2^	Retrospective, 2 years	Patient reported outcomes amongst JIA subtypes, NRS pain	398	65	2,4 (NA)	Poly: 17 Oligo:37 Systemic: 8 Psoriatic: 7 ERA: 23 Undiff.: 8
Shiff et al. 2018^3^	Prospective, 5 years	Pain severity trajectories, VAS pain	1062	63.8	< 12 months	Poly: 24.5 Oligo: 40 Systemic: 6 Psoriatic: 5.7 ERA: 13.5
Arnstad et al. 2018^4^	Prospective, 8 years	Pain at onset related to longterm outcomes JIA, VAS pain.	243	70	At 8 year follow-up: median (IQR) 97(95-102)	Oligo: 49
Learoyd et al. 2019^5^	Retrospective, 1 year	Pain severity trajectories, VAS pain.	97	57	6.78(5.17)	Oligio: 6.2 Poly: 55.7 Systemic: 5.2 ERA: 33.0

### P342 Telemedicine appointments offer substantial benefits for families, but parents report they are not as good as in-person appointments and most want their child’s next appointment to be in-person

#### W. Costello^1,2^, S. Angevare^2,3,4^, R. Beesley^2,5^

##### ^1^Irish Children's Arthritis Network, Bansha, Ireland; ^2^European Network for Children with Arthritis, Geneva, Switzerland; ^3^KAISZ, Amsterdam, Netherlands; ^4^Autoinflammatory Alliance, San Francisco, United States; ^5^Juvenile Arthritis Research, Tonbridge, United Kingdom

###### **Correspondence:** R. Beesley

**Introduction:** During the COVID-19 (coronavirus) pandemic, some provision of healthcare has shifted to remote, technology-assisted appointments (telemedicine). Whilst parents/carers of children and young people (CYP) have reported benefits of telemedicine, some concerns remain.

**Objectives:** To understand the views of parents/carers about telemedicine, identifying the benefits and limitations of remote technology-assisted appointments.

**Methods:** An online survey was developed, translated into multiple languages and shared via social media and patient organisations, targeted at parents of CYP with rheumatic, autoimmune and autoinflammatory conditions. Fieldwork took place 2–22 April 2021. Consent was provided during enrolment.

**Results:** A total of 133 CYP were included (66% female, median age 12). The majority were from the UK, Ireland and Greece (53%, 26%, and 9% respectively).

Over half of respondents (53%) reported that it takes over an hour to travel to in-person appointments with their paediatric rheumatologist, 51% reported an appointment usually takes over three hours in total, and 45% take a full day out of school to attend. Parents reported taking time off work to attend appointments (41% take a full day, and 4% take two days off per appointment). Prior to COVID-19, 47% visited their paediatric rheumatologist four times per year. Overall, this represents a significant time burden for families.

Prior to COVID-19, 92% had never had a telemedicine appointment. Since March 2020, 71% had at least one telemedicine appointment (median 2, range 0-20).

Table 1 shows the scores (1 worst, 5 best) given by parents about their telemedicine experience. Overall, most aspects scored positively (p<.05). However, parents felt telemedicine was not as good as in-person appointments (mean 2.2, 95% CI 1.96-2.46).

56% of respondents reported telemedicine appointments had saved them time. 50% said it enabled them to have an appointment and 47% felt that it made the appointment safer. However, 76% felt that their consultant could not properly assess their child, 27% were concerned that the doctor could not identify changes in their child’s condition, 26% said it was hard to explain their child’s condition, and 27% of parents and 23% of CYP disliked telemedicine.

Overall 82% said they would prefer the next appointment to be in-person.

**Conclusion:** Whilst there are advantages to telemedicine, notably saving time and making appointments accessible, parents reported concerns about the ability to assess their child. There may be value in providing training to parents to allow for home-based assessments, particularly when the disease is stable. However, parents continue to report the value of in-person appointments.

**Disclosure of Interest**

None declared

**Table 1 (abstract P342).** Mean scores for a range of aspects of telemedicine (1-worst; 5-best). * Positive score (p<.05) ** Negative score (p<.05)

Aspect	Mean (95% CI)
Easy to schedule	3.50 (3.18, 3.82) *
On time	3.22 (2.89, 3.55)
Enough time with doctor	3.51 (3.19, 3.83) *
As good as in-person visit	2.21 (1.96, 2.46) **
Easier to see doctor	2.84 (2.55, 3.13)
Easy to sign-in	3.52 (3.22, 3.82) *
Quality of video	3.23 (2.93, 3.53)
Quality of sound	3.54 (3.26, 3.81) *
Able to speak freely	3.61 (3.34, 3.88) *
Able to understand doctor	3.61 (3.32, 3.90) *
Quality of care provided	3.43 (3.12, 3.73) *
Overall telemedicine experience	3.23 (2.91, 3.55)

## e-Poster viewing: Patient/parent organisation initiatives

### P343 Development of a standardised tool to assess abstracts submitted for international conferences

#### W. Costello^1,2^, S. Angevare^1,3,4^, R. Beesley^1,5^

##### ^1^European Network for Children with Arthritis, Geneva, Switzerland; ^2^Irish Children's Arthritis Network, Bansha, Ireland; ^3^Autoinflammatory Alliance, San Francisco, United States; ^4^KAISZ, Amsterdam, Netherlands; ^5^Juvenile Arthritis Research, Tonbridge, United Kingdom

###### **Correspondence:** R. Beesley

**Introduction:** Patient and public involvement (PPI) in paediatric rheumatology extends beyond clinical research and review of healthcare provision, and includes patient organisations working in partnership with clinicians and professional bodies to help ensure the needs of patients, parents and families are represented. The European Network for Children with Arthritis and Autoinflammatory Disease (ENCA) is a network of paediatric patient organisations across the continent of Europe and beyond, and since 2020 have been involved in reviewing abstracts for the joint PReS / ENCA annual congress.

**Objectives:** To develop a standardised tool to allow reviewers to consistently score abstracts submitted to international conferences.

**Methods:** Three members of the ENCA Board were invited to review abstracts ahead of the 2020 PReS Congress. Each reviewer received different abstracts, in line with standard PReS review practices. Abstracts were reviewed individually by reviewers using their experience, skills and expertise. During this process, each reviewer collated a list of questions and their own scoring system for each question. Once all reviews were complete, a single combined scoring system was developed, and checked against the processes followed by each individual. This scoring system was presented back to the PReS Scientific Committee for feedback and update.

**Results:** A single combined scoring system was developed, verified by the three reviewers and accepted by PReS as an example of best practice. The tool asks a total of 20 questions, with a maximum possible score of 20 points. Topics cover all parts of a standard abstract, and draw attention to scientific rigour, research methods, communication, impact on clinical care, validity of conclusions drawn, and involvement of patients and parents in the work.

**Conclusion:** The development of a standard tool has enabled reviewers to adopt a consistent approach to reviewing abstracts, giving a fair and balanced weight to a wide range of important considerations needed in the process. This tool can be used by patient organisations involved in abstract reviews as well as clinicians and researchers, and helps reduce potential bias or inconsistencies. It also helps new reviewers to become involved in the process as they have clear guidelines to follow.

**Disclosure of Interest**

None declared

### P344 Awareness cards for children and young people with arthritis help them explain their condition in an accessible way

#### R. Beesley

##### Juvenile Arthritis Research, Tonbridge, United Kingdom

**Introduction:** Children and young people (CYP) with juvenile idiopathic arthritis (JIA) face many burdens associated with their disease that their peers, teachers and communities often fail to understand. Sharing information about their condition and its impacts can be difficult, particularly in busy environments lacking privacy such as school classrooms and sports halls.

**Objectives:** To develop a resource to enable CYP to highlight they have JIA, allowing them to explain what that means for them in a simple way without the need for further explanation or details. The resource is designed to be small, accessible and transferable, allowing CYP to share with teachers and peers when necessary.

**Methods:** CYP with JIA asked for a resource to help them explain their condition, noting the variability of disease can appear paradoxical to others. A specific challenge for many CYP is that they can be active and appear well one day, but experience intense pain and loss of mobility the next. This can make explaining their condition in their own words more difficult.

A resource was developed with input from CYP with JIA, highlighting the key points about their condition. Printed out as business-card sized resources, these double-sided cards state ‘I have JIA’ on one side, with a summary of important information on the reverse. They are branded by an official JIA charity, giving a greater level of authority than a CYP can express on their own to teachers. The cards are available as printable resources from www.jarproject.org/teen, and are provided in support packs provided by Juvenile Arthritis Research (a UK charity) to CYP and their families.

**Results:** Prior to launch, information about the cards was shared on social media resulting in considerable interest from CYP with JIA and their families around the world. The printable version has been downloaded and shared widely, and the copies provided by the charity have been used across the UK. CYP and their families have reported that the cards have enabled them to tell others about their JIA, that the information has helped explain the impact and variability of their condition to teachers (particularly sports teachers to whom a CYP experiencing physical limitations needs to be able to share this information), that the cards are accessible and light-hearted whilst answering the key questions teachers and peers express, that they help outline why they need to attend medical appointments that may appear to be unrelated to their condition (such as ophthalmology assessments for uveitis), and that the use of the term ‘I have JIA’ reduces the stigma attached to the term ‘arthritis’.

**Conclusion:** CYP with JIA value the awareness cards which provide a new and simple way to share information about their condition, allowing them to explain it to others.

**Disclosure of Interest**

None declared

### P345 Experiences of virtual research involvement activities: from the perspectives of young people and researchers

#### L. E. Lunt^1,2^, J. Leslie^3^, S. Gnanenthiran^3^, M. Purvis^3^, T. Kierkegaard Holt^3^, J. E. McDonagh^1,2^, on behalf of Your Rheum, a national advisory group of the Barbara Ansell National network for Adolescent rheumatology

##### ^1^Centre for Epidemiology Versus Arthritis, Centre for Musculoskeletal Research, Manchester Academic Health Science Centre, University of Manchester; ^2^NIHR Manchester Biomedical Research Centre, Manchester University NHS Foundation Trust; ^3^Your Rheum, a national advisory group of the Barbara Ansell National network for Adolescent rheumatology, Manchester, United Kingdom

###### **Correspondence:** L. E. Lunt

**Introduction:** Your Rheum is a UK young person’s research advisory group, for those aged 11-24 years and diagnosed with a rheumatic condition. Prior to the COVID-19 pandemic, Your Rheum mostly engaged with its members and researchers at face-to-face meetings twice a year, offering online activities to potentially enable broader involvement.

**Objectives:** To explore the advantages and disadvantages of conducting research involvement activities virtually, from the perspectives of both young people and researchers.

**Methods:** Online surveys were only sent to Your Rheum members and researchers, who have engaged with the Group virtually over the past 12 months. 8/16 of these young people responded; 1 male, 7 female; age range 16-22 years and 3/6 researchers representing different research projects.

**Results:** Many young people commented on the convenience of meetings taking place virtually, allowing more young people to be involved regardless of location. Some highlighted that face-to-face meetings were often too far to travel to and therefore required a significant commitment to attend. For example as one young person highlighted in regards to virtual meetings, “no travel is required so it is much less of a whole-day commitment, meaning it is much easier and more likely that I will be able to attend.” Similarly, researchers who responded also stated the convenient aspect to conducting virtual activities. Additionally, for some young people, speaking online is easier and more comfortable than face-to-face communication; with some expressing, it is not such an intimidating environment and there is less pressure to contribute to discussions. Using interactive tools on virtual platforms, such as breakout rooms, is another positive aspect of online meetings. One researcher noted a positive feature was the ability to switch cameras off, helping younger or quieter members to participate. However, a number of young people felt the opposite was true and have found virtual meetings difficult to contribute to, “it is more stressful … as everyone is looking at you and no one else is speaking. The format of having meetings online also makes the whole event feel much more formal … which means that it can be quite intimidating to speak.” Difficulty building virtual relationships and connections was a significant disadvantage. This was highlighted by over half of respondents, who reported that it is easier to get to know others in person as you have the opportunity to deviate from the topic being discussed, allowing for natural conversations to occur. As one young person poignantly noted, online meetings seem to be “lacking true human connection”. From a researcher perspective, the subtle insights gained from face-to-face interactions were missing.

**Conclusion:** This study has highlighted strengths of conducting research involvement activities virtually. For example, logistics and convenience, and for some young people, the development of personal skills such as speaking to others and contributing to group discussions. However, the formal and rigid nature of virtual meetings makes interacting and connecting with other young people difficult. Moving forward, a blended approach to Your Rheum activities is proposed ie the inclusion of frequent virtual meetings, as well as regular bi-annual face-to-face meetings (COVID-19 restrictions permitting). However, when planning virtual meetings, consideration to social interactions and opportunities to get to know one another remains important for young people.

**Disclosure of Interest**

L. Lunt: None declared, J. Leslie: None declared, S. Gnanenthiran: None declared, M. Purvis: None declared, T. Kierkegaard Holt: None declared, J. McDonagh Consultant for: Consultancy fees from Pfizer (2018) and CSL-Behring (2021)

### P346 What can families and researchers learn from each other about glucocorticoid medication? – Lessons from a virtual patient public involvement event

#### S. Singhal^1,2^, E. M. D. Smith^1,2^, L. Roper^2,3^, C. E. Pain ^1,2^

##### ^1^Institute of Life Course and Medical Sciences, University of Liverpool; ^2^Department of Paediatric Rheumatology, Alder Hey Children’s NHS Foundation Trust, Liverpool, UK; ^3^Institute of Population Health, University of Liverpool, Liverpool, United Kingdom

###### **Correspondence:** S. Singhal

**Introduction:** Patients and public involvement (PPI) events allow families to use their own experience to contribute meaningfully to health research, as well as learn more about research processes and findings. Glucocorticoid medications, or steroids, and their side effects remain a central part of the patient experience across a range of paediatric rheumatic diseases. The research team hosted a virtual PPI event around the use of glucocorticoid medication in children and young people (CYP).

**Objectives:** The aims of the virtual event were to share experiences of glucocorticoid treatment, provide education about the research team’s work, and to identify future key CYP/parents that would continue to be involved in a smaller that PPI group to co-develop glucocorticoid associated clinical studies.

**Methods:** The event was advertised to CYP with experience of glucocorticoid medication use and their parents through clinicians (n=4), charities (n=9) and patient groups (n=2), including their associated social media channels. The event was held virtually on Zoom due to Covid-19 restrictions. During the session, the research team shared findings from four on-going studies involving glucocorticoid medications. Interactive polls and structured discussion were used to help participants share their experiences of steroids. Pre- and post-attendance online questionnaires were used to collect quantitative and qualitative feedback about the event.

**Results:** 11 families with a range of conditions necessitating glucocorticoid medication (JIA, Behcet’s, Lupus, Nephrotic Syndrome, Allergic Bronchopulmonary Aspergillosis) participated from across the UK. The ages of CYP ranged from 2-26. Participants had used glucocorticoid medication through a variety of administration routes (oral, intravenous, intra-articular, topical, inhaled). The duration of use varied from <1 year to >10 years.

Online pre-attendance and post-attendance questionnaires showed an improvement in mean self-reported confidence [1 = not at all confident, 5 = very confident] in the following: what steroid medications are (pre = 3.9, post = 4.8), steroid side effects (pre = 3.8, post = 4.6), patient-reported outcome measures (pre = 2.0, post = 4.5), available research on steroids (pre = 2.2, post = 3.5). Most participants (86%) reported that they were likely or very likely to attend future PPI events.

Diversity was seen in the experiences and views of CYP in relation to glucocorticoid medication. Many reported several benefits including rapid improvement in their disease, reduced pain and improved mobility. However, there were a greater number of negative effects reported, impacting upon health-related quality of life, including effects on body image, school, mood and relationships with others. A number of participants reported the most enjoyable part of the event was hearing other participants’ experiences. This suggests that PPI events offer avenues for insight and therapeutic benefit through sharing common experiences.

**Conclusion:** PPI initiatives provide a valuable forum for both researchers and families to share their perspectives. They can be a useful educational tool to disseminate research findings that are relevant to patients and improve health research knowledge. Families found the experience to be beneficial and enjoyed the opportunity to share experiences on the effect of glucocorticoid medication on health-related quality of life. Five families opted into further PPI group work to engage in the co-development of future glucocorticoid studies.

**Disclosure of Interest**

None declared

### P347 Work experience of a regional patient organization, parents of children with juvenile arthritis

#### Y. M. Spivakovskiy^1^, A. Y. Spivakovskaya^2^, I. A. Eliseeva^3^

##### ^1^Depertment of Faculty Pediatrics; ^2^Depertment of Hospitality Pediatrics, Saratov State Medical University; ^3^Patient Organization "Life without pain", Saratov, Russian Federation

###### **Correspondence:** A. Y. Spivakovskaya

**Introduction:** The issues of medical supervision of children with rheumatological diseases are key and priority, but not the only ones when observing patients of this group in real clinical practice. A serious problem in the solution of which the participation of medical personnel is also necessary is the problem of social adaptation of young patients with such a serious pathology. Social adaptation issues include psychological, moral, ethical, deontological and other problems that may arise both in organized groups and in the families of these children.

**Objectives:** In the Saratov region of the Russian Federation, a regional patient organization has been functioning for almost 10 years, uniting the parents and children of JIA patients themselves.

**Methods:** The public association of parents whose children are sick JIA “Life Without Pain” was created in August 2011 and when it was created, it aimed to provide maximum assistance in the implementation of the treatment process to achieve the best result.

**Results:** During its work, the methods and operational goals of the association have undergone certain changes. Taking into account the successfully functioning all-Russian patient organizations capable of having a significant impact on the organization of care for children at the federal level, a natural revision of the goals of the regional patient structure took place. So, as the most relevant were preserved - the possibilities of operational interaction with regional health authorities (regional ministry), operational monitoring of drug supply, as well as educational programs and socio-cultural programs for sick children and their parents. One of the areas of socio-cultural activities of the regional patient association has become the organization of traditional regular events, most often timed to coincide with significant dates in the medical calendar - Word Arthritis Day, World Young Rheumatic Diseases Day and Children's Day. The material component of these events is based on the principles of fundraising, as well as with the sponsorship of the Russian Children's Fund. These events are very popular among children with JIA, especially taking into account the presented possibility of independent performances in various genres (painting, diclomation, choreography, original genre and others). During the COVID-19 pandemic, taking into account the needs of children, all these activities were saved and transferred to an on-line format. A separate aspect of the relevance of these events was the involvement of volunteers from among the students of the medical university.

**Conclusion:** The feasibility of maintaining and functioning of small regional patient organizations is determined by the allocation of a rational segment of their activities in which the efficiency of their work will be maximum. In our case, such a segment is social and cultural events, educational and training activities and interaction with local health authorities.

**Patient Consent Received**

Yes

**Disclosure of Interest**

None declared

## e-Poster viewing: COVID-19 (Coronavirus)

### P348 Multisystem inflammatory syndrome in children associated with SARS-COV-2 and Kawasaki disease: experience of pediatric clinics in Russia

#### I. Avrusin^1^, L. Bregel^2,3^, K. Belozerov^1^, A. Kupreeva^1^, A. Tantasheva^1^, D. Malekov^1^, T. Kornishina^1^, V. Masalova^1^, O. Kalashnikova^1^, V. Chasnyk^1^, M. Kostik^1^

##### ^1^Saint Petersburg State Pediatric Medical University, Saint Petersburg; ^2^Irkutsk State Medical Academy of Postgraduate Education, Branch of Russian Medical Academy of Continuous Professional Education; ^3^Irkutsk Regional Children’s Clinical Hospital, Irkutsk, Russian Federation

###### **Correspondence:** I. Avrusin

**Introduction:** COVID-19 in children is often asymptomatic or with only mild symptoms. However, since April 2020 there are many reports that the new coronavirus infection might be associated with pediatric hyperinflammatory condition, that fully or partially meets the criteria for Kawasaki disease (KD). This phenomenon was later called multisystem inflammatory syndrome in children (MIS-C) or pediatric inflammatory multisystem syndrome temporarily associated with SARS-CoV-2 (PIMS-TS).

**Objectives:** Our study aimed to evaluate main clinical and laboratorial features and course of MIS-C and compare it with Kawasaki disease in children.

**Methods:** The retrospective study included 50 children (34 male, 16 female), aged from 7 months to 16 years 9 months (median 8.8 years), who met the WHO criteria for MIS-C and 60 patients (34 male, 26 female, aged from 3 months to 6 years (median 2 years) with Kawasaki disease.

**Results:** Prior COVID-19 infection in MIS-C group was confirmed by positive SARS-CoV2 test using RT-PCR (n=11) or IgM (n=21), IgG (n=38) and/or close contact with a person with confirmed COVID-19 (n=21) clinical features of previous COVID-19 infection were noted in 22 patients.

Clinical sings of MIS-C included fever (100%), gastrointestinal disorders (81.6%), rash (90%), conjunctivitis (93.6%), sore throat (68.1%) cheilitis (54.6%), cervical lymphadenopathy (68.2%), hands and feet erythema/oedema (69.8%), hepathomegaly (64.6%). In the majority of patients elevated levels of inflammatory biomarkers, D-dimer, troponin, ferritin were found. Most of patients had a tendency to anemia (median hemoglobin 105 g/l). Platelet levels varied greatly (8-919*10^9^/l), 37.5% of patients had thrombocytopenia. Carditis and coronary artery dilatation were found in 48.9% and 22.7%, respectively. Arterial hypotension/shock was in 52.5%. Heart MRI showed signs of myocarditis (n=5): Т1 prolongation (n=2); signs of myocardial edema, pericarditis, severe arrhythmia, and a tendency to diastolic overload (n=1), but no signs of ischemic or non-ischemic myocardial damage, and the global systolic function stayed normal. Patients were treated with high-dose glucocorticoids (93.6%), low-weighted heparin (100%), low dose of aspirin (64.4%), intravenous immunoglobulin (37.8%); Tocilizumab was used in three patients (6%). The median duration of hospitalization was 22 days, and 65.9% of patients required an ICU admission. Some of the most informative indicators for the differential diagnosis of MIS-C and KD are shown in the table.

**Table:** 

**Parameter**	**MIS-C**	**KD**	**p**	**Parameter**	**MIS-C**	**KD**	**p**
Gastrointestinal disorders, %	81.6	53.3	0.0019	Thrombocytosis, %	39.6	82.8	0.000004
Neurological symptoms, %	46.8	6.7	0.000002	Thrombocytopenia, %	37.5	3.5	0.00001
Sore throat, %	68.1	18.3	0.000000	Increased ferritin, %	87.5	61.9	0.029
Face swelling, %	47.7	20.3	0.003	Myocarditis, %	48.9	6.7	0.000001
**Parameter**	**MIS-C**	**KD**	**Sensitivity**	**Specificity**	**AUC**	**OR**	**p**
Age > 50 months, %	97.7	7.6	87.2	42.5	0.919	526.8	0.000001
CRP > 15 8 IU/l, %	64.4	7.7	64.4	92.3	0.786	21.8	0.0000001
Ferritin > 260 ng/ml, %	78.1	14.3	78.1	85.7	0.821	21.4	0.000005
Serum Protein ≤ 63 g/l, %	81.3	42.2	81.3	57.8	0.744	5.8	0.0001

**Conclusion:** MIS-C is severe life-threatening condition in children, which pathogenesis and relation to COVID-19 requires further research. There are differences in the frequency of some signs that possibly can be used as a basis for differential diagnosis of the studied conditions.

This work supported by the Russian Foundation for Basic Research (grant № 18-515-57001)

**Patient Consent Received**

No

**Disclosure of Interest**

None declared

### P349 Outcomes of IV Methylprednisolone monotherapy in MIS-C: is IVIG always necessary?

#### F. Licciardi^1^, L. Baldini^1^, M. Dellepiane^1^, C. Covizzi^1^, R. Mogni^1^, G. Pruccoli^1^, C. Orsi^2^, E. Parodi^1^, F. Mignone^1^, I. Rabbone^2^, D. Montin^1^

##### ^1^Public Health and Pediatrics, Università Degli Studi di Torino, Torino; ^2^Public Health and Pediatrics, Università del Piemonte Orientale, Novara, Italy

###### **Correspondence:** L. Baldini

**Introduction:** Multisystem inflammatory syndrome in children (MIS-C), or paediatric inflammatory multisystem syndrome temporally associated with SARS-CoV-2 (PIMS-TS), is a newly described pediatric syndrome, partially overlapping with Kawasaki Disease (KD) and Macrophage Activation Syndrome. According to the literature, MIS-C requires ICU admission in 73,3% of cases, with 1.9% overall mortality. Patients may develop coronary artery anomalies (CAA), either dilatations (11,6%) or aneurysms (10,3%). Most physicians have been treating MIS-C like KD, so far. Conversely, based on our experience, since April 2020 we have been treating MIS-C patients with IV methylprednisolone (MP) as a first-tier monotherapy: herein, we present the outcome of the first 23 consecutive patients treated according to our treatment protocol.

**Objectives:** To evaluate the outcome (ICU admission, inotropic support, coronary abnormalities) of a cohort of consecutive MIS-C patients treated with MP as first-line monotherapy.

**Methods:** Patients satisfying the WHO preliminary case definition of MIS-C, with no need of inotropic support at admission, have been treated with fluid restriction and MP monotherapy, the dose depending on the presence/absence of myocardial involvement: if hypotension according to age, gender, and height adjusted chart, or Ejection Fraction (EF) <50%, or NT-proBNP ≥1500 pg/ml are present, the patient receives high-dose pulse IV MP 10 mg/Kg/day for 3-5 days, otherwise low dose IV MP 2 mg/Kg/day is administered. After 48 hours, if CRP increases and/or fever persists, the treatment is intensified either with a MP dose increase or with subcutaneous Anakinra 5 mg/Kg/day. IVIG is reserved for patients with suspected CAA at any ultrasound evaluation (defined according to American Heart Association 2017 Guidelines for KD), or presenting persistent symptoms despite defervescence and CRP reduction. We retrospectively collected and analyzed clinical data of a cohort of consecutive MIS-C patients treated with MP mono-therapy between the 1st of April and the 31st of January 2021, at Regina Margherita Children Hospital (Turin, Italy). Clinical data were retrospectively collected; as primary outcomes we considered: rate of ICU admission, rate of inotropic support need, and incidence of CAA. As secondary outcomes we evaluated: CRP halving time, MP and NT-proBNP halving time, and days between first pathological echocardiogram and EF normalization.

**Results:** Twenty-three MIS-C patients were included. 18 patients (78,3%) showed myocardial involvement and were treated with high-dose pulse MP (Group A), 4 needed anakinra due to persistent fever. 5 patients with no cardiac damage (21,7%) were treated with low dose MP (Group B), in 2 of these (40,0%) MP dose was intensified due to persistent fever. All of the patients recovered; 1 (4.3%) needed ICU admission with inotropic support, 1 developed a CAA six days after MP start. Median CRP halving time was 2 days (2 days in Group A and 5 days in Group B), NT-pro-BNP halved in 3 days in Group A, while EF normalized in 4.5 days.

One patient needed ICU admission and inotropic support (4.3%), 1 patient of group A developed a small coronary aneurysm (5 mm, z score 4).

**Conclusion:** Despite some limitations, including the sample size and the absence of a control group treated with IVIG, our data suggest that early administration of MP together with fluid restriction can rapidly decrease the inflammation and restore myocardial contractility in MIS-C, considerably reducing the need of ICU admission and/or inotropic support. Encouragingly enough, the incidence of CAA in our cohort is low compared to published cohorts (4.3% vs 20%). Further studies in bigger cohorts are needed to confirm our findings.

**Patient Consent Received**

No

**Disclosure of Interest**

None declared

### P350 Chilblain-like acral lesions due to C. pneumonia infection during pandemic period

#### A. M. Barisiene^1^, A. Snipaitiene^1,2^

##### ^1^Academy of Medicine, Lithuanian University of Health Sciences; ^2^Pediatric, Hospital of Lithuanian University of Health Sciences Kauno Klinikos, Kaunas, Lithuania

###### **Correspondence:** A. M. Barisiene

**Introduction:** Increase in cases with chilblain-like acral lesions has been observed during the pandemic period. Epidemiological data suggest that children have different immunological responses to SARS-CoV-2 virus, which can cause mild respiratory illness but more frequently involve other organ systems. Co-infection of SARS-CoV-2 with Chlamydia or Mycoplasma pneumoniae has been described both in adults and pediatrics (1).

**Objectives:** To analyze clinical features, laboratory and instrumental findings of a group of patients with chilblain-like lesions during SARS-CoV-2 pandemic period.

**Methods:** Retrospective analysis of 4 patients with chilblain-like lesions.

**Results:** We present 4 patients with chilblain-like acral lesions (2 female and 2 male). The median age was 15.5 years (13 to 16 years old). Two patients had lesions only in toes whilst others had both hand and feet perniosis.

All patients had no laboratorical signs of inflammation (CRP, ESR, WBC, neutrophil, and lymphocyte count were in normal range) and markers for rheumatic and connective tissue diseases (cryoglobulins, antinuclear antibodies, anti-double-stranded DNA antibodies, antineutrophil cytoplasmic antibodies, rheumatoid factor) were negative.

Levels of serum complement C3 (mean value 0.775 g/l) and complement C4 (mean value 0.1425 g/l) was reduced in all cases.

Two patients had positive Chlamydia pneumonia antibodies (IgM titers of >12 were defined as positive), others were not tested. The first patient had lesions in toes and for the second patient, both hands and feet were damaged.

All patients underwent ultrasound of affected sites. One of 4 patients was diagnosed with reactive arthritis in metatarsophalangeal joints in ultrasound and MRI.

There was no epidemiological anamnesis or clinical manifestation or any evidence of SARS-CoV-2 infection (SARS-CoV-2 antibodies were negative) for all patients. Also, there was no anamnesis of any respiratory tract symptoms or diagnosed infection in the last 4 months in all patients.

Patients with C. pneumoniae IgM antibodies positive were given a course of clarithromycin. Despite the prescribed course of antibiotics, the skin lesions remained. One patient with arthritis was treated with NSAIDs. Others resolved by themselves without requiring any treatment.

**Conclusion:** Chilblain-like lesions in the skin can be one of the signs of previous SARS-CoV-2 infection despite negative virus antibodies (2). Possible hypothesis is a high production of type I interferon (IFN) associates with early viral control and suppression of antibody production. A strong IFN response can cause skin manifestations (3). Co-infection with other atypical bacteria can intensify immune complexes formation and skin injury (4). This phenomenon can be observed with low levels of C3 and C4 in blood samples.

References

1. Oliva A, Siccardi G, Migliarini A, Cancelli F, Carnevalini M, D’Andria M, Attilia I, Danese VC, Cecchetti V, Romiti R, Ceccarelli G, Mastroianni CM, Palange P, Venditti M. Co-infection of SARS-CoV-2 with Chlamydia or Mycoplasma pneumoniae: a case series and review of the literature. Infection. 2020;28:1–7. doi: 10.1007/s15010-020-01483-8

2. Kashetsky N, Mukovozov IM, Bergman J. Chilblain-Like Lesions (CLL) Associated With COVID-19 (“COVID Toes”): A Systematic Review. Journal of Cutaneous Medicine and Surgery. April 2021. doi:10.1177/12034754211004575

3. Baeck M, Herman A. COVID toes: where do we stand with the current evidence?. Int J Infect Dis. 2021;102:53-55. doi:10.1016/j.ijid.2020.10.021

4. De Luigi G, Zgraggen L, Kottanattu L, Simonetti G, D, Terraneo L, Vanoni F, Terrani I, Bianchetti M, G, Lava S, A, G, Milani G, P: Skin and Mucous Membrane Eruptions Associated with Chlamydophila Pneumoniae Respiratory Infections: Literature Review. Dermatology 2021;237:230-235. doi: 10.1159/000506460

**Disclosure of Interest**

None declared

### P351 Lithuanian tertiary pediatric centre experience of multi-system inflammatory syndrome in children (MIS-C): clinical cases study

#### A. M. Barisiene^1^, M. V. Petrucionyte^1^, R. Sileikiene^1,2^

##### ^1^Academy of Medicine, Lithuanian University of Health Sciences; ^2^Pediatric, Hospital of Lithuanian University of Health Sciences Kauno Klinikos, Kaunas, Lithuania

###### **Correspondence:** A. M. Barisiene

**Introduction:** Multisystem inflammatory syndrome in children (MIS-C) associated with COVID-19 presents with fever, severe illness, laboratory signs of inflammation, two or more organ systems impairment, and evidence of SARS-CoV-2 infection. Some symptoms of MIS-C mimic Kawasaki disease, toxic shock syndrome, or secondary hemophagocytic lymphohistiocytosis (HLH) (1).

**Objectives:** To present MIS-C clinical and laboratorical features according to the type and follow-up six weeks later after hospital discharge.

**Methods:** A monocentric retrospective study was done, including hospitalized children who met the criteria for the MIS-C. We analyzed clinical features, laboratory and instrumental examinations, treatment, and duration of hospitalization in the intensive care unit (ICU). Data were analysed using SPSS 20. P value <0.05 was considered significant.

**Results:** 15 patients were included in the study, 73.3 % of whom were male. The mean age was 6.8 ± 4.22 years (1 to 17 years old). Five patients of 15 were identified as Kawasaki-like disease, 3 as toxic shock syndrome, and 1 as secondary HLH. The longest duration of fever (6.33 ± 3.21 days) and hospitalization in ICU (5.67 ± 3.21 days) was in cases with toxic shock syndrome, shortest – MIS-C patients (3.16 ± 1.94 and 0.33 ± 0.51 days) (p=0.514, p=0.726, respectively). The following organ systems were damaged: hematologic (n=15, 100 %), coagulation (n=15, 100 %), gastrointestinal (n=14, 93.3 %), lymphatic (n=10, 66.67 %), mucocutaneus (n=8, 53.3 %), cardiac (n=7, 46.67 %), respiratory (n=2, 13.3 %), renal (n=1, 6.6 %). Inflammatory laboratory biomarkers (high CRP, WBC, Neu, ESR) were presented in all MIS-C types except HLH where only ESR was elevated (p=0.062). Highest (CRP, WBC, Neu) mean values were in the toxic shock syndrome (CRP 212 ± 92.9 mg/l, WBC 30.4 ± 22.4 x 10^9^/l, Neu 25.7± 31.8 x 10^9^/l) (p=0.271, p=0.930, p=0.863, respectively). The mean value of PLT was low in HLH (67 x 10^9^/l ) and toxic shock syndrome (88.6 ± 12.6 x 10^9^/l) (p=0.260). Low Hgb was presented in all types except typical MISC (p=0.260). All patients had normal LDH.

The highest mean levels of troponin I and D-dimer were found in the toxic shock syndrome (1.36 ± 1.19 mcg/l and 5.5 ± 1.57 mg/l) and Kawasaki-like disease (0.2 ± 0.39 mcg/l and 3.56 ± 1.32 mg/l) (p=0.801, p=0.125, respectively). All the types were treated with antibiotics (till negative bacteria culture tests), glucocorticoids, and anticoagulants. Treatment with intravenous immunoglobulin was administered with Kawasaki-like disease (80 %) and toxic shock syndrome (33.33 %). Vasoactive support was received by 2 from 3 patients with toxic shock syndrome. 13 patients of 15 came to follow-up after 6 weeks, no residual symptoms were found. All the patients, who had any cardiac system involvement, had no changes in echocardiography and cardiac markers during the follow-up.

**Conclusion:** Manifestation of MIS-C is nonspecific and there is a wide variety of symptoms, but we observed that all of the patients had a fever, laboratory signs of inflammation, coagulation disorders, almost all of them had gastrointestinal symptoms, moreover mucocutaneous system and lymphadenopathy involvement also was a common sign. All patients, that came to follow-up, had no lasting effects of MIS-C.

**References**

1. Nakra NA, Blumberg DA, Herrera-Guerra A, Lakshminrusimha S. Multi-System Inflammatory Syndrome in Children (MIS-C) Following SARS-CoV-2 Infection: Review of Clinical Presentation, Hypothetical Pathogenesis, and Proposed Management. Children (Basel). 2020 Jul 1;7(7):69. doi: 10.3390/children7070069. PMID: 32630212; PMCID: PMC7401880.

**Disclosure of Interest**

None declared

### P352 Pediatric inflammatory multisystemic syndrome temporally associated with COVID 19 (PIMS-TS): characteristics of the Sainte Justine Hospital cohort

#### C. Beaufils^1^, E. Haddad^1^, F. Touzot^1^, H. Decaluwe^1^, J. J. De Bruycker^1^, K. Samaan^1^, N. Dahdah^1^, A. Finzi^2^, C. Renaud^1^, M.-P. Morin^1^

##### ^1^Chu Sainte Justine; ^2^CHUM, Montréal, Canada

###### **Correspondence:** C. Beaufils

**Introduction:** Since the beginning of SARS-Cov-2 pandemic, children represent a small proportion of patients with an acute disease and present with mild symptoms or are either asymptomatic. However pediatric patients might present with late, post infectious, manifestations of COVID 19, namely Pediatric Inflammatory Multisystemic Syndrome temporally associated with COVID 19 (PIMS-TS).

**Objectives:** We aim to describe the characteristics of pediatric patients with PIMS-TS hospitalised in Sainte Justine Hospital and report their evolution according to their treatment and care.

**Methods:** Patients were recruited prospectively since April 2020. Patient were included if they were less than 18 years old, had at least 2 days of fever, had multisystemic involvement (at least 2 systems) and had a temporal association with COVID 19. This temporal association was defined as: positive reverse transcription (RT)-PCR or antibodies to SARS-Cov2, history of contact with a confirmed or suspected SARS-Cov 2 infected individual or symptom appearance during the pandemic. Patient were excluded if they had an alternative diagnosis that explained the symptoms. Patient were sub categorized into two categories depending on their clinical presentation: 1- Complete or incomplete Kawasaki disease (KD), following the American Heart Association Criteria; or 2- Toxic shock syndrome (TSS). Data were collected during the hospitalization and the follow up.

**Results:** Between April 30^th^, 2020 and April 30^th^, 2021, 72 patients were included, 13 (18.1%) with complete KD, 40 (55.6%) with incomplete KD and 19 (26.4%) with TSS. There was more male in the complete KD group (69.2%) and more female in the TSS group (52.6%) but there was no significative difference between the two groups (p = 0.35). Patient with TSS presentation were significatively older than those with KD presentation (10.3 years vs 4.9 years, p < 0.01). Caucasian patients are the most represented ethnic group in the cohort (26.4 %) but Afro-American patient are over-represented in the TSS group (6/19 patients, 31.6%; p= 0.2). Gastro-intestinal symptoms were seen in 50.9% (27/53) and 94.7% (18/19) patients with KD and TSS respectively (p < 0.001). Patients in the TSS group had higher mean value of PCR (239.1 mg/L vs 143.9 mg/L; p< 0.001) and more frequent lymphopenia (89.5% vs 34%; p< 0.001) than those in KD group. Only 40/72 (55.6%) patients in the cohort had either a positive RT-PCR and/or positive antibodies to SARS-Cov-2 and/or a contact with confirmed or suspected infected individual. These proportion increase to 18/19 (94.7%) in the TSS group (p < 0.001). Twenty-three patients (31.9%) required ICU hospitalization including 17 on 19 patients in the TSS group (89.5%; p < 0.001). Cardiac involvement was the most frequent complication either as coronary aneurysm, (15/72 – 20.8%) mainly in patient with KD presentation or as cardiac dysfunction (24/72 – 33.3%), mainly in patient with TSS presentation. Patient received intravenous immunoglobulins (69/72 – 95.8%), steroids (49/72 – 68.1%), sometimes both (48/72 – 66.7%). Five patients (6.9%) required biotherapy: 1 with Enbrel and 4 with Anakinra. Patient received a treatment 6.6 days after the beginning of the symptoms. After 3 months, 4 patients (0.5%) had persistent coronary dilatation and 2 (0.3%) had mitral insufficiency.

**Conclusion:** This cohort study enables a better description of PIMS clinical and biological presentation, which can sometimes be confusing. It also highlights the importance of fast and adequate diagnosis and treatment to avoid the risk of acute and chronic complications, especially cardiac complications.

**Patient Consent Received**

Yes

**Disclosure of Interest**

None declared

### P353 Healthcare professionals play an influential role supporting informed parental choice when considering vaccinating against COVID-19: results of a survey of parents/carers of children and young people with rheumatic and autoinflammatory diseases

#### W. Costello^1,2^, S. Angevare^2,3,4^, R. Beesley^2,5^

##### ^1^Irish Children's Arthritis Network, Bansha, Ireland; ^2^European Network for Children with Arthritis, Geneva, Switzerland; ^3^KAISZ, Amsterdam, Netherlands; ^4^Autoinflammatory Alliance, San Francisco, United States; ^5^Juvenile Arthritis Research, Tonbridge, United Kingdom

###### **Correspondence:** R. Beesley

**Introduction:** Vaccination of children and young people (CYP) with rheumatic and auto-inflammatory diseases is reported to be lower than amongst healthy peers. Whilst vaccination to confer protection against COVID-19 is now underway amongst adults, vaccination of children under 16 years is not yet available.

**Objectives:** To understand the views of parents/carers regarding vaccination against COVID-19 for CYP with rheumatic and autoinflammatory diseases.

**Methods:** An online survey was developed, translated into multiple languages and shared via social media and patient organisations, targeted at parents of children and young people (CYP) with rheumatic, autoimmune and autoinflammatory conditions. Fieldwork took place between 2 and 22 April 2021. Consent was provided during enrolment.

**Results:** A total of 133 CYP were included (66% female, median age 12). The majority were from the UK, Ireland and Greece (53%, 26%, and 9% respectively). The primary diagnosis for the majority was Juvenile Idiopathic Arthritis (JIA; 46% polyarticular, 16% oligoarticular, 13% enthesitis-related JIA, 7% psoriatic, and 7% systemic), although no significant differences in outcomes between any diagnoses were identified.

At the time of completing the survey, the majority of CYP had received no vaccination against COVID-19 (93%), although 5% had received one dose and 2% had received both doses; those receiving any vaccination were all aged 16 and over. The majority of parents/carers (65%) would agree for their child to be vaccinated to prevent COVID-19 if the vaccine was approved and available at no cost, with only 8% saying they would not agree, and 21% unsure. Overall, 45% would allow their child to have the vaccine as soon as it was available, with a further 20% who would prefer to wait, and 11% who will allow their child to have the vaccine only when required to.

Reasons given by parents choosing not to vaccinate their child against COVID-19 focussed on perceived safety, apparent lack of testing, and alleged potential damage caused by vaccines.

When asked about the sources of information most likely to influence their decision about vaccination against COVID-19 for their child, the majority (90%) cited their doctor or health professionals; however this varied significantly (p=.015, chi-square) based on whether they currently would agree to have their child vaccinated, with only 64% of parents who would not vaccinate their child saying their healthcare professional would influence their decision. Around one-third of parents would be influenced by information from their patient organisation.

Unsurprisingly, parents who indicated they would not be vaccinated themselves were less likely to agree for their child to be vaccinated (p<.001, chi-square).

**Conclusion:** Healthcare professionals and patient organisations play a key role in supporting, advising and influencing parental decision-making with regards to COVID-19 vaccination amongst CYP with rheumatic and autoinflammatory conditions, particularly amongst parents/carers who are currently undecided whether to vaccinate or not. As vaccines become available for CYP there is a need for accurate, reliable and clear information for parents and carers to make informed decisions.

**Disclosure of Interest**

None declared

### P354 MIS-C at two tertiary hospitals in Cape Town, South Africa: clinical phenotype and distinguishing features from similar acute inflammatory conditions

#### C. Butters^1^, D. R. Abraham^2^, H. Facey-Thomas^1^, D. Abrahams^1^, A. Faleye^1^, H. Rabie^2^, C. Scott^1^, L. Zühlke^1^, K. Webb^1^

##### ^1^Division of Paediatric Rheumatology, School of Child and Adolescent Health, University of Cape Town; ^2^Tygerberg Hospital, Stellenbosch University, Cape Town, South Africa

###### **Correspondence:** C. Butters

**Introduction:** Distinguishing Multisystem Inflammatory Syndrome in Children (MIS-C) associated with Severe Acute Respiratory Syndrome Coronavirus 2 (SARS-CoV2) from acute, pyrexial childhood illness can be challenging. We present a case series from two tertiary centres in Cape Town, South Africa and compare the clinical phenotype of MIS-C with mimicking systemic inflammatory disorders.

**Objectives:** 1. Describe the clinical characteristics of children with MIS-C in the region.

2. Compare the clinical features of children with confirmed MIS-C to those who presented during the same period with suspected MIS-C and ultimately an alternative diagnosis of inflammatory or infective conditions (inflammatory controls).

**Methods:** Children with MIS-C admitted to the Red Cross War Memorial Children’s Hospital (RXH) and Tygerberg Hospital (TBH) between 22 June 2020 and 5 March 2021 were recruited. At RXH only, children with suspected MIS-C with an ultimate alternate diagnosis (inflammatory controls) were also recruited. Clinical data were collected.

**Results:** During the time period, 70 children had confirmed MIS-C and 27 suspected MIS-C cases had an alternate diagnosis including typhoid, tuberculosis, sepsis and appendicitis among others. Sixty five percent of children with MIS-C had no SARS-CoV2 contact but all had evidence of SARS-CoV2 exposure by antibody (90%) or Polymerase Chain Reaction (PCR) tests (14%). There was no difference in age, sex or ethnic distribution between children with MIS-C and inflammatory controls (Table 1). The most common presenting features of MIS-C were fever (100%), tachycardia (99%), rash (86%), conjunctivitis (79%), and abdominal pain (60%). Compared to inflammatory controls, the presence of tachycardia, abdominal pain and conjunctivitis resulted in 96%; 93% and 91% respectively increased odds of a diagnosis of MIS-C after controlling for all other presenting features. Compared to inflammatory controls, children with MIS-C had lower platelets, sodium and albumin and higher troponin-T and pro-brain natriuretic peptide (pro-BNP) (Table 1).

The median minimum ejection fraction in MIS-C was lower than inflammatory controls (52% vs 63%, p=0.048). Ninety four percent of MIS-C patients received at least one dose of intravenous immunoglobulin (IVIG), 63% required methylprednisolone and 6% received IL-6 inhibition. Children with MIS-C were more commonly admitted to ICU compared to inflammatory controls (38% vs 12.5%, p=0.013) although there was no difference in mean hospital stay which was 8.2 days in MIS-C. There was no difference in requirement for inotropes (p=0.142) or ventilation (p=0.493). No children died.

**Conclusion:** Distinguishing MIS-C from acute infectious or inflammatory causes of childhood fever may be challenging. The presence of conjunctivitis, tachycardia or abdominal pain associates with higher odds of MIS-C in this population. Differences in widely available blood tests like sodium, albumin and platelets may be useful to differentiate MIS-C in the acute setting.

**Patient Consent Received**

Yes

**Disclosure of Interest**

None declared

**Table 1 (abstract P354).** See text for description

	MIS-C N=70	Inflammatory Controls N=27	p-value
Median age (IQR)	7.0 (2.4, 9.7)	5.3 (2.0, 9.5)	0.53
Sex male (%)	36 (51.4)	16 (59.2)	0.506
Ethnicity black African (%)	43 (61.4)	14 (51.8)	0.216
Min platelets (x10^9^/L)	219.2	300.4 N=25	0.021*
Min sodium (mmol/L)	128.9	133.8 N=26	<0.001*
Min albumin (g/L)	27.6 N=65	32.1 N=16	0.015*
Max troponin-T (ng/L)	79.5 N=46	11.7 N=14	0.001*
Max pro-BNP (ng/L)	11981.4 N=63	2712.4 N=19	0.002*

### P355 An international registry on COVID-19 related hyperinflammation in children and young adults (HyperPED-COVID)

#### R. Caorsi^1^, C. Bracaglia^2^, A. Consolaro^1,3^, F. Minoia^4^, P. Brogan^5^, C. Wouters^6^, F. Candotti^7^, I. Meyts^8^, F. De Benedetti^2^, N. Ruperto^9^, M. Gattorno^1^ on behalf of the Steering Committee for HyperPED-COVID registry

##### ^1^Clinica pediatrica e reumatologia, IRCCS Istituto Giannina Gaslini, Genova; ^2^Division of Rheumatology, IRCCS Ospedale Pediatrico Bambino Gesù, Roma; ^3^DiNOGMI, Università di Genova, Genova; ^4^Pediatric rheumatology, Fondazione IRCCS Ca' Granda-Ospedale Maggiore Policlinico, Milano, Italy; ^5^UCL GOS Institute of Child Health, London, United Kingdom; ^6^Pediatric Rheumatology and Immune-inflammatory diseases, University of Leuven, Leuven, Belgium; ^7^Division of Immunology and Allergy, Laboratory of Inherited Immune Disorders, CHUV, Lausanne, Switzerland; ^8^Department of Microbiology, Immunology and Transplantation, University of Leuven, Leuven, Belgium; ^9^Clinica Pediatrica e Reumatologia, PRINTO, IRCCS Istituto Giannina Gaslini, Genova, Italy

###### **Correspondence:** R. Caorsi

**Introduction:** By now, most of the children with COVID-19 infection have only mild symptoms. However, in the past year a small number of children have developed a serious inflammatory condition in temporal association with COVID-19 pandemic. This condition, named Multisystem Inflammatory Syndrome in Children (MIS-C), is characterized by a systemic inflammation with multiorgan failure; the clinical and laboratory features are similar to those usually observed in Kawasaki disease, cytokine storm syndrome and macrophage activation syndrome. While the SARS-CoV-2 PCR on nasal swab is positive only in a minority of patients, the serology is positive in most of them.

**Objectives:** to create an International multicenter collection of patients with MIS-C involving the main pediatric networks committed in the care of patients with hyperinflammatory conditions.

Primary endpoint of the project is to collect information on clinical presentation, laboratory parameters, clinical outcome and response to treatment of patients with MIS-C. Secondary endpoints of the project are: 1) to analyse different clusters in clinical phenotype in relation to age and geographical location, 2) to identify clinical and laboratory predictors of disease severity and outcome, 3) to evaluate the availability of samples of patients in the repositories linked to the different centers.

**Methods:** a steering committee constituted by representatives of ERN-RITA, PRES, ESID and ISSAID and with the coordination of PRINTO developed a shared form to collect clinical manifestations, laboratory features, response to treatment and outcome of patients with MIS-C. The registry is available online on PRINTO website (www.printo.it).

**Results:** a first survey between centers members of PRINTO network identified 365 patients from 51 centers (43 countries). Currently, 180 patients have been enrolled in the registry from 25 centers worldwide. Moreover, 11 additional centers have been activated and are ready to start enrollement. Ethical committee submission or activation procedures are ongoing in 36 more centers. 31 European countries and 12 extra-European countries are actively involved.

**Conclusion:** After some interesting national initiatives, the HyperPED-COVID registry will give the chance to analyze the impact of Multisystem Inflammatory Syndrome Children at the European and extra-European level, giving the possibility to analyze the distribution, clinical presentation and long-term outcome of this condition in different countries. The registry can also represent the basis for further biological and genetic studies in these patients.

**Disclosure of Interest**

None declared

### P356 Familial mediterranean fever and COVID-19: a monocentric Italian study

#### M. Carrabba^1^, M. Zarantonello^2^, V. Di Stefano^2^, A. D. Di Mauro^2^, G. Fabio^1^

##### ^1^Internal Medicine, Fondazione IRCCS Ca' Granda Ospedale Maggiore Policlinico; ^2^Clinical Sciences and Community Health, Università degli Studi, Milan, Italy

###### **Correspondence:** M. Carrabba

**Introduction:** The severe acute respiratory syndrome-coronavirus-2 (SARS-CoV-2) is the causative agent of the coronavirus disease 2019 (COVID-19). Its rapid spreading all over the world has caused a pandemia that has a deleterious impact on public health worldwide.

The COVID-19 is associated with cytokines dysregulation, increased IL-1β tumor necrosis factor (TNF) and IL-6 release with hyperinflammation and risk of cytokine storm syndrome.

Autoinflammatory diseases (AID) are rare, potentially life-threatening conditions caused by pathogenic gene variants encoding for inflammasomes leading to excessive production of pro-inflammatory cytokines. The innate immune system that plays a critical role in the pathogenesis of Familiar Mediterranean Fever (FMF) is also essential in antiviral defense.

Colchicine is the main drug therapy for FMF and fulfills its role with the inhibition of microtubule polymerization and pyrin inflammasome. Colchicine effects on COVID-19 have not yet been evaluated in trials, but some studies on COVID-19 patients under colchicine treatment for FMF arouse interest. Anti-SARS-COV-2 vaccination is of key role for COVID-19 eradication worldwide, few data are available for AID patients.

**Objectives:** This study aims to describe the COVID-19 impact on patients under regular treatment for AID and their outcome after anti-SARS-COV-2 vaccination.

**Methods:** An observational monocentric study has been conduct on a cohort of Italian AID patients who were under regular follow up for their disorders. Data have been collected both retrospectively and prospectively.

**Results:** Seventy-eight adult AID patients (65 FMF, 5 PFAPA, 3 FCAS, 2 TRAPS, 2 Behcet Disease, 1 SAPHO,) followed at Center for Primary Immunodeficiency and Autoinflammatory disorders have been considered. Among FMF patients, eight communicated to the center when they got COVID-19 and were followed by regular phone calls. The mean age was 36.9 years, (range 27-60) and they had no other comorbidities. All the patients were on regular colchicine therapy and were on complete remission before COVID-19. They were started with azithromycin (with precaution for colchicine interference) and four were treated also with low-weight heparin prophylaxis. None of the patients need oxygen or hospitalization. Two sisters (MEFV genotype M694V/V726A) had FMF attack relapsing during COVID-19 disease, despite regular colchicine therapy. All the cases recovered from COVID-19 without complications.

None of the other AID patients declared to have COVID-19, and all the swabs, that they did when they had symptoms suspected for, were negative.

Genetics of FMF patients’ with COVID-19

- 2 M694V/V726A
- 2 M694V
- 1 M694V/M694V
- 1 M680IGC/E148Q
- 1 R761H/E148Q
- 1 -/-

Till now 31 AID patients have been vaccinated (22 FMF, 4 PFAPA, 3 FCAS, 2 Behcet disease). No adverse reactions were observed. All the patients who had COVID-19 have been vaccinated (Pfizer/Biontech or Moderna), two had FMF attack. Two patients with PFAPA had a mild attack. Post-vaccination titre was checked after 4 week since 2nd dose for those patients who were under anti-IL inhibitors (n=5) or anti-TNF (n=1).

**Conclusion:** In this study, the FMF patients who had COVID-19 under chronic colchicine treatment had a mild/moderate disease and recovered without consequences. According to other studies, FMF under colchicine treatment seems not to be a risk factor for severe COVID-19 and colchicine anti-inflammatory effect worths to be considered for treatment in COVID-19. Anti-SARS-COV-2 vaccination in AID patients of this study has been observed to be safe and effective.

**Patient Consent Received**

Yes

**Disclosure of Interest**

None declared

### P357 Multisystem inflammatory syndrome in children: results of a multicenter Italian survey during SARS-COV2 pandemic

#### F. Zunica^1^, S. Della Paolera^2^, M. Giangreco^3^, F. La Torre^4^, C. Bracaglia^5^, G. Filocamo^6^, D. Montin^7^, A. Villani^8^, R. Cimaz^9^, A. Ravelli^10^, M. Cattalini^11^, A. Taddio^12^

##### ^1^Children’s Hospital V. Buzzi, Milan; ^2^Pediatrics, Institute for Maternal and Child Health; ^3^Institute for Maternal and Child Health, University of Trieste, Trieste; ^4^Pediatric Rheumatology Center, Pediatric Unit, “Giovanni XXIII” Pediatric Hospital, Bari; ^5^Division of Rheumatology, Bambino Gesù Children's Hospital IRCCS, Rome; ^6^Fondazione IRCCS Ca’ Granda, Ospedale Maggiore Policlinico, Milan; ^7^Department of Pediatrics and Public Health, University of Turin, Turin; ^8^Pediatrics, Bambino Gesu' Children's Hospital, Rome; ^9^Pediatric Rheumatology, ASST Gaetano Pini-CTO, University of Milan, Milan; ^10^Pediatrics and Rheumatology Unit, IRCCS Istituto Giannina Gaslini,University of Genoa, Genoa; ^11^Pediatrics Clinic, ASST Spedali Civili di Brescia, University of Brescia, Brescia; ^12^Institute for Maternal and Child Health, IRCCS "Burlo Garofolo, 3University of Trieste, Trieste, Italy

###### **Correspondence:** M. Cattalini

**Introduction:** Multisystem Inflammatory Syndrome in children (MIS-C) was initially described during the first phase of COVID-19 pandemic as a severe clinical condition with systemic inflammation and multi-organ involvement. We previously published the results of the Italian multicenter survey of MIS-C, launched by the Rheumatology Study Group of Italian Pediatric Society We suggested that SARS-Cov-2 might determine two types of inflammatory diseases in children: the classic KD, that could be triggered by the coronavirus, and the multisystem inflammatory syndrome, which has some specific clinical peculiarities.

**Objectives:** The aim of our study was to analyze clinical features, laboratory findings and treatment strategies in patients diagnosed with MIS-C in Italy during SARS-CoV2 pandemic and evaluate if different outcomes may be related to different disease phenotype in order to establish specific prognostic criteria.

**Methods:** This is an observational, retrospective, multicenter study. We enrolled the children hospitalized between September 1^st^ 2020 and April 30^th^, 2021 with clinical diagnosis of multi-inflammatory syndrome (MIS-C). For each patient who received MISC diagnosis, we collected demographic, clinical, laboratory data, imaging findings, and treatment information in an online anonymized database (REDCap). We focused on the following main outcomes: the presence of heart abnormalities at dischargement, ICU admission, need of respiratory support or vasoactive agents and number of IVIG cycle administered analyzing a possible relationship with different disease phenotype.

**Results:** 186 children were included in the study. The median age at presentation was 8 years (4-11), 103 (55%) patients were male and 83 (45%) female. 23 (12%) patients had pre-existing comorbidities. 130 (70%) patients presented a positive IgG serology for SARS-CoV-2 and 51% of patients reported a close contact. Markers of systemic inflammation at onset was elevated in all patients: CRP 143,2 mg/dl (111,0- 156,3), ESR 51,5 mm/h (51,0 -54,5), neutrophils 8200/mmc (6490-9011), D-dimer 2175 ng/ml (1076 - 2814). 16 (8%) children needed oxygen supplementation at baseline. 129 patients showed cardiac involvement characterized by myocarditis (23%), valve dysfunction (20%), hypotension (19%) and heart failure (15%). MAS was a complication in 11(6%) patients. ICU admission was required in 40 patients (22%). In our study, a majority of patients were treated with glucocorticoids (77%) and intravenous immunoglobulin (91%), of which 9% receveid two doses of IGEV. At dischargement heart ultrasonography showed valvular insufficiency (19%) and coronary abnormalities (8%).

**Conclusion:** MIS-C has an extensive clinical spectrum that led to serious and life-threating illness. Systemic inflammation and specific organ involvement of cardiac and gastrointestinal involvement are the hallmarks. Good outcomes depends on prompt recognition and timely treatment, based on the combined use of glucocorticoids, high-dose immunoglobulins and anti-cytokine therapy.

**Disclosure of Interest**

None declared

### P358 Pediatric multisystemic inflammatory syndrome with neurological involvement due to SARS-COV-2

#### N. L. De La Rosa, M. I. De la Cera Rodriguez, H. Menchaca Aguayo, E. Mercedes Pérez, P. Ramos Tiñini, E. Faugier Fuentes, S. Rodriguez Aguayo

##### Reumatología Pediátrica, Hospital Infantil de México Federico Gómez, Ciudad Mexico, Mexico

###### **Correspondence:** N. L. De La Rosa

**Introduction:** Pediatric multisystemic inflammatory syndrome (PIMS) is characterized by the appearance of persistent fever associated with hypotension and multisystem compromise. SARS-COV 2 is a neuroinvasive virus with neurological manifestations, caused by the cytokine storm, which causes an exaggerated response such as meningoencephalitis.

**Objectives:** Describe a case of pediatric multisystemic inflammatory syndrome with neurological involvement due to SARS-COV-2

**Methods:** We present the case of a 4-month-old girl who presented a pediatric multisystemic inflammatory syndrome with neurological involvement due to SARS-COV-2, treated with intravenous immunoglobulins (IVIG) and methylprednisolone pulses.

**Results:** 4-month-old female. Epidemiological contact: maternal grandfather with COVID-19 4 weeks previously. Clinical picture: 5-day fever (40 ° C), irritability, sporadic cough, generalized maculopapular rash, non-suppurative conjunctivitis, edema of the hands and feet. On admission irritable, drowsy, bulging anterior fontanelle, tachycardia, bulging pulses, flash capillary filling. He required oxygen, crystalloid charges, and admission to intensive care. It was integrated (PIMS) and meningoencephalitis. Cerebrospinal fluid (CSF): protein spinal cord and leukocytosis, SARS COV2 positive in CSF and serum. Echocardiogram, chest X-ray and skull tomography without alteration. Treatment: gamma globulin (2gkgd), methylprednisolone (2mgkgd) and enoxaparin (1mgkgd). Discharged without sequelae 6 days after hospitalization.

**Conclusion:** We report the exceptional case of SARS-COV-2 meningoencephalitis with positive CRP in the CSF. The comprehensive evaluation of the patient with neurological compromise due to SARS-COVS-2 must be thorough. PIMS is associated with: lethargy and drowsiness. In the present case, the relevant clinical data was irritability. A comprehensive approach to the patient is imperative in the face of the diversity of the infectious behavior of sars-cov-2.

**Disclosure of Interest**

None declared

### P359 Early echocardiographic and cardiac MRI findings in multisystem inflammatory syndrome in children (MIS-C)

#### D. Sirico^1^, A. Basso^1^, M. Fastiggi^2^, A. Meneghel^2^, G. Martini^2^, E. Reffo^1^, A. Cavaliere^3^, B. Castaldi^1^, F. Zulian^2^, G. Di Salvo^1^

##### ^1^Department of Women’s and Children’s Health, Pediatric Cardiology; ^2^Department of Women’s and Children’s Health, Pediatric Rheumatology; ^3^Institute of Radiology, University of Padua, Padua, Italy

###### **Correspondence:** M. Fastiggi

**Introduction:** Multisystem Inflammatory Syndrome in Children (MIS-C) is a known severe condition affecting children previously exposed to SARS-CoV-2. Cardiovascular manifestations in MIS-C are quite common and include myocardial dysfunction, coronary artery dilation or aneurysms, arrhythmias, conduction abnormalities, pericarditis and valvulitis. Severe cases can present even with cardiogenic shock. To date, little is known about the very early myocardial abnormalities in pediatric patients with MIS-C. The Speckle Tracking Echocardiography (STE) and cardiac MRI (cMRI) have shown to be potential candidate for identifying regional ventricular dysfunctions in early stages of inflammatory COVID-related conditions [1,2].

**Objectives:** To describe the early cardiac findings in patients with MIS-C, evaluated by two advanced cardiovascular imaging, STE and cMRI.

**Methods:** Consecutive patients with MIS-C underwent standard transthoracic echocardiography (TTE), speckle-tracking echocardiography (STE) with analysis of left ventricle (LV) global longitudinal strain (GLS) and cardiac MRI (cMRI). Clinical and laboratory data, including markers of systemic inflammation, Troponin I (TnI) and Brain Natriuretic Peptide (BNP) were also collected at onset and during follow up. All patients received intravenous immunoglobulins (IVIGs), intravenous corticosteroids (methylprednisolone) and antiplatelet therapy (aspirin). The use of biological agents (Anakinra) was reserved to patients with severe or critical illness. The need for Intensive Care Unit (ICU) was based on clinical and hemodynamic status at presentation.

**Results:** Twenty-three patients (13M, 10F), mean age 8.1±4years (range 5.4-15.7), all with positive clinical and/or serological evidence of previous SARS-COV2 infection, entered the study. The majority (78.2%) was caucasian. All presented high degree fever, gastrointestinal symptoms and rash. Conjunctivitis and cardiovascular symptoms, as hypotension, thoracic pain or dysrhythmia, were present in 10 (43.5%). Nine children (39.1%) shared Kawasaki Disease-like symptoms. Four patients (17.4%) needed ICU admission and 3 required inotropic support. Short-term survival was 100%. All patients showed an hyperinflammatory state with elevated CRP, ESR, and D-Dimer. Tn-I was abnormal (>34 ng/L) in 15 patients (65.2%), BNP was significantly elevated in 20 (86.9%). Median time to STE evaluation was 8 days and to cMRI was 18 days since fever onset. Mean LVEF and RVEF were respectively 59±10% and 45±7%. Coronary dilation was observed in 6 (26.1%) patients. STE showed reduced mean LVGLS (-17±4.3%). LVEF on cMR was 60±13%, LGE with non-ischemic pattern was evident in 6/16 patients (37.5%) and pericardial effusion in 2 (12.5%).

**Conclusion:** MIS-C can occur in a small but not negligible proportion of children previously affected by COVID-19 and affects the heart in a significant proportion of them. STE and cMRI were shown to be very sensitive tools to evaluate and monitor the early cardiac dysfunctions in patients with MIS-C. The elevation of myocardial necrosis markers, the myocardial injury confirmed by reduced LVGLS and presence of LGE on cMR in about a quarter of the patients support the pathogenetic hypothesis of a post-viral immuno-mediated myocarditis.

**References**

1) Baycan OF et al. Int J Cardiovasc Imaging 2021;37(1):135-144.

2) Huang L. et al. JACC Cardiovasc Imaging. 2020;13:2330–2339.

**Disclosure of Interest**

None declared

### P360 Non convulsive status epilepticus as ongoing symptom of multisystem inflammatory syndrome in children (MIS-C)

#### M. Fastiggi^1^, M. L. Cagnato^2^, S. Sartori^3^, S. Masiero^2^, L. Da Dalt^2^, F. Zulian^1^, G. Martini^1^

##### ^1^Pediatric Rheumatology Unit, Department of Women’s and Children’s Health; ^2^Pediatric Emergency Department, Department of Women’s and Children’s Health; ^3^Paediatric Neurology and Neurophysiology Unit, Department of Women’s and Children’s Health, University Hospital of Padua, Padua, Italy

###### **Correspondence:** M. Fastiggi

**Introduction:** Since April 2020, Multisystem inflammatory syndrome in children (MIS-C) has been reported worldwide and associated with a different spectrum of symptoms. Although mild neurological manifestations in SARS-Cov2 infection and MIS-C have been reported, severe involvement with brain abnormalities, is rare ^1^.

**Objectives:** Describe a child with MIS-C presenting non-convulsive status epilepticus associated with abnormal cerebral magnetic resonance (MR), never previously reported.

**Methods:** Case report.

**Results:** A previously healthy 19-month-old girl presented to our emergency department after a prolonged febrile seizure involving the right side of her body lasting about 25 minutes. She presented with fever lasting more than 24 hours. On physical examination, abdominal distention and tenderness and altered mental status with irritability were detected. RT-PCR for SARS-CoV-2 on nasal swab was negative but her parents had SARS-CoV-2 infection four weeks earlier. Laboratory showed elevated CRP (35 mg/L), while all microbiological analyses in blood, urine and CSF were negative. Computerized tomography (TC) showed a doubtful left temporal hypointensity, and cerebral MRI displayed cytotoxic oedema in left temporal mesial area of the brain on diffusion-weighted imaging (DWI). Few day later, her clinical conditions worsened with irritability and drowsiness associated with persistent abdominal distention, diarrhoea, and high fever. The EEG revealed a pattern suggestive for non-convulsive status epilepticus responsive to benzodiazepines and loading dose of Levetiracetam. Consensually, an increase of inflammatory markers (CRP 153 mg/L, procalcitonin 114 ug/L) was observed. Chest X-ray, EKG, troponin and BNP levels were normal, whereas echocardiogram demonstrated left ventricular diastolic dysfunction and mild pericardial effusion. In the suspicion of MIS-C with abdominal, cardiac and neurological involvement, she was treated with intravenous immunoglobulin (2g/kg), methylprednisolone (2 mg/kg) and acetylsalicylic acid (5mg/kg). Serum SARS-Cov2 antibody test resulted positive for previous infection, confirming the diagnosis of MIS-C. Neuronal antibodies for immune-mediated CNS disorders tested negative. Within 36 hours from therapy start, a significant improvement in general conditions, along with stable apyrexia and decreasing in inflammatory markers were observed. She was discharged two weeks later on oral steroids, ASA and Levetiracetam; the physical examination was normal, and EEG showed a global improvement in brain electrical activity.

**Conclusion:** Neurological symptoms secondary to SARS-Cov2 infection and MIS-C have been reported in children (1) but only a few present severe neurological complications such as status epilepticus. Non-convulsive status epilepticus has been previously described in an adult with acute COVID19^2^ but has never been reported as presenting sign of MIS-C. The current case illustrates the need of a careful neurological evaluation in children with MIS-C, as CNS involvement can represent the main clinical presentation thus underlining the need of an appropriate diagnostic and therapeutic approach.

**References**

1) Omar AM et al. Neurologic and Radiographic Findings Associated With COVID-19 Infection in Children *JAMA Neurol.* 2020;77(11):1440-1445.

2) Rodrigo-Armenteros P. et al. Non-convulsive status epilepticus in a patient with COVID-19 infection. Clin Neurophysiol. 2020 Nov; 131(11): 2588–2590.

**Patient Consent Received**

Yes

**Disclosure of Interest**

None declared

### P361 PET-MRI for the detection of minimal residual disease in MIS-C: a long-term prospective study

#### M. Fastiggi^1^, A. Meneghel^1^, G. Martini^1^, C. Giraudo^2^, D. Cecchin^3^, F. Zulian^1^

##### ^1^Department of Women’s and Children’s Health, Pediatric Rheumatology; ^2^Institute of Radiology; ^3^Institute of Nuclear Medicine, University of Padua, Padua, Italy

###### **Correspondence:** M. Fastiggi

**Introduction:** Multisystem Inflammatory Syndrome in Children (MIS-C) is an emerging clinical condition, similar to the hyperinflammatory response seen in adults with COVID-19^1^. To date, little is known about the natural history of the disease and the long-term monitoring of MIS-C patients. Positron emission tomography PET/MRI is actually used to identify active inflammatory or neoplastic sites using [^18^F]fluorodeoxyglucose (FDG) due to the high glycolytic metabolism of inflammatory/neoplastic tissues^2^. Therefore, it could be indicated to evaluate and monitor the inflammatory disease state^2^.

**Objectives:** To describe the PET/MRI findings for the evaluation of the minimal residual disease in a cohort of patients with MIS-C.

**Methods:** Consecutive patients with MIS-C underwent a whole body FDG PET/MRI by 2 weeks, when possible, and at 6 weeks after the onset of fever. Each patient, after a 36 hours of fasting and high-fat low carbohydrate (<5g/day) diet preparation, was scanned using 3 MBq/kg FDG to minimize the radiation exposure. Clinical and laboratory data were also collected at onset and during follow up.

**Results:** Ten patients (7M, 3F), mean age 10.2 years (range 5.4-17.7), all with positive clinical and/or serological evidence of previous SARS-COV2 infection, entered the study. All presented high degree fever, gastrointestinal symptoms and rash. Conjunctivitis and cardiovascular involvement, as hypotension, significant myocardial dysfunction and increased myocardiolysis markers, were also present in half of them. Only one patient needed intensive care support for five days. Systemic inflammatory and prothrombotic markers were elevated in all patients on admission (mean CRP 166.3 mg/L; procalcitonin 11.8 ug/L; D-dimer 2348 ug/L, ferritin 1135 ng/L). All patients were treated, 4.5 (± 1.5) days from fever onset with pulse IVIG (2 g/kg) and IV methyprednisone (MPDN 2 mg/kg/day, max 80 mg) for 2 weeks then with oral PDN tapered down to 0 in further 4 weeks. PET/MRI was performed 13.3 days (± 1.5) after fever onset in three patients and 48 days (± 10.6) in 8.

During the acute phase, all patients showed pelvic effusion and edema of the abdominal wall tissues at the total body MRI, not seen in patients during the late phase. Lymph node involvement was present in 81% of MRI findings. The cervical district appeared to be the most involved one as compared to the thoracic, mesenteric and retroperitoneal ones (72% vs 45, 36 and 45% respectively). However, a residual mesenteric lymphadenopathy was exclusive to the late phase (5/8 patients).

**Conclusion:** PET/MRI confirms the good metabolic response to treatment in patients with MIS-C. The abdominal region is more intensively involved in the early stage of the disease, likely related to the hyperinflammatory state. A slow normalization through the lymph node compartment is present in the late stage. PET/MRI is a highly sensitive and specific tool for assessing minimal residual disease in MIS-C and should be indicated for patients with incomplete clinical response to treatment.

**References**

1) Li Jiang, et al. COVID-19 and multisystem inflammatory syndrome in children and adolescents. Lancet Infect Dis 2020; 20: e276–88.

2) Treglia G et al. Diagnostic Performance of ^18^ F-FDG PET/CT in Infectious and Inflammatory Diseases according to Published Meta-Analyses. Contrast Media Mol. Imaging 2019:3018349.

**Disclosure of Interest**

None declared

### P362 Case series of children with multisystem inflammatory syndrome following SARS-COV-2 infection in Switzerland

#### A. Fouriki^1^, T. Arlabosse^1^, C. Schnider^1^, K. Theodoropoulou^1^, Y. Fougère^1^, G. Blanchard Rohner^2^, S. Grazioli^3^, N. Wagner^3^, J. Pachlopnik Schmid^4^, J. Truck^4^, L. Kottanatu^5^, M.-H. Perez^6^, D. Schaffner^6^, S. Asner^6^, M. Hofer^6^

##### ^1^DFME, CHUV, Lausanne; ^2^HUG, Geneve; ^3^HUG, Genève; ^4^Kinderspital Zürich, Zurich; ^5^Bellinzona Hospital, Bellinzona; ^6^CHUV, Lausanne, Switzerland

###### **Correspondence:** A. Fouriki

**Introduction:** Since the beginning of the severe SARS-CoV-2 pandemic, an increasing number of countries reported cases of a systemic hyperinflammatory condition defined as multi-system inflammatory syndrome in children (MIS-C). The clinical features of MIS-C can be an overlap of Kawasaki Disease (KD), Toxic Shock Syndrome (TSS), Macrophage Activation Syndrome (MAS) or can have an acute abdominal presentation.

**Objectives:** We report the demographic profile, clinical presentation, management, and outcome of this emerging syndrome in 15 children in Switzerland. Understanding these parameters will enable streamlining of early diagnosis and treatment, thus leading to a favorable outcome.

**Methods:** Our clinical study is a case series that includes patients identified during the study registration period (consecutive, formal) at Geneva, Zurich and Lausanne. The data for most of Lausanne patients were extracted from JIR cohort database. This is an observational (descriptive research design), retrospective, multicentric study of a case series.

**Results:** The socio-demographic profile showed male predilection (12 male patients (80%) and 3 female), with no significant racial predisposition.

Concerning the symptoms, fever was always present (15/15, *100%*). A high incidence of gastrointestinal (10/15 with abdominal pain *(67%)*, 10/15 with emesis *(67%)* and 9/15 with diarrheas *(60%)*- these symptoms did not necessarily overlap) and mucocutaneous symptoms (rash 9/15 *(60%)*, conjunctival ejection 11/15 *(73%)*, cheilitis 3/15 *(20%))* and extremity changes 9/15 *(60%))* was noted. Other manifestations included adenopathy (6/15, *40%*), neurological symptoms (6/15, *40%*), respiratory insufficiency (10/15, *67%*), shock (8/15, *53%*), cardiac abnormalities (9/15, *60%*)- either like coronary artery anomaly (5/15, *33%*) or left ventricular dysfunction (7/15, *47%*).

Concerning the biological profile, serological evidence of SARS-CoV-2 was present in all our patients (15/15, *100%*) as was also the case for the elevated inflammatory markers (C-reactive protein and sedimentation rate). Serum cytokine profile investigations showed increased IL1RA levels in all tested patients (6/6 patients, 9 patients had not been tested) and increased IL6 levels for 3 patients (3/7 patients tested).

Concerning the treatment, 13/15 patients (*87%*) received at least one dose of intravenous immunoglobulins (IVIG), while 8/15 (*53%*) patients also had a steroid treatment. One patient did not need any treatment except from supportive care. One patient received only anakinra with favorable evolution, and five combined with other treatments (6/15 in total, *40%*). The treatments combined with anakinra were: IVIG for all the 5 patients; 4 of them received additionally corticosteroids, while 2 patients further needed one dose of Tocilizumab (anti-IL6 treatment, because of their cytokine profile demonstrating high levels of IL6).

Nine out of 15 (*60%*) patients were transferred to the PICU (pediatric intensive care unit), and there were no deaths. Most of the patients (14/15) recovered fully and only one showed long COVID symptoms.

**Conclusion:** Awareness about post COVID inflammatory syndrome should be raised among pediatricians because early diagnosis and management of this syndrome by a multidisciplinary specialized team can lead to a favorable outcome. In conclusion, our case series reports on clinical and laboratory findings, as well as on the management of Swiss cases with MIS-C.

**Patient Consent Received**

Yes

**Disclosure of Interest**

None declared

### P363 The frequency and clinical course of SARS-COV2 infection in children with juvenile idiopathic arthritis

#### M. F. Gicchino, A. Amodio, E. Miraglia del Giudice, F. Abbate, A. N. Olivieri

##### Department of Woman, Child and General and Specialistic Surgery, University of the Study of Campania Luigi Vanvitelli, Naples, Italy

###### **Correspondence:** M. F. Gicchino

**Introduction:** Severe acute respiratory syndrome coronavirus-2 (SARS-CoV-2), first reported from the Wuhan city of China in December 2019, swept the world in a few months and became a global health emergency of primary international concern continues to be a priority health problem. Recent studies suggest that subjects with autoimmune disorders (JIA, RA, SLE) do not have an increased risk to get Sars-Cov2. Juvenile Idiopathic Arthritis (JIA) is an inflammatory chronic disease concerning joints and others structures. According to International League of Association for Rheumatology (ILAR) seven subtypes of arthritis can be defined in relation with the number of joints and the extra-articular involvement occurring in the first six months of disease. NSAIDs and intra-articular steroids represent the first line treatment for JIA. Systemic steroids, disease modifying anti-rheumatic drugs (DMARDs) and biologic drugs are used in children with severe disease.

**Objectives:** To evaluate the incidence and the impact on the disease course of Sars-cov2 infection in a group of children affected from juvenile idiopathic arthritis in treatment with Methotrexate and/or biological drugs.

**Methods:** This study includes 77 children affected from JIA (55 females, 22 males). JIA diagnosis was made according to ILAR criteria and treatment was assigned with recommendations of the American College of Rheumatology. For each patient we recorded the type and the duration of pharmacological treatment, JIA subtype and relapses (defined according to Wallace criteria). During follow up visit from Nov 1st, 2020 to April 30th, 2021 we investigated in each patient history of Sars-Cov2 infection and related symptoms, diagnostic tests for Sars-Cov2.

**Results:** The mean age at the last follow-up visit was 13.3±5.61 years. Thirteen out of 77 patients was affected Sars-Cov2 infection, none of them needed to be hospitalized. The most common symptom was headache (8 of 13 patients), followed by myalgia (6 of 13), fever (4 of 13), anosmia or dysgeusia (3 of 13), upper respiratory tract symptoms (3 of 13) and nausea (3 of 13). Five out of 13 patients were asymptomatic (38,5%). The mean duration of Sars-Cov2 infection was 12,5 days (confirmed with nose pharyngeal swab). In the group of patients with Sars-Cov2 infection 4 of 13 (30%) were in treatment with MTX, 2 of 13 (15%) with both MTX and biological drugs, 5 of 13(38%) with biological drugs, 2 of 13 (15%) with NSAIDs. In the group of patients without Sars-Cov2 infection 37 of 64 (48%) patients were in treatment with MTX, 32 of 64 (41,5%) with biological drugs, 18 of 64 (28%) with both MTX and biological drugs, 10 of 64 (15,6%) with NSAIDs. We didn’t find a higher risk to contract Sars-Cov2 infection in patients under MTX treatment (48% vs 46.15 %; p value 0.6416), in ones under MTX and biological drugs (28.1% vs 15.4%p value 0.543) or in ones under only biological therapy (50% vs 53.8% ; p value 0.231). We found that in patient with JIA the risk to get Sars-Cov2 infection is not related to the treatment. The percentage of JIA relapses was higher in patients with Sars-Cov2 infection than in the ones without infection (53,8% vs 9%; p value 0.0004).

**Conclusion:** Treatment with MTX or biological drugs did not increase the risk to get Sars-Cov2 infection. The frequency of JIA relapses was higher in patients who got Sars-Cov2 infection than in ones who didn’t get it.

**Patient Consent Received**

Yes

**Disclosure of Interest**

None declared

### P364 Kawasaki-like syndrome associated with COVID-19: clinical characteristics of mild to moderate forms of a disease

#### M. Golubovic, V. Nikolic, M. Jakovljevic, A. Ognjanovic, M. Stojkovic, Z. Gocic, M. Karadzic, A. Stankovic, M. Milenkovic, J. Vojinovic

##### Clinic of Pediatrics, University Clinical Center Nis, Nis, Serbia, Niš, Serbia

###### **Correspondence:** M. Golubovic

**Introduction:** In setting of global pandemic of coronavirus disease 19 (COVID-19) caused by severe acute respiratory syndrome coronavirus 2 (SARS-CoV-2), cases resembling Kawasaki disease (KD) were repeatedly reported. Soon afterwards it began to be considered as separate entity named multisystem inflammatory syndrome in children (MIS-C). In addition to this newly recognized syndrome which has overlapping features with KD and is still occasionally described as its from, during COVID-19, a high incidence of Kawasaki-like syndrome (KLS) with mild to moderate symptoms was also noted. Recently, it was proposed that even such cases should, in clinical context, be treated as MIS-C if appropriate criteria are met. On the other hand, clear distinction of the two entities can help researchers to answer the question of its etiology and pathogenesis and could direct the clinicians what to expect during the course of the disease.

**Objectives:** The aim was to describe main epidemiologic and clinical characteristics of KLS during the first year of COVID-19 pandemic.

**Methods:** This retrospective study included analysis of medical documentation of patients treated for KLS at Clinic of Pediatrics, University Clinical Center Niš, between March 2020 and 2021. Inclusion criteria was fulfillment of KD or KLS criteria, based on CDC^1^ or WHO^2^ recommendation. Severe form of the disease with signs of a shock was exclusion criteria. The results were elaborated with the statistical method of descriptive and quantitative analysis.

**Results:** A total of 21 subjects fulfilled the criteria for KLS during the study period. The cumulative incidence was 8.48 per 10^5^ minors. Male to female ratio was 2:1 and mean age at diagnosis was 6.5 years (min. 8 months; max. 17 years). In fourteen cases current or recent COVID-19 infection or confirmed COVID-19 exposure was observed. When only these patients are analyzed average age was 8.2 years. Antibodies against SARS-CoV-2 were confirmed in seven patients, three had positive PCR test for COVID-19 and one had close contact with someone who has COVID-19 but COVID-19 was not proved. Interestingly, three patients that were antigen positive had respiratory organ involvement. In the reaming seven cases all the other possible causes of symptoms were ruled out and possible contact with SARS-CoV-2 was assumed.

In addition to prolonged fever, that was present in all patients, polymorphous rash (95%) was the most common clinical feature flowed by bilateral nonpurulent conjunctivitis (47%). Gastrointestinal tract involvement was the most common internal organ manifestation. Treatment included corticosteroids (15 patients), intravenous immunoglobulin (1 patient) and two patients received nonsteroidal anti-inflammatory drug in anti-inflammatory dose, while the rest were only treated symptomatically. Favorable outcome was achieved in all patients with no morphological changes observed on echocardiography during the hospitalization and 2 weeks after discharge.

**Conclusion:** Our findings suggest connection between infection and occurrence of the disease in susceptible children. Yet, a large portion of the population had contact with SARS-CoV-2, thus the exact role of infectious agent and pathophysiological mechanisms have to be determined. Gender distribution with male dominance among our patients is expected. Still, average age at diagnosis was bit higher than what is usually observed in classic KD and is more in line with characteristics of MIS-C.

Further research are to be done in order to define what determine progression of the disease and are there any signs that may point in which way it will develop. Only in that way the clinicians would made right choices regarding the patients treatment.

**Patient Consent Received**

Yes

**Disclosure of Interest**

None declared

### P365 Asymptomatic SARS-COV-2 seropositivity: patients with childhood onset rheumatic diseases versus healthy children

#### F. Haslak^1^, D. Ozbey^2^, M. Yildiz^1^, A. Adrovic^1^, S. Sahin^1^, O. Koker^1^, A. Aliyeva^1^, V. Guliyeva^1^, G. Yalcin^1^, G. Inanli^1^, B. S. Kocazeybek^2^, O. Kasapcopur^1^, K. Barut^1^

##### ^1^Pediatric Rheumatology; ^2^Microbiology, Istanbul University-Cerrahpasa, Cerrahpasa Medical Faculty, Istanbul, Turkey

###### **Correspondence:** F. Haslak

**Introduction:** Although 10-20% of the Severe Acute Respiratory Syndrome Coronavirus 2 (SARS-CoV-2) infected individuals experience life-threatening events, children are most likely to have a significantly milder COVID-19 disease course. However, they might have a pivotal role in the transmission. Raised concerns regarding the vulnerability of those with several comorbidities led to studies evaluating the patients with rheumatic diseases, but they were not found to be significant risk factors for a severe disease course, neither in childhood nor in adulthood.

**Objectives:** We aimed to find out the asymptomatic SARS-CoV-2 seroprevalence among pediatric patients with rheumatic diseases and healthy children and to compare them with each other.

**Methods:** Patients with familial Mediterranean fever (FMF), juvenile idiopathic arthritis (JIA), juvenile systemic lupus erythematosus (jSLE) and healthy children (HC) who remained asymptomatic during the pandemic are examined by ELISA Immunoglobulin (Ig) A and IgG tests in this cross-sectional study.

**Results:** Overall, 149 subjects (90 females) were included in the study. While IgA was positive in 15 subjects (10%) (HC: 8, jSLE: 3, FMF: 2, JIA: 2; p=0.196), IgG was positive in 14 subjects (9.4%) (HC: 7, JIA: 5, FMF: 1, jSLE: 1; p=0.156). Nineteen subjects (12.75%) were IgA or IgG positive (HC: 8, JIA: 5, jSLE: 3, FMF: 3; p=0.644). Although not significant, seropositivity was more often in HC group. The mean age of the IgA and IgG positive subjects was 11.62 ± 5.11 (p=0.391) and 11.18 ± 5.95 (p=0.433) years, respectively. Ten (66.7%) of the IgA positive subjects (p=0.807), and 9 (64.3%) of the IgG positive subjects (p=0.980) were females. Two of the seropositive subjects had a contact history with a confirmed COVID-19 case (FMF:1, IgA: negative, IgG: positive; HC:1, both IgA and IgG positive). During the pandemic, 3 (20%, p=1) of the IgA positive subjects were receiving hydroxychloroquine (HCQ), 2 (13.3%, p=0.738) were receiving colchicine, one was receiving steroids (6.7%, p=0.694), one was receiving conventional disease modifying anti-rheumatic drugs (cDMARDs) (azathioprine: 1) (6.7%, p=0.116), and one was receiving biologic DMARDs (bDMARDs) (adalimumab: 1) (6.7%, p=0.699). On the other hand, 3 (21.4%, p=0.404) of the IgG positive subjects were receiving steroids, 2 (14.3%, p=1) were receiving bDMARDs (adalimumab: 2), 2 (14.3%, p=0.520) were receiving cDMARDs (azathioprine:1, methotrexate:1), one was receiving colchicine (7.1%, p=0.302), and one was receiving HCQ (7.1%, p=0.467). Both IgA and IgG positivity were not found to be related to age, sex, underlying rheumatic diseases and received treatments of the patients.

**Conclusion:** We revealed that patients with childhood-onset rheumatic diseases, even if they receive immunosuppressive medication such as bDMARDs or cDMARDs, might have an asymptomatic SARS-CoV-2 infection, similarly to their healthy peers.

**Patient Consent Received**

No

**Disclosure of Interest**

None declared

### P366 Clinical features and outcomes OF 76 patients with COVID-19-related multi-system inflammatory syndrome in children

#### F. Haslak^1^, K. Barut^1^, C. Durak^2^, A. Aliyeva^1^, M. Yildiz^1^, V. Guliyeva^1^, S. E. Varol^1^, S. Oral Cebeci ^3^, F. Aygun ^2^, Y. Z. Varli ^4^, A. Ozel ^5^, S. H. Onan^6^, U. Kocoglu^7^, M. Erol ^5^, F. Karagozlu^8^, N. Ulug ^8^, R. Dedeoglu^8^, S. Sahin^9^, A. Adrovic^1^, F. Oztunc^8^, O. Kasapcopur^1^

##### ^1^Pediatric Rheumatology; ^2^Pediatric Intensive Care; ^3^Pediatric Emergency, Istanbul University-Cerrahpasa, Cerrahpasa Medical Faculty; ^4^Pediatrics, Cam and Sakura Hospital; ^5^Pediatrics; ^6^Pediartric Cardiology; ^7^Pediatric Intensive Care, Bagcilar Education and Training Hospital; ^8^Pediartric Cardiology, Istanbul University-Cerrahpasa, Cerrahpasa Medical Faculty; ^9^Pediatric Rheumatology, Cam and Sakura Hospital, Istanbul, Turkey

###### **Correspondence:** F. Haslak

**Introduction:** Given the recent data regarding a broad spectrum of variabilities in clinical and immunologic findings, a complex immune mechanism probably influenced by geographical and ethnic circumstances was thought to play the role in the pathogenesis of COVID-19 related multi-system inflammatory syndrome in children (MIS-C). Therefore, further clinical, and observational studies and case series from all around the world are required for better understanding of disease pathogenesis. Moreover, although there are several studies describing MIS-C patients which were admitted to the pediatric intensive care unit (PICU)s, there is a scarce data regarding the predictors of PICU admission.

**Objectives:** We aimed to describe the clinical features and outcomes of our patients with MIS-C and to evaluate the associated factors for the PICU admission.

**Methods:** The MIS-C patients under 18 years old diagnosed and treated in three referral centers between July 2020 and March 2021 were included. Data of the patients were retrospectively obtained from their medical records.

**Results:** Overall, 76 subjects (24 females) with a mean age of 8.17 ± 4.42 years were enrolled. Twelve (15.8%) patients had an underlying chronic disease (asthma:2, acute lymphoblastic leukemia (ALL): 2, familial Mediterranean fever (FMF): 1, cerebral palsy:1, morbid obesity:1, obsessive-compulsive disorder:1, cyanotic congenital heart disease:1, cardiac rhythm disturbance:1, type 1 diabetes mellitus:1, tuberous sclerosis: 1), and 5 of 12 were under a long-term medication (anti-epileptic drugs: 1, insulin: 1, colchicine: 1, chemotherapy: 1, selective serotonin re-uptake inhibitor: 1). At the onset of MIS-C, the attacks of the patient with FMF were under control by colchicine. The two most common systemic involvement patterns were cardiac and gastrointestinal. Twenty-seven (35.5%) patients were admitted to the PICUs. There was only one lethal outcome in a patient with underlying ALL. Those with higher procalcitonin levels at admission were found to stay longer in the hospital (r=0.254, p=0.027). Older age (aOR: 1.277; 95%C.I.: 1.089-1.498; p: 0.003) and lower initial albumin levels (aOR: 0.105; 95%C.I.: 0.029-0.378; p: 0.001) were found to be significant associated factors for predicting PICU admission in multivariate linear regression analysis.

**Conclusion:** Although there is wide clinical variability among the patients with MIS-C, we suggest that those with older age and lower initial serum albumin levels merit closely monitoring due to their higher risk for PICU admission.

**Patient Consent Received**

No

**Disclosure of Interest**

None declared

### P367 Clinical features and laboratory findings of multisystem inflammatory syndrome in children (MIS-C)

#### M. Jakovljevic^1^, I. Nakev^1^, M. Golubovic^1^, A. Ognjanovic^1^, M. Zdravkoviç^1^, S. Stajic^1^, J. Stankovic^1^, B. Bjelakovic^1,2^, J. Vojinovic^1,2^

##### ^1^Clinic od Paediatric, University Clinical Center of Nis; ^2^Medical Faculty, University of Nis, Nis, Serbia

###### **Correspondence:** M. Jakovljevic

**Introduction:** Children mostly have mild or asymptomatic forms of SARS-CoV-2 infection, but during pandemic a higher incidence of Kawasaki disease, Kawasaki-like syndrome and the emergence of a new clinical entity, multisystem inflammatory post-covid syndrome (MIS-C) has also been observed.

**Objectives:** The aim of this study is to determine clinical features and laboratory findings in patients with MIS-C.

**Methods:** Retrospective analysis of clinical features and laboratory findings of MIS-C patients treated at our tertiary referal center (Clinic of Pediatric, University Clinical Centre Nis, Serbia).

**Results:** From 18^th^ of March 2020 till 30^st^ of April 2021 there were 10 patients diagnosed as MIS-C according to CDC criteria. Eight patients were male and two were female. Patients age was 2 to 13 years (average 7.9 years, median 7 years). All patients had SARS -CoV-2 N-protein IgG antibodies but without history of disease symptoms and had positive contact four weeks prior to the onset of MIS-C symptoms. First symptom of MIS-C was fever (over 38C) which lasted in average for 4.4 days (3-7 days). Muco-cutaneous and gastrointestinal manifestations were most common. All patients had bulbar conjuctivitis, rash was present in 8 patients (80%), hand/foot oedema in 6 cases (60%), anterior cervical lymphadenopathy and cheliitis in 4 cases (40%) and periobital oedema in one case (details presented in Table 1. Clinical features of MIS-C patients). Nine patients (90%) presented with gastrointestinal symptoms while nervous system was affected in 5 patients. Three patients developed heart insuffitiency and one patient developed early signs of right coronary arthery aneurism. All patients had elevated inflammatory markers. Complete blood count showed elevated levels of white blood cells in 9 patients. Hypoalbuminemia and hypoproteinemia, low levels of serum potassium and sodium were present during ten days after the onset of symptoms. Troponines were elevated in 4 cases, proBNP in 5 cases. Abdominal ultrasound was performed and 6 patients presented with hepatoplenomegaly, 3 with enlarged spleen, one with enlagred liver and 4 had ascites. All patients were treated with combination of two antibiotics till cultures were proven negative, corticosteroid therapy and antiaggregation therapy. Three patients received a IVIG in a single dose (2gr/kg). All patients had good response to corticosteroid therapy (2mg/kg). Corticosteroid therapy was continued for four weeks (tapering).

**Conclusion:** MIS-C can be a life-threatening condition in children. Early diagnosis and timely adequate treatment are of paramount importance. In children less than 5 years of age, the distinction between Kawasaki (Kawasaki shock) syndrome and MIS-C might be difficult, influencing the decision to use IVIG or steroids alone.

**Patient Consent Received**

No

**Disclosure of Interest**

None declared

**Table 1 (abstract P367).** See text for description

2,5 y/o male	Rash Conjuctivis Cheiliitis Hand/foot oedema	Opstipation	Hypotension	No
5 y/o female	Rash Conjuctivis Hand/foot oedema	Vomiting	Hypotension Left ventricular dysfunction	No
6 y/o male	Rash Conjuctivis Periorital oedema	Opstipation	Hypotension	Headache Meningeal irritation
7 y/o female	Conjuctivis Cheiliitis	Abdominal pain	No	Photophobia
9 y/o male	Rash Conjuctivis	Intestinal suboclussion	No	No
12y/o male	Rash Conjuctivis Hand/foot oedema	Vomitation	Tachycardia	Meningeal irritation
12,5 y/o male	Rash Conjuctivis Cheliitis Hand/foot oedema and desqumation	Abdominal pain	Hypotension Myocardial dysfunction	Photophobia
12,5 y/o male	Rash Cheliitis Conjuctivis Hand/foot oedema	Vomitation	Hypotension Myocardial dysfunction, tricuspidal and mitral regurgitation	No
13y/o male	Rash Conjuctivis Hand/foot oedema and desqumation	Diarrhea	Coronary aneurism	Headache Photophobia Meningeal irritation
13y/o male	Conjuctivis	Abdominal pain	No	No

### P368 Impact of the COVID-19 pandemic on the frequency of the pediatric rheumatic diseases

#### U. Kaya Akca^1^, E. Atalay^1^, M. Kasap Cuceoglu^1^, Z. Balik^1^, S. Sener^1^, Y. Ozsurekci^2^, O. Basaran^1^, E. D. Batu^1^, Y. Bilginer^1^, S. Ozen^1^

##### ^1^Department of Pediatrics, Division of Pediatric Rheumatology; ^2^Department of Pediatric Infectious Diseases, Hacettepe University Faculty of Medicine, Ankara, Turkey

###### **Correspondence:** U. Kaya Akca

**Introduction:** Coronavirus disease (COVID-19) has brought many changes in our daily lives such as wearing masks, social distancing, and curfews. These implemented measures and restrictions have probably prevented some other infections. The impact of the COVID-19 pandemic, and implemented measures/restrictions on the frequency of the pediatric rheumatic diseases remain unknown.

**Objectives:** We aim to investigate the effect of COVID-19 on the frequency of the pediatric rheumatic diseases in our practice.

**Methods:** We retrospectively reviewed the medical records of patients admitted to the pediatric rheumatology unit between February 2016 and March 2021. Patients were divided into five groups according to the year of diagnosis (February 2016 to February 2017; March 2017 to February 2018; March 2018 to February 2019; March 2019 to February 2020; and March 2020 to March 2021). The distribution of the patients who were newly diagnosed with a rheumatic disease in the pre-COVID-19 period (February 2016-March 2020) and during the COVID-19 pandemic (March 2020-March 2021) was compared.

**Results:** A total of 32,333 patients visited the pediatric rheumatology department between 2016 and 2021. The mean annual number of patients decreased by 42% during the COVID-19 pandemic (7060 patients/year vs. 4090 patients/ year). 25,156 out of 32,333 admissions (77.8%) were recurrent visits. Among the 7177 patients who remained after the exclusion of repeated visits, 2728 patients got 2813 new diagnoses of rheumatic diseases. In the pre-pandemic period, the most frequently diagnosed rheumatic disease was familial Mediterranean fever (FMF) (n=695, 28.3%), whereas multisystem inflammatory syndrome in children (MIS-C) (n=68, 18.6%) was the most common diagnosis in the pandemic period. There were significant differences in the numbers of newly diagnosed cases with FMF, Behçet's disease, and IgA vasculitis (IgAV), chronic non-bacterial osteomyelitis (CNO), cutaneous vasculitis, primary central nervous system (CNS) vasculitis, and idiopathic orbital myositis. When we compared the frequencies during the 2019-2020 period with those in the pandemic period (2020-2021), there were significant decreases in the numbers of newly diagnosed patients with FMF (p=0.043), IgAV (p=0.001), primary CNS vasculitis (p=0.035), and idiopathic uveitis (p=0.015); while the number of newly diagnosed cases with CNO increased (p<0.001). Also, the numbers of newly diagnosed patients with juvenile idiopathic arthritis, PFAPA (periodic fever, aphthous stomatitis, pharyngitis, and cervical adenitis) syndrome, acute rheumatic fever, and Kawasaki disease decreased remarkably in the pandemic period although these differences were not statistically significant.

**Conclusion:** This study highlighted the decreased prevalence of some rheumatic diseases in the COVID-19 period. The potential decrease in infectious diseases due to pandemic restrictions could be affecting the diagnostic rates in pediatric rheumatology since infection is a trigger for some rheumatic diseases such as IgAV. Another reason could be the decrease in clinical visits of patients during the pandemic.

**Patient Consent Received**

No

**Disclosure of Interest**

None declared

**Table 1 (abstract P368).** Distributions of patients newly diagnosed with rheumatic diseases according to the years

	2016-2017 (n)	2017-2018 (n)	2018-2019 (n)	2019-2020 (n)	2020-2021 (n)	P value
MAS	7	3	4	3	0	0.406
FMF	228	165	159	143	55	**<0.001**
PFAPA syndrome	67	72	69	84	45	0.127
MIS-C	0	0	0	0	68	**<0.001**
Behçet’s disease	6	13	7	20	10	**0.008**
IgA vasculitis	97	84	102	97	26	**0.005**
Kawasaki disease	19	18	14	16	2	0.235
CNO	6	6	6	4	13	**<0.001**
Acute rheumatic fever	28	25	14	18	6	0.157

### P369 (Auto)immune manifestations in COVID-19

#### B. Koren, V. Berce

##### Pediatric Clinic, University Medical Centre, Maribor, Slovenia

###### **Correspondence:** B. Koren

**Introduction:** It is well established that many types of infections can lead to autoimmunity or autoimmune disease. Since the emergence of coronavirus disease 2019 (COVID-19), a number of confirmed cases reported autoimmune manifestations.

**Objectives:** To evaluate occurence of (auto)immune manifestations in pediatric patients with evidence of recent infection with severe acute respiratory syndrome (SARS) associated coronavirus 2 (SARS-CoV-2).

**Methods:** A single small center study was performed. Included were patients with (auto)immune manifestations and evidence of concomitant or recent SARS-CoV-2 infection seen at our pediatric clinic from December 2020 till April 2021. Infections with other microorganisms were excluded. Data was collected from patients' medical records.

**Results:** A total of 12 patients were enrolled. The results are presented in Table 1.

The mean age of all patients was 9.5 years (range 4-18 years). Interestingly, there was a slight male predominance (7 patients, 58%). Among immune diseases related to COVID-19, MIS-C/PIMS-TS was the most common (7 patients, 58%). It has been suggested that the syndrome results from an abnormal immune response to the virus and it is not considered an autoimmune disease. 2 boys with MIS-C/PIMS-TS, aged 4 and 5 years, also fulfilled diagnostic criteria for Kawasaki disease. Autoantibodies were detected in 2 patients, in a 15 years old boy with Miller Fisher syndrome (anti GQ1b antiganglioside antibodies) and in a 13 years old girl with Henoch- Schonlein purpura (antinuclear antibodies, ANA).

Most of our patients had positive COVID-19 serology (10 patients, 83.3%), negative PCR swab for COVID-19 (9 patients, 75%) and had a family history of COVID-19 (9 patients, 75%).

**Conclusion:** So far, some patients have been reported to develop (auto)immune diseases after COVID-19. It is speculated that SARS-CoV-2 can disturb self- tolerance and trigger autoimmune responses through cross- reactivity with host cells. Overall, more data are needed to further understand the relationship between COVID-19 and autoimmunity.

**References**

1. Liu Y et al. COVID-19 and autoimmune diseases. Curr Opin Rheumatol 2021, 33:155-62.

2. Saad MA et al. COVID-19 and autoimmune diseases. A systematic review of reported cases. Current Rheumatology Reviews 2021; 17:193.

3. Ehrenfeld M et al. COVID-19 and autoimmunity. Autoimmun Rev 2020; 19(8):102597.

**Disclosure of Interest**

None declared

**Table 1 (abstract P369).** See text for description

Disease	Age (years, mean, range)	Male (n,%)	COVID-19 symptoms (n,%)	COVID-19 serology (IgG positive, n,%)	COVID-19 PCR (positive, n,%)	Family history of COVID-19 (n,%)
MIS-C/ PIMS-TS (n=7)	8.4 (4-15)	3 (42.8%)	3 (42.8%)	5 (71.4%)	3 (42.8%)	4 (57.1%)
Henoch- Schonlein purpura (n=2)	10 (7-13)	1 (50%)	0	2 (100%)	0	2 (100%)
Miller Fisher syndrome (n=1)	15	1 (100%)	1 (100%)	1 (100%)	0	1 (100%)
Erythema nodosum (n=1)	3	1 (100%)	0	1 (100%)	0	1 (100%)
Covid toes (n=1)	18	1 (100%)	1 (100%)	1 (100%)	0	1 (100%)

MIS-C (multisystem inflammatory syndrome in children); PIMS-TS (pediatric inflammatory multisystem syndrome temporally associated with SARS-CoV-2 infection)

### P370 The magnitude of COVID-19 in children with rheumatic/autoinflammatory diseases on immunomodulatory agents: a single center experience

#### P. Krepis^1^, F. Dasoula^1^, C. Papadaki^1^, A. Syggelou^2^, D. N. Maritsi^2^

##### ^1^Paidwn Aglaia Kyriakou, Athens, Greece; ^2^Pediatrics, Paidwn Aglaia Kyriakou, Athens, Greece

###### **Correspondence:** P. Krepis

**Introduction:** Children appear less susceptible to SARS-CoV2 infection comparing to adults; however, the burden of the disease remains unclear particularly among specific groups, including immunocompromised children.

**Objectives:** To assess the impact of COVID-19 in children with chronic rheumatic/autoinflammatory diseases (AIRD) being treated with disease-modifying antirheumatic drugs (DMARDs).

**Methods:** We conducted a telephone survey between May 2020 and May 2021 interviewing the parents of all children with AIRD that attend the Immunology and 2Rheumatology Unit of our center. A hospital physician inquired about AIRD characteristics, current DMARDs use and duration, COVID-19 main symptoms, if any, source of transmission and duration.

**Results:** Of all 453 patients with AIRD who were currently on DMARDs, 20 children (4.4%) had tested positive for SARS-CoV2 by polymerase chain reaction of nasopharyngeal specimen, during the study period. Male/Female ratio of patients was 0.8 and the median age was 13 years old (interquartile range 5.3-15 years). The underlying AIRD was polyarticular juvenile idiopathic arthritis (35%), followed by oligoarticular idiopathic arthritis (25%). In all cases, primary AIRD was in remission at the time of SARS-CoV2 acquisition. Comorbidities were recorded in three patients (15%), including one adolescent with chronic stable course of asthma, one case of Hashimoto thyroiditis and one child with atopic dermatitis. Six out of all patients (30%) were symptomatic, with a mild course of COVID-19. Predominant symptoms were malaise (67%), cough (67%) and fever (50%); sore throat, muscle aches and abdominal aches were also reported in 33% of cases, respectively. No hospitalizations nor flares of the underlying AIRD were recorded. No in-school transmission was documented. All patients successfully recovered after a median of three days, without experiencing any post-COVID-19 conditions, and were followed until having three serial negative molecular tests. Biologic agents were not administered during COVID-19 course, according to The American College of Rheumatology guidance. Tocilizumab and adalimumab were the most prevalent biologic DMARDs (35%) followed by etanercept (15%). None of the patients were on corticosteroids, while seven (35%) were receiving conventional DMARDs concomitantly, mainly methotrexate (86%). The six COVID-19 symptomatic children were receiving adalimumab (3/6), tocilizumab (1/6), canacimumab (1/6) and belimumab (1/6).

**Conclusion:** In our small cohort of children with AIRD and DMARDs, SARS-CoV2 infection was relatively mild in all symptomatic cases without triggering any relapse of the primary AIRD. Our results may suggest a potential protective role of DMARDs in the evolution of COVID-19 among children with AIRD, particularly when the AIRD is in remission and there are no significant comorbidities.

**Patient Consent Received**

Yes

**Disclosure of Interest**

None declared

### P371 Efficacy and safety of anakinra in MIS-C: a retrospective multicenter study

#### F. Licciardi^1^, C. Covizzi^1^, M. Dellepiane^1^, N. Olivini^2^, M. Mastrolia^3^, A. LoVecchio^4^, V. Monno^5^, M. Tardi^6^, A. Mauro^6^, M. Alessio^4^, G. Filocamo^7^, M. Cattalini^8^, A. Taddio^9^, R. Caorsi^10^, G. Marseglia^11^, F. LaTorre^5^, A. Campana^2^, G. Simonini^3^, A. Ravelli^10^, D. Montin^1^, on behalf of Gruppo di studio Reumatologia, SIP

##### ^1^Ospedale Infantile Regina Margherita, Turin; ^2^Bambino Gesù Children's Hospital, Rome; ^3^Anna Meyer Children's Hospital, Florence; ^4^Policlinico Federico II, Naples; ^5^Giovanni XXIII Pediatric Hospital, Bari; ^6^Santobono-Pausilipon Children's Hospital, Naples; ^7^Cà Granda Ospedale Maggiore Policlinico, Milano; ^8^Spedali Civili, Brescia; ^9^Burlo Garofolo, Trieste; ^10^Istituto Giannina Gaslini, Genoa; ^11^Policlinico S. Matteo, Pavia, Italy

###### **Correspondence:** F. Licciardi

**Introduction:** Multisystem inflammatory syndrome in children (MIS-C) is a severe and recently described disease affecting pediatric patients, triggered by SARS-CoV2. Current treatment is based upon IVIG and steroids but some patients are resistant to first line therapy. In these patients some authors have used IL1 receptor antagonist (Anakinra) with benefit, but data regarding efficacy, dose and route of administration are lacking.

**Objectives:** To analyze the outcomes of MIS-C patients treated with anakinra (ANK) in Italy since 1/4/20.

**Methods:** We performed an anonymous retrospective multicenter study of patients diagnosed with MIS-C, according to the preliminary WHO case definition, treated with ANK from 1/4/20 to 28/2/21. SARS-CoV2 infection was demonstrated either by serology or by positive molecular swab (RT-PCR) in the six weeks prior to admission. After the start of ANK we measured the following outcomes: rate of patients needing further therapeutic step-up, rate of patients achieving clinical (fever defervescence in 24 hours) and laboratory response (CRP halving in 48 hours), rate of Coronary Artery Anomalies (CAA) development during follow-up.

**Results:** In the study period 35 MIS-C patients were treated with ANK: 13 patients (37.1%) in Intensive Care Unit (ICU, Group A) and 22 (62.9%) in non-ICU settings (Group B). Epidemiological, clinical, and laboratoristic features at ANK prescription are described below:

**Table:** 

	Group A	Group B
Age at diagnosis	12y (8-13)	8.5y (4-12)
WBC (/mm3)	10780 (5020-14280)	10935 (6155-18500)
Lymphocytes (/mm3)	960 (660-980)	1710 (760-1970)
Platelets (/mm3)	185 (107-189)	152 (89-187)
CRP (mg/L)	293 (212-305)	86 (17-153)
Ferritin (ng/mL)	744 (334-1133)	574 (512-748)
Hypotension Hypotension needing inotropic support	69.2% 69.2%	36.4% 0%
NT-proBNP (ng/L)	16549 (3247-20163)	6043 (1405-10502)
CAA before ANK start	0%	9.1%

In Group A the most common indication for ANK was cardiac function worsening (46.1%), while in Group B ANK was started mostly for persistent elevation in inflammatory markers (ferritin, CRP) unresponsive to IVIG and/or steroids (31.8%). Endovenous (ev) ANK was used in all Group A patients (mean dose 8mg/Kg), while most patients in Group B (72.7%) received subcutaneous (sc) ANK (mean dose 4mg/Kg).

Overall only 2 patients (5.7%) needed a step-up treatment after ANK start (1 required IVIG, 1 methylprednisolone dose increase), most of the patients achieved clinical (85.7%) and laboratory response (74.3%). 2 patients had CAA before ANK, none developed CAA after starting ANK. Overall NT-proBNP halved in 2.5 days in Group A and 2 days in Group B, while Ejection Fraction (EF) normalized respectively in 2 and 3 days.

None of the patients in Group B needed ICU admission or inotropic support after ANK.

The most frequently observed side effect was ALT increase (30.8% in Group A and 9.1% in Group B), only 1 patient had injection site reaction.

**Conclusion:** MIS-C is a severe emerging disease with a high ICU admission rate. Our retrospective data suggest that both ev and sc ANK is effective in controlling inflammation, fever and cardiac dysfunction. Side effects are transient and usually mild. Overall the reported incidence of CAA in MIS-C cohorts is 10%, interestingly in our cohort no patient has developed CAA after beginning ANK, possibly suggesting a protective role of IL1 inhibition in aneurysm formation. Further studies in bigger cohorts are needed to define the most effective timing and dose of ANK in MIS-C.

**Disclosure of Interest**

F. Licciardi: None declared, C. Covizzi: None declared, M. Dellepiane: None declared, N. Olivini: None declared, M. Mastrolia Consultant for: SOBI, A. LoVecchio: None declared, V. Monno: None declared, M. Tardi: None declared, A. Mauro: None declared, M. Alessio: None declared, G. Filocamo Consultant for: SOBI, M. Cattalini: None declared, A. Taddio: None declared, R. Caorsi: None declared, G. Marseglia: None declared, F. LaTorre: None declared, A. Campana: None declared, G. Simonini Consultant for: SOBI, A. Ravelli: None declared, D. Montin: None declared

### P372 COVID-19 temporally related multisystem inflammatory syndrome (MIS-C): an early window of opportunity is a good treatment strategy? The experience of the paediatric covid center in Palermo

#### S. Giordano^1^, M. C. Failla^2^, M. G. Campione^3^, L. Siracusa^2^, L. Messina^2^, L. Alessi^2^, M. C. Maggio^4^

##### ^1^U.O.C. of Paediatric Infectious Diseases, Paediatric COVID Center, Children Hospital “G. Di Cristina”, ARNAS, Palermo, Via dei Benedettini 1, 90100 Palermo; ^2^U.O.C. of Paediatric Infectious Diseases, Paediatric COVID Center, Children Hospital “G. Di Cristina”, ARNAS, Palermo, Via dei Benedettini 1, 90100 Palermo; ^3^University Department PROMISE “G. D’Alessandro”, University of Palermo, Via del Vespro 129, 90100 Palermo; ^4^University Department PROMISE "G. D'Alessandro" - Children Hospital "G. Di Cristina", University of Palermo, via dei benedettini n. 1, Palermo, Italy

###### **Correspondence:** M. C. Maggio

**Introduction:** Multi-system inflammatory syndrome in children (MIS-C) shows a presentation mimicking Kawasaki Disease (KD), Toxic Shock Syndrome (TSS), Macrophage Activation Syndrome (MAS). Furthermore, many children show respiratory or abdominal symptoms.

**Objectives:** Intravenous immunoglobulin (IVIG) is recommended as first line treatment as in KD, followed by aspirin, steroids and, in IVIG-resistant patients, IL-1 or IL-6 blocking agents.

**Methods:** We describe a cohort of 16 Sicilian children (6M;10F; age:1.4-14 years), with MIS-C, with clinical features compatible with classical or incomplete KD, in some cases with MAS and/or TSS. Demographic, clinical, laboratory, echocardiographic and imaging findings, treatment strategy and outcome were collected.

**Results:** Common presenting symptoms included: fever (94%), abdominal pain or vomiting (50%), mucocutaneous rash (50%), conjunctivitis (44%), latero-cervical lymphadenitis (63%), cheilitis/pharyngeal hyperaemia (81%), hands and feet oedema (13%). Symptoms started 1-8 days before the hospitalization. Nasopharyngeal swab for SARS-CoV-19 was positive in 12/16 patients, with positive serological IgG, negative or grey zone IgM-type antibodies. 2 patients with negative swab had a history of recent infection and positive IgG-type antibodies; 2 patients had parents with positive swab.

All the patients showed significant increase of C-reactive protein (CRP). AST, ALT, gamma-GT were increased in 25%. Pancreatic amylase and lipase were increased in 13%, 19% showed lymphocytopenia.

Pro-BNP was increased (129-3980pg/ml) in 44% and troponin was increased (27.3-246ng/ml) in 31%. In addition, hyponatraemia was found in 100% of cases. Furthermore, 31 % had proteinuria. 50% showed cardiac involvement (3 pericardial effusion; 5 mitral insufficiency; 2 mitral and aortic insufficiency; 1 coronaritis). Pleural, ascitic, pericardial effusion and abdominal adenitis were found in 19%, 25%, 19% and 31% of cases, respectively.

IL-6 levels were evaluated in 9/16 patients and 8/9 showed a significant increase (30.2-285pg/ml) with a rapid normalization after steroids and IVIG treatment. Pro-BNP persisted increased for 7-10 days after IVG and steroids treatment. 25% of patients dramatically and rapidly evolved in a MAS-like form, fulfilling the classification criteria for the diagnosis of MAS (ACR/EULAR 2016). High doses of steroids and IVIG were promptly started with a significant improvement of the clinical course. In all the patients, treatment was started within 72 hours of admission, with IVIG (2 g/Kg/dose), methylprednisolone (2mg/Kg/day in 56% of patients; 30 mg/Kg/day for 3 days, followed by 2 mg/Kg/day in 38% of patients). 2 patients were treated with enoxaparin. TSS was described in 2 patients, who received additionally vasoactive drugs, albumin and diuretics.

**Conclusion:** In our series, most of patients received a prompt treatment with IVIG and steroids. This approach could explain the good outcome in all the cases and the rapid restoring of cardiac function also in patients with MAS or TSS. Patients showed a wide spectrum of presenting signs and symptoms; evidence of inflammation with pathological values of CRP, ESR, D-dimer, ferritin, pro-BNP, troponin, transaminase, pancreatic amylase and albumin; a multi-organ involvement was documented in a high percentage of cases, inducing the clinician to perform a multi-specialistic approach.

**Patient Consent Received**

Yes

**Disclosure of Interest**

None declared

### P373 COVID-19 temporally related multisystem inflammatory syndrome (MIS-C) and cardiovascular involvement assessed with cardiac magnetic resonance (CMR). Experience of the children hospital of Palermo

#### F. Finazzo^1^, A. Alaimo^2^, S. Giordano^3^, M. C. Failla^3^, V. Vanella^3^, L. A. Canduscio^3^, A. Lembo^4^, M. C. Maggio^4^

##### ^1^UOC of Paediatric Radiology, Children Hospital “G. Di Cristina”; ^2^U.O.C. of Paediatric Cardiologic Division, Children Hospital “G. Di Cristina”; ^3^U.O.C. of Paediatric Infectious Diseases, Paediatric COVID Center, Children Hospital “G. Di Cristina”, ARNAS; ^4^University Department PROMISE “G. D’Alessandro” – Children Hospital “G. Di Cristina”, ARNAS Palermo, University of Palermo, Palermo, Italy

###### **Correspondence:** M. C. Maggio

**Introduction:** MIS-C is a hyperinflammatory syndrome that follows exposure to SARS-CoV-2 by 2-6 weeks. However, some aspects remain unclear, such as cardiac involvement.

**Objectives:** to evaluate the role and effectiveness of cardiac magnetic resonance (CMR) in heart involvement in children affected by MIS-C; to review the expert groups' clinical experience in the field.

**Methods:** we describe a case series of 7 children (age: 2-11 years), admitted to the tertiary care Children Hospital “G. Di Cristina”, Palermo, between December 2020 and May 2021 with clinical symptoms meeting the criteria for the diagnosis of MISC-C. All the patients showed findings of cardiac involvement without coronary artery lesions. Transthoracic echocardiography demonstrated temporary systolic dysfunction that lasted for 2-5 days. CMR was performed during the recovery phase or after the discharge (the median time to CMR was 10-30 days after the onset of illness). CMR was performed with a 1,5 Tesla scanner (GE Signa Explorer). 5/7 didn't undergo CMR study during the acute phase because they were clinically unstable and needed general anesthesia or sedation.

The protocol included, before intravenous contrast media injection, retrospective ECG-Gated fiesta cine sequences (short axis, 4, 3 and 2 chamber views), sequences for edema, and hyperemia T2 -short tau inversion recovery (Stir) (repetition time =1689ms, echo time55.10 ms).

Myocardial edema was evaluated by following the Lake Louise criteria. Because normal value in native T1 mapping and T2 relaxation time in children have poor reference, myocardial edema was characterized by increased signal intensity on T2-weighted imaging and myocardial damage by non-ischemic patterns late gadolinium enhancement.

Study for evaluating myocyte necrosis and fibrosis: Late gadolinium-enhanced 2D inversion recovery sequences performed at 6 min following intravenous contrast medium administration (0,2 mmol/kg).

**Results:** In 5/7 patients, T2-Stir sequences didn't show myocardial edema and hyperemia. Mean indexed left ventricular end-diastolic volume (iLVEDV), indexed left ventricular end-systolic volume (iLVESV), and indexed left ventricular stroke volume (iLVSV) were within normal range corrected for BSA. In 2 patients CMR showed late gadolinium enhancement in non-ischemic pattern. 1 patient, studied in subacute phase, after steroids and IVIG treatment, showed ventricular apical septum and lateral wall myocardial oedema, without fibrosis and an imaging compatible with focal acute myocarditis. Ventricular systolic function was normal. 1 patient, studied 1 month after the acute phase, and showed myocardial fibrosis.

**Conclusion:** international literature reports that children with MIS-C develop a transitory myocardial impairment, resembling myocarditis, with full recovery in most of them. Until now, the pathophysiology of the event is still object of debate.

CMR is an excellent noninvasive diagnostic tool for the diagnosis and follow-up of myocarditis. Furthermore, CMR can predict prognosis and recognize children at high risk to develop arrhythmias and unfavorable events. CMR is a codified method highlighting specific features of myocardial damage: inflammation, edema, necrosis, contractile scar impairment, and pericardial effusion. 6/7 didn't demonstrate myocardial oedema, probably because the CMR was performed during the recovery.

**Disclosure of Interest**

None declared

### P374 Livedo reticularis as a late sign of COVID-19 temporally related multisystem inflammatory syndrome (MIS-C): a case series

#### S. Giordano^1^, M. C. Failla^1^, V. Vanella^1^, L. A. Canduscio^1^, M. Scalisi^2^, M. C. Maggio^3,4^

##### ^1^U.O.C. of Paediatric Infectious Diseases, Paediatric COVID Center, Children Hospital “G. Di Cristina”; ^2^U.O.C. of Paediatric Infectious Diseases, Paediatric COVID Center, Children Hospital “G. Di Cristina”, ARNAS, ARNAS; ^3^University Department PROMISE “G. D’Alessandro”, Children Hospital "G. Di Cristina", ARNAS; ^4^University Department PROMISE “G. D’Alessandro” – Children Hospital “G. Di Cristina”, ARNAS Palermo, University of Palermo, Palermo, Italy

###### **Correspondence:** M. C. Maggio

**Introduction:** In children, the dermatologic features appear to occur early with other COVID-19 manifestations. Dermatologists play a key role in the early diagnosis of COVID-19. Multi-system inflammatory syndrome in children (MIS-C) shows a presentation mimicking Kawasaki Disease (KD), with mucocutaneous signs. However, late onset dermatological signs are poorly described.

**Objectives:** to evaluate children with MIS-C during the follow-up and to describe late dermatological signs in these patients.

**Methods:** We followed 14 children (3M; 11 F) with MIS-C, with clinical, biochemical, imaging data. Autoantibodies, D-Dimer, CRP, ESR, C3, C4, ferritin, serum amyloid, IgA, IgM, IgG were detected 1-2 months after the resolution of the clinical manifestations of MIS-C.

**Results:** 8/14 children (58%) showed livedo reticularis at the legs, arms, trunk. The livedo was more evident at the legs in all the patients. The livedo started at the remission, after normalization of CRP, ESR, D-Dimer; the sign lasted also for 1-2 months after the discontinuation of steroids and the normalization of haematochemical parameters. 4/8 showed low-title positive autoimmune tests (ANA in 2; ENA anti-Sm in 2; anti-cardiolipin IgG in 1; ASCA in 2).

**Conclusion:** In our series, 8/14 patients showed a livedo reticularis, more marked in the legs, however in some cases with a wide distribution to arms and the trunk. Low-title autoantibodies were transiently positive in 50% of these cases, negative in later detections.

Livedo reticularis was a late sign, linked to MIS-C related vasculitis, persisting 1-2 months after the resolution of MIS-C. A different treatment regimen (IVIG plus steroids at 1-2 or 30 mg/Kg/day) did not influence the progress of this clinical manifestation. In 50% of children we documented a transient autoimmune response.

**Patient Consent Received**

Yes

**Disclosure of Interest**

None declared

### P375 Acute cardiovascular manifestations in children with multisystem inflammatory syndrome associated with COVID-19 infection in a Sicilian case series

#### A. Alaimo^1^, S. Giordano^2^, M. C. Failla^3^, F. Finazzo^4^, L. Siracusa^3^, L. Messina^3^, L. Alessi^3^, M. Scalisi^3^, M. C. Maggio^5^

##### ^1^U.O.C. of Paediatric Cardiologic Division, Children Hospital “G. Di Cristina”, ARNAS; ^2^U.O.C. of Paediatric Infectious Diseases, Paediatric COVID Center, Children Hospital “G. Di Cristina”, ARNAS; ^3^U.O.C. of Paediatric Infectious Diseases, Paediatric COVID Center, Children Hospital “G. Di Cristina”; ^4^UOC of Paediatric Radiology, Children Hospital “G. Di Cristina”, ARNAS; ^5^University department PROMISE “G. D’Alessandro” - Children Hospital “G. Di Cristina”, ARNAS, Palermo, University of Palermo, Palermo, Italy

###### **Correspondence:** M. C. Maggio

**Introduction:** Multisystem inflammatory syndrome in children (MIS-C) is a severe complication of COVID-19 infection, typically evidenced 4-6 weeks after the infection. The debated pathogenesis is a dysregulation of inflammatory response to SARS-CoV-2 infection ad a cytokine hyperexpression. Persistent fever, respiratory and gastrointestinal symptoms are the most common manifestations, associated with typical clinical signs described in Kawasaki Disease (KD). Furthermore, pleiomorphic cardiac manifestations are described, including ventricular dysfunction, coronary artery dilation and aneurysms, arrhythmia, conduction abnormalities and pericardial effusion. These manifestations are a strong link with KD, even if in MIS-C they are more frequently documented. Severe cases can present as Toxyc Shock Syndrome (TSS) with vasodilatory or cardiogenic shock, requiring treatment with plasma expanders, inotropic drugs, diuretics, albumin and -in the more severe patients- extracorporeal membrane oxygenation and mechanical ventilation. KD experience guided the clinicians to treat these children with intravenous immunoglobulin (IVIG), steroids, aspirin (ASA) and, in refractory cases, anti-IL-1 monoclonal antibodies.

**Objectives:** Most patients recover within days to a couple of weeks and mortality is rare, although the medium- and long-term sequelae, particularly cardiovascular complications, are not yet known.

**Methods:** We describe the short-term outcome in a case series of 12 Sicilian children (4M; 8F; age: 1.4-14 years) with MIS-C and a documented recent or actual infection by SARS-CoV-2 who showed cardiac involvement.

**Results:** The cardiac features were: 3 patients showed pericardial effusion; 1 coronaritis; 6 transient mitral valve regurgitation; 1 Brugada pattern, evidenced when he was febrile; 2 showed associated mitral and aortic valve regurgitation). 7/8 patients with valve regurgitation showed a significant increase of pro-BNP, normalized during the follow-up.

TSS was described in 2 patients, showing a significant increase of troponin, promptly treated with high dose of methylprednisolone, IVIG, vasoactive drugs, albumin and diuretics.

3 patients (21%), after the resolution of the acute phase, showed bradycardia (heart rate < 50/min), persisting for 7-10 days. The bradycardia was not associated with first-degree AVB, or a pathological PR. 6 patients (42%) showed an altered ventricular repolarization phase, in association with an increase of pro-BNP (129-3980 pg/ml). 4/12 (33%) had increased troponin levels (27.3-246 ng/ml) in the acute phase, with the normalization of troponin after IVIG and steroids treatment. Pro-BNP persisted increased for a longer time, besides the clinical improvement and the normalization of blood chemistry parameters.

**Conclusion:** Generally, pro-BNP and troponin levels in MIS-C are higher than in KD, reflecting vasculopathy and cardiomyocytes damage extent. Persistence of increased levels of pro-BNP, in patients with a normalization of inflammatory parameters, suggests a mechanism of myocardial oedema, persisting besides the intensive care approach useful, however, to limit effects on cardiac function and normalize inflammatory parameters. Patients admitted with MIS-C require close electrocardiogram monitoring during the acute phase and the recovery, even if they do not manifest dyselectroliteemia, coronary lesions, pericardial effusion, myocarditis, shock. This approach can avoid severe arrythmia.

**Patient Consent Received**

Yes

**Disclosure of Interest**

None declared

### P376 Step-by-step surveillance in children with multisystem inflammatory syndrome associated with COVID-19 infection: proposal of a cardiologic follow-up

#### F. Finazzo^1^, A. Alaimo^2^, S. Giordano^3^, M. C. Failla^3^, M. C. Maggio^4^

##### ^1^UOC of Paediatric Radiology, Children Hospital “G. Di Cristina”; ^2^U.O.C. of Paediatric Cardiologic Division, Children Hospital “G. Di Cristina”; ^3^U.O.C. of Paediatric Infectious Diseases, Paediatric COVID Center, Children Hospital “G. Di Cristina”, ARNAS; ^4^University Department PROMISE “G. D’Alessandro” – Children Hospital “G. Di Cristina”, ARNAS Palermo, University of Palermo, Palermo, Italy

###### **Correspondence:** M. C. Maggio

**Introduction:** Multisystem Inflammatory Syndrome Associated with COVID-19 Infection (MIS-C) shows many matches with children with Kawasaki disease (KD) and most children with MIS-C have incomplete or complete KD-like phenotype. In these patients cardiologic involvement mimics KD, showing, however, a higher incidence of severe acute manifestations.

**Objectives:** In children with MIS-C and clinical findings related to heart disease, ECG and echocardiography are the first-line imaging. Until now, there are no international guidelines on the management of these patients. We suggest to take inspiration from the recommendations for KD for the significant phenotypic overlap between MIS-C and KD.

**Methods:** We propose a step-by-step cardiologic imaging follow-up:

- in all the patients, we recommend ECG and echocardiography at the diagnosis, at the worsening of the clinical and/or blood chemist parameters (CRP, ESR, ferritin; proBNP, troponin, D-Dimer), at any change of treatment supported by clinical worsening, at 8, 30, 45, 60, 90, 180 days since the diagnosis. The time-table may be changed in consideration of the outcome of the patient.

-In patients with coronary artery dilatation (CAL), documented by echocardiography, it is advisable to follow-up them, since the diagnosis, with ECG, echocardiography, D-dimer, pro-BNP, troponin. -If CAL are oversized with z-score >- 2,5, according to age and body surface or increase during the follow-up:

-it is recommended to perform Coronary Computed Tomography (CT) (CCA) or Cardiac Magnetic Resonance Angiography (CMRA). In fact, echocardiography cannot visualize the whole coronary artery vessels.

**Results:** Both allow visualization of coronary artery aneurysms, vessels thickening, myocardial perfusion defects, permitting risk stratification and handing treatment decisions.

CMRA is the first choice, because it is a radiation-free imaging method. It can evaluate the entire coronary artery system and provides details on myocardial function ischemia (detecting areas of inducible myocardial ischemia with pharmacological stress), infarction, inflammation, fibrosis.

However, the new generation Multidetector Single –Source CT scanners and Dual Source Ct scanner allow a fast heart CT study with low radiation dose and reduce the need for sedation.

CMR is less suitable because it is a lengthy examination and very often requires general anesthesia.

If echocardiography demonstrates myocardial dysfunction or valve regurgitation at admission or during hospitalization, we suggest performing CMR. There is no consensus on the right timing.

**Conclusion:** We suggest performing CMR during the acute or subacute phase: it is a step of the relieve in the cardiologic diagnosis to assess ventricular function and myocardial active injuries (oedema, hyperemia, ischemia, necrosis) and to repeat the imaging at the discharge in patients with the first pathological CMR, to evaluate fibrosis by myocardial delayed enhancement. CMR at the discharge is suggested also in cases with the first CMR normal, who showed a worsening of the echocardiographic parameters, the relieve of new-insurance valvulitis, persistent arrhythmia. At 3-6 months since the patient showed the remission, the CMR must be repeated to avoid any fibrotic lesions.

**Disclosure of Interest**

None declared

### P377 Safety and tolerability of the biontech COVID-19 vaccine in adolescent patients with JIA on TNFi

#### D. Maritsi, F. Dasoula, G. Vartzelis, D. Dimopoulou, C. Papadaki, M. Tsolia

##### Infectious Diseases, Immunology and Rheumatology Unit, Second Department of Pediatrics, Athens Medical School, Athens, Greece

###### **Correspondence:** D. Dimopoulou

**Introduction:** The post-authorization safety reports of the novel mRNA vaccines against COVID-19 are generally reassuring; nonetheless their safely profile has not been evaluated in adolescents with MRDs on immune-modulating treatment.

**Objectives:** To evaluate the safety and tolerability of the BNT162b2(Pfizer-BioNTech) COVID-19 vaccine in adolescent and young adult patients with juvenile idiopathic arthritis (JIA) on TNFi treatment.

**Methods:** Study population: The study involved 21 subjects aged 16-21 years (median 17 years) with stable JIA who have been diagnosed and treated for at least 1 year with TNFi. In particular, 10 were receiving adalimumab at two weekly intervals, eleven were given etanercept once a week, whereas 15 patients were on concomitant weekly subcutaneous methotrexate. Eight patients had poly-articular JIA, 7 psoriatic JIA and 6 ERA. Written informed consent was obtained at enrolment. Study procedures: The patients received two doses of the COVID-19 vaccine (Pfizer-BioNTech) intramuscularly at 0 and 3 weeks. In addition to the visits for vaccine administration, further visits were planned at 2, 6 and 12 months after enrolment. A blood sample for the evaluation of vaccine immunogenicity is planned to be taken from all of the subjects at the time of enrolment, after 2, 6 and 12 months after the last vaccine dose. All participants were observed for 30 min after the injection in order to assess vaccine safety and tolerability. All patients were given a diary card to record the occurrence of local symptoms or systemic symptoms for the following 14 days. The symptoms were classified as mild or severe (requiring medical attention). Adverse reactions were defined as any reaction that lasted for more than 7 days after the vaccination and serious adverse reactions as any reaction that required medical attention or hospitalization during the study period. Disease activity was evaluated by using the JADAS-27 score at all planned assessments performed so far. The ESR and CRP levels were measured simultaneously. Data were analysed using SPSS 18.0.

**Results:** All subjects were seronegative at baseline. All participants tolerated both doses of the vaccine well. Local reactions were frequent (74%) in the majority of participants, no difference was noted between patients on etnanercept (71%) versus adalimumab (75%)(p=0.09). Localized erythema(73%), pain(72%) and swelling(68%) were among common side effects. There were no differences noted in patients with different JIA types. In addition, systemic reactions [headache (34%), myalgias(23%), tiredness(12%), transient arthralgia(11%)] were relatively infrequent (18%). The type of JIA or medication received did not reveal any differences in the rates of systemic reactions. Most localized and systemic reactions were noted after the second dose of the vaccine (p=0.02). One patient developed an allergic reaction (hives) after the second dose, which was alleviated with anti-histamines. Finally, there were no significant changes in 27-JADAS or laboratory tests as noted at 2 the months’ follow-up.

**Conclusion:** The mRNA vaccine seemed safe and well tolerated in adolescents with JIA on TNFi. Although our sample size was small and a restricted number of patients were included within each JIA-type and treatment groups, it can be concluded that the vaccine assures an adequate safety and tolerability profile and not provoking disease flare. Further studies are needed to evaluate the immune response of patients receiving biological agents in more detail, analyse the immunogenicity of the two-dose schedule, and determine the real duration of immune protection as well as the potential use of a booster dose.

**Disclosure of Interest**

None declared

### P378 The unexpected decrease of Kawasaki syndrome during the second COVID-19 wave: the report from the COVASAKI survey

#### M. V. Mastrolia^1^, R. Agostiniani^2^, C. Azzari^1^, R. Bernardini^2^, U. Bottone^2^, G. B. Calabri^1^, F. Civitelli^2^, R. Consolini^2^, R. Danieli^2^, R. Di Silvio^2^, S. Falorni^2^, L. Gagliardi^2^, S. Grosso^2^, G. Indolfi^1^, M. L'Erario^1^, M. Martini^2^, G. Memmini^2^, D. Peroni^2^, M. Pezzati^2^, G. Suriano^2^, L. Tafi^2^, S. Trapani^1^, A. Vaccaro^2^, P. L. Vasarri^2^, G. Simonini^1^

##### ^1^Meyer Children's University Hospital, Firenze; ^2^Pediatric Tuscany Network, Tuscany, Italy

###### **Correspondence:** M. V. Mastrolia

**Introduction:** During the first pandemic wave, Tuscany reported the fifth Italian highest number of COVID-19 cases in Italy even if this prevalence was lower if compared to other high-prevalence regions in the North of Italy. From September 2020, Tuscany situation has deeply changed with a significant increase of SARS CoV-2 positive cases, currently standing almost 234,000.

**Objectives:** The Pediatric Tuscany Network continued the COVASAKI survey with the aim to track children who received a Kawasaki Syndrome (KS) or Multisystem Infalmmatory syndrome (MIS-C) diagnosis during the second vawe of COVID-19 pandemic.

**Methods:** We retrospectively collected demographics, clinical findings, treatment and outcome of KS and MIS-C children between November 1st, 2020 to April 30th,2021 and compared the number of KS cases during this period to the number of reported KS children in the first pandemic vawe and in the previous five years in the same region.

**Results:** 14 MIS-C children were admitted to 5 Paediatric Units (incidence 2.3/month), 10 boys and 4 girls (mean age of 9.6 years [IQR] 8.8-12). 11/14 patients required intensive care unit admission: 10 needed amines and 3 underwent mechanical ventilation. Echocardiography revealed a reduced left ventricular ejection fraction in 8/14. A diffuse coronary artery ectasia was found in 1. All children completely recovered with a timely immunomodulatory treatment with intravenous immunoglobulins, steroids and, in case of severe cardiac involvement, anakinra. Nasopharyngeal swabs and serological test for SARS CoV-2 resulted positive in 5/13 and 14/14 respectively. The MIS-C incidence rate, adjusted for the 5,170 children hospitalized, resulted 0.27% and represented the 13.9 % of paediatric COVID 19-related hospital admissions in Tuscany. Conversely, the number of observed KS significantly reduced comparing to the first six months of COVASAKI survey: 3 cases, 0.5 incidence/month *vs* 11 cases, 1.8 incidence/month (p <0.03, RR 0.27, 95% CI 0.06 to 0.92). Comparing the 2.7 incidence/month of the 165 diagnosed KS from 1st January 2015 to 31th January 2020, a statistically significant difference has been detected (p <0.0005, RR 0.24, 95% CI 0.07 to 0.59). The same result has been found limiting the analysis to the 92 children with KS diagnosed during the same corresponding 6 months of the last 5 years: 3.0 versus 0.7 incidence/month (p <0.0002, RR 0.21, 95% CI 0.06 to 0.53).

**Conclusion:** Our results seem in accordance with the hypothesis of an infectious trigger in KS pathogenesis. The stay-home imposed by pandemic and the extensive adoption of barrier protection devices have concomitantly reduced the incidence of respiratory infections among general and paediatric population. At this regard, the massive drop in the number of influenza and Syncytial Virus infections during the winter months results emblematic. From this point of view, it could be hypothesized that, in contrast to what had been previously reported in the early stages of its outbreak, the SARS CoV-2 pandemic could lead to a reduction rather than a substantial increase in the number of KS cases. Although indirectly, the behavioural measures adopted to contain the contagion or maybe further mechanisms not yet identified might be the reason.

**Patient Consent Received**

Yes

**Disclosure of Interest**

None declared

### P379 Multisystem inflammatory syndrome in children related to COVID-19 in Hospital Infantil de México Federico Gómez

#### H. Menchaca^1^, P. Ramos^1^, C. Mares Beltran^2^, S. Rodriguez^1^, D. Alpizar-Rodriguez^3^, E. Faugier Fuentes^1^

##### ^1^Hospital Infantil del Mexico Federico Gomez, Hospital Infantil del Mexico Federico Gomez, Ciudad de Mexico, Mexico; ^2^Pediatrics, Mount Sinai Hospital, New York, United States; ^3^Research Unit, Mexican College of Rheumatology, Ciudad de mexico, Mexico

###### **Correspondence:** H. Menchaca

**Introduction:** It was recently observed and described an association between a pediatric hyperinflammatory state and the infection by SARS-CoV-2 which was named Multisystem inflammatory syndrome in children related to COVID-19 (PIMS- TS) or Multisystem inflammatory syndrome (in children) MIS-C.

**Objectives:** We aimed our study at describing the clinical features, epidemiologic characteristics, management, and prognosis.

**Methods:** This is a retrospective, descriptive and observational study performed at the Hospital Infantil de Mexico Federico Gomez, a third-level children’s hospital in Mexico City. The study includes all the cases that met criteria for PIMS-TS/MIS-C of the RCPCH, WHO, and/or CDC, that were diagnosed and treated between March 2020 and March 2021. We identified a total of 41 cases.

**Results:** The depicted table describe the demographics of our studied population. The highest incidence was seen in previously healthy, school-aged children. No differences were noted based on sex. In 50% of the cases, there was history of exposure to COVID-19. 7.3% of patients had an associated comorbidity. The SARS-CoV2 was isolated from CSF in one patient with PIMS. There were no documented cases of macrophage activation syndrome (MAS). While coagulopathy was observed, there were no cases of disseminated intravascular coagulation (DIC). These results are consistent with the results reported by Hoste L et al. Eur J Pediatr. 2021.

**Table:** 

Age (3 m – 17 y)	9 (10 – 11 y)	22%
**Mucocutaneous inflammation signs**	26	63%
**Hypotension or shock**	28	68%
**Features of myocardial dysfunction, pericarditis, valvulitis, or coronary abnormalities (including ECHO findings)**	13	32%
**Evidence of coagulopathy (elevated D-dimers)**	37	90%
**Acute gastrointestinal problems (diarrhea, vomiting, or abdominal pain)**	37	90%
**Elevated markers of inflammation (ESR, CRP, or procalcitonin)**	33	80%
**Intravenous immunoglobulins (IVIG)**	27	66%
**Corticosteroids**	37	90 %
**Mortality**	0	0 %

**Conclusion:** The present study depicts the experience of our institution with the new nosological entity named PIMS. We highlight the null mortality, the effectiveness of steroids and gammaglobulin in lieu of biologic therapy as part of the management, and the predominance of previously healthy patients without significant comorbidities. We consider this a broad evaluation, as our sample size consisted of 41 patients from the same location.

**Disclosure of Interest**

None declared

### P380 Pediatric inflammatory multisystem syndrome temporally-associated with SARS-COV-2 – a Portuguese single centre case series

#### S. B. Miranda^1^, B. Santos^2^, I. Cardoso^3^, F. Aguiar^4,5^, M. Rodrigues^4,5^, I. Brito^4,5^

##### ^1^Paediatrics, Hospital de Braga, Braga; ^2^Rheumatology, Centro Hospitalar do Baixo Vouga, Aveiro; ^3^Paediatrics, Centro Hospitalar Vila Nova de Gaia/Espinho, Vila Nova de Gaia; ^4^Paediatrics and Young Adult Rheumatology Unity, Centro Hospitalar Universitário São João; ^5^Faculty of Medicine of Porto University, Porto, Portugal

###### **Correspondence:** B. Santos

**Introduction:** Across the globe, coronavirus disease 2019 (COVID-19) caused by SARS-CoV-2 appears to affect paediatric population in a milder and nonthreatening way. However, since April 2020 case reports of previously healthy children presenting with unremitting fever, biologic inflammatory syndrome and cardiac dysfunction have been emerging. This syndrome, termed Inflammatory Multisystem Syndrome Temporally Associated with SARS-CoV-2 infection (PIMS-TS), represents a rare complication of COVID-19 in children.

**Objectives:** The aim of this study is to describe the clinical and laboratory characteristics, course, management and outcomes of hospitalized children diagnosed with PIMS-TS in a Portuguese tertiary care hospital.

**Methods:** A retrospective study including children that attended our hospital from April 2020 to April 2021 was performed. All the children fulfilled the case definition of PIMS-TS published by the Centers for Disease Control and Prevention. Data were collected. Statistical analysis was performed using SPSS 26.0.

**Results:** A total of 19 children met the criteria for PIMS-TS, 68% male with a mean age at diagnosis of 8 years old (IQR 5.8-15). They were all caucasian, except for a mixed-race patient, and all previously healthy, except one patient who was obese. Twelve had recent infection by SARS-CoV-2 detected by reverse transcriptase (RT) PCR and 18 had positive IgG serology. One patient had negative RT-PCR and serology, but a positive contact history.

All had fever at diagnosis, with a median duration of 6 days (IQR 5-6).

89.5% had mucocutaneous, gastrointestinal and haematological attainment, respectively. Other affected systems were respiratory (73.7%), cardiovascular (63%), lymphoid organs (52.6%), musculoskeletal (47%), genito-urinary (31.6%) and neurological (26.3%).

Laboratory findings can be found in table 1.

36.8% were admitted in intensive care unit for a median duration of 8 days (IQR 4-9). 42.1% needed respiratory support, 87.5% with supplemental oxygen therapy, 62.5% with mechanical ventilation and 12.5% with non-invasive ventilation.

All patients received intravenous (IV) immunoglobulin, 52.6% IV corticosteroid (CS) pulses and 78.9% IV and oral CS. Other treatments included acetylsalicylic acid (n=18), heparin (n=8) and antibiotic therapy (n=19).

Seventeen fully recovered and 2 had sequelae: coronary artery aneurysms (n=1) and other exertional dyspnea (n=1).

**Conclusion:** In this case series there was a wide spectrum of presenting signs and symptoms and disease severity, ranging from fever and systemic inflammation to critical care admission with myocardial injury, shock, and development of coronary artery aneurysms.

Despite short-term morbidity, there were no mortality cases, with most of them recovering without sequelae.

All physicians providing clinical care to children should consider this rare but severe delayed syndrome in paediatric population.

**Patient Consent Received**

Yes

**Disclosure of Interest**

None declared

**Table 1 (abstract P380).** Laboratory results at presentation

**Hematology**^**a**^		
	**Reference range**	
**Total white blood cells count, x10**^**9**^**/L**	4.0-11.0	10.19 (7.37-14.85)
**Neutrophil count, x10**^**9**^**/L**	1.5-7	10.38 (5.71-11.79)
**Lymphocyte count, x10**^**9**^**/L**	1.5-4	0.94 (0.67-1.45)
**Hemoglobin, g/dL**	12-16	11 (9.4-12.1)
**Platelet count, x10**^**9**^**/L**	150-400	156 (104-248)
***Inflammatory markers*** ^**a**^		
	**Reference range**	
**C-reactive protein, mg/L**	0-3	218.5 (132-262)
**Procalcitonin, ng/mL**	0-0.05	2.72 (0.00-8.18)
**Ferritin, ug/L**	-140	598 (226-769)
***Cardiac markers*** ^**a**^		
**Troponin, ng/L**	0-34	118 (34-1021)
**CK-MB, ng/mL**	0.00-6.4	0.7 (0.3-8.1)
**Pro-BNP, pg/mL**	0-100	238.4 (67.5-525)

Abbreviations: *PIMS-TS* pediatric inflammatory multisystem syndrome temporally associated with SARS-CoV-2

^a^ Data showed as median and inter Quartile Range (P25, P75)

^b^ Data showed as frequency and percentage

### P381 Polyclonal expansion of TCR VBETA 21.3+ CD4+ and CD8+ T CELLS is a hallmark of multisystem inflammatory syndrome in children

#### M. Moreews^1^, K. Le gouge^2^, S. Khaldi-Plassart^3^, R. Pescarmona^4^, A.-L. Mathieu^1^, C. Malcus^5,6^, S. Djebali^1^, A. Bellomo^1^, O. Dauwalder^1^, M. Perret^4^, M. Villard^4^, E. Chopin^7^, I. Rouvet^7^, F. Vandenesh^1^, C. Dupieux^1^, R. Pouyau^8^, S. Teyssedre^8^, M. Guerder^8^, T. Louazon^9^, A. Moulin-Zinsch^10^, M. Duperril^11^, H. Patural^11^, L. Giovannini-Chami^12^, A. Portefoix^13^, B. Kassai^13^, F. Venet^1,5^, G. Monneret^5,6^, C. Lombard^4^, H. Flodrops^14^, J.-M. De Guillebon^15^, F. Bajolle^16^, V. Launay^17^, P. Bastard^18,19^, S.-Y. Zhang^18,19,20^, V. Dubois^21^, O. Thaunat^21,22^, J.-C. Richard^23^, M. Mezidi^23^, O. Allatif^1^, K. Saker^1,24^, M. Dreux^1^, L. Abel^18,19,20^, J.-L. Casanova^18,19,20,25^, J. Marvel^1^, S. Trouillet-Assant^1,24^, D. Klatzmann^2,26^, T. Walzer^1^, E. Mariotti-Ferrandiz^2,26^, E. Javouhey^6,8^, A. Belot^1,5^

##### ^1^CIRI, Centre International de Recherche en Infectiologie, Univ Lyon, Inserm, U1111, Université Claude Bernard, Lyon 1, CNRS, UMR5308, ENS de Lyon, Lyon; ^2^Sorbonne Université, UPMC Univ Paris 06, INSERM UMRS 959, Immunology Immunopathology-Immunotherapy (i3), Paris; ^3^RAISE, Pediatric Nephrology, Rheumatology, Dermatology Unit, Hôpital Femme Mère Enfant, Hospices Civils de Lyon; ^4^Immunology Laboratory, Hospices Civils de Lyon; ^5^Hospices Civils de Lyon, Edouard Herriot Hospital, Immunology Laboratory; ^6^EA 7426 “Pathophysiology of Injury-Induced Immunosuppression, Université Claude Bernard Lyon 1 - Hospices Civils de Lyon – bioMérieux; ^7^Cellular Biotechnology Department and Biobank, Hospices Civils de Lyon, Lyon; ^8^Réanimation Pédiatrique Hôpital Femme-Mère-Enfant Hospices Civils de Lyon, Bron; ^9^Service de pédiatrie, Centre Hospitalier de Valence, Valence; ^10^Unité medico-chirurgicale des cardiopathies congénitales, hôpital Louis-Pradel, hospices civils de Lyon, Bron; ^11^Pediatric intensive care unit - University hospital of Saint-Étienne, Saint-Etienne; ^12^Pediatric Pulmonology and Allergology Department, Hôpitaux pédiatriques de Nice CHU-Lenval, Nice; ^13^Center of Clinical Investigation, Lyon University Hospital, Bron, France; ^14^Service de Pédiatrie, Groupe Hospitalier Sud Réunion, CHU de La Réunion, Saint-Pierre, Réunion; ^15^Service de Néphrologie, Rhumatologie pédiatrique, Hôpitaux pédiatriques de Nice CHU-Lenval, Nice; ^16^Hôpital Necker Enfants Malades, Centre de référence M3C, AP-HP, Paris; ^17^Urgences pédiatriques, Hôpital femme Mère Enfant, Hospices Civils de Lyon, Bron; ^18^Laboratory of Human Genetics of Infectious Diseases, Necker Branch, INSERM U1163, Necker Hospital for Sick Children; ^19^University of Paris, Imagine Institute, Paris, France; ^20^St. Giles Laboratory of Human Genetics of Infectious Diseases, Rockefeller Branch, The Rockefeller University, New York, United States; ^21^EFS Auvergne Rhône Alpes, laboratoire Histocompatibilité, Décines; ^22^Department of Transplantation, Nephrology and Clinical Immunology, Edouard Herriot University Hospital; ^23^Médecine Intensive-Réanimation, Hôpital de la Croix-Rousse, Hospices Civils de Lyon; ^24^Laboratoire de Virologie, Institut des Agents Infectieux, Laboratoire associé au Centre National de Référence des virus des infections respiratoires, Hospices Civils de Lyon, Lyon, France; ^25^Howard Hughes Medical Institute, New York, United States; ^26^Assistance Publique - Hôpitaux de Paris, Hôpital Pitié-Salpêtrière, Biotherapy and Département Hospitalo-Universitaire Inflammation-Immunopathology-Biotherapy (i2B), Paris, France

###### **Correspondence:** M. Moreews

**Introduction:** At the end of April 2020, European clinicians warned the Public Health Agencies about an abnormal increase of Kawasaki-like diseases and myocarditis requiring critical care support in the context of the ongoing COVID-19 epidemic in children. American clinicians also reported a large outbreak of severe inflammation in children following COVID-19 infection, a condition that is now named Pediatric Inflammatory Multisystemic Syndrome (PIMS) or Multisystem Inflammatory Syndrome in children (MIS-C).

**Objectives:** As MIS-C combines clinical features of Kawasaki disease (KD) and Toxic Shock Syndrome (TSS), we aimed to compare the immunological profile of pediatric patients with these different conditions.

**Methods:** We analysed blood cytokine expression, and the T cell repertoire and phenotype in 36 MIS-C cases, which were compared to 16 KD, 58 TSS, and 42 COVID-19 cases.

**Results:** We observed an increase of serum inflammatory cytokines (IL-6, IL-10, IL-18, TNF-a, IFNg, CD25s, MCP1, IL-1RA) in MIS-C, TSS and KD, contrasting with low expression of HLA-DR in monocytes. We detected a specific expansion of activated T cells expressing the Vβ21.3 T cell receptor β chain variable region in both CD4 and CD8 subsets in 75% of MIS-C patients and not in any patient with TSS, KD, or acute COVID-19; this correlated with the cytokine storm detected. The T cell repertoire returned to baseline within weeks after MIS-C resolution. Vβ21.3+ T cells from MIS-C patients expressed high levels of HLA-DR, CD38 and CX3CR1 but had weak responses to SARS-CoV-2 peptides *in vitro.* Consistently, the T cell expansion was not associated with specific classical HLA alleles.

**Conclusion:** Thus, our data suggested that MIS-C is characterized by a polyclonal Vβ21.3 T cell expansion not directed against SARS-CoV-2 antigenic peptides, which is not seen in KD, TSS and acute COVID-19.

**Patient Consent Received**

Yes

**Disclosure of Interest**

None declared

### P382 Frequency and clinical features of SARS-COV-2 infection in a cohort of patients with juvenile idiopathic arthritis

#### R. Naddei, M. Amico, M. Castaldo, M. Alessio

##### Pediatric Rheumatology Unit, Mother and Child Department, University of Naples Federico II, Naples, Italy

###### **Correspondence:** R. Naddei

**Introduction:** To date, the clinical features of SARS-CoV-2 infection in children and adolescents with juvenile idiopathic arthritis (JIA) and the impact of immunoregulator drugs on COVID-19 in these patients are still scarcely characterized.

**Objectives:** To evaluate the frequency and the clinical characteristics of COVID-19 in a cohort of patients with JIA.

**Methods:** Between 1^st^ April and 28^th^ May, 21, parents of patients with JIA were asked to answer a survey investigating the history of contacts with confirmed cases of COVID-19 and the results of diagnostic tests for SARS-CoV-2 in these patients. In subjects with a diagnosis of SARS-CoV-2 infection, the clinical features of COVID-19 were recorded. Demographic and clinical data regarding JIA were also collected from clinical charts.

**Results:** 147 patients answered the survey (median age 11.9, IQR 9.5-15; females 74.8%). The most frequent JIA subtype was the oligoarticular one (53.6%), followed by polyarticular (23.1%) and the systemic (10.2%) JIA. There were 35 (23.8%) patients presenting contact histories with confirmed COVID-19 cases. 18 patients had a positive polymerase chain reaction test for SARS-CoV-2 at the nasopharyngeal swab, resulting in a COVID-19 prevalence of 12.2% in our cohort. No statistical difference was observed with regard to age and sex patients with or without SARS-CoV-2 infection. No patient with polyarticular JIA presented COVID-19, resulting in a statically significant difference compared to patients without COVID-19 history (0% vs 25.6%, p=0.013). 3 patients presented remission off-medication at the time of COVID-19 and 1 patient was in monotherapy with NSAID. 4 patients were treated only with methotrexate, while 5 only with a biotechnological disease modifying anti-rheumatic drug (bDMARD). 4 patients were on combined therapy with both MTX and a bDMARD. 9 out of the 18 patients with COVID-19 presented symptoms during the infection: fever, musculoskeletal pain and cough were the most frequent (27.8%), followed by headache (22.2%). 3 patients were hospitalized during COVID-19, 2 of them in order to undergo the periodic drug infusions for JIA (tocilizumab and infliximab); the third was hospitalized for clinical observation since the parents reported cough with dyspnea and desaturation at home, which were not confirmed during her stay in hospital. No one of our patients with SARS-CoV-2 infection presented a severe COVID-19 requiring oxygen or developed a multisystem inflammatory syndrome, irrespective of the ongoing treatment for JIA.

**Conclusion:** We found a COVID-19 prevalence of about 12% in our cohort of patients with JIA and reported no cases of severe SARS-CoV-2 infection or COVID-19-related complications in our patients. Our data support that the interruption of the ongoing treatment for JIA in patients with COVID-19 may not be needed.

**Disclosure of Interest**

None declared

### P383 Management of PIMS-TS in children from a low-income country: a single center experience

#### H. Nassih, R. Elqadiry, A. Bourrahouat, I. Aitsab

##### Pediatrics, Mohammed 6 University Hospital Center of Marrakesh, Marrakesh, Morocco

###### **Correspondence:** H. Nassih

**Introduction:** PIMS-TS features a toxic shock-like syndrome or Kawasaki-like syndrome in the setting of SARS-CoV-2 positive diagnostic testing. The main cause is a severe cytokine storm resulting in a wide spectrum of clinical phenotypes, ranging from mild self-resolving to severe life-threatening presentations.

**Objectives:** To report the experience of a single center from a low-income country, in the management of PIMS-TS.

**Methods:** A retrospective descriptive study of 9 cases with PIMS-TS.

**Results:** We had 4 boys and 5 girls. The mean age was of 11-year-old, with extremes of 7 and 15-yesr-old. Medical history was marked by one case of Churg and Strauss syndrome under steroids, one case of recurrent idiopathic pancreatitis under steroids and azathioprine, one case of sarcoidosis under steroids and mycophenolate mofetil, and one case of nephrosis. All cases had history of contact with a COVID-19 positive case. SARS-CoV-2 RT-PCR was positive in 5 cases, while SARS-CoV-2 IgM antibodies were positive in one case and SARS-CoV-2 IgG antibodies were positive in all cases. Telltale symptoms were acute fever in one case, protracted fever in 4 cases, respiratory tract infection (rhinitis, caught, polypnea) in one case, Kawasaki -like symptoms (cheilitis, conjunctivitis, cervical lymphadenopathies and rush) in 2 cases, myocarditis in 3 cases, gastrointestinal symptoms (abdominal pain, vomiting, diarrhea) in 3 cases, toxic shock in one case, uveitis in one case, acute kidney injury in 8 cases, and liver failure in 4 cases. All patients had high acute phase reactants (ESR, CRP, ferritin). Hematological involvement was in the form of microcytic anemia in 3 cases, hemolytic anemia in 2 cases, polynuclear neutrophils' leukocytosis in 4 cases, leucopenia in 4 cases, lymphopenia in 3 cases, thrombocytosis in 5 cases, and thrombocytopenia in 3 cases. Two children had hepatic cytolysis, while 6 had high D-dimers and LDH levels, 5 had high fibrinogen level, and 3 had high triglycerides level. Management was based on steroids in all cases (3 pulses of methylprednisolone at 1g/1.73 m^2^/day, followed by oral prednisone for a mean time of 3 weeks). Meanwhile, one case was put under tocilizumab and one case was put under intravenous immunoglobulins. Hemodialysis was needed in the acute phase in 4 children. Two cases were put under intravenous noradrenaline, while 3 cases were treated by antibiotics. Anticoagulation was prescribed in 3 cases. Evolution was marked by complete remission in 8 cases and in a mean time of 2 weeks. We deplore one death from multiple organ dysfunction Syndrome following septic choc.

**Conclusion:** In our experience, PIMS-TS was more prevalent in immunocompromised children. Steroids were effective as a first line treatment. Complete remission with no sequela is the rule.

**Disclosure of Interest**

None declared

### P384 Spectrum of 45 children with pediatric multi-system inflammatory syndrome temporally associated with COVID-19 from an Indian state of Gujarat

#### D. B. Pandya^1^, J. R. Patel^2^, on behalf of Amruta, Arogyam, Bambhani, Bhojvani, Divine, Dobariya, GGH-Jamnagar,GMRES Valsad,Hope, Kalola, Lifeline, Maa Sharda,PDU, Panth, Rainbow, Saurashtra, Sanidhya, Shaishav & Thakker Children's Hospitals, Gujarat & AOP-Rajkot Team, India.

##### ^1^Pediatric Department, Dev Pediatric Rheumatology & Immunology Centre; ^2^Pediatric Department, Amruta Hospital, Rajkot, India

###### **Correspondence:** D. B. Pandya

**Introduction:** Paediatric multisystem inflammatory syndrome (PIMS) is characterized by high fever, acute gastrointestinal manifestations, Kawasaki-like presentation, multisystem involvement with severe systemic inflammation.

**Objectives:** To describe clinical presentation, laboratory findings, management and prognosis in 45 children affected with PIMS.

**Methods:** We gathered a retrospective data of 45 children who were presented to different paediatric hospitals in our region between May 2020 and May 2021. Our data included demographics, presenting symptoms, examination findings, investigations, management and follow up.

**Results:** There were37 males(82%) and 8 females(18%) aged from 1 month to 16 years.

Table A shows characteristics of 45 children with PIMS

**Table:** 

Clinical Presentation	Total Number of Patients = 45 (%)
Fever	45 (100%)
Gastrointestinal Symptoms	37 (82%)
Rash	29 (64%)
Oral mucosal changes	24 (53%)
Red Eyes	21 (47%)
Edema of hands and/or feet	17 (38%)
Lymphadenopathy	2 (5%)
Cardiovascular System (CVS) involvement including shock	7 (15.5%)
Central Nervous Systems (CNS)	6 (13%)
Respiratory involvement (RS)	6(13%)
Renal Involvement	3 (7%)
Musculoskeletal (MSK) involvement	9 (20%)
Epidemiological Link	Observed /total (%)
SARS-COV 2 Serology Positive	31/35 (89%)
Contact with Proven CoVID patient in previous 4-6	12 / 45 (27%)
CoVID Antigen Test Positive	1/6 (16%)
CoVID RT PCR Positive	1/6 (16%)
Basic Laboratory Changes at Onset	Observed/total (%)
Anaemia (Hemoglobin < 10 gm%)	18/45 (40%)
Lymphopenia (Absolute Lymphocyte Count < 1000/ul)	27/45 (60%)
Thrombocytopenia (Platelet count < 1,50,000/ul)	14/45 (31%)
CRP > 40 mg/L	40/45 (89%)
ESR > 40 mm/1^st^ hour	23/31 (74%)
Elevated Pro-calcitonin	6/9 (66%)
SGPT above normal range	16/ 34 (47%)
Ferritin more than normal range but below < 1000 ng/ml	24/37 (65%)
*Ferritin > 1000 ng/ml*	*2/37 (5%)*
LDH above normal range but < 1000 IU/L	20/21(95%)
Albumin level lower than normal value	15/17 (88%)
Extremely elevated D-Dimer	*9/34 (26%)*
Deranged PT, aPTT	2 / 18 (11%)
Low Fibrinogen	1/2 (50%)
Elevated NT Pro BNP > 1000	9/15 (60%)
Elevated Troponins	2/5 (40%)
Elevated IL-6 > 100 pg/ml	3/4 (75%)
Elevated Triglycerides	2/2 (100%)
2D ECHO Findings	Observed /total (%)
Mild Pericardial Effusion	17/41(42%)
Mitral Regurgitation	14/41 (34%)
Left Ventricular Dysfunction	14/41 (34%)
*Small Coronary Aneurysms*	*6/41 (14%)*
*Large Coronary Aneurysm*	*2/41 (5%)*
HRCT findings	
Bilateral pleural effusions	2/8 (25%)
*Severe CoVID pneumonia (CoRADS Score 14/25)*	*1/8 (12.5%)*
Management	Observed / Total (%)
Only IV Immunoglobulin (Ig) 2gm/kg Single Dose	4/45 (9%)
Only Steroids (IV or Oral)	16/45 (36%)
IVIg + Steroids	20/45 (45%)
Oral Aspirin	16/45 (36%)
Low Molecular Weight Heparin	6/45 (13%)
Tocilizumab	1/45 (2%)
Oxygen Therapy	8/45 (18%)
Inotropic Support	7/45 (15.5%)
Ventilator Care	1/45 (2%)
Follow Up	Observed/Total (%)
Doing well off medications	35/40 (88%)
Death	1/45 (2%)

**Conclusion:** The commonest presenting features were high fever, gastrointestinal symptoms and Kawasaki–like manifestations. Systemic involvement could be depicted as CVS > CNS > RS > Kidney. Arthritis was noted in few patients. Lymphopenia, high CRP with moderately elevated ESR & ferritin were consistent findings. On echocardiography, pericardial effusion and left ventricular dysfunction were found to be more common than coronary aneurysms. Severe lung involvement was the least common. There was only one death reoprted. Any child who presents with high fever, acute GI symptoms,kawasaki-like manifestations,multisystem involvement with systemic inflammation especially 2-4 weeks after a peak of CoVID19 cases in the region, PIMS must be considered in differentials. Early diagnosis, IVIg(2gm/kg) and a short course of high dose steroids(10-30mg/kg/day) are life-saving in severe cases.

**Patient Consent Received**

Yes

**Disclosure of Interest**

None declared

### P385 SARS-COV2 chilbalin-like lesions in a cross-sectional study of children and adolescent

#### F. Peri^1^, A. Tommasini^1^, S. Della Paolera^1^, L. Levantino^1^, F. Biscaro^2^, S. Martelossi^2^, A. Taddio^1^

##### ^1^University of Trieste, Trieste; ^2^Pediatric Department, Ca' Foncello Hospital, Treviso, Italy

###### **Correspondence:** F. Peri

**Introduction:** Chilblain-like lesions have become a common complaint among children and adolescent during COVID-19 pandemic. A clear and objective relationship between this skin manifestation and SARS-CoV-2 infection has not been fully established neither its potential triggering rheumatologic disorders.

**Objectives:** To characterize a group of children and adolescents with chilblain-like lesions.

**Methods:** Data from out-patients of two northeast Italian hospitals were collected between December 2020 and April 2021. Clinical evaluation, blood test, interferon score and capillaroscopy were recorded in an electronic database.

**Results:** We collected data from 35 patients, almost all adolescents (mean age: 13.1 years, 7 male and 28 females), with evidence of chilblain-like lesions. 5/35 had a parent who presented with COVID-19-related symptoms and/or with a positive swab test for SARS-CoV2 in the previous months. Only 2/35 had a positive swab test respectively three and four months before clinical evidence of chilblain-like lesions and none of those tested had a positive IgG serology. The average duration of the skin lesions, which appeared to be mainly localized to the toes, at the time of the first visit was 13.4 weeks. Two patients presented with signs of arthritis and tenosynovitis of the ankles. No other physical complaints were reported by the other patients. The most common capillaroscopic pattern was characterized by mild pericapillary oedema without hemorrhages or capillary abnormalities. No alterations in whole blood count and inflammatory markers were noted. 3/35 showed antinuclear antibodies in low counts. Interferon score was positive in 7 patients with a maximum of 10.1. (n.v. < 2.4) A trial of oral prednisone was the most common therapeutic approach in 17/35 patients. Three patients received Nifedipine and a course of hydroxychloroquine was started in those with severe complaints and a positive interferon score.

**Conclusion:** Chilblain-like lesions presented as a benign clinical entity without a clear and objective proof of SARS-CoV2 contact in almost of the cases. All the investigations performed did not show any evidence of underlying systemic inflammation except for an enhanced interferon mediated response in some cases. We believe a strict follow-up of these cases is strongly warranted.

**Patient Consent Received**

Yes

**Disclosure of Interest**

None declared

### P386 Our experience with COVID-19- clinical features and outcomes in pediatric patients with rheumatic diseases treated with biologic and nonbiologic dMARD therapy

#### M. Šenjug Perica^1^, M. Vidović^2^, H. Munivrana Škvorc^1^, K. Rubelj^2^, L. Tambić Bukovac^1^

##### ^1^Department of Pediatric Rheumatology, Childrens Hospital Srebrnjak; ^2^Department of Pediatric Rheumatology, University Hospital Sisters of Mercy, Zagreb, Croatia

###### **Correspondence:** M. Vidović

**Introduction:** Although it is known that COVID-19 related diseases are less frequent and less aggressive in children, pediatric patients with rheumatic diseases are at higher risk for infections due to immunomodulatory therapy and illness itself.

**Objectives:** The objective was to assess clinical features and outcomes of COVID-19 among pediatric patients with rheumatic diseases being treated with biologic (bDMARD) and nonbiologic (nbDMARD) therapy.

**Methods:** A retrospective search of two pediatric rheumatology hospital centre database was done for patients with rheumatic diseases receiving bDMARD and nbDMARD therapy during SARS-CoV-2 infection. Data regarding diagnosis of SARS-CoV-2 infection, clinical presentation, management of infection and outcome were analyzed for each patient receiving bDMARD or nbDMARD including previous treatment duration, disease activity and eventual therapy intermission.

**Results:** A total of 34 patients was identified after the database search. Due to lack of SARS-CoV-2 infection evidence, 7 patients were excluded from the analysis. Among 27 analyzed patients, 21 (77,8%) were female and 6 (22.2%) were male, with mean age of 11,46 years (min 4- max 18). Twenty five were juvenile idiopathic arthritis (JIA) patients (14 have oligoarticular, 5 polyarticular RF -, 2 polyarticular RF+, 2 ERA, 2 psoriatic JIA), one patient has only uveits, and one juvenile SLE. Mean duration of rheumatic disease was 5,32 years. Among oligo JIA patients two had uveitis and one ERA patient had Crohn’s disease. Mean duration of bDMARD therapy at the time of SARS-CoV-2 infection was 1,31 years and nbDMARD 2,5 years. Only two JIA patients were receiving small dose of oral steroids (5mg/daily), 19 patients were on methotrexate therapy, patient with Crohn’s and JIA received azathioprine, and jSLE patient hydroxychlorquine. Regarding bDMARDs, 8 patients were being treated with adalimumab, 2 with etanercept and 2 with tocilizumab. NSAID-s were concomitant therapy in 21 patient.

Twenty four patients (88,9%) had positive PCR test and 3 patients (11,1%) had positive serology test for SARS-CoV-2. None of 27 patients diagnosed with COVID-19 required hospitalization. Seven were completely asymptomatic (25,9%), 10 (37,0%) had mild symptoms such as cough, headache or anosmia, without fever, 6 patients (22,2%) had only subfebrile episodes for a day or two, 3 patients (11,1%) were febrile less than 5 days and only one patient experienced dyspnea but without objective signs of lung involvement. One JIA patient with cough during COVID 19 was treated with azitromicin, while others received only symptomatic therapy or no treatment at all.

Ten patients (37,0%) continued receiving bDMARs and nbDMARDs, all of them asymptomatic and diagnosed due to epidemiological screening (3 by serological and 7 by PCR test). NbDMARD was paused for 1 or 2 weeks (5 and 7 patients respectively) and bDMARDs were paused in 3 patients for2 weeks in agreement with pediatric rheumatologist. Both bDMARD and nbDMARD were paused in 2 patients (1 and 2 weeks respectively). Decision regarding duration of therapy intermission was based on COVID-19 symptoms and due to parents concern in two occasions. No flares of rheumatic diseases were noticed, only 2 patients with active disease previous to infection needed therapy readjustment.

**Conclusion:** Data on COVID-19 in pediatric patients with rheumatic diseases is still scarce. Our group of patients receiving bDMARD and nbDMARD had mild presentation of COVID-19, not requiring hospitalization. According to our data, treatment with bDMARD or nbDMARD does not pose risk for more severe COVID-19 disease. Due to small sample size more studies are needed for definite conclusions.

**Patient Consent Received**

Yes

**Disclosure of Interest**

None declared

### P387 A rapid review and meta-analysis of the laboratory phenotype of multisystem inflammatory syndrome in children

#### W. Slamang^1^, L. H. Abdullahi^2^, F. Mustafa^3^, M. J. T. Harrison^4^, C. M. Butters^1^, C. T. Deakin^5^, C. Scott^1^, K. Webb^1,6^

##### ^1^Department of Paediatric Rheumatology, School of Child and Adolescent Health, Red Cross Children's Hospital, University of Cape Town, Cape Town, South Africa; ^2^African Institute for Development Policy (AFIDEP), Nairobi, Kenya; ^3^Department of Immunology and Department of Paediatrics and Child Health, University of Pretoria, Pretoria; ^4^Fort Beaufort Provincial Hospital, Fort Beaufort, South Africa; ^5^Infection Immunity and Inflammation Research and Teaching UCL GOS Institute of Child Health, NIHR Great Ormond Street Biomedical Research Centre and Centre for Adolescent Rheumatology Versus Arthritis at UCL, UCLH and GOSH, London, UK; ^6^The Francis Crick Institute. Crick African Network, London, United Kingdom

###### **Correspondence:** W. Slamang

**Introduction:**

Laboratory features are included in the current case definitions of the multisystem inflammatory syndrome in children (MIS-C) associated with the severe acute respiratory syndrome coronavirus-2 (SARS-CoV2).

**Objectives:**

We reviewed the clinical laboratory test values reported in MIS-C, to better characterise the laboratory phenotype.

**Methods:**

A comprehensive search of the WHO COVID-19 database was conducted from January to November 2020. The median test values reported in children 0-19 years with MIS-C, were extracted for each laboratory variable reviewed. Random effects meta-analyses were performed and the quantile estimation method used to determine estimates of the pooled median for each variable. The risk of bias was assessed using the QUADAS-2 tool.

**Results:**

Twenty-two observational studies were included in the analyses N=831 children. The overall risk of bias was considered moderate with varying heterogeneity between the studies reviewed for each variable (I^2^ 52.4 – 96.63%). The estimated pooled median values which were abnormal included CRP 188.12 mg/L (95% CI 158.54, 217.70), ESR 60.07 mm/hr (95% CI 49.88, 70.26), ferritin (587.63 ng/ml; 95% CI (476.29, 698.97), pro-BNP 6927ng/ml (95% CI 794.52, 13061.13), D-dimer 3.03 ng/L (95% CI 2.41, 3.65), absolute neutrophil count 9.61x10^9^L (95% CI 7.72, 11.50), absolute lymphocyte count 0.92 x10^9^/L (95% CI 0.74, 1.10), platelet count 150.82 x10^9^/L (95% CI 135.09, 145.07), albumin 28.89g/L (95% CI 25.56, 32.22) and sodium 132.67mmol/L (95% CI 131.44, 133.89).

Limitations

The analyses were limited by varying reporting methods and the use of different normal reference ranges across studies.

**Conclusion:**

Markers of inflammation, coagulopathy and cardiac dysfunction, were confirmed as important features of the laboratory phenotype of MIS-C. It is necessary however, to further compare the laboratory values of MIS-C to those with COVID-19 and other hyperinflammatory syndromes in children, to develop evidence-based diagnostic criteria.

**Disclosure of Interest**

None declared

### P388 The clinical course and short-term health outcomes of multisystem inflammatory syndrome in children in the single pediatric rheumatology center

#### B. Sozeri^1^, S. caglayan^1^, V. Atasayan^2^, K. Ulu^1^, T. Coskuner^1^, O. P. Akbay^3^, C. Hasbal Akkus^3^, G. Atay^4^, E. Salı^5^, M. Karacan^2^, T. Onel^2^, S. Erdogan^4^, F. Demir^1^

##### ^1^Pediatric Rheumatology; ^2^Pediatric Cardiology; ^3^Pediatrics; ^4^Pediatric Intensive Care Unit; ^5^Pediatric Infectious Disease, University of Health Sciences, Istanbul, Umraniye Training and Research Hospital, Istanbul, Turkey

###### **Correspondence:** B. Sozeri

**Introduction:** Multisystem inflammatory syndrome in children (MIS-C) is a rare but severe condition resulting in excessive response of the immune system after SARS-CoV-2 infection.

**Objectives:** We report a single center cohort of children with MIS-C, describing the spectrum of presentation, therapies, clinical course, and short-term outcomes.

**Methods:** This is a prospective observational study from to a tertiary pediatric rheumatology center including patients (aged 1 month to 21 years) diagnosed with MIS-C between April 2020-April 2021. The Centers for Disease Control and Prevention case definition for MIS-C is used to define a confirmed case of MIS-C. Demographic, clinical, laboratory results and follow-up data were collected through the electronic patient record system and analyzed.

**Results:** A total of 67 patients with MIS-C were included in to the study. Fever was detected in all patients; gastrointestinal system symptoms was found in 67.2% of the patients, rash in 38.8%, conjunctivitis in 31.3%, hypotension in 26.9% myocarditis and/or pericarditis in 22.4%, respectively. Respiratory symptoms were only in 5 patients (7.5%). Kawasaki Disease like presentation was found 37.3 % of patients. The mean duration of hospitalization was 11.8 ±7.07 days. Fifty- seven patients (85%) received intravenous immunoglobulin (IVIG), 45 (67%) received corticosteroids, 17 (25.3%) received anakinra, and one (1.5%) received tocilizumab. Seven of the patients (10.4%) underwent therapeutic plasma exchange (TPE). In 21 (31.3%) patients, a pediatric intensive care unit (PICU) was required in a median of 2 days. The first finding to improve was fever, while the first parameter to decrease was ferritin (median 6.5 days (IQR, 4-11.2 days)). Sixty-five patients were discharged home with a median duration of hospital stay of 10 days (IQR, 7-15 days).

**Conclusion:** The patients with MIS-C may have severe cardiac findings and intensive care requirements in admission and hospital follow-up. The vast majority of these findings improve with effective treatment without any sequelae until discharge and in a short time in follow-up. Although the pathogenesis and treatment plan of the disease are partially elucidated, follow-up studies are needed in terms of long-term prognosis and relapse probabilities.

**Disclosure of Interest**

None declared

### P389 Clinical course of COVID-19 in children with rheumatic disease under biologic therapy from Turkey

#### B. Sözeri^1^, K. Ulu^1^, Ü. Kaya^2^, F. Haşlak^3^, A. P. Kisaarslan^4^, G. Otar Yener^5^, Ö. Baba^6^, Ö. Altuğ Gücenmez^7^, N. Şahin^8^, E. Bağlan^9^, H. E. Sönmez^10^, F. Çakmak^11^, K. Öztürk^12^, D. G. Yıldırım^13^, S. Şener^2^, K. Barut^3^, E. D. Batu^2^, M. Yıldız^3^, Ö. Başaran^2^, A. Adrovic^3^, S. Şahin^3^, S. Özdel^9^, Y. Bilginer^2^, H. Poyrazoğlu^4^, T. Coşkuner^1^, S. Çağlayan^1^, F. Demir^1^, S. Yüksel^14^, M. Kalyoncu^6^, Ö. Kasapçopur^3^, S. Özen^2^, N. Aktay Ayaz^11^

##### ^1^Pediatric Rheumatology, University of Health Sciences, Ümraniye Research and Training Hospital, Istanbul; ^2^Pediatric Rheumatology, Hacettepe University, Ankara; ^3^Pediatric Rheumatology, Istanbul University-Cerrahpasa, Istanbul; ^4^Pediatric Rheumatology, Erciyes University, Kayseri; ^5^Pediatric Rheumatology, Şanlıurfa Research and Training Hospital, Şanlıurfa; ^6^Pediatric Rheumatology, Karadeniz Technic University, Trabzon; ^7^Pediatric Rheumatology, Dr Behcet Uz Children's Hospital, Izmir; ^8^Pediatric Rheumatology, Bursa City Hospital, Bursa; ^9^Pediatric Rheumatology, Sami Ulus Training and Research Hospital, Ankara; ^10^Pediatric Rheumatology, Kocaeli University, Kocaeli; ^11^Pediatric Rheumatology, Istanbul University; ^12^Pediatric Rheumatology, Medeniyet University, Istanbul; ^13^Pediatric Rheumatology, Diyarbakır Children’s Hospital, Diyarbakır; ^14^Pediatric Rheumatology, Pamukkale University, Denizli, Turkey

###### **Correspondence:** B. Sözeri

**Introduction:** What a biological disease-modifying antirheumatic drugs (bDMARDs) and/or underlying rheumatological diseases, which we frequently use in our pediatric rheumatology practice, affect the clinical course of COVID-19 has not been fully demonstrated.

**Objectives:** Here, we aimed to reveal the course of COVID-19 infection in patients with rheumatic disease and receiving bDMARD treatment.

**Methods:** This was a retrospective, multicenter study in patients with a biological treatment had been initiated. This real-life study is based on secondary data collection from medical records of patients evaluated at the 14 Pediatric Rheumatology Clinics in Turkey from April 2020 to April 2021. The diagnosis of COVID-19 was confirmed in 101 patients by nasal PCR and in 10 patients by antibody test.

**Results:** The study population of 112 patients consisted of 70 females (63.6%). The mean age of patients was 12.87 ± 4.69 years. The primary diagnosis of patients was as follows; 59 juvenile idiopathic arthritis, 33 systemic autoinflammatory diseases, 10 vasculitis, 8 connective tissue diseases. The mean duration of primary disease was 4.62±3.65 years. Nineteen patients had also additional comorbid diseases (hypertension, Chron's disease, hereditary spherocytosis, and chronic renal failure, astma, cardiomyopathy, adrenal insufficiency in individual patients). Prior to COVID-19 infection, 35 patients (31.8%) were using canakinumab, 10 were infliximab (9.1%), 25 were adalimumab (21.8%), 18 were etanercept (16.4%), 9 were tocilizumab (8.2%), 4 were anakinra (3.6%), 6 were rituximab (5.5%), 1 was abatacept (0.9%), and 3 was tofacitinib (2.7%). The median exposure time of a biologic drug was 13.5 months. Additionally, 66 patients were using DMARD, and 27 patients were also receiving corticosteroid. 70 (63.6%) patients had at least one COVID-19-related symptom (fever, cough, diarrhea, myalgia, anosmia and/or rash), while 40 (36.4%) patients were asymptomatic. Respiratory findings were seen in 26% of all patients, 7 patients also had pathology in computed tomography. Hospitalization was required in 25 patients (22.7%) at median of 6 days (IQR: 4-10). Five patients developed MIS-C and 2 of these patients were followed up in the pediatric intensive care unit. Laboratory tests revealed that fourteen patients had elevated acute phase reactants, ten had elevated D-dimer levels, 5 had lymphopenia (<1000/mm^3^), and five had hyperferritinemia.

**Conclusion:** In patients with underlying comorbidities, COVID-19 can have a severe course regardless of the use of bDMARD. In the light of these findings, it would not be correct to say that the currently used bDMARDs worsen the course of COVID-19 infection or to say whether they affect the severity of the disease, but still, the disease findings-modifying effects of these drugs, especially high fever and myalgia, have been observed.

**Patient Consent Received**

Yes

**Disclosure of Interest**

None declared

### P390 Medium-term outcomes of the multisystem inflammatory syndrome in children (MIS-C) from two tertiary care centers of South India

#### A. Tiwari^1^, A. Rauf^2^, S. Kesavan^3^, M. Kappanayil^4^, S. Sivadas^5^, A. V^6^, A. Vijayan^2^, P. Chickermane^1^, S. Balan^1^

##### ^1^Clinical Immunology and Rheumatology, Amrita Institute of Medical Sciences,Kochi,Kerala,India., Kochi; ^2^Pediatrics, Baby Memorial Hospital, Kozhikode; ^3^Pediatric pulmonology and critical care; ^4^Pediatric cardiology; ^5^Pediatrics; ^6^Microbiology, Amrita Institute of Medical Sciences,Kochi,Kerala,India., Kochi, India

###### **Correspondence:** A. Tiwari

**Introduction:** Multisystem inflammatory syndrome in children (MIS-C) is a rare association of coronavirus disease 2019 (COVID-19) infection in children. MIS-C presents as a hyperinflammatory syndrome with fever, gastrointestinal and mucocutaneous symptoms, features of incomplete Kawasaki disease, and macrophage activation syndrome. As it is a new disease there is a paucity of literature on medium-term and long-term outcomes hence this study was planned.

**Objectives:** To study medium-term outcomes in cases of MIS-C from two tertiary care centers from south India.

**Methods:** MIS-C cases diagnosed from March 2020 to February 2021, who had completed three months from acute illness of MIS-C by May 2021, were enrolled in the study. At the time of follow-up at three months, their baseline characteristics, clinical features, laboratory parameters, echocardiographic findings, and ongoing treatment were noted.

**Results:** A total of 37 MIS-C cases had completed 3 months follow-up by April 2021 from both centers. Residual sequalae were cardiac and detected only on follow-up echocardiography. All discharged patients were clinically well and echocardiographic changes were also on improving trend. However, 6 (16%) patients had some echocardiographic changes. Coronary abnormalities were noted in a total of 4 (11%) cases; coronary dilation and small coronary aneurysm were noted in one patient each and two patients had hyperechoic/non-tapering coronaries. Left ventricular dysfunction and pulmonary arterial hypertension were present in one patient each. Patients with persistent coronary abnormalities were on Aspirin and the other two patients required treatment of left ventricular dysfunction and pulmonary arterial hypertension respectively.

**Conclusion:** Medium-term outcomes in MIS-C patients are favorable; provided optimum treatment, follow up with clinical, laboratory, and echocardiographic assessment. Sequalae at 3 months follow up were cardiac and were only detectable by echocardiography, this emphasizes the need for subsequent echocardiogram during follow-up periods to decide the appropriate treatment. All echocardiographic parameters were on improving trends hence these cases are required to be followed up for long-term outcomes.

**Disclosure of Interest**

None declared

### P391 COVID-19 in children with rheumatic diseases - experiences of a single centre

#### N. Toplak^1^, M. Zajc^2^, A. Koren Jeverica^2^, T. Vesel Tajnšek^2^, N. Emeršič^2^, Š. Blazina^2^, G. Markelj^2^, T. Avcin^1^

##### ^1^Department of Allergology, Rheumatology and Clinical Immunology, University Medical Centre Ljubljana, Faculty of medicine, University of Ljubljana; ^2^Department of Allergology, Rheumatology and Clinical Immunology, University Medical Centre Ljubljana, Ljubljana, Slovenia

###### **Correspondence:** N. Toplak

**Introduction:** To date, there are few data on SARS-CoV-2 infection in children with rheumatic diseases (RD), including children treated with immunosuppressive therapy.

**Objectives:** To investigate the clinical presentation of SARS-CoV-2 infection in children with RD, whether it affects the disease activity, and if therapy affects the serological response to SARS-CoV-2 in the long term.

**Methods:** This is a partially retrospective study with prospective follow-up of disease activity and serology after SARS-CoV-2 infection. We collected children with RD, treated at UCH Ljubljana, Slovenia, who were exposed to SARS-CoV-2. If a member of the same household had a confirmed SARS-CoV-2 infection, we performed a serology test even if the patient had no symptoms. We collected demographical data, clinical presentation of infection, diagnosis of RD, therapy and disease activity at the time of infection and location of the contact with SARS-CoV-2. Serology for SARS-CoV-2 was tested several times during the follow-up.

**Results:** From July 2020 to May 2021, we identified 36 children with RD, who had been exposed to SARS-CoV-2 (31 female; median age 14.8 years, range 2.5-23 years). The majority (28/36; 77%) had juvenile idiopathic arthritis.

At the time of SARS-CoV-2 infection 14/36 (39%) children were taking TNFα inhibitors (TNFαi), 4 in combination with methotrexate (MTX), 5/36 MTX only, 2/36 cyclosporine, 2/36 mycophenolate mofetil, 2/36 low dose methylprednisolone, 1/36 azathioprine, 1/36 baricitinib, 1/36 anakinra, 1/36 abatacept, 1/36 tocilizumab, 1/36 NSAID, and 5/36 children were in remission without therapy.

Seven children (7/36; 19%) had a moderate disease course of COVID -19 (fever, musculoskeletal pain, headache, fatigue). Eight children (8/36; 22%) had no signs at all, but serology was performed in 7 and was positive. A total of 10/36 children (27%) lost the sense of smell and taste. Among them, 2 had no other signs. No child needed hospitalization.

In the group of children with moderate COVID -19 infection (4/7 female; median age 15 years, range 11-21 years), 3 were treated with TNFαi, 2 with MTX, 1 with baricitinib, and 1 with NSAIDs.

One child had recurrence of uveitis 5 months after infection. So far, no other relapse had been noticed.

Contact was known in 27/36 (75%) children and 24/36 (88%) got the virus at home. Only in 9/36 children (25%) the smear was taken and was positive. Serology was determined in 11 children in the first 3 months after infection and in 6 children later than 3 months after infection. In 4 children, serology test had already been performed twice. Only one child treated with adalimumab at the time of the acute infection lost protection at 5 months, while children treated with baricitinib, anakinra and methylprednisolone still had protective antibodies 5-6 months after acute infection.

**Conclusion:** From these preliminary results it appears that children with RD, regardless of the therapy they are taking, are not at serious risk for SARS-CoV-2 infection or for disease relapse. It is possible that the therapy they are taking affects the serological response in the long term, but this needs to be clarified in the future.

**Patient Consent Received**

Yes

**Disclosure of Interest**

None declared

### P392 Clinical course of COVID-19 in children with rheumatic disease under biologic therapy

#### F. Demir, K. Ulu, S. Çağlayan, T. Coşkuner, B. Sözeri

##### Department of Pediatric Rheumatology, Umraniye Training and Research Hospital, Istanbul, Turkey

###### **Correspondence:** K. Ulu

**Introduction:** Comorbidities and immunosuppression are known as the risk factors for the worse course of COVID-19 infection. However, it has not been shown exactly how biological disease-modifying anti-rheumatic drug (bDMARD)s, used frequently in our pediatric rheumatology practice, and the underlying rheumatological diseases affect the clinical course of COVID-19. Emerge of serious infections have been reported in adults and children treated with bDMARDs.

**Objectives:** Here, we aimed to reveal the outcome of COVID-19 infection in our patients with pediatric rheumatic disease and treated with bDMARDs.

**Methods:** During the period between April 1, 2020, and December 1, 2020, the patients who received bDMARDs (N:436) were evaluated at the regular outpatient clinic follow-up or by telemedicine with a maximum of three-month intervals. Clinical and demographic characteristics, COVID-19 data, and outcomes of these patients were retrospectively collected.

**Results:** Out of the 436 patients treated with bDMARDs, 39 children were infected with COVID-19. The diagnosis was confirmed in 37 patients by RT-PCR (nasalpharyngeal swab) and in twoby an antibody test. All patients were under biological treatment when they were diagnosed with COVID-19 or MIS-C.

Twenty-two(56.4%) patients were female (17 male, %43.6) and the median age of patients were 12.3 years (min-max:1.2-20.9). The primary diagnosis of patients were as follows; 20 juvenile idiopathic arthritis (JIA), 12 systemic autoinflammatory disease (SAID)s, three vasculitis, three chronic recurrent multifocal osteomyelitis (CRMO) and one Sjögren’s syndrome. Prior to COVID-19 infection, 13 patients(33.3%) were using canakinumab, seven were on infliximab(18%), five were on adalimumab(12.8%), four were on etanercept (10.2%), four were on tocilizumab(10.2%), three were on anakinra(7.7%), two were on rituximab(5.1%), and one was on tofacitinib(2.6%). Additionally, 14 patients were using conventional DMARD, 12 were colchicine, two were mycophenolate mofetil and one was cyclosporine.

Of the 39 patients, 21 had at least one COVID-19-related symptom, while 18 patients were asymptomatic. Laboratory tests revealed that fourteen patients had elevated acute phase reactants, six had elevated D-dimer levels, three had lymphopenia (<1000/mm^3^), and three had hyperferritinemia. Viral pneumonia compatible findings were detected in three of nine patients who underwent computed tomography.

Hospitalization was required in 20 patients(51.3%) at median of 7-days (min-max: 3-17) and pediatric intensive care unit admission in one. Five patients developed MIS-C and one of these patients was followed up in the pediatric intensive care unit. Myocardial dysfunction was developed in one patient and he died despite treatments. This patient had a previous case of macrophage activation syndrome (MAS) attack and was diagnosed with systemic JIA. The other four patients fully recovered with no remaining morbidity.

**Conclusion:** Considering the literature data and the results of our study, it is not possible to say that currently used bDMARDs worsen the course of COVID-19 infection. Whether bDMARDs does not affect the severity of the disease, but it is still not true to say that these drugs are protective. Since the cessation of bDMARDs for COVID-risk may cause exacerbation of the primary rheumatic disease, continuing with current treatments seems an appropriate approach. In a patient treated with bDMARD during the active COVID-19 infection period, it may be considered to interrupt the biological treatment on a patient basis by the current biological agent, primary disease status, and clinical findings of COVID-19 infection, according to ACR and PReS recommendations

**Trial registration identifying number:** (Approval no/date: B10.1TKH.4.34.H.G.P.0.01/101, 25.05.2020).

**Patient Consent Received**

Yes

**Disclosure of Interest**

None declared

### P393 Quantitative, semi-quantitative and qualitative nailfold videocapillaroscopy assessment of microvascular involvement in pediatric multisystem inflammatory syndrome temporally associated with SARS-COV-2

#### A. V. Villarreal Treviño, F. García Rodríguez, K. V. Jirón Mendiola, R. C. Calderón Zamora, M. de la O Cavazos, N. Rubio Pérez

##### Pediatric Rheumatology, Hospital Universitario “José Eleuterio González”, Monterrey, Mexico

###### **Correspondence:** A. V. Villarreal Treviño

**Introduction:** Nailfold videocapillaroscopy (VCP) is an in vivo, non-invasive, rapid, and inexpensive imaging technique that allows quantitative assessment of microcirculation. Pediatric Multisystem inflammatory syndrome in children temporally associated with COVID-19 (MIS-C) affects small, medium and large vessels. Nailfold capillaroscopy findings as pericapillary edema, meandering capillaries and reduced capillary density has been described in adults patients with COVID-19. Case report shows non specific alterations however quantitative microcirculatory impairment has not been clearly documented in MIS-C.

**Objectives:** To describe qualitative and semi-quantitative and quantitative nailfold videocapillaroscopy assessment in Mexican patients with pediatric Multisystem Inflammatory Syndrome temporally associated with SARS-CoV-2.

**Methods:** The present study was a cross-sectional observational study that analyzed 40 images from 5 patients with Pediatric Multisystem Inflammatory Syndrome according the case definition published by Cattalini et al. VCP was performed by the same examiner (AVT), images were obtained from all fingers except thumbs of both hands using a videocapillaroscope equipped with a 200x optical probe. The images were collected, coded, and stored using OptiPix software (version 1.7.16), 2015 Optilia Instruments. Qualitative and semi-quantitative assessment were realized according Cutolo classification. Quantitative assessment consist in the measurement of the number of capillaries per millimeter, capillary loop length, capillary width in micron (μm).

**Results:** 2 patients had coronary abnormalities, 1 valvular insufficiency, 1 myocarditis all patients received intravenous Immunoglobulin. 40 images of 5 patients with MIS-C (Image 1) were included in this series, all patients with Hispanic ethnicity and 60% male (n=5). The qualitative assessment did no reveal images with normal capillaroscopy pattern, nonspecific alterations were found in 100%, no pictures suggestive of sleroderma pattern-like have been observed. The semi-quantitative and quantitative assessment.

Lower capillary density (4.8 capillaries/mm), increased capillary width in all patients 2^nd^ Left medium 24.6μm, moderate edema in 90% (n=36) of images, altered architecture 75% (n=30), peri-capillary stripping hemosiderin deposits probably due capillary leak 60% (n=24), tortuous capillaries 40%(n=16), bizzare capillaries 40% (n=16), meandering 75%(n=30) and irregular ectasias 90% (n=36).

**Conclusion:** This study found qualitative and quantitative abnormalities in VCP suggesting systemic microvascular damage. Further studies are needed to asses the clinical relevance of VCP and its relationship with cardiac alterations and multisystemic damage in MIS-C.

**Patient Consent Received**

Yes

**Disclosure of Interest**

None declared

### P394 Characteristics of MIS-C patient cohort in Slovenia and distinguishing features to other febrile illnesses

#### M. Zajc Avramovič^1,2^, K. Vincek^3^, T. Plankar^3^, T. Avcin^1^

##### ^1^Department for Allergology, Rheumatology and Clinical Immunology,, University Children Hospital Ljubljana, Slovenia; ^2^Medical Faculty, University of Ljubljana; ^3^Clinic for Infectious Diseases, UMC Ljubljana, Ljubljana, Slovenia

###### **Correspondence:** T. Avcin

**Introduction:** Multisystem inflammatory syndrome in children (MIS-C) was recognized during the 2020 pandemic of SARS-CoV-2 and knowledge is still limited.

**Objectives:** To report the characteristics of patients with MIS-C and in a cohort study in Slovenia and report distinctions in regard to patients with suspected MIS-C but a different final diagnosis.

**Methods:** This is a prospective cohort study of consecutive patients with suspected MIS-C, admitted from March 2020 to January 2021 to University Medical Centre Ljubljana, Slovenia. The inclusion criteria for the study was suspected MIS-C on referral. Inclusion criteria for MIS-C group was meeting the WHO criteria and serology was used to confirm SARS-CoV-2 infection.

**Results:** MIS-C group. 23 patients (14male, median age 12.4 yrs), all Caucasian were enrolled and prevalence of MIS-C was 5.8/100 000 persons younger than 19 years of age. Detailed analyses were available in 20 patients. Two patients were treated in ICU and none died. Four patients had symptomatic SARS-CoV-2 infection, all had positive serology. Troponin was elevated in 15/20(75%) patients during the disease course, 7/15(47%) of these had normal level at admission. Six patients (30 %) had elevated pancreatic enzymes, 1 patient developed asymptomatic acute pancreatitis (max serum lipase 25mkat/L). All had elevated levels of D-dimer with no signs of thrombosis. All patients received IVIG and systemic corticosteroids. Four patients (20%) received high dose methylprednisolone pulse therapy. Biologic therapy with anakinra was started in 2 patients. Nineteen patients (19/20, 95%) received acetylsalicylic acid and prophylactic anticoagulation was prescribed in 15/20 (75%) of patients. The mean follow up was 50 days (14 – 122). At the last follow-up visit all patients had normal laboratory parameters of inflammation, troponin, pro-BNP, d-dimer values and normal heart function. Non MIS-C group. Thirteen patients (6M, median age 7.5 years), referred as MIS-C and later diagnosed with a different disease were included. The final diagnoses were: Campylobacter gastroenterocolitis (3), staphiloccocal sepsis (2), urinary tract infection (2), renal abscess (1), appenditicis (1), adenovirus tonsilopharyngitis (1), streptococcal faringitis (1), pancreatic mass (1) and TNF receptor-associated periodic syndrome (1). Group characteristics are shown in Table 1. The main differences between the groups hystory of SARS-CoV-2 infection, lip/mouth changes, conjunctivitis and later the presence of myocarditis. The initial inflammatory parameters did not differ between groups, but in the MIS-C group significantly lower values of platelets, sodium and albumins and higher values of troponin, pro-BNP and ferritin were noted at admission.

**Table:** 

	MIS-C* (20 patients)	Non MIS-C* (13 patients)	P value
Fever	20 (100)	12 (92)	0,207
GIT symptoms	19 (95)	11 (85)	0,310
Skin/mucous symptoms	14 (70)	6 (46)	0,170
- **Lip and mouth**	12 (60)	3 (23)	**0,037**
- **conjunctivitis**	14 (70)	4 (30)	**0,027**
**Cardiac involvement**	20 (100)	0 (0)	**<0,0001**
CRP (mg/L)**	140 [29; 341]	137 [28; 231]	0,806
**Platelets (10**^**9**^/L)**	160 [65; 605]	264 [162; 499]	**< 0.001**
Sodium (mmol/L)**	133 [127;141]	137 [132; 143]	**0,007**
Ferritin **	788 [40; 2824]	182 [9; 486]	**0,001**
Pro-BNP**	2943 [12.9; 19224]	548 [10; 1654]	**0,034**

* n (%) **mean value [min;max]

**Conclusion:** A very high incidence of MIS-C, estimated 5.8/100 000 persons under the age of 19 with a predominantly cardiac involvement but very good outcome was noted in European Caucasian population in Slovenia. Attention to newly described pancreatic involvement should be raised. Very different diseases can have a similar presentation at onset and attention should be payed to the specifics of the condition.

**Patient Consent Received**

Yes

**Disclosure of Interest**

None declared

## Late-breaking lightning talks

### LB1 Outcomes of multisystem inflammatory syndrome in children temporally related to COVID-19: a longitudinal study

#### N. K. Bagri, on behalf of Rakesh Kumar Deepak,Suneeta Meena, Saurabh Kumar Gupta, Satya Prakash, Kritika Setlur, Jagatshreya Satapathy, Karan Chopra, Ashish Datt Upadhyay, Sivasubramanian Ramakrishnan, Rakesh Lodha, Lalit Dar, Anjan Trikha, Sushil Kumar Kabra

##### Division of Pediatric Rheumatology, Department of Pediatrics, All India Institute of Medical Sciences, New Delhi, India, New Delhi, India

###### **Correspondence:** N. K. Bagri

**Introduction:** The spectrum of clinical manifestations of COVID-19 in children is expanding since the global emergence of the COVID-19 pandemic from early reports in January 2020 depicting respiratory distress to a severe multisystem inflammatory syndrome (MIS-C) within various pediatric clusters. There is a paucity of data from resource-poor countries with respect to follow-up outcomes, particularly for coronary artery abnormalities. Considering this, we conducted a single centre prospective longitudinal study to describe the clinical, laboratory,echocardiographic findings and follow-up of children with MIS-C.

**Objectives:** To study the clinical and laboratory characteristics and outcomes of multisystem inflammatory syndrome in children (MIS-C) temporally related to COVID-19.

**Methods:** All children meeting the WHO case definition of MIS-C were prospectively enrolled. Baseline clinical and laboratory parameters were compared between survivors and non-survivors. Enrolled subjects were followed up for 4-6 weeks for evaluation of cardiac outcomes using echocardiography. The statistical data were analyzed using the SPSS version 12 software.

**Results:** 31 children with MIS-C were enrolled in an eleven-month period. Twelve children had preexisting chronic systemic comorbidity. Fever was a universal finding; gastrointestinal and respiratory manifestations were noted in 70.9% and 64.3%, respectively, while 57.1% had a skin rash. Fifty-eight % of children presented with shock, and 22.5% required mechanical ventilation. The median (IQR) duration of hospital stay was 9 (6.5-18.5) days. Four children with preexisting comorbidities succumbed to the illness. The serum ferritin levels (ng/ml) [median (IQR)] were significantly higher in non-survivors as compared to survivors [1061 (581,2750) vs 309.5 (140,720.08), p value=0.045] (table 1). Six children had coronary artery involvement: 5 recovered during follow-up, while one was still admitted. Twenty-six children received immunomodulatory drugs, and five improved without immunomodulation. The choice of immunomodulation (steroids or intravenous immunoglobulin) did not affect the outcome (table 1).

**Conclusion:** Most children with MIS-C present with acute hemodynamic and respiratory symptoms. The outcome is favourable in children without preexisting comorbidities. Raised ferritin level may be a poor prognostic marker. The coronary outcomes on follow-up were reassuring.

**Trial registration identifying number:** Not applicable

**Disclosure of Interest**

None declared

**Table 1 (abstract LB1).** Comparison of clinical and laboratory parameters and treatment modalities based on mortality

Parameter	Survivors (n=27)	Non-survivors (n=4)	p-value
Age, Age in months, median (IQR)	96 (60,120)	130.5 (72,161)	0.26
Shock, n (%)	14 (51.85)	4 (100)	0.12
Gastrointestinal symptoms,n (%)	18 (66.66)	4 (100)	0.29
Respiratory symptoms, n(%)	17 (62.96)	4 (100)	0.29
Skin rash, n(%)	17 (62.96)	0	**0.032**
Pre-existing comorbidity, n(%)	8/27	4/4	0.189
***Laboratory parameters***			
Total Leucocyte Count, median (IQR)	13240 (7700,20000)	19685 (12450,27285)	0.51
Platelet count (x10^5^/mm^3^), median (IQR)	2.05 (1.5,3.46)	0.46 (0.17, 2.56)	0.098
Serum IL-6 (5.00-15.0 pg/mL), median (IQR)	41.84 (12.9,194) (n=19)	55.06 (27.06,328.76) (n=4)	0.21
Serum ferritin (12- 300 ng/ml), median (IQR)	309.5 (140,720.08)	1061 (581,2750)	**0.045**
NT-ProBNP (pg/mL), median (IQR) (<125pg/ml)	248.63 (24.66,3189.7) (n=13)	6078.29 (1500.04,10656.5) (n=2)	1.46
D-dimer (ng/ml), median (IQR) (0.00-255.00ng/ml)	1050 (741.05,5056) (n=17)	4285 (385,14035) (n=4)	0.16
**Immunomodulation therapy**			
Supportive care only (n=5)	5	0	0.137
Only IVIG (n=6)	5	1	
Only steroids (n=5)	4	1	
Steroid plus Tocilizumab (n=1)	0	1	
IVIG plus steroids (n=14)	13	1	

### LB2 Safety and immunogenicity of the BNT162B2 mRNA COVID-19 vaccine in adolescents with juvenile-onset autoimmune inflammatory rheumatic diseases

#### M. Heshin-Bekenstein^1,2^, O. Elkayam^2,3^, A. Ziv^4^, N. Toplak^5^, D. Hagin^2,6^, D. Kadishevich^4^, Y. Aviel Butbul^7,8^, E. Saiag^9^, S. Pel^3^, G. Shefer^10^, O. Sharon^10^, T. Freund^6^, Y. Uziel^2,4^

##### ^1^Pediatric Rheumatology, Tel Aviv Medical Center; ^2^Tel Aviv University; ^3^Rheumatology, Tel Aviv Medical Center, Tel Aviv; ^4^Pediatric Rheumatology, Meir Medical Center, Kfar Saba, Israel; ^5^Allergy, Rheumatology and Clinical Immunology, University Children's Hospital, Lubliana, Slovenia; ^6^Allergy and Clinical Immunology, Tel Aviv Medical Center, Tel Aviv; ^7^Pediatric Rheumatology, Rambam Medical Center; ^8^Technion School of Medicine, Haifa; ^9^Hospital Management, Information and Operation Branch; ^10^Endocrinology Metabolism and Hypertension, Tel Aviv Medical Center, Tel Aviv, Israel

###### **Correspondence:** M. Heshin-Bekenstein

**Introduction: :** The safety, efficacy, and immunogenicity data of the vaccine against COVID-19 for adolescents with Juvenile-onset Autoimmune Inflammatory Rheumatic diseases (AIIRD) is currently limited. Vaccinating the immunocompromised adolescents for COVID-19 is particularly valuable to protect this vulnerable population.

**Objectives:** To evaluate the safety and immunogenicity of the BNT162b2 mRNA vaccine in adolescents with AIRD treated with immunosuppressive medications compared with healthy adolescents.

**Methods:** This prospective multicenter study examined the safety and immunogenicity of the two-dose regimen BNT162b2 mRNA vaccine in adolescents aged 12-18 years diagnosed with juvenile-onset AIIRD including Juvenile Idiopathic Arthritis (JIA), connective tissues diseases (CTD) including systemic lupus erythematosus (SLE), systemic vasculitides and uveitis.

Patients were evaluated 2-10 weeks after the second dose of the vaccine. Safety and post-vaccination COVID-19 infection were evaluated, as well as disease activity prior and following the vaccine. Post vaccination serum IgG antibody levels against SARS-CoV-2 spike S1/S2 proteins were measured. Seropositivity was defined as IgG ≥15 binding antibody units (AU/ml). Anti-Nuclear (N) IgG antibodies were measured for evidence of past COVID-19 infection (level above 1.4RLU was considered positive).

**Results:** 71 adolescents with AIIRD patients and 28 controls from 2 countries, 4 centers, participated in the study. The most common diagnosis in the AIIRD cohort was JIA (N=27), followed by SLE (N=14). The mean disease duration was 5.1±4.48 years (N=70). A total 84.5% (N=60) of the patients were treated with immunomodulatory medications. Post vaccination disease activity remained stable in 96.88% of the adolescents with AIIRD, and post vaccination treatment change was made in the minority of the patients (N=3, 4.84%). Both patients and controls have tolerated the vaccine well, with minimal side effects. There were no severe adverse events in both groups. No post vaccination infection with COVID-19 was documented in both groups.

Seropositivity rate was 90.32% in adolescents with AIIRD and 100% in the healthy controls (N=28/31 vs. N=14/14; p=0.54). The level of the S1/S2 antibodies was significantly reduced in adolescents with AIIRD compared to controls (mean± SD 218.97±150.9 vs 380.78±71.89, P<0.0001). The N Index was negative in both adolescents with AIIRD (0.09±0.09, [N=15]) and healthy controls (0.054±0.036 [N=11]), indicating that none of these participants suffered from past COVID-19 infection.

**Conclusion:** In our cohort, the BNTb262 mRNA COVID-19 vaccine was shown to have excellent safety profile in immunocompromised adolescents with Juvenile-onset AIIRD, with mild post vaccination side effects, similar to the safety profile of the healthy controls. No post vaccination COVID-19 illness was documented. Post vaccination disease activity was mostly kept stable. Immunogenicity was very good in both groups, with significantly higher S antibody titers in the healthy controls.

**Consent**

I have obtained written consent

**Disclosure of Interest**

None declared

### LB3 SARS-COV-2 vaccination is tolerated following multisystem inflammatory syndrome in children (MIS-C)

#### T. Vogel^1,2^, A. Chun^1,2^, M. DeGuzman^1,2^, E. Muscal^1,2^, S. K. Sexson Tejtel^2,3^, F. Munoz^2,4^

##### ^1^Pediatrics-Rheumatology, Baylor College of Medicine; ^2^Texas Children's Hospital; ^3^Pediatrics-Cardiology; ^4^Pediatrics-Infectious Diseases, Baylor College of Medicine, Houston, United States

###### **Correspondence:** T. Vogel

**Introduction:** Most children who contract SARS-CoV-2 infection are asymptomatic or mildly symptomatic. However, a subset go on to develop a potentially life-threatening hyperinflammatory condition called multisystem inflammatory syndrome in children (MIS-C) 4-6 weeks after COVID-19. The mechanisms by which MIS-C occurs are not yet clear, resulting in hesitation to vaccinate this subset of children against SARS-CoV-2 due to concerns for a reoccurrence of hyperinflammation.

**Objectives:** To evaluate outcomes following SARS-CoV-2 vaccination in patients who were previously diagnosed with MIS-C after COVID-19.

**Methods:** Medical records of patients who were treated for MIS-C at our institution were retrospectively reviewed. Details for those who were subsequently vaccinated against SARS-CoV-2 were extracted.

**Results:** A total of 164 patients were treated for MIS-C between May 2020 and May 2021. 22 patients were 16 years of age or older and an additional 30 patients were age 12-15 years, resulting in a total of 52 patients eligible for SARS-CoV-2 vaccination. 10 (19%) of these patients were vaccinated using the Pfizer-BioNTech product in our COVID-19 vaccine clinic.

The age of the patients ranged from 12 to 17 years. 8 were male, and 8 were from racial/ethnic minority groups. All were generally healthy (3 asthma, 1 repaired congenital heart disease) prior to their MIS-C diagnosis.

The patients presented between July 2020 and February 2021 with a febrile illness, and fulfilled the case definition for MIS-C established by the Centers for Disease Control and Prevention, including all 10 having positive SARS-CoV-2 serologic testing and 9 with myocarditis or coronary changes (measured by troponin elevation and/or electrocardiographic or echocardiographic evidence). 8 presented in shock or with hypotension, and 6 were admitted to the intensive care unit (ICU), among which 3 required vasoactive medications and 2 intubation. ICU length of stay ranged from 2-15 days, and total hospital stay from 2-23 days. All 10 patients were treated with corticosteroids, 8 received intravenous immunoglobulin, and 5 anakinra. All patients had normal cardiac function without coronary artery dilation at the time of last cardiology follow-up.

The patients were vaccinated an average of 199 days from MIS-C hospitalization discharge (range 83 to 337 days). Thus far, a median of 57 days (range 1 to 117 days) has elapsed since 9 of the 10 patients completed the second vaccine dose. None has developed a recurrence of MIS-C or a hyperinflammatory condition. No significant adverse events have occurred following vaccination.

**Conclusion:** These 10 patients who experienced MIS-C after COVID-19 have tolerated vaccination against SARS-CoV-2 without the subsequent development of a similar hyperinflammatory state providing critical information as the COVID-19 pandemic continues to rage across the globe. As we move toward vaccination for children younger than 12 years, a growing number of prior MIS-C patients (average age 8-9 years) will become eligible for vaccination. Given the risk of re-infection with SARS-CoV-2 and the known additive protection from re-infection provided by vaccinating previously infected individuals, it is imperative that patients with a history of MIS-C be offered vaccination against SARS-CoV-2.

**Disclosure of Interest**

None declared

### LB4 MAS and lung disease as complications of HLA-linked severe delayed reactions to inhibitors of IL-1 and IL-6 in still’s disease

#### V. Saper^1^, M. Ombrello^2^, G. Montero-Martin^1^, S. Prahalad^3^, S. Canna^4^, L. Tian^1^, M. Fernandez Vina^1^, J. Hollenbach^5^, E. Mellins^1^ on behalf of the Drug hypersensitivity consortium, the INCHARGE consortium, and on behalf of the CARRA Registry Investigators

##### ^1^Stanford University, Stanford, CA; ^2^NIAMS, NIH, Bethesda, MD; ^3^Emory University, Atlanta, GA; ^4^Children's Hospital of Philadelphia, Philadelphia, PA; ^5^University of California San Francisco, San Francisco, CA, United States

###### **Correspondence:** V. Saper

**Introduction:** Drug reaction with eosinophilia and systemic symptoms (DRESS) is a severe delayed hypersensitivity reaction, which can include lung involvement.^1^ Previously, we described DRESS to inhibitors of interleukin 1 (IL-1) or interleukin 6 (IL-6) in a group of Still's patients developing atypical lung disease during treatment.^2^

**Objectives:** To characterize clinical and genetic features of Still’s patients with DRESS compared to drug-tolerant controls.

**Methods:** We conducted a multicenter, retrospective case/control study of 131 Still’s patients: n=66 with features of inhibitor-related DRESS and n=65 Still’s patients who were tolerant to these drugs. Additionally, we compared clinical data from drug-reactive cases and drug-exposed sJIA subjects in the Childhood Arthritis and Rheumatology Research Alliance (CARRA) registry (n=655). HLA-typing was available in 94/131 subjects. European descent Still’s-DRESS cases were ancestry-matched to INCHARGE pediatric Still's cases^3^ (n=550) and compared for HLA allele frequencies. HLA association also was analyzed using Still’s-DRESS cases (n=64) compared to drug-tolerant Still’s controls (n=30).

**Results:** In cases with HLA typing available, DRESS features included eosinophilia (89%), AST-ALT elevation (75%), and non-evanescent rash (95%; 88% involving face). Macrophage activation syndrome (MAS) during treatment was frequent in DRESS subjects (64%) compared to drug-tolerant controls [3%; p=1.9x10^-14^; OR(95%CI) 55.8 (12.4-250.7)] or to CARRA registry controls [2.9%; p=1.66 x 10^-32^; OR(95%CI) 48.7(25.0-95.0)]. We found striking enrichment for HLA-DRB1*15 haplotypes in cases versus INCHARGE controls [p=7.5x10^-13^; OR(95%CI) 55.8 (12.4-250.7) and versus self-identified, ancestry-matched drug-tolerant controls [p=6.3x10^-10^; OR (95%CI) Infinite (16.05-Inf)]. The HLA link to DRB1*15 alleles occurred in DRESS cases with (37/45) or without (14/19) lung involvement and across ancestries (51/64). Consistent with the overall US population frequency of these alleles (~20%^4^), the frequency of DRB1*15:01 in the European-descent INCHARGE sJIA cohort was 24% and 25% in INCHARGE healthy controls. In 9 cases followed for a median of 10 months (range 3-40) after drug withdrawal, significant improvement (symptoms and imaging) or resolution of lung disease occurred.

**Conclusion:** DRESS-type delayed reactions occur among Still’s patients treated with IL-1/IL-6 inhibitors and strongly associate with common HLA-DRB1*15 haplotypes across ancestries. These haplotypes are not associated with Still’s disease itself. MAS during drug treatment, a known feature of DRESS reactions^5^, should raise suspicion of DRESS to IL-1/IL-6 inhibitors in Still’s. Consideration of pre-prescription HLA typing and vigilance for serious delayed reactions is warranted. Additional investigation is required to assess lung disease outcome after withdrawal of the implicated drugs.

References

1. Taweesedt PT et al Pulmonary Manifestations of DRESS Syndrome: A Systematic Review. Biomed Res Int. 2019

2. Saper VE et al. Emergent high fatality lung disease in systemic juvenile arthritis. *Ann Rheum Dis* 2019;78(12)

3. Ombrello MJ et al. HLA-DRB1*11 and variants of the MHC class II locus are strong risk factors for systemic juvenile idiopathic arthritis. *Proc Natl Acad Sci U S A* 2015;112(52)

4. Maiers M et al. High-resolution HLA alleles and haplotypes in the United States population. *Hum Immunol* 2007;68(9)

5. Yang JJ et al. Overlap between hemophagocytic lymphohistiocytosis and drug reaction and eosinophilia with systemic symptoms: a review. *Int J Dermatol* 2020

**Consent**

I have obtained written consent

**Disclosure of Interest**

None declared

### LB5 Pediatric non infectious uveitis. Clinical outcome and treatment exposure. Results of real life clinical setting from Spanish pediatric non infectious uveitis registry

#### P. Mesa-Del-Castillo B.^1^, I. Yago Ugarte^2^, J. M. Bolarin^3^, B. López Montesinos^4^, D. Clemente Garulo^5^, J. C. Lopez Robledillo^6^, B. Bravo Mancheño^7^, C. Alba Linero^8^, E. Nuñez Cuadros^9^, M. C. Mir Perelló^10^, M. C. Pinedo Gago^11^, A. Souto Vilas^12^, C. Zarallo Reales^13^, N. Palmou Fontana^14^, V. Jovani Casano^15^, A. M. Brandy García^16^, B. Sevilla Perez^17^, A. Tarrago^18^, J. Calzada Hernandez^19^, C. Gavilán Martín^20^, S. Recuero Díaz^21^, N. Quilis Martí^22^, J. Rosas ^23^, J. C. Nieto González^24^, L. Ibares^25^, J. De Inocencio^26^

##### ^1^Pediatric Rheumatology; ^2^Pediatric Ophtalmology, Hospital Clínico Universitario Virgen de la Arrixaca; ^3^Centro Tecnológico de las tecnologías de la información y comunicaciones (CENTIC), Murcia; ^4^Pediatric Rheumatology, Hospital Universitario La Fe, Valencia; ^5^Pediatric Rheumatology; ^6^Reumatologia, Hospital Niño Jesús, Madrid; ^7^Pediatric Rheumatology, Hospital Virgen de las Nieves, Granada; ^8^Pediatric Rheumatology; ^9^Pediatrics, Hospital Materno Infantil Malaga, Malaga; ^10^Pediatric Rheumatology, Hospital Son Espases, Palma de Mallorca; ^11^Pediatric Rheumatology, Hospital Cruces, Bilbao; ^12^Pediatric rheumatology, Hospital Universitario Santiago, Santiago de Compostela; ^13^Reumatologia, Hospital Materno Infantil Badajoz, Badajoz; ^14^Reumatologia, Hospital Marques de Valdecilla, Santander; ^15^Reumatologia, Hospital Universitario Alicante, Alicante; ^16^Pediatric rheumatology, Hospital Cabueñes Gijón, Gijón; ^17^Pediatric rheumatology, Hospital San Cecilio, Granada; ^18^Pediatrics, Hospital Infanta Sofía, Madrid; ^19^Pediatric rheumatology, Hospital San Joan de Deu, Barcelona; ^20^Pediatrics, Hospital San Juan, Alicante; ^21^Reumatologia, Fundación Jiménez Díaz, Madrid; ^22^Reumatologia, Hospital Universitario Vinalopó, Elche; ^23^Reumatologia, Hospital Marina Baixa, Villajoyosa; ^24^Pediatric rheumatology; ^25^Ophtalmology, Hospital Gregorio Marañón; ^26^Pediatric rheumatology, Hospital 12 de Octubre, Madrid, Spain

###### **Correspondence:** P. Mesa-Del-Castillo B

**Introduction:** Pediatric noninfectious uveitis is a mayor challenge for Pediatric ophthalmologist and rheumatologist.

**Objectives:** Describe a large cohort of spanish pediatric onset uveitis.

**Methods:** A cross sectional and retrospective cohort study was conducted from october’19 to march’21. Multidisciplinary participation was mandatory for entering the study and only fully completed patients were included for analysis. Three distinct groups were made for comparison: Juvenile Idiopathic Arthritis related uveitis (JIAu), Idiopathic childhood uveitis (ICU) and Pars Planitis (PP).

**Results:** A total of 501 patients from 20 centers were included in the analysis, 310 (61.9 %) with an associated immune disease, 91 (18.2%) ophthalmologicaly defined and 100 (20%) idiopathic. JIAu accounted for 54.8 % (275), 259 (51.6%) non B27 related; 67 patients (13.3%) had PP. JIAu was significantly associated to females, younger at uveitis onset, ANA positive, anterior location and insidious presentation, with less ocular complications, systemic corticoid use and ocular surgeries. DMARD exposure was longer and started earlier after uveitis onset with less unilateral severe visual loss. ICU tended to present as panuveitis an associated to males, older age at onset, ANA negative, panuveitis, acute presentation and acute clinical course. Ocular complications were higher, mainly synechiae at onset and cataracts. Exposition to DMARD was significantly lower for both synthetic and biologic agents. PP showed the highest complication rate within the groups (71.6%), specially cataracts during the follow up and greater tendency to present vitreoretinal complications (macular edema, epiretinal membrane and retinoschisis), requiring ocular surgery more frequently. These patients received more systemic steroids, but no difference in regard to DMARD use rate.

**Conclusion:** Our data suggest that differences in outcome may correspond partially to differences in treatment approach depending on the origin of the uveitis. Surprisingly, JIAu patients sowed the best prognosis, despite its chronicity, in comparison with the other groups, traditionally considered mild self-limited diseases. Awareness of ophthalmologists and rheumatologists on these observations may ameliorate the burden of disease by inducing rheumatology referral for early treatment.

**Consent**

I have obtained written consent

**Disclosure of Interest**

None declared

